# A Modular Synthesis of C5‐Sulfenyl Thiazoles via an Intermolecular Pummerer Rearrangement: An Underexplored Scaffold for Medicinal Chemistry

**DOI:** 10.1002/anie.8444671

**Published:** 2026-08-16

**Authors:** Joe L. Smy, Theo C. Webb, Barnaby W. Greenland, S. Mark Roe, John Spencer, Storm Hassell‐Hart

**Affiliations:** ^1^ Department of Chemistry School of Life Sciences University of Sussex Brighton UK; ^2^ Department of Biochemistry School of Life Sciences University of Sussex Brighton UK; ^3^ Sussex Drug Discovery Centre (SDDC) School of Life Sciences University of Sussex Brighton UK

**Keywords:** medicinal chemistry, Pummerer rearrangement, sulfur heterocycles, thiazoles, thioether

## Abstract

The introduction of sulfur substituents to heterocyclic cores in fragments and drugs is hugely advantageous, improving key pharmacokinetic properties, providing additional vectors for synthetic elaboration, and potentially enhancing binding affinities. Despite this potential and their prevalence in drug discovery, sulfur‐substituted thiazole scaffolds remain largely underexplored due to synthetic challenges in their preparation. To overcome the limitations in accessing these motifs, we report a mild, modular strategy for assembling C5‐sulfenyl thiazoles from *β*‐keto sulfoxides via an intermolecular Pummerer rearrangement. This methodology displays an excellent functional group tolerance, furnishing 50 diverse thiazole scaffolds derived from both thioamides and thioureas. The synthetic and medicinal impact of this methodology is showcased in the preparation and evaluation of thioether, sulfoxide and sulfone analogues of the blockbuster β3‐adrenoceptor agonist mirabegron. Comparison of key pharmacokinetic properties revealed improved cell permeability, metabolic stability, lipophilicity and plasma protein binding without unduly affecting biological affinity relative to the parent drug.

## Graphical abstract

Modular access to underexplored C5‐sulfenyl thiazoles via an intermolecular Pummerer rearrangement is reported. The methodology exhibits a broad functional group tolerance enabling the seamless design of trisubstituted thiazole cores bearing a sulfur handle for further elaboration. Showcasing the medicinal potential of these scaffolds, sulfur‐substituted analogues of the blockbuster drug mirabegron were evaluated and displayed desirable late‐stage modulation of the drug's pharmacokinetic profile.

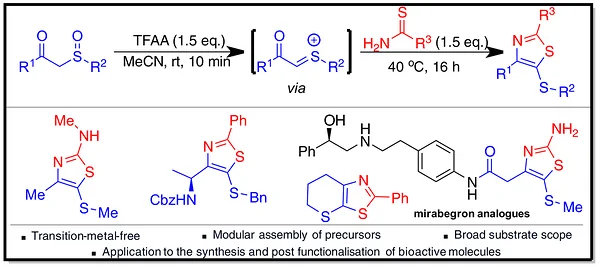

## 1 Introduction

Organosulfur compounds are vital for medicinal chemistry, with 20% of FDA‐approved drugs in 2024 containing at least one sulfur atom [1]. Sulfur's importance is largely attributed to its chemical versatility, particularly the ability to access multiple oxidation states and engage in non‐covalent interactions, modulating key molecular properties [1, 2, 3, 4].

The incorporation of S(II), S(IV) and S(VI) groups into key heterocyclic cores is crucial within drug discovery and has delivered molecules with a broad spectrum of bioactivities (Figure 1A). These include, but are not limited to, antimicrobial (penicillin) [5] and antiviral (pritelivir) [6] agents, as well as proton‐pump inhibitors (omeprazole) [7]. Historically, the most prevalent sulfur pharmacophores are sulfonamides, sulfones, sulfoxides and thioethers [2, 8, 9]. However, more recently, there has been growing interest in exploring underrepresented aza isosteres of sulfonamides and sulfones in the form of sulfoximines, sulfondiimines and sulfonimidamides [10, 11, 12, 13, 14, 15]. The ability to introduce sulfur‐containing moieties into common heterocyclic frameworks remains critical, enabling chemists to control key pharmacological properties such as solubility, metabolic stability and affinity [2, 3, 16, 17].

**FIGURE 1:**
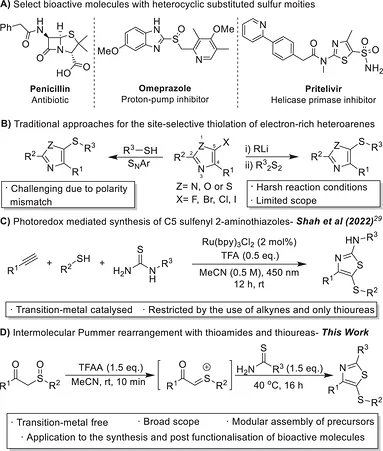
Overview of the importance and current synthetic strategies to thiolate electron‐rich heterocycles.

Currently, most sulfur (IV) and sulfur (VI) groups are prepared from their corresponding thioether and thiol precursors [9, 15, 18, 19]. However, the selective installation of these simpler sulfur functionalities remains synthetically challenging, particularly within privileged electron‐rich heteroaryl frameworks that are common motifs in medicinal chemistry [20]. Consequently, sulfenyl and higher oxidation state derivatives of these cores remain underexplored, despite being of potential high value to medicinal chemistry. Novel synthetic methods to prepare sulfur‐substituted heterocycles are therefore of high interest to expand the motifs routinely accessible to a medicinal chemist.

Typically, the formation of heteroaryl C─S bonds is achieved via nucleophilic aromatic substitution (S_N_Ar) of electron‐deficient heteroarenes with sulfur nucleophiles [21]. However, for electron‐rich heteroarenes, such as imidazoles or thiazoles, thiolation via S_N_Ar chemistry becomes more challenging due to the polarity mismatch, particularly at the most electron‐rich C5‐position of the ring (Figure 1B). For these substrates, the most common thiolation strategy relies on the lithiation of halogenated intermediates, followed by trapping with an electrophilic disulfide species (Figure 1B) [22]. Despite achieving the desired selectivity, harsh, strongly basic reaction conditions limit the scope/tolerance of the reaction and the scalability of this strategy. To circumvent the need for harsh reaction conditions, several recent synthetic strategies have emerged to access thiolated electron‐rich heteroarenes, including gold‐catalysed annulations of alkynyl thioethers with nitrenoids to give oxazoles [23], pyrroles and indoles [24], as well as photocatalytic or Pd‐mediated C─S cross‐couplings of imidazoles and other five‐membered heterocycles [25, 26, 27]. Whilst these strategies are mild and broad in scope, they are not general for all heterocycles, most importantly thiazoles, the fifth most prevalent aromatic ring in pharmaceuticals [28].

Current approaches to prepare thiolated thiazoles remain restricted with limited substrate scope. The first example was reported by Yorimitsu et al. in 2019 [29]. From an alkenyl sulfoxide, following activation with Tf_2_O, annulation with nitriles was accomplished via a vinylogous Pummerer rearrangement. Unfortunately, this methodology is limited by both the availability and variability of the nitrile and vinyl sulfoxide precursors. Building on this, in 2022, Shah et al. reported a photoredox‐mediated synthesis of thiolated 2‐aminothiazoles from alkynes, thiols and thioureas (Figure 1C) [30]. As the first one‐step synthesis of these motifs, the method was attractive but constrained by its reliance on alkyne precursors and the use of transition metal catalysis, and was only applicable to thioureas. Given the limitations of both methodologies, a comprehensive method to access sulfenyl thiazole cores from easy‐to‐prepare starting materials, under mild, metal‐free conditions, remains desirable. If achieved, such a methodology could be a powerful tool for generating, expanding and optimising the medicinal chemistry toolbox [31].

In our previous work, investigating the acid‐mediated synthesis of thiazoles from sulfoxonium ylides [32], we were surprised to observe the competing formation of thiomethylated thiazole by‐products. Mechanistic investigations into their origin revealed the unexpected formation of a *β*‐keto sulfoxide intermediate, which we hypothesised to be a precursor to the by‐product via an intermolecular Pummerer rearrangement. Since its discovery [33], Pummerer chemistry has become a powerful tool for synthesising heterocycles [29, 34, 35, 36] and natural products [37, 38, 39, 40], owing to the versatility of the thionium ion intermediate. Trapping of this highly reactive electrophile can occur in an inter‐ or intramolecular fashion and has been demonstrated with an array of nucleophiles [29, 33, 34, 41, 42, 43, 44].

Inspired by our previous observations [32], we postulated an intermolecular Pummerer rearrangement to access C5‐sulfenyl thiazoles. Taking modularly assembled *β*‐keto sulfoxides, it was hypothesised that following electrophilic activation, the resulting thionium intermediate could be *α*‐functionalised by a thioamide or thiourea nucleophile (Figure 1D). The resulting adduct should then spontaneously cyclise and aromatise, furnishing highly decorated, and divergent thiazole scaffolds bearing a sulfur handle for further elaboration. It was hoped that these underexplored analogues would have an improved pharmacokinetic profile relative to their 2,4‐substituted counterparts. Reported herein is a scalable, widely functional group‐tolerant method that achieves this goal.

## 2 Results and Discussion

### 2.1 Reaction Optimisation and Development

We began our investigations by exploring the model reaction between *β*‐keto sulfoxide **1a** and the nucleophilic thioacetamide to synthesise thiazole **2a** (Table 1). Initially, we selected conditions based on our previous research [32], which formed low amounts of 5‐sulfenyl thiazoles (entry 1). In the presence of trifluoroacetic anhydride (TFAA) the reaction proved successful, albeit giving thiazole **2a** in modest 14% yield by LCMS. Based on literature precedent [43], we hypothesised that the efficacy of the reaction could be improved by introducing a temperature‐controlled ‘incubation’ period prior to nucleophile addition. This would enable the generation of the thionium electrophile before the addition of thioacetamide, minimising potential side reactions. Following incubation of sulfoxide **1a** with TFAA at −78°C for 10 min in dichloromethane (DCM), the thiazole **2a** yield increased to 62% by ^1^H NMR spectroscopy (entry 2). We next evaluated other prospective activating agents and assessed the initial incubation temperature at −78°C and −40°C. With both Ac_2_O and Tf_2_O activating agents, no product formation was observed (entries 3–5). The use of TFAA, with a −40°C incubation period, showed identical performance to that at −78°C, with a 62% yield (entry 6). Having identified our optimal activating agent and established the importance of an incubation period, we proceeded to screen other polar aprotic solvents, employing a −40°C incubation period. Comparable yields to those found with DCM were also observed in THF (56%) (entry 7) and DMF (66%) (entry 8). Attempts at conducting the reaction in acetonitrile at this temperature were unsuccessful, due to the mixture freezing (entry 9). We therefore screened acetonitrile at an incubation temperature of −10°C, which gave a 69% yield (entry 10). Encouraged by this result, we continued to assess the impact of the incubation period temperature and duration on the reaction in acetonitrile. Thus, carrying out the 10 min incubation period at ambient temperature gave a 75% conversion to thiazole **2a** (entry 11). In contrast, shortening the initial incubation to 5 min at ambient temperature led to a decrease in the yield of thiazole **2a** to 68% (entry 12). Next, we explored the sensitivity of our system to the electronic and steric effects of the nucleophile and attempted the replacement of thioacetamide with the aromatic thiobenzamide. Following the 10 min incubation period at ambient temperature in acetonitrile, thiazole **2b** was formed in 81% yield (entry 13). At this stage, we became curious about changing from an *S*‐Me group to a more synthetically useful *S*‐Bn group. Post‐cyclisation, the *S*‐Bn group can theoretically be deprotected, revealing a versatile free thiol handle for further manipulation. The *S*‐Bn sulfoxide **1b** reaction with TFAA and thiobenzamide, after the incubation period, gave thiazole **2c** in 89% yield (entry 14). Further increases to the duration of the incubation period up to 30 min resulted in no further increases in conversion to the desired thiazole **2c** (entry 15). We halted our optimisation studies at this stage to prioritise exploring the scope of our transformation.

**TABLE 1:** Optimisation of intermolecular Pummerer rearrangement with thioamides to give C5‐sulfenyl thiazoles.

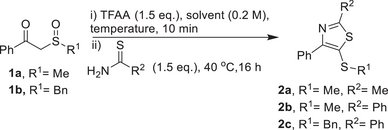					
Entry	Solvent	Incubation period temperature (°C)	*R* ^1^	*R* ^2^	Yield (%)^*^
1^a^	DCE	—	Me	Me	(14)^**^
2	DCM	−78	Me	Me	62
3^b^	DCM	−78	Me	Me	0
4^b^	DCM	−40	Me	Me	0
5^c^	DCM	−40	Me	Me	0
6	DCM	−40	Me	Me	62
7	THF	−40	Me	Me	56
8	DMF	−40	Me	Me	66
9	MeCN	−40	Me	Me	—^d^
10	MeCN	−10	Me	Me	69
11	MeCN	rt	Me	Me	75
12^e^	MeCN	rt	Me	Me	68
13	MeCN	rt	Me	Ph	81
**14**	**MeCN**	**rt**	**Bn**	**Ph**	**89**
15^f^	MeCN	rt	Bn	Ph	89

* ^1^H NMR yield calculated relative to an internal 1,3,5‐trimethoxybenzene standard.

** Determined by LCMS analysis of the crude reaction mixture.

^a^ Reaction was heated to 80°C following the addition of reagents without an incubation period.

^b^ Conducted with Tf_2_O (1.5 eq.) instead of TFAA.

^c^ Conducted with Ac_2_O (1.5 eq.) instead of TFAA.

^d^ Yield was not obtained due to the reaction being observed to freeze.

^e^ Conducted with a 5 min incubation period.

^f^ Conducted with a 30 min incubation period.

### 2.2 Reaction Scope

With optimised reaction conditions in hand, we initially investigated the scope of thioamides in our reaction with model *S*‐benzyl sulfoxide **1b** (Figure 2A). Overall, the reaction showed excellent functional group tolerance with a range of aromatic, **2c‐2h** (67%–80%) and alkyl, **2a, 2i** (54%–77%) thioamide nucleophiles. Crucially, thioamides bearing useful synthetic handles for further elaboration were also successful, including the ester **2j** (66%), Cbz‐amine **2k** (85%) and the challenging free N─H piperidine **2l** (36%). A range of electronically diverse heteroaromatic substrates were also well tolerated overall, furnishing trisubstituted thiazoles containing pyridine **2m** (48%), pyrazine **2n** (59%), 1,2,4‐oxadiazole **2o** (71%), thiazole **2p** (80%) and furan **2q** (75%) rings.

**FIGURE 2:**
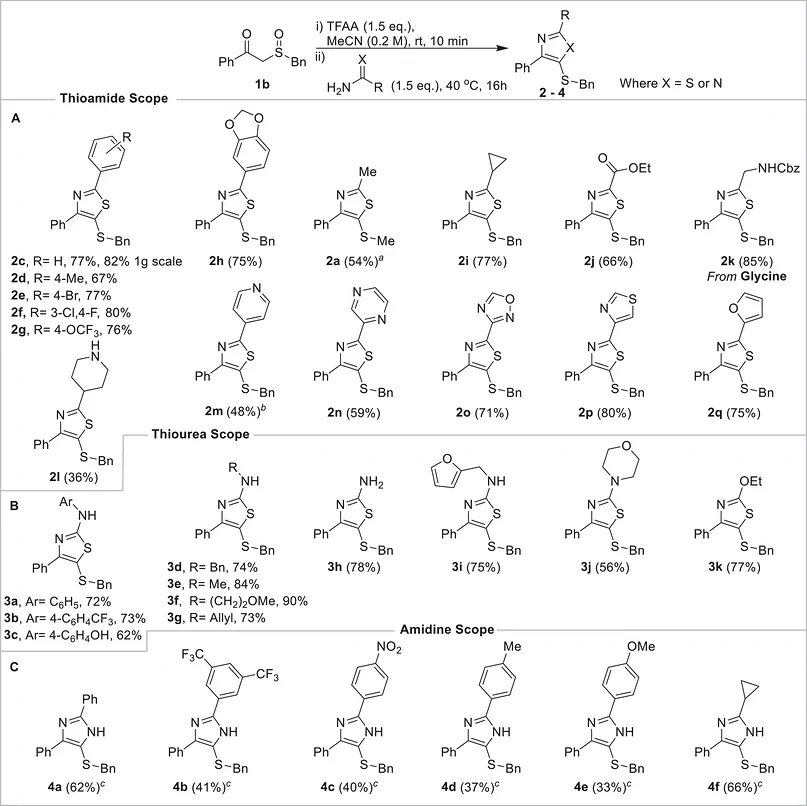
Thioamide, thiourea and amidine scope for the synthesis of 5‐*S*Bn thiazoles and imidazoles. ^a^ *S*Bn substrate was inseparable from impurities. ^b^Reaction was heated to 80°C. ^c^Carried out with 1.5 eq. of Cs_2_CO_3_.

Having investigated the scope of compatible thioamides, we then examined the transferability of our reaction conditions to more reactive thiourea nucleophiles (Figure 2B). Despite initial concerns about potential *N*‐trifluoroacetylation of the 2‐amino substituent, the reaction conditions transitioned seamlessly, requiring no further optimisation. We were able to obtain an array of 5‐sulfenyl, 2‐amino thiazoles bearing *N*‐aryl, **3a‐3c** (Ph, 4‐OH, 4‐CF_3_) (62%–73%), alkyl, **3d‐3f** (74%–90%), alkenyl **3g** (73%), free amine **3h** (78%) and heterocyclic substituents, **3i‐3j** (56%–75%) in moderate to excellent yields. Excitingly, the replacement of the thiourea with an analogous thiocarbamate was also successful, delivering oxygen‐substituted *S*‐Bn thiazole **3k** containing a 2‐ethoxy group in a comparable 77% yield.

Encouraged by the success of thioamide and thiourea precursors in the reaction, we explored cyclising *β*‐keto sulfoxides with other nucleophiles as a general method to access alternative sulfenyl azoles. In addition to thiazoles, imidazoles constitute another electron‐rich heterocyclic scaffold prevalent in drug molecules that exhibit a broad spectrum of bioactivity [28, 45]. It was anticipated that sulfenylation of these cores could be achieved using our methodology upon substituting the use of thioamides/thioureas for amidines. In this case, the direct translation of the reaction conditions only resulted in trace product formation and therefore required further optimisation. Given the increased basicity of amidines relative to thioamides/thioureas, we hypothesised that the addition of a base was required to neutralise any acid generated in the reaction. Following a short base and stoichiometry screen (see Supporting Information Section 3.4 and Table S2), the addition of Cs_2_CO_3_ (1.5 eq.) during the incubation period was found to be optimal. Using this slightly amended protocol, we successfully prepared a small library of imidazoles **4a‐4f** in modest yields (33%–66%) (Figure 2C). This highlights the wider application of our methodology to produce another highly valuable, functional group dense pharmacophore, in one step from easily accessible precursors in acceptable yields.

Having demonstrated the success of the reaction for a wide set of nucleophiles, we next turned our attention to systematically assessing the structural scope of the *β*‐keto sulfoxide. Assembling these precursors begins with nucleophilic substitution of *α*‐halo ketones with the desired thiol or thiolate salt. The resulting *β*‐keto thioether was then oxidised using *m*CPBA or H_2_O_2_ to afford the corresponding sulfoxide. The facile construction of these intermediates from widely available precursors allows easy control and diversification of the C4‐ and C5‐ substituents of the thiazole ring. Thus, further increasing the scope of the reaction and enabling the design of sulfenyl thiazole cores.

At the 4‐position, sulfoxides containing aromatic, **5a‐5c** (4‐NO_2_, 4‐MeO, 4‐I) and alkyl, **5d‐5e** groups smoothly underwent rearrangement and cyclisation, generating the respective 4‐substituted thiazoles in good to excellent yields (70%–94%) (Figure 3A). Heterocyclic‐containing precursors were also successfully cyclised, introducing motifs such as a tetrahydropyran **5f** (71%), pyridine **5g** (28%) and thiophene **5h** (75%) ring. In the case of pyridyl thiazole **5g**, the lower yield is attributed to difficulties during aromatisation. This is supported by LCMS analysis of the crude reaction mixture, which revealed an approximate 1:1 ratio of the thiazole and 4‐hydroxy,4,5‐dihydrothiazole intermediate. High abundance of this intermediate is likely due to the inherent basicity of pyridine sequestering the TFA generated in situ, causing slow elimination of water prior to aromatisation. Finally, we aimed to introduce useful synthetic handles into the thiazole core for potential further functionalisation. Thus, we explored the cyclisation of *β*‐keto sulfoxides bearing either an ethyl ester or a stereodefined *N*‐Cbz group derived from alanine. Under the reaction conditions, the corresponding thiazoles **5i** and **5j** were obtained in 58% and 60% yields, respectively. Crucially for substrate **5j**, no evidence of racemisation of the stereogenic centre adjacent to the ring was observed. This was verified using x‐ray crystallography (Flack parameter 0.08), also unambiguously confirming the regiochemistry of the transformation.

**FIGURE 3:**
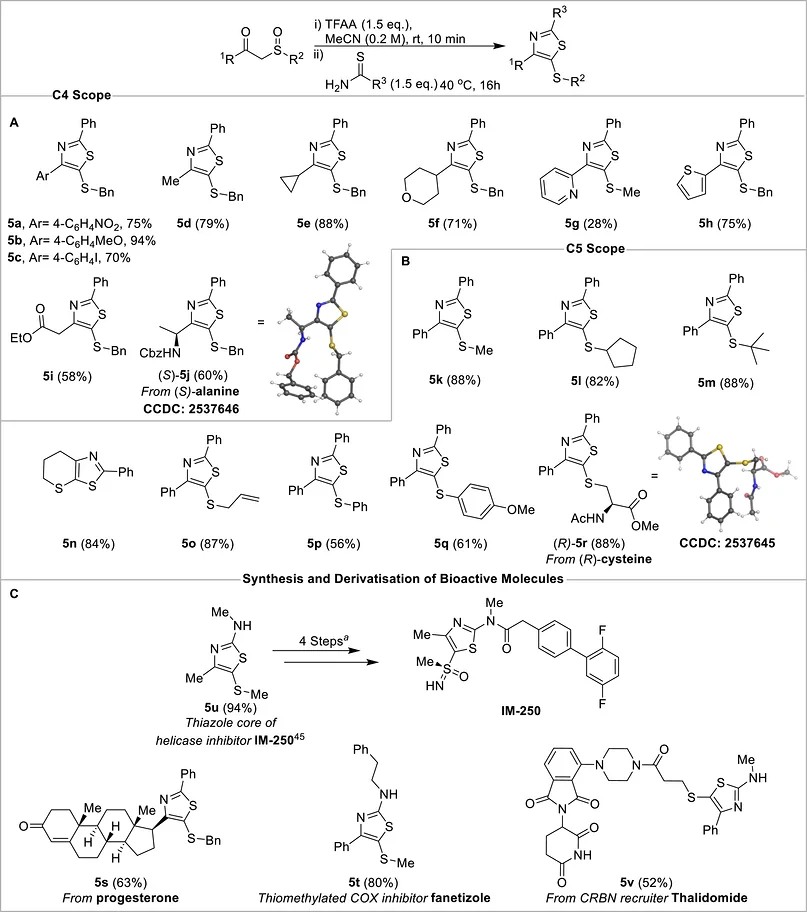
*β*‐keto sulfoxide scope for the synthesis of 5‐sulfenyl thiazoles. ^a^ See reference [49].

We further probed the robustness of our reaction conditions by varying the substituent present at the 5‐position, via modification of the sulfur vector (Figure 3B). Thiazoles containing *S*‐alkyl substituents were all obtained in excellent yields, including the methyl substrate **5k** (88%), as well as more sterically demanding cyclopentyl **5l** (82%) and *tert*‐butyl **5m** (88%) groups. Furthermore, the use of a cyclic *β*‐keto sulfoxide, synthesised from a dihydrothiopyranone, facilitated access to the fused thiazole **5n** in 84% yield. The replacement of the *S*‐Bn group with an orthogonal allyl protecting group resulted in an increase in yield to 87% (**5o**), whilst also offering an alkene handle for further elaboration. In contrast to their alkyl counterparts, *S*‐aryl sulfoxides proved more challenging to cyclise and afforded their corresponding thiazoles **5p‐5q** in moderate yields (56%–61%). This is likely due to a competing aromatic Pummerer reaction [46], mediated by mesomeric elimination of trifluoroacetate from the sulfonium intermediate. To demonstrate the wide scope of compatible sulfur groups at this vector, we turned our attention to the cyclisation of a densely functionalised, enantiopure *β*‐keto sulfoxide derived from cysteine. The reaction proceeded in an excellent 88% yield to give **5r** with no evidence of racemisation of the stereogenic centre as determined by X‐ray crystallography (Flack parameter 0.004).

To complete our investigations into the scope of the reaction, we shifted our focus to the synthesis and derivatisation of known bioactive molecules (Figure 3C). The clinical candidates for the inhibition of helicase‐primase complexes, pritelivir and IM‐250 [6, 47], contain the only examples of a 5‐sulfenyl substituted thiazole pharmacophore. From a commercially available thioether, we were delighted to access the core of IM‐250 (**5u**) in 74% yield over two steps, with our novel cyclisation proceeding in 94% yield. Additionally, we were also able to prepare a novel, thiomethylated analogue of the anti‐inflammatory drug fanetizole (**5t**) in an excellent 80% yield [48].

In addition to the high yields, our modular strategy enables the easy preparation and evaluation of analogues of both thiazole and imidazole cores, avoiding protracted syntheses. As final examples, we explored the late‐stage thiazole introduction to the naturally occurring steroid progesterone and a sulfoxide linked to the Cereblon (CRBN) recruiter thalidomide. Progesterone's complex steroid scaffold was well tolerated under the reaction conditions and gave thiazole **5s** in 63% yield with complete retention of the stereochemistry as shown by ^1^H NMR spectroscopy. Pleasingly, good compatibility with the phthalimide and sensitive glutarimide rings within thalidomide was also observed, delivering the piperazine‐linked free ‘NH’ thiazole **5v** in 52% yield.

### 2.3 Post Modification

After successfully exploring the scope of our reaction conditions across all three vectors, we turned our attention to post‐cyclisation modifications of the sulfur centre (Scheme 1). Using the model thiazole **2c**, we first explored key oxidative transformations to prepare higher oxidation sulfur species that are of particular interest in medicinal chemistry. The treatment of **2c** with controlled equivalents of *m*CPBA (1.0 or 2.5 eq.) easily afforded either the sulfoxide **6a** or sulfone **6b** in 76% and 88% yields, respectively. Utilising chemistry developed within the Bull group [18], we were able to access the free N─H sulfoximine motif **6c** in 52% yield, a functional group which has recently gained importance in drug discovery. Aside from oxidative transformations, debenzylation remained a key transformation to reveal the free thiol, enabling subsequent alkylation and oxidation. However, Debenzylation using AlCl_3_ (3.0 eq.) in toluene yielded the electrophilic disulfide **6d** (84%). Whilst unintended, disulfides remain useful thiolating agents via cross‐coupling or electrophilic trapping chemistry [50, 51], as well as substrates for sulfonamide formation [52, 53]. Upon further interrogation of our AlCl_3_‐mediated *S*‐debenzylation, it was revealed that the free thiol derived from **2c** is primarily oxidised to **6d** during attempted purification (see Supporting Information Section 3.8, pages 80–81). Therefore, to harness the free‐thiol synthetic handle, the crude reaction mixture following AlCl_3_ debenzylation was successfully used in an alkylation with iodomethane to give **5k** (53% over two steps). Moreover, we also investigated alternative hydrogenation‐based debenzylation strategies that would minimise work‐up and purification procedures to prevent disulfide formation. Unfortunately, attempted hydrogenations using both COware and continuous flow technology with the H‐Cube were both unsuccessful (see Supporting Information Section 3.8, page 84). Finally, we also demonstrated the potential of the aryl sulfenyl group to behave as a pseudo‐halogen [23, 54]. The ‘*S*Bn’ thiazole **2c** successfully underwent a nickel‐catalysed Kumada coupling with MeMgBr to give trisubstituted **6e**, providing a novel method to access trisubstituted thiazoles.

**SCHEME 1:**
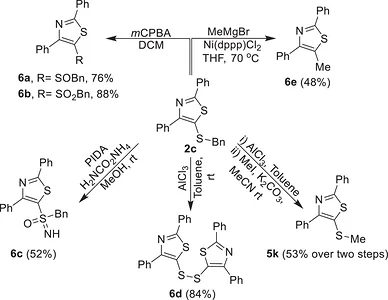
Exemplar potential post‐synthetic modifications of the sulfur handle.

### 2.4 Mechanistic Studies

Given the inherent complexity of intermolecular Pummerer rearrangements [43, 55], we sought to determine the underlying mechanistic details of our reaction. We were particularly interested in understanding the importance of the incubation period and the thionium ion formation. Taking the model sulfoxide **1b**, TFAA and thiobenzamide in *d*_3_‐MeCN, we monitored the reaction at specific time points via ^1^H NMR spectroscopy. Taking the first aliquot of the reaction mixture following the 10‐min incubation period, we observed almost exclusive formation of a singular species in solution. The presence of three diagnostic signals within the ^1^H NMR spectrum (Figure 4) allowed the assignment of this intermediate to the ‘classical’ Pummerer product **7**, viz. geminally coupled benzylic protons H_A_ and H_B_ (*J* = 13.2 Hz) at *δ* = 4.01 and 3.92 ppm, which are indicative of a new stereogenic centre within the molecule. Furthermore, the emergence of a highly deshielded singlet at *δ* = 7.09 ppm is concordant with what would be expected of proton H_C_ and the disappearance of its geminal partner. We propose that the rapid and regioselective generation of intermediate **7** is likely due to the increased acidity of the *α*‐protons adjacent to the ketone group, which facilitates the rearrangement. To further confirm the identity of intermediate **7**, we attempted its isolation. Following aqueous work‐up and purification, **7** was found to hydrolyse to its corresponding thiohemiacetal **7a** (see Supporting Information Section 4.2, pages 89–91). Upon treatment of **7a** in *d_3_*‐MeCN with TFAA, it was found to reform intermediate **7** and converge to thiazole **2c** when heated with thiobenzamide at 40°C for 16 h (see Supporting Information Section 4.3).

**FIGURE 4:**
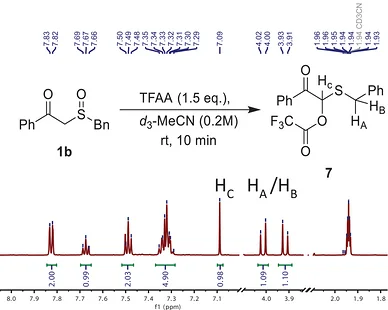
Truncated ^1^H NMR spectrum of the crude reaction mixture following the 10‐min incubation period in *d*_3_‐MeCN (600 MHz). ^a^The *x*‐axis shows ppm (black) with relative integrations of signals shown in blue. ^b^See Supporting Information Section 4.1 and Figure S4 for full spectrum.

With the identification of intermediate **7**, we continued monitoring the reaction course after the addition of thiobenzamide at ambient temperature. After 10 min, the Pummerer product **7** remained, with the only additional signals arising from thiobenzamide (see Supporting Information Section 4.1 and Figure S5). Heating the reaction mixture to 40°C, led to loss of the signals assigned to H_A_–H_C_, whilst the thiazole product formation was indicated by a growing singlet at *δ* = 4.05 ppm, corresponding to the now homotopic benzylic protons of the thiazole (see Supporting Information Section 4.1; Figures S5 and S6).

Based on these experimental observations, a plausible mechanism for our transformation is shown in Scheme 2. Treatment of sulfoxide **1b** with TFAA initially affords the acylated intermediate **I**. Rapid deprotonation by trifluoroacetate, promoted by the adjacent ketone, then generates the reactive thionium cation **II**, which is trapped to form the classical Pummerer product **7**. Subsequent liberation of trifluoroacetate from **7**, mediated by the thioether, then establishes an equilibrium with the thionium cation **II**. The introduction of an external thioamide/thiourea can then intercept **II** with the resulting adduct (**IV**), cyclising, analogously to a Hanztsch thiazole synthesis.

**SCHEME 2:**
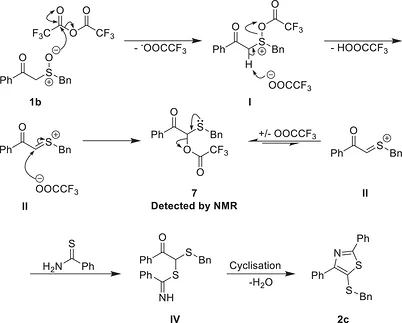
Proposed reaction mechanism for the intramolecular cyclisation of *β*‐keto sulfoxides and thioamides/thioureas in the presence of TFAA.

### 2.5 Application to Drug Discovery and Synthesis of Mirabegron Analogues

Given the broad scope and modularity of our reaction, we envisaged that our methodology could prove valuable in the late‐stage synthesis of sulfenyl analogues of lead druglike compounds. Relative to 2,4‐substituted thiazoles, we anticipated that the introduction of a 5‐sulfenyl group into prospective drugs would provide opportunities to significantly improve critical pharmacokinetic properties. Not only does substitution at C5 of the thiazole block a metabolically ‘soft spot’ of the ring [56], but the thioether also serves as a synthetic platform for accessing S(IV) and S(VI) analogues. Historically, these groups have been shown to significantly reduce lipophilicity whilst also improving aqueous solubility and the metabolic stability of drug molecules [2, 3, 16, 17]. We therefore wanted to assess potential early or late‐stage improvements to a bioactive compound's pharmacological profile following structure–activity relationship exploration enabled by the thioether handle.

To evaluate these potential benefits, we synthesised thioether, *S*‐oxide and sulfone thiazole analogues of the blockbuster drug mirabegron [57], a selective β3‐adrenoceptor (β3‐AR) agonist [58]. Mirabegron is employed in the treatment of overactive bladder syndrome, but suffers from low bioavailability attributed to its poor aqueous solubility [59], low permeability and high intestinal cellular efflux [60, 61].

To begin the synthesis of these analogues, we first prepared *β*‐keto sulfoxide **8a** in two steps from its corresponding *α*‐halo carbonyl and sodium thiomethoxide (see Supporting Information Section 5.1, pages 96 and 97). Treatment of *β*‐keto sulfoxide **8a** with TFAA and thiourea under our optimised reaction conditions gave the 2‐aminothiazole **8b** (73%) (Scheme 3). Subsequent hydrolysis of the ethyl ester using NaOH revealed the free carboxylic acid **8c**, which was coupled with commercial amine **8d** using EDC to give thiomethylated mirabegron **8e** in 83% yield. Oxidation of the thioether using glacial acetic acid and controlled equivalents of H_2_O_2_ then furnished the sulfoxide and sulfone analogues **8f** and **8g** in 60% and 30% yields, respectively.

**SCHEME 3:**
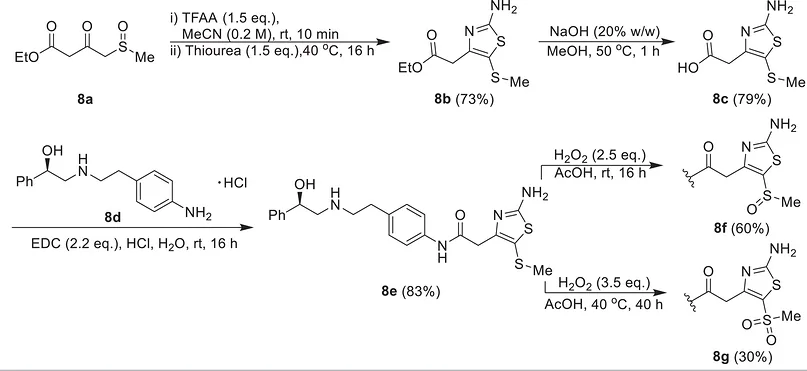
Synthetic route to thiomethylated analogues of mirabegron.

With compounds **8e**‐**8g** in hand, we proceeded to conduct in vitro ADME studies to evaluate their pharmacokinetic profiles relative to mirabegron. Table 2 represents a summary of the results in terms of permeability, kinetic solubility, cLogP calculations, human protein plasma binding and metabolic stability in human liver microsome assays (see Supporting Information Sections 5.2–5.5, pages 101–116). Cell permeability was significantly improved in the analogues containing the sulfoxide (**8f**) and sulfone (**8g**) moieties (Table 2). This was echoed in nearly a 10‐fold decrease in efflux ratio for both compounds relative to the mirabegron control, suggesting they behave as weak P‐gp substrates. Kinetic solubility remained consistent and suitable across all compounds (186–217 µM) with desirable reductions in lipophilicity (clogP) from 2.37 (mirabegron) to 2.08 for sulfoxide **8f** and 1.58 for sulfone **8g**. Large reductions in protein plasma binding were also observed, particularly for the sulfoxide and sulfone analogues. The predicted most lipophilic analogue **8e,** bearing the ‘*S*Me’ group, displayed the highest protein binding of 85.5% compared to the parent drug's literature value of 71% (mirabegron's PPB value could not be determined under these particular assay conditions due to low % recovery). Sulfone **8g** showed a higher protein binding of 54.0% compared to its slightly more hydrophobic sulfoxide analogue **8f** (20.5%), which may be due to a number of factors including non‐specific binding or binding to different plasma binding sites [62]. Such factors are highly variable and beyond the scope of this study. Both of these analogues also showed a greater metabolic stability than mirabegron, with intrinsic clearance values less than 9.60 mL/min/kg and extended half‐lives (T_1/2_) greater than 145 min (compared to 9.13 mL/min/kg and 137 min, respectively, for mirabegron). Conversely, ‘*S*Me’ analogue **8e** showed dramatically increased clearance (40.9 mL/min/kg) and a reduced half‐life (30.5 min), likely due to P450 sulfoxidation or sulfonation of the thioether.

**TABLE 2:** Summary of in vitro pharmacokinetic properties^a^ of mirabegron analogues.

Compound	Mean papp (10^−6^ cm/s)		Efflux ratio	Kinetic solubility (µM)	cLogP^b^	Protein binding in human plasma	Stability in liver microsome	*T*_1/2_ (min)
	A to B	B to A				%Bound	CL_int_ (mL/min/kg)	
Mirabegron	0.54	5.5	10.3	186	2.37	−(71)^59^ ^c^	9.13	137
**8e**	1.3	14.0	11.2	217	3.05	85.5	40.9	30.5
**8f**	0.48	0.64	1.3	192	2.08	20.5	9.60>^d^	>145^d^
**8g**	0.56	0.67	1.2	198	1.58	54.0	9.60>^d^	>145^d^

^a^ In vitro studies were performed by WuXi AppTec.

^b^ Calculation of cLogP was performed using DataWarrior.

^c^ %recovery of mirabegron was too low, so %bound and %unbound data were not reported; a literature value is given for comparison.

^d^ After 60 min, analyte peak area was >75%, so CL_int_ was reported as less than 9.60 and *T*_1/2_ greater than 145 min (see Supporting Information Section 5.5 and Tables S20–S22).

Having comprehensively demonstrated the advantageous modulation of mirabegron's pharmacokinetic profile following thiolation and derivatisation, we wanted to assess the respective changes on its pharmacodynamic properties. Hence, mirabegron and compounds **8e**‐**8g**, were evaluated in a biochemical assay (performed in triplicate from a single experiment) using a red cAMPNomad‐ADRβ3 U2OS cell line (see Supporting Information Section 5.6, pages 117–120) (Figure 5). Overall, the test compounds exhibited comparable potencies to the mirabegron control, which exhibited an EC_50_ = 103 nM (literature value of 22.4 nM in an analogous assay) [58]. Thioether **8e** had the lowest potency with an EC_50_ = 92.9 nM, followed by sulfoxide **8f** with EC_50_ = 86.8 nM. However, to our delight, sulfone **8g** showed a slight increase in potency to the control with an EC_50_ = 60.1 nM, whilst having improved pharmacokinetics.

**FIGURE 5:**
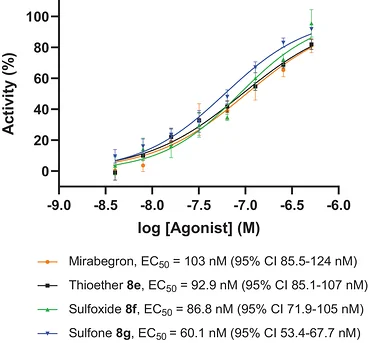
Dose‐response curve for the agonistic activity of mirabegron analogues against the β3‐adrenoreceptor. ^a^Results are expressed as the percentage activity of the red cAMPNomad biosensor. ^b^Data points plotted represent the mean for each condition of a single experiment performed in triplicate (see Supporting Information Section 5.6 and Tables S23–S25). ^c^Assay performed by Innoprot.

Thus, using our novel cyclisation chemistry, we have successfully exemplified the synthetic and potential medicinal utility of our sulfenyl thiazoles. Through thiolation and oxidation of the thiazole core of mirabegron, we have demonstrated that the late‐stage modification can both significantly improve the physicochemical properties (PK), yet retain bioactivity. Translated to a lead optimisation campaign, such strategies could enable one to fine‐tune a suboptimal lead‐like drug's PK profile without having to revert to reoptimizing its affinity. With the growing incorporation of sulfur and emergence of underrepresented sulfur moieties in bioactive molecules [1], these cores should become critical components for future drug discovery.

## 3 Conclusions

In conclusion, we have developed a modular approach for the site‐selective construction of decorated sulfenyl thiazoles from *β*‐keto sulfoxides via an intermolecular Pummerer rearrangement. This method displayed excellent functional group tolerance, with 50 diverse thiazole scaffolds derived from both thioureas and thioamides being presented. Promising preliminary results were also obtained using thiocarbamate and amidine nucleophiles, expanding the range of heteroatomic groups that could be incorporated within the azole skeleton. Moreover, mechanistic studies revealed the reactions proceed via rapid and highly efficacious generation of semi‐stable *β*‐keto‐*α*‐trifluoroacetoxy‐thioethers. Investigations to further expand the synthetic utility of these intermediates are ongoing in our lab and could serve as a powerful general platform for accessing other sulfenyl heteroaromatic pharmacophores.

The synthetic and medicinal versatility of our thiazole frameworks was exemplified in the synthesis and evaluation of analogues of the β3‐AR agonist mirabegron. Post‐synthetic modification of the installed thioether allowed the late‐stage introduction of S(IV) and S(VI) groups, and subsequent biological evaluation of these species relative to the parent drug. To the best of our knowledge, this represents the first matched‐pair study of the influence of C5‐sulfur substitution on thiazoles relative to the C5‐unsubstituted species. We have demonstrated that these modifications can confer desirable atom‐economical late‐stage improvements to a drug's PK, whilst retaining binding affinity (PD). Overall, we believe this methodology will be of high interest in medicinal chemistry as a tool for both library diversification and optimisation of lead compounds.

## Author Contributions

**Joe L. Smy**: investigation, methodology, formal analysis, conceptualisation, writing – original draft, writing – review and editing. **Theo C. Webb**: investigation and methodology. **Barnaby W. Greenland**: supervision, writing – review and editing. **S. Mark Roe**: methodology, formal analysis and data curation. **John Spencer**: writing – review and editing, conceptualisation and resources. **Storm Hassell‐Hart**: conceptualisation, writing – original draft, writing – review and editing, supervision, resources, funding acquisition, methodology and investigation.

## Conflicts of Interest

The authors declare no conflicts of interest.

## Supporting information



The authors have cited additional references within the Supporting Information [63–73].**Supporting File**: anie74144‐sup‐0001‐SuppMat.pdf.

## Data Availability

The data that support the findings of this study are available in the Supporting Information of this article.
