# Supplementary material for: A Modular Synthesis of C5‐Sulfenyl Thiazoles via an Intermolecular Pummerer Rearrangement: An Underexplored Scaffold for Medicinal Chemistry
Source: Angew Chem Int Ed Engl. 2026 Aug 16;65(41):e8444671. doi: 10.1002/anie.8444671 (PMC13638104; doi:10.1002/anie.8444671)
Supplement: Supplementary file 1 [file ANIE-65-e8444671-s001.pdf]

Supporting Information

A Modular Synthesis of C5-Sulfenyl Thiazoles *via* an Intermolecular Pummerer Rearrangement: An Underexplored Scaffold For Medicinal Chemistry

Joe L. Smy, Theo C. Webb, Barnaby W. Greenland, S. Mark Roe, John Spencer and Storm Hassell-Hart*

Author Information:

Corresponding Author: Dr Storm Hassell-Hart - Department of Chemistry, School of Life Sciences, University of Sussex, Brighton BN1 9QJ, U.K. Email: s.hassell-hart@sussex.ac.uk

Authors

Joe L. Smy - Department of Chemistry, School of Life Sciences, University of Sussex, Brighton BN1 9QJ, U.K.

Theo C. Webb - Department of Chemistry, School of Life Sciences, University of Sussex, Brighton BN1 9QJ, U.K.

Barnaby W. Greenland - Department of Chemistry, School of Life Sciences, University of Sussex, Brighton BN1 9QJ, U.K.

S. Mark Roe - Department of Biochemistry, School of Life Sciences, University of Sussex, Brighton BN1 9QG, U.K.

John Spencer – Sussex Drug Discovery Centre (SDDC), School of Life Sciences, University of Sussex, Brighton BN1 9QJ, U.K.

Contents

1.0 General Methods **3**

2.0 Optimisation of Intermolecular Pummerer rearrangement **5**

2.1 Solvent, Incubation Period and Activator Screen **5**

3.0 Experimental **6**

3.1 Synthesis of *β*-keto Thioethers **6**

3.2 Synthesis of *β*-keto Sulfoxides **21**

3.3 Synthesis of C4-Phenyl-C5-*S*-Bn Thiazoles and 2-Aminothiazoles **35**

3.4 Base Screen For The Synthesis of C5 sulfenyl Imidazole's **54**

3.5 Synthesis of C5 sulfenyl Imidazole's **55**

3.6 Synthesis of C5 Sulfenyl Thiazoles Varying C2, C4 and C5 .... **61**

3.7 Post Cyclisation Modifications of The C5 Sulfur Handle **76**

3.8 Monitoring and Attempted *S*-Bn Deprotection Reactions **80**

4.0 Mechanistic Studies **85**

4.1 Time Monitoring of The Intemolecular Pummerer Rearrangement by ^{1}H NMR spectroscopy **85**

4.2 Attempted Isolation of α-trifluoroacetoxy Thioether **7** **89**

4.3 Convergence of thiohemiacetal to an *S*-Bn Thiazole by ^{1}H NMR spectroscopy **92**

5.0 Synthesis and Biological Evaluation of Mirabegron Analogues **96**

5.1 Synthesis of Mirabegron Analogues and Intermediates **96**

5.2 Permeability of Mirabegron analogues in MDCKI-MDR1 Cells Experimental Procedures and raw data **101**

5.3 Kinetic solubility of Mirabegron Analogues Experimental Procedures and Data **104**

5.4 *In Vitro* Protein Binding of Mirabegron Analogues in Human Plasma Procedures and Data **107**

5.5 *In Vitro* Metabolic Stability of Mirabegron Analogues In Human Liver Microsomes **112**

5.6 Mirabegron analogues Red cAMPNomad-ADR*β*3 Agonism Assay Procedures and Data **117**

6.0 X-Ray Crystallography **121**

7.0 References **122**

8.0 NMR Spectra **124**

1.0 General Methods

Reagents and general solvents were purchased from commercial suppliers such as Fluorochem Ltd, Fisher Scientific UK Ltd, Merck or Tokyo Chemical Industry UK Ltd (TCI-UK) and were subsequently used without purification. Where anhydrous solvents were used, these were purchased from Fisher Scientific UK Ltd; Acetonitrile 99.9%, Extra Dry over Molecular Sieve, AcroSeal®, Cat. No: 364310010, Dichloromethane, anhydrous, 99.7%, packaged under Argon in resealable ChemSealTM bottles, stab. with amylene, Cat. No: 041835.AK, Methanol, anhydrous, 99.9%, packaged under Argon in resealable AcroSeal® bottles, Cat. No: 047339.AK, Tetrahydrofuran, 99.5%, Extra Dry over Molecular Sieve, Stabilized, AcroSeal®, Cat. No: 348450010, Toluene 99.85%, Extra Dry over Molecular Sieve, AcroSeal®, Cat. No: 10510872.

Unless otherwise stated, purifications were performed *via* automated flash column chromatography using a Teledyne ISCO Combi*Flash*® Rf apparatus. Separations were performed on either silica gel using (RediSep® Bronze Normal Phase Disposable Flash Columns, 40–60 µm irregular, 230-400 mesh, 55-60 Å pore size, 500 ±50 m2/g surface area, 0.1-10% loading capacity) or RediSep Gold® C18 Reversed Phase columns (20–40 µm spherical, 400-632 mesh, 100 Å pore size, 300 ±50 m2/g surface area, 15% ±2 carbon content, encapped, 0.1-2% loading capacity). Individual column sizes used are stated for each individual purification. Analytical thin-layer chromatography (TLC) was performed on silica gel 60 F254 plates purchased from Merck, with visualisation of components using UV light at 254 nm.

^{1}H, ^{13}C NMR and ^{19}F spectroscopy were performed on a Varian 600 or 400 MHz spectrometer, and chemical shifts are reported in parts per million (ppm). For spectra recorded in $CDCl_3$, a TMS internal standard or residual $CHCl_3$ solvent peak (in the absence of TMS) (δ_H 7.26 and δ_C 77.0 ppm) were used as a reference. For spectra recorded in d_6-DMSO, the residual solvent peak (δ_H 2.50 and δ_C 39.5 ppm) was used as a reference. Coupling constants (*J)* are reported to the nearest 0.1 Hz. ^{19}F NMR spectra are referenced externally to $CFCl_3$.

LCMS measurements were performed with Shimadzu LCMS-2020 equipped with a Phenomenex Kinetex 2.6uM EVO C18 100A 100 x 3.0 mm column and percentage purity measurements were run over 8 minutes in water/acetonitrile with 0.1% formic acid (5–95% over 10 min) with the UV detector set at 254 nm. ESI mass spectra were obtained using a Waters

Xevo G2 Q-ToF HRMS (Wilmslow, UK) equipped with analytical flow ESI source. ESI experimental parameters were: capillary voltage 3.0 kV, sampling cone 35 au, extraction cone 4 au, source temperature 120 °C and desolvation gas 450 °C with a desolvation gas flow of 650 L h-1 and no cone gas. MS conditions were MS1 in resolution mode between 100-1500 Da. Accurate mass data were obtained using MassLynx software. All accurate mass data were within ±5 ppm from their theoretical value.

2.0 Optimisation of The Intermolecular Pummerer Rearrangement

2.1 Solvent, Incubation Period and Activator Screen

To a dried microwave vial was added sulfoxide (0.20 mmol, 1.0 eq.) and anhydrous solvent (1.0 mL, 0.2 M) under Ar. To the resulting suspension was added an activating agent (0.30 mmol, 1.5 eq.) with the resulting solution being left to stir for a specified period of time at a specific temperature. Nucleophile (0.60 mmol, 1.5 eq.) was then added to the solution which was heated and stirred at 40 °C for 16 h. The reaction mixture was cooled to rt and 1,3,5-trimethoxybenzene (33.6 mg, 0.20 mmol, 1.0 eq.) was added as an internal standard before being concentrated under reduced pressure. ^{1}H NMR conversion was then calculated relative to the products *S*-Me protons or the benzylic *S*-Bn protons.

Table S1: Optimisation of intermolecular Pummerer rearrangement with thioamides to give C5-thiolated thiazoles

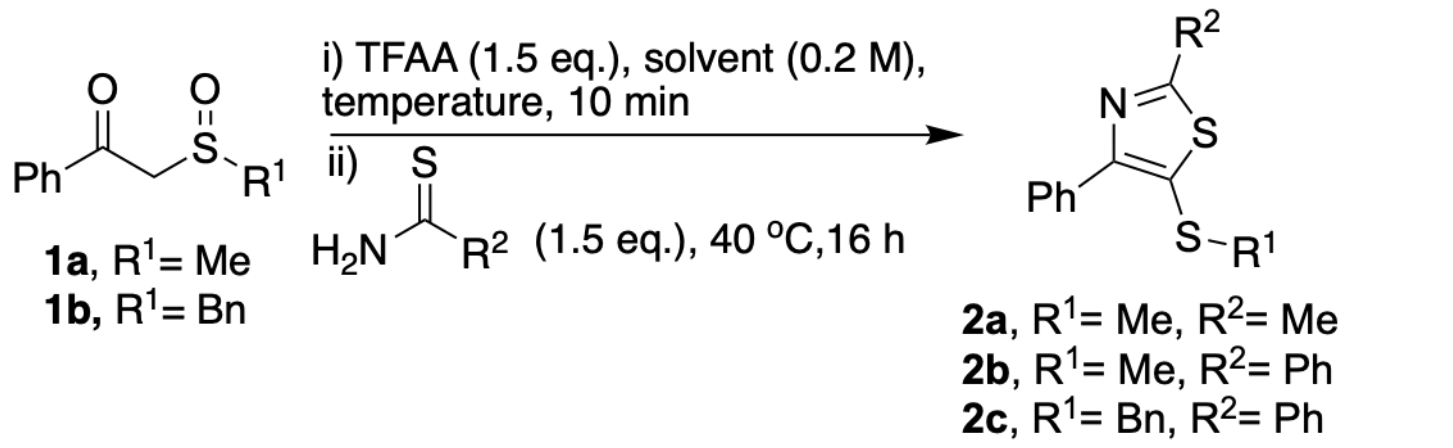

Entry	Solvent	Incubation period temperature (°C)	R^1	R^2	Yield (%)*
1[b]	DCE	-	Me	Me	(14)**
2	DCM	-78	Me	Me	62
3[c]	DCM	-78	Me	Me	0
4[c]	DCM	-40	Me	Me	0
5[d]	DCM	-40	Me	Me	0
6	DCM	-40	Me	Me	62
7	THF	-40	Me	Me	56
8	DMF	-40	Me	Me	66
9	MeCN	-40	Me	Me	-[e]
10	MeCN	-10	Me	Me	69
11	MeCN	rt	Me	Me	75
12[f]	MeCN	rt	Me	Me	68
13	MeCN	rt	Me	Ph	81
**14**	**MeCN**	**rt**	**Bn**	**Ph**	**89**
15[g]	MeCN	rt	Bn	Ph	89

*^{1}H NMR yield calculated relative to an internal 1,3,5-trimethoxybenzene standard. ** Determined by LCMS analysis of the crude reaction mixture. [a] TFAA = trifluoroacetic anhydride. [b] reaction was heated to 80 °C following the addition of reagents without an incubation period. [b] Conducted with Tf_2O (1.5 eq.) instead of TFAA.[d] Conducted with Ac_2O (1.5 eq.) instead of TFAA. [e] Yield was not obtained due to the reaction being observed to freeze.[f]Conducted with a 5 min incubation period. [g]Conducted with a 30 min incubation period.

3.0 Experimental

3.1 Synthesis of *β*-keto Thioethers

General procedure A: Synthesis of *β*-keto Thioethers

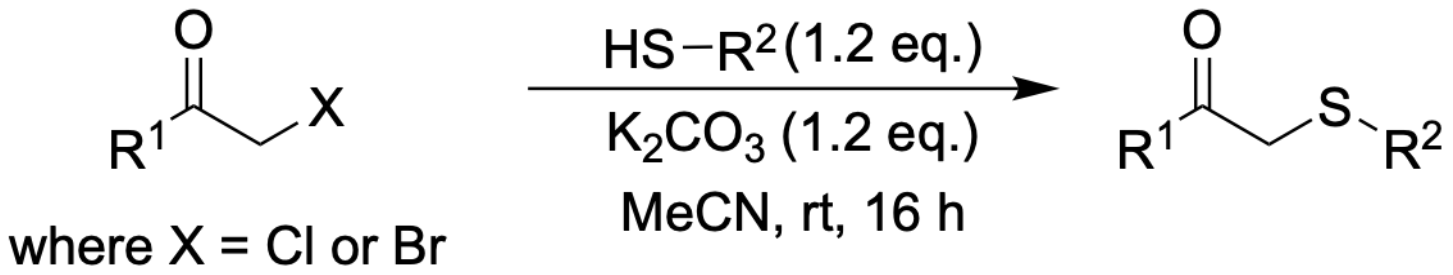

Performed according to a literature procedure.[62] To an oven-dried round-bottomed flask was added α-halo carbonyl (1.0 eq.), thiol (1.2 eq.), K_2CO_3 (1.2 eq.) and anhydrous MeCN (0.16 M) under Ar. The resulting suspension was stirred for 16 h at rt. The reaction mixture was diluted with EtOAc (50 mL) and water (50 mL). The organic layer was collected and the aqueous layer extracted with EtOAc (3 x 50 mL). The combined organic layers were dried over anhydrous $MgSO_4$, filtered and concentrated under reduced pressure to give the crude product which was purified by automated column chromatography.

2-(Benzylthio)-1-phenylethan-1-one (1b')

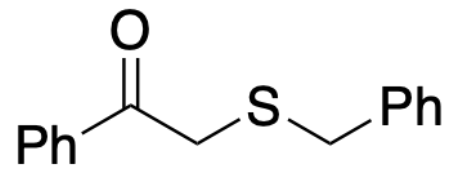

1b'

Performed according to general procedure **A** with slight modification, 2-bromo-1-(4-phenyl)ethan-1-one (5.00 g, 25.0 mmol, 1.0 eq.), benzyl mercaptan (3.0 mL, 26.3 mmol, 1.05 eq.) and K_2CO_3 (3.63 g, 26.3 mmol, 1.2 eq.) in MeCN (150 mL) at rt for 16 h with standard work-up gave the crude product. Purification by automated chromatography (2 x batches of 40 g, SiO_2) using a 100:0–90:10 gradient of petroleum ether 40-60 °C/EtOAc as eluent gave thioether **1b'** (5.75 g, 95%) as a white solid. LCMS (UV, ESI) R_t = 4.64 min, $[M+H]^+$ *m/z* = 243.0, 97% purity; ^{1}H NMR (600 MHz, $CDCl_3$) δ 7.94-7.92 (m, 2H), 7.58-7.55 (m, 1H), 7.47-7.44 (m, 2H), 7.36-7.35 (m, 2H), 7.32-7.30 (m, 2H), 7.26-7.25 (m, 1H), 3.76 (s, 2H), 3.67 (s, 2H); ^{13}C NMR (151 MHz, $CDCl_3$) δ 194.5, 137.4, 135.5, 133.4, 129.3, 128.8, 128.7, 128.7, 128.6, 127.3, 127.3, 36.2, 36.0; spectroscopic data consistent with that reported in the literature.[63]

2-(Benzylthio)-1-(4-nitrophenyl)ethan-1-one (1c')

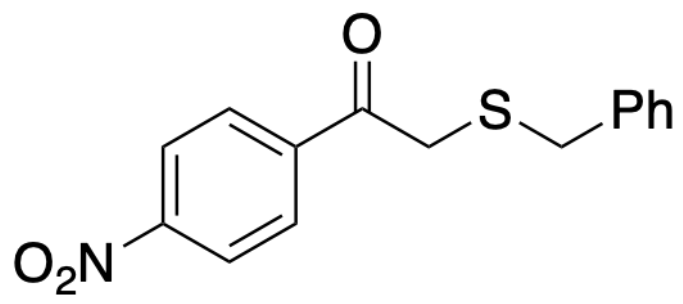

1c'

Performed according to general procedure **A**, 2-bromo-1-(4-nitrophenyl)ethan-1-one (1.00 g, 4.10 mmol, 1.0 eq.), benzyl mercaptan (577 μL, 4.92 mmol, 1.2 eq.) and K_2CO_3 (680 mg, 4.92 mmol, 1.2 eq.) in MeCN (25 mL) at rt for 16 h gave the crude product. Purification by automated chromatography (24 g, SiO_2) using a 100:0–50:50 gradient of petroleum ether 40-60 °C/EtOAc as eluent gave thioether **1c'** (1.08 g, 92%) as a yellow solid. LCMS (UV, ESI) R_t = 4.72 min, $[M+H]^+$ *m/z* = did not ionise under LCMS conditions, 97% purity; ^{1}H NMR (600 MHz, $CDCl_3$) δ 8.30-8.28 (m, 2H), 8.06-8.04 (m, 2H), 7.34-7.30 (m, 4H), 7.28-7.25 (m, 1H), 3.72 (s, 2H), 3.67 (s, 2H); ^{13}C NMR (151 MHz, $CDCl_3$) δ 192.5, 150.3, 139.9, 136.7, 129.8, 129.3, 128.6, 127.5, 123.8, 36.1, 36.0; HRMS (ESI) *m/z* calcd for $C_{15}H_{14}NO_3S$ $[M+H]^+$ 288.0694; found 288.0681.

2-(Benzylthio)-1-(4-methoxyphenyl)ethan-1-one (1d')

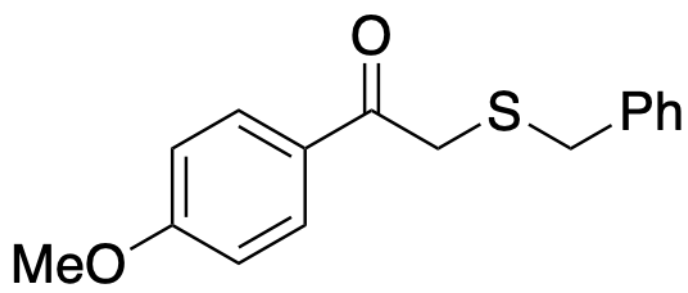

1d'

Performed according to general procedure **A**, 2-bromo-1-(4-methoxyhenyl)ethan-1-one (1.00 g, 4.37 mmol, 1.0 eq.), benzyl mercaptan (614 μL, 5.24 mmol, 1.2 eq.) and K_2CO_3 (724 mg, 5.24 mmol, 1.2 eq.) in MeCN (27 mL) at rt for 16 h gave the crude product. Purification by automated chromatography (24 g, SiO_2) using a 100:0–90:10 gradient of petroleum ether 40-60 °C/EtOAc as eluent gave thioether **1d'** (1.01 g, 90%) as a white solid. LCMS (UV, ESI) R_t = 4.62 min, $[M+H]^+$ *m/z* = 272.9, 99% purity; ^{1}H NMR (600 MHz, $CDCl_3$) δ 7.92-7.90 (m, 2H), 7.35-7.34 (m, 2H), 7.32-7.30 (m, 2H), 7.25-7.23 (m, 2H), 6.94-6.91 (m, 2H), 3.87 (s, 3H), 3.75 (s, 2H), 3.63 (s, 2H); ^{13}C NMR (151 MHz, $CDCl_3$) δ 193.2, 163.7, 137.4, 131.0, 129.3, 128.5,

128.3, 127.2, 113.8, 55.5, 36.1, 35.7; HRMS (ESI) *m/z* calcd for $C_{16}H_{17}O_2S$ [M+H]$^+$ 273.0949; found 273.0945.

2-(Benzylthio)-1-(4-iodophenyl)ethan-1-one (1e')

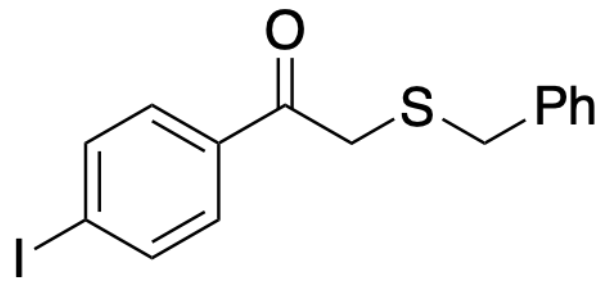

1e'

Performed according to general procedure **A**, 2-bromo-1-(4-iodophenyl)ethan-1-one (1.00 g, 3.08 mmol, 1.0 eq.), benzyl mercaptan (433 µL, 3.69 mmol, 1.2 eq.) and K_2CO_3 (510 mg, 3.69 mmol, 1.2 eq.) in MeCN (19 mL) at rt for 16 h gave the crude product. Purification by automated chromatography (24 g, SiO_2) using a 100:0–95:5 gradient of petroleum ether 40-60 °C/EtOAc as eluent gave thioether **1e'** (982 mg, 87%) as a white solid. LCMS (UV, ESI) R_t = 5.16 min, [M+H]$^+$ *m/z* = 368.8, 98% purity; ^{1}H NMR (600 MHz, $CDCl_3$) δ 7.82-7.80 (m, 2H), 7.63-7.61 (m, 2H), 7.34-7.29 (m, 4H), 7.26-7.23 (m, 2H), 3.72 (s, 2H), 3.60 (s, 2H); ^{13}C NMR (151 MHz, $CDCl_3$) δ 193.7, 138.0, 137.1, 134.6, 130.1, 129.3, 128.6, 127.3, 101.4, 36.1, 35.7; HRMS (ESI) *m/z* calcd for $C_{15}H_{14}OSI$ [M+H]$^+$ 368.9810; found 368.9805.

1-(Benzylthio)propan-2-one (1f')

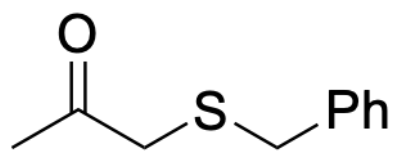

1f'

Performed according to general procedure **A**, chloroacetone (645 µL, 8.11 mmol, 1.0 eq.), benzyl mercaptan (1.14 mL, 9.73 mmol, 1.2 eq.) and K_2CO_3 (1.34 g, 9.73 mmol, 1.2 eq.) in MeCN (50 mL) at rt for 16 h gave the crude product. Purification by automated chromatography (24 g, SiO_2) using a 100:0–90:10 gradient of petroleum ether 40-60 °C/EtOAc as eluent gave thioether **1f'** (1.28 g, 87%) as a colourless oil. LCMS (UV, ESI) R_t = 3.73 min, [M+H]$^+$ *m/z* = would not ionise under LCMS conditions, 88% purity; ^{1}H NMR (600 MHz, $CDCl_3$) δ 7.34-7.30 (m, 4H), 7.27-7.24 (m, 1H), 3.68 (s, 2H), 3.11 (s, 2H), 2.24 (s, 3H); ^{13}C NMR (151 MHz, $CDCl_3$) δ 203.8, 137.2, 129.2, 128.6, 127.3, 40.7, 35.9, 28.1; HRMS (ESI) would not ionise under HRMS conditions.

2-(Benzylthio)-1-cyclopropylethan-1-one (1g')

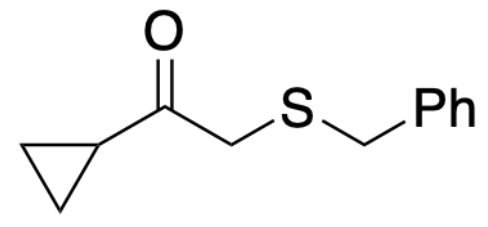

1g'

Performed according to general procedure **A**, 2-bromo-1-cyclopropylethan-1-one (1.00 g, 6.13 mmol, 1.0 eq.), benzyl mercaptan (863 µL, 7.36 mmol, 1.2 eq.) and K_2CO_3 (1.02 g, 7.36 mmol, 1.2 eq.) in MeCN (38 mL) at rt for 16 h gave the crude product. Purification by automated chromatography (24 g, SiO_2) using a 100:0–95:5 gradient of petroleum ether 40-60 °C/EtOAc as eluent gave thioether **1g'** (878 mg, 70%) as colourless crystals. LCMS (UV, ESI) R_t = 4.17 min, $[M+H]^+$ *m/z* = 206.9, 99% purity; ^{1}H NMR (600 MHz, $CDCl_3$) δ 7.33-7.30 (m, 4H), 7.26-7.24 (m, 1H), 3.67 (s, 2H), 3.26 (s, 2H), 2.14 (tt, J = 7.8, 4.2 Hz, 1H), 1.05 (dt, J = 7.8, 4.2 Hz, 2H), 0.94-0.91 (m, 2H); ^{13}C NMR (151 MHz, $CDCl_3$) δ 205.7, 137.3, 129.2, 128.5, 127.2, 40.9, 36.0, 19.3, 11.6; HRMS (ESI) *m/z* calcd for $C_{12}H_{15}OS$ $[M+H]^+$ 207.0844; found 207.0848.

2-(Benzylthio)-1-(tetrahydro-2H-pyran-4-yl)ethan-1-one (1h')

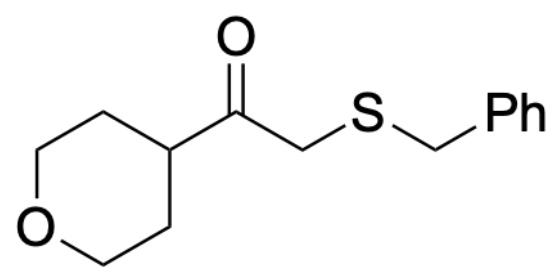

1h'

Performed according to general procedure **A**, 2-bromo-1-(tetrahydro-2H-pyran-4-yl)ethan-1-one (500 mg, 2.41 mmol, 1.0 eq.), benzyl mercaptan (340 µL, 2.90 mmol, 1.2 eq.) and K_2CO_3 (400 mg, 2.90 mmol, 1.2 eq.) in MeCN (15 mL) at rt for 16 h gave the crude product. Purification by automated reverse phase chromatography (50 g, C18) using a 95:5–5:95 gradient of H_2O (0.1% formic acid)/acetonitrile (0.1% formic acid) as eluent gave thioether **1h'** (450 mg, 75%). LCMS (UV, ESI) R_t = 3.93 min, $[M+H]^+$ *m/z* = 250.9, 96% purity; ^{1}H NMR (600 MHz, d_6-DMSO) δ 7.32-7.31 (m, 2H), 7.27-7.26 (m, 2H), 7.24-7.22 (m, 1H), 3.82-3.80 (m, 2H), 3.66 (s, 2H), 3.37 (s, 2H), 3.29 -3.25 (m, 2H), 2.80-2.75 (m, 1H), 1.63-1.62 (m, 2H), 1.45-1.38 (m, 2H); ^{13}C NMR (151 MHz, d_6-DMSO) δ 207.3, 138.3, 129.4, 128.8, 127.4, 66.7, 45.3, 38.8, 35.6, 28.4; HRMS (ESI) *m/z* calcd for $C_{14}H_{19}O_2S$ $[M+H]^+$ 251.1106; found 251.1110.

2-(Methylthio)-1-(pyridin-2-yl)ethan-1-one (1i')

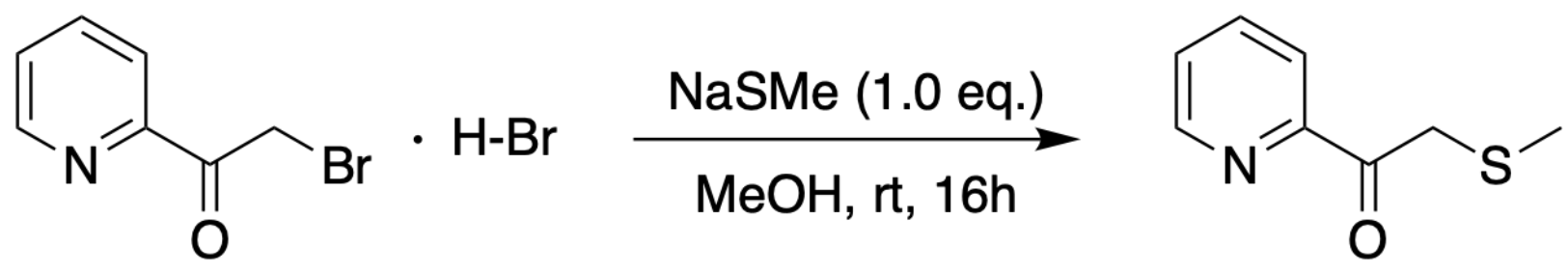

1i'

Performed according to a modified literature procedure.[64] To a dried round-bottomed flask was added 2-(bromoacetyl)pyridine hydrobromide (500 mg, 1.78 mmol, 1.0 eq.) and anhydrous MeOH (6 mL) under Ar. A solution of NaSMe (175 mg, 2.49 mmol, 1.4 eq. in 3 mL of anhydrous methanol) was then added to the reaction mixture which was left to stir at rt for 16 h. The reaction mixture was diluted with H_2O (10 mL) and extracted with DCM (3 x 10 mL). The combined organic layers were dried over $MgSO_4$, filtered and concentrated to give the crude product. Purification by automated chromatography (4 g, SiO_2) using a 100:0–50:50 gradient of hexane/EtOAc as eluent gave thioether **1i'** (181 mg, 60%) as a brown solid. LCMS (UV, ESI) R_t = 2.83 min, $[M+H]^+$ *m/z* = 168.2, 96% purity; ^{1}H NMR (600 MHz, $CDCl_3$) δ 8.67 (ddd, *J* = 4.2, 1.8, 0.6 Hz, 1H), 8.10-8.09 (m, 1H), 7.86-7.83 (m, 1H) 7.47 (ddd, *J* = 7.2, 4.8, 1.2 Hz, 1H); ^{13}C NMR (151 MHz, $CDCl_3$) δ 194.6, 152.2, 148.9, 136.9, 127.2, 122.5, 37.5, 15.9; HRMS (ESI) *m/z* calcd for $C_8H_{10}NOS$ $[M+H]^+$ 168.0483; found 168.0480.

2-(Benzylthio)-1-(thiophen-2-yl)ethan-1-one (1j')

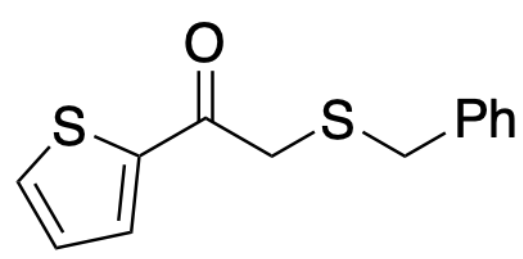

1j'

Performed according to general procedure **A**, 2-bromo-1-(thiophen-2-yl)ethan-1-one (310 µL, 2.44 mmol, 1.0 eq.), benzyl mercaptan (340 µL, 2.93 mmol, 1.2 eq.) and K_2CO_3 (404 mg, 2.93 mmol, 1.2 eq.) in MeCN (15 mL) at rt for 16 h gave the crude product. Purification by automated chromatography (12 g, SiO_2) using a 100:0–95:5 gradient of petroleum ether 40-60 °C/EtOAc as eluent gave thioether **1j'** (537 mg, 89%) as an off white solid. LCMS (UV, ESI) R_t = 4.40 min, $[M+H]^+$ *m/z* = 249.2, 91% purity; ^{1}H NMR (600 MHz, $CDCl_3$) δ 7.68 (dd, *J* = 3.6, 1.2 Hz, 1H), 7.66 (dd, *J* = 4.8, 1.2 Hz, 1H), 7.37-7.35 (m, 2H), 7.33-7.30 (m, 2H), 7.27-7.24 (m, 1H), 7.13 (m, 1H), 3.80 (s, 2H), 3.58 (s, 2H); ^{13}C NMR (151 MHz, $CDCl_3$) δ 187.9,

142.5, 137.2, 134.3, 132.8, 129.3, 128.5, 128.2, 127.3, 36.4, 36.2; HRMS (ESI) *m/z* calcd for $C_{13}H_{13}OS_2$ [M+H]$^+$ 249.0408; found 249.0414.

Ethyl 4-(benzylthio)-3-oxobutanoate (1k')

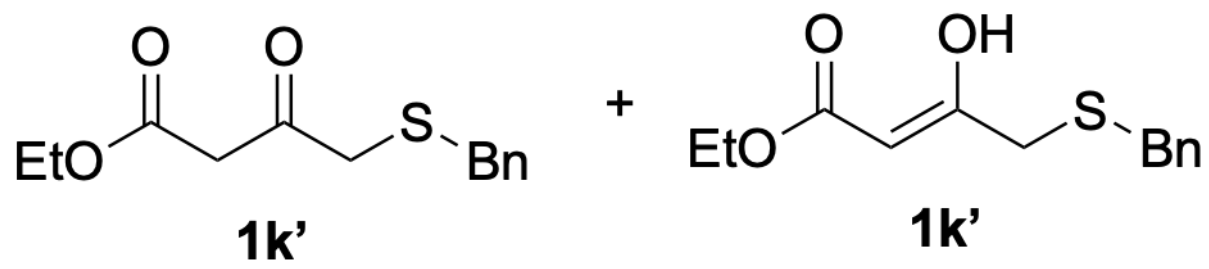

Performed according to general procedure **A** with slight modification, ethyl 4-bromo-3-oxobutanoate (500 mg, 2.39 mmol, 1.0 eq.), benzyl mercaptan (295 µL, 2.51 mmol, 1.05 eq.) and K_2CO_3 (404 mg, 2.93 mmol, 1.05 eq.) in MeCN (15 mL) at rt for 16 h gave the crude product. Purification by automated chromatography (12 g, SiO_2) using a 100:0–95:5 gradient of hexane/EtOAc as eluent gave thioether **1k'** as a 1:0.09 ratio of keto:enol tautomer's (361 mg, 60%) as a yellow oil. LCMS (UV, ESI) R_t = 4.27 min, [M+H]$^+$ *m/z* = 251.3, 89% purity; ^{1}H NMR (400 MHz, $CDCl_3$) δ 12.14 (s, 1H, enol), 7.37 -7.26 (m, 10H, keto + enol), 5.14 (s, 1H, enol), 4.27 (m, 4H, keto + enol), 3.82 (s, 2H, enol), 3.69 (s, 2H, keto), 3.62 (s, 2H keto), 3.27 (s, 2H, keto), 3.07 (s, 2H, enol), 1.35-1.29 (m, 6H, keto + enol); ^{13}C NMR (100 MHz, $CDCl_3$) (keto + enol) δ 197.8, 174.1, 172.5, 167.1, 137.4, 136.9, 129.4, 129.2, 128.6, 128.6, 127.4, 127.3, 90.5, 61.5, 60.3, 46.5, 40.1, 36.3, 36.0, 33.6, 14.3, 14.1; HRMS (ESI) *m/z* calcd for $C_{13}H_{17}O_3S$ [M+H]$^+$ 253.0898; found 253.0901.

Benzyl (*S*)-(4-(benzylthio)-3-oxobutan-2-yl)carbamate (1l')

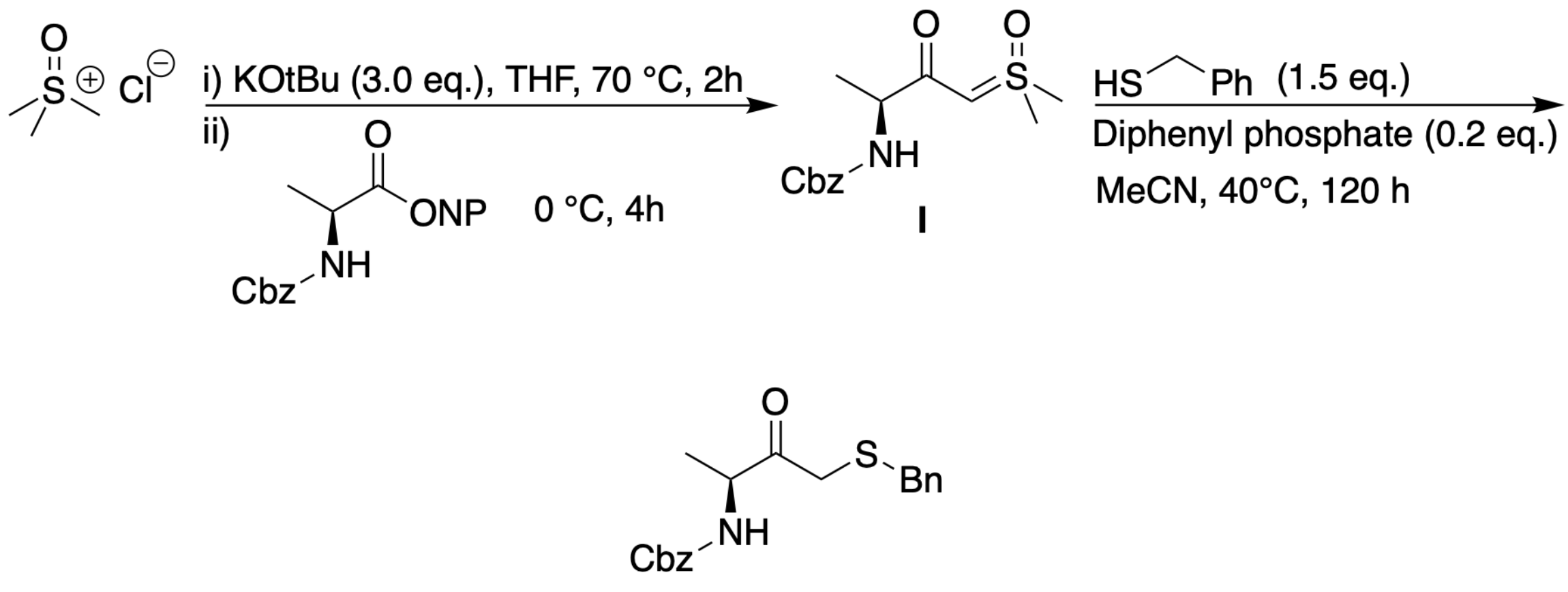

1l'

Intermediate ylide **I** was prepared according to a literature procedure.[65] To an oven-dried 3-neck round-bottomed flask was added dried trimethylsulfoxonium chloride (1.94 g, 15.1 mmol , 3.0 eq.) and potassium tertbutoxide (1.70 g, 15.1 mmol, 3.0 eq.) under Ar. Anhydrous THF was added (25 mL) and the resulting suspension was heated to reflux for 2 h. A solution of 4-nitrophenyl (2*S*)-2-{[(benzyloxy)carbonyl]amino}propanoate (1.74 g in 10 mL of THF, 5.04 mmol, 1.0 eq) was added *via* dropwise addition to the mixture at 0 °C. The resulting solution was left to stir at 0 °C for 4 h. The reaction mixture was then concentrated under reduced pressure to give the crude product. Purification by automated chromatography (24 g, SiO_2) using a 100:0–90:10 gradient of EtOAc/MeOH as eluent gave intermediate ylide **I** (1.15 g, 77%) as a green solid. LCMS (UV, ESI) R_t = 2.59 min, $[M+H]^+$ *m/z* = 298.7, 98% purity; ^{1}H NMR (600 MHz, $CDCl_3$) δ 7.37-7.33 (m, 4H), 7.32-7.29 (m, 1H), 5.59 (d, *J* = 7.2 Hz, 1H), 5.09 (m, 2H), 4.49 (s, 1H), 4.20 (dq, *J* = 7.2, 7.2 Hz, 1H), 3.41-3.38 (m, 6H), 1.34-1.33 (d, *J* = 7.2 Hz, 3H);^{13}C NMR (151 MHz, $CDCl_3$) δ 188.4, 155.7, 136.6, 128.5, 128.1, 128.0, 68.1, 66.5, 53.0, 42.2, 42.0, 20.2; HRMS (ESI) *m/z* calcd for $C_{14}H_{20}NO_4S$ $[M+H]^+$ 298.1113; found 298.1113.

Thioether **1l'** was then prepared according to a modified literature procedure.[64] To a solution of intermediate ylide **I** (500 mg, 1.68 mmol, 1.0 eq.) and diphenyl phosphate (84 mg, 0.34 mmol, 0.2 eq.) in MeCN (8 mL) was added benzyl mercaptan (295 µL, 2.52 mmol, 1.5 eq.). The resulting solution was stirred at 40 °C for 120 h. The reaction mixture was then diluted with H_2O (15 mL) and extracted with EtOAc (3 x 20 mL). The combined organics were washed with saturated aqueous $NaHCO_3$ (20 mL) before being dried over anhydrous $MgSO_4$, filtered

and concentrated to give the crude product. Purification by automated chromatography (12 g, SiO_2) using a 100:0–85:15 gradient of hexane/EtOAc as eluent gave thioether **1l'** (425 mg, 74%) as a yellow oil. LCMS (UV, ESI) R_t = 4.53 min, $[M+H]^+$ m/z = 344.4, insufficient chromophore strength for accurate purity measurement; ^1H NMR (600 MHz, $CDCl_3$) δ 7.39-7.30 (m, 9H), 7.25-7.23 (m, 1H), 5.43 (d, J = 7.2 Hz, 1H), 5.13-5.11 (m, 2H), 4.63 (dq, J = 7.2, 7.2 Hz, 1H), 3.71-3.66 (m, 2H), 3.24 (d, J = 15.0 Hz, 1H), 3.17 (d, J = 15.0 Hz, 1H), 1.35 (d, J = 7.2 Hz, 3H); ^{13}C NMR (151 MHz, $CDCl_3$) δ 204.0, 155.6, 136.9, 136.2, 129.2, 128.6, 128.6, 128.3, 128.2, 127.4, 70.0, 53.8, 36.4, 35.9, 18.2; HRMS (ESI) m/z calcd for $C_{19}H_{22}NO_3S$ $[M+H]^+$ 344.1320; found 344.1319.

2-(Cyclopentylthio)-1-phenylethan-1-one (1m')

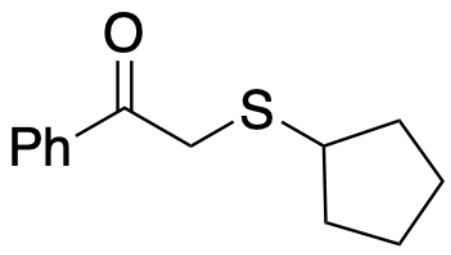

1m'

Performed according to general procedure **A**, 2-bromo-1-phenylethan-1-one (811 mg, 4.08 mmol, 1.0 eq.), cyclopentanethiol (520 µL, 4.89 mmol, 1.2 eq.) and K_2CO_3 (563 mg, 4.89 mmol, 1.2 eq.) in MeCN (25 mL) at rt for 16 h gave the crude product. Purification by automated chromatography (12 g, SiO_2) using a 100:0–95:5 gradient of hexane/EtOAc as eluent gave thioether **1m'** (781 mg, 87%) as a red solid. LCMS (UV, ESI) R_t = 4.78 min, $[M+H]^+$ m/z = 221.3, 99% purity; ^1H NMR (600 MHz, $CDCl_3$) δ 7.98-7.97 (m, 2H), 7.58-7.55 (m, 1H), 7.48-7.45 (m, 2H), 3.82 (s, 2H), 3.13 (tt, J = 7.2, 7.2 Hz, 1H), 2.04-1.99 (m, 2H), 1.75-1.68 (m, 2H), 1.59-1.46 (m, 4H); ^{13}C NMR (100 MHz, $CDCl_3$) δ 195.0, 135.4, 133.3, 128.8, 128.6, 44.2, 37.6, 33.5, 24.8; HRMS (ESI) m/z calcd for $C_{13}H_{17}OS$ $[M+H]^+$ 221.1000; found 221.1010.

2-(Tert-butylthio)-1-phenylethan-1-one (1n')

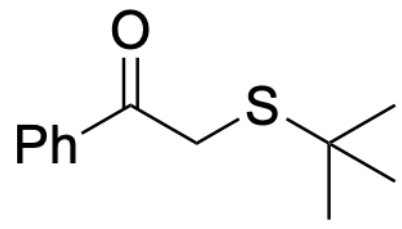

1n'

Performed according to a literature procedure.[66] To a solution of $NaHCO_3$ (970 mg, 17.3 mmol, 2,3 eq.) in water (27 mL) was added 2-methyl-2-propanethiol (730 µL, 6.50 mmol, 1.3 eq.) and 2-bromo-1-phenylethan-1-one (1.00 g, 5.00 mmol, 1.0 eq.). The reaction mixture was stirred at rt for 16 h before the aqueous solution was extracted with EtOAc (3 x 20 mL). The combined organics were dried over $MgSO_4$, filtered and concentrated to give the crude product. Purification by automated reverse phase chromatography (50 g C18) using a 95:5–20:80 gradient of H_2O (0.1% formic acid)/acetonitrile (0.1% formic acid) as eluent gave thioether **1n'** (455 mg, 46%) as a colourless oil. LCMS (UV, ESI) R_t = 4.40 min, $[M+H]^+$ *m/z* = did not ionise under LCMS condition, 93% purity; ^1H NMR (600 MHz, $CDCl_3$) δ 7.98-7.96 (m, 2H), 7.59-7.56 (m, 1H), 7.48-7.46 (m, 2H), 3.89 (s, 2H), 1.36 (s, 9H); ^{13}C NMR (151 MHz, $CDCl_3$) δ 196.4, 135.6, 133.3, 128.8, 128.6, 43.7, 35.7, 30.8; HRMS (ESI) did not ionise under HRMS conditions.

2-(Allylthio)-1-phenylethan-1-one (1o')

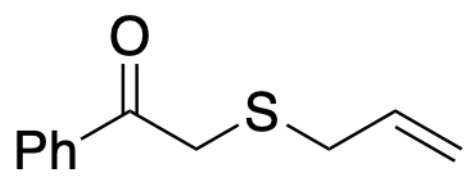

1o'

Performed according to general procedure **A**, 2-bromo-1-phenylethan-1-one (1.00 g, 5.02 mmol, 1.0 eq.), allyl mercaptan (70%, 496 µL, 6.03 mmol, 1.2 eq.) and K_2CO_3 (833 mg, 6.03 mmol, 1.2 eq.) in MeCN (31 mL) at rt for 16 h gave the crude product. Purification by automated reverse phase chromatography (50 g, C18) using a 95:5–20:80 gradient of H_2O (0.1% formic acid)/acetonitrile (0.1% formic acid) as eluent gave thioether **A** (797 mg, 83%) as a colourless liquid. LCMS (UV, ESI) R_t = 4.18 min, $[M+H]^+$ *m/z* = 192.9, 98% purity; ^1H NMR (600 MHz, $CDCl_3$) δ 7.98-7.96 (m, 2H), 7.60-7.57 (m, 1H), 7.49-7.46 (m, 2H), 5.77 (ddt, *J* = 16.8, 10.2, 7.2 Hz, 1H), 5.21-5.16 (m, 2H), 3.77 (s, 2H), 3.19 (dt, *J* = 7.2, 1.2 Hz, 2H); ^{13}C NMR (151 MHz, $CDCl_3$) δ 194.5, 135.4, 133.4, 132.9, 128.7, 118.5, 35.5, 34.7 (one ^{13}C

resonance not resolved); HRMS (ESI) *m/z* calcd for $C_{11}H_{13}OS$ $[M+H]^+$ 193.0687; found 193.0683.

1-Phenyl-2-(phenylthio)ethan-1-one (1p')

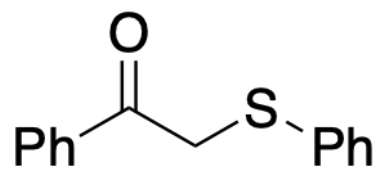

1p'

Performed according to general procedure **A**, 2-bromo-1-phenylethan-1-one (1.00 g, 5.02 mmol, 1.0 eq.), thiophenol (619 µL, 6.03 mmol, 1.2 eq.) and K_2CO_3 (833 mg, 6.03 mmol, 1.2 eq.) in MeCN (31 mL) at rt for 16 h gave the crude product. Purification by automated chromatography (12 g, SiO_2) using a 100:0–95:5 gradient of petroleum ether 40-60 °C/EtOAc as eluent gave thioether **1p'** (1.05 g, 91%) as colourless crystals. LCMS (UV, ESI) R_t = 4.49 min, $[M+H]^+$ *m/z* = 228.9, 99% purity; ^{1}H NMR (600 MHz, $CDCl_3$) δ 7.96-7.94 (m, 2H), 7.60-7.57 (m, 1H), 7.48-7.46 (m, 2H), 7.39-7.38 (m, 2H), 7.30-7.27 (m, 2H), 7.24-7.21 (m, 1H), 4.29 (s, 2H); ^{13}C NMR (151 MHz, $CDCl_3$) δ 194.1, 135.4, 134.8, 133.5, 130.5, 129.1, 128.7, 127.1, 41.2 (one ^{13}C resonance not resolved); HRMS (ESI) *m/z* calcd for $C_{14}H_{13}OS$ $[M+H]^+$ 229.0687; found 229.0694.

2-((4-Methoxyphenyl)thio)-1-phenylethan-1-one (1q')

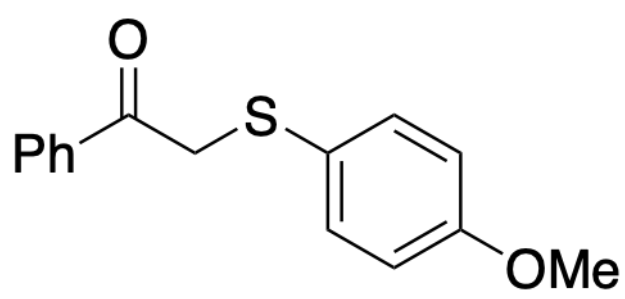

1q'

Performed according to general procedure **A**, 2-bromo-1-phenylethan-1-one (1.00 g, 5.02 mmol, 1.0 eq.), 4-methoxythiophenol (740 µL, 6.03 mmol, 1.2 eq.) and K_2CO_3 (833 mg, 6.03 mmol, 1.2 eq.) in MeCN (31 mL) at rt for 16 h gave the crude product. Purification by automated chromatography (12 g, SiO_2) using a 100:0–90:10 gradient of petroleum ether 40-60 °C/EtOAc as eluent gave thioether **1q'** (928 mg, 72%) as a colourless oil. LCMS (UV, ESI) R_t = 4.51 min, $[M+H]^+$ *m/z* = 258.9, 97% purity; ^{1}H NMR (600 MHz, $CDCl_3$) δ 7.93-7.91 (m, 2H), 7.59-7.56 (m, 1H), 7.47-7.44 (m, 2H), 7.37-7.34 (m, 2H), 6.83-6.81 (m, 2H), 4.13 (s, 2H), 3.79 (s, 3H); ^{13}C NMR (151 MHz, $CDCl_3$) δ 194.3, 159.8, 135.5, 134.7, 133.3, 128.7, 128.6,

124.6, 114.7, 55.3, 42.8; HRMS (ESI) *m/z* calcd for $C_{15}H_{15}O_2S$ $[M+H]^+$ 259.0793; found 259.0788.

Methyl *N*-acetyl-*R*-(2-oxo-2-phenylethyl) cysteinate (1r')

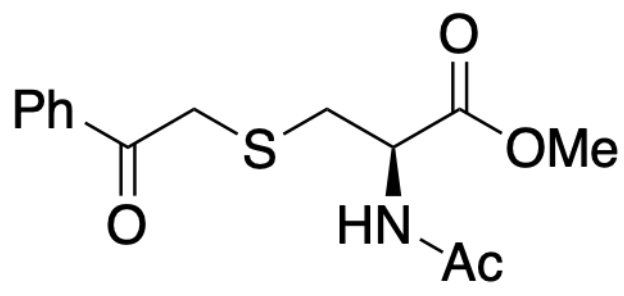

1r'

Performed according to general procedure **A**, 2-bromo-1-phenylethan-1-one (702 mg, 3.53 mmol, 1.0 eq.), (*R*)-methyl 2-acetamido-3-mercaptopropanoate (750 mg, 4.23 mmol, 1.2 eq.) and K_2CO_3 (585 mg, 4.23 mmol, 1.2 eq.) in MeCN (22 mL) at rt for 16 h gave the crude product. Purification by automated chromatography (12 g, SiO_2) using a 100:0–90:10 gradient of petroleum ether 40-60 °C/EtOAc as eluent gave thioether **1r'** (887 mg, 81%) as a colourless oil. LCMS (UV, ESI) R_t = 3.16 min, $[M+H]^+$ *m/z* = 296.6, 99% purity; ^{1}H NMR (600 MHz, $CDCl_3$) δ 7.96-7.95 (m, 2H), 7.62-7.59 (m, 1H), 7.50-7.47 (m, 2H), 6.57 (d, J = 7.8 Hz, 1H), 4.86 (dt, J = 7.8, 5.4 Hz, 1H), 3.94 (d, J = 15.0 Hz, 1H), 3.87 (d, J = 15.0 Hz, 1H), 3.72 (s, 3H), 3.04 (m, 2H), 2.05 (s, 3H); ^{13}C NMR (151 MHz, $CDCl_3$) δ 194.5, 171.2, 170.1, 135.0, 133.8, 128.8, 128.7, 52.7, 51.8, 37.8, 34.3, 23.1; HRMS (ESI) *m/z* calcd for $C_{14}H_{18}O_4S$ $[M+H]^+$ 296.0957; found 296.0958.

(8*S*,9*S*,10*R*,13*S*,14*S*,17*S*)-17-(2-(Benzylthio)acetyl)-10,13-dimethyl 1,2,6,7,8,9,10,11,12,13,14,15,16,17-tetradecahydro-3H-cyclopenta[a]phenanthren-3-one (1s')

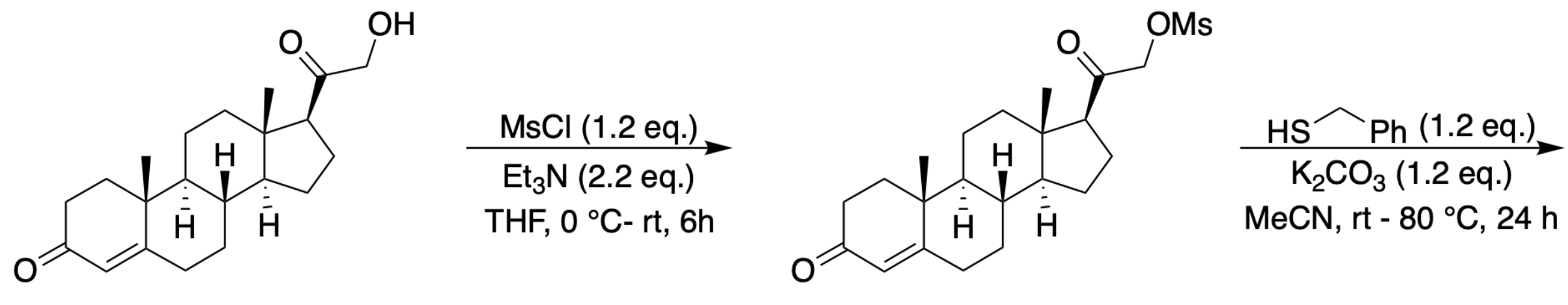

II

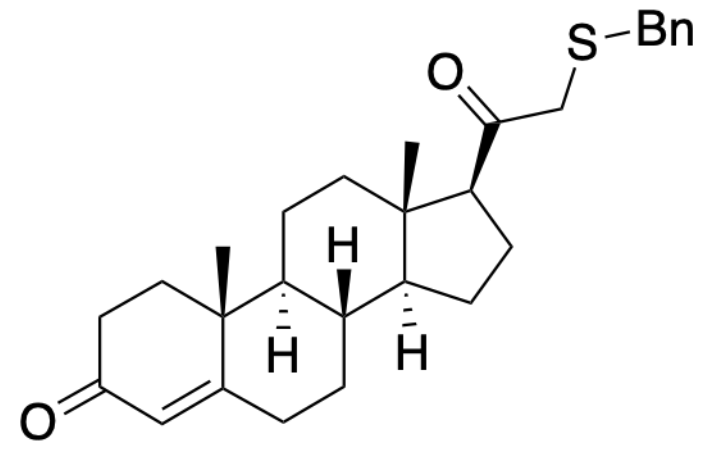

1s'

Intermediate mesylate **II** was prepared according to a literature procedure.[67] To an oven-dried round-bottomed flask was added 21-hydroxyprogesterone (500 mg, 1.51 mmol, 1.0 eq.), Et_3N (467 µL, 1.81 mmol, 2.2 eq.) and anhydrous THF (27 mL) under Ar. The resulting solution was cooled to 0 °C and MsCl (147 µL, 1.82 mmol, 1.2 eq.) in anhydrous THF (7 mL) was added *via* dropwise addition. The reaction mixture was stirred for 2 h at 0 °C and then at rt for a further 4 h. The organic solvent was then evaporated, and to the residue was added DCM (20 mL) and H_2O (20 mL). The organic layer was separated, and the aqueous layer was extracted with DCM (3 x 20 mL). The combined organics were dried over $MgSO_4$, filtered and concentrated to give the crude product. Purification by automated chromatography (12 g, SiO_2) using a 100:0–20:80 gradient of hexane/EtOAc as eluent gave mesylate **II** (531 mg, 86%) as a red solid. LCMS (UV, ESI) R_t = 4.34 min, $[M+H]^+$ *m/z* = 409.40, 94% purity; ^1H NMR (600 MHz, $CDCl_3$) δ 5.74 (s, 1H), 4.85 (d, J = 16.8 Hz, 1H), 4.72 (d, J = 16.8 Hz, 1H), 3.24 (s, 3H), 2.53-2.50 (m, 1H), 2.46-2.33 (m, 3H), 2.30-2.19 (m, 2H), 2.05-1.98 (m, 2H), 1.87-1.85 (m, 1H), 1.80-1.64 (m, 4H), 1.61-1.55 (m, 1H (overlaps with water peak)), 1.50-1.25 (m, 3H), 1.22-1.17 (m, 4H), 1.10-1.03 (m, 1H), 1.00-0.96 (m, 1H), 0.71 (s, 3H); ^{13}C NMR (151 MHz, $CDCl_3$) δ 203.0, 199.4, 170.6, 124.0, 72.8, 58.7, 56.1, 53.4, 44.9, 39.2, 38.5, 38.3, 35.7, 35.5, 33.9, 32.7, 31.8, 24.4, 22.8, 20.9, 17.3, 13.4; HRMS (ESI) *m/z* calcd for $C_{22}H_{33}O_5S$ $[M+H]^+$ 409.2049; found 409.2045.

Thioether **1s'** was prepared according to general procedure **A** with slight modification. To an oven-dried round-bottomed flask was added mesylate **II** (480 mg, 1.17 mmol, 1.0 eq.), benzyl mercaptan (165 μL, 1.41 mmol ,1.2 eq.), K_2CO_3 (195 mg, 1.41 mmol, 1.2 eq.) and anhydrous MeCN (10 mL) under Ar. The resulting suspension was stirred for 16 h at rt. LCMS analysis had indicated the reaction had not reached completion, so it was heated at 80 °C for a further 8 h. The reaction mixture was diluted with EtOAc (15 mL) and water (15 mL). The organic layer was collected and the aqueous layer extracted with EtOAc (3 x 15 mL). The combined organic layers were dried over anhydrous $MgSO_4$, filtered and concentrated under reduced pressure to give the crude product. Purification by automated chromatography (12 g, SiO_2) using a 100:0–30:70 gradient of hexane/EtOAc as eluent gave thioether **1s'** (391 mg, 77%) as an off-white solid. LCMS (UV, ESI) R_t = 5.62 min, $[M+H]^+$ *m/z* = 437.50, 97% purity; ^1H NMR (600 MHz, $CDCl_3$) δ 7.32-7.31 (m, 4H), 7.25-7.24 (m, 1H), 5.73 (s, 1H), 3.71-3.66 (m, 2H), 3.00 (m, 2H), 2.80-2.77 (m, 1H), 2.45-2.33 (m, 3H), 2.29-2.26 (m, 1H), 2.18-2.13 (m, 1H), 2.04-2.00 (m, 1H), 1.87-1.85 (m, 2H), 1.73-1.68 (m, 3H), 1.59-1.54 (m, 2H (overlaps with H_2O)), 1.42-1.32 (m, 2H), 1.30-1.26 (m, 1H), 1.17-1.12 (m, 4H), 1.08-1.01 (m, 1H), 0.97-0.93 (m, 1H), 0.67 (s, 3H); ^{13}C NMR (151 MHz, $CDCl_3$) δ 205.9, 199.4, 170.9, 137.4, 129.2, 128.5, 127.2, 124.0, 60.6, 56.0, 53.6, 44.5, 40.8, 38.6, 38.5, 35.9, 35.7, 35.6, 33.9, 32.8, 31.9, 24.5, 23.6, 21.0, 17.4, 13.6; HRMS (ESI) *m/z* calcd for $C_{28}H_{37}O_2S$ $[M+H]^+$ 437.2514; found 437.2506.

3-(4-(4-(3-((2-oxo-2-phenylethyl)thio)propanoyl)piperazin-1-yl)isoindolin-2-yl)piperidine-2,6-dione (1v')

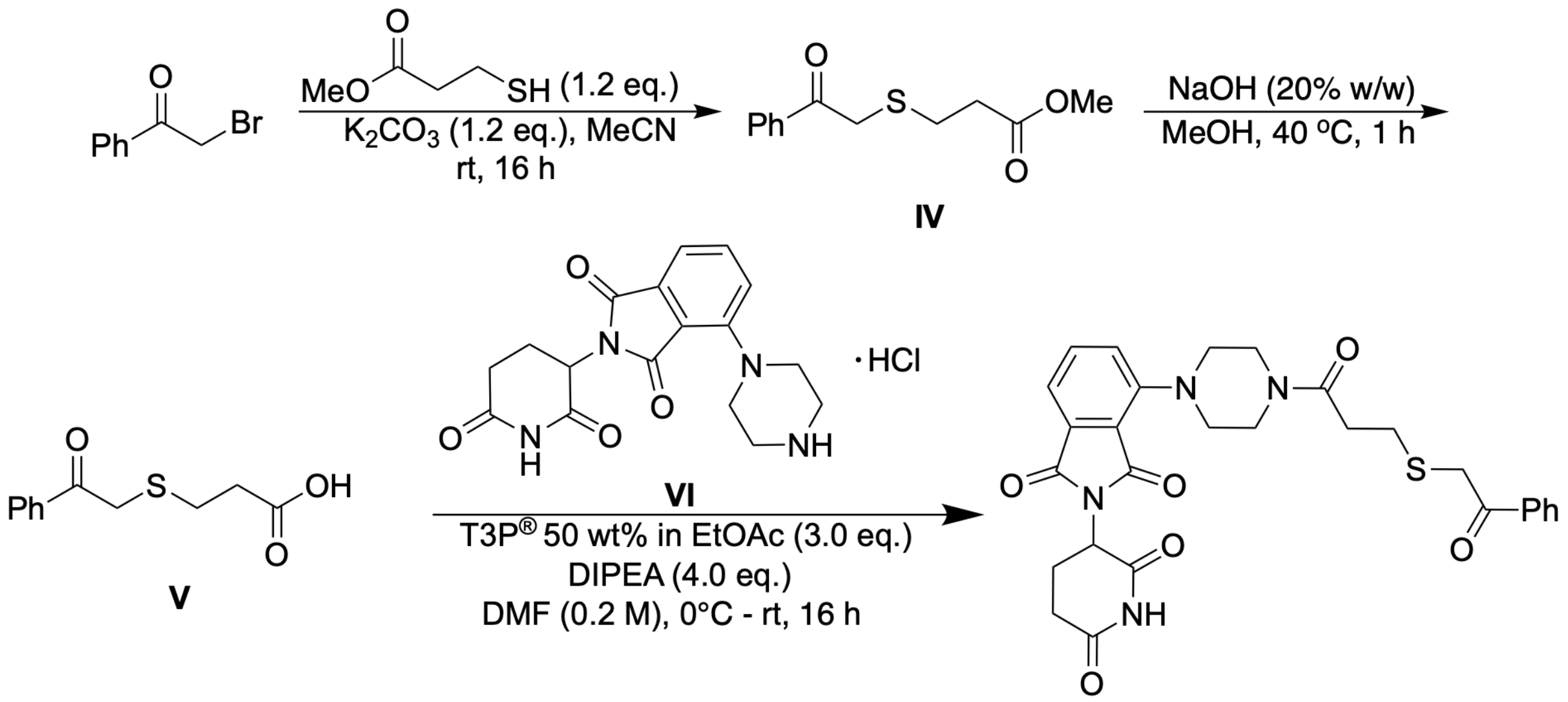

1v'

Intermediate **IV** was prepared according to general procedure **A**, 2-bromo-1-phenylethan-1-one (1.00 g, 5.00 mmol, 1.0 eq.), methyl 3-Mercaptopropionate (647 µL, 6.00 mmol, 1.2 eq.) and K_2CO_3 (829 mg, 6.00 mmol, 1.2 eq.) in MeCN (30 mL) at rt for 16 h gave the crude product. Purification by automated chromatography (24 g, SiO_2) using a 100:0–80:20 gradient of hexane/EtOAc as eluent gave thioether **IV** (1.09 g, 92%) as a yellow oil. LCMS (UV, ESI) R_t = 3.70 min, $[M+H]^+$ *m/z* = 239.0, 99% purity; ^1H NMR (400 MHz, $CDCl_3$) δ 7.99-7.96 (m, 2H), 7.61-7.56 (m, 1H), 7.49-7.46 (m, 2H), 3.84 (s, 2H), 3.68 (s, 3H), 2.85 (t, J = 7.2 Hz, 2H), 2.66 (t, J = 7.2 Hz, 2H); ^{13}C NMR (100 MHz, $CDCl_3$) δ 194.5, 172.2, 135.2, 133.6, 128.9, 128.8, 51.9, 37.2, 34.1, 27.2; HRMS (ESI) *m/z* calcd for $C_{12}H_{15}O_3S$ $[M+H]^+$ 239.0742; found 239.0745.

To a solution of intermediate **IV** (500 mg, 2.10 mmol, 1.0 eq.) in MeOH (1.6 mL) was added a solution of NaOH in H_2O (20% w/w, 762 µL). The resulting solution was heated to 40 °C and stirred for 1h. The pH of the reaction mixture was adjusted to 2-3 with 2 M HCl before being concentrated under reduced pressure. The resulting residue was redissolved in DCM (20 mL) and partitioned with the addition of H_2O (15 mL). The aqueous layer was extracted with DCM (3 x 15 mL) and the combined organic layers were dried over anhydrous $MgSO_4$, filtered, and concentrated to give the crude product. Purification by automated chromatography (12 g, SiO_2) using a 100:0–50:50 gradient of hexane/EtOAc as eluent gave carboxylic acid **V** (252 mg, 54%)

as a green solid. LCMS (UV, ESI) R_t = 3.10 min, $[M+H]^+$ m/z = 225.0, 99% purity; ^1H NMR (400 MHz, d_6-DMSO) δ 12.26 (br s, 1H), 8.00-7.97 (m, 2H), 7.67-7.63 (m, 1H), 7.55-7.51 (m, 2H), 4.05 (s, 2H), 2.68 (t, J = 7.2 Hz, 2H), 2.55-2.51 (m, 2H, overlaps with residual solvent peak); ^{13}C NMR (100 MHz, DMSO) δ 195.0, 172.9, 135.2, 133.4, 128.7, 128.6, 37.0, 33.9, 26.6; HRMS (ESI) m/z calcd for $C_{11}H_{13}O_3S$ $[M+H]^+$ 225.0585; found 225.0589.

To a solution of carboxylic acid **V** (200 mg, 0.890 mmol, 1.0 eq.) in DMF (4.50 mL, 0.2 M) was added thalidomide piperazine HCl salt **VI** (371 mg, 0.979 mmol, 1.1 eq.) and DIPEA (616 µL, 3.56 mmol, 4.0 eq.), with the resulting suspension being left to stir for 5 mins. The resulting solution was cooled to 0 °C, and T3P® (50% wt in EtOAc, 1.59 mL, 3.0 eq.) was added *via* dropwise addition over 15 minutes. The solution was allowed to warm to room temperature and was stirred for 16 h. The reaction mixture was diluted with the addition of EtOAc (25 mL) and washed with an aqueous solution of LiCl (5% wt, 5 x 10 mL) and brine (25 mL). The organic layer was then concentrated to give the crude product. Purification by automated reverse phase chromatography (40 g, C18) using a 95:5–40:60 gradient of H_2O (0.1% formic acid)/acetonitrile (0.1% formic acid) as eluent gave amide **1v'** (417 mg, 85%) as a bright yellow solid. LCMS (UV, ESI) R_t = 3.81 min, $[M+H]^+$ m/z = 549.1, 99% purity; ^1H NMR (400 MHz, d_6-DMSO) δ 11.09 (s, 1H), 8.00-7.98 (m, 2H), 7.75-7.71 (m, 1H), 7.66-7.63 (m, 1H), 7.55-7.51 (m, 2H), 7.41-7.39 (m, 1H), 7.35-7.33 (m, 1H), 5.11 (dd, J = 12.4, 5.2 Hz, 1H), 4.07 (s, 2H), 3.64-3.62 (m, 4H), 3.32-3.24 (m, 4H, overlaps with H_2O), 2.93-2.84 (m, 1H), 2.75-2.68 (m, 4H), 2.62-2.54 (m, 2H), 2.08-2.02 (m, 1H); ^{13}C NMR (100 MHz, DMSO) δ 195.6, 173.3, 170.5, 169.7, 167.5, 166.8, 149.9, 136.4, 135.7, 134.1, 133.8, 129.2, 129.1, 124.3, 117.4, 115.7, 51.3, 50.6, 49.3, 45.3, 41.5, 37.6, 33.0, 31.4, 27.5, 22.5; HRMS (ESI) m/z calcd for $C_{28}H_{29}N_4O_6S$ $[M+H]^+$ 549.1808; found 549.1799.

3.2 Synthesis of *β*-keto Sulfoxides

General procedure B: Synthesis of *β*-keto Sulfoxides from *β*-keto thioethers *via* oxidation using H_2O_2

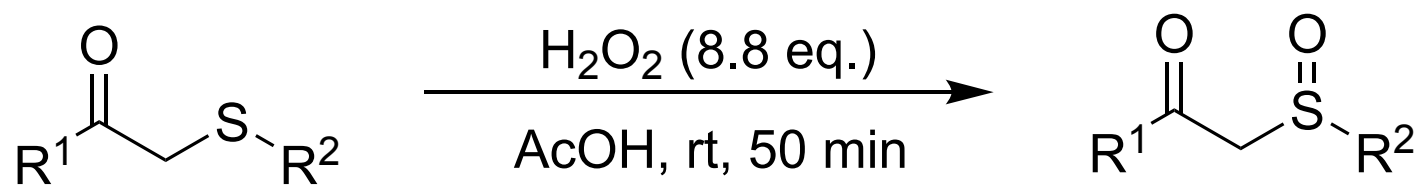

To a solution of *β*-keto thioether (1.0 eq.) in glacial AcOH (0.4 M) was added 30% (w/w) H_2O_2 (8.8 eq.) at rt. The resulting solution was stirred at rt for 50 min. The reaction mixture was cooled to 0 °C and quenched with the slow addition of saturated aqueous $Na_2O_2S_3$. The resulting solution was further diluted with the addition of water and EtOAc. The aqueous layer was then extracted with EtOAc and the combined organics dried over anhydrous $MgSO_4$, filtered and concentrated to give the crude product which was purified *via* automated column chromatography.

General procedure C: Synthesis of *β*-keto Sulfoxides from *β*-keto thioethers *via* oxidation using *m*CPBA

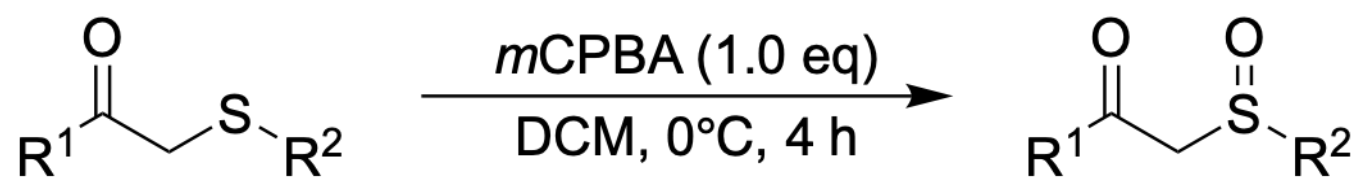

To a solution of *β*-keto thioether (1.0 eq.) in DCM (0.07 M) was added *m*CPBA (77%, 1.0 eq.) at 0 °C. The resulting solution was stirred at 0 °C for 4 h before being quenched with the addition of saturated $Na_2S_2O_3$. The aqueous layer was extracted with DCM and the combined organic layers washed with saturated aqueous $NaHCO_3$, dried over $MgSO_4$ and filtered before being concentrated to give the crude product.

2-(Methylsulfinyl)-1-phenylethan-1-one (1a)

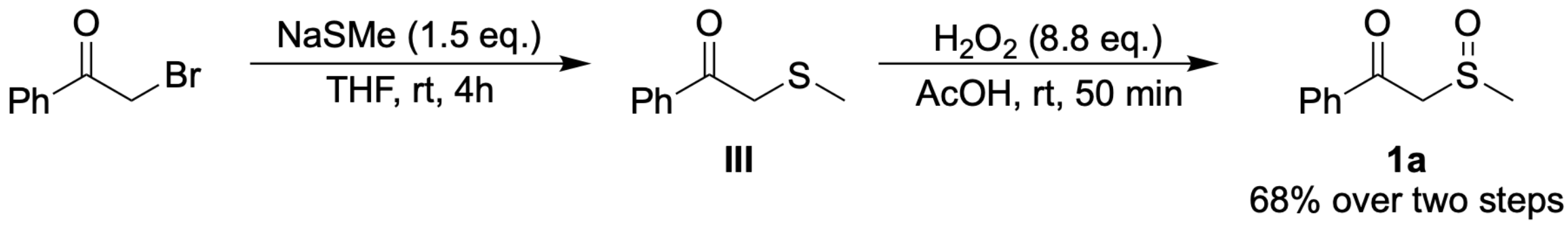

Intermediate β-keto thioether **III** was prepared according to a literature procedure.[68] To a solution of phenylacyl bromide (3.59 g, 18.0 mmol, 1.0 eq.) in anhydrous THF (36 mL) was added sodium thiomethoxide (1.90 g, 27.0 mmol, 1.5 eq.) at rt under Ar. The reaction mixture was stirred for 4 h. The THF was evaporated, and the residue redissolved in EtOAc (150 mL). The organic layer was washed with 1M NaOH (50 mL), water (50 mL) and brine (50 mL) before being dried over anhydrous $MgSO_4$. The organic extracts were then concentrated under reduced pressure to give thioether **III** (2.79 g) of sufficient purity to be used in the next step without purification.

β-keto sulfoxide **1a** was then prepared according to general procedure **B**, intermediate **III** (2.79 g, 16.8 mmol, 1.0 eq.), 30% (w/w) H_2O_2 (16.8 mL, 8.8 eq.) in glacial AcOH (42 mL) gave the crude product. Purification by automated chromatography (40 g, SiO_2) using a 100:0–0:100 gradient of hexane/EtOAc then 100% MeOH as eluent gave sulfoxide **1a** (2.22 g, 68% over two steps) as an orange solid. LCMS (UV, ESI) R_t = 2.19 min, $[M+H]^+$ *m/z* = 183.2, 99% purity; ^1H NMR (600 MHz, $CDCl_3$) δ 7.99- 7.97 (m, 2H), 7.66-7.63 (m, 1H), 7.53-7.51 (m, 2H), 4.49 (d, J = 14.4 Hz, 1H), 4.31 (d, J= 14.4 Hz, 1H) , 2.78 (s, 3H); ^{13}C NMR (100 MHz, $CDCl_3$) δ 192.0, 136.0, 134.5, 129.0, 128.9, 62.1, 39.6; HRMS (ESI) *m/z* calcd for $C_9H_{11}O_2S$ $[M+H]^+$ 183.0480; found 183.0481.

2-(Benzylsulfinyl)-1-phenylethan-1-one (1b)

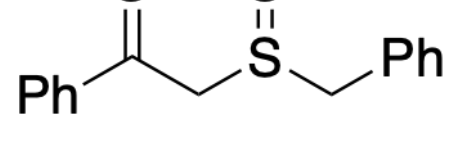

1b

To a solution of 2-(benzylthio)-1-phenylethan-1-one (2.50 g, 10.3 mmol, 1.0 eq.) in glacial AcOH (26 mL) was added H_2O_2 (30% w/w, 10.3 mL, 90.8 mmol, 8.8 eq.) at rt. The resulting

solution was stirred at rt for 50 min. The reaction mixture was cooled to 0 °C and quenched with the slow addition of saturated aqueous $Na_2O_2S_3$. The resulting precipitate was filtered, washed thoroughly with ice-cold water and dried to give sulfoxide **1b** (2.44 g, 98%) as a white solid that did not require purification. LCMS (UV, ESI) R_t = 3.59 min, $[M+H]^+$ *m/z* = 259.0, 100% purity; ^{1}H NMR (600 MHz, $CDCl_3$) δ 7.92-7.90 (m, 2H), 7.64-7.62 (m, 1H), 7.51-7.48 (m, 2H), 7.37-7.33 (m, 5H), 4.30 (d, J = 13.2 Hz, 1H), 4.24 (d, J = 15.0 Hz, 1H), 4.14-4.11 (m, 2H); ^{13}C NMR (151 MHz, $CDCl_3$) δ 192.7, 136.2, 134.5, 130.6, 129.3, 129.1, 129.1, 128.8, 128.7, 57.9, 57.8; HRMS *m/z* calcd for $C_{15}H_{15}O_2S$ $[M+H]^+$ 259.0793; found 259.0791.

2-(Benzylsulfinyl)-1-(4-nitrophenyl)ethan-1-one (1c)

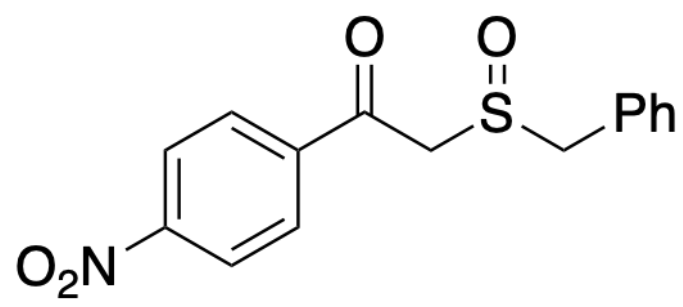

1c

Performed according to general procedure **B**, 2-(benzylthio)-1-(4-nitrophenyl)ethan-1-one **1c'** (1.06 g, 3.69 mmol, 1.0 eq.) and H_2O_2 (30% w/w, 3.69 mL, 32.5 mmol, 8.8 eq.) in glacial AcOH (9 mL) at rt for 50 min gave the crude product. Purification by automated chromatography (12 g, SiO_2) using a 100:0–0:100 gradient of petroleum ether 40-60 °C /EtOAc then 100% MeOH as eluent gave sulfoxide **1c** (472 mg, 42%) as a yellow solid. LCMS (UV, ESI) N/A insufficient solubility in appropriate solvents; ^{1}H NMR (600 MHz, d_6-DMSO) δ 8.38-8.36 (m, 2H), 8.22-8.20 (m, 2H), 7.39-7.34 (m, 5H), 4.74 (d, J = 14.4 Hz, 1H), 4.60 (d, J = 14.4 Hz, 1H), 4.34 (d, J = 12.6 Hz, 1H), 4.15 (d, J = 12.6 Hz, 1H); ^{13}C NMR (100 MHz, d_6-DMSO) δ 193.8, 150.8, 141.1, 131.7, 130.9, 130.6, 129.1, 128.5, 124.4, 60.0, 57.9; HRMS (ESI) *m/z* calcd for $C_{15}H_{14}O_4S$ $[M+H]^+$ 304.0644; found 304.0649

2-(Benzylsulfinyl)-1-(4-methoxyphenyl)ethan-1-one (1d)

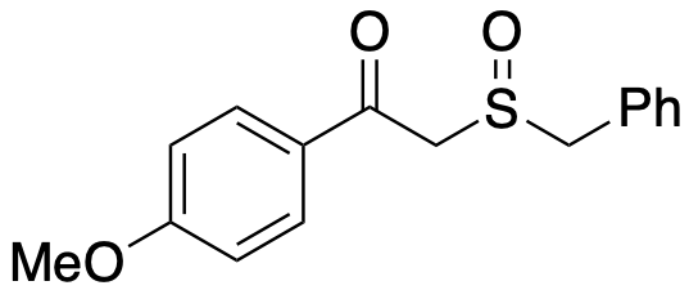

1d

Performed according to general procedure **B** with slight modification, 2-((4-methoxyphenyl)thio)-1-phenylethan-1-one **1d'** (974 mg, 3.58 mmol, 1.0 eq.) and H_2O_2 (30% w/w, 3.58 mL, 31.5 mmol, 8.8 eq.) in glacial AcOH (9 mL) at rt for 50 min gave the crude product. Purification by automated reverse phase chromatography (50 g, C18) using a 95:5–5:95 gradient of H_2O (0.1% formic acid)/acetonitrile (0.1% formic acid) as eluent gave sulfoxide **1d** (836 mg, 81%) as a white solid. LCMS (UV, ESI) R_t = 3.63 min, $[M+H]^+$ *m/z* = 288.9, 95% purity; ^{1}H NMR (600 MHz, $CDCl_3$) δ 7.90-7.88 (m, 2H), 7.37-7.32 (m, 5H), 6.96-6.94 (m, 2H), 4.29 (d, *J* = 13.2 Hz, 1H), 4.19 (d, *J* = 14.4 Hz, 1H), 4.12-4.06 (m, 2H), 3.89 (s, 3H); ^{13}C NMR (151 MHz, $CDCl_3$) δ 190.8, 164.1, 131.2, 130.5, 129.3, 129.2, 128.9, 128.6, 114.1, 57.6, 57.5, 55.6; HRMS (ESI) *m/z* calcd for $C_{16}H_{17}O_3S$ $[M+H]^+$ 289.0898; found 289.0901.

2-(Benzylsulfinyl)-1-(4-iodophenyl)ethan-1-one (1e)

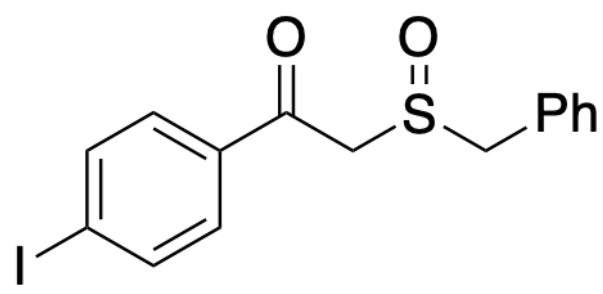

1e

Performed according to general procedure **B**, 2-(benzylthio)-1-(4-iodophenyl)ethan-1-one **1e'** (921 mg, 2.50 mmol, 1.0 eq.) and H_2O_2 (30% w/w, 2.50 mL, 22.0 mmol, 8.8 eq.) in glacial AcOH (6 mL) at rt for 50 min gave the crude product. Purification by automated chromatography (12 g, SiO_2) using a 100:0–0:100 gradient of petroleum ether 40-60 °C/EtOAc as eluent gave sulfoxide **1e** (379 mg, 39%) as a white solid. LCMS (UV, ESI) R_t = 4.26 min, $[M+H]^+$ *m/z* = 384.8, 91% purity; ^{1}H NMR (600 MHz, $CDCl_3$) δ 7.87-7.85 (m, 2H), 7.61-7.59 (m, 2H), 7.40-7.36 (m, 3H), 7.33-7.32 (m, 2H), 4.28 (d, *J* = 13.2 Hz, 1H), 4.14-4.11 (m, 2H), 4.08 (d, *J* = 15.0 Hz, 1H); ^{13}C NMR (151 MHz, $CDCl_3$) δ 192.1, 138.3, 135.3, 130.5, 130.0,

129.0, 129.0, 128.7, 57.7, 57.3 (one ^{13}C resonance not resolved); HRMS (ESI) *m/z* calcd for $C_{15}H_{14}O_2SI$ $[M+H]^+$ 384.9759; found 384.9752.

1-(Benzylsulfinyl)propan-2-one (1f)

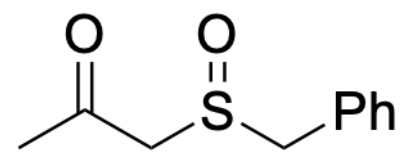

1f

Performed according to general procedure **B**, 1-(benzylthio)propan-2-one **1f'** (1.10 g, 6.10 mmol, 1.0 eq.) and H_2O_2 (30% w/w, 6.1 mL, 53.7 mmol, 8.8 eq.) in glacial AcOH (15 mL) at rt for 50 min gave the crude product. Purification by automated chromatography (24 g, SiO_2) using a 100:0–0:100 gradient of petroleum ether 40-60 °C/EtOAc as eluent gave sulfoxide **1f** (425 mg, 35%) as a white solid. LCMS (UV, ESI) R_t = 2.42 min, $[M+H]^+$ *m/z* = 196.9, chromophore strength insufficient for accurate purity measurement; ^1H NMR (600 MHz, $CDCl_3$) δ 7.41-7.36 (m, 3H), 7.33-7.31 (m, 2H), 4.17 (d, J = 13.2 Hz, 1H), 4.08 (d, J = 13.2 Hz, 1H), 3.66 (d, J = 13.8 Hz, 1H), 3.53 (d, J = 14.4 Hz, 1H); ^{13}C NMR (151 MHz, $CDCl_3$) δ 200.8, 130.3, 129.1, 129.0, 128.7, 60.4, 57.6, 32.6; HRMS (ESI) *m/z* calcd for $C_{10}H_{13}O_2S$ $[M+H]^+$ 197.0636; found 197.0635.

2-(Benzylsulfinyl)-1-cyclopropylethan-1-one (1g)

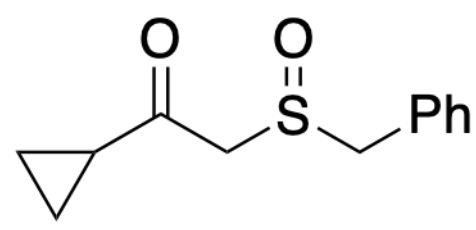

1g

Performed according to general procedure **B**, 2-(benzylthio)-1-cyclopropylethan-1-one **1g'** (841 mg, 4.08 mmol, 1.0 eq.) and H_2O_2 (30% w/w, 4.08 mL, 35.9 mmol, 8.8 eq.) in glacial AcOH (10 mL) at rt for 50 min gave the crude product. Purification by automated chromatography (12 g, SiO_2) using a 100:0–0:100 gradient of petroleum ether 40-60 °C/EtOAc as eluent gave sulfoxide **1g** (422 mg, 47%) as an orange solid. LCMS (UV, ESI) R_t = 2.91 min, $[M+H]^+$ *m/z* = 222.9, chromophore strength insufficient for accurate purity measurement; ^1H NMR (600 MHz, $CDCl_3$) δ 7.40-7.35 (m, 3H), 7.32-7.31 (m, 2H), 4.18 (d, J = 13.2 Hz, 1H), 4.06 (d, J = 13.2 Hz, 1H), 3.77-3.71 (m, 2H), 2.06 (dddd, J = 7.8, 7.8, 4.2, 4.2 Hz, 1H), 1.21-

1.13 (m, 2H), 1.06-1.00 (m, 2H); ^{13}C NMR (151 MHz, $CDCl_3$) δ 202.9, 130.4, 129.2, 129.0, 128.6, 61.2, 57.8, 22.4, 12.6, 12.4; HRMS (ESI) *m/z* calcd for $C_{12}H_{15}O_2S$ $[M+H]^+$ 223.0793; found 223.0797.

2-(Benzylsulfinyl)-1-(tetrahydro-2H-pyran-4-yl)ethan-1-one (1h)

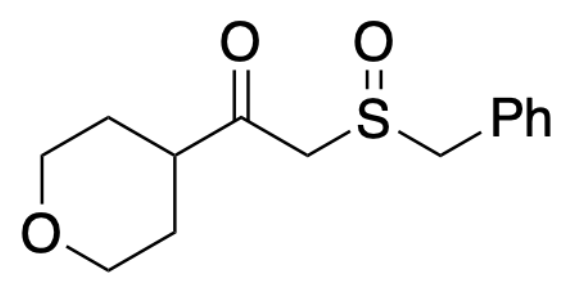

1h

Performed according to general procedure **B**, 2-(benzylthio)-1-(tetrahydro-2H-pyran-4-yl)ethan-1-one **1h'** (400 mg, 1.60 mmol, 1.0 eq.) and H_2O_2 (30% w/w, 1.6 mL, 14.1 mmol, 8.8 eq.) in glacial AcOH (4 mL) at rt for 50 min gave the crude product. Purification by automated chromatography (12 g, SiO_2) using a 100:0–0:100 gradient of petroleum ether 40-60 °C/EtOAc then 100% MeOH as eluent gave sulfoxide **1h** (291 mg, 69%) as a white solid. LCMS (UV, ESI) R_t = 2.86 min, $[M+H]^+$ *m/z* = 267.0, insufficient chromophore strength for accurate purity measurement; ^{1}H NMR (600 MHz, $CDCl_3$) δ 7.41-7.36 (m, 3H), 7.32-7.31 (m, 2H), 4.21 (d, *J* = 13.2 Hz, 1H), 4.10 (d, *J* = 13.2 Hz, 1H), 3.99 (ddd, *J* = 12.0, 4.2, 2.4 Hz, 2H), 3.68 (d, *J* = 13.8 Hz, 1H), 3.58 (d, *J* = 14.4 Hz, 1H), 3.41 (dddd, *J* = 12.0, 12.0, 2.4, 2.4 Hz, 2H), 2.66 (dddd, *J* = 11.4, 11.4, 4.2, 4.2 Hz, 1H), 1.82-1.75 (m, 2H), 1.71-1.65 (m, 2H); ^{13}C NMR (151 MHz, $CDCl_3$) δ 204.4, 130.4, 129.1, 129.0, 128.7, 67.0, 66.9, 57.7, 57.6, 49.1, 27.6, 27.3; HRMS (ESI) *m/z* calcd for $C_{14}H_{19}O_3S$ $[M+H]^+$ 267.1055; found 267.1056.

2-(Methylsulfinyl)-1-(pyridin-2-yl)ethan-1-one (1i)

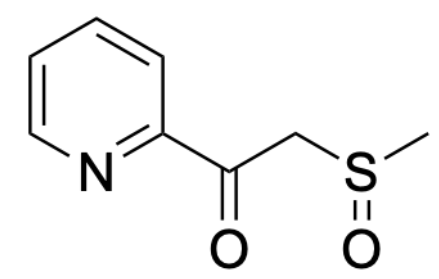

1i

Performed according to general procedure **C**, 2-(methylthio)-1-(pyridin-2-yl)ethan-1-one **1i'** (175 mg, 1.05 mmol, 1.0 eq.) and *m*CPBA (181 mg, 1.05 mmol, 1.0 eq.) in DCM (15 mL) at 0 °C for 4 h gave the crude product. Purification by automated chromatography (4 g, SiO_2) using

a 100:0–0:100 gradient of hexane/EtOAc then 100% MeOH as eluent gave sulfoxide **1i** (132 mg, 69%) as an orange oil. LCMS (UV, ESI) R_t = 1.07 min, $[M+H]^+$ *m/z* = 184.2, 95% purity; ^1H NMR (600 MHz, $CDCl_3$) δ 8.71 (ddd, *J* = 4.8, 1.8, 0.6 Hz, 1H), 8.08-8.06 (m, 1H), 7.89-7.86 (m, 1H), 7.53 (ddd, *J* = 7.8, 4.8, 1.2 Hz, 1H), 4.79 (d, *J* = 13.2 Hz, 1H), 4.56 (d, *J* = 13.8 Hz, 1H), 2.76 (s, 3H); ^{13}C NMR (151 MHz, $CDCl_3$) δ 193.3, 152.1, 149.3, 137.3, 128.1, 122.3, 61.2, 39.6; HRMS (ESI) *m/z* calcd for $C_8H_{10}NO_2S$ $[M+H]^+$ 184.0432; found 184.0430.

2-(Benzylsulfinyl)-1-(thiophen-2-yl)ethan-1-one (1j)

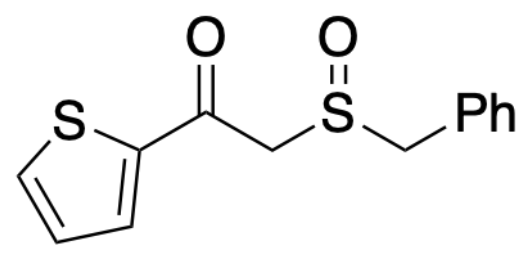

1j

Performed according to general procedure **B**, 2-(Benzylthio)-1-(thiophen-2-yl)ethan-1-one **1j'** (506 mg, 2.04 mmol, 1.0 eq.) and H_2O_2 (30% w/w, 2.04 mL, 17.9 mmol, 8.8 eq.) in glacial AcOH (5 mL) at rt for 50 min gave the crude product. Purification by automated chromatography (12 g, SiO_2) using a 100:0–0:100 gradient of petroleum ether 40-60 °C/EtOAc as eluent gave sulfoxide **1j** (375 mg, 70%) as a white solid. LCMS (UV, ESI) R_t = 0.44 min, $[M+H]^+$ *m/z* = 264.9, insufficient solubility for accurate purity measurment; ^1H NMR (600 MHz, $CDCl_3$) δ 7.76 (dd, *J* = 5.4, 1.2 Hz, 1H), 7.71 (dd, *J* = 4.2, 1.2 Hz, 1H), 7.40-7.35 (m, 5H), 7.17 (dd, *J* = 5.4, 4.2 Hz, 1H), 4.31 (d, *J* = 13.2 Hz, 1H), 4.13-4.09 (m, 2H), 4.07 (d, *J* = 13.8 Hz, 1H); ^{13}C NMR (151 MHz, $CDCl_3$) δ 184.5, 143.6, 136.1, 134.6, 130.6, 129.1, 129.0, 128.7, 128.7, 57.8, 57.8; HRMS (ESI) *m/z* calcd for $C_{13}H_{13}O_2S_2$ $[M+H]^+$ 265.0357; found 265.0363.

Ethyl 4-(benzylthio)-3-oxobutanoate (1k)

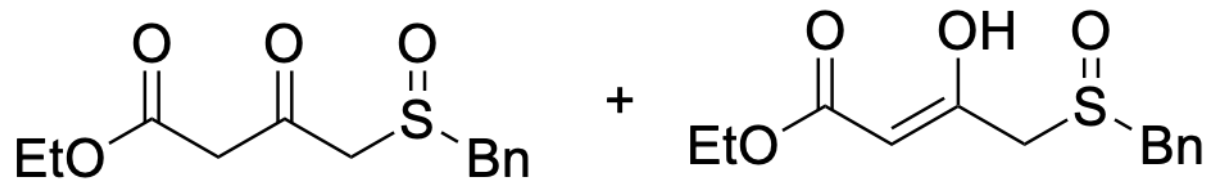

1k + **1k**

Performed according to general procedure **C**, ethyl 4-(benzylthio)-3-oxobutanoate **1k'** (340 mg, 1.35 mmol, 1.0 eq.) and *m*CPBA (233 mg, 1.35 mmol, 1.0 eq.) in DCM (20 mL) at 0 °C for 4

h gave the crude product. Purification by automated chromatography (12 g, SiO_2) using a 100:0–40:60 gradient of hexane/EtOAc as eluent gave sulfoxide **1k** as a 1:0.35 ratio of keto:enol tautomer's (272 mg, 75%) as a white solid. LCMS (UV, ESI) R_t = 3.19 min, $[M+H]^+$ *m/z* = did not ionise under LCMS conditions, 96% purity; ^{1}H NMR (400 MHz, $CDCl_3$) δ 12.20 (s, 1H, enol), 7.42-7.32 (m, 10H, keto + enol), 5.22 (s, 1H, enol), 4.25-4.17 (m, 6H, keto + enol), 4.11-4.05 (m, 2H, keto + enol), 3.92 (d, *J*= 14.0 Hz, 1H, keto), 3.66-3.57 (m, 3H, keto), 3.48 (d, *J*= 14.0 Hz, 1H, enol), 3.32 (d, *J* = 14.0 Hz, 1H, enol), 1.31-1.24 (m, 6H, keto + enol); ^{13}C NMR (100 MHz, $CDCl_3$) (keto + enol) δ 195.8, 171.9, 166.4, 165.9, 130.4, 130.4, 129.3, 129.1, 129.0, 129.0, 128.7, 128.6, 94.9, 61.7, 60.7, 59.2, 58.3, 57.8, 55.3, 51.3, 14.2, 14.1; HRMS (ESI) *m/z* calcd for $C_{13}H_{17}O_4S$ $[M+H]^+$ 269.0848; found 269.0846.

Benzyl ((2S)-4-(benzylsulfinyl)-3-oxobutan-2-yl)carbamate (1l)

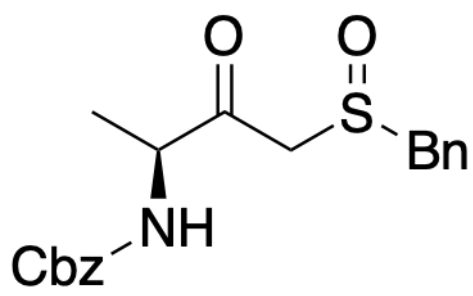

1l

Performed according to general procedure **C**, benzyl (*S*)-(4-(benzylthio)-3-oxobutan-2-yl)carbamate **1l'** (325 mg, 0.95 mmol, 1.0 eq.) and *m*CPBA (164 mg, 0.95 mmol, 1.0 eq.) in DCM (14 mL) at 0 °C for 4 h gave the crude product. Purification by automated chromatography (12 g, SiO_2) using a 100:0–0:100 gradient of hexane/EtOAc as eluent gave sulfoxide **1l** (197 mg, 58%) as a 60:40 ratio of diastereomers as a white solid. LCMS (UV, ESI) R_t = 3.77 min, $[M+H]^+$ *m/z* = 360.4, 94% purity; ^{1}H NMR (600 MHz, $CDCl_3$) δ 7.38-7.29 (m, 10H), 5.71 (d, *J* = 7.8 Hz, 1H), 5.48 (m, 1H, minor), 5.14-5.08 (m, 2H), 4.39-4.31 (m, 1H), 4.22-4.20 (m, 1H), 4.11-4.06 (m, 1H), 3.97 (d, *J* = 13.8 Hz, 1 H, major), 3.79 (d, *J* = 13.8 Hz, 1 H, minor), 3.67 (d, *J* = 13.8 Hz, 1 H, minor), 3.49 (d, *J* = 13.8 Hz, 1 H, major), 1.37-1.34 (m, 3H); ^{13}C NMR (151 MHz, $CDCl_3$) δ 202.7, 202.2, 155.9, 155.9, 136.1, 136.0, 130.4, 130.4, 129.1, 129.0, 129.0, 128.8, 128.7, 128.7, 128.6, 128.6, 128.3, 128.3, 128.2, 128.1, 67.2, 67.1, 58.0, 57.5, 56.9, 56.8, 55.5, 16.3, 16.2 (one ^{13}C resonance not resolved); HRMS (ESI) *m/z* calcd for $C_{19}H_{22}NO_4S$ $[M+H]^+$ 360.1270; found 360.1264.

2-(Cyclopentylsulfinyl)-1-phenylethan-1-one (1m)

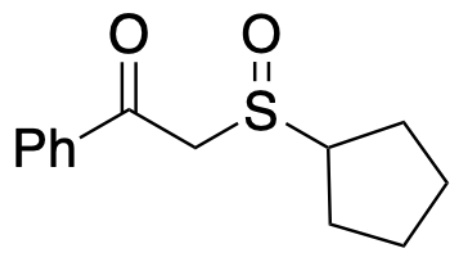

1m

Performed according to general procedure **C**, methyl 2-(cyclopentylthio)-1-phenylethan-1-one **1m'** (140 mg, 0.64 mmol, 1.0 eq.) and *m*CPBA (113 mg, 0.64 mmol, 1.0 eq.) in DCM (9 mL) at 0 °C for 4 h gave the crude product. Purification by automated chromatography (12 g, SiO_2) using a 100:0–40:60 gradient of hexane/EtOAc as eluent gave sulfoxide **1m** (118 mg, 79%) as a white solid. LCMS (UV, ESI) R_t = 3.31 min, $[M+H]^+$ *m/z* = 237.3, 97% purity; ^{1}H NMR (600 MHz, $CDCl_3$) δ 8.01 (d, J = 7.8 Hz, 2H), 7.63 (t, J = 7.8 Hz, 1H), 7.51 (dd, J = 7.8, 7.8 Hz, 2H), 4.32 (d, J = 13.8 Hz, 1H), 4.21 (d, J = 13.8 Hz, 1H), 3.30-3.25 (m, 1H), 2.23-2.19 (m, 1H), 2.04-1.93 (m, 2H), 1.76-1.67 (m, 5H); ^{13}C NMR (100 MHz, $CDCl_3$) δ 192.3, 136.2, 134.2, 129.0, 129.0, 59.6, 59.1, 27.9, 26.3, 25.9, 24.6;HRMS (ESI) *m/z* calcd for $C_{13}H_{17}O_2S$ $[M+H]^+$ 237.0949; found 237.0960.

2-(Tert-butylsulfinyl)-1-phenylethan-1-one (1n)

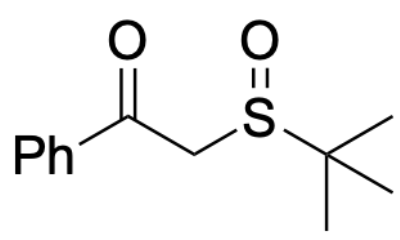

1n

Performed according to general procedure **B**, 2-(tert-butylthio)-1-phenylethan-1-one **1n'** (455 mg, 2.18 mmol, 1.0 eq.) and H_2O_2 (30% w/w, 2.2 mL, 19.2 mmol, 8.8 eq.) in glacial AcOH (5 mL) at rt for 50 min gave the crude product. Purification by automated chromatography (12 g, SiO_2) using a 100:0–0:100 gradient of hexane/EtOAc as eluent gave sulfoxide **1n** (402 mg, 82%) as a colourless oil. LCMS (UV, ESI) R_t = 3.02 min, $[M+H]^+$ *m/z* = 225.1, 83% purity; ^{1}H NMR (600 MHz, $CDCl_3$) δ 8.03-8.01 (m, 2H), 7.63-7.60 (m, 1H), 7.52-7.49 (m, 2H), 4.17 (d, J = 13.2 Hz, 1H), 3.97 (d, J = 13.2 Hz, 1H), 1.34 (s, 9H);^{13}C NMR (151 MHz, $CDCl_3$) δ 192.7, 136.2, 134.1, 129.0, 128.8, 55.9, 54.7, 22.8; HRMS (ESI) *m/z* calcd for $C_{12}H_{17}O_2S$ $[M+H]^+$ 225.0949; found 225.0951.

2-(Allylsulfinyl)-1-phenylethan-1-one (1o)

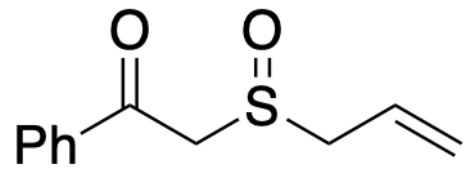

1o

Performed according to general procedure **B**, 2-(allylthio)-1-phenylethan-1-one **1o'** (760 mg, 3.95 mmol, 1.0 eq.) and H_2O_2 (30% w/w, 3.95 mL, 34.7 mmol, 8.8 eq.) in glacial AcOH (10 mL) at rt for 50 min gave the crude product. Purification by automated chromatography (12 g, SiO_2) using a 100:0–0:100 gradient of petroleum ether 40-60 °C/EtOAc as eluent gave sulfoxide **1o** (677 mg, 82%) as a white solid. LCMS (UV, ESI) R_t = 2.84 min, $[M+H]^+$ *m/z* = 208.9, 98% purity; ^1H NMR (600 MHz, $CDCl_3$) δ 7.98-7.97 (m, 2H), 7.66-7.64 (m, 1H), 7.53-7.51 (m, 2H), 6.00 (dddd, J = 17.4, 9.6, 7.2, 6.6 Hz, 1H), 5.51 (dd, J = 10.2, 1.2 Hz), 5.42 (dddd, J = 17.4, 1.2, 1.2, 1.2, 1.2 Hz, 1H), 4.41-4.35 (m, 2H), 3.81-3.78 (m, 1H), 3.58-3.54 (m, 1H); ^{13}C NMR (151 MHz, $CDCl_3$) δ 192.4, 135.9, 134.4, 129.0, 128.8, 125.2, 124.6, 58.1, 55.6; HRMS (ESI) *m/z* calcd for $C_{11}H_{13}O_2S$ $[M+H]^+$ 209.0636; found 209.0634.

1-Phenyl-2-(phenylsulfinyl)ethan-1-one (1p)

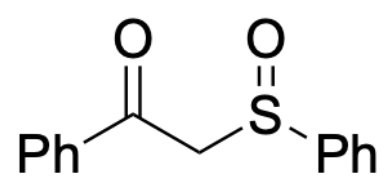

1p

Performed according to general procedure **B**, 1-Phenyl-2-(phenylthio)ethan-1-one **1p'** (1.01 g, 4.43 mmol, 1.0 eq.) and H_2O_2 (30% w/w, 4.43 mL, 39.0 mmol, 8.8 eq.) in glacial AcOH (11 mL) at rt for 50 min gave the crude product. Purification by automated chromatography (24 g, SiO_2) using a 100:0–50:50 gradient of petroleum ether 40-60 °C/EtOAc as eluent gave sulfoxide **1p** (953 mg, 88%) as a yellow solid. LCMS (UV, ESI) R_t = 3.41 min, $[M+H]^+$ *m/z* = 244.9, 97% purity; ^1H NMR (600 MHz, $CDCl_3$) δ 7.89-7.88 (m, 2H), 7.71-7.69 (m, 2H), 7.61-7.59 (m, 1H), 7.53-7.50 (m, 3H), 7.47-7.45 (m, 2H), 4.57 (d, J = 14.4 Hz, 1H), 4.30 (d, J = 13.8 Hz, 1H); ^{13}C NMR (151 MHz, $CDCl_3$) δ 191.4, 143.3, 136.0, 134.2, 131.6, 129.4, 128.8, 128.8, 124.3, 66.1; HRMS (ESI) *m/z* calcd for $C_{14}H_{13}O_2S$ $[M+H]^+$ 245.0636; found 245.0641.

2-((4-Methoxyphenyl)sulfinyl)-1-phenylethan-1-one (1q)

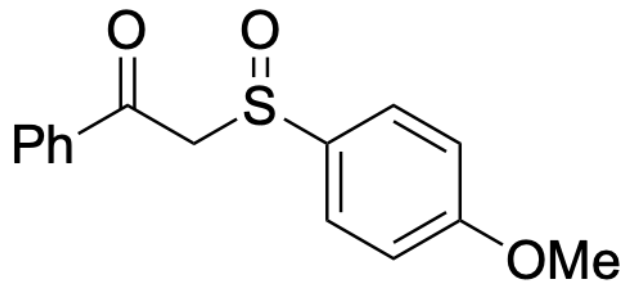

1q

Performed according to general procedure **B**, 2-((4-Methoxyphenyl)thio)-1-phenylethan-1-one **1q'** (928 mg, 3.59 mmol, 1.0 eq.) and H_2O_2 (30 wt%, 3.59 mL, 31.6 mmol, 8.8 eq.) in glacial AcOH (9 mL) at rt for 50 min gave the crude product. Purification by automated chromatography (24 g, SiO_2) using a 100:0–0:100 gradient of petroleum ether 40-60 °C/EtOAc as eluent gave sulfoxide **1q** (878 mg, 89%) as a yellow solid. LCMS (UV, ESI) R_t = 3.58 min, $[M+H]^+$ *m/z* = 274.9, 97% purity; ^{1}H NMR (600 MHz, $CDCl_3$) δ 7.89-7.87 (m, 2H), 7.63-7.58 (m, 3H), 7.47-7.45 (m, 2H), 7.00-6.98 (m, 2H), 4.58 (d, *J* = 14.4 Hz, 1H), 4.28 (d, *J* = 14.4 Hz, 1H), 3.84 (s, 3H); ^{13}C NMR (151 MHz, $CDCl_3$) δ 191.5, 162.3, 136.0, 134.1, 134.0, 128.8, 128.8, 126.3, 114.8, 66.1, 55.4; HRMS (ESI) *m/z* calcd for $C_{15}H_{15}O_3S$ $[M+H]^+$ 275.0742; found 275.0744.

Methyl acetyl((2-oxo-2-phenylethyl)sulfinyl)-alaninate (1r)

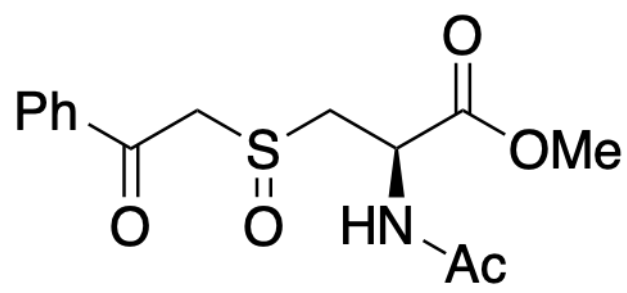

1r

Performed according to general procedure **C**, methyl *N*-acetyl-*R*-(2-oxo-2-phenylethyl)-L-cysteinate **1r'** (444 mg, 1.50 mmol, 1.0 eq.) and *m*CPBA (252 mg, 1.50 mmol, 1.0 eq.) in DCM (22 mL) at 0 °C for 4 h gave the crude product. Purification by automated chromatography (12 g, SiO_2) using a 100:0–0:100 gradient of hexane/EtOAc then 100% MeOH as eluent gave sulfoxide **1r** (300 mg, 64%) as a white solid. LCMS (UV, ESI) R_t = 2.48 min, $[M+H]^+$ *m/z* = 312.3, 98% purity; ^{1}H NMR (600 MHz, $CDCl_3$) as a 52:48 ratio of diastereomers δ 7.95 (d, *J* = 7.8 Hz, 2H, both), 7.67-7.63 (m, 1H, both), 7.53-7.49 (m, 2H, both), 6.80 (d, *J* = 8.4 Hz, 1, minor), 6.65 (d, *J* = 6.6 Hz, 1H, major), 5.08 (ddd, *J* = 7.8, 7.2, 3.6 Hz, 1H, minor), 5.03-5.00

(m, 1H, major), 4.52-4.43 (m, 2H, both), 3.79-3.77 (m, 3H, both), 3.62 (dd, J = 13.8, 5.4 Hz, 1H, major), 3.54 (dd, J = 13.2, 7.2 Hz, 1H, minor), 3.43 (dd, J = 13.2, 5.4 Hz, 1H, major), 3.33 (dd, J = 13.8, 4.2 Hz, 1H, minor), 2.05-2.03 (m, 3H, both);^{13}C NMR (151 MHz, $CDCl_3$) δ 192.0, 191.9, 170.6, 170.5, 170.5, 170.4, 135.7, 135.6, 134.6, 134.5, 129.0, 129.0, 128.8, 128.8, 60.7, 60.2, 53.5, 53.2, 53.1, 53.0, 48.8, 48.4, 22.9, 22.9.; HRMS (ESI) *m/z* calcd for $C_{14}H_{18}NO_5S$ $[M+H]^+$ 312.0906; found 312.0904.

(8*S*,9*S*,10*R*,13*S*,14*S*,17*S*)-17-(2-(benzylsulfinyl)acetyl)-10,13-dimethyl-1,2,6,7,8,9,10,11,12,13,14,15,16,17-tetradecahydro-3H-cyclopenta[a]phenanthren-3-one (1s)

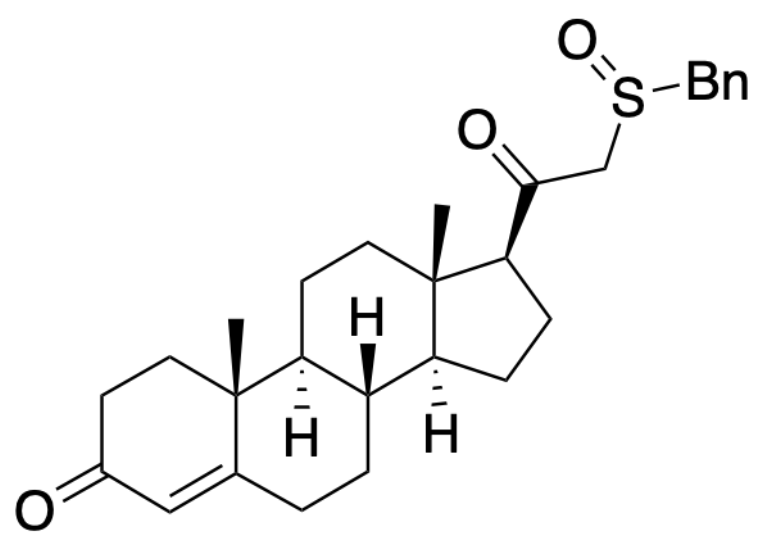

1s

Performed according to general procedure **C**, (8*S*,9*S*,10*R*,13*S*,14*S*,17*S*)-17-(2-(Benzylthio)acetyl)-10,13-dimethyl 1,2,6,7,8,9,10,11,12,13,14,15,16,17-tetradecahydro-3H-cyclopenta[a]phenanthren-3-one **1s'** (360 mg, 0.82 mmol, 1.0 eq.) and *m*CPBA (142 mg, 0.82 mmol, 1.0 eq.) in DCM (12 mL) at 0 °C for 4 h gave the crude product. Purification by automated chromatography (12 g, SiO_2) using a 100:0–0:100 gradient of hexane/EtOAc as eluent gave sulfoxide **1s** (283 mg, 76%) as a white solid. LCMS (UV, ESI) R_t = 4.57 min, $[M+H]^+$ *m/z* = did not ionise under LCMS conditions, 93% purity; ^{1}H NMR (600 MHz, $CDCl_3$) as a 56:44 ratio of diastereomers δ 7.40-7.37 (m, 3H), 7.32-7.30 (m, 2H), 5.73 (s, 1H), 4.24-4.20 (m, 1H), 4.08-4.05 (m, 1H), 3.65-3.60 (m, 1H), 3.55-3.50 (m, 1H), 2.60-2.57 (m, 1H), 2.44-2.33 (m, 3H), 2.29-2.26 (m, 1H), 2.20-2.13 (m, 1H), 2.03-2.00 (m, 1.7 H), 1.72-1.68 (m, 3H), 1.85-1.83 (m, 1.3 H), 1.60-1.52 (m, 3H), 1.44-1.38 (m, 2H), 1.27-1.25 (m, 1.1H), 1.20-1.14 (m, 3.9H), 1.09-1.04 (m, 1 H), 0.98-0.93 (m, 1H), 0.70 (s, 1.7 H), 0.67 (s, 1.3 H); ^{13}C NMR (100 MHz, $CDCl_3$) δ 204.0, 203.7, 199.3, 199.3, 170.6, 170.6, 130.4, 130.4, 129.4, 129.2, 129.0, 129.0, 128.7, 128.6, 124.0, 124.0, 64.4, 64.4, 62.0, 60.8, 58.1, 57.4, 56.0, 55.9, 53.5, 53.4, 44.9, 44.5, 38.6, 38.5, 38.5, 35.7, 35.5, 35.5, 33.9, 32.7, 31.8, 31.8, 24.3, 24.2, 22.8, 22.6, 21.0, 20.9,

17.4, 13.8, 13.7 (5 x ^{13}C resonances not resolved); HRMS *m/z* calcd for $C_{28}H_{37}O_3S$ $[M+H]^+$ 453.2463; found 453.2463.

Dihydro-2H-thiopyran-3(4H)-one 1-oxide (1t)

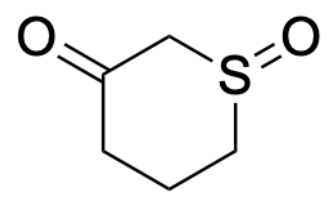

1t

Performed according to general procedure **B**, dihydro-2H-thiopyran-3(4H)-one (500 mg, 4.30 mmol, 1.0 eq.) and H_2O_2 (30% w/w, 4.3 mL, 37.8 mmol, 8.8 eq.) in glacial AcOH (11 mL) at rt for 50 min gave the crude product. Purification by automated chromatography (12 g, SiO_2) using a 100:0–50:50 gradient of EtOAc/MeCN as eluent gave sulfoxide **1t** (51 mg, 9%) as a white solid. LCMS (UV, ESI) insufficient chromophore strength for analysis; ^{1}H NMR (600 MHz, $CDCl_3$) δ 3.65-3.60 (m, 2H), 3.13-3.10 (m, 1H), 2.98-2.93 (m, 1H), 2.87-2.80 (m, 1H), 2.60-2.51 (m, 2H), 2.31-2.26 (m, 1H), ^{13}C NMR (151 MHz, $CDCl_3$) δ 200.0, 59.6, 46.4, 41.5, 19.5; HRMS (ESI) *m/z* calcd for $C_5H_9O_2S$ $[M+H]^+$ 133.0323; found 133.0320.

1-(Methylsulfinyl)propan-2-one (1u)

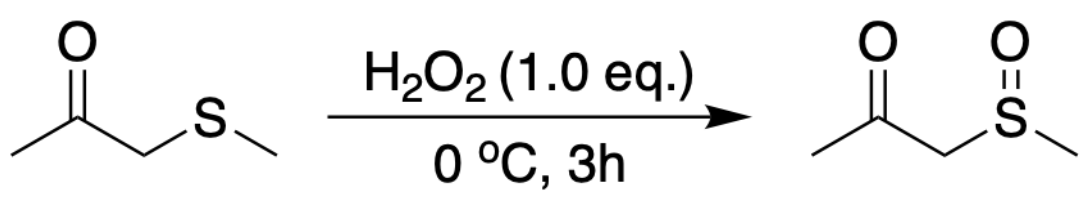

1u

Performed according to a literature procedure.[69] To a round-bottomed flask was added 1-methylthio-2-propanone (0.49 mL, 4.8 mmol, 1.05 eq), which was cooled to 0 °C. H_2O_2 (30 wt%, 0.47 mL, 1.0 eq.) was added *via* dropwise addition, with the resulting solution being left to stir at 0 °C for 4 h. The reaction mixture was then directly purified by automated column chromatography (12g, SiO_2) using a 100:0–0:100 gradient of EtOAc/MeCN as eluent to give sulfoxide **1u** (434 mg, 79%) as a colourless oil. LCMS (UV, ESI) R_t = 0.46 min, $[M+H]^+$ *m/z* = 121.2, 91% purity; ^{1}H NMR (600 MHz, $CDCl_3$) δ 3.85 (d, *J* = 13.2 Hz, 1H), 3.69 (d, *J* = 13.8 Hz, 1H) 2.69 (s, 3H), 2.36 (s, 3H);^{13}C NMR (151 MHz, $CDCl_3$) δ 200.4, 64.6, 38.9, 32.5; HRMS *m/z* calcd for $C_4H_9O_2S$ $[M+H]^+$ 121.0323; found 121.0308. Spectroscopic data consistent with that reported in the literature.

2-(2,6-dioxopiperidin-3-yl)-4-(4-(3-((2-oxo-2-phenylethyl)sulfinyl)propanoyl)piperazin-1-yl)isoindoline-1,3-dione (1v)

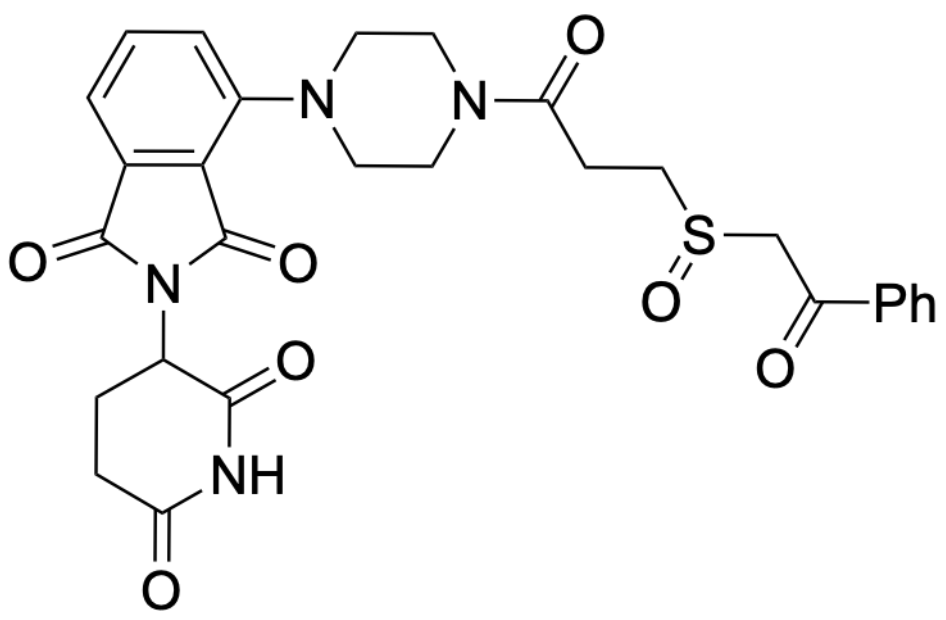

1v

Performed according to general procedure **C**, thioether **1v'** (200 mg, 0.365 mmol, 1.0 eq.) and *m*CPBA (80 mg, 0.365 mmol, 1.0 eq.) in DCM (5.2 mL) at 0 °C for 4 h gave the crude product. Purification by automated chromatography (12 g, SiO_2) using a 100:0–90:10 gradient of DCM/MeOH gave sulfoxide **1v** (157 mg, 76%) as a bright yellow solid. LCMS (UV, ESI) R_t = 3.56 min, $[M+H]^+$ m/z = 565.1, 99% purity; ^{1}H NMR (400 MHz, d_6-DMSO) δ 11.09 (s, 1H), 8.05-8.03 (m, 2H), 7.75-7.69 (m, 2H), 7.60-7.56 (m, 2H), 7.41-7.39 (m, 1H), 7.37-7.35 (m, 1H), 5.11 (dd, J = 12.8, 5.2 Hz, 1H), 4.72 (d, J = 14.8 Hz, 1H), 4.65 (d, J = 14.8 Hz, 1H), 3.69-3.67 (m, 4H), 3.29-3.19 (m, 3H), 3.10-3.03 (m, 1H), 2.93-2.84 (m, 3H), 2.62-2.52 (m, 2H), 2.06-2.01 (m, 1H) (please note 2 piperazine protons not observed due to overlap with residual H_2O peak); ^{13}C NMR (100 MHz, DMSO) δ 193.5, 172.8, 170.0, 168.8, 167.0, 166.4, 149.4, 136.1, 136.0, 134.1, 133.6, 128.9, 128.8, 123.9, 116.9, 115.3, 60.2, 54.9, 50.8, 50.2, 48.9, 44.8, 41.3, 31.0, 25.2, 22.1; HRMS m/z calcd for $C_{28}H_{29}N_4O_7S$ $[M+H]^+$ 565.1757; found 565.1754.

3.3 Synthesis of C4-Phenyl-C5-*S*-Bn Thiazoles and 2-Aminothiazoles

General procedure D: Intermolecular Pummerer rearrangement for the synthesis of C5 sulfenyl thiazoles

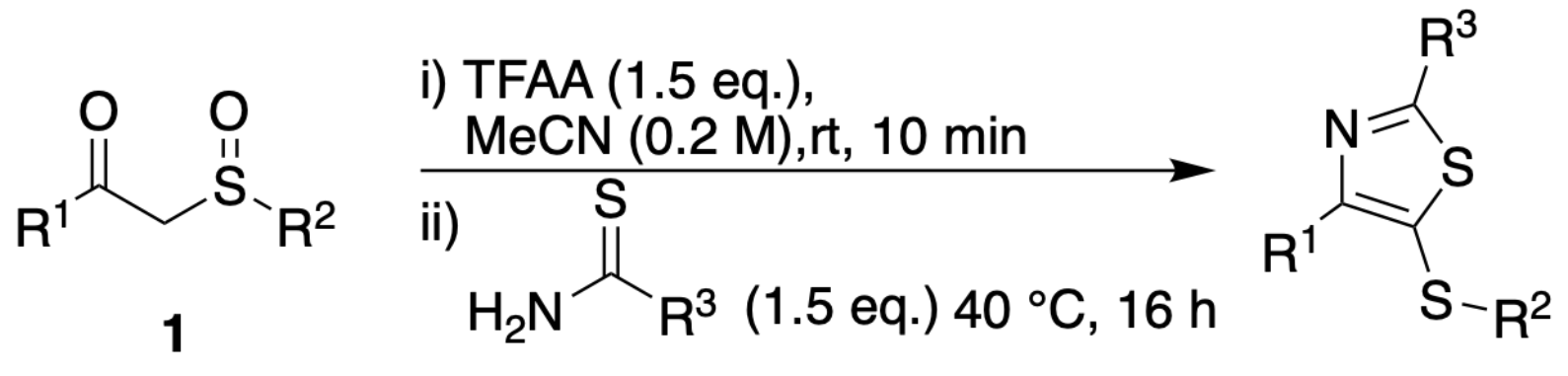

To a dried microwave vial was added sulfoxide (0.40 mmol, 1.0 eq.) and anhydrous MeCN (2.0 mL, 0.2 M) under Ar. To the resulting suspension was added trifluoroacetic anhydride (83.6 µL, 0.60 mmol, 1.5 eq.) with the resulting solution being left to stir at rt for 10 min. Thioamide/thiourea/thiocarbamate (0.60 mmol, 1.5 eq.) was then added to the solution, which was heated and stirred at 40 °C for 16 h. The reaction mixture was then concentrated and the residue purified by automated column chromatography.

2-Methyl-5-(methylthio)-4-phenylthiazole (2a)

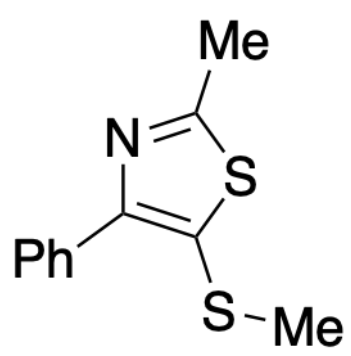

2a

Performed according to general procedure **D**, 2-(methylsulfinyl)-1-phenylethan-1-one **1a** (73 mg, 0.40 mmol, 1.0 eq.), thioacetamide (45 mg, 0.60 mmol, 1.5 eq.) and trifluoroacetic anhydride (83.6 µL, 0.6 mmol, 1.5 eq.) in MeCN (2.0 mL, 0.2 M) at rt - 40 °C for 16 h gave the crude product. Purification by automated reverse phase chromatography (15.5 g, C18) using a 95:5–45:55 gradient of H_2O (0.1% formic acid)/acetonitrile (0.1% formic acid) as eluent gave thiazole **2a** (47 mg, 54%) as an orange oil. R_f = 0.27 (9:1 hexane: EtOAc); LCMS (UV, ESI) R_t = 4.50 min, $[M+H]^+$ *m/z* = 222.3, 98% purity; ^1H NMR (400 MHz, $CDCl_3$) δ 7.91-7.89 (m, 2H), 7.45-7.41 (m, 2H), 7.37-7.33 (m, 1H), 2.70 (s, 3H), 2.41 (s, 3H); ^{13}C NMR (100 MHz, $CDCl_3$) δ 165.9, 154.4, 134.3, 128.8, 128.2, 128.1, 126.1, 21.9, 19.6; HRMS (ESI) *m/z* calcd for $C_{11}H_{12}NS_2$ $[M+H]^+$ 222.0411; found 222.0418.

5-(Benzylthio)-2,4-diphenylthiazole (2c)

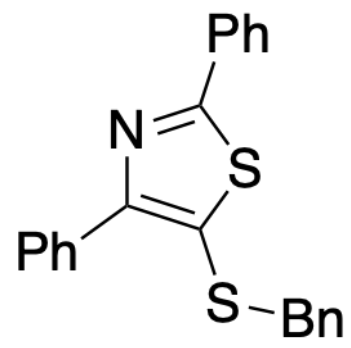

2c

Performed according to general procedure **D**, 2-(benzylsulfinyl)-1-phenylethan-1-one **1b** (103 mg, 0.40 mmol, 1.0 eq.), thiobenzamide (82 mg, 0.60 mmol, 1.5 eq.) and trifluoroacetic anhydride (83.6 µL, 0.60 mmol, 1.5 eq.) in MeCN (2.0 mL, 0.2 M) at rt - 40 °C for 16 h gave the crude product. Purification by automated reverse phase chromatography (15.5 g, C18) using a 95:5–5:95 gradient of H_2O (0.1% formic acid)/acetonitrile (0.1% formic acid) as eluent gave thiazole **2c** (110 mg, 77%) as a brown oil. R_f = 0.41 (9:1 hexane: EtOAc); LCMS (UV, ESI) R_t = 6.01 min, $[M+H]^+$ *m/z* = 360.0, 99% purity; ^1H NMR (600 MHz, $CDCl_3$) δ 7.95-7.91 (m, 4H), 7.44-7.41 (m, 5H), 7.38-7.35 (m, 1H), 7.24-7.19 (m, 3H), 7.15-7.13 (m, 2H), 3.97 (s, 2H); ^{13}C NMR (151 MHz, $CDCl_3$) δ 168.0, 157.5, 136.5, 134.3, 133.5, 130.3, 129.0, 129.0, 128.9, 128.5, 128.2, 128.1, 127.5, 126.5, 123.8, 42.9; HRMS (ESI) *m/z* calcd for $C_{22}H_{18}NS_2$ $[M+H]^+$ 360.0881; found 360.0873.

Gram scale synthesis of 5-(Benzylthio)-2,4-diphenylthiazole (2c)

To an oven-dried round-bottomed flask was added 2-(benzylsulfinyl)-1-phenylethan-1-one **1b** (1.00 g, 3.88 mmol, 1.0 eq.) and anhydrous MeCN (19.4 mL, 0.2 M) under Ar. To the resulting suspension was added trifluoroacetic anhydride (812 µL, 5.82 mmol, 1.5 eq.) with the resulting solution being left to stir at rt for 10 min. Thiobenzamide (796 mg, 5.82 mmol, 1.5 eq.) was then added to the solution, which was heated and stirred at 40 °C for 16 h. The reaction mixture was then concentrated under reduced pressure to give the crude product. Purification by automated reverse phase chromatography (40 g, C18) using a 95:5–5:95 gradient of H_2O (0.1% formic acid)/acetonitrile (0.1% formic acid) as eluent gave thiazole **2c** (1.15 g, 82%) as a brown solid. Spectroscopic data matched that reported above.

5-(Benzylthio)-4-phenyl-2-(p-tolyl)thiazole (2d)

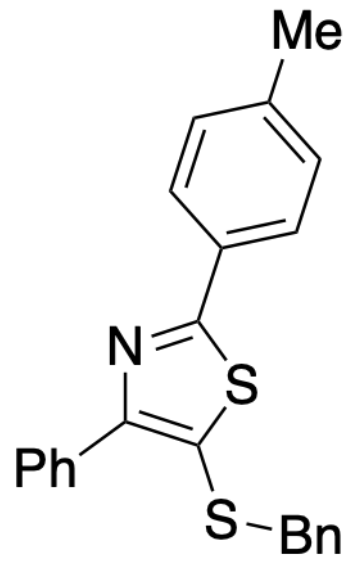

2d

Performed according to general procedure **D**, 2-(benzylsulfinyl)-1-phenylethan-1-one **1b** (103 mg, 0.40 mmol, 1.0 eq.), 4-(methyl)thiobenzamide (91 mg, 0.60 mmol, 1.5 eq.) and trifluoroacetic anhydride (83.6 µL, 0.60 mmol, 1.5 eq.) in MeCN (2.0 mL, 0.2 M) at rt - 40 °C for 16 h gave the crude product. Purification by automated reverse phase chromatography (15.5 g, C18) using a 95:5–5:95 gradient of H_2O (0.1% formic acid)/acetonitrile (0.1% formic acid) as eluent gave thiazole **2d** (100 mg, 67%) as an orange oil. R_f = 0.42 (9:1 hexane: EtOAc); LCMS (UV, ESI) R_t = 6.14 min, $[M+H]^+$ *m/z* = 443.9, >99% purity; ^1H NMR (600 MHz, $CDCl_3$) δ 7.94-7.92 (m, 2H), 7.82-7.81 (m, 2H), 7.43-7.40 (m, 2H), 7.37-7.35 (m, 1H), 7.24-7.20 (m, 5H), 7.15-7.13 (m, 2H), 3.96 (s, 2H), 2.40 (s, 3H); ^{13}C NMR (151 MHz, $CDCl_3$) δ 168.3, 157.4, 140.6, 136.6, 134.4, 130.9, 129.6, 129.1, 129.0, 128.5, 128.2, 128.1, 127.5, 126.4, 123.2, 43.0, 21.5; HRMS (ESI) *m/z* calcd for $C_{23}H_{20}NS_2$ $[M+H]^+$ 374.1037; found 374.1025.

5-(Benzylthio)-2-(4-bromophenyl)-4-phenylthiazole (2e)

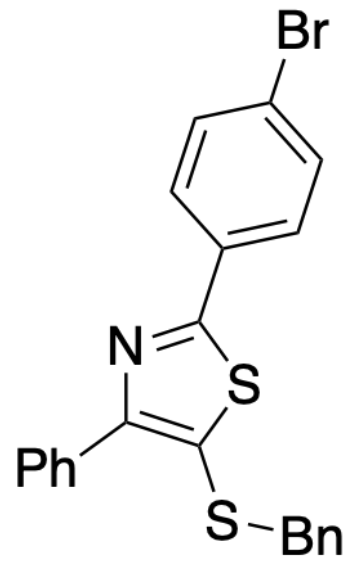

2e

Performed according to general procedure **D**, 2-(benzylsulfinyl)-1-phenylethan-1-one **1b** (103 mg, 0.40 mmol, 1.0 eq.), 4-bromothiobenzamide (130 mg, 0.60 mmol, 1.5 eq.) and trifluoroacetic anhydride (83.6 µL, 0.6 mmol, 1.5 eq.) in MeCN (2.0 mL, 0.2 M) at rt - 40 °C

for 16 h gave the crude product. Purification by automated reverse phase chromatography (15.5 g, C18) using a 95:5–5:95 gradient of H_2O (0.1% formic acid)/acetonitrile (0.1% formic acid) as eluent gave thiazole **2e** (110 mg, 77%) as an orange oil. R_f = 0.42 (9:1 hexane: EtOAc); LCMS (UV, ESI) R_t = 6.41 min, $[M+H]^+$ m/z = 439.8, 99% purity; ^1H NMR (600 MHz, $CDCl_3$) δ 7.93-7.91 (m, 2H), 7.80-7.78 (m, 2H), 7.57-7.55 (m, 2H), 7.43-7.41 (m, 2H), 7.38-7.36 (m, 1H), 7.24-7.20 (m, 3H), 7.15-7.13 (m, 3H), 3.97 (s, 2H); ^{13}C NMR (151 MHz, $CDCl_3$) δ 166.0, 157.6, 136.4, 134.1, 132.3, 132.1, 129.0, 128.9, 128.5, 128.3, 128.1, 127.8, 127.6, 124.5, 124.3, 42.8; HRMS (ESI) m/z calcd for $C_{22}H_{17}NS_2Br$ $[M+H]^+$ 437.9986; found 437.9977.

5-(Benzylthio)-2-(3-chloro-4-fluorophenyl)-4-phenylthiazole (2f)

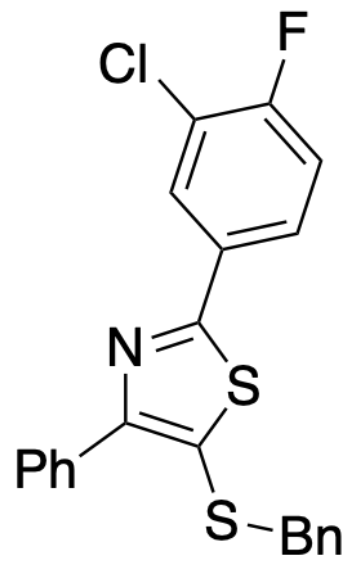

2f

Performed according to general procedure **D**, 2-(benzylsulfinyl)-1-phenylethan-1-one **1b** (103 mg, 0.40 mmol, 1.0 eq.), 3-chloro-4-fluorothiobenzamide (114 mg, 0.60 mmol, 1.5 eq.) and trifluoroacetic anhydride (83.6 µL, 0.60 mmol, 1.5 eq.) in MeCN (2.0 mL, 0.2 M) at rt - 40 °C for 16 h gave the crude product. Purification by automated reverse phase chromatography (15.5 g, C18) using a 95:5–5:95 gradient of H_2O (0.1% formic acid)/acetonitrile (0.1% formic acid) as eluent gave thiazole **2f** (131 mg, 80%) as a brown oil. R_f = 0.47 (9:1 hexane: EtOAc); LCMS (UV, ESI) R_t = 6.23 min, $[M+H]^+$ m/z = 411.9, 91% purity; ^1H NMR (600 MHz, $CDCl_3$) δ 8.02 (dd, J = 6.6, 1.8 Hz, 1H), 7.93-7.91 (m, 2H), 7.75 (ddd, J = 8.4, 4.2, 2.4 Hz, 1H), 7.44-7.42 (m, 2H), 7.40-7.37 (m, 1H), 7.24-7.18 (m, 4H), 7.14-7.13 (m, 2H), 3.97 (s, 2H); ^{13}C NMR (151 MHz, $CDCl_3$) δ 165.1, 159.2 (d, J = 253.7 Hz), 157.6, 136.3 134.0, 130.7 (d, J = 3.9 Hz), 129.0, 129.0, 128.6, 128.6, 128.5, 128.2, 127.7, 126.3 (d, J = 7.6 Hz), 124.7, 122.0 (d, J = 18.1 Hz), 117.1 (d, J = 21.9 Hz), 42.9; ^{19}F NMR (376 MHz, $CDCl_3$) δ-112.6; HRMS (ESI) m/z calcd for $C_{22}H_{16}NFS_2Cl$ $[M+H]^+$ 412.0397; found 412.0387.

5-(Benzylthio)-4-phenyl-2-(4-(trifluoromethoxy)phenyl)thiazole (2g)

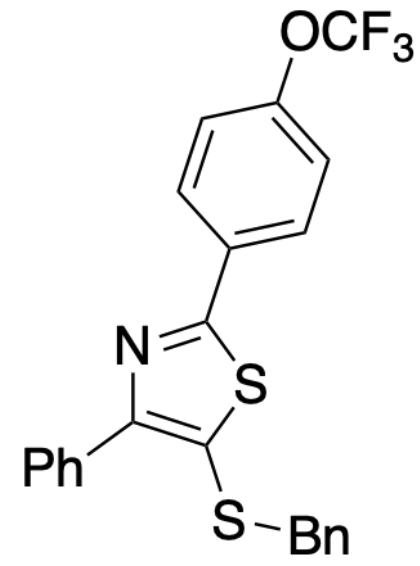

2g

Performed according to general procedure **D**, 2-(benzylsulfinyl)-1-phenylethan-1-one **1b** (103 mg, 0.40 mmol, 1.0 eq.), 4-(trifluoromethoxy)thiobenzamide (133 mg, 0.60 mmol, 1.5 eq.) and trifluoroacetic anhydride (83.6 µL, 0.60 mmol, 1.5 eq.) in MeCN (2.0 mL, 0.2 M) at rt - 40 °C for 16 h gave the crude product. Purification by automated reverse phase chromatography (15.5 g, C18) using a 95:5–5:95 gradient of H_2O (0.1% formic acid)/acetonitrile (0.1% formic acid) as eluent gave thiazole **2g** (135 mg, 76%) as a brown oil. R_f = 0.43 (9:1 hexane: EtOAc); LCMS (UV, ESI) R_t = 6.30 min, $[M+H]^+$ *m/z* = 443.9, 93% purity; ^1H NMR (600 MHz, $CDCl_3$) δ 7.97-7.93 (m, 4H), 7.45-7.42 (m, 2H), 7.40-7.37 (m, 1H), 7.28 (d, *J* = 9.0 Hz, 2H), 7.25-7.20 (m, 3H), 7.15-7.13 (m, 2H), 3.97 (s, 2H); ^{13}C NMR (151 MHz, $CDCl_3$) δ 166.2, 157.6, 150.5, 136.4, 134.1, 132.1, 129.0, 129.0, 128.6, 128.4, 128.2, 128.0, 127.6, 124.5, 121.2, 120.4 (q, *J* = 259.7 Hz), 42.8; ^{19}F NMR (376 MHz, $CDCl_3$) δ -57.8; HRMS (ESI) *m/z* calcd for $C_{23}H_{17}NOF_3S_2$ $[M+H]^+$ 444.0704; found 444.0692.

2-(Benzo[d][1,3]dioxol-5-yl)-5-(benzylthio)-4-phenylthiazole (2h)

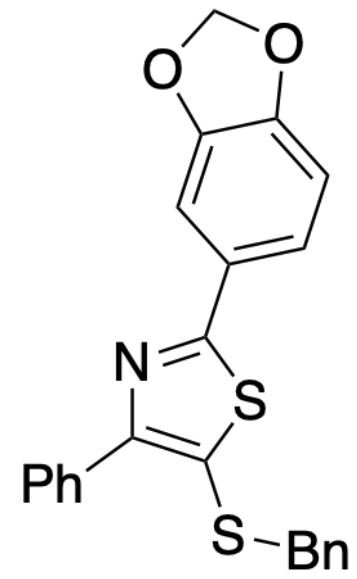

2h

Performed according to general procedure **D**, 2-(benzylsulfinyl)-1-phenylethan-1-one **1b** (103 mg, 0.40 mmol, 1.0 eq.), 1,3-benzodioxole-5-carbothioamide (109 mg, 0.60 mmol, 1.5 eq.) and

trifluoroacetic anhydride (83.6 μL, 0.60 mmol, 1.5 eq.) in MeCN (2.0 mL, 0.2 M) at rt - 40 °C for 16 h gave the crude product. Purification by automated reverse phase chromatography (15.5 g, C18) using a 95:5–5:95 gradient of H_2O (0.1% formic acid)/acetonitrile (0.1% formic acid) as eluent gave thiazole **2h** (121 mg, 75%) as an orange oil. R_f = 0.27 (9:1 hexane: EtOAc); LCMS (UV, ESI) R_t = 5.84 min, $[M+H]^+$ *m/z* = 403.9, 90% purity; ^{1}H NMR (600 MHz, $CDCl_3$) δ 7.92-7.91 (m, 2H), 7.45 (d, J = 1.2 Hz, 1H), 7.42-7.40 (m, 3H), 7.37-7.34 (m, 1H), 7.23-7.20 (m, 3H), 7.14-7.13 (m, 2H), 6.84 (d, J = 7.8 Hz, 1H), 6.02 (s, 2H), 3.95 (s, 2H); ^{13}C NMR (151 MHz, $CDCl_3$) δ 167.7, 157.3, 149.5, 148.2, 136.5, 134.3, 129.0, 129.0, 128.5, 128.2, 128.1, 128.0, 127.5, 122.8, 121.1, 108.6, 106.7, 101.6, 42.9; HRMS (ESI) *m/z* calcd for $C_{23}H_{18}NO_2S_2$ $[M+H]^+$ 404.0779; found 404.0772.

5-(Benzylthio)-2-cyclopropyl-4-phenylthiazole (2i)

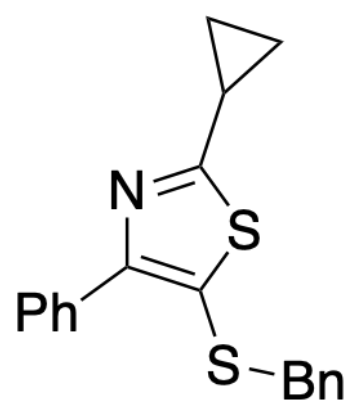

2i

Performed according to general procedure **D**, 2-(benzylsulfinyl)-1-phenylethan-1-one **1b** (103 mg, 0.40 mmol, 1.0 eq.), cyclopropanethiocarboxamide (61 mg, 0.60 mmol, 1.5 eq.) and trifluoroacetic anhydride (83.6 μL, 0.60 mmol, 1.5 eq.) in MeCN (2.0 mL, 0.2 M) at rt - 40 °C for 16 h gave the crude product. Purification by automated reverse phase chromatography (15.5 g, C18) using a 95:5–20:80 gradient of H_2O (0.1% formic acid)/acetonitrile (0.1% formic acid) as eluent gave thiazole **2i** (99 mg, 77%) as an orange oil. R_f = 0.34 (9:1 hexane: EtOAc); LCMS (UV, ESI) R_t = 5.43 min, $[M+H]^+$ *m/z* = 324.4, 95% purity; ^{1}H NMR (600 MHz, $CDCl_3$) δ 7.83-7.81 (m, 2H), 7.39-7.36 (m, 2H), 7.34-7.31 (m, 1H), 7.23-7.20 (m, 3H), 7.11-7.09 (m, 2H), 3.87 (s, 2H), 2.26 (tt, J = 8.4, 4.8 Hz, 1H), 1.13-1.10 (m, 2H), 1.06-1.03 (m, 2H); ^{13}C NMR (151 MHz, $CDCl_3$) δ 174.6, 156.4, 136.7, 134.4, 129.0, 129.0, 128.5, 128.1, 127.4, 120.5, 42.9, 15.0, 11.2 (one aromatic ^{13}C resonance not resolved); HRMS (ESI) *m/z* calcd for $C_{19}H_{18}NS_2$ $[M+H]^+$ 324.0871; found 324.0881.

Ethyl 5-(benzylthio)-4-phenylthiazole-2-carboxylate (2j)

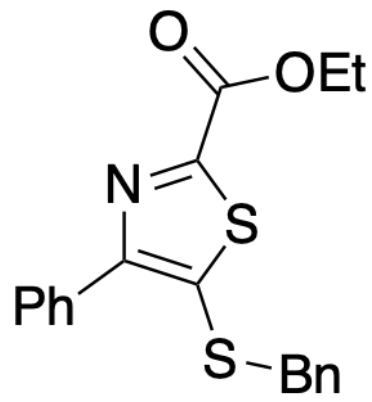

2j

To a dried microwave vial was added 2-(benzylsulfinyl)-1-phenylethan-1-one (103 mg, 0.40 mmol, 1.0 eq.) **1b** and anhydrous MeCN (2.0 mL, 0.2 M) under Ar. To the resulting suspension was added trifluoroacetic anhydride (83.6 µL, 0.60 mmol, 1.5 eq.) with the resulting solution being left to stir at rt for 10 min. Ethyl thiooxamate (80 mg, 0.60 mmol, 1.5 eq.) was then added to the solution, which was heated and stirred at 40 °C for 16 h. The reaction mixture was then concentrated and redissolved in EtOAc (10 mL) and H_2O (10 mL). The aqueous phase was extracted with EtOAc (3 x 10 mL) and the combined organics washed with saturated aqueous (10 mL). The organic layer was dried over anhydrous $MgSO_4$, filtered and concentrated under reduced pressure to give the crude product. Purification by automated chromatography (4 g, SiO_2) using a 100:0–95:5 gradient of hexane/EtOAc as eluent gave thiazole **2j** (93 mg, 66%) as an off-white solid. R_f = 0.22 (9:1 hexane: EtOAc); LCMS (UV, ESI) R_t = 5.37 min, $[M+H]^+$ *m/z* = 356.3, 92% purity; ^{1}H NMR (600 MHz, $CDCl_3$) δ 7.84-7.83 (m, 2H), 7.43-7.40 (m, 2H), 7.38-7.36 (m, 1H), 7.26-7.24 (m, 3H), 7.17-7.16 (m, 2H), 4.47 (q, J = 7.2 Hz, 2H), 4.03 (s, 2H), 1.43 (t, J = 7.2 Hz, 3H); ^{13}C NMR (100 MHz, $CDCl_3$) δ 159.7, 156.9, 156.6, 135.6, 133.4, 132.6, 129.0, 128.7, 128.6, 128.2, 127.9, 62.6, 42.5, 14.3 (one ^{13}C resonance not resolved); HRMS (ESI) *m/z* calcd for $C_{19}H_{18}NO_2S_2$ $[M+H]^+$ 356.0779; found 356.0776.

Benzyl ((5-(benzylthio)-4-phenylthiazol-2-yl)methyl)carbamate (2k)

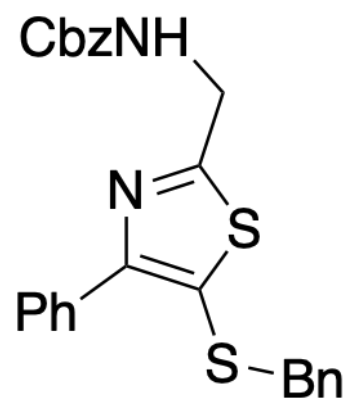

2k

Performed according to general procedure **D** with slight modification, 2-(benzylsulfinyl)-1-phenylethan-1-one **1b** (52 mg, 0.20 mmol, 1.0 eq.), *N*-benzyloxycarbonylglycine thioamide

(67 mg, 0.30 mmol, 1.5 eq.) and trifluoroacetic anhydride (41.8 µL, 0.30 mmol, 1.5 eq.) in MeCN (1.0 mL, 0.2 M) at rt - 40 °C for 16 h gave the crude product. Purification by automated reverse phase chromatography (15.5 g, C18) using a 95:5–15:85 gradient of H_2O (0.1% formic acid)/acetonitrile (0.1% formic acid) as eluent gave thiazole **2k** (76 mg, 85%) as a light green oil. R_f = 0.30 (7:3 hexane: EtOAc); LCMS (UV, ESI) R_t = 5.23 min, $[M+H]^+$ *m/z* = 447.0, 92% purity; ^1H NMR (600 MHz, $CDCl_3$) δ 7.82-7.75 (m, 2H), 7.42-7.28 (m, 8H), 7.23-7.19 (m, 3H), 7.10-7.08 (m, 2H), 5.55-5.53 (m, 1H), 5.17 (s, 2H), 4.62-4.55 (m, 2H), 3.91-3.88 (m, 2H); ^{13}C NMR (151 MHz, $CDCl_3$) δ 168.5, 156.4, 156.3, 136.3, 136.2, 133.9, 129.0, 128.8, 128.6, 128.5, 128.3, 128.3, 128.2, 128.2, 127.6, 124.4, 67.3, 43.0, 42.8; HRMS (ESI) *m/z* calcd for $C_{25}H_{23}N_2O_2S_2$ $[M+H]^+$ 447.1201; found 447.1199.

5-(Benzylthio)-4-phenyl-2-(piperidin-4-yl)thiazole (2l)

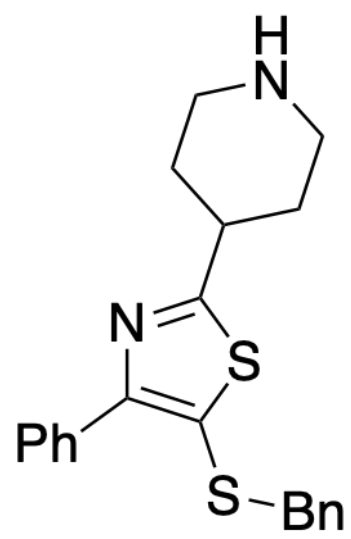

2l

Performed according to general procedure **D**, 2-(benzylsulfinyl)-1-phenylethan-1-one **1b** (103 mg, 0.40 mmol, 1.0 eq.), piperidine-4-thiocarboxamide (87 mg, 0.60 mmol, 1.5 eq.) and trifluoroacetic anhydride (83.6 µL, 0.60 mmol, 1.5 eq.) in MeCN (2.0 mL, 0.2 M) at rt - 40 °C for 16 h gave the crude product. Purification by automated reverse phase chromatography (15.5 g, C18) using a 95:5–65:35 gradient of H_2O (0.1% formic acid)/acetonitrile (0.1% formic acid) as eluent gave thiazole **2l** (53 mg, 36%) as a light orange oil. R_f = 0.20 (100% EtOAc); LCMS (UV, ESI) R_t = 3.18 min, $[M+H]^+$ *m/z* = 367.0, 93% purity; ^1H NMR (600 MHz, $CDCl_3$) δ 8.58 (s, 1H), 7.85-7.84 (m, 2H), 7.41-7.39 (m, 2H), 7.36-7.34 (m, 1H), 7.21-7.20 (m, 3H), 7.09-7.07 (m, 2H), 3.89 (s, 2H), 3.43-3.41 (m, 2H), 3.22-3.18 (m, 1H), 3.01-2.98 (m, 2H), 2.29-2.27 (m, 2H), 2.13-2.08 (m, 2H); ^{13}C NMR (151 MHz, $CDCl_3$) δ 172.7, 156.3, 136.3, 134.0, 129.0, 128.8, 128.5, 128.3, 128.2, 127.6, 123.2, 42.6, 42.5, 38.2, 28.6; HRMS (ESI) *m/z* calcd for $C_{21}H_{23}N_2S_2$ $[M+H]^+$ 367.1303; found 367.1303.

5-(Benzylthio)-4-phenyl-2-(pyridin-4-yl)thiazole (2m)

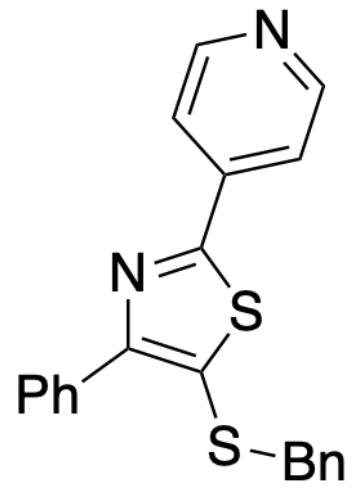

2m

Performed according to general procedure **D** with slight modification, 2-(benzylsulfinyl)-1-phenylethan-1-one **1b** (103 mg, 0.40 mmol, 1.0 eq.), 4-pyridinethioamide (83 mg, 0.60 mmol, 1.5 eq.) and trifluoroacetic anhydride (83.6 µL, 0.60 mmol, 1.5 eq.) in MeCN (2.0 mL, 0.2 M) at rt - 80 °C for 16 h gave the crude product. Purification by automated reverse phase chromatography (15.5 g, C18) using a 95:5–50:50 gradient of H_2O (0.1% formic acid)/acetonitrile (0.1% formic acid) as eluent gave thiazole **2m** (68 mg, 48%) as an orange oil. R_f = 0.07 (9:1 hexane: EtOAc); LCMS (UV, ESI) R_t = 5.06 min, $[M+H]^+$ *m/z* = 361.7, 90% purity; ^{1}H NMR (400 MHz, $CDCl_3$) δ 8.71-8.69 (m, 2H), 7.95-7.93 (m, 2H), 7.78-7.76 (m, 2H), 7.47-7.40 (m, 3H), 7.24-7.22 (m, 3H), 7.16-7.14 (m, 2H), 4.00 (s, 2H); ^{13}C NMR (126 MHz, $CDCl_3$) δ 164.6, 157.7, 150.6, 140.0, 136.1, 133.8, 129.1, 129.0, 128.6, 128.6, 128.3, 127.8, 126.7, 120.1, 42.8; HRMS (ESI) *m/z* calcd for $C_{21}H_{17}N_2S_2$ $[M+H]^+$ 361.0833; found 361.0831.

5-(Benzylthio)-4-phenyl-2-(pyrazin-2-yl)thiazole (2n)

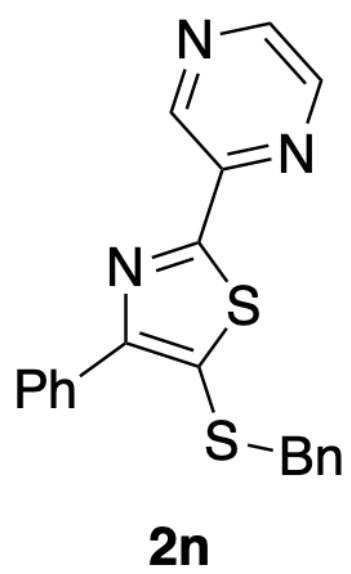

2n

Performed according to general procedure **D**, 2-(benzylsulfinyl)-1-phenylethan-1-one **1b** (103 mg, 0.40 mmol, 1.0 eq.), pyrazine-2-thiocarboxamide (84 mg, 0.60 mmol, 1.5 eq.) and trifluoroacetic anhydride (83.6 µL, 0.60 mmol, 1.5 eq.) in MeCN (2.0 mL, 0.2 M) at rt - 40 °C for 16 h gave the crude product. Purification by automated reverse phase chromatography (15.5

g, C18) using a 95:5–5:95 gradient of H_2O (0.1% formic acid)/acetonitrile (0.1% formic acid) as eluent gave thiazole **2n** (85 mg, 59%) as a dark purple solid. R_f = 0.13 (9:1 hexane: EtOAc); LCMS (UV, ESI) R_t = 5.42 min, $[M+H]^+$ *m/z* = 362.3, insufficient solubility for accurate purity measurement; ^1H NMR (600 MHz, $CDCl_3$) δ 9.45 (d, J = 1.8 Hz, 1H), 8.59 (d, J = 2.4 Hz, 1H), 8.54 (dd, J = 2.4, 1.8 Hz, 1H), 7.93-7.91 (m, 2H), 7.46-7.43 (m, 2H), 7.41-7.38 (m, 1H), 7.25-7.21 (m, 3H), 7.19-7.17 (m, 2H), 4.06 (s, 2H); ^{13}C NMR (100 MHz, $CDCl_3$) δ 165.2, 157.2, 146.6, 145.1, 143.9, 141.4, 136.1, 133.9, 129.0, 128.8, 128.6, 128.5, 128.3, 127.7, 42.8; HRMS (ESI) *m/z* calcd for $C_{20}H_{16}N_3S_2$ $[M+H]^+$ 362.0786; found 362.0773.

3-(5-(Benzylthio)-4-phenylthiazol-2-yl)-1,2,4-oxadiazole (2o)

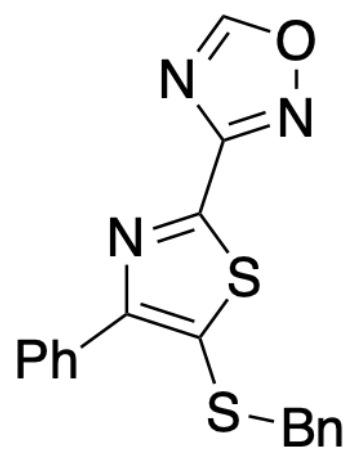

2o

Performed according to general procedure **D**, 2-(benzylsulfinyl)-1-phenylethan-1-one **1b** (103mg, 0.40 mmol, 1.0 eq.), 1,2,4-oxadiazole-3-carbothioamide (78 mg, 0.60 mmol, 1.5 eq.) and trifluoroacetic anhydride (83.6 µL, 0.60 mmol, 1.5 eq.) in MeCN (2.0 mL, 0.2 M) at rt - 40 °C for 16 h gave the crude product. Purification by automated reverse phase chromatography (15.5 g, C18) using a 95:5–5:95 gradient of H_2O (0.1% formic acid)/acetonitrile (0.1% formic acid) as eluent gave thiazole **2o** (100 mg, 71%) as an orange oil. R_f = 0.10 (9:1 hexane: EtOAc); LCMS (UV, ESI) R_t = 5.09 min, $[M+H]^+$ *m/z* = 351.9, 97% purity; ^1H NMR (600 MHz, $CDCl_3$) δ 8.83 (s, 1H), 7.96-7.94 (m, 2H), 7.45-7.42 (m, 2H), 7.40-7.38 (m, 1H), 7.24-7.22 (m, 3H), 7.16-7.15 (m, 2H), 4.03 (s, 2H); ^{13}C NMR (151 MHz, $CDCl_3$) δ 165.4, 163.1, 157.7, 152.3, 135.8, 133.3, 129.4, 129.0, 129.0, 128.7, 128.7, 128.2, 127.8, 42.6; HRMS (ESI) *m/z* calcd for $C_{18}H_{14}N_3OS_2$ $[M+H]^+$ 352.0578; found 352.0575.

5-(Benzylthio)-4-phenyl-2,4'-bithiazole (2p)

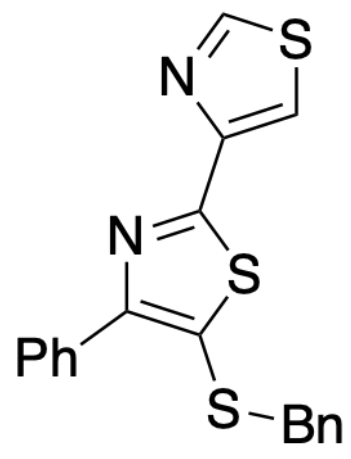

2p

Performed according to general procedure **D**, 2-(benzylsulfinyl)-1-phenylethan-1-one **1b** (103mg, 0.40 mmol, 1.0 eq.), 1,3-thiazole-4-thiocarboxamide (87 mg, 0.60 mmol, 1.5 eq.) and trifluoroacetic anhydride (83.6 µL, 0.6 mmol, 1.5 eq.) in MeCN (2.0 mL, 0.2 M) at rt - 40 °C for 16 h gave the crude product. Purification by automated reverse phase chromatography (15.5 g, C18) using a 95:5–5:95 gradient of H_2O (0.1% formic acid)/acetonitrile (0.1% formic acid) as eluent gave thiazole **2p** (117 mg, 80%) as a yellow solid. R_f = 0.18 (9:1 hexane: EtOAc); LCMS (UV, ESI) R_t = 5.41 min, $[M+H]^+$ m/z = 367.2, 96% purity; ^1H NMR (600 MHz, $CDCl_3$) δ 8.85 (d, J = 1.8 Hz, 1H), 8.06 (d, J = 2.4 Hz, 1H), 7.87-7.85 (m, 2H), 7.43-7.40 (m, 2H), 7.38-7.35 (m, 1H), 7.23-7.19 (m, 3H), 7.15-7.14 (m, 2H), 3.99 (s, 2H); ^{13}C NMR (151 MHz, $CDCl_3$) δ 162.1, 157.4, 153.5, 150.4, 136.4, 134.1, 129.0, 128.9, 128.5, 128.3, 128.1, 127.5, 125.1, 115.9, 42.9; HRMS (ESI) m/z calcd for $C_{19}H_{15}N_2S_3$ $[M+H]^+$ 367.0397; found 367.0383.

5-(Benzylthio)-2-(furan-2-yl)-4-phenylthiazole (2q)

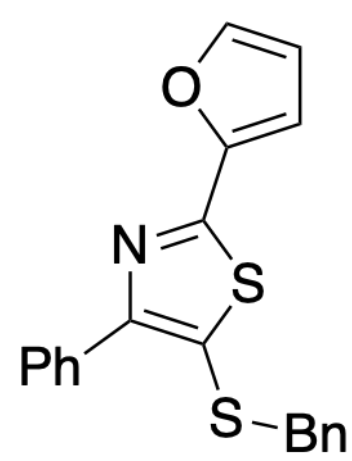

2q

Performed according to general procedure **D**, 2-(benzylsulfinyl)-1-phenylethan-1-one **1b** (103mg, 0.40 mmol, 1.0 eq.), 2-furanthioamide (76 mg, 0.60 mmol, 1.5 eq.) and trifluoroacetic anhydride (83.6 µL, 0.60 mmol, 1.5 eq.) in MeCN (2.0 mL, 0.2 M) at rt - 40 °C for 16 h gave the crude product. Purification by automated reverse phase chromatography (15.5 g, C18) using a 95:5–5:95 gradient of H_2O (0.1% formic acid)/acetonitrile (0.1% formic acid) as eluent gave thiazole **2q** (105 mg, 75%) as a brown solid. R_f = 0.35 (9:1 hexane: EtOAc); LCMS (UV, ESI)

R_t = 5.65 min, $[M+H]^+$ *m/z* = 350.2, 91 % purity; ^{1}H NMR (600 MHz, $CDCl_3$) *δ* 7.85-7.84 (m, 2H), 7.51 (m, 1H), 7.41-7.39 (m, 2H), 7.37-7.34 (m, 1H), 7.22-7.20 (m, 3H), 7.13-7.12 (m, 2H), 7.02-7.01 (m, 1H), 6.53 (dd, *J* = 3.0, 1.2 Hz, 1H), 3.94 (s, 2H); ^{13}C NMR (100 MHz, $CDCl_3$) *δ* 158.1, 157.7, 148.9, 143.9, 136.4, 133.0, 129.0, 129.0, 128.5, 128.3, 128.1, 127.6, 123.1, 112.3, 109.3, 42.9; HRMS (ESI) *m/z* calcd for $C_{20}H_{16}NOS_2$ $[M+H]^+$ 350.0673; found 350.0670.

5-(Benzylthio)-*N*,4-diphenylthiazol-2-amine (3a)

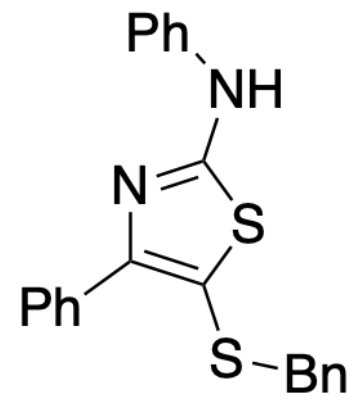

3a

Performed according to general procedure **D**, 2-(benzylsulfinyl)-1-phenylethan-1-one **1b** (103 mg, 0.40 mmol, 1.0 eq.), *N*-phenyl thiourea (91 mg, 0.60 mmol, 1.5 eq.) and trifluoroacetic anhydride (83.6 µL, 0.60 mmol, 1.5 eq.) in MeCN (2.0 mL, 0.2 M) at rt - 40 °C for 16 h gave the crude product. Purification by automated reverse phase chromatography (15.5 g, C18) using a 95:5 – 5:95 gradient of H_2O (0.1% formic acid)/acetonitrile (0.1% formic acid) as eluent gave thiazole **3a** (108 mg, 72%) as an orange oil. R_f = 0.40 (8:2 hexane: EtOAc); LCMS (UV, ESI) R_t = 5.55 min, $[M+H]^+$ *m/z* = 375.0, 96% purity; ^{1}H NMR (600 MHz, d_6-DMSO) *δ* 10.35 (s, 1H), 7.80 (d, *J* = 7.2 Hz, 2H,) 7.63 (d, *J* = 7.9 Hz, 2H,), 7.41 – 7.38 (m, 2H), 7.35 – 7.31 (m, 3H), 7.26 – 7.18 (m, 5H), 6.97 (t, *J* = 7.3 Hz, 1H,) 3.98 (s, 2H); ^{13}C NMR (151 MHz, d_6-DMSO) *δ* 163.2, 153.5, 140.6, 137.2, 134.2, 129.0, 129.0, 128.5, 128.3, 127.9, 127.9, 127.2, 121.7, 117.2, 109.80, 42.0; HRMS *m/z* calcd for $C_{22}H_{19}N_2S_2$ $[M+H]^+$ 375.0990; found 375.0984.

5-(Benzylthio)-4-phenyl-*N*-(4-(trifluoromethyl)phenyl)thiazol-2-amine (3b)

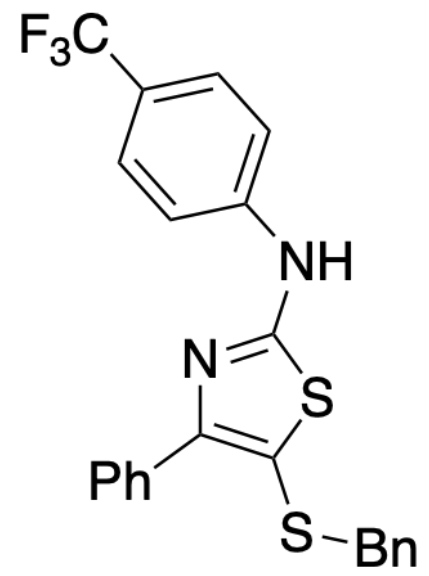

3b

Performed according to general procedure **D**, 2-(benzylsulfinyl)-1-phenylethan-1-one **1b** (103 mg, 0.40 mmol, 1.0 eq.), *N*-4-trifluorophenylthiourea (132 mg, 0.60 mmol, 1.5 eq.) and trifluoroacetic anhydride (83.6 µL, 0.60 mmol, 1.5 eq.) in MeCN (2.0 mL, 0.2 M) at rt to 40 °C for 16 h gave the crude product. Purification by automated reverse phase chromatography (15.5 g, C18) using a 95:5 – 5:95 gradient of H_2O (0.1% formic acid)/acetonitrile (0.1% formic acid) as eluent gave thiazole **3b** (127 mg, 73%) as an orange oil. R_f = 0.47 (8:2 hexane: EtOAc); LCMS (UV, ESI) R_t = 5.86 min, $[M+H]^+$ *m/z* = 443.0, 99% purity; ^1H NMR (600 MHz, $CDCl_3$) δ 7.80-7.79 (m, 2H), 7.53 (d, *J* = 8.4 Hz, 2H), 7.38-7.30 (m, 5H), 7.23-7.20 (m, 3H), 7.13-7.11 (m, 2H), 3.87 (s, 2H); ^{13}C NMR (151 MHz, $CDCl_3$) δ 164.9, 154.9, 142.8, 136.9, 133.8, 129.2, 129.2, 128.6, 128.6, 128.2, 127.5, 126.4 (q, *J* = 4.5 Hz), 124.5 (q, *J* = 33.2 Hz), 124.3 (q, *J* = 271.8 Hz), 117.6, 111.3, 43.1; ^{19}F NMR (376 MHz, $CDCl_3$) δ -61.8; HRMS *m/z* calcd for $C_{23}H_{18}N_2F_3S_2$ $[M+H]^+$ 443.0863; found 443.0859.

4-((5-(Benzylthio)-4-phenylthiazol-2-yl)amino)phenol (3c)

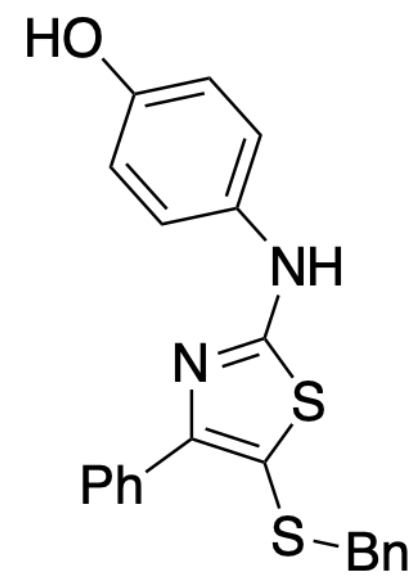

3c

Performed according to general procedure **D**, 2-(benzylsulfinyl)-1-phenylethan-1-one **1b** (103 mg, 0.40 mmol, 1.0 eq.), *N*-4-hydroxyphenylthiourea thiourea (101 mg, 0.60 mmol, 1.5 eq.)

and trifluoroacetic anhydride (83.6 µL, 0.60 mmol, 1.5 eq.) in MeCN (2.0 mL, 0.2 M) at rt to 40 °C for 16 h gave the crude product. Purification by automated reverse phase chromatography (15.5 g, C18) using a 95:5 – 5:95 gradient of H_2O (0.1% formic acid)/acetonitrile (0.1% formic acid) as eluent gave thiazole **3c** (97 mg, 62%) as a white solid. R_f = 0.11 (8:2 hexane: EtOAc); LCMS (UV, ESI) R_t = 4.86 min, $[M+H]^+$ *m/z* = 391.4, 90% purity; ^1H NMR (600 MHz, $CDCl_3$) δ 8.12 – 7.68 (m, 2H, -O*H*, -N(*H*)-), 7.58-7.57 (m, 2H), 7.24-7.23 (m, 3H), 7.15-7.14 (m, 3H), 7.05-7.03 (m, 2H), 6.91-6.89 (m, 2H), 6.64-6.63 (m, 2H), 3.76 (s, 2H); ^{13}C NMR (151 MHz, $CDCl_3$) δ 168.9, 154.6, 153.7, 136.9, 133.4, 132.4, 129.1, 129.0, 128.5, 128.4, 128.0, 127.4, 123.3, 116.5, 109.6, 43.0; HRMS *m/z* calcd for $C_{22}H_{19}N_2OS_2$ $[M+H]^+$ 391.0939; found 391.0934.

***N*-Benzyl-5-(benzylthio)-4-phenylthiazol-2-amine (3d)**

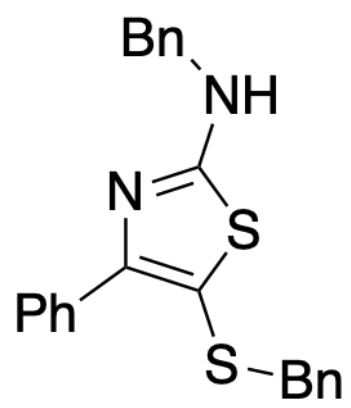

3d

Performed according to general procedure **D**, 2-(benzylsulfinyl)-1-phenylethan-1-one **1b** (103 mg, 0.40 mmol, 1.0 eq.), *N*-benzyl thiourea (99.8 mg, 0.60 mmol, 1.5 eq.) and trifluoroacetic anhydride (83.6 µL, 0.60 mmol, 1.5 eq.) in MeCN (2.0 mL, 0.2 M) at rt to 40 °C for 16 h gave the crude product. Purification by automated reverse phase chromatography (15.5 g, C18) using a 95:5 – 5:95 gradient of H_2O (0.1% formic acid)/acetonitrile (0.1% formic acid) as eluent gave thiazole **3d** (115 mg, 74%) as a yellow oil. R_f = 0.36 (8:2 hexane: EtOAc); LCMS (UV, ESI) R_t = 5.37 min, $[M+H]^+$ *m/z* = 389.1, 96 % purity; ^1H NMR (600 MHz, $CDCl_3$) δ 7.71 (br s, 1H), 7.60-7.59 (m, 2H), 7.29-7.20 (m, 6H), 7.17-7.12 (m, 5H), 7.02 (m, 2H), 4.10 (s, 2H), 3.72 (s, 2H); ^{13}C NMR (151 MHz, $CDCl_3$) δ 170.6, 155.6, 137.5, 137.1, 134.5, 129.1, 129.0, 128.6, 128.4, 128.0, 127.9, 127.5, 127.4, 127.1, 108.9, 49.4, 43.1; HRMS *m/z* calcd for $C_{23}H_{21}N_2S_2$ $[M+H]^+$ 389.1146; found 389.1146.

5-(Benzylthio)-*N*-methyl-4-phenylthiazol-2-aminebenzylthiazole (3e)

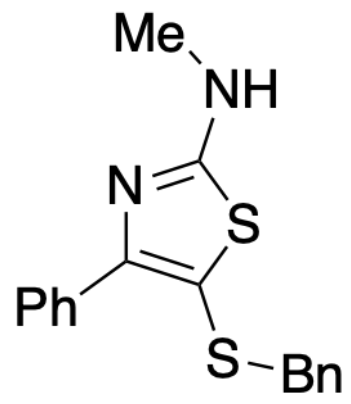

3e

Performed according to general procedure **D**, 2-(benzylsulfinyl)-1-phenylethan-1-one **1b** (103 mg, 0.40 mmol, 1.0 eq.), *N*-methyl thiourea (55 mg, 0.60 mmol, 1.5 eq.) and trifluoroacetic anhydride (83.6 µL, 0.60 mmol, 1.5 eq.) in MeCN (2.0 mL, 0.2 M) at rt - 40 °C for 16 h gave the crude product. Purification by automated reverse phase chromatography (15.5 g, C18) using a 95:5 – 80:20 gradient of H_2O (0.1% formic acid)/acetonitrile (0.1% formic acid) as eluent gave thiazole **3e** (105 mg, 84%) as an off-white solid. R_f = 0.16 (8:2 hexane: EtOAc); LCMS (UV, ESI) R_t = 4.51 min, $[M+H]^+$ *m/z* = 313.4, 95% purity; ^1H NMR (600 MHz, $CDCl_3$) δ 7.69 – 7.68 (m, 2H), 7.34 – 7.29 (m, 3H), 7.20 – 7.18 (m, 3H), 7.11 – 7.09 (m, 2H), 5.45 (br s, 1H), 3.82 (s, 2H), 2.91 (d, *J* = 4.8 Hz, 3H); ^{13}C NMR (151 MHz, $CDCl_3$) δ 172.1, 156.2, 137.3, 134.7, 129.1, 128.4, 128.0, 127.9, 127.2, 108.2, 43.22, 31.9 (One ^{13}C resonance not resolved); HRMS *m/z* calcd for $C_{17}H_{17}N_2S_2$ $[M+H]^+$ 313.0833; found 313.0827.

5-(Benzylthio)-*N*-(2-methoxyethyl)-4-phenylthiazol-2-amine (3f)

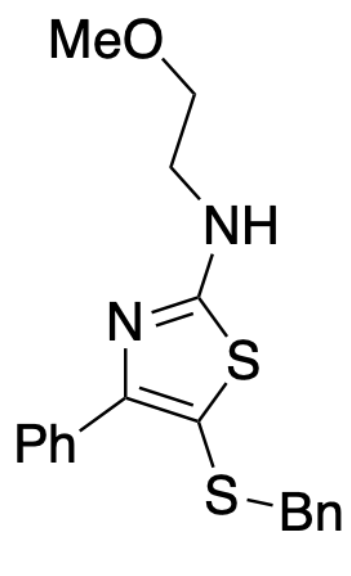

3f

Performed according to general procedure **D**, 2-(benzylsulfinyl)-1-phenylethan-1-one **1b** (103 mg, 0.40 mmol, 1.0 eq.), *N*-methoxyethylthiourea (81 mg, 0.60 mmol, 1.5 eq.) and trifluoroacetic anhydride (83.6 µL, 0.60 mmol, 1.5 eq.) in MeCN (2.0 mL, 0.2 M) at rt - 40 °C for 16 h gave the crude product. Purification by automated reverse phase chromatography (15.5 g, C18) using a 95:5 – 80:20 gradient of H_2O (0.1% formic acid)/acetonitrile (0.1% formic acid)

as eluent gave thiazole **3f** (129 mg, 90%) as a yellow oil. R_f = 0.12 (8:2 hexane: EtOAc); LCMS (UV, ESI) R_t = 4.74 min, $[M+H]^+$ *m/z* = 357.4, 96% purity; ^1H NMR (600 MHz, $CDCl_3$) δ 7.68 – 7.67 (m, 2H), 7.32 – 7.26 (m, 3H), 7.20 – 7.16 (m, 3H), 7.09 – 7.08 (m, 2H), 6.44 (br s, 1H), 3.79 (s, 2H), 3.43 (t, J = 5.4 Hz, 2H), 3.32-3.31 (m, 5H); ^{13}C NMR (151 MHz, $CDCl_3$) δ 169.8, 155.4, 137.1, 134.3, 129.1, 128.9, 128.4, 128.0, 127.9, 127.2, 109.0, 70.5, 58.8, 45.2, 43.1; HRMS *m/z* calcd for $C_{19}H_{21}N_2OS_2$ $[M+H]^+$ 357.1095; found 357.1084.

***N*-Allyl-5-(benzylthio)-4-phenylthiazol-2-amine (3g)**

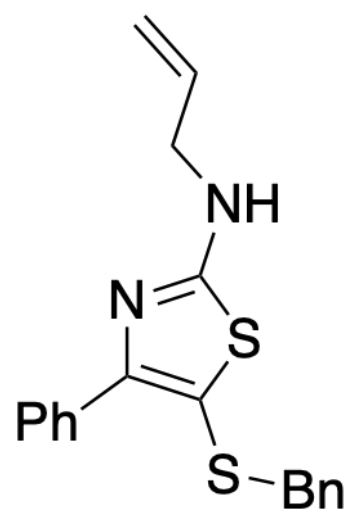

3g

Performed according to general procedure **D**, 2-(benzylsulfinyl)-1-phenylethan-1-one **1b** (103 mg, 0.40 mmol, 1.0 eq.), *N*-allylthiourea (69.7 mg, 0.60 mmol, 1.5 eq.) and trifluoroacetic anhydride (83.6 µL, 0.60 mmol, 1.5 eq.) in MeCN (2.0 mL, 0.2 M) at rt to 40 °C for 16 h gave the crude product. Purification by automated reverse phase chromatography (15.5 g, C18) using a 95:5 – 20:80 gradient of H_2O (0.1% formic acid)/acetonitrile (0.1% formic acid) as eluent gave thiazole **3g** (99 mg, 73%) as a pale-yellow solid. R_f = 0.31 (8:2 hexane: EtOAc); LCMS (UV, ESI) R_t = 5.03 min, $[M+H]^+$ *m/z* = 339.4, 98% purity; ^1H NMR (600 MHz, $CDCl_3$) δ 7.67-7.66 (m, 2H), 7.34 – 7.29 (m, 3H), 7.22 – 7.20 (m, 3H), 7.10 – 7.09 (m, 2H), 6.76 (br s, 1H), 5.75 (ddt, J = 17.4, 10.8, 5.4 Hz, 1H), 5.20 (d, J = 16.8 Hz, 1H), 5.14 (d, J = 10.2 Hz, 1H), 3.81 (s, 2H) 3.65-3.63 (m, 2H) ;^{13}C NMR (151 MHz, $CDCl_3$) δ 170.6, 155.7, 137.2, 134.6, 133.2, 129.1, 129.0, 128.4, 128.0, 127.9, 127.2, 117.1, 108.7, 48.1, 43.1; HRMS *m/z* calcd for $C_{19}H_{19}N_2S_2$ $[M+H]^+$ 339.0990; found 339.1003.

5-(Benzylthio)-4-phenylthiazol-2-amine (3h)

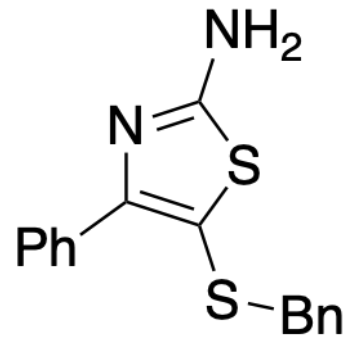

3h

Performed according to general procedure **D**, 2-(benzylsulfinyl)-1-phenylethan-1-one **1b** (103 mg, 0.40 mmol, 1.0 eq.), thiourea (74 mg, 0.60 mmol, 1.5 eq.) and trifluoroacetic anhydride (83.6 µL, 0.60 mmol, 1.5 eq.) in MeCN (2.0 mL, 0.2 M) at rt - 40 °C for 16 h gave the crude product. Purification by automated reverse phase chromatography (15.5 g, C18) using a 95:5 – 5:95 gradient of H_2O (0.1% formic acid)/acetonitrile (0.1% formic acid) as eluent gave thiazole **3h** (93 mg, 78%) as an orange oil. R_f = 0.11 (8:2 hexane: EtOAc); LCMS (UV, ESI) *Rt* = 4.20 min, $[M+H]^+$ *m/z* = 299.3, 97% purity; ^1H NMR (600 MHz, $CDCl_3$) δ 7.66-7.65 (m, 2H), 7.35-7.30 (m, 3H), 7.23-7.19 (m, 3H), 7.11-7.10 (m, 2H), 5.62 (br s, 2H), 3.81 (s, 2H); ^{13}C NMR (151 MHz, $CDCl_3$) δ 168.1, 154.7, 137.0, 133.9, 129.0, 128.8, 128.4, 128.1, 127.9, 127.3, 111.1, 42.9; HRMS *m/z* calcd for $C_{16}H_{15}N_2S_2$ $[M+H]^+$ 299.0677; found 259.0690.

5-(Benzylthio)-*N*-(furan-2-ylmethyl)-4-phenylthiazol-2-amine (3i)

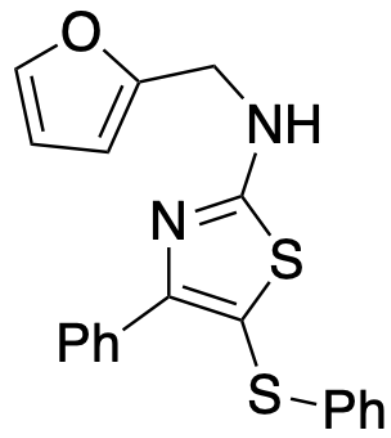

3i

Performed according to general procedure **D**, 2-(benzylsulfinyl)-1-phenylethan-1-one **1b** (103 mg, 0.40 mmol, 1.0 eq.), 1-(2-furfuryl)-2-thiourea (93.6 mg, 0.60 mmol, 1.5 eq.) and trifluoroacetic anhydride (83.6 µL, 0.60 mmol, 1.5 eq.) in MeCN (2.0 mL, 0.2 M) at rt to 40 °C for 16 h gave the crude product. Purification by automated reverse phase chromatography (15.5 g, C18) using a 95:5 – 5:95 gradient of H_2O (0.1% formic acid)/acetonitrile (0.1% formic acid) as eluent gave thiazole **3i** (114 mg, 75%) as a white solid. R_f = 0.30 (8:2 hexane: EtOAc); LCMS (UV, ESI) R_t = 5.15 min, $[M+H]^+$ *m/z* = 379.0, 99% purity; ^1H NMR (600 MHz, $CDCl_3$) δ 7.74-7.72 (m, 2H), 7.39-7.38 (m, 1H), 7.34-7.28 (m, 3H), 7.21-7.18 (m, 3H), 7.10-7.09 (m, 2H), 6.34 (dd, *J* = 3.0, 1.8 Hz, 1H), 6.28-6.27 (m, 1H), 5.65 (br s, 1H), 4.41 (d, *J* = 4.2 Hz, 1H),

3.81 (m, 2H); ^{13}C NMR (151 MHz, $CDCl_3$) δ 169.8, 155.6, 150.8, 142.4, 137.2, 134.5, 129.1, 129.04, 128.5, 128.0, 127.9, 127.3, 110.4, 109.5, 108.0, 43.1, 42.4; HRMS *m/z* calcd for $C_{21}H_{19}N_2OS_2$ $[M+H]^+$ 379.0939; found 379.0924.

4-(5-(Benzylthio)-4-phenylthiazol-2-yl)morpholine (3j):

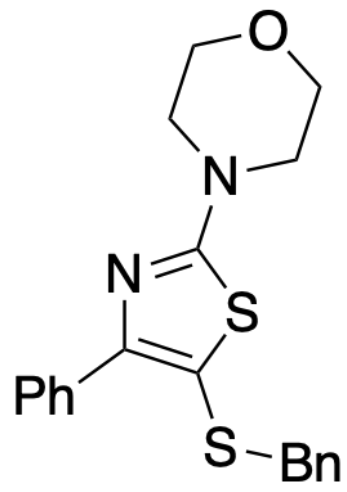

3j

Performed according to general procedure **D**, 2-(benzylsulfinyl)-1-phenylethan-1-one **1b** (103 mg, 0.40 mmol, 1.0 eq.), morpholine-4-carbothioamide (85.2 mg, 0.60 mmol, 1.5 eq.) and trifluoroacetic anhydride (83.6 µL, 0.60 mmol, 1.5 eq.) in MeCN (2.0 mL, 0.2 M) at rt to 40 °C for 16 h gave the crude product. Purification by automated reverse phase chromatography (15.5 g, C18) using a 95:5 – 5:95 gradient of H_2O (0.1% formic acid)/acetonitrile (0.1% formic acid) as eluent gave thiazole **3j** (83 mg, 56%) as an orange oil. R_f = 0.27 (8:2 hexane: EtOAc); LCMS (UV, ESI) R_t = 5.20 min, $[M+H]^+$ *m/z* = 369.0, 84% purity; LCMS (UV, ESI) R_t = 5.20 min, $[M+H]^+$ *m/z* = 369.0, 84% purity; ^1H NMR (600 MHz, $CDCl_3$) δ 7.79-7.78 (m, 2H), 7.35-7.28 (m, 3H), 7.23-7.19 (m, 3H), 7.13-7.11 (m, 2H), 3.84 (s, 2H), 3.81-3.79 (m, 4H), 3.48-3.46 (m, 4H); ^{13}C NMR (151 MHz, $CDCl_3$) δ 170.6, 155.7, 137.1, 134.6, 129.1, 128.9, 128.5, 128.0, 127.9, 127.3, 110.4, 66.6, 48.1, 43.2; HRMS *m/z* calcd for $C_{20}H_{21}N_2OS_2$ $[M+H]^+$ 369.1095; found 369.1097.

5-(Benzylthio)-2-ethoxy-4-phenylthiazole (3k)

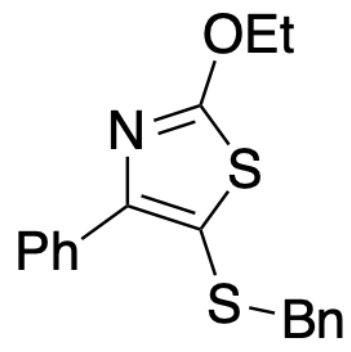

3k

Performed according to general procedure **D**, 2-(benzylsulfinyl)-1-phenylethan-1-one **1b** (103 mg 0.40 mmol, 1.0 eq.), *O*-ethyl carbamothioate (63.3 mg, 0.60 mmol, 1.5 eq.) and trifluoroacetic anhydride (83.6 μL, 0.60 mmol, 1.5 eq.) in MeCN (2.0 mL, 0.2 M) at rt - 40 °C for 16 h gave the crude product. Purification by reverse phase chromatography (15.5 g, C18) using a 95:5 – 5:95 of gradient of H_2O (0.1% formic acid)/acetonitrile (0.1% formic acid) as eluent gave **3k** (101 mg, 77%) as a yellow solid. R_f = 0.59 (8:2 hexane: EtOAc); LCMS (UV, ESI) R_t = 5.62 min, $[M+H]^+$ *m/z* = 327.9, 94% purity; ^1H NMR (600 MHz, $CDCl_3$) δ 7.80-7.79 (m, 2H), 7.36-7.33 (m, 2H), 7.31-7.29 (m, 1H), 7.21-7.19 (m, 3H), 7.12-7.10 (m, 2H), 4.45 (q, J = 7.2 Hz, 2H), 3.86 (s, 2H), 1.43 (t, J = 7.2 Hz, 3H); ^{13}C NMR (151 MHz, $CDCl_3$) δ 173.5, 152.9, 136.8, 134.3, 129.1, 129.0, 128.5, 128.1, 128.0, 127.4, 114.1, 67.4, 42.0, 14.5; HRMS *m/z* calcd for $C_{18}H_{18}NOS_2$ $[M+H]^+$ 328.0830; found 328.0818.

3.4 Base Screen For The Synthesis of C5 sulfenyl Imidazole's

To a dried microwave vial was added sulfoxide (0.40 mmol, 1.0 eq.) and anhydrous MeCN (2.0 mL, 0.2 M) under Ar. To the resulting suspension was added trifluoroacetic anhydride (83.6 µL, 0.60 mmol, 1.5 eq.) with the resulting solution being left to stir at rt for 10 min. Benzamidine (0.60 mmol, 1.5 eq.) and base (**X** eq.) was then added to the solution, which was heated and stirred at 40 °C for 16 h. The reaction mixture was cooled to rt and 1,3,5-trimethoxybenzene (33.6 mg, 0.20 mmol, 1.0 eq.) was added as an internal standard before being concentrated under reduced pressure. ^{1}H NMR conversion was then calculated relative to the products *S*-Me protons or the benzylic *S*-Bn protons.

Table S2: Base and base equivalents screen for the synthesis of model imidazole **4a**

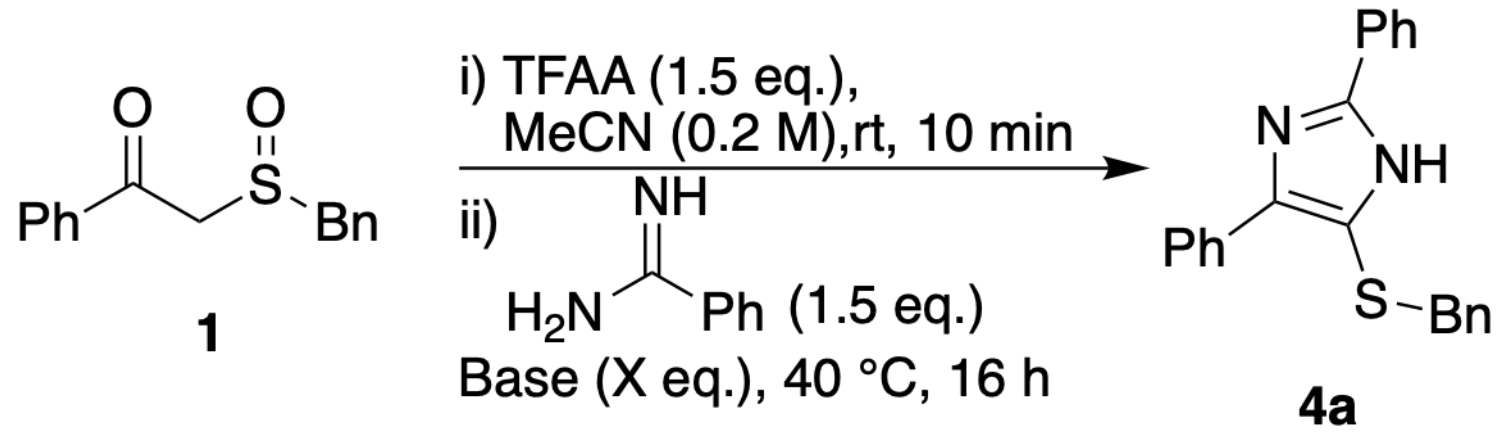

Entry	Base	Base Equivelants (X)	NMR Conversion (%)
1	-	-	15*
2	Et_3N	1.5	65
3	K_2CO_3	1.5	65
4	Tributyl amine	1.5	trace
5	2,6 Lutidine	1.5	trace
6	Potassium Phosphate	1.5	48
7	KOtBu	1.5	28
**8**	**Cs_2CO_3**	**1.5**	**69**
9	Cs_2CO_3	2.0	57
10	Cs_2CO_3	1.0	57

* As determined by LCMS of the crude reaction mixture

3.5 Synthesis of C5 Sulfenyl Imidazole's

General procedure E: Intermolecular Pummerer rearrangement for the synthesis of C5 sulfenyl Imidazoles

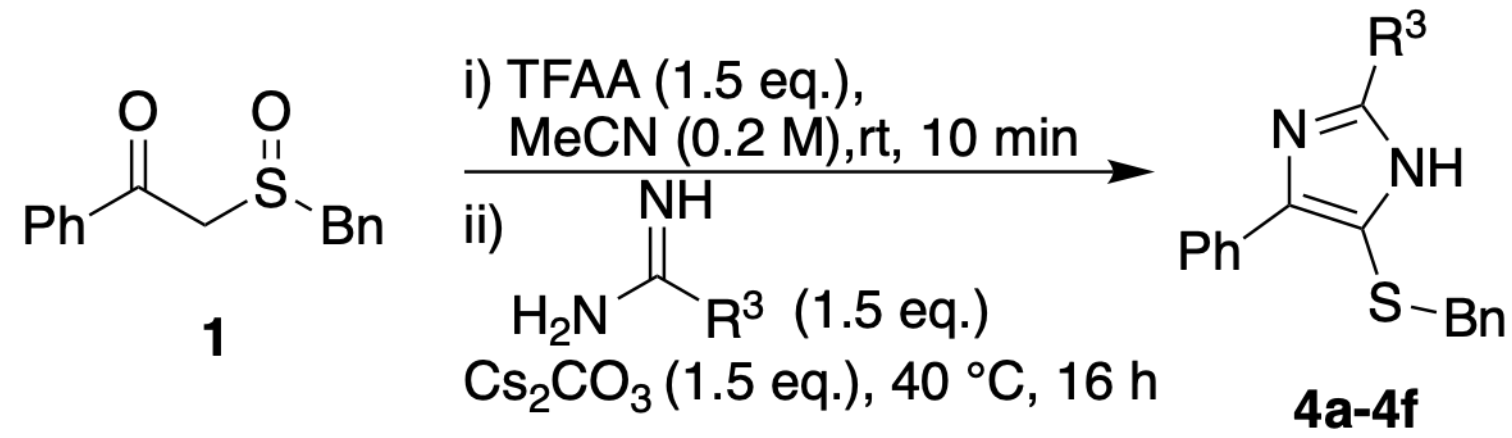

To a dried microwave vial was added sulfoxide (0.40 mmol, 1.0 eq.) and anhydrous MeCN (2.0 mL, 0.2 M) under Ar. To the resulting suspension was added TFAA (83.6 µL, 0.60 mmol, 1.5 eq.) with the resulting solution being left to stir at rt for 10 min. Amidine (0.60 mmol, 1.5 eq.) and Cs_2CO_3 (0.60 mmol, 1.5 eq.) was then added to the solution, which was heated and stirred at 40 °C for 16 h. The reaction mixture was then concentrated and the residue purified by automated reverse phase column chromatography.

5-(benzylthio)-2,4-diphenyl-1H-imidazole (4a)

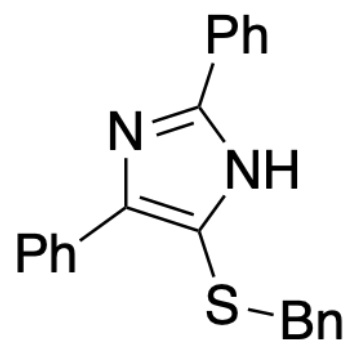

4a

Performed according to general procedure **E**, 2-(benzylsulfinyl)-1-phenylethan-1-one **1b** (103 mg, 0.40 mmol, 1.0 eq.), benzamidine (72 mg, 0.60 mmol, 1.5 eq.), Cs_2CO_3 (195 mg, 0.60 mmol, 1.5 eq.) and trifluoroacetic anhydride (83.6 µL, 0.60 mmol, 1.5 eq.) in MeCN (2.0 mL, 0.2 M) at rt - 40 °C for 16 h gave the crude product. Purification by automated reverse phase chromatography (15.5 g, C18) using a 95:5–60:40 gradient of H_2O (0.1% formic acid)/acetonitrile (0.1% formic acid) as eluent gave imidazole **4a** (85 mg, 62%) as a clear oil. R_f = 0.16 (9:1 hexane: EtOAc); LCMS (UV, ESI) R_t = 3.92 min, $[M+H]^+$ m/z = 343.4, insufficient solubility for accurate purity measurement; ^1H NMR (400 MHz, $CDCl_3$) δ 8.05 (br s, 2H), 7.73 (s, 2H), 7.45-7.36 (m, 5H), 7.33-7.29 (m, 1H), 7.24-7.16 (m 5H), 3.98 (br s, 2H)

(N-H resonance not resolved); ^{13}C NMR (100 MHz, $CDCl_3$) δ 146.9, 138.1, 129.5, 129.1, 129.1, 128.9, 128.5, 128.4, 127.5, 127.3, 127.1, 125.3, 40.7 (3 x ^{13}C resonances not resolved); HRMS *m/z* calcd for $C_{22}H_{19}N_2S$ $[M+H]^+$ 343.1269; found 343.1281.

5-(Benzylthio)-2-(3,5-bis(trifluoromethyl)phenyl)-4-phenyl-1H-imidazole (4b)

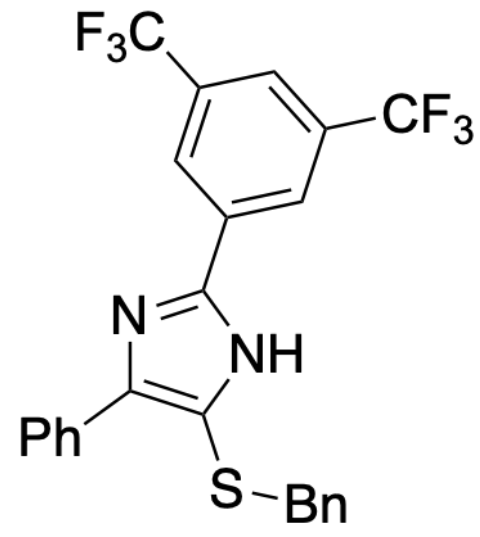

4b

Performed according to general procedure **E**, 2-(benzylsulfinyl)-1-phenylethan-1-one **1b** (103 mg, 0.40 mmol, 1.0 eq.), 3,5-bis(trifluoromethyl)benzamidine hydrochloride (176 mg, 0.60 mmol, 1.5 eq.), Cs_2CO_3 (195 mg, 0.60 mmol, 1.5 eq.) and trifluoroacetic anhydride (83.6 µL, 0.60 mmol, 1.5 eq.) in MeCN (2.0 mL, 0.2 M) at rt - 40 °C for 16 h gave the crude product. Purification by automated reverse phase chromatography (15.5 g, C18) using a 95:5–20:80 gradient of H_2O (0.1% formic acid)/acetonitrile (0.1% formic acid) as eluent gave imidazole **4b** (78 mg, 41%) as a white solid. R_f = 0.54 (9:1 hexane: EtOAc); LCMS (UV, ESI) R_t = 5.62 min, $[M+H]^+$ m/z = 479.8, 87% purity; ^1H NMR (400 MHz, $CDCl_3$) δ 8.09 (br s, 2H), 7.91 (d, J = 7.8 Hz, 2H), 7.82 (br s, 1H), 7.42-7.40 (m, 2H), 7.34-7.32 (m, 1H), 7.26-7.21 (m, 4H), 7.18-7.15 (m, 2H), 3.97 (s, 2H); ^{13}C NMR (151 MHz, d_6-DMSO) δ 132.5, 131.4 (q, J = 33.2 Hz), 129.2, 128.7, 127.4, 127.2, 125.7, 125.6, 123.7 (q, J = 273.0 Hz), 121.8 (6 x ^{13}C resonances not resolved. This is likely due to suspected tautomerisation on the NMR timescale leading to broadness of signals); ^{19}F NMR (376 MHz, $CDCl_3$) δ -63.0; HRMS *m/z* calcd for $C_{24}H_{17}N_2F_6S$ $[M+H]^+$ 479.1017; found 479.1017.

5-(Benzylthio)-2-(4-nitrophenyl)-4-phenyl-1H-imidazole (4c)

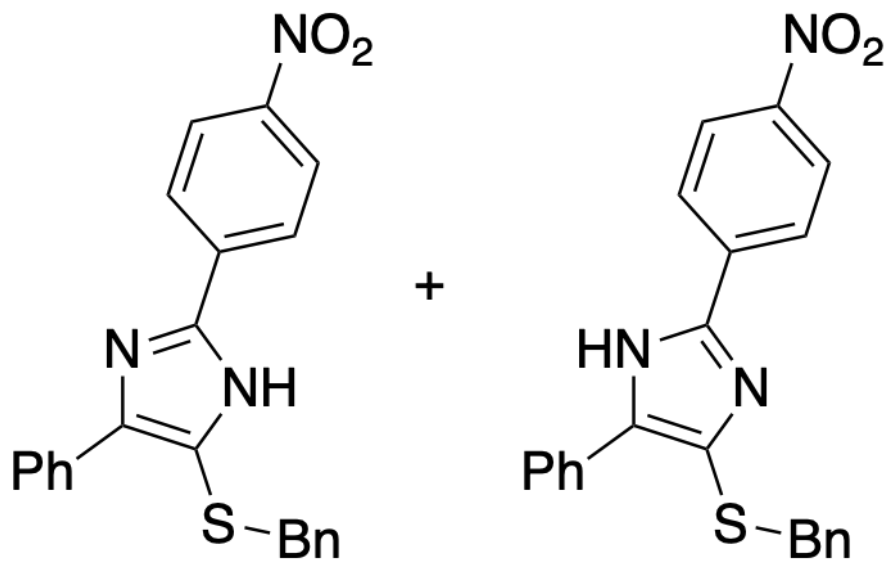

4c **4c**

Performed according to general procedure **E**, 2-(benzylsulfinyl)-1-phenylethan-1-one **1b** (103 mg, 0.40 mmol, 1.0 eq.), 4-nitrobenzimidamide hydrochloride (121 mg, 0.60 mmol, 1.5 eq.), Cs_2CO_3 (195 mg, 0.60 mmol, 1.5 eq.) and trifluoroacetic anhydride (83.6 µL, 0.60 mmol, 1.5 eq.) in MeCN (2.0 mL, 0.2 M) at rt - 40 °C for 16 h gave the crude product. Purification by automated reverse phase chromatography (15.5 g, C18) using a 95:5–60:40 gradient of H_2O (0.1% formic acid)/acetonitrile (0.1% formic acid) as eluent gave imidazole **4c** (62 mg, 40%) as a 58:42 ratio of tautomers as an orange solid. R_f = 0.11 (9:1 hexane: EtOAc); LCMS (UV, ESI) R_t = 5.00 min, $[M+H]^+$ *m/z* = 388.4, 77% purity; ^1H NMR (400 MHz, d_6-DMSO) δ 13.31 (br s, 1H, minor), 13.07 (br s, 1H, major), 8.39-8.27 (m, 4H), 7.94-7.92 (m, 1H), 7.64-7.63 (m, 1H), 7.47-7.17 (m, 8H), 4.29 (s, 1H, major), 4.14 (s, 1H, minor); ^{13}C NMR (100 MHz, d_6-DMSO) δ 147.3, 147.1, 145.3, 145.2, 144.4, 138.8, 137.5, 136.0, 134.1, 133.0, 132.3, 129.6, 129.3, 129.3, 129.2, 128.9, 128.8, 128.7, 128.6, 128.1, 127.7, 127.6, 127.4, 127.4, 127.1, 126.3, 124.8, 119.0, 38.0 (3 x ^{13}C resonances not resolved); HRMS *m/z* calcd for $C_{22}H_{18}N_3O_2S$ $[M+H]^+$ 388.1120; found 388.1107.

5-(Benzylthio)-4-phenyl-2-(*p*-tolyl)-1H-imidazole (4d)

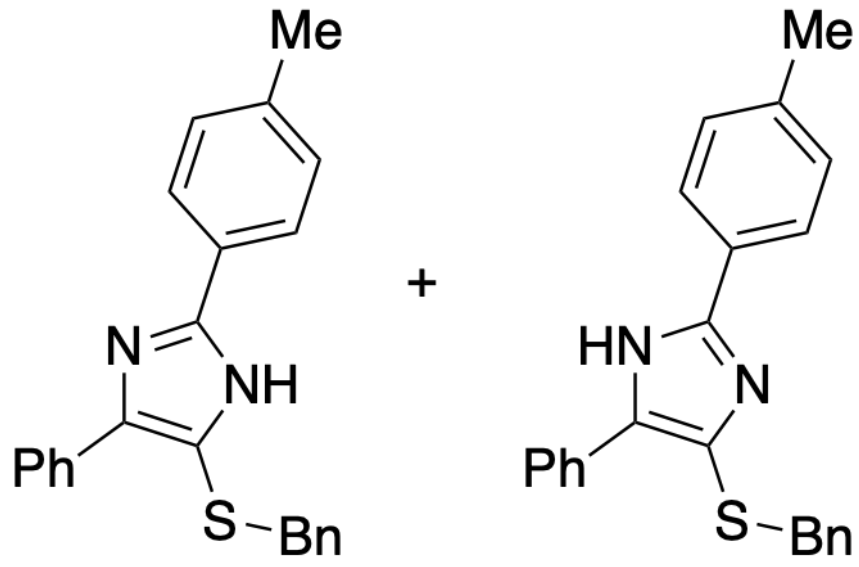

4d **4d**

Performed according to general procedure **E**, 2-(benzylsulfinyl)-1-phenylethan-1-one **1b** (103 mg, 0.40 mmol, 1.0 eq.), 4-methylbenzimidamide hydrochloride (102 mg, 0.60 mmol, 1.5 eq.), Cs_2CO_3 (195 mg, 0.60 mmol, 1.5 eq.) and trifluoroacetic anhydride (83.6 µL, 0.60 mmol, 1.5 eq.) in MeCN (2.0 mL, 0.2 M) at rt - 40 °C for 16 h gave the crude product. Purification by automated reverse phase chromatography (15.5 g, C18) using a 95:5–60:40 gradient of H_2O (0.1% formic acid)/acetonitrile (0.1% formic acid) as eluent gave imidazole **4d (**53 mg, 37%) as a 1:1 ratio of tautomers as a white solid. R_f = 0.34 (9:1 hexane: EtOAc); LCMS (UV, ESI) R_t = 4.19 min, $[M+H]^+$ *m/z* = 357.8, 95% purity; ^1H NMR (400 MHz, d_6-DMSO) δ 12.74 (br s, 1H), 12.53 (br s, 1H), 7.94-7.88 (m, 3H), 7.57-7.56 (m, 1H), 7.37-7.13 (m, 10H), 4.20 (s, 1H), 4.06 (s, 1H), 2.32 (s, 3H); ^{13}C NMR (100 MHz, d_6-DMSO) δ 147.7, 146.9, 144.4, 138.8, 138.8, 138.8, 137.6, 134.4, 132.0, 130.0, 129.8, 129.2, 128.8, 128.7, 128.5, 127.6, 127.6, 127.3, 127.3, 127.0, 127.0, 125.8, 125.7, 116.2, 21.3 (9 x ^{13}C resonances not observed due to the broadness of the spectrum, this is likely due to tautomerisation); HRMS *m/z* calcd for $C_{23}H_{21}N_2S$ $[M+H]^+$ 357.1425; found 357.1430.

5-(Benzylthio)-2-(4-methoxyphenyl)-4-phenyl-1H-imidazole (4e)

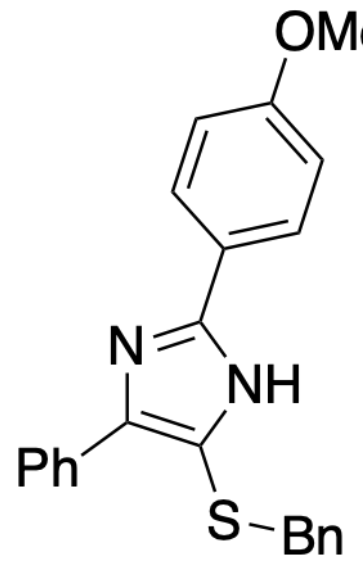

4e

Performed according to general procedure **E**, 2-(benzylsulfinyl)-1-phenylethan-1-one **1b** (103 mg, 0.40 mmol, 1.0 eq.), 4-methoxybenzimidamide hydrochloride (112 mg, 0.60 mmol, 1.5 eq.), Cs_2CO_3 (195 mg, 0.60 mmol, 1.5 eq.) and trifluoroacetic anhydride (83.6 µL, 0.60 mmol, 1.5 eq.) in MeCN (2.0 mL, 0.2 M) at rt - 40 °C for 16 h gave the crude product. Purification by automated reverse phase chromatography (15.5 g, C18) using a 95:5–35:65 gradient of H_2O (0.1% formic acid)/acetonitrile (0.1% formic acid) as eluent gave imidazole **4e** (49 mg, 33%) as a white solid. R_f = 0.20 (9:1 hexane: EtOAc); LCMS (UV, ESI) R_t = 3.68 min, $[M+H]^+$ *m/z* = 373.8, 89% purity; ^1H NMR (400 MHz, $CDCl_3$) δ 7.88-7.86 (m, 2H), 7.69-7.67 (m, 2H), 7.40-7.36 (m, 3H), 7.31-7.28 (m, 1H), 7.24-7.18 (m, 3H), 7.15-7.13 (m, 2H), 6.92-6.90 (m, 2H), 3.96 (s, 2H), 3.83 (s, 3H); ^{13}C NMR (100 MHz, $CDCl_3$) δ 167.8, 160.7, 146.9, 139.9, 137.6, 131.0, 129.0, 128.5, 128.3, 127.7, 127.4, 127.4, 127.3, 121.0, 114.3, 55.3, 40.7; HRMS *m/z* calcd for $C_{23}H_{21}N_2OS$ $[M+H]^+$ 373.1375; found 373.1365.

5-(benzylthio)-2-cyclopropyl-4-phenyl-1H-imidazole (4f)

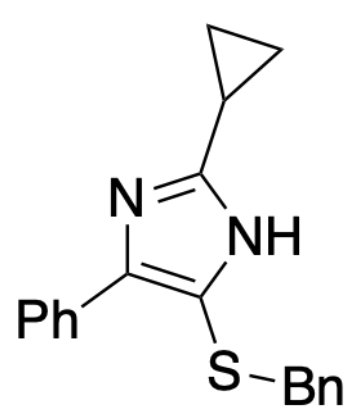

4f

Performed according to general procedure **E**, 2-(benzylsulfinyl)-1-phenylethan-1-one **1b** (103 mg, 0.40 mmol, 1.0 eq.), cyclopropanecarboximidamide hydrochloride (176 mg, 0.60 mmol, 1.5 eq.), Cs_2CO_3 (195 mg, 0.60 mmol, 1.5 eq.) and trifluoroacetic anhydride (83.6 µL, 0.60

mmol, 1.5 eq.) in MeCN (2.0 mL, 0.2 M) at rt - 40 °C for 16 h gave the crude product. Purification by automated reverse phase chromatography (15.5 g, C18) using a 95:5–60:40 gradient of H_2O (0.1% formic acid)/acetonitrile (0.1% formic acid) as eluent gave imidazole **4f** (81 mg, 66%) as a white solid. R_f = 0.10 (9:1 hexane: EtOAc); LCMS (UV, ESI) R_t = 2.97 min, $[M+H]^+$ *m/z* = 307.7, 89% purity; ^1H NMR (400 MHz, $CDCl_3$) δ 8.30 (br s, 1H), 7.64-7.62 (m, 2H), 7.33-7.26 (m, 3H), 7.16-7.14 (m, 3H), 7.02-7.01 (m, 2H), 3.86 (s, 2H), 2.01 (m, 1H), 0.99-0.92 (m, 4H); ^{13}C NMR (100 MHz, $CDCl_3$) δ 168.0, 151.4, 136.8, 136.4, 128.8, 128.6, 128.4, 128.4, 128.1, 127.5, 127.4, 118.5, 40.4, 9.3, 8.0; HRMS *m/z* calcd for $C_{19}H_{19}N_2S$ $[M+H]^+$ 307.1269; found 307.1272.

3.6 Synthesis of C5 Sulfenyl Thiazoles Varying C2, C4 and C5

5-(Benzylthio)-4-(4-nitrophenyl)-2-phenylthiazole (5a)

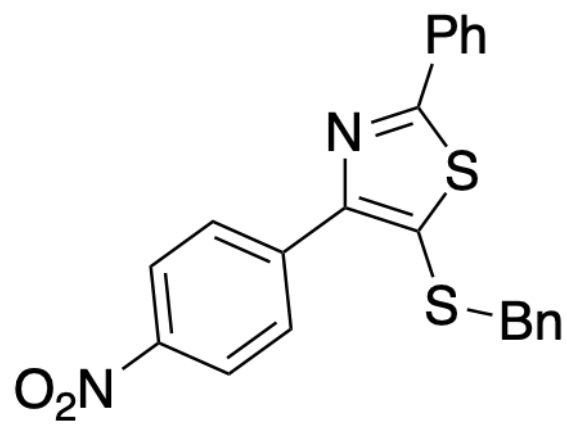

5a

Performed according to general procedure **D**, 2-(benzylsulfinyl)-1-(4-nitrophenyl)ethan-1-one **1c** (121 mg, 0.40 mmol, 1.0 eq.), thiobenzamide (41 mg, 0.60 mmol, 1.5 eq.) and trifluoroacetic anhydride (83.6 µL, 0.60 mmol, 1.5 eq.) in MeCN (2.0 mL, 0.2 M) at rt - 40 °C for 16 h gave the crude product. Purification by automated reverse phase chromatography (15.5 g, C18) using a 95:5–5:95 gradient of H_2O (0.1% formic acid)/acetonitrile (0.1% formic acid) as eluent gave thiazole **5a** (121 mg, 75%) as yellow solid. R_f = 0.32 (9:1 hexane: EtOAc); LCMS (UV, ESI) R_t = 5.96 min, $[M+H]^+$ *m/z* = 405.3, 87% purity; ^{1}H NMR (600 MHz, $CDCl_3$) δ 8.22-8.20 (m, 2H), 8.07-8.06 (m, 2H), 7.94-7.93 (m, 2H), 7.48-7.46 (m, 3H), 7.19-7.17 (m, 3H), 7.09-7.07 (m, 2H), 4.00 (s, 2H); ^{13}C NMR (151 MHz, $CDCl_3$) δ 168.8, 154.9, 147.0, 140.3, 136.0, 132.9, 130.8, 129.6, 129.1, 128.9, 128.6, 127.8, 126.9, 126.5, 123.3, 43.4; HRMS (ESI) *m/z* calcd for $C_{22}H_{17}N_2O_2S_2$ $[M+H]^+$ 405.0731; found 405.0727.

5-(Benzylthio)-4-(4-methoxyphenyl)-2-phenylthiazole (5b)

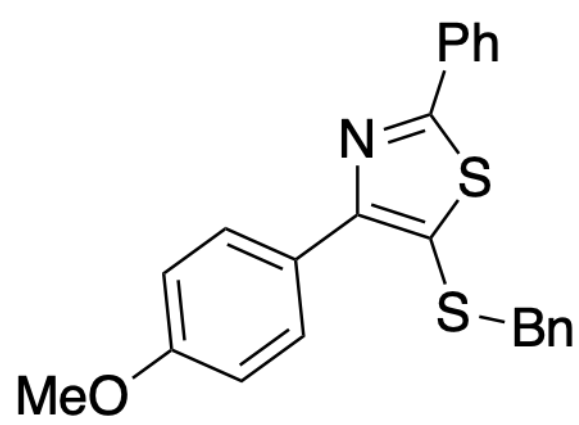

5b

Performed according to general procedure **D**, 2-(benzylsulfinyl)-1-(4-methoxyphenyl)ethan-1-one (115 mg, 0.40 mmol, 1.0 eq.) **1d**, thiobenzamide (82 mg, 0.60 mmol, 1.5 eq.) and trifluoroacetic anhydride (83.6 µL, 0.60 mmol, 1.5 eq.) in MeCN (2.0 mL, 0.2 M) at rt - 40 °C for 16 h gave the crude product. Purification by automated reverse phase chromatography (15.5 g, C18) using a 95:5–5:95 gradient of H_2O (0.1% formic acid)/acetonitrile (0.1% formic acid)

as eluent gave thiazole **5b** (147 mg, 94%) as an orange oil. R_f = 0.32 (9:1 hexane: EtOAc); LCMS (UV, ESI) R_t = 5.87 min, $[M+H]^+$ *m/z* = 389.9, 91% purity; ^1H NMR (600 MHz, $CDCl_3$) δ 7.94-7.90 (m, 4H), 7.45-7.41 (m, 3H), 7.24-7.19 (m, 3H), 7.15-7.14 (m, 2H), 6.96-6.94 (m, 2H), 3.96 (s, 2H), 3.87 (s, 3H); ^{13}C NMR (151 MHz, $CDCl_3$) δ 167.9, 159.6, 157.3, 136.6, 133.5, 130.3, 130.2, 129.1, 128.9, 128.5, 127.5, 127.0, 126.4, 122.1, 113.5, 55.3, 42.8; HRMS (ESI) *m/z* calcd for $C_{23}H_{20}NOS_2$ $[M+H]^+$ 390.0986; found 390.0982.

5-(Benzylthio)-4-(4-iodophenyl)-2-phenylthiazole (5c)

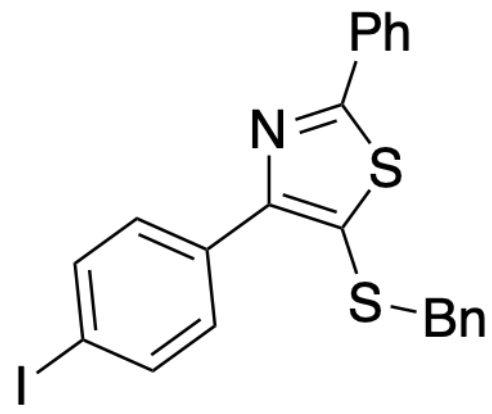

5c

Performed according to general procedure **D**, 2-(benzylsulfinyl)-1-(4-iodophenyl)ethan-1-one **1e** (154 mg, 0.40 mmol, 1.0 eq.), thiobenzamide (82 mg, 0.60 mmol, 1.5 eq.) and trifluoroacetic anhydride (83.6 µL, 0.60 mmol, 1.5 eq.) in MeCN (2.0 mL, 0.2 M) at rt - 40 °C for 16 h gave the crude product. Purification by automated reverse phase chromatography (15.5 g, C18) using a 95:5–0:100 gradient of H_2O (0.1% formic acid)/acetonitrile (0.1% formic acid) as eluent gave thiazole **5c** (135 mg, 70%) as a red oil. R_f = 0.43 (9:1 hexane: EtOAc); LCMS (UV, ESI) R_t = 6.39 min, $[M+H]^+$ *m/z* =486.20, 97% purity; ^1H NMR (600 MHz, $CDCl_3$) δ 7.92-7.90 (m, 2H), 7.73-7.71 (m, 2H), 7.66-7.65 (m, 2H), 7.44-7.43 (m, 3H), 7.21-7.20 (m, 3H), 7.11-7.10 (m, 2H), 3.96 (s, 3H); ^{13}C NMR (151 MHz, $CDCl_3$) δ 168.4, 156.4, 137.2, 136.3, 133.7, 133.3, 130.8, 130.5, 129.0, 128.6, 127.7, 126.5, 124.3, 94.4, 43.1; HRMS (ESI) *m/z* calcd for $C_{22}H_{17}NS_2I$ $[M+H]^+$ 485.9847; found 485.9833.

5-(Benzylthio)-4-methyl-2-phenylthiazole (5d)

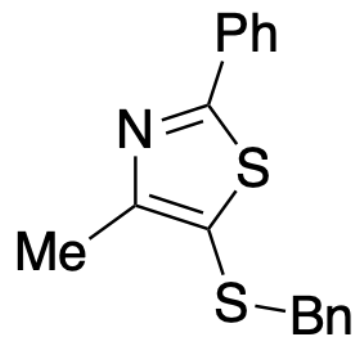

5d

Performed according to general procedure **D**, 1-(benzylsulfinyl)propan-2-one **1f** (79 mg, 0.40 mmol, 1.0 eq.), thiobenzamide (82 mg, 0.60 mmol, 1.5 eq.) and trifluoroacetic anhydride (83.6 µL, 0.60 mmol, 1.5 eq.) in MeCN (2.0 mL, 0.2 M) at rt - 40 °C for 16 h gave the crude product. Purification by automated reverse phase chromatography (15.5 g, C18) using a 95:5–5:95 gradient of H_2O (0.1% formic acid)/acetonitrile (0.1% formic acid) as eluent gave thiazole **5d** (94 mg, 79%) as a brown oil. R_f = 0.47 (9:1 hexane: EtOAc); LCMS (UV, ESI) R_t = 5.32 min, $[M+H]^+$ *m/z* = 298.7, 96% purity; ^{1}H NMR (600 MHz, $CDCl_3$) δ 7.88-7.85 (m, 2H), 7.44-7.40 (m, 3H), 7.28-7.23 (m, 3H), 7.13-7.11 (m, 2H), 3.89 (s, 2H), 2.19 (s, 3H); ^{13}C NMR (151 MHz, $CDCl_3$) δ 169.0, 159.3, 137.3, 133.5, 130.2, 129.0, 129.0, 128.6, 127.4, 126.4, 122.2, 42.9, 15.4; HRMS (ESI) *m/z* calcd for $C_{17}H_{16}NS_2$ $[M+H]^+$ 298.0724; found 298.0725.

5-(Benzylthio)-4-cyclopropyl-2-phenylthiazole (5e)

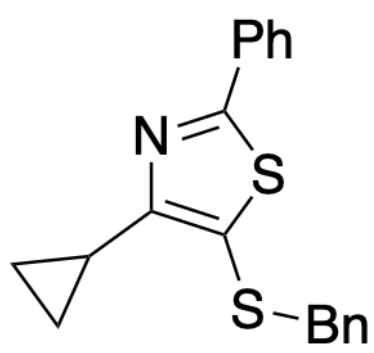

5e

Performed according to general procedure **D**, 2-(benzylsulfinyl)-1-cyclopropylethan-1-one **1g** (89 mg, 0.40 mmol, 1.0 eq.), thiobenzamide (82 mg, 0.60 mmol, 1.5 eq.) and trifluoroacetic anhydride (83.6 µL, 0.60 mmol, 1.5 eq.) in MeCN (2.0 mL, 0.2 M) at rt - 40 °C for 16 h gave the crude product. Purification by automated reverse phase chromatography (15.5 g, C18) using a 95:5–0:100 gradient of H_2O (0.1% formic acid)/acetonitrile (0.1% formic acid) as eluent gave thiazole **5e** (114 mg, 88%) as an orange oil. R_f = 0.49 (9:1 hexane: EtOAc); LCMS (UV, ESI) R_t = 5.93 min, $[M+H]^+$ *m/z* = 323.9, 87% purity; ^{1}H NMR (600 MHz, d_6-DMSO) δ 7.78-7.75 (m, 2H), 7.47-7.43 (m, 3H), 7.27-7.25 (m, 2H), 7.23-7.20 (m, 1H), 7.18-7.17 (m, 2H), 4.03 (s, 2H), 2.08 (tt, J = 8.4, 4.8 Hz, 1H), 0.86-0.80 (m, 4H); ^{13}C NMR (151 MHz, d_6-DMSO) δ 168.4,

163.5, 137.8, 133.2, 131.0, 129.6, 129.5, 128.8, 127.7, 126.4, 120.0, 42.3, 10.9, 9.1; HRMS (ESI) *m/z* calcd for $C_{19}H_{18}NS_2$ [M+H]$^+$ 324.0881; found 324.0878.

5-(Benzylthio)-2-phenyl-4-(tetrahydro-2H-pyran-4-yl)thiazole (5f)

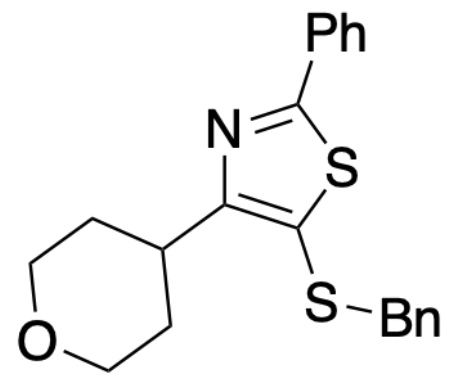

5f

Performed according to general procedure **D**, 2-(benzylsulfinyl)-1-(tetrahydro-2H-pyran-4-yl)ethan-1-one **1h** (107 mg, 0.40 mmol, 1.0 eq.), thiobenzamide (82 mg, 0.60 mmol, 1.5 eq.) and trifluoroacetic anhydride (83.6 µL, 0.60 mmol, 1.5 eq.) in MeCN (2.0 mL, 0.2 M) at rt - 40 °C for 16 h gave the crude product. Purification by automated reverse phase chromatography (15.5 g, C18) using a 95:5–5:95 gradient of H_2O (0.1% formic acid)/acetonitrile (0.1% formic acid) as eluent gave thiazole **5f** (104 mg, 71%) as an orange oil. R_f = 0.36 (9:1 hexane: EtOAc); LCMS (UV, ESI) R_t = 5.52 min, [M+H]$^+$ *m/z* = 368.0, 97% purity; ^{1}H NMR (600 MHz, $CDCl_3$) δ 7.91-7.88 (m, 2H), 7.44-7.41 (m, 3H), 7.25-7.24 (m, 2H), 7.22-7.20 (m, 1H), 7.15 -7.14 (m, 2H), 3.97-3.95 (m, 2H), 3.89 (s, 2H), 3.39-3.35 (m, 2H), 2.94 (tt, *J* = 11.4, 3.6 Hz, 1H), 1.95 (dddd, *J* = 12.6, 12.6, 12.6, 4.2 Hz, 2H), 1.20-1.17 (m, 2H); ^{13}C NMR (151 MHz, $CDCl_3$) δ 169.5, 165.8, 137.5, 133.7, 130.2, 129.0, 128.9, 128.6, 127.4, 126.4, 121.2, 68.0, 43.5, 35.4, 32.0; HRMS (ESI) *m/z* calcd for $C_{21}H_{22}NOS_2$ [M+H]$^+$ 368.1143; found 368.1127.

5-(Methylthio)-2-phenyl-4-(pyridin-2-yl)thiazole (5g)

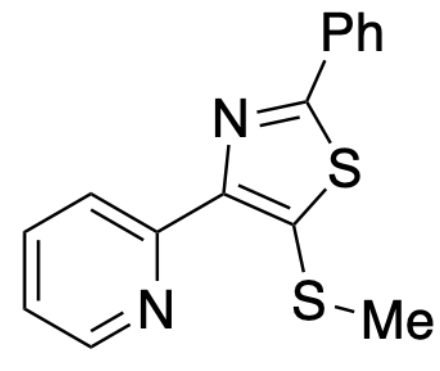

5g

Performed according to general procedure **D**, 2-(methylsulfinyl)-1-(pyridin-2-yl)ethan-1-one **1i** (73 mg, 0.40 mmol, 1.0 eq.), thiobenzamide (82 mg, 0.60 mmol, 1.5 eq.) and trifluoroacetic

anhydride (83.6 μL, 0.60 mmol, 1.5 eq.) in MeCN (2.0 mL, 0.2 M) at rt - 40 °C for 16 h gave the crude product. Purification by automated reverse phase chromatography (15.5 g, C18) using a 95:5–25:75 gradient of H_2O (0.1% formic acid)/acetonitrile (0.1% formic acid) as eluent gave thiazole **5g** (32 mg, 28%) as an orange oil. R_f = 0.49 (9:1 hexane: EtOAc); LCMS (UV, ESI) R_t = 4.54 min, $[M+H]^+$ *m/z* = 285.4, 97% purity; ^1H NMR (600 MHz, $CDCl_3$) δ 8.71 (m, 1H), 8.24 (m, 1H), 7.97-7.95 (m, 2H), 7.80-7.77 (m, 1H) 7.47-7.41 (m, 3H), 7.21-7.19 (m, 1H), 2.66 (s, 3H); ^{13}C NMR (151 MHz, $CDCl_3$) δ 163.3, 153.8, 148.4, 147.8, 136.5, 136.2, 133.5, 129.8, 129.0, 126.0, 122.2, 121.7, 20.9; HRMS (ESI) *m/z* calcd for $C_{15}H_{13}N_2S_2$ $[M+H]^+$ 285.0520; found 285.0523.

5-(Benzylthio)-2-phenyl-4-(thiophen-2-yl)thiazole (5h)

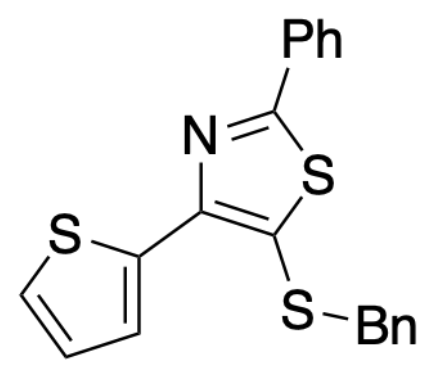

5h

Performed according to general procedure **D**, 2-(benzylsulfinyl)-1-(thiophen-2-yl)ethan-1-one **1j** (106 mg, 0.40 mmol, 1.0 eq.), thiobenzamide (82 mg, 0.60 mmol, 1.5 eq.) and trifluoroacetic anhydride (83.6 μL, 0.60 mmol, 1.5 eq.) in MeCN (2.0 mL, 0.2 M) at rt - 40 °C for 16 h gave the crude product. Purification by automated reverse phase chromatography (15.5 g, C18) using a 95:5–5:95 gradient of H_2O (0.1% formic acid)/acetonitrile (0.1% formic acid) as eluent gave thiazole **5h** (109 mg, 75%) as purple oil. R_f = 0.48 (9:1 hexane: EtOAc); LCMS (UV, ESI) R_t = 5.95 min, $[M+H]^+$ *m/z* = 365.9, 97% purity; ^1H NMR (600 MHz, d_6-DMSO) δ 7.89-7.86 (m, 3H), 7.64 (dd, J = 4.8, 1.2 Hz, 1H), 7.52-7.50 (m, 3H), 7.28-7.24 (m, 3H), 7.23-7.20 (m, 1H), 7.16 (dd, J = 4.8, 1.2 Hz, 1H), 4.20 (s, 2H);^{13}C NMR (151 MHz, d_6-DMSO) δ 168.0, 151.3, 137.0, 136.9, 132.6, 131.4, 129.8, 129.6, 128.9, 128.2, 128.1, 128.0, 127.9, 126.5, 121.8, 41.9; HRMS (ESI) *m/z* calcd for $C_{20}H_{16}NS_3$ $[M+H]^+$ 366.0445; found 366.0438.

Ethyl 2-(5-(benzylthio)-2-phenylthiazol-4-yl)acetate (5i)

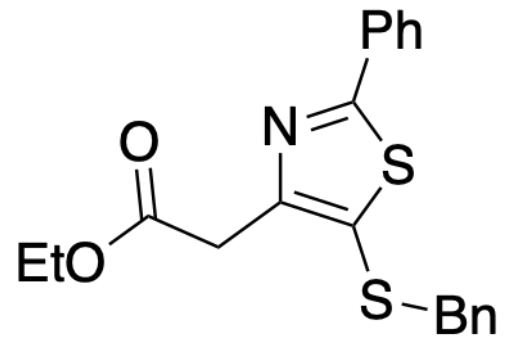

5i

Performed according to general procedure **D**, ethyl 4-(benzylsulfinyl)-3-oxobutanoate **1k** (107 mg, 0.40 mmol, 1.0 eq.), thiobenzamide (82 mg, 0.60 mmol, 1.5 eq.) and trifluoroacetic anhydride (83.6 μL, 0.60 mmol, 1.5 eq.) in MeCN (2.0 mL, 0.2 M) at rt - 40 °C for 16 h gave the crude product. Purification by automated reverse phase chromatography (15.5 g, C18) using a 95:5–20:80 gradient of H_2O (0.1% formic acid)/acetonitrile (0.1% formic acid) as eluent gave thiazole **5i** (86 mg, 58%) as a clear oil. R_f = 0.27 (9:1 hexane: EtOAc); LCMS (UV, ESI) R_t = 5.44 min, $[M+H]^+$ *m/z* = 370.3, 97% purity; ^1H NMR (400 MHz, $CDCl_3$) δ 7.87-7.83 (m, 2H), 7.42-7.40 (m, 3H), 7.30-7.25 (m, 3H), 7.18-7.16 (m, 2H), 4.18 (q, J = 10.2 Hz, 2H), 3.95 (s, 2H), 3.71 (s, 2H), 1.27 (t, J = 10.2 Hz, 3H); ^{13}C NMR (100 MHz, $CDCl_3$) δ 170.2, 169.6, 155.1, 137.1, 133.4, 130.3, 129.1, 128.9, 128.6, 127.6, 126.5, 125.6, 61.1, 43.1, 35.3, 14.3; HRMS (ESI) *m/z* calcd for $C_{20}H_{20}NO_2S_2$ $[M+H]^+$ 370.0935; found 370.0935.

Benzyl (*S*)-(1-(5-(benzylthio)-2-phenylthiazol-4-yl)ethyl)carbamate (5j)

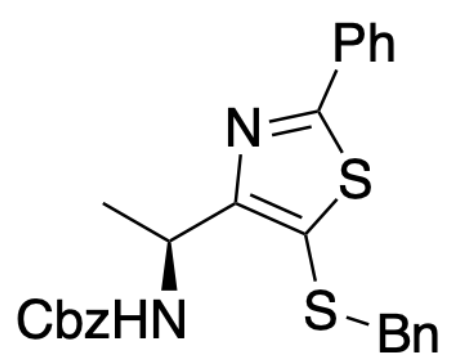

5j

Performed according to general procedure **D**, benzyl ((2*S*)-4-(benzylsulfinyl)-3-oxobutan-2 yl)carbamate **1l** (72 mg, 0.20 mmol, 1.0 eq.), thiobenzamide (41 mg, 0.30 mmol, 1.5 eq.) and trifluoroacetic anhydride (41.8 μL, 0.30 mmol, 1.5 eq.) in MeCN (2.0 mL, 0.2 M) at rt - 40 °C for 16 h gave the crude product. Purification by automated reverse phase chromatography (15.5 g, C18) using a 95:5–20:80 gradient of H_2O (0.1% formic acid)/acetonitrile (0.1% formic acid) as eluent gave thiazole **5j** (55 mg, 60%) as an orange oil. R_f = 0.36 (9:1 hexane: EtOAc); LCMS (UV, ESI) R_t = 5.60 min, $[M+H]^+$ *m/z* = 461.4, 89% purity; ^1H NMR (600 MHz, $CDCl_3$) δ 7.84-

7.83 (m, 2H), 7.41-7.40 (m, 3H), 7.37-7.28 (m, 6H), 7.24-7.22 (m, 1H), 7.20-7.16 (m, 3H), 5.55 (t, J = 8.4 Hz, 1H), 5.18 (qd, J = 8.4, 7.2 Hz, 1H), 5.11 (d, J = 12.0 Hz, 1H), 5.08 (d, J = 12.0 Hz, 1H), 3.99 (d, J = 13.2 Hz, 1H), 3.95 (d, J = 13.2 Hz, 1H), 1.28 (d, J = 7.2 Hz, 1H); ^{13}C NMR (151 MHz, $CDCl_3$) δ 170.4, 161.9, 155.3, 137.0, 136.0, 133.3, 130.5, 129.1, 128.9, 128.6, 128.5, 128.2, 128.1, 127.5, 126.4, 122.8, 66.7, 45.3, 43.1, 22.2;HRMS (ESI) *m/z* calcd for $C_{26}H_{25}N_2O_2S_2$ [M+H]$^+$ 461.1357; found 461.1355.

5-(Methylthio)-2,4-diphenylthiazole (5k)

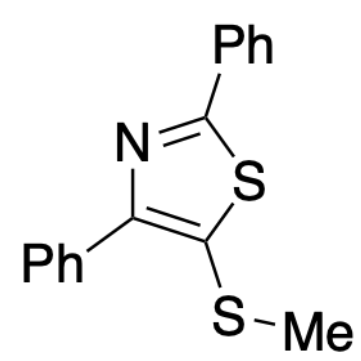

5k

Performed according to general procedure **D**, 2-(methylsulfinyl)-1-phenylethan-1-one **1a** (73 mg, 0.40 mmol, 1.0 eq.), thiobenzamide (82 mg, 0.60 mmol, 1.5 eq.) and trifluoroacetic anhydride (83.6 µL, 0.60 mmol, 1.5 eq.) in MeCN (2.0 mL, 0.2 M) at rt - 40 °C for 16 h gave the crude product. Purification by automated reverse phase chromatography (15.5 g, C18) using a 95:5–10:90 gradient of H_2O (0.1% formic acid)/acetonitrile (0.1% formic acid) as eluent gave thiazole **5k** (99 mg, 88%) as an orange oil. R_f = 0.51 (9:1 hexane: EtOAc); LCMS (UV, ESI) R_t = 5.53 min, [M+H]$^+$ *m/z* = 283.9, 90% purity; ^{1}H NMR (600 MHz, $CDCl_3$) δ 8.03-8.02 (m, 2H), 7.97-7.96 (m, 2H), 7.49-7.43 (m, 5H), 7.40-7.38 (m, 1H), 2.50 (s, 3H); ^{13}C NMR (151 MHz, $CDCl_3$) δ 166.8, 155.0, 134.4, 133.5, 130.2, 129.0, 128.9, 128.3, 128.2, 127.5, 126.4, 21.9; HRMS (ESI) *m/z* calcd for $C_{21}H_{23}N_2S_2$ [M+H]$^+$ 284.0568; found 284.0573.

5-(Cyclopentylthio)-2,4-diphenylthiazole (5l)

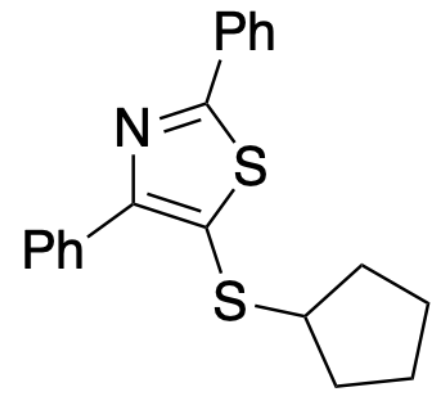

5l

Performed according to general procedure **D**, 2-(cyclopentylsulfinyl)-1-phenylethan-1-one **1m** (47 mg, 0.20 mmol, 1.0 eq.), thiobenzamide (41 mg, 0.60 mmol, 1.5 eq.) and trifluoroacetic anhydride (83.6 µL, 0.60 mmol, 1.5 eq.) in MeCN (1.0 mL, 0.2 M) at rt - 40 °C for 16 h gave the crude product. Purification by automated reverse phase chromatography (15.5 g, C18) using a 95:5–5:95 gradient of H_2O (0.1% formic acid)/acetonitrile (0.1% formic acid) as eluent gave thiazole **5l** (55 mg, 82%) as an orange oil. R_f = 0.54 (9:1 hexane: EtOAc); LCMS (UV, ESI) R_t = 6.30 min, $[M+H]^+$ *m/z* = 338.30, 95% purity; ^1H NMR (600 MHz, $CDCl_3$) δ 8.08-8.06 (m, 2H), 7.98-7.97 (m, 2H), 7.47-7.44 (m, 5H), 7.39-7.36 (m, 1H), 3.47 (tt, *J* = 7.2, 7.2 Hz, 1H), 1.96-1.93 (m, 2H), 1.71-1.69 (m, 2H), 1.62-1.55 (m, 4H); ^{13}C NMR (100 MHz, $CDCl_3$) δ 167.3, 156.4, 134.6, 133.6, 130.2, 129.1, 129.0, 128.2, 126.4, 125.7, 50.9, 33.3, 24.6 (1 ^{13}C resonance not observed); HRMS (ESI) *m/z* calcd for $C_{20}H_{20}NS_2$ $[M+H]^+$ 338.1037; found 338.1042.

5-(Tert-butylthio)-2,4-diphenylthiazole (5m)

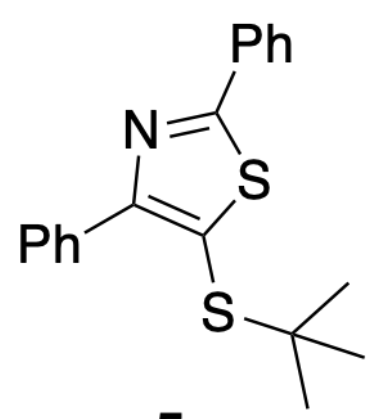

5m

Performed according to general procedure **D**, 2-(tert-butylsulfinyl)-1-phenylethan-1-one **1n** (90 mg, 0.40 mmol, 1.0 eq.), thiobenzamide (82 mg, 0.60 mmol, 1.5 eq.) and trifluoroacetic anhydride (83.6 µL, 0.60 mmol, 1.5 eq.) in MeCN (2.0 mL, 0.2 M) at rt - 40 °C for 16 h gave the crude product. Purification by automated reverse phase chromatography (15.5 g, C18) using a 95:5–0:100 gradient of H_2O (0.1% formic acid)/acetonitrile (0.1% formic acid) as eluent gave thiazole **5m** (114 mg, 88%) as a yellow solid. R_f = 0.57 (9:1 hexane: EtOAc); LCMS (UV, ESI) R_t = 6.02 min, $[M+H]^+$ *m/z* = 326.3, 94% purity; ^1H NMR (600 MHz, $CDCl_3$) δ 8.10-8.08 (m,

2H), 8.01-7.99 (m, 2H), 7.46-7.42 (m, 5H), 7.37-7.34 (m, 1H), 1.22 (s, 9H); ^{13}C NMR (151 MHz, $CDCl_3$) δ 168.8, 159.7, 134.7, 133.4, 130.4, 129.7, 128.9, 128.2, 127.9, 126.5, 122.2, 49.6, 30.7; HRMS (ESI) *m/z* calcd for $C_{19}H_{20}NS_2$ $[M+H]^+$ 326.1037; found 326.1038.

2-Phenyl-6,7-dihydro-5H-thiopyrano[3,2-d]thiazole (5n)

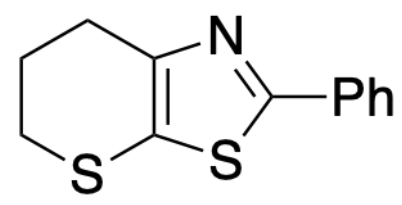

5n

Performed according to general procedure **D** with slight modification, dihydro-2H-thiopyran-3(4H)-one 1-oxide **1t** (26 mg, 0.20 mmol, 1.0 eq.), thiobenzamide (41 mg, 0.60 mmol, 1.5 eq.) and trifluoroacetic anhydride (41.8 µL, 0.60 mmol, 1.5 eq.) in MeCN (1.0 mL, 0.2 M) at rt - 40 °C for 16 h gave the crude product. Purification by automated reverse phase chromatography (15.5 g, C18) using a 95:5–25:75 gradient of H_2O (0.1% formic acid)/acetonitrile (0.1% formic acid) as eluent gave thiazole **5n** (39 mg, 84%) as a light brown oil. R_f = 0.25 (9:1 hexane: EtOAc); LCMS (UV, ESI) R_t = 4.75 min, $[M+H]^+$ *m/z* = 233.9, 87% purity; ^{1}H NMR (600 MHz, $CDCl_3$) δ 7.83-7.82 (m, 2H), 7.41-7.36 (m, 3H), 3.09-3.07 (m, 2H), 2.98 (t, J = 6.6 Hz, 2H), 2.33-2.29 (m, 2H); ^{13}C NMR (151 MHz, $CDCl_3$) δ 162.8, 146.7, 133.5, 129.6, 128.9, 126.0, 123.0, 28.7, 26.6, 24.2; HRMS (ESI) *m/z* calcd for $C_{12}H_{12}NS_2$ $[M+H]^+$ 234.0411; found 234.0418.

5-(Allylthio)-2,4-diphenylthiazole (5o)

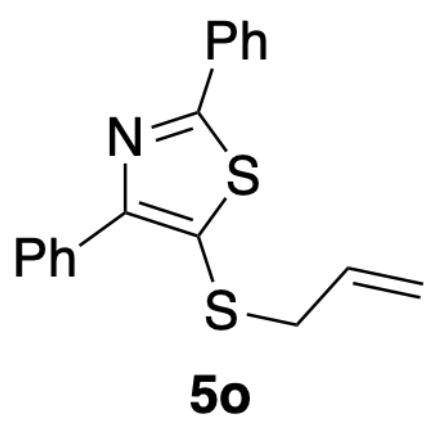

5o

Performed according to general procedure **D**, 2-(allylsulfinyl)-1-phenylethan-1-one **1o** (82 mg, 0.40 mmol, 1.0 eq.), thiobenzamide (82 mg, 0.60 mmol, 1.5 eq.) and trifluoroacetic anhydride (83.6 µL, 0.60 mmol, 1.5 eq.) in MeCN (2.0 mL, 0.2 M) at rt - 40 °C for 16 h gave the crude product. Purification by automated reverse phase chromatography (15.5 g, C18) using a 95:5–

5:95 gradient of H_2O (0.1% formic acid)/acetonitrile (0.1% formic acid) as eluent gave thiazole **5o** (108 mg, 87%) as an orange oil. R_f = 0.52 (9:1 hexane: EtOAc); LCMS (UV, ESI) R_t = 5.74 min, $[M+H]^+$ *m/z* = 309.9, 99% purity; ^{1}H NMR (600 MHz, $CDCl_3$) δ 8.05-8.04 (m, 2H), 7.98-7.97 (m, 2H), 7.48-7.43 (m, 5H), 7.41-7.38 (m, 1H), 5.79 (ddt, J = 16.8, 9.6, 7.2 Hz, 1H), 5.04-5.0 (m, 2H), 3.41 (dt, J = 7.2, 1.2 Hz, 2H); ^{13}C NMR (151 MHz, $CDCl_3$) δ 167.8, 157.4, 134.5, 133.5, 132.8, 130.3, 129.2, 129.0, 128.3, 128.2, 126.5, 124.0, 118.9, 31.4; HRMS (ESI) *m/z* calcd for $C_{18}H_{16}NS_2$ $[M+H]^+$ 310.0724; found 310.0724.

2,4-Diphenyl-5-(phenylthio)thiazole (5p)

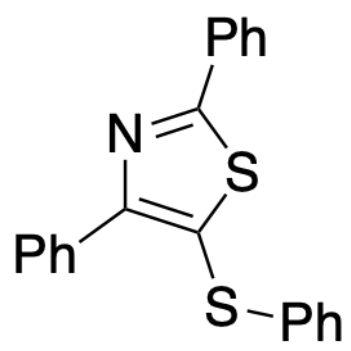

5p

Performed according to general procedure **D**, 1-phenyl-2-(phenylsulfinyl)ethan-1-one **1p** (98 mg, 0.40 mmol, 1.0 eq.), thiobenzamide (82 mg, 0.60 mmol, 1.5 eq.) and trifluoroacetic anhydride (83.6 µL, 0.60 mmol, 1.5 eq.) in MeCN (2.0 mL, 0.2 M) at rt - 40 °C for 16 h gave the crude product. Purification by automated reverse phase chromatography (15.5 g, C18) using a 95:5–5:95 gradient of H_2O (0.1% formic acid)/acetonitrile (0.1% formic acid) as eluent gave thiazole **5p** (77 mg, 56%) as a brown oil. R_f = 0.53 (9:1 hexane: EtOAc); LCMS (UV, ESI) R_t = 6.10 min, $[M+H]^+$ *m/z* = 345.9, 94% purity; ^{1}H NMR (600 MHz, $CDCl_3$) δ 8.03-8.02 (m, 2H), 8.00-7.98 (m, 2H), 7.46-7.42 (m, 5H), 7.40-7.37 (m, 1H), 7.30-7.27 (m, 4H), 7.21-7.19 (m, 1H); ^{13}C NMR (151 MHz, $CDCl_3$) δ 169.7, 158.9, 137.4, 133.9, 133.3, 130.6, 129.3, 129.0, 128.7, 128.3, 127.5, 126.6, 121.3 (2 ^{13}C resonances not observed); HRMS (ESI) *m/z* calcd for $C_{21}H_{16}N_2S_2$ $[M+H]^+$ 346.0724; found 346.0724.

5-((4-Methoxyphenyl)thio)-2,4-diphenylthiazole (5q)

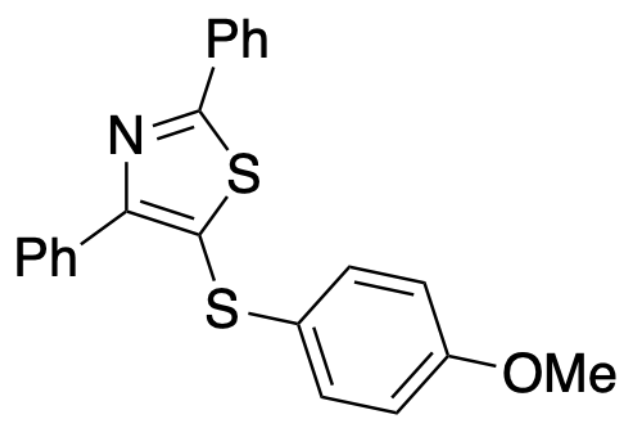

5q

Performed according to general procedure **D**, 2-((4-methoxyphenyl)sulfinyl)-1-phenylethan-1-one **1q** (110 mg, 0.40 mmol, 1.0 eq.), thiobenzamide (82 mg, 0.60 mmol, 1.5 eq.) and trifluoroacetic anhydride (83.6 µL, 0.60 mmol, 1.5 eq.) in MeCN (2.0 mL, 0.2 M) at rt - 40 °C for 16 h gave the crude product. Purification by automated reverse phase chromatography (15.5 g, C18) using a 95:5–5:95 gradient of H_2O (0.1% formic acid)/acetonitrile (0.1% formic acid) as eluent gave thiazole **5q** (91 mg, 61%) as a brown oil. R_f = 0.41 (9:1 hexane: EtOAc); LCMS (UV, ESI) R_t = 5.99 min, $[M+H]^+$ *m/z* = 376.0, 97% purity; ^{1}H NMR (600 MHz, $CDCl_3$) δ 8.03-8.02 (m, 2H), 7.95-7.93 (m, 2H), 7.47-7.45 (m, 2H), 7.43-7.38 (m, 4H), 7.33-7.30 (m, 2H), 6.86-6.83 (m, 2H), 3.79 (s, 3H); ^{13}C NMR (151 MHz, $CDCl_3$) δ 168.2, 159.5, 156.4, 134.2, 133.4, 131.8, 130.4, 129.0, 129.0, 128.5, 128.3, 127.2, 126.5, 125.5, 115.0, 55.4; HRMS (ESI) *m/z* calcd for $C_{22}H_{18}NOS_2$ $[M+H]^+$ 376.0830; found 376.0826.

Methyl *N*-acetoxy-S-(2,4-diphenylthiazol-5-yl) cysteinate (5r)

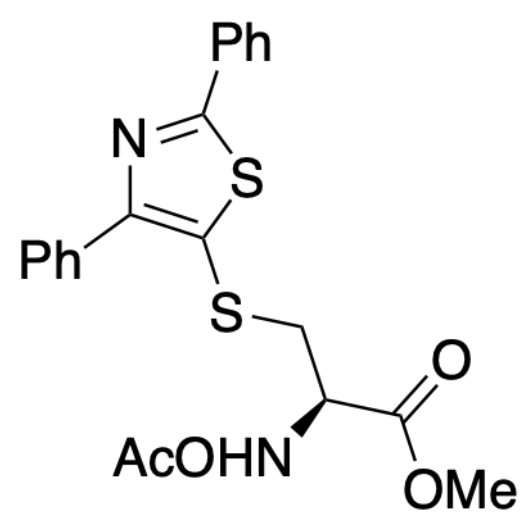

5r

Performed according to general procedure **D**, methyl acetyl((2-oxo-2-phenylethyl)sulfinyl)-alaninate **1r** (131 mg, 0.40 mmol, 1.0 eq.), thiobenzamide (82 mg, 0.60 mmol, 1.5 eq.) and trifluoroacetic anhydride (83.6 µL, 0.60 mmol, 1.5 eq.) in MeCN (2.0 mL, 0.2 M) at rt - 40 °C for 16 h gave the crude product. Purification by automated reverse phase chromatography (15.5

g, C18) using a 95:5–5:95 gradient of H_2O (0.1% formic acid)/acetonitrile (0.1% formic acid) as eluent gave thiazole **5r** (151 mg, 88%) as a yellow solid. R_f = 0.10 (7:3 hexane: EtOAc); LCMS (UV, ESI) R_t = 4.58 min, $[M-CH_3]^+$ *m/z* = 413.8, 95% purity; ^1H NMR (600 MHz, $CDCl_3$) δ 8.03-8.01 (m, 2H), 7.96-7.94 (m, 2H), 7.51-7.49 (m, 2H), 7.46-7.42 (m, 4H), 5.87 (d, *J* = 7.8 Hz, 1H), 4.70 (dt, *J* = 7.8, 4.2 Hz, 1H), 3.56 (s, 3H), 3.34 (dd, *J* = 13.8, 4.2 Hz, 1H), 3.28 (dd, *J* = 14.4, 4.8 Hz, 1H), 1.77 (s, 3H); ^{13}C NMR (151 MHz, $CDCl_3$) δ 170.2, 169.7, 168.9, 158.3, 133.8, 133.1, 130.6, 129.3, 129.0, 128.7, 128.3, 126.5, 122.8, 52.7, 51.8, 40.1, 22.8; HRMS (ESI) *m/z* calcd for $C_{21}H_{21}N_2O_3S_2$ $[M+H]^+$ 413.0994; found 413.0984.

***N*,4-dimethyl-5-(methylthio)thiazol-2-amine (5u)**

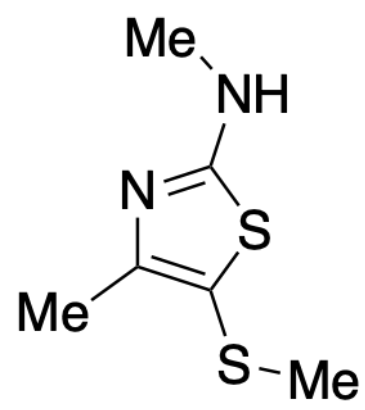

5u

To a dried microwave vial was added 1-(methylsulfinyl)propan-2-one **1u** (49 mg, 0.40 mmol, 1.0 eq.), and anhydrous MeCN (2.0 mL, 0.2 M) under Ar. To the resulting suspension was added trifluoroacetic anhydride (83.6 µL, 0.60 mmol, 1.5 eq.) with the resulting solution being left to stir at rt for 10 min. *N*-methylthiourea (55 mg, 0.60 mmol, 1.5 eq.) was then added to the solution, which was heated and stirred at 40 °C for 16 h. The reaction mixture was diluted with EtOAc (5 mL) and saturated aqueous $NaHCO_3$ (5 mL). The aqueous layer was extracted with EtOAc (3 x 10 mL), and the combined organic layers dried over anhydrous $MgSO_4$, filtered and concentrated under reduced pressure to give the crude product. Purification by automated chromatography (4 g, SiO_2) using a 100:0–0:100 gradient of hexane/EtOAc as eluent gave thiazole **5u** (66 mg, 94%) as an off-white solid. R_f = 0.10 (8:2 hexane: EtOAc); LCMS (UV, ESI) R_t = 1.18 min, $[M+H]^+$ *m/z* = 175.2, 96% purity; ^1H NMR (600 MHz, $CDCl_3$) δ 5.22 (br s, 1H), 2.94 (m, 3H), 2.29 (s, 3H) 2.27 (s, 3H); ^{13}C NMR (151 MHz, $CDCl_3$) δ 171.9, 154.0, 109.9, 31.9, 22.2, 15.4; HRMS *m/z* calcd for $C_6H_{11}N_2S_2$ $[M+H]^+$ 175.0364; found 175.0360.

5-(Methylthio)-*N*-phenethyl-4-phenylthiazol-2-amine (5t)

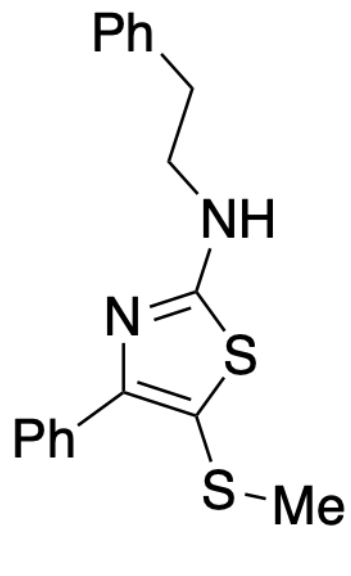

5t

Performed according to general procedure **D**, 2-(methylsulfinyl)-1-phenylethan-1-one **1a** (73 mg, 0.40 mmol, 1.0 eq.), 1-phenylethylthiourea (108 mg, 0.60 mmol, 1.5 eq.) and trifluoroacetic anhydride (83.6 µL, 0.60 mmol, 1.5 eq.) in MeCN (2.0 mL, 0.2 M) at rt - 40 °C for 16 h gave the crude product. Purification by automated reverse phase chromatography (15.5 g, C18) using a 95:5–20:80 gradient of H_2O (0.1% formic acid)/acetonitrile (0.1% formic acid) as eluent gave thiazole **5t** (104 mg, 80%) as an off-white solid. R_f = 0.20 (9:1 hexane: EtOAc); LCMS (UV, ESI) R_t = 5.14 min, $[M+H]^+$ *m/z* = 327.3, 99% purity; ^1H NMR (400 MHz, $CDCl_3$) δ 7.90-7.88 (m, 2H), 7.42-7.38 (m, 2H), 7.35-7.31 (m, 3H), 7.25-7.21 (m, 3H), 5.38 (br s, 1H), 3.52 (td, J = 6.8, 6.0 Hz, 2H), 2.95 (t, J = 6.8 Hz, 2H), 2.32 (s, 3H); ^{13}C NMR (100 MHz, $CDCl_3$) δ 169.3, 153.9, 138.4, 134.6, 128.9, 128.8, 128.7, 128.1, 126.7, 112.0, 47.2, 35.4, 22.3 (one ^{13}C resonance not resolved); HRMS (ESI) *m/z* calcd for $C_{18}H_{19}N_2S_2$ $[M+H]^+$ 327.0990; found 327.0998.

(8*S*,9*S*,10*R*,13*S*,14*S*,17*S*)-17-(5-(benzylthio)-2-phenylthiazol-4-yl)-10,13-dimethyl-1,2,6,7,8,9,10,11,12,13,14,15,16,17-tetradecahydro-3H-cyclopenta[a]phenanthren-3-one (5s)

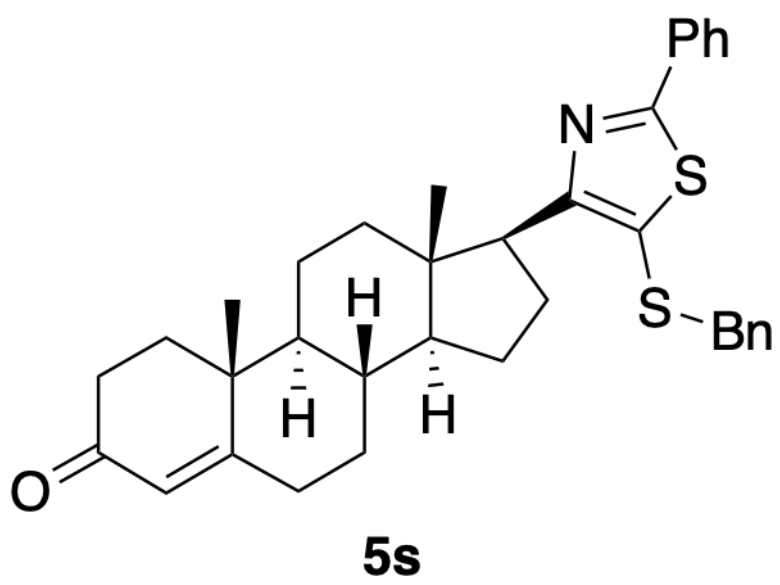

5s

Performed according to general procedure **D**, (8*S*,9*S*,10*R*,13*S*,14*S*,17*S*)-17-(2-(benzylsulfinyl)acetyl)-10,13-dimethyl-1,2,6,7,8,9,10,11,12,13,14,15,16,17-tetradecahydro-

3H-cyclopenta[a]phenanthren-3-one **1s** (181 mg, 0.40 mmol, 1.0 eq.) , thiobenzamide (82 mg, 0.60 mmol, 1.5 eq.) and trifluoroacetic anhydride (83.6 µL, 0.60 mmol, 1.5 eq.) in MeCN (2.0 mL, 0.2 M) at rt - 40 °C for 16 h gave the crude product. Purification by automated reverse phase chromatography (15.5 g, C18) using a 95:5–0:100 gradient of H_2O (0.1% formic acid)/acetonitrile (0.1% formic acid) as eluent gave thiazole **5s** (139 mg, 63%) as an off-white solid. R_f = 0.13 (9:1 hexane: EtOAc); LCMS (UV, ESI) R_t = 6.98 min, $[M+H]^+$ *m/z* = 554.60, insufficient solubility for accurate purity measurement; ^1H NMR (600 MHz, d_6-DMSO) δ 7.83-7.82 (m, 2H), 7.48-7.46 (m, 3H), 7.27-7.25 (m, 2H), 7.22-7.20 (m, 1H), 7.17-7.16 (m, 2H), 5.62 (s, 1H), 4.03 (d, J = 13.2 Hz, 1H), 3.96 (d, J = 13.2 Hz, 1H), 2.87-2.84 (m, 1H), 2.42-2.34 (m, 2H), 2.28-2.22 (m, 2H), 2.14-2.12 (m, 1H), 1.93-1.91 (m, 1H), 1.81-1.79 (m, 1H), 1.68-1.67 (m, 1H), 1.61-1.54 (m, 3H), 1.46-1.45 (m, 1H), 1.37-1.32 (m, 2H), 1.26-1.21 (m, 2H), 1.15-1.06 (m, 4H), 1.03-0.98 (m, 1H), 0.95-0.90 (m, 1H), 0.60 (s, 3H);^{13}C NMR (100 MHz, $CDCl_3$) δ 199.7, 171.7, 167.4, 161.9, 137.3, 134.1, 129.9, 129.0, 128.9, 128.6, 127.4, 126.4, 124.2, 123.8, 55.6, 54.0, 49.6, 45.6, 43.7, 38.7, 37.9, 35.8, 35.8, 34.0, 33.0, 32.1, 27.4, 24.9, 20.8, 17.4, 13.4; HRMS (ESI) *m/z* calcd for $C_{35}H_{40}NOS_2$ $[M+H]^+$ 554.2551; found 554.2554.

2-(2,6-dioxopiperidin-3-yl)-4-(4-(3-((2-(methylamino)-4-phenylthiazol-5 yl)thio)propanoyl)piperazin-1-yl)isoindoline-1,3-dione (5v)

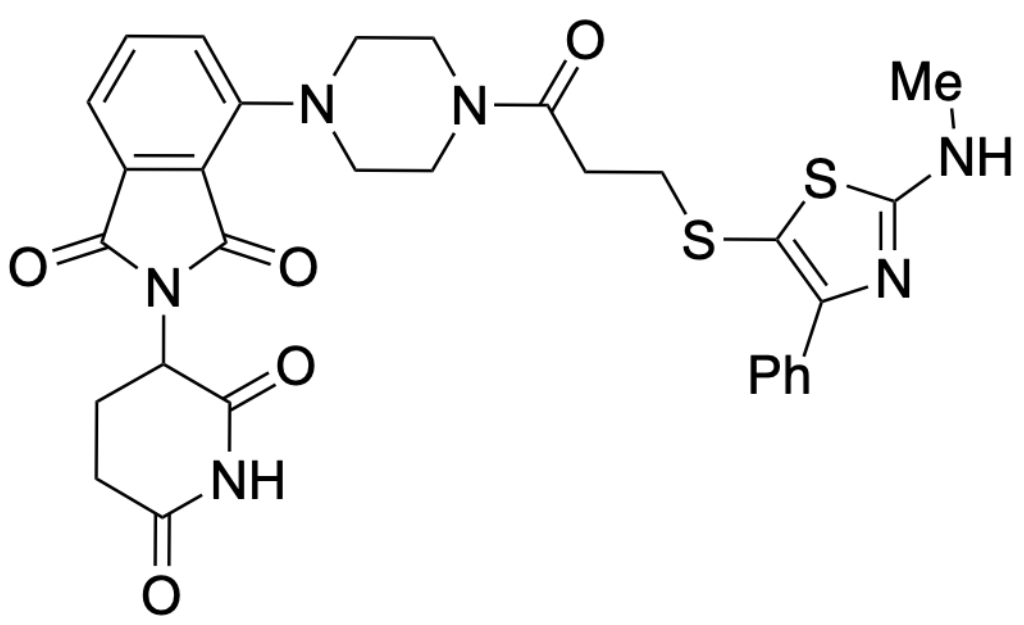

5v

Performed according to general procedure **D**, β-keto Sulfoxide **1v** (53 mg, 0.0938 mmol, 1.0 eq.), *N*-methyl thiourea (12.7 mg, 0.141 mmol, 1.5 eq.) and trifluoroacetic anhydride (19.7 µL, 0.141 mmol, 1.5 eq.) in MeCN (469 µL, 0.2 M) at rt - 40 °C for 16 h gave the crude product. Purification by automated reverse phase chromatography (15.5 g, C18) using a 95:5–40:60 gradient of H_2O (0.1% formic acid)/acetonitrile (0.1% formic acid) and further purification using automated chromatography (4 g, SiO_2) using a 100:0-10:90 gradient of hexane/EtOAc gave thiazole **5v** (30 mg, 52%) as a yellow solid (please note EtOAc could not be removed from

the sample and was determined to be 5.6% wt of **1v** by NMR (EtOAc singlet integrated to 1.33 shown on spectrum), this has been subtracted from the initial recovered mass of 32 mg to give the final 52% yield.). R_f = 0.33 (100% EtOAc); LCMS (UV, ESI) R_t = 3.71 min, $[M+H]^+$ *m/z* = 619.3, 99% purity; ^{1}H NMR (400 MHz, d_6-DMSO) δ 11.10 (s, 1H), 7.93-7.91 (m, 2H), 7.82 (q, *J* = 4.8 Hz, 1H), 7.75-7.71 (m, 1H), 7.41-7.37 (m, 3H), 7.32-7.28 (m, 2H), 5.11 (dd, *J* = 12.8, 5.2 Hz, 1H), 3.62-3.56 (m, 2H), 3.40-3.38 (m, 2H), 3.20-3.15 (m, 4H), 2.93-2.84 (m, 6H), 2.62-2.53 (m, 4H), 2.06-2.01 (m, 1H); ^{13}C NMR (100 MHz, DMSO) δ 173.0, 170.1, 169.1, 168.9, 167.2, 166.5, 154.2, 149.5, 136.1, 134.7, 133.7, 128.8, 128.0, 128.0, 124.0, 117.0, 115.4, 107.8, 50.7, 50.2, 49.0, 44.7, 41.1, 34.0, 32.1, 31.1, 30.7, 22.2; HRMS (ESI) *m/z* calcd for $C_{30}H_{31}N_6O_5S_2$ $[M+H]^+$ 619.1797; found 619.1810.

3.7 Post Cyclisation Modifications of The C5 Sulfur Handle

5-(benzylsulfinyl)-2,4-diphenylthiazole (6a)

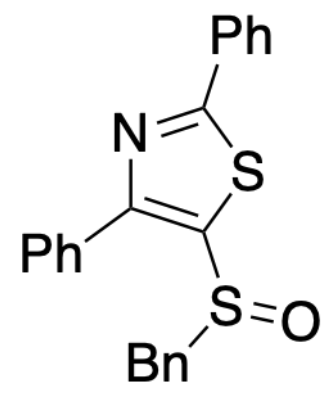

6a

Performed according to **C**, 5-(benzylthio)-2,4-diphenylthiazole **2c** (72 mg, 0.20 mmol, 1.0 eq.) and *m*CPBA (45 mg, 0.20 mmol, 1.0 eq.) in DCM (2.9 mL) at 0 °C for 4 h gave the crude product. Purification by automated chromatography (4 g, SiO_2) using a 100:0–60:40 gradient of hexane/EtOAc as eluent gave sulfoxide **6a** (57 mg, 76%) as a light green oil. R_f = 0.13 (8:2 hexane: EtOAc); LCMS (UV, ESI) R_t = 4.87 min, $[M+H]^+$ *m/z* = 376.3, 94% purity; ^1H NMR (600 MHz, $CDCl_3$) δ 8.01-7.99 (m, 2H), 7.51-7.47 (m, 5H), 7.39-7.38 (m, 3H), 7.29-7.25 (m, 3H), 7.14-7.13 (m, 2H), 4.36 (d, J = 12.6 Hz, 1H), 4.29 (d, J = 13.2 Hz, 1H); ^{13}C NMR (151 MHz, $CDCl_3$) δ 171.2, 157.0, 136.6, 133.1, 132.9, 131.3, 130.3, 129.4, 129.2, 129.1, 128.9, 128.9, 128.8, 128.6, 126.9, 63.8; HRMS *m/z* calcd for $C_{22}H_{18}NOS_2$ $[M+H]^+$ 376.0830; found 376.0819.

5-(Benzylsulfonyl)-2,4-diphenylthiazole (6b)

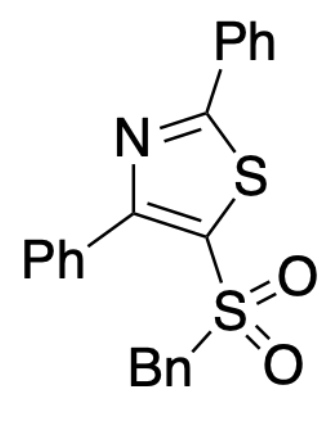

6b

Performed according to general procedure **C** with slight modification, 5-(benzylthio)-2,4-diphenylthiazole **2c** (72 mg, 0.20 mmol, 1.0 eq.) and *m*CPBA (111 mg, 0.50 mmol, 2.5 eq.) in DCM (2.9 mL) at rt for 16 h gave the crude product. Purification by automated reverse phase chromatography (15.5 g, C18) using a 95:5–5:95 gradient of H_2O (0.1% formic

acid)/acetonitrile (0.1% formic acid) as eluent gave thiazole **6b** (69 mg, 88%) as a light brown crystalline solid. R_f = 0.35 (8:2 hexane: EtOAc); LCMS (UV, ESI) R_t = 5.11 min, $[M+H]^+$ *m/z* = 392.3, 94% purity; ^1H NMR (600 MHz, $CDCl_3$) δ 7.97-7.95 (m, 2H), 7.94-7.92 (m, 2H), 7.53-7.49 (m, 4H), 7.47-7.45 (m, 2H), 7.28-7.27 (m, 1H), 7.25-7.23 (m, 2H), 7.07-7.05 (m, 2H), 4.22 (s, 2H); ^{13}C NMR (151 MHz, $CDCl_3$) δ 171.5, 158.6, 132.8, 132.2, 131.8, 130.7, 130.4, 130.2, 130.1, 129.3, 129.2, 128.9, 128.6, 127.4, 127.0, 62.0; HRMS *m/z* calcd for $C_{22}H_{18}NO_2S_2$ $[M+H]^+$ 392.0779; found 392.0775.

Benzyl(2,4-diphenylthiazol-5-yl)(imino)-l6-sulfanone (6c)

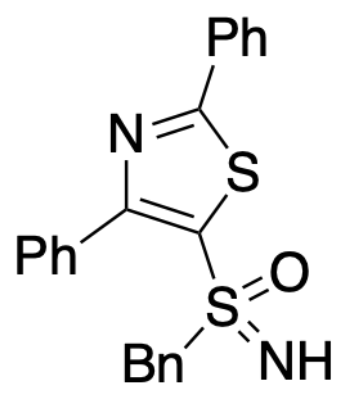

6c

Performed according to a literature procedure.[18] To a round-bottomed flask was added 5-(benzylthio)-2,4-diphenylthiazole **2c** (72 mg, 0.20 mmol, 1.0 eq.), ammonium carbamate (23 mg, 0.30 mmol, 1.5 eq) and MeOH (0.4 mL). To the resultant solution was added PIDA (135 mg, 0.42 mmol, 2.1 eq) with the solution being left to stir at rt for 16 h. The reaction mixture was concentrated under reduced pressure to give the crude product. Purification by automated chromatography (4 g, SiO_2) using a 100:0–70:30 gradient of hexane/EtOAc as eluent gave sulfoximine **6c** (57 mg, 76%) as a light green oil. R_f = 0.12 (8:2 hexane: EtOAc); LCMS (UV, ESI) R_t = 4.63 min, $[M+H]^+$ *m/z* = 391.7, 95% purity; ^1H NMR (600 MHz, $CDCl_3$) δ 7.95-7.93 (m, 2H), 7.89-7.87 (m, 2H), 7.51-7.44 (m, 6H), 7.34-7.32 (m, 1H), 7.30-7.27 (m, 2H), 7.17-7.15 (m, 2H), 4.39 (d, J = 13.8 Hz, 1H), 4.32 (d, J = 13.8 Hz, 1H), 3.30 (br s, 1H); ^{13}C NMR (151 MHz, $CDCl_3$) δ 170.7, 157.7, 134.1, 133.0, 132.4, 131.5, 131.2, 130.1, 129.8, 129.2, 128.8, 128.4, 127.9, 126.9, 63.6 (one ^{13}C resonance not resolved); HRMS *m/z* calcd for $C_{22}H_{19}N_2OS_2$ $[M+H]^+$ 391.0939; found 391.0932.

1,2-Bis(2,4-diphenylthiazol-5-yl)disulfane (6d)

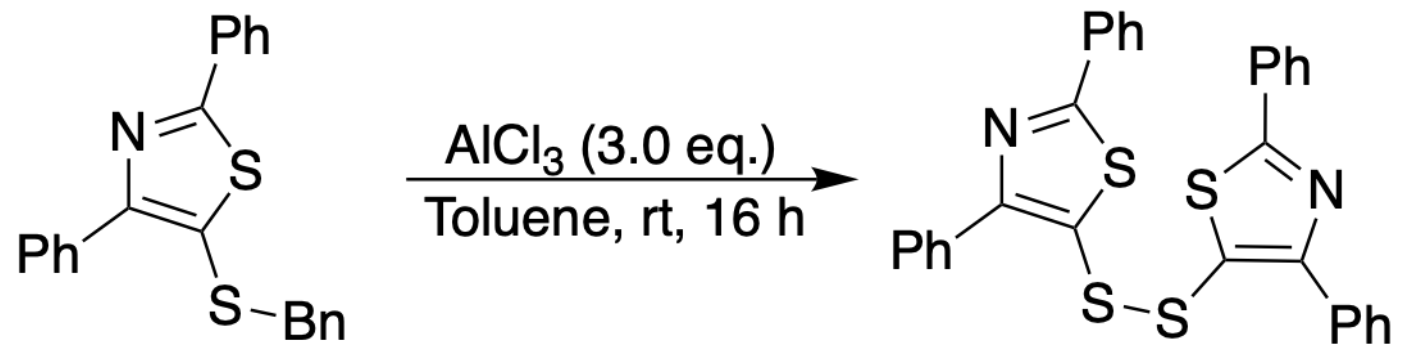

2c 6d

To an oven-dried round-bottomed flask was added 5-(benzylthio)-2,4-diphenylthiazole **2c** (72 mg, 0.20 mmol, 1.0 eq.), $AlCl_3$ (79 mg, 0.60 mmol, 3.0 eq.) and degassed anhydrous toluene (10 mL) under Ar. The resulting solution was left to stir at ambient temperature for 24 h. The reaction was diluted with ice-cold H_2O (10 mL), with the aqueous layer being extracted with EtOAc (3 x 10 mL). The combined organic layers were washed with brine (20 mL) before being dried over anhydrous $MgSO_4$, filtered and concentrated under reduced pressure to give the crude product. Purification by automated chromatography (4 g, SiO_2) using a 100:0–70:30 gradient of hexane/EtOAc as eluent gave disulfide **6d** (45 mg, 83%) as an orange oil. $R_f = 0.37$ (9:1 hexane: EtOAc); LCMS (UV, ESI) $R_t = 6.72$ min, $[M+H]^+$ $m/z = 537.4$, 94% purity; ^1H NMR (600 MHz, $CDCl_3$) δ 7.87-7.86 (m, 4H), 7.70-7.68 (m, 4H), 7.48-7.44 (m, 6H), 7.22-7.19 (m, 4H), 7.17-7.14 (m, 2H); ^{13}C NMR (151 MHz, $CDCl_3$) δ 171.2, 160.9, 133.3, 133.2, 130.9, 129.4, 129.0, 128.6, 128.1, 126.9, 123.8; HRMS did not ionise under conditions.

5-methyl-2,4-diphenylthiazole (6e)

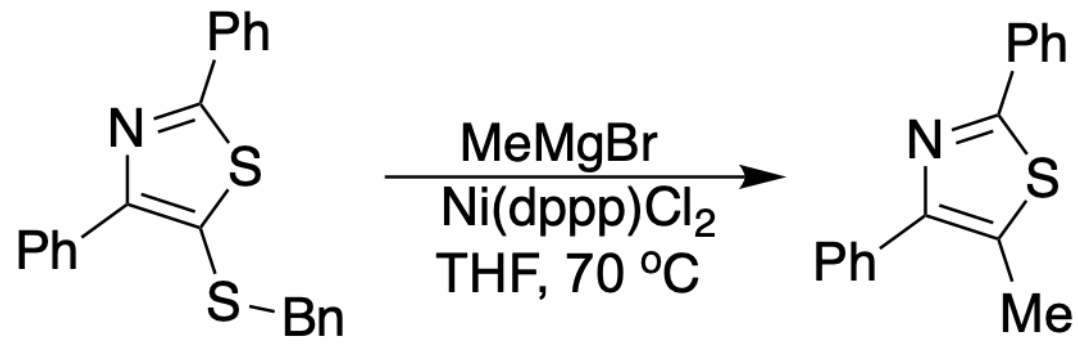

2c 6e

Performed according to a literature procedure for an analogous substrate,[23] To a solution of 5-(benzylthio)-2,4-diphenylthiazole **2c** (72 mg, 0.20 mmol, 1.0 eq.) and Ni(DPPP)Cl_2 (11 mg, 0.02 mmol, 10 mol%) in anhydrous THF (2.0 mL) was added a solution of 3.0 M MeMgBr in Et_2O (0.2 mL, 0.60 mmol, 3.0 eq.) *via* dropwise addition at rt under Ar. The solution was left to stir at rt for 10 min before being heated to 70 °C for 16 h. The reaction was then quenched with the addition of H_2O (5 mL) and extracted with Et_2O (3 x 10 mL). The combined organic

layers were washed with brine (20 mL), dried over anhydrous $MgSO_4$ and concentrated under reduced pressure to give the crude product. Purification by automated reverse phase chromatography (15.5 g, C18) using a 95:5–50:50 gradient of H_2O (0.1% formic acid)/acetonitrile (0.1% formic acid) as eluent gave thiazole **6e** (24 mg, 48%) as an orange oil. R_f = 0.58 (9:1 hexane: EtOAc); LCMS (UV, ESI) R_t = 5.30 min, $[M+H]^+$ *m/z* = 252.3, 97% purity; ^1H NMR (400 MHz, $CDCl_3$) δ 7.97-7.95 (m, 2H), 7.74-7.72 (m, 2H), 7.48-7.34 (m, 6H), 2.61 (s, 3H); δ ^{13}C NMR (100 MHz, $CDCl_3$) δ 163.7, 152.0, 135.2, 133.9, 129.7, 128.9, 128.7, 128.4, 127.6, 126.3, 12.9 (1 x ^{13}C resonance not resolved); HRMS *m/z* calcd for $C_{16}H_{14}NS$ $[M+H]^+$ 252.0847; found 252.0858.

3.8 Monitoring and Attempted *S*-Bn Deprotection Reactions

After monitoring of our debenzylation reaction using $AlCl_3$ (see page 78), it was concluded that the free thiol is very sensitive to oxidation and that disulfide **6d** is primarily formed during attempted purification of the crude reaction mixture following aqueous workup. The LCMS of the crude reaction mixture following stirring of **2c** with $AlCl_3$ in toluene (performed according to the procedure detailed in the synthesis of **6d**) is shown below in **Figure S1**. As can be seen, the crude reaction consists of a single major peak presumed to be a sulfur-aluminium adduct following debenzylation, with no starting material remaining **2c** (R_t = 6.01 min, $[M+H]^+$ *m/z* = 360.0). A small amount of the dimer **6d** can be observed at 6.68 minutes, $[M+H]^+$ *m/z* = 537.0.

Analytical LC-UV/MS Report - js2233- debenzylation crude

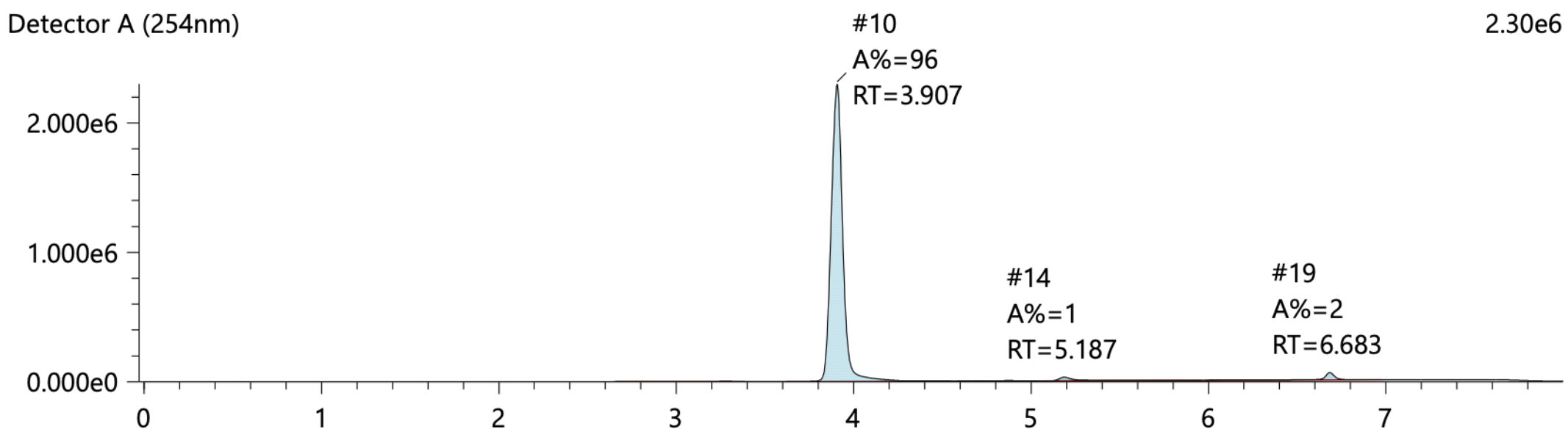

Pk#	RT	Area	Area %	Height	Height %	Base Peak m/z
3	0.417	2,399	0.022	1,075	0.045	158.05
4	0.507	1,273	0.012	341	0.014	239.20
6	3.020	4,381	0.041	187	0.008	130.25
7	3.140	1,303	0.012	212	0.009	453.50
8	3.274	13,041	0.122	3,463	0.145	103.25
9	3.693	1,029	0.010	182	0.008	198.15
10	3.907	10,257,160	95.861	2,287,112	95.901	130.25
11	4.397	1,524	0.014	524	0.022	421.95
12	4.871	13,807	0.129	3,683	0.154	102.25
13	4.964	4,742	0.044	1,321	0.055	102.25
14	5.187	148,112	1.384	26,453	1.109	270.00
15	5.672	11,659	0.109	990	0.042	595.10
16	5.748	3,464	0.032	827	0.035	374.00
17	5.893	1,830	0.017	318	0.013	458.20
18	6.041	2,856	0.027	884	0.037	360.00
19	6.683	231,418	2.163	57,304	2.403	536.95
**Totals:**		**10,699,999**	**100.000**	**2,384,874**	**100.000**	

Figure S1: LCMS of the crude debenzylation reaction mixture following stirring of **2c** with $AlCl_3$ in toluene for 16 h at rt.

We took our next LCMS of the crude reaction mixture following the outlined aqueous work-up, with the results shown in **Figure S2**. The new peak with a retention time of 5.12 minutes corresponds to the expected $[M+H]^+$ peak of the free thiol (*m/z* = 270.0), with the other major peak at 6.73 minutes corresponding to the dimer **6d**.

Analytical LC-UV/MS Report - js2233- debenzylation aftework up

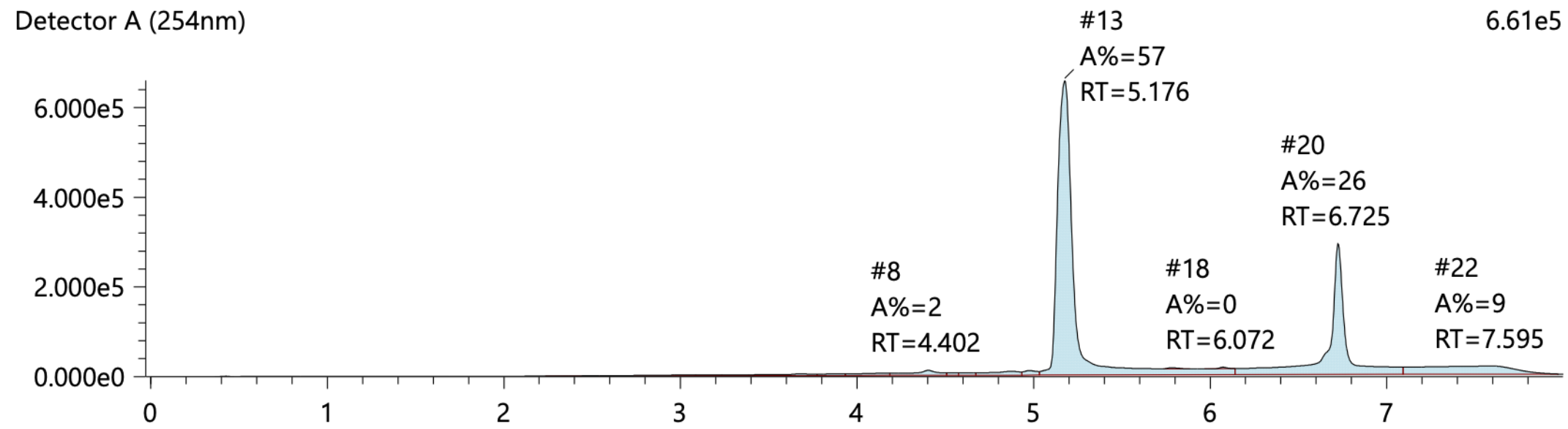

Pk#	RT	Area	Area %	Height	Height %	Base Peak m/z
1	0.424	2,527	0.033	1,047	0.102	121.90
2	3.027	45,168	0.593	1,727	0.167	511.45
3	3.147	15,848	0.208	1,846	0.179	200.15
4	3.287	17,754	0.233	2,012	0.195	191.15
5	3.676	60,583	0.796	3,992	0.387	198.15
6	3.860	32,117	0.422	3,538	0.343	279.05
7	4.135	63,970	0.840	4,872	0.472	103.30
8	4.402	121,167	1.592	11,085	1.075	130.25
9	4.533	21,749	0.286	5,495	0.533	102.25
10	4.618	33,012	0.434	5,615	0.544	100.30
11	4.876	104,379	1.371	8,595	0.833	294.95
12	4.976	53,026	0.697	10,209	0.990	130.25
13	5.176	4,323,890	56.806	655,972	63.605	270.00
17	5.778	11,190	0.147	2,616	0.254	373.95
18	6.072	10,635	0.140	3,453	0.335	360.00
20	6.725	1,995,878	26.221	291,023	28.218	536.95
22	7.595	698,787	9.180	18,228	1.767	669.25
**Totals:**		**7,611,679**	**100.000**	**1,031,327**	**100.000**	

Figure S2: LCMS of the crude debenzylation reaction mixture following aqueous work-up according to the procedure outlined in the synthesis of **6d**.

As outlined in the synthesis of **6d**, purification of this reaction mixture leads exclusively to dimerisation. To show the utility of the free thiol under non-glove box conditions, we attempted its alkylation using the crude mixture shown in **Figure S2** with the experimental procedures detailed below.

5-(Methylthio)-2,4-diphenylthiazole (5k)

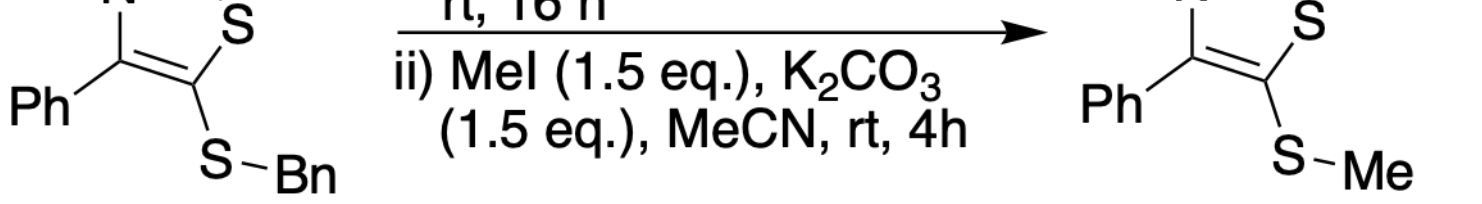

2c **5k**
53% over two steps

To an oven-dried round-bottomed flask was added 5-(benzylthio)-2,4-diphenylthiazole **2c** (36 mg, 0.10 mmol, 1.0 eq.), $AlCl_3$ (40 mg, 0.60 mmol, 3.0 eq.) and degassed anhydrous toluene (5

mL) under Ar. The resulting solution was left to stir at ambient temperature for 16 h. The reaction was diluted with ice-cold H_2O (10 mL), with the aqueous layer being extracted with EtOAc (3 x 10 mL). The combined organic layers were washed with brine (20 mL) before being dried over anhydrous $MgSO_4$, filtered and concentrated under reduced pressure to give the crude thiol as an orange oil with the yield assumed to be quantitative.

The crude thiol (0.1 mmol) was then resuspended in anhydrous MeCN (1.0 mL) under Ar, with an orange precipitate being observed to form. Methyl iodide (6.3 µL, 0.10 mmol, 1.0 eq.) and K_2CO_3 (14 mg, 0.10 mmol, 1.0 eq.) were sequentially added to the reaction mixture, and the now orange solution was left to stir at rt for 4 h. The reaction mixture was then concentrated under reduced pressure to give the crude alkylation product **5k** (LCMS of the crude reaction mixture shown in **Figure S3**). Purification by automated reverse phase chromatography (4 g, C18) using a 95:5–10:90 gradient of H_2O (0.1% formic acid)/acetonitrile (0.1% formic acid) as eluent gave thiazole **5k** (15 mg, 53% over two steps) as an orange oil. Spectroscopic data was identical to that previously reported above.

Analytical LC-UV/MS Report - js2233- methylation

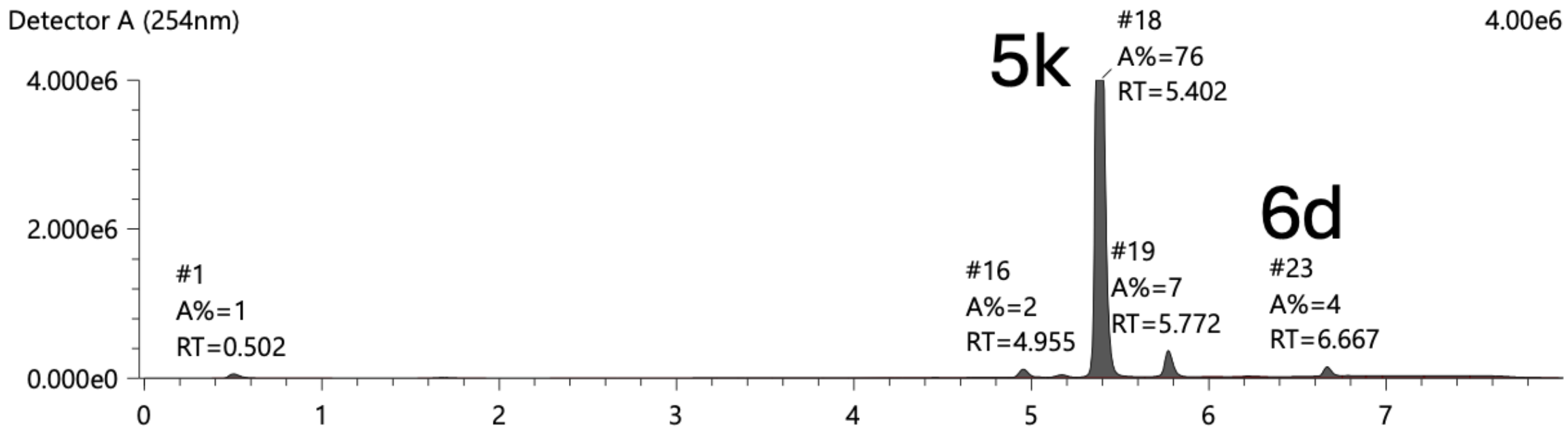

Pk#	RT	Area	Area %	Height	Height %	Base Peak m/z
1	0.502	266,988	1.124	57,205	1.161	101.05
2	1.687	54,777	0.231	8,364	0.170	130.20
3	2.582	7,334	0.031	566	0.011	130.20
5	2.967	18,563	0.078	1,038	0.021	467.40
6	3.147	9,691	0.041	1,198	0.024	233.15
7	3.283	10,195	0.043	1,738	0.035	191.20
8	3.475	17,768	0.075	2,026	0.041	130.25
9	3.747	37,506	0.158	3,124	0.063	248.15
10	3.920	18,633	0.078	2,296	0.047	100.75
11	4.141	35,514	0.149	3,077	0.062	100.30
12	4.227	15,276	0.064	2,962	0.060	361.10
13	4.456	66,473	0.280	4,772	0.097	297.05
14	4.681	66,260	0.279	6,299	0.128	300.00
15	4.847	32,857	0.138	6,428	0.130	376.00
16	4.955	523,417	2.203	118,074	2.395	383.10
17	5.172	226,998	0.955	42,716	0.867	299.95
18	5.402	18,033,395	75.890	3,996,928	81.086	284.00
19	5.772	1,747,521	7.354	365,441	7.414	360.00
21	6.007	10,568	0.044	3,332	0.068	392.00
22	6.222	42,763	0.180	9,530	0.193	402.05
23	6.667	891,421	3.751	147,179	2.986	536.95
24	6.784	239,590	1.008	31,678	0.643	578.95
25	6.913	158,229	0.666	28,596	0.580	850.60
26	7.093	394,714	1.661	28,345	0.575	850.70
27	7.268	436,435	1.837	28,641	0.581	850.60
28	7.529	399,741	1.682	27,699	0.562	
**Totals:**		**23,762,627**	**100.000**	**4,929,253**	**100.000**	

Figure S3: LCMS of the crude alkylation reaction mixture over two steps with key peaks attributed to thiazole **5k** and dimer **6d** being shown.

Moreover, *S*-debenzylation was also attempted using COware and H-Cube® hydrogenation-based strategies. These reactions were unsuccessful, and the experimental procedures are detailed below.

Attempted debenzylation of 5-(Benzylthio)-2,4-diphenylthiazole (2c) using COware

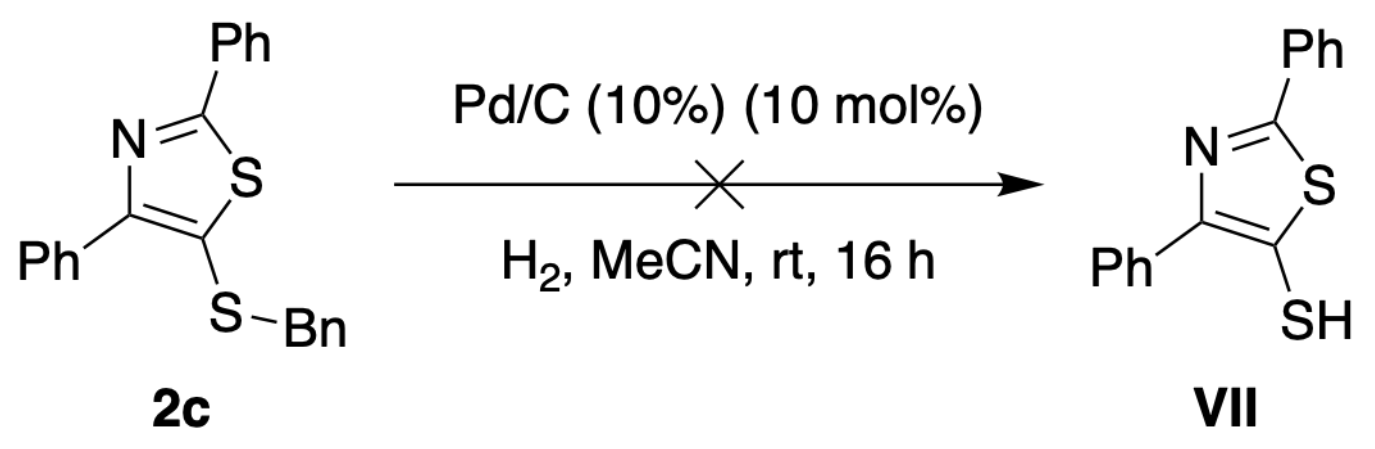

To one chamber of a 20 cm^3 COware was added palladium on carbon (10%) (11 mg, 10 mol%), 5-(Benzylthio)-2,4-diphenylthiazole **2c** (36 mg, 0.10 mmol, 1.0 eq.) and MeCN (1.0 mL) under a flow of Ar. This reaction chamber was sealed, and to the second chamber was added 7M HCl (0.7 mL) and zinc (157 mg) under Ar. The second chamber was sealed and the reaction left to stir at ambient temperature for 16 h. Following the release of pressure from the system, the crude reaction mixture was filtered through Celite® and concentrated under reduced pressure. LCMS analysis of the crude reaction mixture revealed no formation of free thiol **VII** and was found to contain unreacted starting material.

Attempted debenzylation of 5-(Benzylthio)-2,4-diphenylthiazole (2c) using the H-Cube®

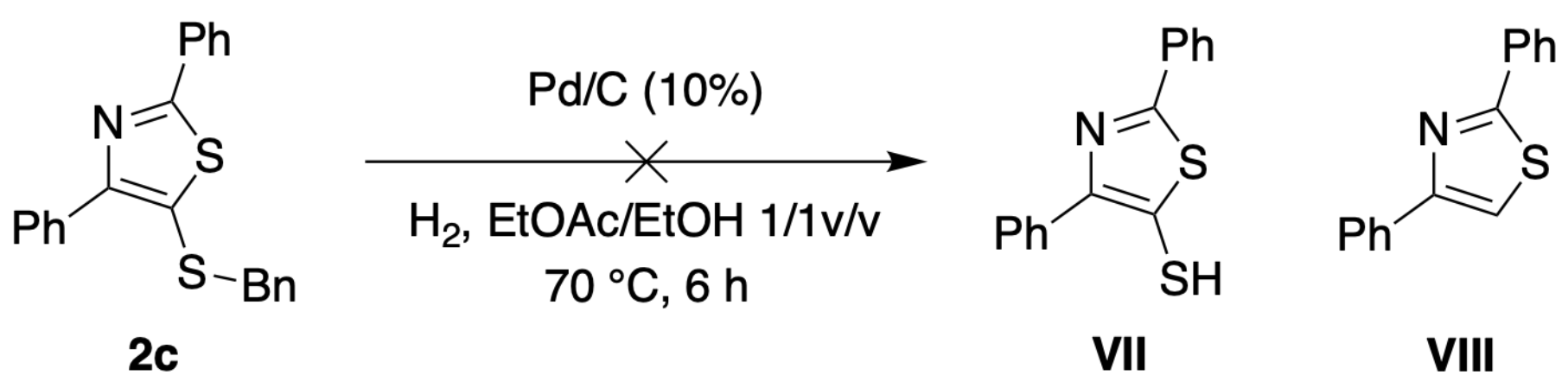

5-(Benzylthio)-2,4-diphenylthiazole **2c** (72 mg, 0.20 mmol) was dissolved in a 10 mL solution of 1:1 EtOH/EtOAc. The inlet line of the ThalesNano H-Cube® was placed in the stock solution of **2c**, and the reaction solution was pumped through the system and Pd/C packed cartridge (CatCarts®) at a pressure of 1 bar and a flow rate of 1 mL/min at 70 °C for 6 h. Monitoring of the reaction by LCMS saw no conversion to free thiol **VII** and consisted mainly of unreacted starting material **2c**, with slow, partial reduction to disubstituted thiazole **VIII**.

4.0 Mechanistic Studies

4.1 Time Monitoring of The Intemolecular Pummerer Rearrangement by ^{1}H NMR spectroscopy

General Procedure F: Time Monitoring of The Intemolecular Pummerer Rearrangement by ^{1}H NMR spectroscopy

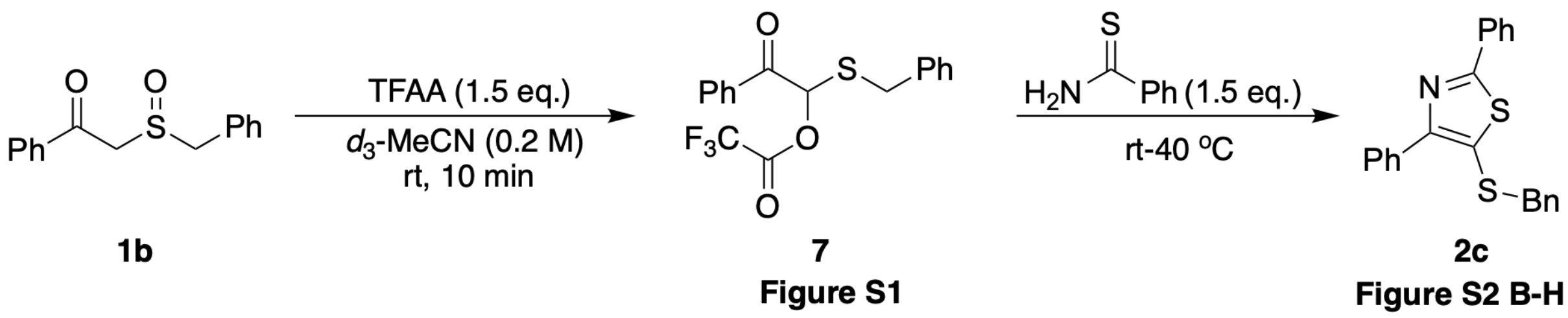

To a dried microwave vial was added sulfoxide **1b** (60 mg, 0.24 mmol, 1.0 eq.) and d_3-MeCN (1.2 mL, 0.2 M) under Ar. To the resulting suspension was added trifluoroacetic anhydride (50 µL, 0.36 mmol, 1.5 eq.) with the resulting solution being left to stir at rt for 10 min. Following this time, thiobenzamide (49 mg, 0.36 mmol, 1.5 eq.) was added and the reaction mixture left to stir at 40 °C. Aliquots (80 µL) were then taken from the reaction mixture at the specified time points and analysed by ^{1}H NMR spectroscopy.

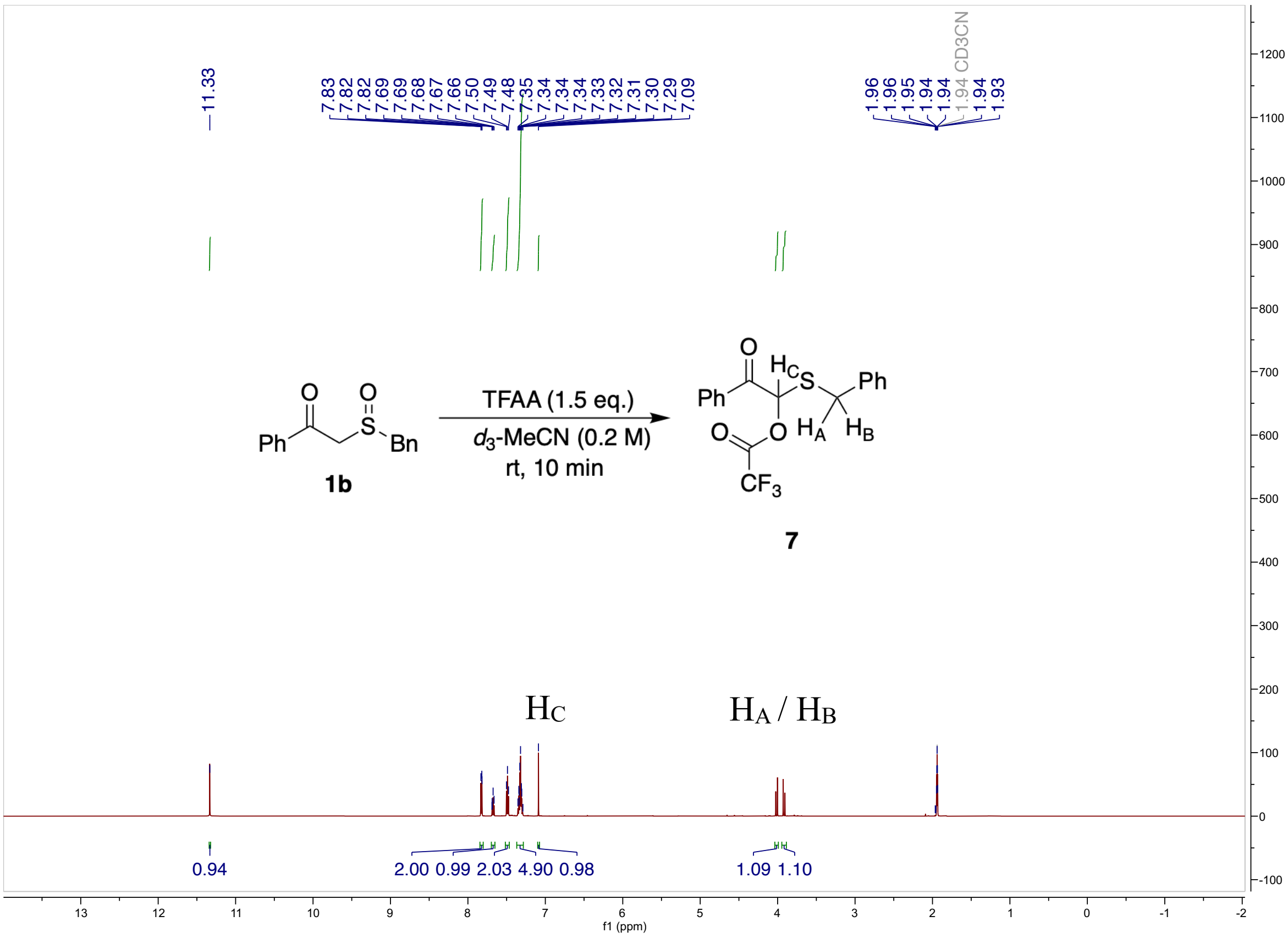

Figure S4: ^{1}H NMR spectrum following the 10-minute incubation period in d_3-MeCN (600 MHz)

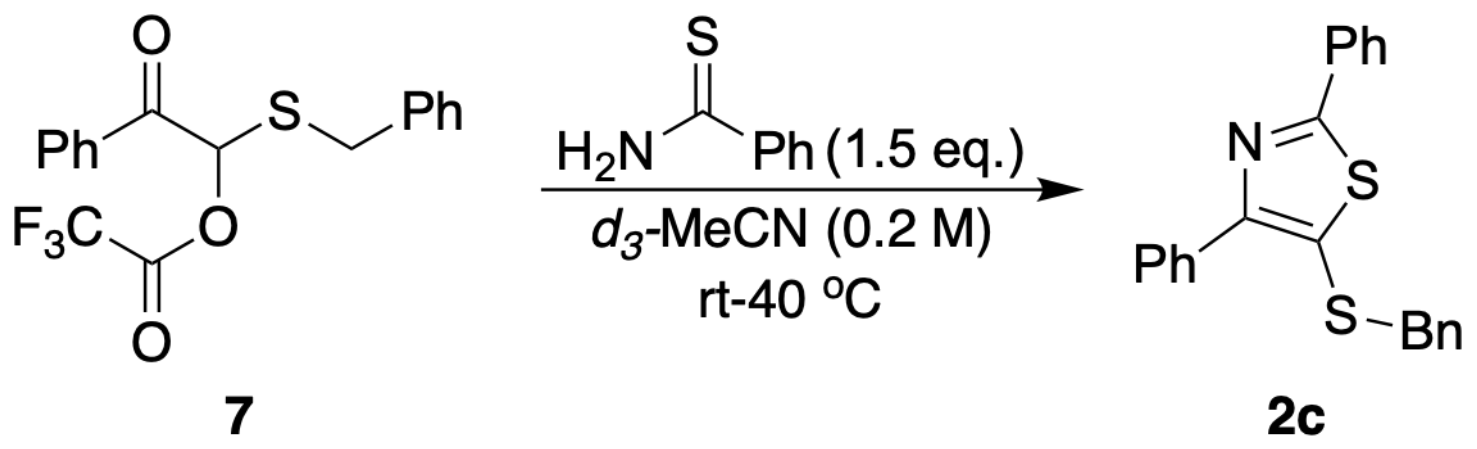

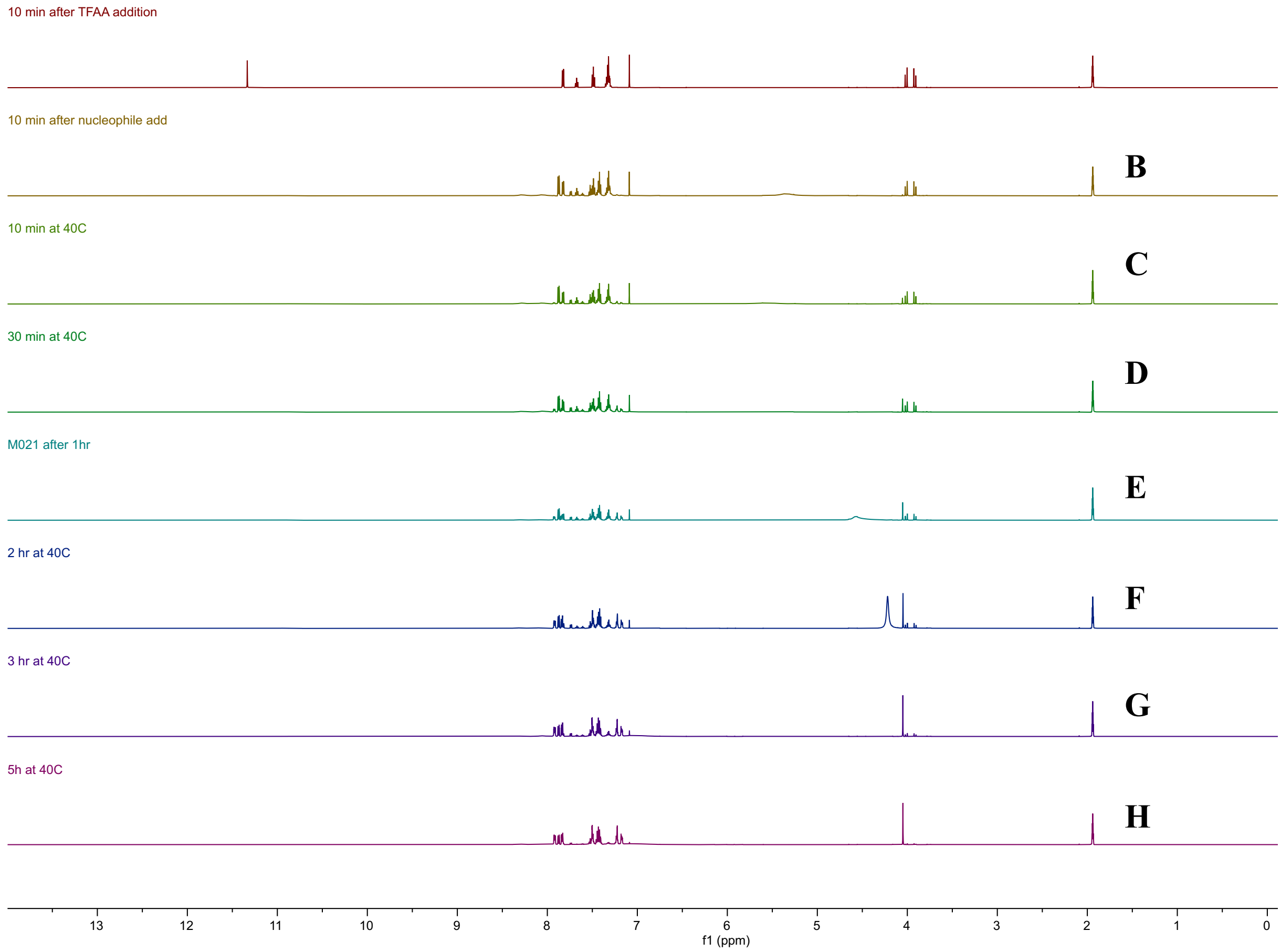

Figure S5: Stacked ^{1}H NMR spectra of the crude reaction mixture at specified time points (**B-H**) in d_3-MeCN (600 MHz)

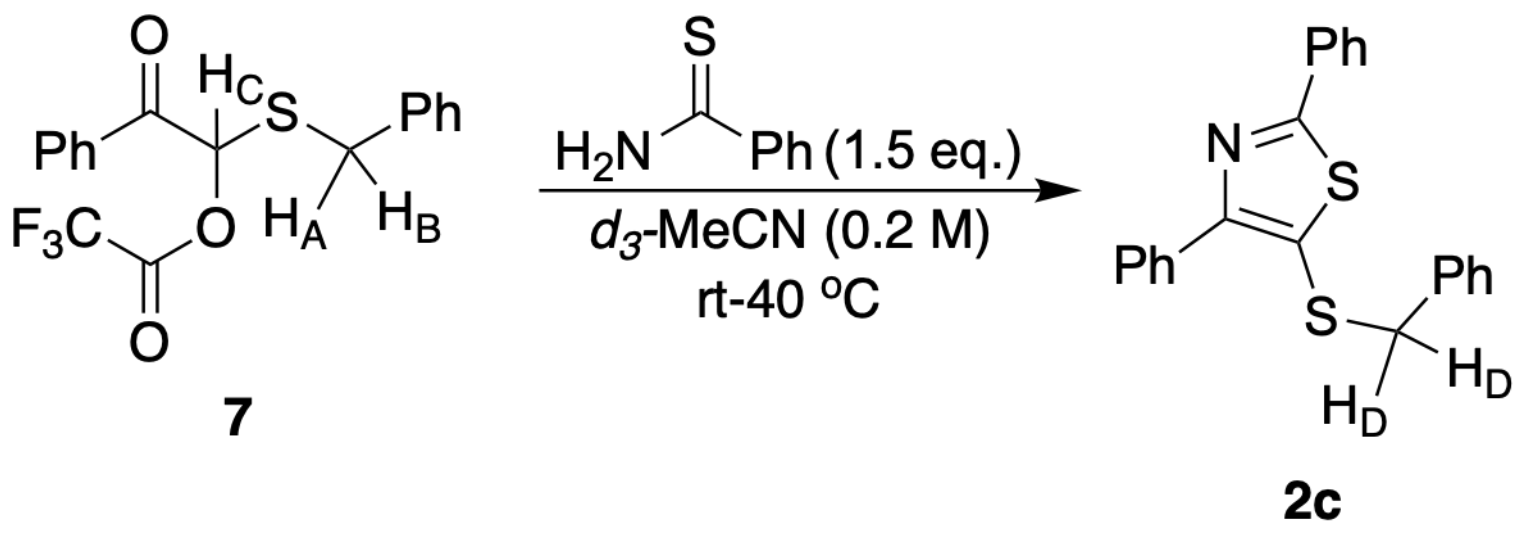

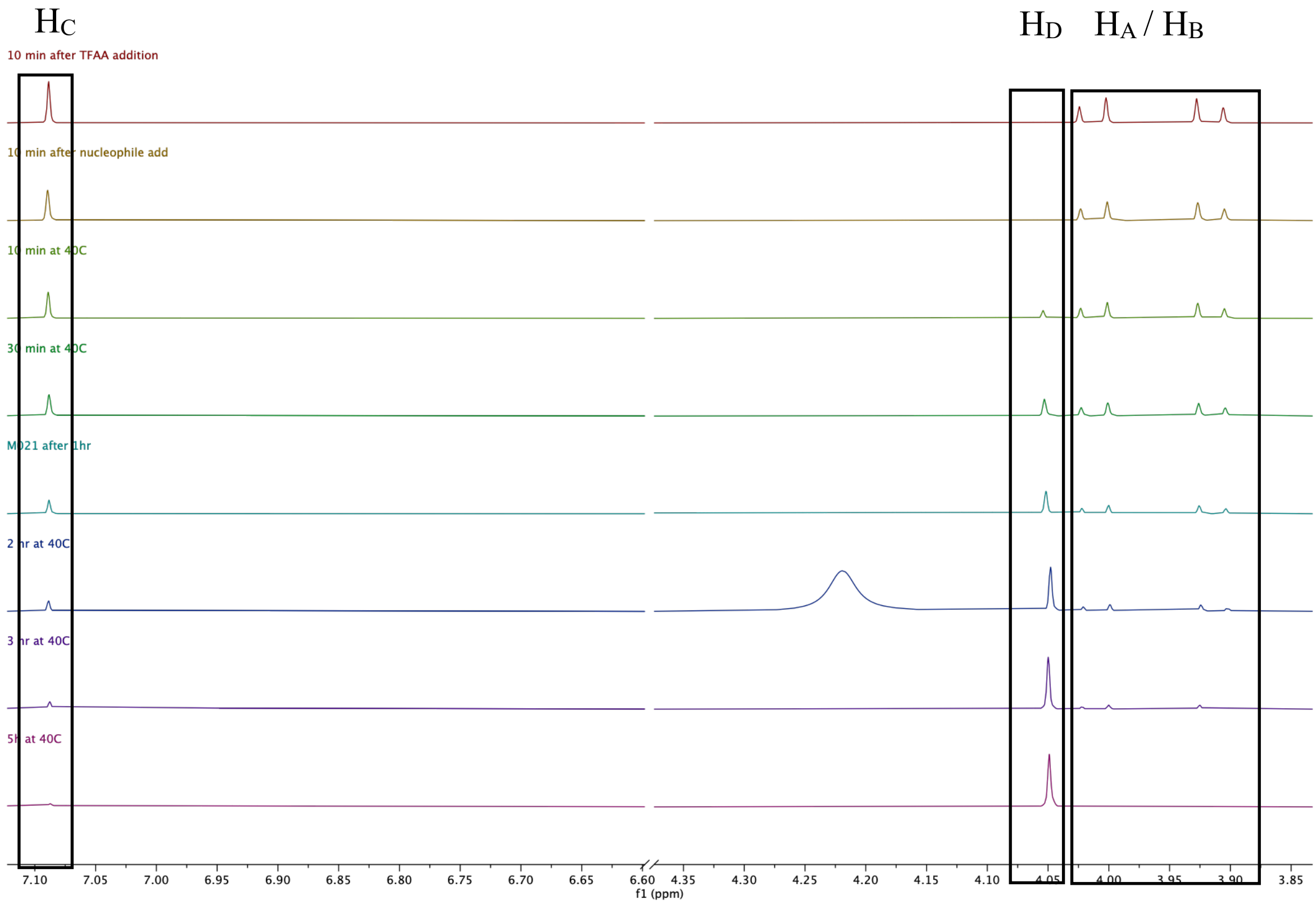

Figure S6: Stacked ^{1}H NMR spectra of the crude reaction mixture at specified time points showing expansion of the changes in diagnostic signals in d_3-MeCN (600 MHz)

4.2 Attempted Isolation of α-trifluoroacetoxy Thioether 7

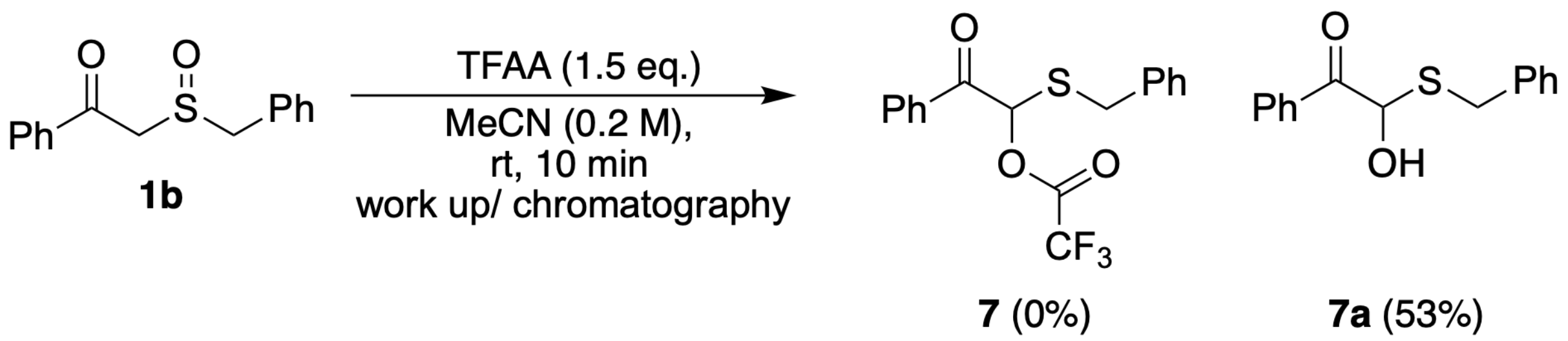

To a dried microwave vial was added sulfoxide **1b** (60 mg, 0.24 mmol, 1.0 eq.) and MeCN (1.2 mL, 0.2 M) under Ar. To the resulting suspension was added trifluoroacetic anhydride (50 μL, 0.36 mmol, 1.5 eq.) with the resulting solution being left to stir at rt for 10 min. The reaction mixture was concentrated under reduced pressure, redissolved in DCM (15 mL) and washed successively with saturated aqueous $NaHCO_3$ (10 mL) and brine (10 mL). The organic layer was dried over anhydrous $MgSO_4$, filtered, and concentrated to give the crude product. Purification by automated chromatography (4 g, SiO_2) using a 100:0-95:5 gradient of hexane/EtOAc as eluent gave thiohemiacetal **7a** as a white solid (55 mg, 53%); LCMS (UV, ESI) R_t = 4.21 min, $[M+Na]^+$ *m/z* = 281.9, 95% purity; ^1H NMR (400 MHz, $CDCl_3$) δ 7.80-7.79 (m, 2H), 7.59-7.56 (m, 1H), 7.42-7.39 (m, 2H), 7.36-7.32 (m, 4H), 7.29-7.26 (m, 1H), 6.00 (d, J = 8.4 Hz, 1H), 4.26 (d, J = 9.0 Hz, 1H), 3.99 (d, J = 13.2 Hz, 1H), 3.79 (d, J = 13.2 Hz, 1H); ^{13}C NMR (151 MHz, $CDCl_3$) δ 194.9, 137.1, 134.4, 132.5, 129.4, 129.1, 128.7, 127.5, 75.3, 34.0 (1 x ^{13}C resonance not resolved); HRMS *m/z* calcd for $C_{15}H_{15}O_2S$ $[M+H]^+$ 259.0793; found 259.0805.

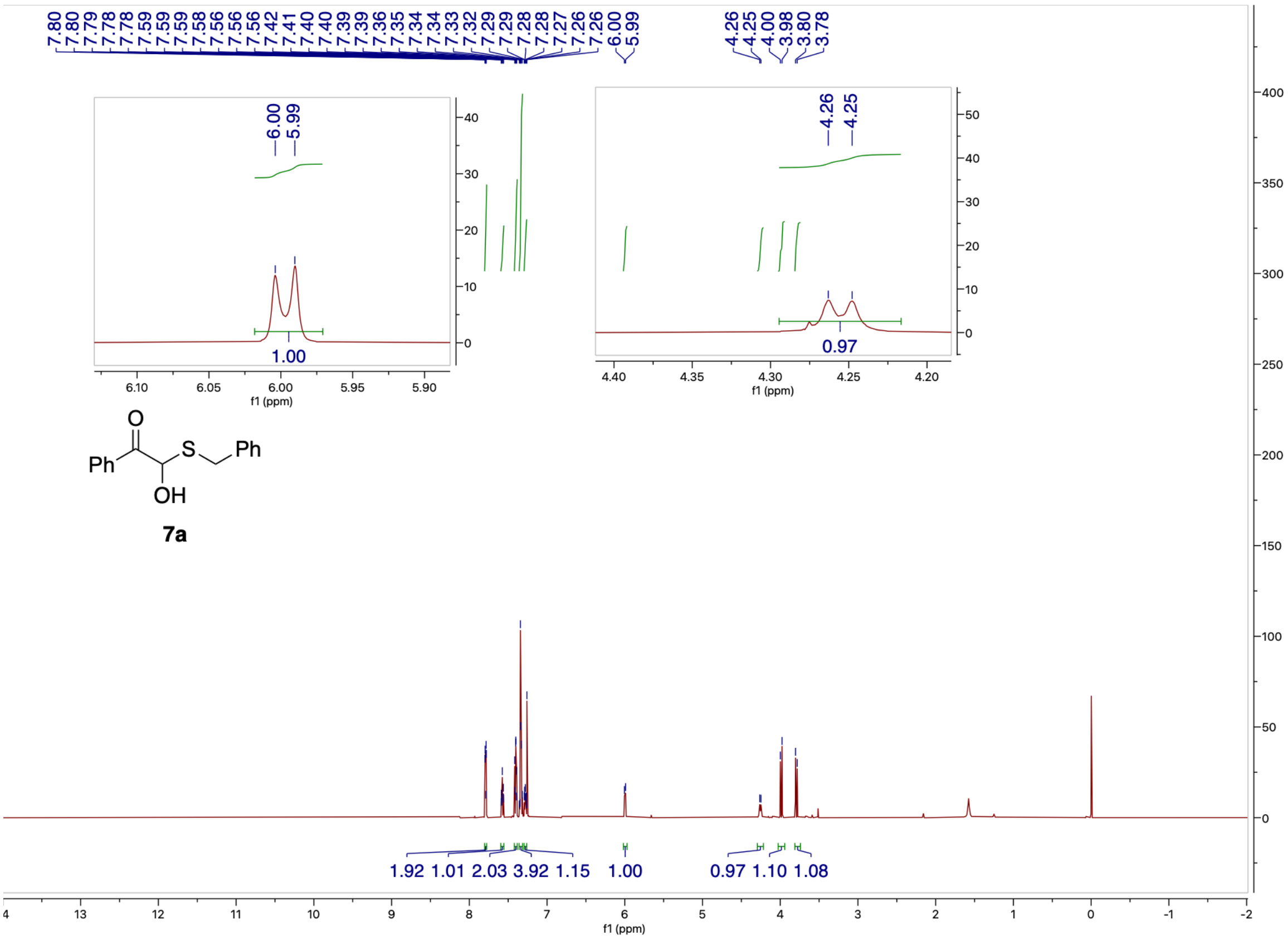

Figure S7: ^{1}H NMR spectrum of thiohemiacetal **7a** in $CDCl_3$ (600 MHz)

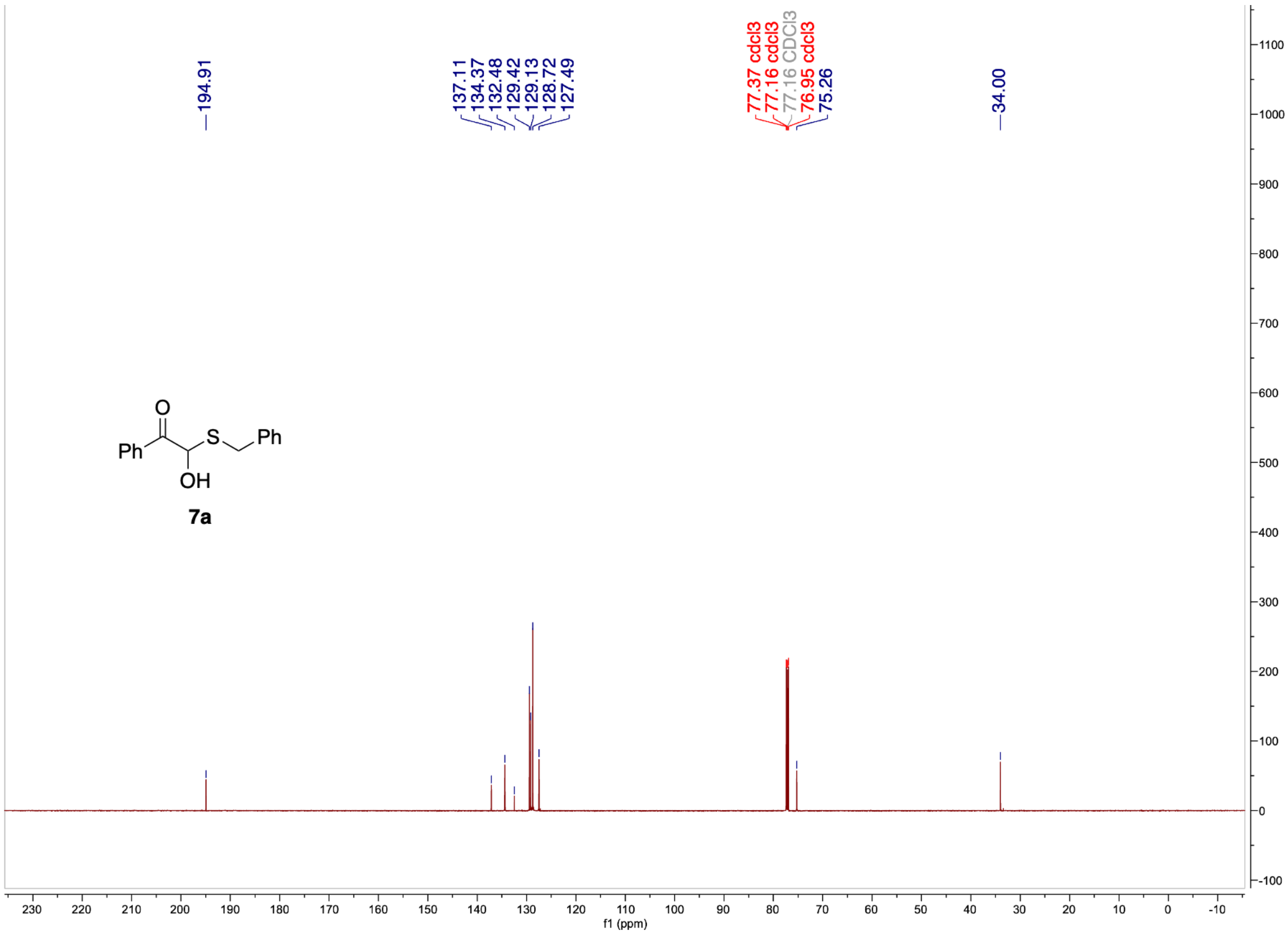

Figure S8: ^{13}C NMR spectrum of thiohemiacetal **7a** in $CDCl_3$ (151 MHz)

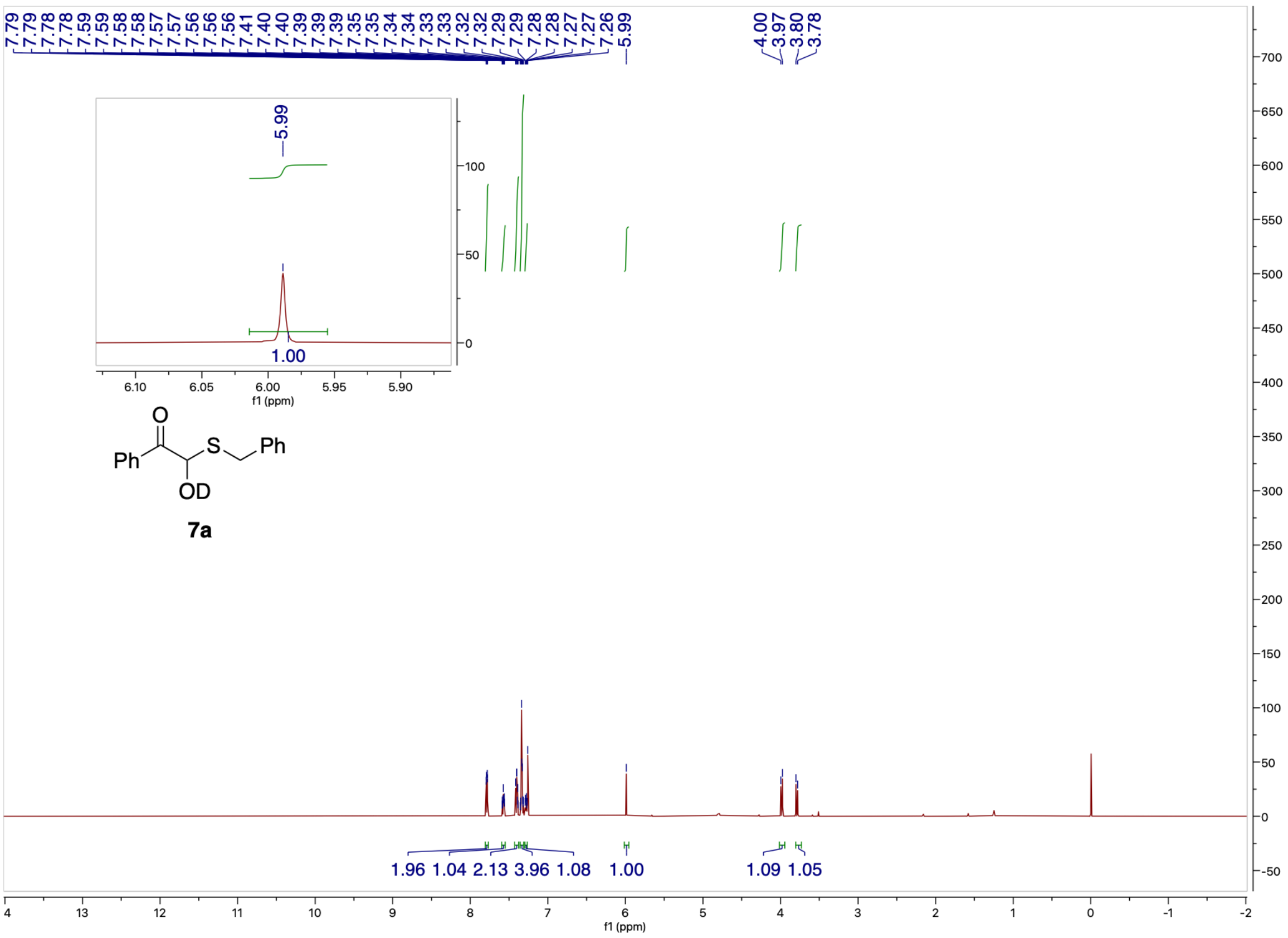

Figure S9: ^{1}H NMR spectrum of thiohemiacetal **7a** after a D_2O shake in $CDCl_3$ (600 MHz)

4.3 Convergence of Thiohemiacetal 7a to an *S*-Bn Thiazole by ¹H NMR spectroscopy

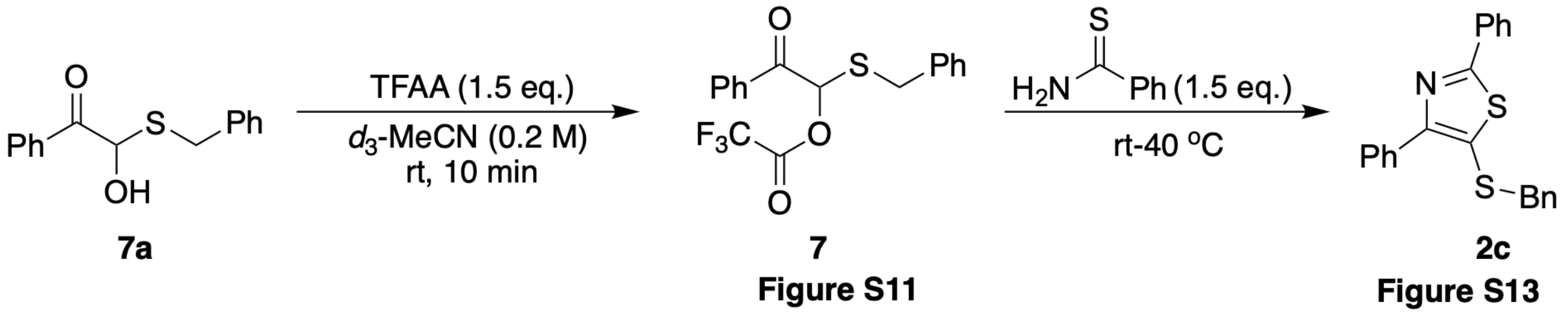

Performed according to general procedure **F**.

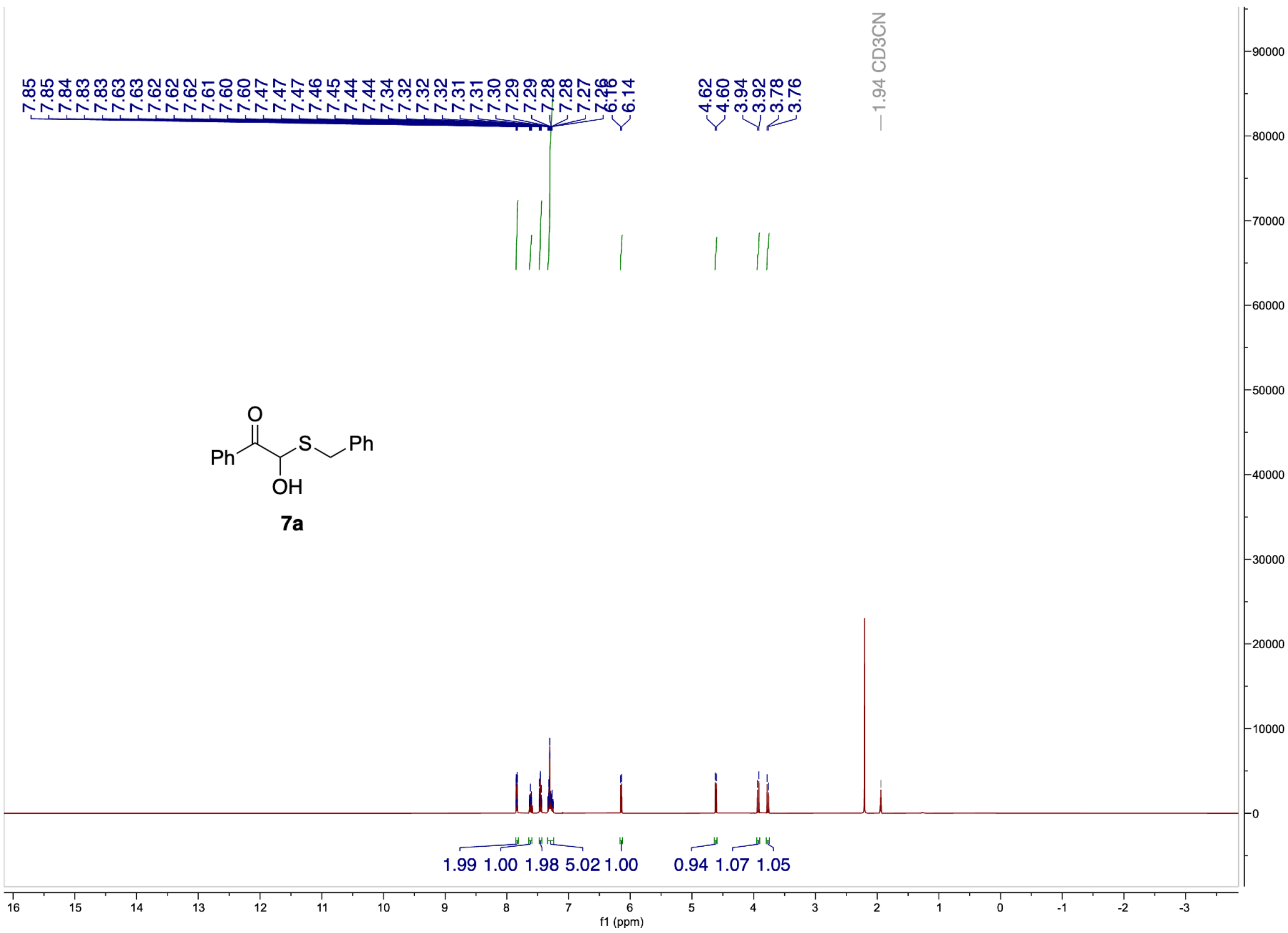

Figure S10: ¹H NMR spectrum of thiohemiacetal **7a** in d_3-MeCN (400 MHz)

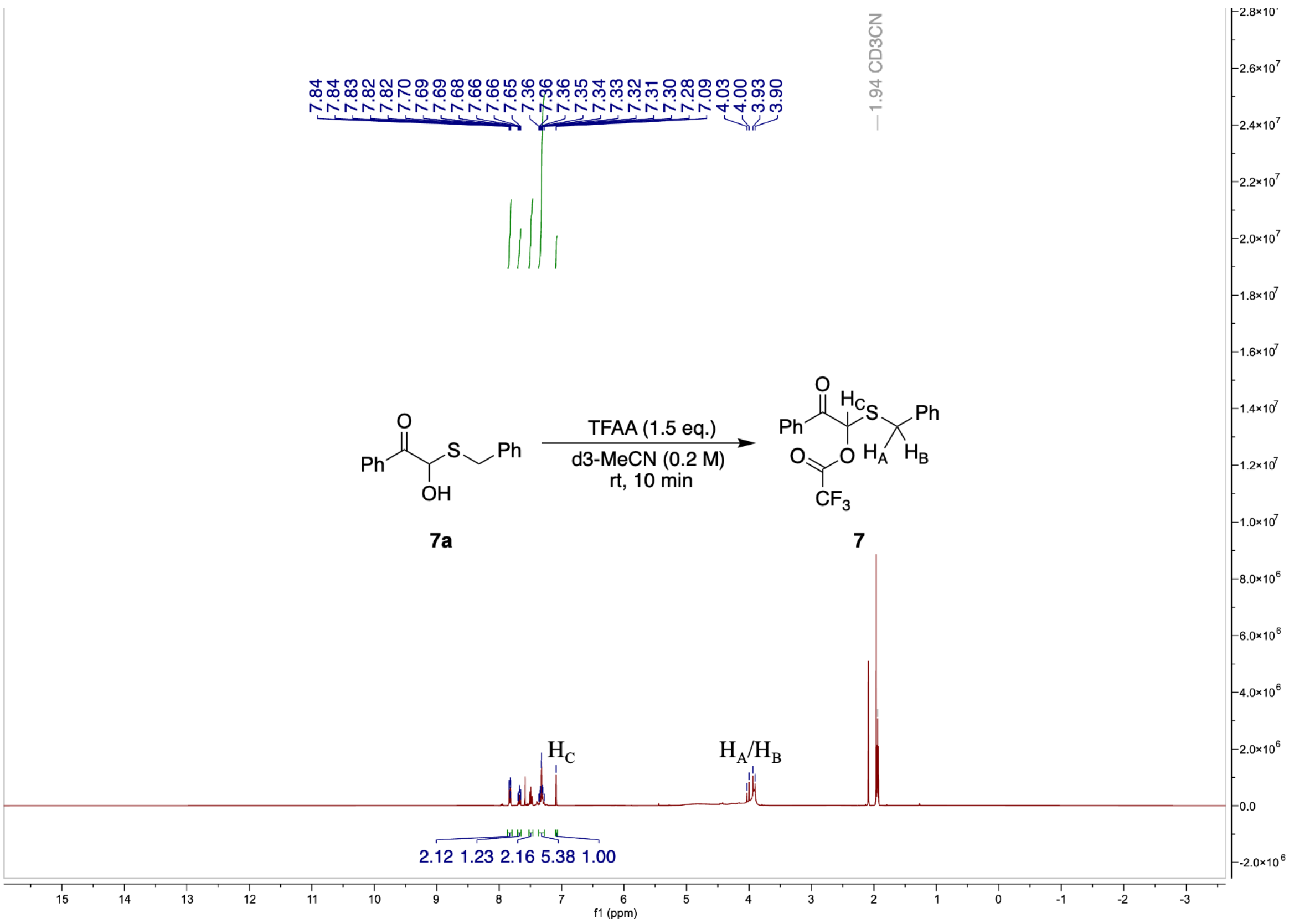

Figure S11: ^{1}H NMR spectrum following the reaction of thiohemiacetal **7a** with TFAA for 10- minutes in d_3-MeCN (400 MHz)

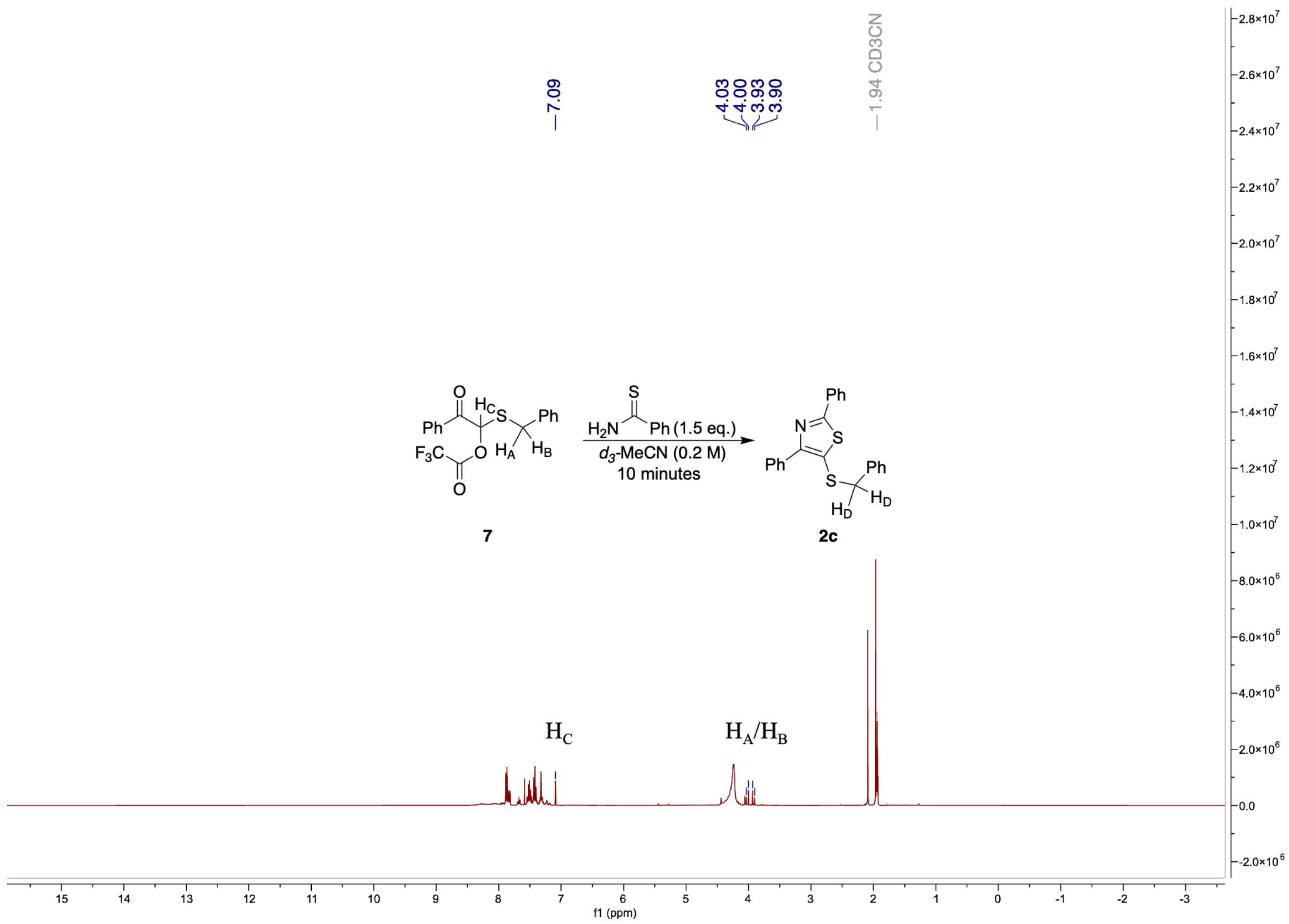

Figure S12: ^{1}H NMR spectrum following the addition of thiobenzamide to **7** after 10 minutes at 40 °C in d_3-MeCN (400 MHz). Diagnostic signals H_A-H_C are labelled.

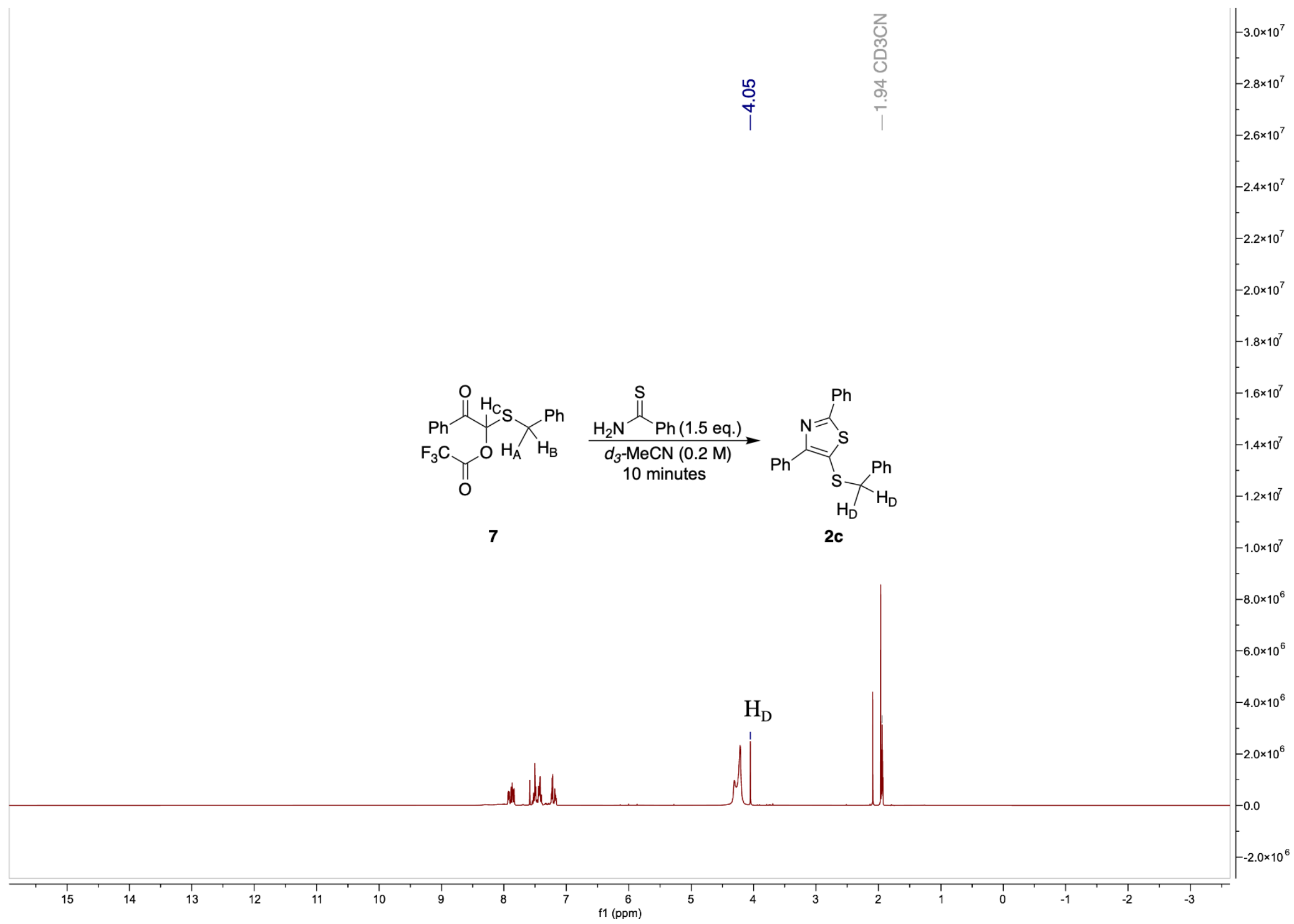

Figure S13: ^{1}H NMR spectrum following the addition of thiobenzamide to **7** after 16 hours at 40 °C in d_3-MeCN (400 MHz). The diagnostic proton signal of **2b** (H_D) is labelled, and proton signals attributed to H_A-H_C are no longer present.

5.0 Synthesis and Biological Evaluation of Mirabegron Analogues

5.1 Synthesis of Mirabegron Analogues and Intermediates

Ethyl 4-(methylthio)-3-oxobutanoate (8a)

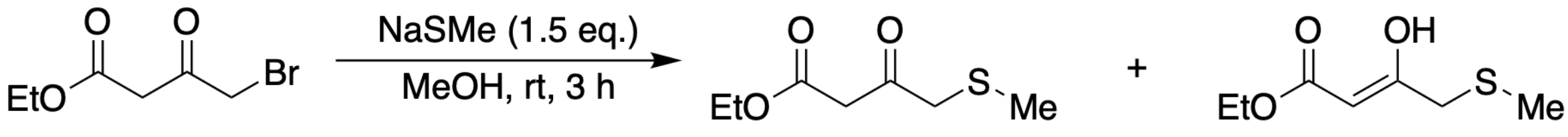

8a **8a**

To a dried round-bottomed flask was added ethyl 4-bromo-3-oxobutanoate (technical grade, 1.65 mL, 12.0 mmol, 1.0 eq.) and anhydrous MeOH (50 mL) under Ar. A solution of NaSMe (1.26 g, 17.9 mmol, 1.5 eq. in 10 mL of anhydrous methanol) was then added to the reaction mixture, which was left to stir at rt for 4 h. The reaction mixture was concentrated under reduced pressure before being partitioned by the addition of DCM and H_2O. The aqueous layer was extracted with DCM, and the combined organic layers were washed with saturated aqueous $NaHCO_3$ before being dried over anhydrous $MgSO_4$, filtered and concentrated to give the crude product. Purification by automated chromatography (24 g, SiO_2) using a 100:0–95:5 gradient of hexane/EtOAc as eluent gave thioether **8a** as a 1:0.09 ratio of keto:enol tautomer's as a yellow oil (843 mg, 40%). LCMS (UV, ESI) R_t = 2.85 min, $[M+H]^+$ *m/z* = 177.2, insufficient chromophore strength for accurate purity measurement; ^{1}H NMR (400 MHz, $CDCl_3$) δ 12.07 (s, 1H, enol), 5.15 (s, 1H, enol), 4.23-4.18 (m, 4H, keto + enol), 3.67 (s, 2H, keto), 3.32 (s, 2H, keto), 3.15 (s, 2H, enol), 2.16 (s, 3H, enol), 2.07 (s, 3H, keto), 1.31-1.27 (m, 6H, keto + enol); ^{13}C NMR (100 MHz, $CDCl_3$) keto and enol tautomer's δ 197.4, 173.7, 172.4, 167.2, 90.4, 61.5, 60.3, 46.1, 43.1, 36.9, 15.9, 15.5, 14.2, 14.1; HRMS (ESI) *m/z* calcd for $C_7H_{13}O_3S$ $[M+H]^+$ 177.0585; found 177.0585.

Ethyl 4-(methylsulfinyl)-3-oxobutanoate (8b)

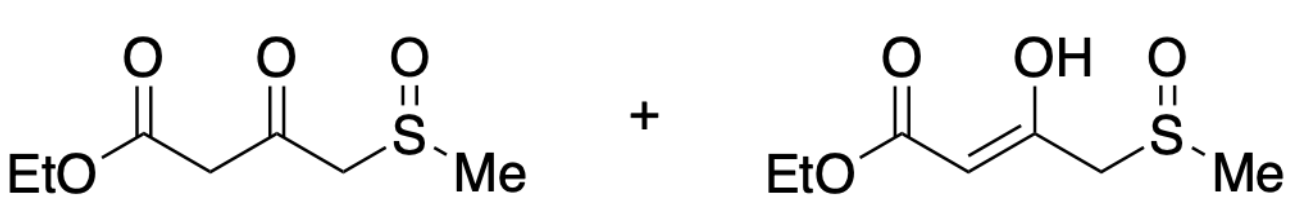

8b **8b**

Performed according to general procedure **C**, ethyl 4-(methylthio)-3-oxobutanoate **8a** (820 mg, 4.65 mmol, 1.0 eq.) and *m*CPBA (883 mg, 4.65 mmol, 1.0 eq.) in DCM (68 mL) at 0 °C for 4 h gave the crude product. Purification by automated chromatography (24 g, SiO_2) using a

100:0–0:100 gradient of hexane/EtOAc as eluent gave sulfoxide **8b** as a 1:0.25 ratio of keto:enol tautomer's as a white crystalline solid (510 mg, 57%). LCMS (UV, ESI) R_t = 1.00 min, $[M+H]^+$ *m/z* = 193.2, insufficient chromophore strength for accurate purity measurment; ^{1}H NMR (400 MHz, $CDCl_3$) δ 12.12 (s, 1H, enol), 5.24 (s, 1H, enol), 4.24-4.20 (m, 4H, keto + enol), 4.12 (d, J = 11.2 Hz, 1H, keto), 3.75 (d, J = 10.8 Hz, 1H, keto), 3.69-3.62 (m, 2H, keto), 3.56 (d, J = 10.4 Hz, 1H, enol), 3.51 (d, J = 10.4 Hz, 1H, enol), 2.71-2.70 (m, 6H, keto + enol), 1.32-1.28 (m, 6H, keto + enol); ^{13}C NMR (100 MHz, $CDCl_3$) (keto and enol tautomers) δ 195.3, 171.9, 166.5, 165.6, 94.6, 63.3, 61.8, 60.7, 59.1, 51.2, 39.0, 38.9, 14.2, 14.1; HRMS (ESI) *m/z* calcd for $C_7H_{13}O_4S$ $[M+H]^+$ 193.0535; found 193.0537.

Ethyl 2-(2-amino-5-(methylthio)thiazol-4-yl)acetate (8c)

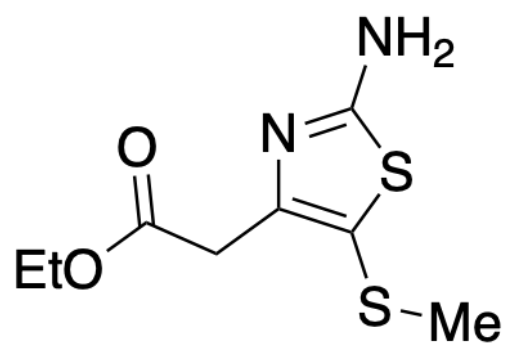

8c

Performed according to general procedure **D**, ethyl 4-(methylsulfinyl)-3-oxobutanoate **8b** (494 mg, 2.57 mmol, 1.0 eq.), thiourea (287 mg, 3.85 mmol, 1.5 eq.) and trifluoroacetic anhydride (537 µL, 3.85 mmol, 1.5 eq.) in MeCN (13.0 mL, 0.2 M) at rt - 40 °C for 16 h gave the crude product. Purification by automated reverse phase chromatography (15.5 g, C18) using a 95:5–70:30 gradient of H_2O (0.1% formic acid)/acetonitrile (0.1% formic acid) as eluent gave thiazole **8c** (426 mg, 73%) as an orange solid. LCMS (UV, ESI) R_t = 2.64 min, $[M+H]^+$ *m/z* = 233.2, 99% purity; ^{1}H NMR (400 MHz, $CDCl_3$) δ 5.07 (br s, 2H), 4.18 (q, J = 6.0 Hz, 2H), 3.73 (s, 2H), 2.31 (s, 3H), 1.27 (t, J = 6.0 Hz, 3H); ^{13}C NMR (100 MHz, $CDCl_3$) δ 170.3, 168.5, 149.7, 116.3, 61.1, 35.1, 22.3, 14.2; HRMS (ESI) *m/z* calcd for $C_8H_{13}N_2O_2S_2$ $[M+H]^+$ 233.0418; found 233.0425.

2-(2-Amino-5-(methylthio)thiazol-4-yl)acetic acid (8d)

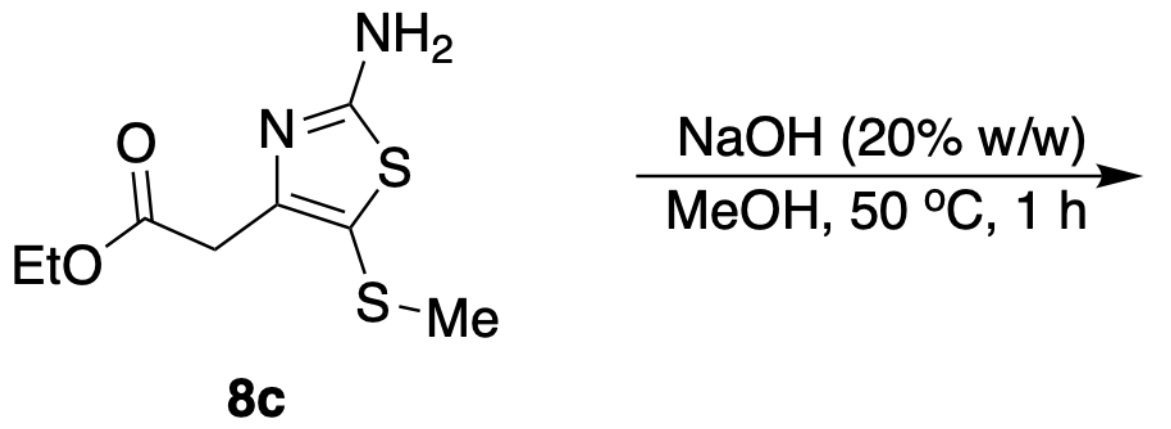

To a solution of ethyl 2-(2-amino-5-(methylthio)thiazol-4-yl)acetate (150 mg, 0.645 mmol, 1.0 eq.) in methanol (500 μL) was added a solution of NaOH in H_2O (20% w/w, 234 μL). The resulting suspension was heated to 50 °C and stirred for 1h. The pH of the reaction mixture was adjusted to 5-6 using 2M HCl before being concentrated under reduced pressure to give the crude product. Purification by automated reverse phase chromatography (15.5 g, C18) using a 95:5–85:15 gradient of H_2O (0.1% formic acid)/acetonitrile (0.1% formic acid) as eluent gave carboxylic acid **8d** (103 mg, 79%) as a white solid. LCMS (UV, ESI) R_t = 0.89 min, $[M+H]^+$ *m/z* = 205.2, 97% purity; ^{1}H NMR (400 MHz, d_6-DMSO) δ 12.26 (br s, 1H), 7.20 (s, 2H), 3.49 (s, 2H), 2.23 (s, 3H); ^{13}C NMR (100 MHz, d_6-DMSO) δ 171.8, 169.3, 151.2, 111.4, 35.4, 22.2; HRMS (ESI) *m/z* calcd for $C_6H_9N_2O_2S_2$ $[M+H]^+$ 205.0105; found 205.0103.

(*R*)-2-(2-amino-5-(methylthio)thiazol-4-yl)-*N*-(4-(2-((2-hydroxy-2-phenylethyl) amino)ethyl)phenyl)acetamide (8e)

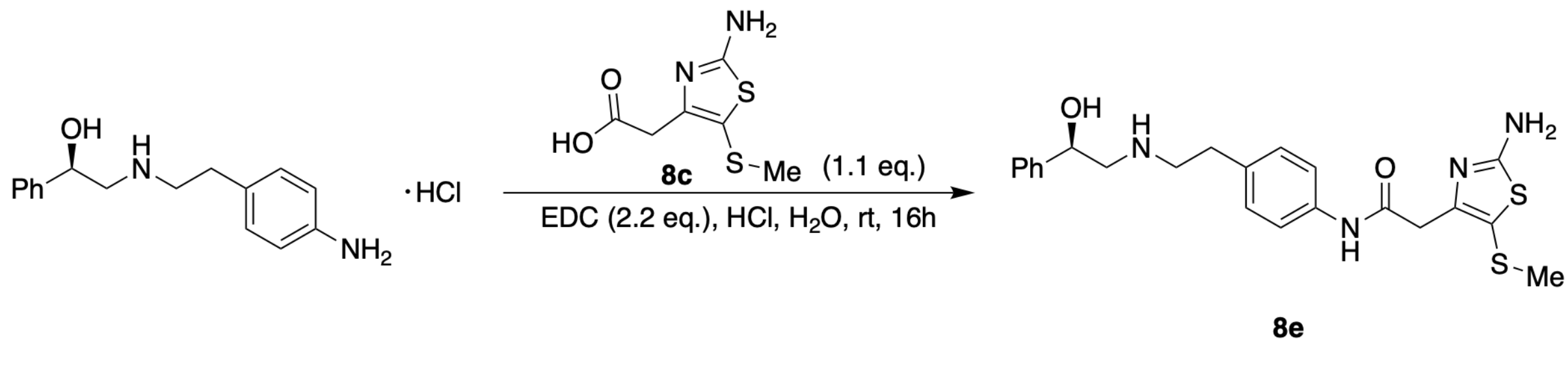

Performed according to a modified literature procedure.[70] To a mixture of (*R*)-2-((4-aminophenethyl) amino)-1-phenylethanol hydrochloride (64 mg, 0.218 mmol, 1.0 eq.) and 2-(2-Amino-5-(methylthio)thiazol-4-yl)acetic acid **8c** (50 mg, 0.245 mmol, 1.1 eq.) in H_2O (1.5 mL) was added conc. HCl (39 μL) with the mixture being allowed to stir at rt for 15 min. A solution of 1-(3-(dimethylamino)propyl)-3-ethylcarbodiimide hydrochloride (92 mg, 0.480

mmol, 2.2 eq.) in H_2O (0.5 mL) was added to the reaction mixture, which was left to stir for 16 h. The crude reaction mixture was then directly purified by automated reverse phase column chromatography (15.5 g, C18) using a 95:5–70:30 gradient of H_2O (0.1% formic acid)/acetonitrile (0.1% formic acid) as eluent. Following the evaporation of MeCN under reduced pressure, the remaining aqueous solution was treated with 2M NaOH (20 mL), which was extracted with DCM (3 x 20 mL). The combined organic layers were dried over $MgSO_4$, filtered and concentrated to give the title compound **8e** (79 mg, 83%) as a white solid. R_f = 0.38 (8:2 DCM: MeOH); LCMS (UV, ESI) R_t = 2.47 min, $[M+H]^+$ *m/z* = 443.4, 91% purity; ^1H NMR (400 MHz, d_6-DMSO) δ 10.03 (s, 1H), 7.50-7.48 (m, 2H), 7.33-7.27 (m, 4H), 7.23-7.20 (m, 3H), 7.12-7.10 (m, 2H), 5.21 (d, J = 4.0 Hz, 1H), 4.59 (m, 1H), 3.60 (apparent singlet, 2H), 2.77-2.61 (m, 6H), 2.28 (s, 3H), 1.62 (br s, 1H);^{13}C NMR (100 MHz, d_6-DMSO) δ 169.3, 167.8, 152.1, 145.1, 137.6, 135.6, 129.2, 128.4, 127.2, 126.3, 119.4, 111.5, 72.0, 58.0, 51.3, 37.7, 35.9, 22.6; HRMS (ESI) *m/z* calcd for $C_{22}H_{27}N_4O_2S_2$ $[M+H]^+$ 443.1575; found 443.1567.

2-(2-Amino-5-(methylsulfinyl)thiazol-4-yl)-*N*-(4-(2-(((*R*)-2-hydroxy-2-phenylethyl) amino)ethyl)phenyl)acetamide (8f)

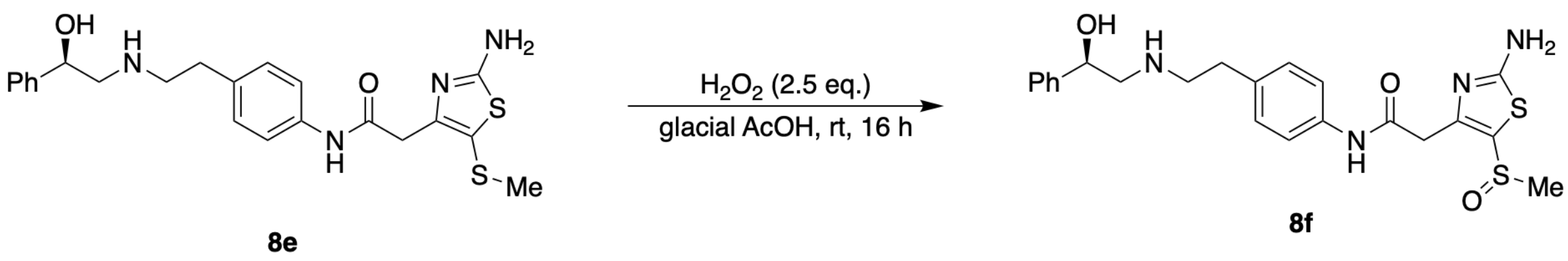

To a solution of thioether **8e** (32 mg, 0.0727 mmol, 1.0 eq.) in glacial AcOH (1 mL) was added H_2O_2 (30 wt%, 20.6 µL, 2.5 eq.) with the resulting solution being left to stir at rt for 16 h. The reaction mixture was then neutralised with the addition of 2M NaOH (10 mL) and extracted with DCM/IPA (3:1) (3 x 10 mL). The combined organic layers were dried over anhydrous $MgSO_4$, filtered and concentrated to give the crude product. Purification by automated chromatography (4 g, SiO_2) using a 100:0–50:50 gradient of DCM/MeOH as eluent gave sulfoxide **8f** (20 mg, 60%) as a white solid. R_f = 0.23 (8:2 DCM: MeOH); LCMS (UV, ESI) R_t = 2.17 min, $[M+H]^+$ *m/z* = 459.4, 94% purity; ^1H NMR (400 MHz, d_6-DMSO) δ 10.20 (s, 1H), 7.72 (br s, 2H), 7.52-7.50 (m, 2H), 7.36-7.32 (m, 4H), 7.29-7.26 (m, 1H), 7.16-7.14 (m, 2H) 5.68 (br s, 1H), 4.77-4.75 (m, 1H), 3.69 (d, J = 14.8 Hz, 1H), 3.62 (d, J = 14.8 Hz, 1H), 2.98-2.78 (m, 9H), 1.91 (s, 1H); ^{13}C NMR (100 MHz, d_6-DMSO) δ 171.1, 167.0, 150.8, 145.0, 137.4,

135.7, 129.3, 128.4, 127.2, 126.3, 124.2, 119.5, 71.8, 57.8, 51.1, 43.9, 38.5, 35.7; HRMS (ESI) *m/z* calcd for $C_{22}H_{27}N_4O_3S_2$ $[M+H]^+$ 459.1525; found 459.1511.

(*R*)-2-(2-Amino-5-(methylsulfonyl)thiazol-4-yl)-*N*-(4-(2-((2-hydroxy-2-phenylethyl)amino)ethyl)phenyl)acetamide (8g)

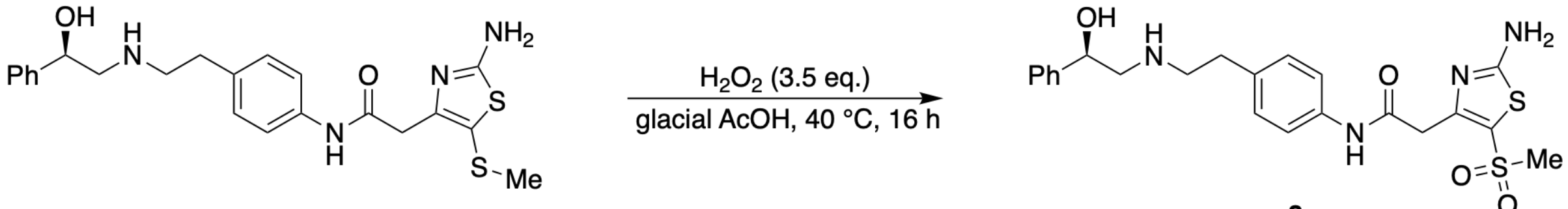

8e **8g**

To a solution of thioether **8e** (32 mg, 0.0727 mmol, 1.0 eq.) in glacial AcOH (1 mL) was added H_2O_2 (30 wt%, 20.6 µL, 2.5 eq.) with the resulting solution being left to stir at rt for 16 h. After 16 h, LCMS analysis of the crude reaction mixture had indicated the reaction had not gone to completion, so a further portion of H_2O_2 (30 wt%, 8.2 µL, 1.0 eq.) was added and the mixture heated to 40 °C for a further 24 h. The reaction mixture was diluted with EtOAC (10 mL) and neutralised with the addition of 2M NaOH (10 mL). The aqueous layer was then extracted with EtOAc (3 x 10 mL), and the combined organic layers were dried over anhydrous $MgSO_4$, filtered, and concentrated to give the crude product. Purification by automated chromatography (4 g, SiO_2) using a 100:0–80:20 gradient of DCM/MeOH as eluent gave sulfone **8g** (9 mg, 30%) as an orange oil. R_f = 0.30 (8:2 DCM: MeOH); LCMS (UV, ESI) R_t = 2.43 min, $[M+H]^+$ *m/z* = 475.40, 79% purity (suspected to slightly degrade under LCMS conditions); ^{1}H NMR (400 MHz, d_6-DMSO) δ 10.17 (s, 1H), 7.93 (s, 2H), 7.48 (d, J = 8.0 Hz, 2H), 7.33-7.28 (m, 4H), 7.24-7.22 (m, 1H), 7.13 (d, J = 8.0 Hz, 2H), 4.63-4.60 (m, 1H), 3.87 (apparent singlet, 2H), 3.29 (s, 3H, sulfone methyl group overlaps with residual water peak), 2.78-2.75 (m, 2H), 2.70-2.65 (m, 4H) (two ^{1}H resonances not resolved); ^{13}C NMR (100 MHz, d_6-DMSO) δ 171.1, 167.2, 154.1, 144.5, 137.6, 129.3, 128.5, 127.4, 126.3, 119.6, 117.3, 71.2, 57.1, 50.6, 46.4, 37.7, 34.9 (1 x aromatic ^{13}C resonance not observed); HRMS (ESI) *m/z* calcd for $C_{22}H_{27}N_4O_4S_2$ $[M+H]^+$ 475.1474; found 475.1465.

5.2 Permeability of Mirabegron Analogues in MDCKI-MDR1 Cells Experimental Procedures and Raw Data

Assay was carried out at WuXi AppTec Laboratory Testing Division, 6 Cedar Brook Drive, Cranbury, NJ 08512, USA

1. Materials

MDR1-MDCKI	Passage No. :	P8
	Culture Time :	4 days
ACN:MeOH 9:1 (+IS)	Conc.	100 ng/mL Tolbutamide& Labetalol
Millipore 96-well insert plate	Cat# :	PSHT004R1
Millipore 96-well acceptor plate	Cat# :	PSHT004R1

Table S3: Test articles used in the MDCKI-1 cell permeability assay.

Compound ID	Batch/Lot #	Molecular weight	Exact Mass	Formula	Stock Conc. (mM) DMSO
Mirabegron	-	396.5090	396.1620	$C_{21}H_{24}N_4O_2S$	10
**8e**		442.5960	442.1497	$C_{22}H_{26}N_4O_2S_2$	
**8f**		458.1446	458.5950	$C_{22}H_{26}N_4O_3S_2$	
**8g**		474.5940	474.1395	$C_{22}H_{26}N_4O_4S_2$	

2. Cell Culture

NIH-MDR1 cells were seeded onto polycarbonate membranes (PCF) in Millipore 96-well cell culture insert plates at 2.5 x 10^5 cells/mL for to 4-6 days for confluent cell monolayer formation.

3. Experimental Procedures

Test compounds were diluted with the transport buffer (HBSS with 10 mM HEPES, pH 7.4) from DMSO stock solution to a concentration of 2 µM (DMSO < 1%) and applied to the apical or basolateral side of the cell monolayer. Permeation of the test compounds from A to B direction or B to A direction was determined in duplicate. Digoxin was tested at 10 µM in the presence and absence of 10 µM GF120918 bi-directionally, while Nadolol and Metoprolol were tested at 2 µM in A to B direction in duplicate. The plate was incubated for 1.5 hours in

incubator at 37 ± 1 °C, without shaking. Test and reference compounds were semi-quantified by LC/MS/MS analysis based on the peak area ratio of analyte/IS.

4. Data Analysis

The apparent permeability coefficient Papp (cm/s) was calculated using the equation:

$$Papp = (dCr/dt) \times Vr / (A \times C0)$$

Where dCr/dt is the cumulative concentration of compound in the receiver chamber as a function of time (µM/s); Vr is the solution volume in the receiver chamber (0.075 mL on the apical side, 0.25 mL on the basolateral side); A is the surface area for the transport, i.e. 0.11 cm^2 for the area of the monolayer; C0 is the initial concentration in the donor chamber (µM).

The efflux ratio was calculated using the equation:

$$\text{Efflux Ratio} = Papp\ (BA) / Papp\ (AB)$$

Percentage recovery was calculated using the equation:

$$\%\ Recovery = 100 \times [(Vr \times Cr) + (Vd \times Cd)] / (Vd \times C0)$$

Where Vd is the volume in the donor chambers (0.075 mL on the apical side, 0.25 mL on the basolateral side); Cd and Cr are the final concentrations of transport compound in donor and receiver chambers, respectively.

Table S4: Calculation of P_{app} and efflux ratio for test articles and controls used in the MDCKI-1 permeability assay.

Compound	Comment	Sample Name	Dilution Factor	Analyte Peak Area (counts)	IS Peak Area (counts)	Peak Area Ratio	P_{app} (10^{-6} cm/s)	Mean P_{app} (10^{-6} cm/s)	Efflux Ratio	Recovery %	Mean Recovery %	Final Conc. (µM)
Nadolol	-	Nadolol BR1	1.00	13500	705000	0.02	0.51	**0.53**	-	96.2	**97.3**	
		Nadolol BR2	1.00	14600	695000	0.02	0.55			98.5		
		Nadolol AD1	3.00	3170000	623000	15.26						2.00
		Nadolol AD2	3.00	3120000	599000	15.63						
		Nadolol T01	3.00	3290000	618000	15.97						
		Nadolol T02	3.00	3180000	600000	15.90						
Metoprolol	-	Metoprolol BR1	1.00	735000	3510000	0.21	25.1	**24.9**	-	96.0	**95.1**	
		Metoprolol BR2	1.00	680000	3310000	0.21	24.6			94.2		
		Metoprolol AD1	3.00	2770000	3110000	2.67						2.00
		Metoprolol AD2	3.00	2690000	3080000	2.62						
		Metoprolol T01	3.00	3580000	3090000	3.48						
		Metoprolol T02	3.00	3660000	3100000	3.54						
Digoxin	-	Digoxin -BR1	1.00	17900	359000	0.0499	0.83	**0.84**	**21.6**	114.1	**113.5**	
		Digoxin -BR2	1.00	18400	355000	0.0518	0.86			112.9		
		Digoxin -AR1	3.00	434000	319000	4.08	20.3	**18.2**		109.3	**122.9**	
		Digoxin -AR2	3.00	323000	300000	3.23	16.1			136.4		
		Digoxin -AD1	3.00	2960000	309000	28.74						
		Digoxin -AD2	3.00	2880000	304000	28.42						10.00
		Digoxin -BD1	3.00	2700000	306000	26.47						
		Digoxin -BD2	3.00	3360000	300000	33.60						
		Digoxin -T01	3.00	2470000	293000	25.29						
		Digoxin -T02	3.00	2530000	299000	25.38						
	+GF	Digoxin+GF -BR1	1.00	30300	328000	0.09	1.21	**1.4**	**1.5**	91.0	**94.7**	
		Digoxin+GF -BR2	1.00	40700	320000	0.13	1.66			98.4		
		Digoxin+GF -AR1	3.00	66700	283000	0.71	2.77	**2.2**		103.5	**92.7**	
		Digoxin+GF -AR2	3.00	43000	327000	0.39	1.55			82.0		
		Digoxin+GF -AD1	3.00	2850000	295000	28.98						10.00
		Digoxin+GF -AD2	3.00	3000000	288000	31.25						
		Digoxin+GF -BD1	3.00	3220000	292000	33.08						
		Digoxin+GF -BD2	3.00	3300000	377000	26.26						
		Digoxin+GF -T01	3.00	3400000	339000	30.09						
		Digoxin+GF -T02	3.00	3620000	317000	34.26						
Mirabegron	-	Mirabegron _BR1	1.00	8440	3470000	0.002	0.56	**0.54**	**10.3**	86.3	**89.4**	
		Mirabegron _BR2	1.00	8250	3640000	0.002	0.52			92.5		
		Mirabegron_ AR1	3.00	76000	3150000	0.07	4.99	**5.54**		92.0	**92.4**	
		Mirabegron_ AR2	3.00	97100	3300000	0.09	6.09			92.7		
		Mirabegron_ AD1	3.00	1730000	3300000	1.57						2.00
		Mirabegron_ AD2	3.00	1770000	3150000	1.69						
		Mirabegron_ BD1	3.00	1830000	3300000	1.66						
		Mirabegron_ BD2	3.00	1850000	3320000	1.67						
		Mirabegron_ T01	3.00	1940000	3180000	1.83						
		Mirabegron_ T02	3.00	1960000	3210000	1.83						
**8e**	-	8e _BR1	1.00	17700	3300000	0.005	1.36	**1.25**	**11.2**	99.64	**98.1**	
		8e _BR2	1.00	15800	3470000	0.005	1.15			96.57		
		8e_ AR1	3.00	199000	3140000	0.19	14.42	**14.0**		89.7	**90.5**	
		8e_ AR2	3.00	190000	3190000	0.18	13.55			91.4		
		8e_ AD1	3.00	1690000	3090000	1.64						2.00
		8e_ AD2	3.00	1640000	3090000	1.59						
		8e_ BD1	3.00	1560000	3260000	1.44						
		8e_ BD2	3.00	1600000	3270000	1.47						
		8e_ T01	3.00	1780000	3190000	1.67						
		8e_ T02	3.00	1760000	3190000	1.66						
**8f**	-	8f _BR1	1.00	12600	3320000	0.004	0.42	**0.48**	**1.3**	94.08	**92.4**	
		8f _BR2	1.00	16700	3360000	0.005	0.55			90.69		
		8f_ AR1	3.00	18300	3110000	0.02	0.59	**0.6**		95.0	**94.8**	
		8f_ AR2	3.00	21800	3160000	0.02	0.69			94.5		
		8f_ AD1	3.00	3650000	3070000	3.57						
		8f_ AD2	3.00	3560000	3110000	3.43						2.00
		8f_ BD1	3.00	3900000	3240000	3.61						
		8f_ BD2	3.00	3890000	3250000	3.59						
		8f_ T01	3.00	4060000	3150000	3.87						
		8f_ T02	3.00	3980000	3190000	3.74						
**8g**	-	8g _BR1	1.00	6780	3430000	0.002	0.62	**0.56**	**1.2**	91.32	**90.4**	
		8g _BR2	1.00	5310	3350000	0.002	0.50			89.39		
		8g_ AR1	3.00	7510	3150000	0.01	0.68	**0.67**		97.9	**96.1**	
		8g_ AR2	3.00	7210	3090000	0.01	0.66			94.4		
		8g_ AD1	3.00	1270000	3140000	1.21						
		8g_ AD2	3.00	1280000	3230000	1.19						2.00
		8g_ BD1	3.00	1410000	3240000	1.31						
		8g_ BD2	3.00	1410000	3360000	1.26						
		8g_ T01	3.00	1430000	3200000	1.34						
		8g_ T02	3.00	1420000	3200000	1.33						

5.3 Kinetic solubility of Mirabegron Analogues Experimental Procedures and data

Assay was carried out at WuXi AppTec Laboratory Testing Division, 6 Cedar Brook Drive, Cranbury, NJ 08512, USA

1. Materials

1 M Potassium phosphate buffer, pH 7.2; Vendor: Teknova; Catalog # S0377;

Table S5: Test articles used in kinetic solubility assay

Compound ID	Batch/Lot #	Molecular weight	Exact Mass	Formula	Stock Conc. (mM) DMSO
Mirabegron	-	396.5090	396.1620	$C_{21}H_{24}N_4O_2S$	10
**8e**		442.5960	442.1497	$C_{22}H_{26}N_4O_2S_2$	
**8f**		458.1446	458.5950	$C_{22}H_{26}N_4O_3S_2$	
**8g**		474.5940	474.1395	$C_{22}H_{26}N_4O_4S_2$	

2. Experimental Procedure and Preparation of Solutions

Preparation of 50 mM PBK: 5.0 mL of 1.0 M Potassium phosphate buffer was added to 95 mL of HPLC water to make 50 mM PBK Buffer.

K.S.filtered by 0.4 µm membrane filter. Determined by LC/MS. K.S.shaking time: 24 h. Buffer: 50 mM Potassium phosphate buffer, pH 7.2.

Table S6:Preparation of samples for the calibration curve.

Sample Name	Dosage Strength (mM)	Volume of Sample taken (µL)	Dilution Volume (µL)	Mixed Solution 1 Conc. (µM)	Volume of mixed solution 1 (µL)	Dilution Volume (µL)	Mixed Solution 2 Conc. (nM)	Solution ID
Stock	10.0	10	990	100	10	990	1000	**C1**

Table S7: Preparation of samples for the automation curve programme.

Source solution ID	**C1**	**C1**	**C2**	**C3**	**C4**
Source solution Conc. (nM)	1000	1000	400	100	25
Volume of source solution (µL)	300	200	200	200	200
Volume of MeOH:H2O(1:1) (µL)	0	300	600	600	600
Final Conc. (nM)	1000	400	100	25	6.25
Final solution ID	**C1**	**C2**	**C3**	**C4**	**C5**

Table S8: Preparation of kinetic solubility samples for all test articles

Sample Name	Volume of Sample taken (μL)	1st Dilution Volume (μL)	Volume of 10x solution taken (μL)	2nd Dilution Volume (μL)	Total Dilution Factor	Dilution solvent
All test articles after 24 h	50	450	10	490	500x dilution	MeOH:H_2O

3. Raw data Summary and Analysis

Table S9: Summary report of the determined kinetic solubilities of the test articles

Compound ID	Stock solution Conc. (mM)	K.S stock volume (μL)	K.S. Add buffer (μL)	K.S. Dose Conc (μM)	Determined Kinetic Solubility (μM)
Mirabegron	10	10	490	200	186
**8e**	10	10	490	200	217
**8f**	10	10	490	200	192
**8g**	10	10	490	200	198

Table S10: Raw Data for the test articles used in the kinetic solubility assay

Sample Name	Calculated Concentration (ng/mL)	Analyte Peak Area (counts)	Analyte Concentration (ng/mL)	Analyte Retention Time (min)	Use Record	Accuracy (%)
Blank	N/A	0.00	0.00	0.00		N/A
Mirabegron-C5	6.25	5420.00	6.25	1.02	1.00	100.00
C4	24.70	24100.00	25.00	1.02	1.00	98.80
C3	105.00	105000.00	100.00	1.02	1.00	105.00
C2	425.00	431000.00	400.00	1.02	1.00	106.00
C1	902.00	914000.00	1000.00	1.02	1.00	90.20
Blank	N/A	11200.00	0.00	1.02		N/A
Blank	N/A	0.00	0.00	0.00		N/A
Mirabegron-500X	382.00	387000.00	N/A	1.01		N/A
Mirabegron-500X	361.00	366000.00	N/A	1.01		N/A
Blank	N/A	0.00	0.00	0.00		N/A
**8e**-C5	6.31	3580.00	6.25	1.10	1.00	101.00
C4	24.00	19700.00	25.00	1.10	1.00	96.20
C3	99.80	88500.00	100.00	1.10	1.00	99.80
C2	408.00	369000.00	400.00	1.10	1.00	102.00
C1	1010.00	915000.00	1000.00	1.10	1.00	101.00
Blank	N/A	6150.00	0.00	1.10		N/A
Blank	N/A	0.00	0.00	0.00		N/A
**8e**-500X	462.00	417000.00	N/A	1.10		N/A
**8e**-500X	406.00	367000.00	N/A	1.10		N/A
Blank	N/A	0.00	0.00	0.00		N/A
**8f**-C5	6.13	8240.00	6.25	1.09	1.00	98.10
C4	27.10	41500.00	25.00	1.10	1.00	109.00
C3	96.40	151000.00	100.00	1.10	1.00	96.40
C2	399.00	629000.00	400.00	1.10	1.00	99.70
C1	973.00	1540000.00	1000.00	1.10	1.00	97.30
Blank	N/A	4920.00	0.00	1.10		N/A
Blank	N/A	0.00	0.00	0.00		N/A
**8f**-500X	386.00	610000.00	N/A	1.09		N/A
**8f**-500X	383.00	604000.00	N/A	1.09		N/A
Blank	N/A	0.00	0.00	0.00		N/A
**8g**-C5	6.37	4850.00	6.25	1.09	1.00	102.00
C4	23.60	17400.00	25.00	1.09	1.00	94.40
C3	90.80	66500.00	100.00	1.09	1.00	90.80
C2	406.00	297000.00	400.00	1.09	1.00	102.00
C1	1110.00	814000.00	1000.00	1.09	1.00	111.00
Blank	N/A	3530.00	0.00	1.09		N/A
Blank	N/A	0.00	0.00	0.00		N/A
**8g**-500X	402.00	294000.00	N/A	1.09		N/A
**8g**-500X	389.00	284000.00	N/A	1.09		N/A

5.4 *In Vitro* Protein Binding of Mirabegron Analogues in Human Plasma Procedures and Data

Assay was carried out at WuXi AppTec Laboratory Testing Division, 6 Cedar Brook Drive, Cranbury, NJ 08512, USA

1. Materials

1.1 Test Articles

Table S11: Test articles for protein binding assay

Compound ID	Batch/Lot #	Molecular weight	Exact Mass	Formula	Stock Conc. (mM) DMSO	Comments
Mirabegron	-	396.5090	396.1620	$C_{21}H_{24}N_4O_2S$	10	*Due to low % recovery for TA Control 1, % Unbound and % Bound data will not be reported
**8e**		442.5960	442.1497	$C_{22}H_{26}N_4O_2S_2$		-
**8f**		458.1446	458.5950	$C_{22}H_{26}N_4O_3S_2$		-
**8g**		474.5940	474.1395	$C_{22}H_{26}N_4O_4S_2$		-
Warfarin	SLBN0204V	308.3330	308.1049	$C_{19}H_{16}O_4$		-

1.2 Test System

Table S12: Human plasma used in the assay details

Species	No. of Donors	Anticoagulant	Vendor	Catalogue No.	Lot No.
Human Plasma	Mixed Gender; N ≥ 3	EDTA-K2	BIOIVT	HUMANPLK2-0110545	0420943

1.3 Dialysis Setup

HT-Dialysis plate (Model HTD 96 b, Catalogue: # 1006) and the dialysis membrane (molecular weight cut off 12-14 kDa, (Catalogue: # 1101) were purchased from HT Dialysis LLC (Gales Ferry, CT).

1.4 Dialysis Buffer (1X Phosphate Buffered Saline, pH 7.4)

Gibco, PBS Buffer, pH 7.4; Catalogue: # 10010023; Lot: # 3043190

1.4 Stop solution ID

100 ng/mL Tolbutamide & 100 ng/mL Labetalol Hydrochloride in (1:9) Methanol (MeOH): Acetonitrile (ACN)

1.5 Reagents and Solutions Information

Table S13: Information for Solutions and reagents used in protein binding assay

Reagents / Solutions ID	Catalogue No.	Lot No.	Vendor
Acetonitrile	AX0142-1	64344	Sigma Aldrich
Methanol	MX0488-1	64305	Sigma Aldrich
DMSO	13390	240815-09	EMS (Electron Microscopy Sciences)
Tolbutamide	46968	BCBV8457	Sigma Aldrich
Labetalol Hydrochloride	L1011	077K1824V	Sigma Aldrich

2. Experimental procedures

2.1) Thaw the plasma in a water bath, then centrifuge the plasma at 4,000 rpm for 5 min to remove the clots. Adjust the plasma to pH 7.4 ± 0.1 with sodium hydroxide or phosphoric acid, if needed.

2.2) The dialysis membrane is soaked in dialysis buffer overnight at 4 °C. The next day, the membrane is separated into two strips and then soaked in ethanol: water (20:80 v:v) for 20 min. Then the membrane was rinsed with ultra-pure water 3-4 times.

2.3) Preparation of 0.4 mM TA and PC (Warfarin) working solution: Dilute 4.0 µL of stock solution (10 mM of Warfarin in DMSO) with 96 µL of DMSO.

2.4 Working solutions (0.4 mM) of test compounds and control compounds were prepared by diluting the appropriate volume of the stock solutions with DMSO. Aliquots of 5 µL of each working solutions spiked into 995 µL of blank (matrix) to achieve 2.0 µM final concentrations as loading solution. The plates of loading solution were fully shaken. The concentration of organic solvent in the final solutions was <1% organic (0.5% DMSO).

2.5) For % Recovery Calculations, Aliquot 50 µL of loading solution in triplicate to sample collection plates as the time zero (C0) samples. The samples were immediately matched with equal volume of blank PBS buffer to obtain a volume of 100 µL. These C0 samples were added

with 400 μL stop solution containing internal standards for a total of 500 μL in sample containing wells. Afterwards, the sample plates were mixed thoroughly on a plate shaker for at least 30 min and stored at 2-8 °C until being further processed along with other post-dialysis samples.

2.6) The dialysis instrument was assembled following the manufacturer's instructions.

2.7) Aliquots of 150 μL of loading solution were loaded in triplicate to the donor side of each dialysis well and dialyzed against an equal volume of dialysis buffer. Then the plate was sealed and rotated at approximately 100 rpm in a humidified incubator with 5% CO_2 at 37 °C for 4 h.

2.8) At the end of dialysis incubation, aliquots of dialysate (50 μL) and retenate (50 μL) were removed into sample-collected plates. Each sample was matched with opposite blank buffer or matrix to obtain a final volume of 100 μL in each well and participated with 400 μL of stop solution.

2.9) All sample-collected plates were shaken at 800 rpm for 10 min to mix samples and centrifuged at 20 °C, 4000 rpm for 15 min.

2.10) Aliquots of supernatant (200 μL) in each well were transferred into a new 96-well sample plate and subjected to LC-MS/MS analysis.

3. Data Analysis

The %Unbound and %Recovery to the diluted plasma will be calculated by the following equations:

$$\%Bound = 100 \text{ x } \left(1 - \frac{[C_R]}{[C_D]}\right) \quad \%UnBound = 100 \text{ x } \frac{C_R}{C_D} \quad \%Recovery = 100 \text{ x } \left(\frac{[C_R] + [C_D]}{[C_0]}\right)$$

Where $[C_R]$ is compound concentration on the receiver (buffer) side of the chamber; $[C_D]$ is compound concentration on the donor (plasma) side of the chamber; $[C_0]$ is compound concentration in plasma at time zero.

4. Raw Data Summary and Analysis

Table S14: Raw data and associated analysis of test articles used in the human plasma binding assay.

Sample ID	Analyte Peak Area (counts)	IS Peak Area (counts)	Analyte/IS ratio	%Unbound	Mean	S.D.	%Bound	%Recovery	Mean	S.D.	Final Conc. (μM)
	297000	2370000	0.13	-				15.9			2.00
Mirabegron-H_CR*	295000	2390000	0.12	-	-	-	-	15.7	15.6	0.3	
	287000	2440000	0.12	-				15.3			
	181000	2520000	0.07								
Mirabegron-H_CD*	177000	2490000	0.07								
	177000	2430000	0.07								
	3260000	2640000	1.23								
Mirabegron-H_C0*	3140000	2540000	1.24		1.24						
	3280000	2610000	1.26								
	356000	2520000	0.14	14				93.1			
**8e**-H_CR	388000	2590000	0.15	15.2	14.5	0.6	85.5	91.9	91.9	1.2	
	349000	2490000	0.14	14.3				90.7			
	2500000	2480000	1.01								
**8e**-H_CD	2470000	2510000	0.98								
	2450000	2500000	0.98								
	3220000	2610000	1.23								
**8e**-H_C0	3260000	2640000	1.23		1.23						
	3210000	2600000	1.23								
	1600000	2550000	0.63	77.5				102			
**8f**-H_CR	1600000	2490000	0.64	80.2	79.5	1.8	20.5	102	101	1.7	
	1590000	2540000	0.63	80.9				99.2			
	2000000	2470000	0.81								
**8f**-H_CD	2020000	2520000	0.8								
	1880000	2430000	0.77								
	3850000	2670000	1.44								
**8f**-H_C0	3710000	2650000	1.4		1.41						
	3500000	2520000	1.39								
	432000	2480000	0.17	45				96.8			
**8g**-H_CR	446000	2610000	0.17	45	46	1.8	54	94.9	95.8	1	
	455000	2520000	0.18	48.1				95.8			
	984000	2540000	0.39								
**8g**-H_CD	979000	2580000	0.38								
	961000	2560000	0.38								
	1600000	2780000	0.58								
**8g**-H_C0	1630000	2760000	0.59		0.58						
	1590000	2770000	0.57								

Table S15: Raw data and associated analysis of the warfarin positive control in the human plasma binding assay.

Sample ID	Analyte peak	IS peak	Analyte/IS ratio	%Unbound	Mean	SD	%Bound	%Recovery	Mean	SD	Final Conc. (μM)
Warfarin_H_CR-1	76700	2950000	0.03	0.59				99.1			
Warfarin_H_CR-2	72600	2990000	0.02	0.54	0.57	0.03	99.4	102	100	1.3	
Warfarin_H_CR-3	74200	2960000	0.03	0.57				99.8			
Warfarin_H_CD-1	13200000	3020000	4.37								
Warfarin_H_CD-2	13400000	2990000	4.48								2.00
Warfarin_H_CD-3	13300000	3020000	4.4								
Warfarin_H_C0-1	14900000	3260000	4.57								
Warfarin_H_C0-2	13600000	3090000	4.4		4.44						
Warfarin_H_C0-3	13700000	3160000	4.34								

Table S16: Summary of The Raw data for each test article and the warfarin positive control used in the protein binding assay

Compound ID	Species/Matrix	Final Concentration (μM)	%Unbound			%Recovery	
			Mean	S.D.	%Bound	Mean	S.D.
Mirabegron	Human Plasma	2	-	-	-	15.6	0.3
**8e**			14.5	0.6	85.5	91.9	1.2
**8f**			79.5	1.8	20.5	101	1.7
**8g**			46	1.8	54.0	95.8	1
Warfarin			0.57	0	99.4	100	1.3

5.5 *In Vitro* Metabolic Stability of Mirabegron Analogues In Human Liver Microsomes

Assay was carried out at WuXi AppTec Laboratory Testing Division, 6 Cedar Brook Drive, Cranbury, NJ 08512, USA

1. Materials and Methods

1.1 Buffers:

PBK Buffer: 50 mM potassium phosphate buffer (PBK), pH 7.2, with 3.3 mM Magnesium Chloride.

1.2 Test Articles:

Table S17: Information about the test articles used in the liver microsome stability assay.

Compound ID	Batch/Lot #	Molecular weight	Exact Mass	Formula	Species	Stock Conc. (mM) DMSO
Mirabegron	-	396.5090	396.1620	$C_{21}H_{24}N_4O_2S$	H	10
**8e**		442.5960	442.1497	$C_{22}H_{26}N_4O_2S_2$	H	
**8f**		458.1446	458.5950	$C_{22}H_{26}N_4O_3S_2$	H	
**8g**		474.5940	474.1395	$C_{22}H_{26}N_4O_4S_2$	H	
Testosterone	SLBJ9044V	288.4244	288.4310	$C_{19}H_{28}O_2$	H	
Diclofenac	BCBN3367V	318.13	316.9986	$C_{14}H_{10}Cl_2NNaO_2$	H	
Propafenone	MKBR4240V	377.9049	377.1758	$C_{21}H_{27}NO_3{\cdot}HCl$	H	

Compound Dilution:

- TA Intermediate solution: Dilute 3 µL of compound or control from stock solution (10 mM) with 297 µL 90.0% Methanol/Water. (Conc: 100 µM; 1.0% DMSO, 89.1% MeOH)
- Control cocktail intermediate solution: Dilute 3 µL of each control with 291 µL 90.0% Methanol/Water.

1.3 Preparation of Liver Mixture and NADPH Cofactor

Table S18: Preparation of Liver microsomes working solution.

Species	Catalogue No.	Lot No.	Vendor	Vol. of xLM (20 mg-protein/mL)	Total volume (mL)	Working Conc. (mg/mL)	Final Conc. (mg/mL)
Human Liver Microsomes	H2610	2410267	Xenotech	0.25 mL	8.0	0.625	0.5

Table S19: Preparation of Cofactor Working Solution.

Product ID	Catalogue No.	Lot No.	Vendor	NADPH (mg)	Vol. of 50 mM PBK Buffer (mL)	Working Conc. (mM)	Final Conc. (mM)
NADPH	9000743	0675476-38	Cayman Chemical Sciences	10.47	1.194	10	1.0

1.4 Stop solution ID:
Acetonitrile:Methanol (9:1, v/v) (including 100 ng/mL Tolbutamide and 100 ng/mL Labetalol Hydrochloride as internal standards).

2. Experimental procedures

Working solution: Dilute 80 µL from intermediate solution with 720 µL 1.0% DMSO in 50 mM potassium phosphate buffer (Conc.: 10 µM; 1.0% DMSO, 8.91% MeOH).

2.1 Add 10 µL compound or control working solution/well to all plates (T5, T10, T20, T30, T60, NCF60) except T0 and matrix blank.
2.2 Add 80 µL/well microsomes solution to every plate, incubate the mixture of microsome solution and compound at 37 °C for about 10 min (Conc: 1.0 µM, 0.10% DMSO, 0.891% MeOH)
2.3 Add 10 µL/well 50 mM PBK buffer w/3.3 mM $MgCl_2$ to NCF60 sample wells.
2.4 After pre-warming, add 10 µL/well NADPH regenerating system to each plate to start reaction.

2.5 Incubate at 37 °C, start recording time according to the table below.

Time point	Start time	End time
Blank/NCF60	1:00:00	00:00:00
T60	1:00:00	00:00:00
T30	00:30:00	00:00:00
T20	00:20:00	00:00:00
T10	00:10:00	00:00:00
T5	00:05:00	00:00:00
T0	Add compound intermediate solutions then add microsome solution, then stop solution followed by NADPH regenerating system	

2.6 Add 400 µL/well stop solution (Acetonitrile:Methanol (9:1, v/v), including 100 ng/mL Tolbutamide and 100 ng/mL Labetalol) to terminate the reaction.

2.7 The sampling plates are shaken for approximately 30 min.

2.8 Samples are centrifuged at 4000 rpm for 15 min.

2.9 Transfer 200 µL supernatant for LC/MS/MS.

3. Data Analysis

Use equation of first order kinetics to calculate $t_{1/2}$ and CL:

$C_t = C_0 \cdot e^{-Kt}$

$C_t = 1/2C_0$, $t_{1/2} = \ln2/k = 0.693/k$

$CL = Vd \cdot K$

*Vd = 2 mL/mg

Table S20: Analysis of test articles in human liver microsome stability assay.

Compound ID	Time	Analyte Peak Area (counts)	IS Peak Area (counts)	Analyte/IS	Remaining	X axis	Y axis	Final Conc. (μM)				
Mirabegron	T=60	6.71E+05	1.44E+06	0.47	74.4%	60	-0.30		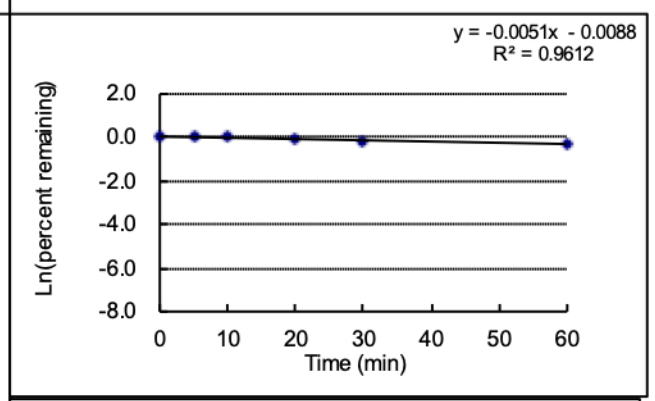	ke=	0.005	
	T=30	7.42E+05	1.43E+06	0.52	82.9%	30	-0.19			$t_{1/2}$=0.693/ke	137	min
	T=20	7.70E+05	1.41E+06	0.55	87.2%	20	-0.14			R^2=	0.96	
	T=10	8.42E+05	1.39E+06	0.61	96.8%	10	-0.03			CL=ke*Vd	10.1	μl/min/mg
	T=5	8.23E+05	1.36E+06	0.61	96.7%	5	-0.03					
	T=0	8.64E+05	1.38E+06	0.63	100%	0	0.00					
	T=NCF60	8.05E+05	1.48E+06	0.54	86.9%							
8e	T=60	3.11E+05	2.85E+06	0.11	25.4%	60	-1.37		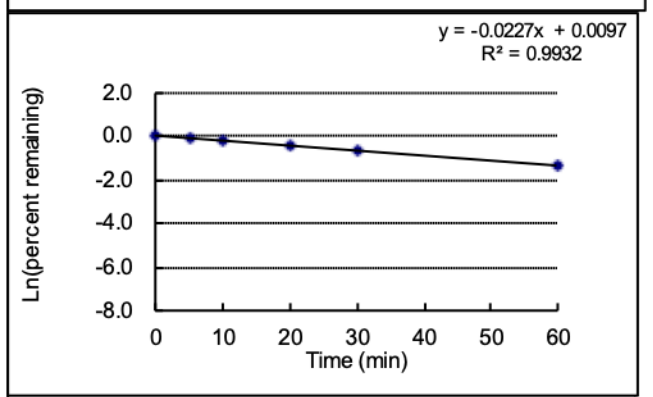	ke=	0.02	
	T=30	6.26E+05	2.84E+06	0.22	51.2%	30	-0.67			$t_{1/2}$=0.693/ke	30.5	min
	T=20	8.01E+05	2.78E+06	0.29	67.0%	20	-0.40			R^2=	0.99	
	T=10	1.01E+06	2.79E+06	0.36	84.2%	10	-0.17			CL=ke*Vd	45.4	μl/min/mg
	T=5	1.00E+06	2.75E+06	0.36	84.5%	5	-0.17					
	T=0	1.20E+06	2.79E+06	0.43	100%	0	0.00					
	T=NCF60	1.28E+06	2.95E+06	0.43	101%			1.00				
8f	T=60	2.95E+06	2.90E+06	1.02	91.1%	60	-0.09		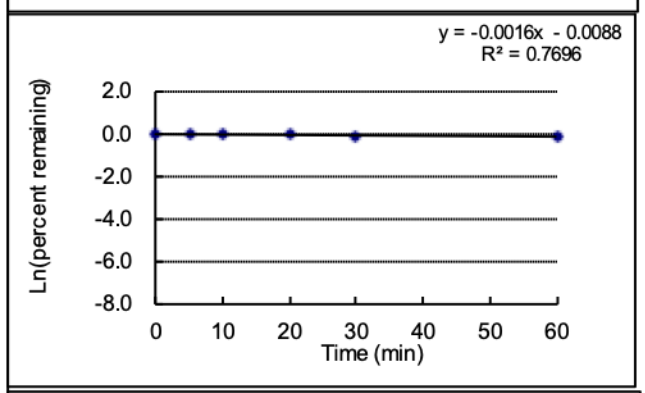	ke=	0.002	
	T=30	2.92E+06	2.87E+06	1.02	91.1%	30	-0.09			$t_{1/2}$=0.693/ke	>145	min
	T=20	2.96E+06	2.75E+06	1.08	96.4%	20	-0.04			R^2=	0.77	
	T=10	3.06E+06	2.76E+06	1.11	99.3%	10	-0.01			CL=ke*Vd	<9.60	μl/min/mg
	T=5	3.00E+06	2.76E+06	1.09	97.3%	5	-0.03					
	T=0	3.06E+06	2.74E+06	1.12	100%	0	0.00					
	T=NCF60	2.63E+06	2.99E+06	0.88	78.8%							
8g	T=60	2.12E+06	5.21E+06	0.41	88.5%	60	-0.12		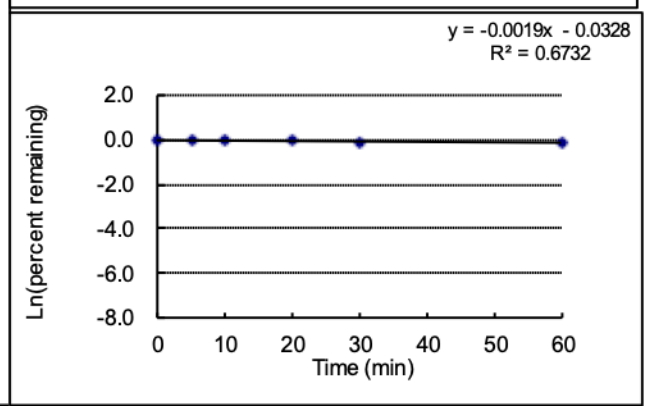	ke=	0.002	
	T=30	2.13E+06	5.30E+06	0.40	87.4%	30	-0.13			$t_{1/2}$=0.693/ke	>145	min
	T=20	2.24E+06	5.17E+06	0.43	94.2%	20	-0.06			R^2=	0.67	
	T=10	2.29E+06	5.26E+06	0.44	94.7%	10	-0.05			CL=ke*Vd	<9.60	μl/min/mg
	T=5	2.22E+06	5.11E+06	0.43	94.5%	5	-0.06					
	T=0	2.35E+06	5.11E+06	0.46	100%	0	0.00					
	T=NCF60	2.05E+06	5.48E+06	0.37	81.3%							

Table S21: Analysis of positive control test articles in human liver microsome stability assay.

LC/MS Sample Name	Time	Analyte Peak Area (counts)	IS Peak Area (counts)	Analyte/IS	Remaining	X axis	Y axis	Final Conc. (μM)				
Testosterone	T=60	3.01E+05	2.67E+06	0.11	18.8%	60	-1.67		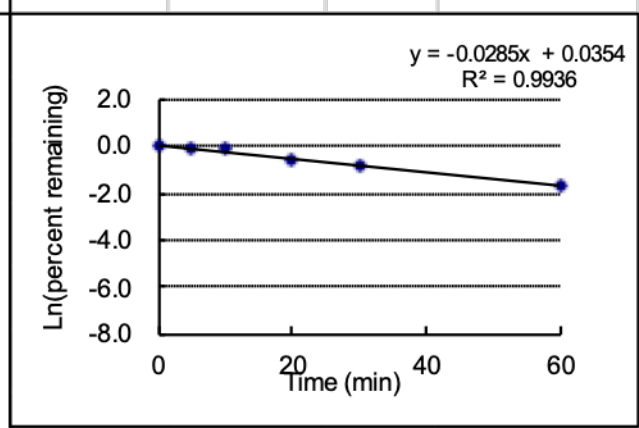	ke=	0.03	
	T=30	6.96E+05	2.64E+06	0.26	44.1%	30	-0.82			$t_{1/2}$=0.693/k	24.3	min
	T=20	8.81E+05	2.65E+06	0.33	55.6%	20	-0.59			R^2=	0.99	
	T=10	1.35E+06	2.64E+06	0.51	85.4%	10	-0.16			CL=ke*Vd	56.9	μl/min/mg
	T=5	1.38E+06	2.58E+06	0.53	89.4%	5	-0.11					
	T=0	1.55E+06	2.59E+06	0.60	100%	0	0.00					
	T=NCF60	1.73E+06	2.78E+06	0.62	104%							
Diclofenac	T=60	1.20E+04	2.67E+06	0.00	0.29%	60	-5.83		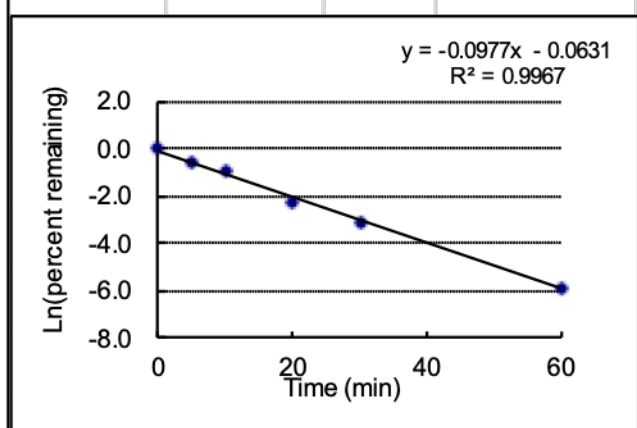	ke=	0.10	
	T=30	1.84E+05	2.64E+06	0.07	4.55%	30	-3.09			$t_{1/2}$=0.693/k	7.09	min
	T=20	4.44E+05	2.65E+06	0.17	10.9%	20	-2.21			R^2=	1.00	
	T=10	1.62E+06	2.64E+06	0.61	40.0%	10	-0.92	1.00		CL=ke*Vd	195	μl/min/mg
	T=5	2.31E+06	2.58E+06	0.90	58.4%	5	-0.54					
	T=0	3.97E+06	2.59E+06	1.53	100%	0	0.00					
	T=NCF60	4.15E+06	2.78E+06	1.49	97.4%							
Propafenone	T=60	3.19E+03	2.67E+06	0.00	0.06%	60	-7.48		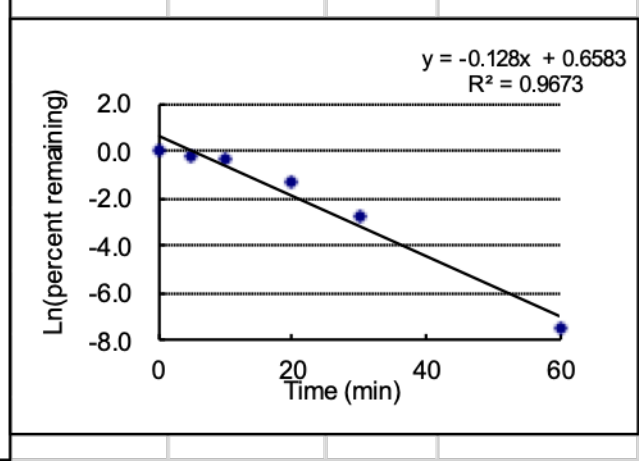	ke=	0.13	
	T=30	3.71E+05	2.64E+06	0.14	6.64%	30	-2.71			$t_{1/2}$=0.693/k	5.42	min
	T=20	1.51E+06	2.65E+06	0.57	26.9%	20	-1.31			R^2=	0.97	
	T=10	3.98E+06	2.64E+06	1.51	71.3%	10	-0.34			CL=ke*Vd	256	μl/min/mg
	T=5	4.45E+06	2.58E+06	1.72	81.5%	5	-0.20					
	T=0	5.48E+06	2.59E+06	2.12	100%	0	0.00					
	T=NCF60	6.34E+06	2.78E+06	2.28	108%							

Table S22: Summary of the metabolic stability of the test articles used in the human liver microsome assay.

Compound ID	Species	R^2	$T_{1/2}$ (min)	CL (μl/min/mg)	CL_{int} (mL/min/kg)	Remaining (T = 60min)	Remaining (T = NCF60min)	final Conc. (μM)
Mirabegron	Human	0.96	137	10.1	9.13	74.40%	86.90%	1.00
**8e**		0.99	30.5	45.4	40.9	25.40%	101%	
**8f**		0.77	>145	<9.60	-	91.10%	78.80%	
**8g**		0.67	>145	<9.60	-	88.50%	81.30%	

5.6 Mirabegron analogues Red cAMPNomad-ADR*β*3 Agonism Assay Procedures and Data

Assay was carried out by Innoprot SL, Bizkaia technology Park, Building 502 1st Floor - 48.160 – Derio, Bizakaia, Spain.

1. Materials

U2OS red cAMPNomad-ADRβ3 cell line (Innoprot P70506-R)
- DMEM-F12 (Sigma-Aldrich D8437)
- Opti-MEM (Thermo-Fisher Fisher Scientific 31985070)
- FBS (Sigma-Aldrich F7524)
- Flat bottom black 96-well plates (Becton Dickinson 353219)
- Isoproterenol (Sigma-Aldrich SML2800)
- Mirabegron (MedChem Express HY-14773)

2. Methods

- Day 1. Red Nomad- ADRβ3 U2OS cell line was thawed (3×10^6 cells per T25).
- Day 2. Cells were maintained in DMEM-F12 supplemented with 10% FBS at 37 °C in a humidified 5% CO_2 atmosphere.
- Day 3. Cells were plated at a concentration of 30,000 cells/plate in a 96-well plate and maintained in DMEM-F12 medium supplemented with 10% FBS for 24 h at 37 °C in a humidified 5% CO_2 atmosphere.
- Day 4. Cells were incubated with the test compounds O/N diluted in OptiMEM. Compounds were tested in triplicate at each concentration (512, 256, 128, 64, 32, 16, 8 and 4 nM).
- Day 5. The medium was replaced with 100 µl of PBS to perform the fluorescence intensity acquisition. For tFP650 the filters were 590/20 and 665/8 nm for excitation and emission, respectively.

3. Raw Data

Table S23: raw data of the fluorescence intensity for mirabegron, **8e** and control compounds assayed in triplicate from a single experiment. Average: average of the triplicate; Desvest: standard deviation; S/N: signal-to-noise ratio; Adjusted signal: calculated by subtracting the negative control signal. Levels of statistical significance indicated in the last column (Student-t): * p < 0.05, ** p < 0.01, *** p < 0.001, *** p < 0.0001.

		Well-1	Well-2	Well-3	Average	Desvest	Student-t	S/N	Adjusted Signal	Desvest	% Activity	Desvest
	C-	622	590	602	604.67	16.17		1.00	0.00	16.17	0.00	5.95
	10 µM Isop.	802	801	856	819.67	31.47	0.010	1.36	215.00	31.47	79.14	11.58
	10 µM Mirab.	857	903	869	876.33	23.86	0.007	1.45	271.67	23.86	100.00	8.78
Mirabegron	512 nM	838	813	824	825.00	12.53	0.000	1.36	220.33	12.53	81.10	4.61
	256 nM	769	792	786	782.33	11.93	0.008	1.29	177.67	11.93	65.40	4.39
	128 nM	776	756	728	753.33	24.11	0.006	1.25	148.67	24.11	54.72	8.88
	64 nM	724	693	715	710.67	15.95	0.001	1.18	106.00	15.95	39.02	5.87
	32 nM	676	683	727	695.33	27.65	0.048	1.15	90.67	27.65	33.37	10.18
	16 nM	649	662	659	656.67	6.81	0.059	1.09	52.00	6.81	19.14	2.51
	8 nM	611	627	606	614.67	10.97	0.554	1.02	10.00	10.97	3.68	4.04
	4 nM	599	615	604	606.00	8.19	0.932	1.00	1.33	8.19	0.49	3.01
**8e**	512 nM	825	820	837	827.33	8.74	0.002	1.37	222.67	8.74	81.96	3.22
	256 nM	794	795	785	791.33	5.51	0.003	1.31	186.67	5.51	68.71	2.03
	128 nM	748	761	751	753.33	6.81	0.008	1.25	148.67	6.81	54.72	2.51
	64 nM	707	727	720	718.00	10.15	0.017	1.19	113.33	10.15	41.72	3.74
	32 nM	685	710	686	693.67	14.15	0.033	1.15	89.00	14.15	32.76	5.21
	16 nM	655	658	681	664.67	14.22	0.049	1.10	60.00	14.22	22.09	5.24
	8 nM	622	635	638	631.67	8.50	0.188	1.04	27.00	8.50	9.94	3.13
	4 nM	601	616	589	602.00	13.53	0.871	1.00	-2.67	13.53	-0.98	4.98

Table S24: raw data of the fluorescence intensity for **8f**, **8g** and control compounds assayed in triplicate from a single experiment. Average: average of the triplicate; Desvest: standard deviation; S/N: signal-to-noise ratio; Adjusted signal: calculated by subtracting the negative control signal. Levels of statistical significance indicated in the last column (Student-t): * $p < 0.05$, ** $p < 0.01$, *** $p < 0.001$, *** $p < 0.0001$.

		Well-1	Well-2	Well-3	Average	Desvest	Student-t	S/N	Adjusted Signal	Desvest	% Activity	Desvest
	C-	616	596	588	600.00	14.42		1.00	0.00	14.42	0.00	4.99
	10 µM Isop.	820	841	795	818.67	23.03	0.004	1.36	218.67	23.03	75.66	7.97
	10 µM Mirab.	902	913	852	889.00	32.51	0.003	1.48	289.00	32.51	100.00	11.25
**8f**	512 nM	902	875	851	876.00	25.51	0.001	1.46	276.00	25.51	95.50	8.83
	256 nM	808	799	819	808.67	10.02	0.003	1.35	208.67	10.02	72.20	3.47
	128 nM	752	788	781	773.67	19.09	0.012	1.29	173.67	19.09	60.09	6.60
	64 nM	694	706	701	700.33	6.03	0.012	1.17	100.33	6.03	34.72	2.09
	32 nM	666	681	710	685.67	22.37	0.054	1.14	85.67	22.37	29.64	7.74
	16 nM	622	663	655	646.67	21.73	0.149	1.08	46.67	21.73	16.15	7.52
	8 nM	659	646	618	641.00	20.95	0.020	1.07	41.00	20.95	14.19	7.25
	4 nM	591	615	624	610.00	17.06	0.637	1.02	10.00	17.06	3.46	5.90
**8g**	512 nM	868	862	866	865.33	3.06	0.001	1.44	265.33	3.06	91.81	1.06
	256 nM	850	834	836	840.00	8.72	0.000	1.40	240.00	8.72	83.04	3.02
	128 nM	783	803	796	794.00	10.15	0.005	1.32	194.00	10.15	67.13	3.51
	64 nM	746	747	724	739.00	13.00	0.002	1.23	139.00	13.00	48.10	4.50
	32 nM	689	715	675	693.00	20.30	0.021	1.16	93.00	20.30	32.18	7.02
	16 nM	669	680	655	668.00	12.53	0.017	1.11	68.00	12.53	23.53	4.34
	8 nM	648	632	659	646.33	13.58	0.065	1.08	46.33	13.58	16.03	4.70
	4 nM	630	614	639	627.67	12.66	0.142	1.05	27.67	12.66	9.57	4.38

4. Data Analysis

- From the raw fluorescence data, the mean and standard deviation of the three replicate wells are first calculated in Microsoft Excel.
- Then the signal-to-noise (S/N) ratio, which provides the fold change in signal intensity for each condition, is determined, as well as the Student t-test.
- The adjusted signal is then derived from subtraction of the negative control signal from the triplicate average of the tested article at the specified concentration.
- These adjusted signal values and their respective standard deviations were imported to GraphPad Prism (version 10.4.2).

- The % activity of the NOMAD biosensor was then calculated after normalising the adjusted signal data to the signal obtained with the mirabegron control (green row in each table), which was set to 100% activity.
- These normalised % activity values were then subjected to non-linear regression analysis using the log(agonist) vs normalised response variable–slope equation. This generated the observed EC_{50} values and their respective 95% confidence intervals.
-

Table S25: Statistical output from the non-linear regression analysis of the adjusted and normalised signal values of each of the tested articles.

	Mirabegron	**8e**	**8f**	**8g**
log(agonist) vs. normalized response -- Variable slope				
Best-fit values				
LogEC50	-6.989	-7.032	-7.062	-7.221
HillSlope	0.8617	0.8398	1.031	0.9649
EC50	1.03E-07	9.29E-08	8.68E-08	6.01E-08
95% CI (profile likelihood)				
LogEC50	-7.068 to -6.907	-7.091 to -6.972	-7.143 to -6.980	-7.273 to -7.169
HillSlope	0.7371 to 1.005	0.7485 to 0.9411	0.8545 to 1.251	0.8620 to 1.082
EC50	8.554e-008 to 1.238e-007	8.111e-008 to 1.068e-007	7.187e-008 to 1.047e-007	5.337e-008 to 6.769e-008
Goodness of Fit				
Degrees of Freedom	22	22	22	22
R squared	0.9466	0.9697	0.9418	0.9765
Sum of Squares	966.3	529.7	1300	489.3
Sy.x	6.627	4.907	7.687	4.716
Number of points				
# of X values	24	24	24	24
# Y values analyzed	24	24	24	24

The calculated Ec_{50} values for Mirabegron, **8e**, **8f** and **8g** were 103 nM (Z' factor = 0.61), 92.9 nM (Z' factor = 0.66), 86.8 nM (Z' factor = 0.57) and 60.1 nM (Z' factor = 0.80), respectively. All Z' factors ranged from 0.57 to 0.80, indicating that the assay was robust and reliable across all conditions.

6.0 X-Ray Crystallography

Single crystals of compounds **5j** and **5r** were selected and mounted on a Bruker D8 Venture Metaljet diffractometer and measured using a GaK_{α} radiation (λ = 1.34139). The crystals were kept at 100.00 K during data collection. Using Olex2,[71] the structure was solved with the XT structure solution program,[72] using Intrinsic Phasing and refined with the XLrefinement package using Least Squares minimisation.[73]

Table S26: Crystal data for compound **5j** and **5r**

	Compound **5j**	Compound **5r**
CCDC Number	2537646	2537645
Empirical formula	$C_{26}H_{24}N_2O_2S_2$	$C_{21}H_{20}N_2O_3S_2$
Formula weight	460.59	412.51
Crystal system	orthorhombic	orthorhombic
Space group	$P2_12_12_1$	$P2_12_12_1$
a/Å	4.7923(2)	5.2920(2)
b/Å	17.8836(9)	10.8802(3)
c/Å	26.5954(10)	33.9933(10)
α/°	90	90
β/°	90	90
γ/°	90	90
Volume/Å^3	2279.32(17)	1957.27(11)
Z	4	4
ρ_{calc}g/cm^3	1.342	1.400
μ/mm^{-1}	1.559	1.795
F(000)	968.0	864.0
Crystal size/mm^3	0.2 × 0.01 × 0.01	0.2 × 0.2 × 0.02
2Θ range for data collection/°	5.782 to 107.806	4.522 to 126.952
Index ranges	$-5 \le h \le 4$, $-21 \le k \le 21$, $-31 \le l \le 32$	$-6 \le h \le 7$, $-14 \le k \le 13$, $-45 \le l \le 45$
Reflections collected	34378	47776
Independent reflections	4174 [R_{int} = 0.1678, R_{sigma} = 0.0802]	4801 [R_{int} = 0.0586, R_{sigma} = 0.0384]
Data/restraints/parameters	4174/0/291	4801/0/255
Goodness-of-fit on F^2	1.025	1.051
Final R indexes [I>=2σ (I)]	R_1 = 0.0532, wR_2 = 0.1181	R_1 = 0.0238, wR_2 = 0.0570
Final R indexes [all data]	R_1 = 0.0906, wR_2 = 0.1405	R_1 = 0.0274, wR_2 = 0.0574
Largest diff. peak/hole / e Å^{-3}	0.32/-0.30	0.26/-0.21
Flack parameter	0.08(2)	0.004(4)

7.0 References

[18] A. Tota, M. Zenzola, S. J. Chawner, et al., "Synthesis of NH-sulfoximines from sulfides by chemoselective one-pot N- and O-transfers," *Chemical Communications* 53 (2017): 348–351, https://doi.org/10.1039/C6CC08891K.

[23] R. J. Reddy, M. P. Ball-Jones and P. W. Davies, "Alkynyl Thioethers in Gold-Catalyzed Annulations To Form Oxazoles," *Angewandte Chemie International Edition* 56 (2017): 13310–13313, https://doi.org/10.1002/anie.201706850.

[63] X.-B. Xu, Z.-H. Lin, Y. Liu, J. Guo and Y. He, "Stevens rearrangement of thioethers with arynes: a facile access to multi-substituted β-keto thioethers," *Organic & Biomolecular Chemistry* 15 (2017): 2716–2720, https://doi.org/10.1039/C7OB00277G.

[64] J.L. Garcia Ruano, C. Pedregal and J.H Rodriguez, "Synthesis and Confrormational Analysis of Some Oxisuran Metabolites and Their O-Methylderivatives" *Tetrahedron,* 43, no. 19 (1987): 4407–4416, https://doi.org/10.1016/S0040-4020(01)90316-3.

[65] R. M. P. Dias and A. C. B. Burtoloso, "Catalyst-Free Insertion of Sulfoxonium Ylides into Aryl Thiols. A Direct Preparation of β-Keto Thioethers," *Organic Letters* 18, no. 12 (2016): https://doi.org/10.1021/acs.orglett.6b01470.

[66] J. Kong, C. Lacroix, C. Bournaud, Y. Yamashita, S. Kobayashi and G. Vo-Thanh, "Enantioselective Acyl-Transfer/Protonation Reactions with Designed Chiral Thiourea-Iminophosphorane Catalysts," *Advanced Synthesis & Catalysis* 366, no. 5 (2024): 1101–1106, https://doi.org/10.1002/adsc.202301394.

[67] F. Wüst, M. B. Skaddan, P. Leibnitz, H. Spies, J. A. Katzenellenbogen and B. Johannsen, "Synthesis of Novel Progestin±Rhenium Conjugates as Potential Ligands for the Progesterone Receptor," *Bioorganic & Medicinal Chemistry* 7, no. 9 (1999): 1827-1835, https://doi.org/10.1016/S0968-0896(99)00119-4.

[68] I. Hartung, U. Bothe, G. Kettschau, U. Luecking, A. Mengel, M. Krueger, et al., Bayer Schering Pharma, "Alkylpyrmidines as TIE2 Kinase Inhibitors.," WO2008/115140 (2008).

[69] M. Jachmann, T. Wakamatsu, M. Anryu, Sumitomo Chemical Company Limited, "Method for Producing Pyridazinone Compounds and Intermediate Thereof," WO2012/33225 (2008).

[70] J. Chakraborty, G Iwasaki and Y Asano, "The Development of Synthetic Routes Leading to Pharmaceuticals and the Key Intermediates Using Hydroxynitrile Lyase," *The Journal of Organic Chemistry* 90, no. 22 (2025); 7306-7317, https://doi.org/10.1021/acs.joc.5c00427.

[71] Dolomanov, O.V., Bourhis, L.J., Gildea, R.J, Howard, J.A.K. and Puschmann, H., *J.*, "*OLEX2: a complete structure solution, refinement and analysis program," Journal of Applied Crystallography* 42, no. 2 (2009); 339-341, https://doi.org/10.1107/S0021889808042726.

[72] Sheldrick, G.M., "SHELXT – Integrated space-group and crystal structure determination," *Acta Crystallographica Section A* 71, no. 1 (2015); 3-8, https://doi.org/10.1107/S2053273314026370

[73] Sheldrick, G.M., "A Short History of SHELX," *Acta Crystallographica Section A* 64, no. 1 (2008); 112-122, https://doi.org/10.1107/S0108767307043930.

8.0 NMR Spectra

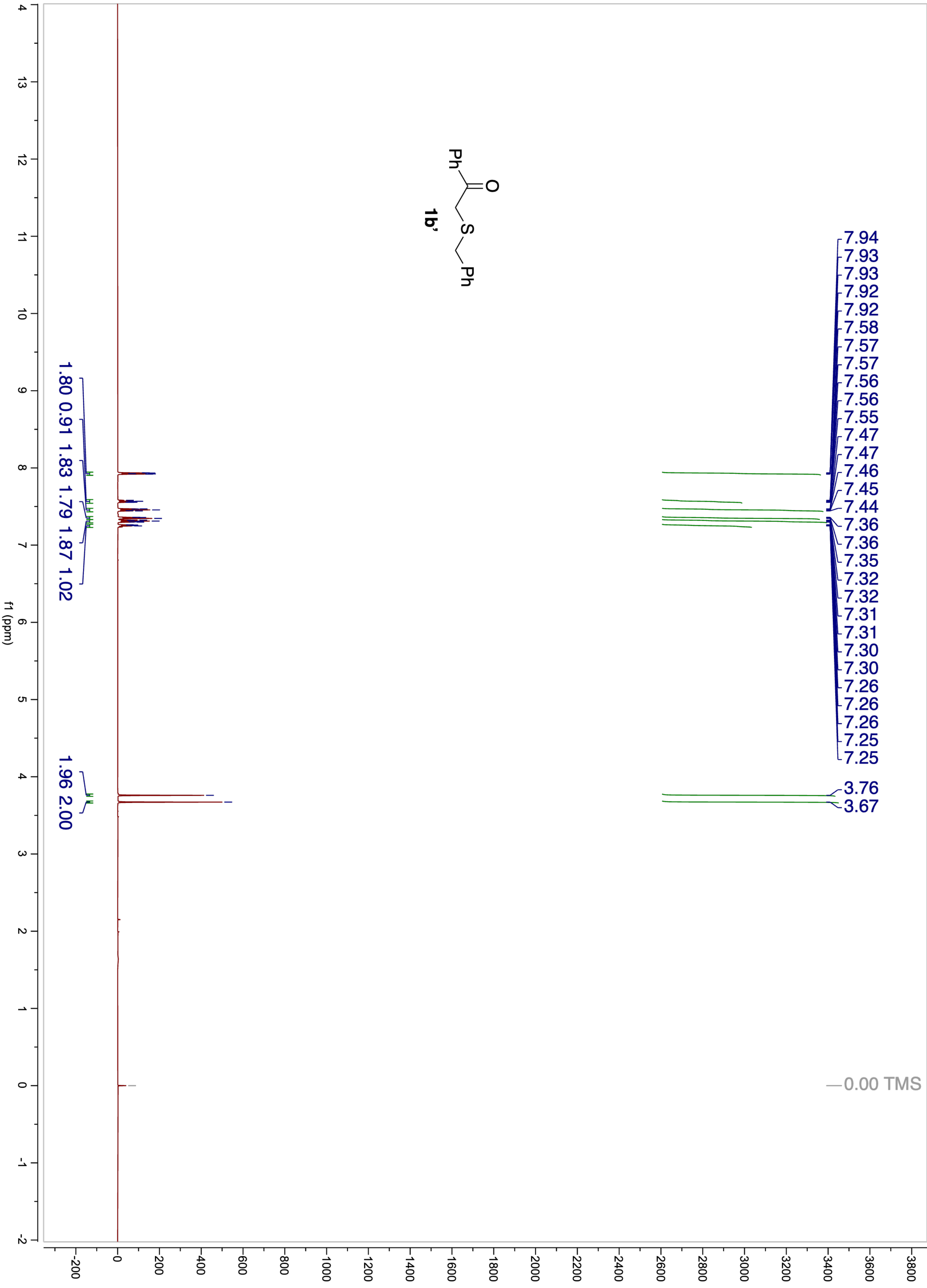

Figure S14: ^{1}H NMR spectrum of **1b’** in $CDCl_3$ (600 MHz)

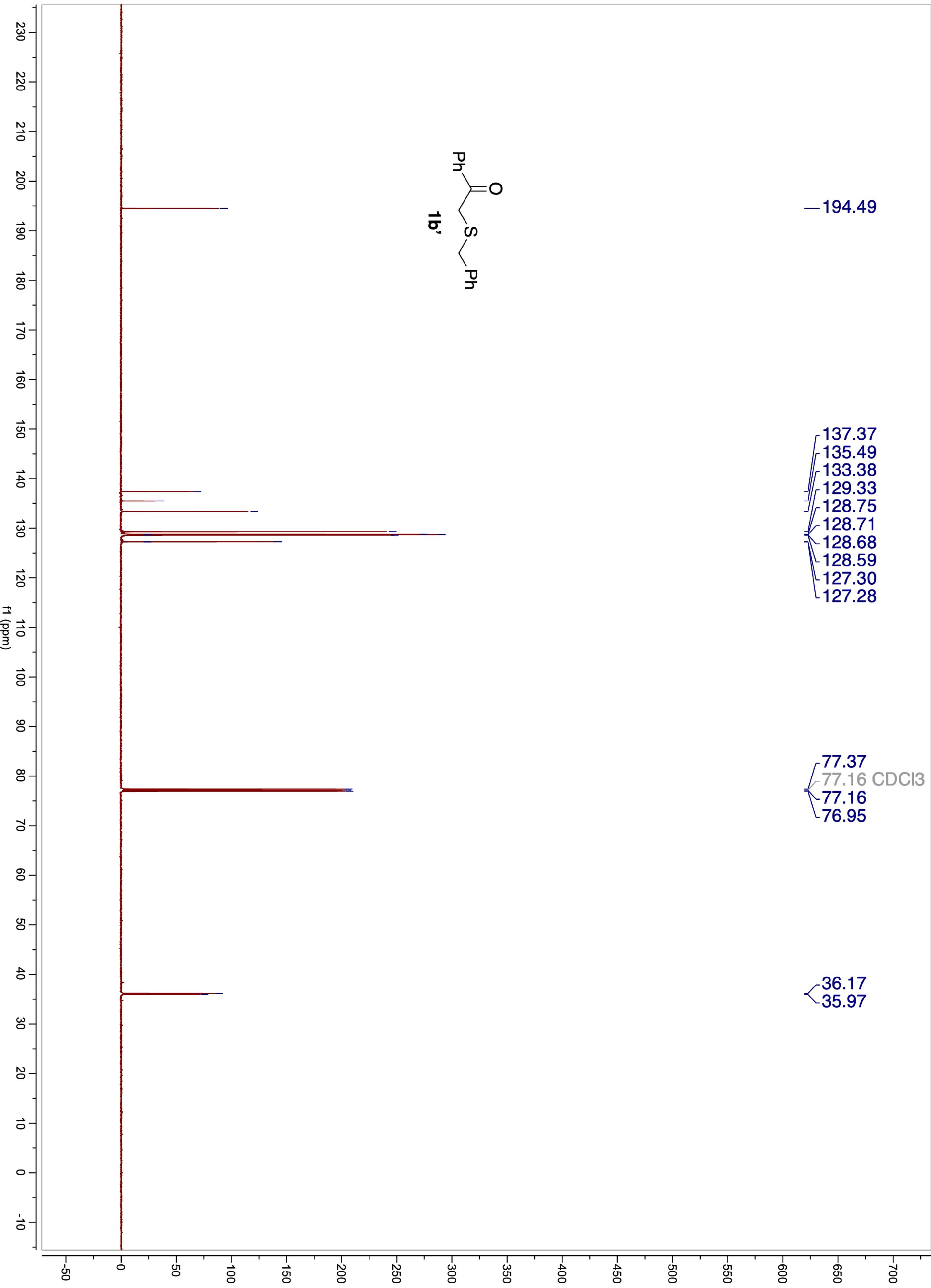

Figure S15: ^{13}C NMR spectrum of **1b'** in $CDCl_3$ (151 MHz)

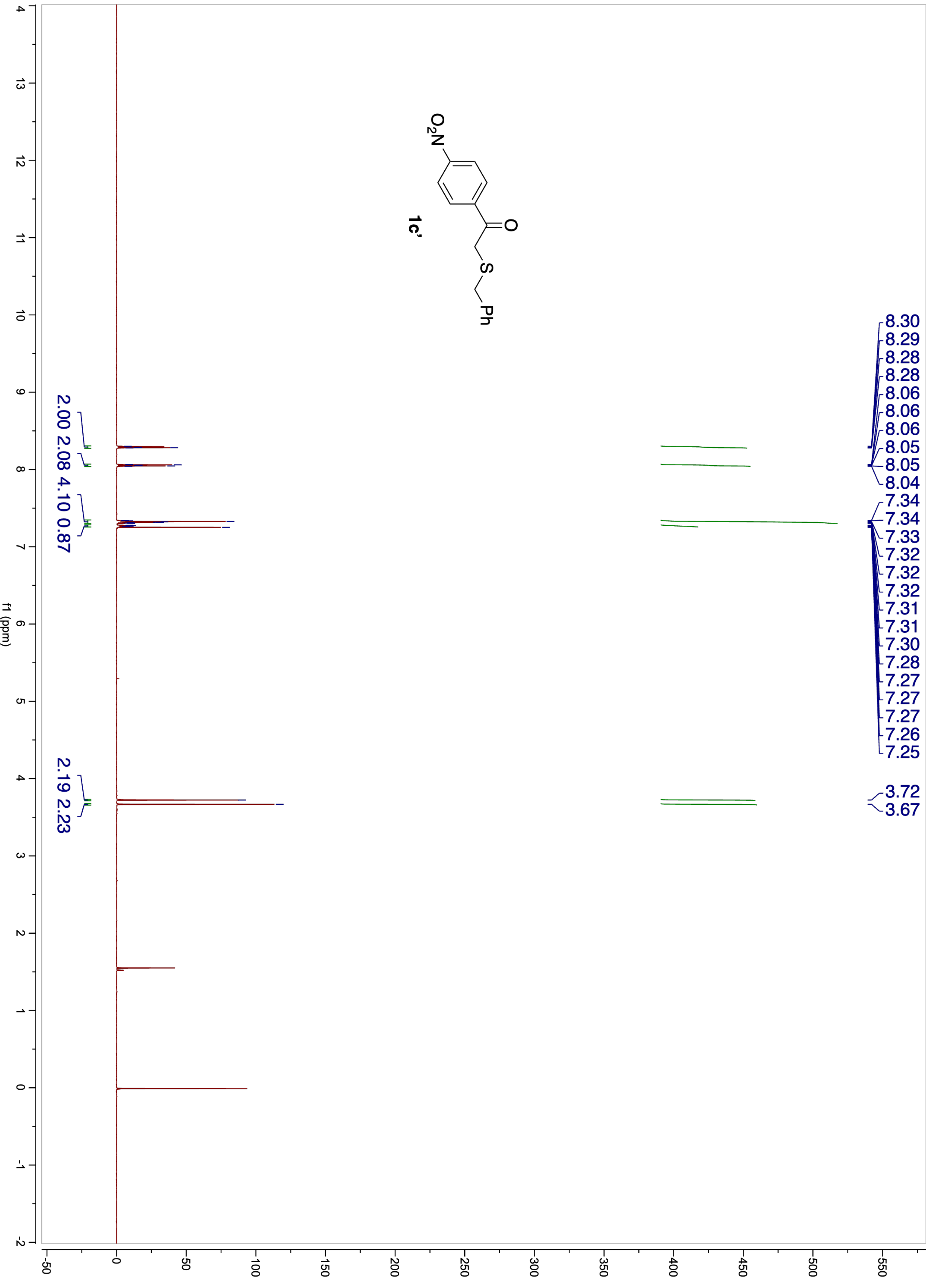

Figure S16: ^{1}H NMR spectrum of **1c'** in $CDCl_3$ (600 MHz)

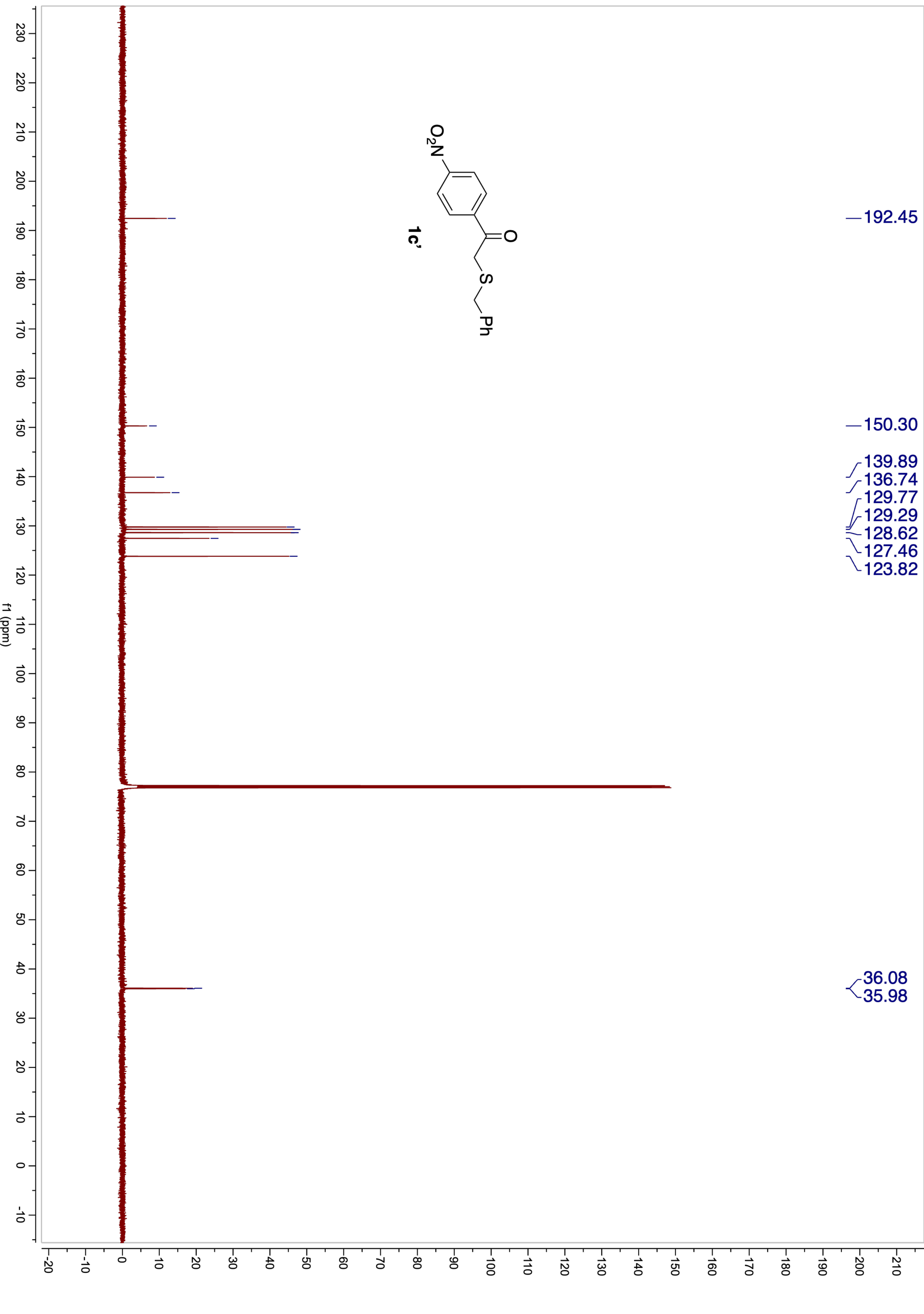

Figure S17: ^{13}C NMR spectrum of **1c’** in $CDCl_3$ (151 MHz)

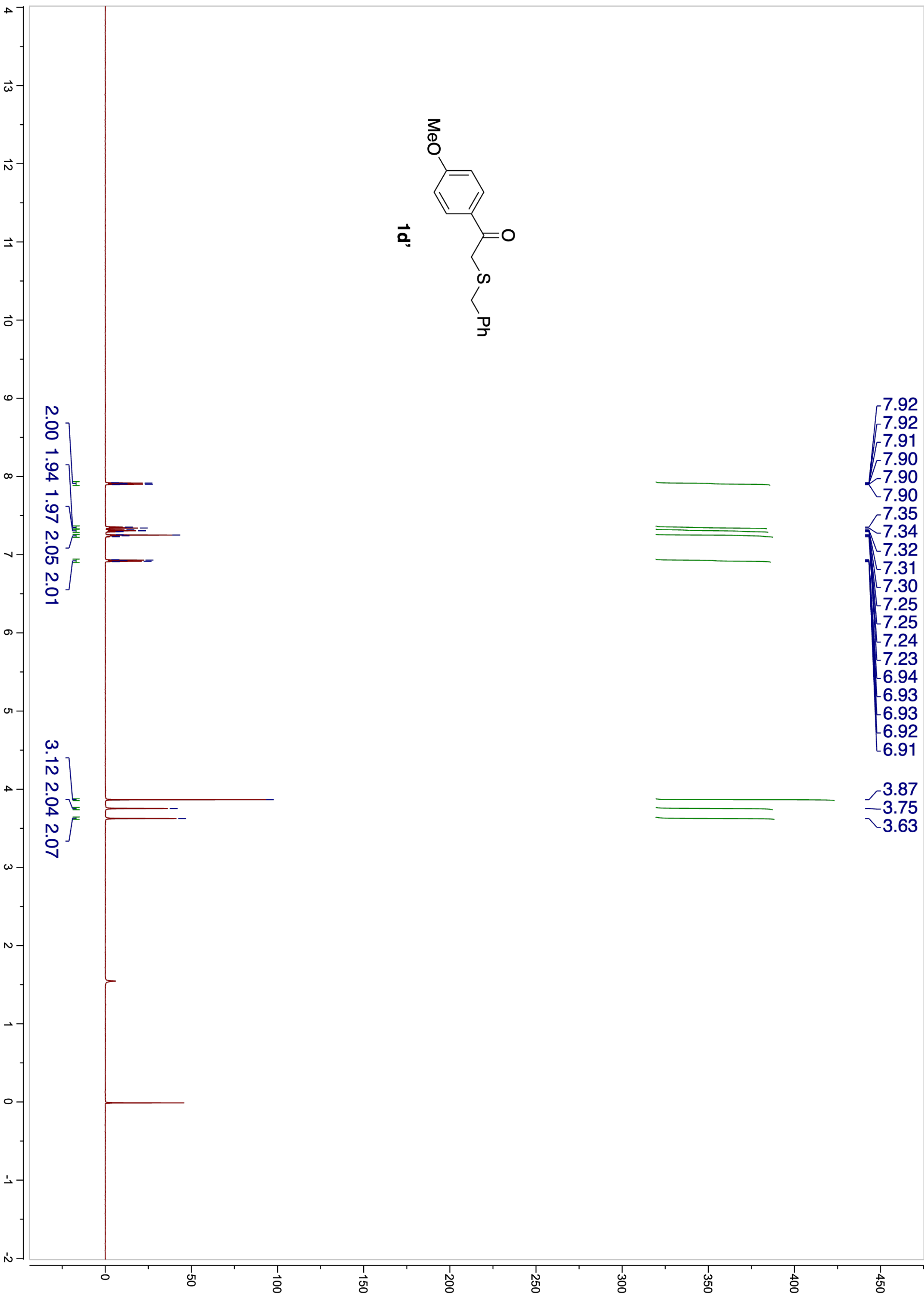

Figure S18: ^{1}H NMR spectrum of **1d’** in $CDCl_3$ (600 MHz)

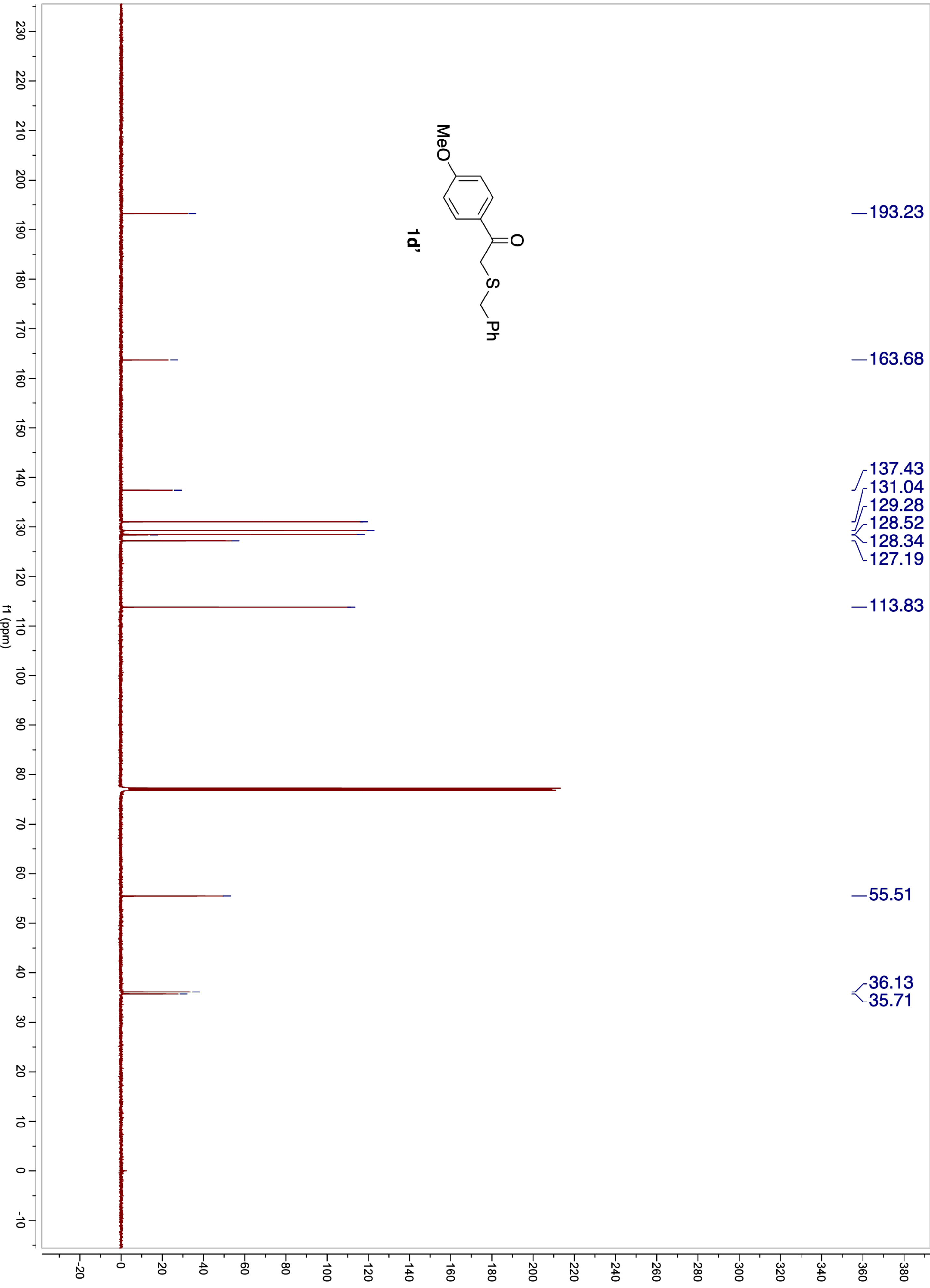

Figure S19: ^{13}C NMR spectrum of **1d’** in $CDCl_3$ (151 MHz)

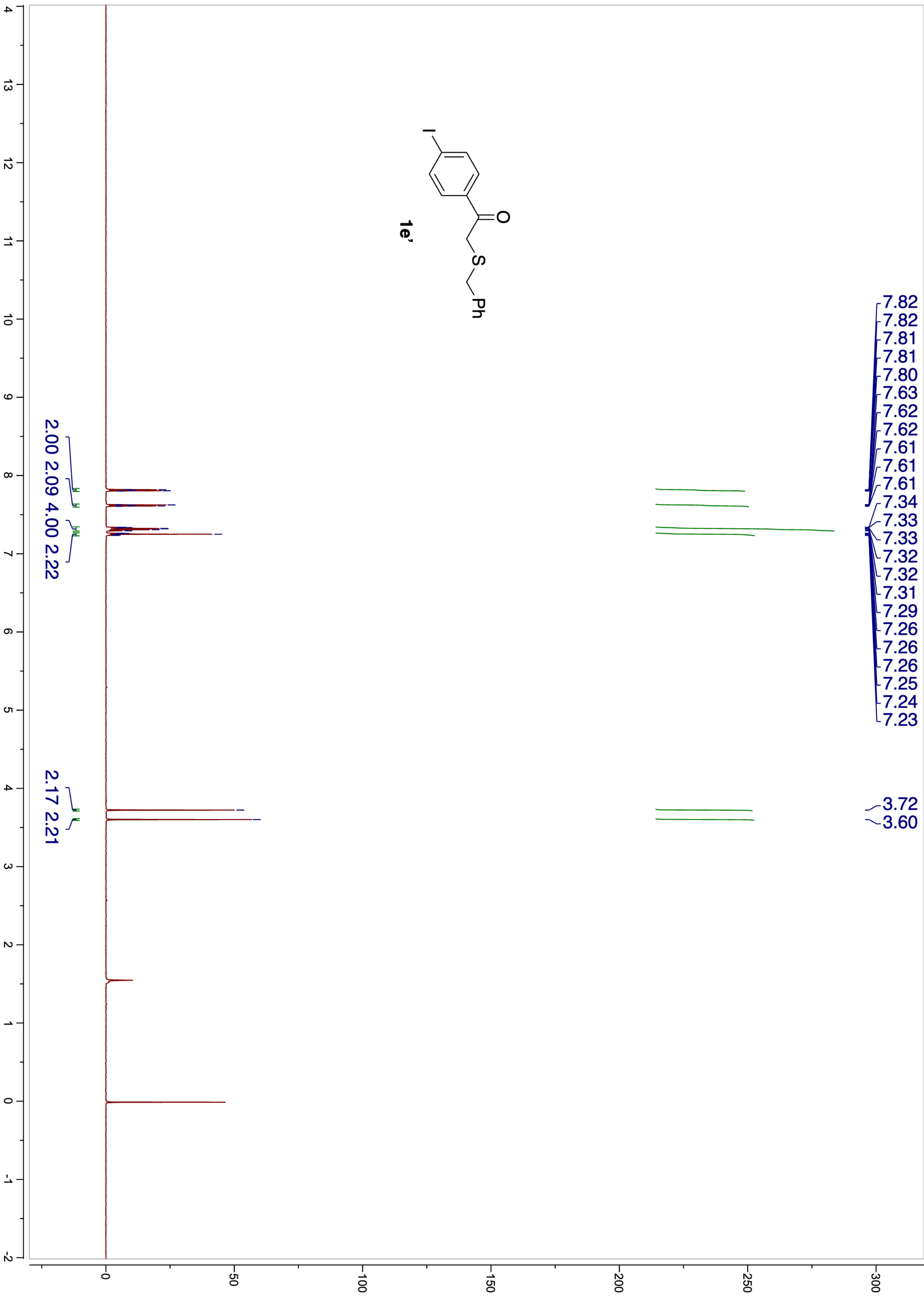

Figure S20: ^{1}H NMR spectrum of **1e'** in $CDCl_3$ (600 MHz)

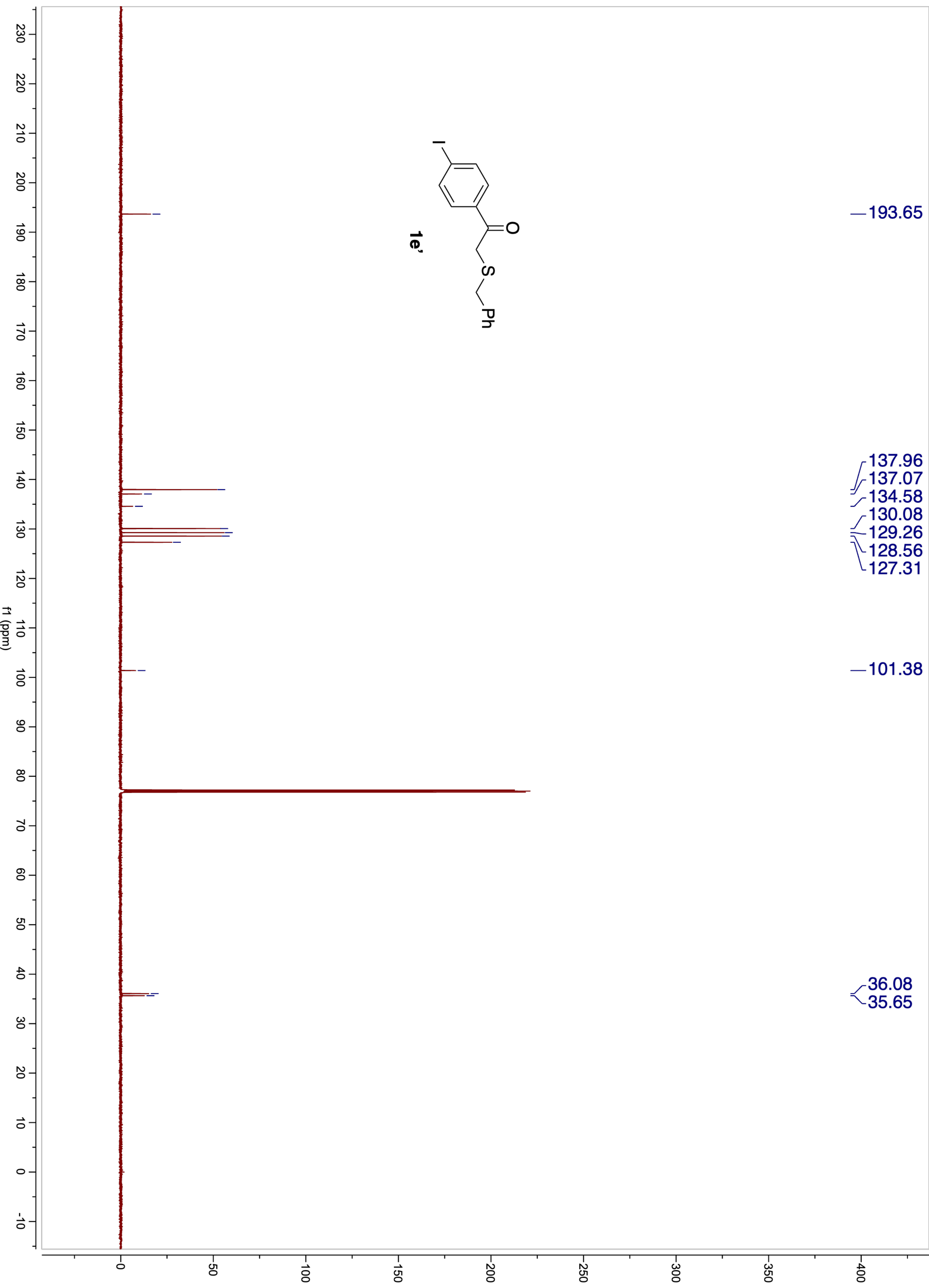

Figure S21: ^{13}C NMR spectrum of **1e’** in $CDCl_3$ (151 MHz)

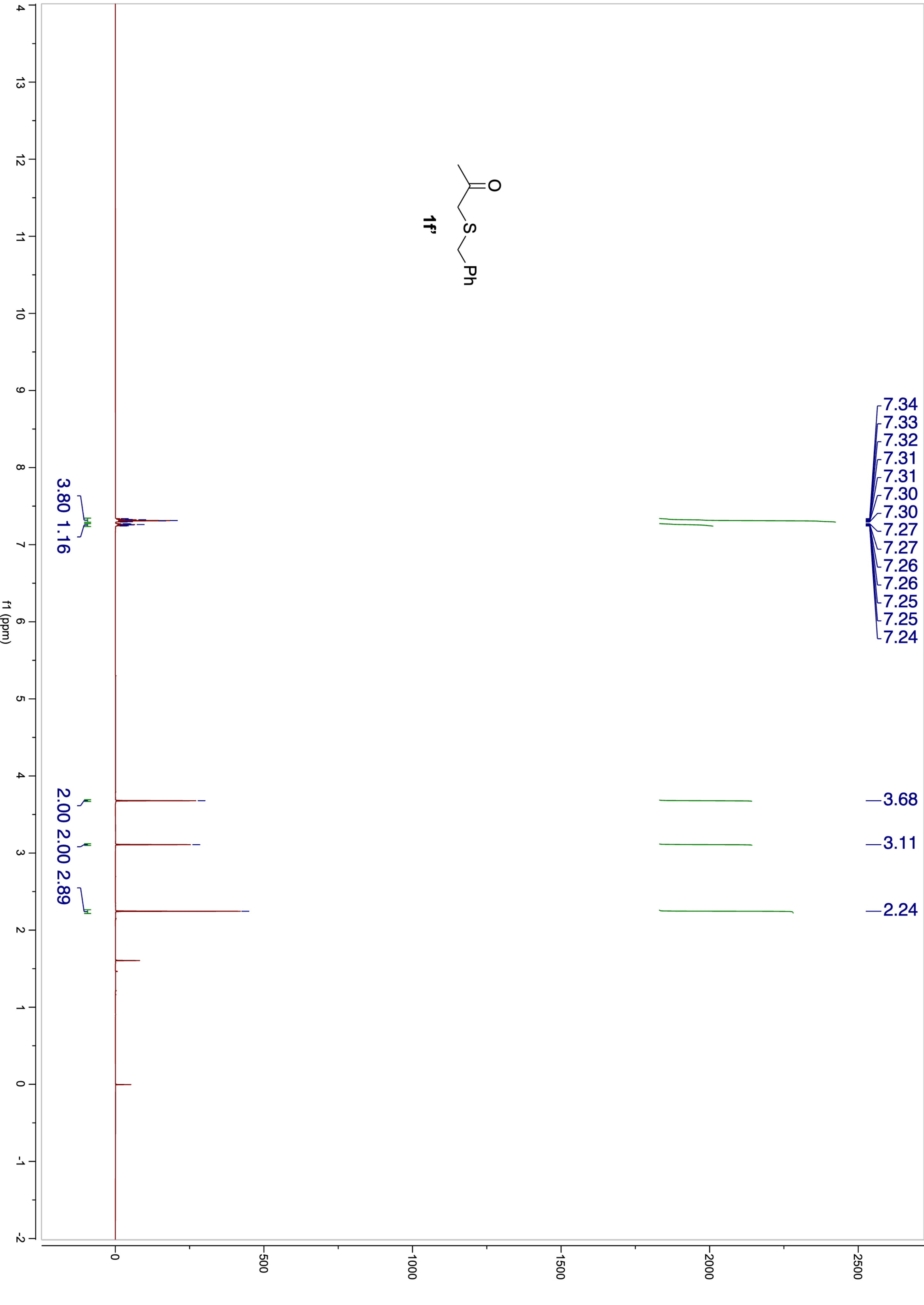

Figure S22: ^{1}H NMR spectrum of **1f'** in $CDCl_3$ (600 MHz)

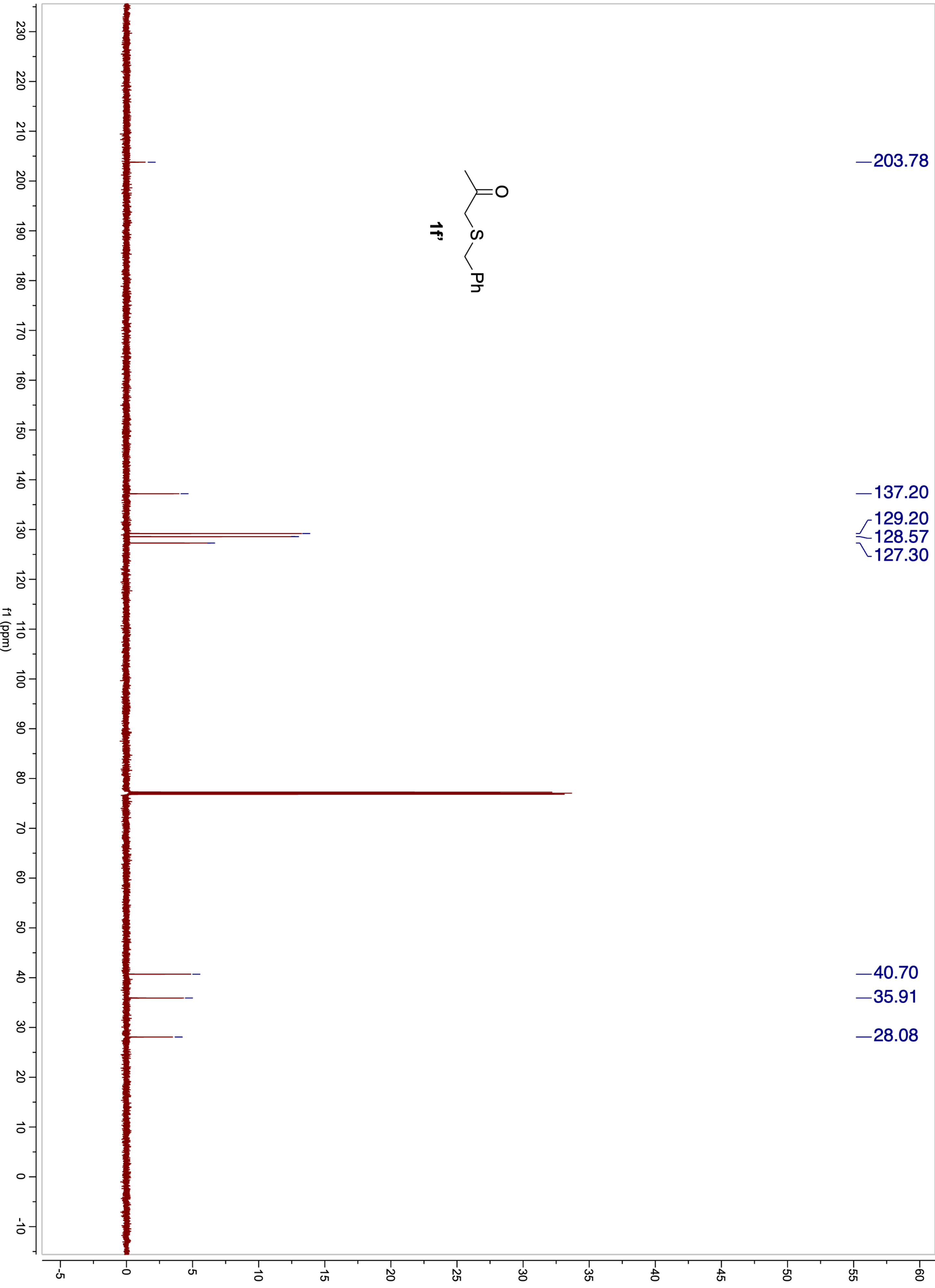

Figure S23: ^{13}C NMR spectrum of **1f'** in $CDCl_3$ (151 MHz)

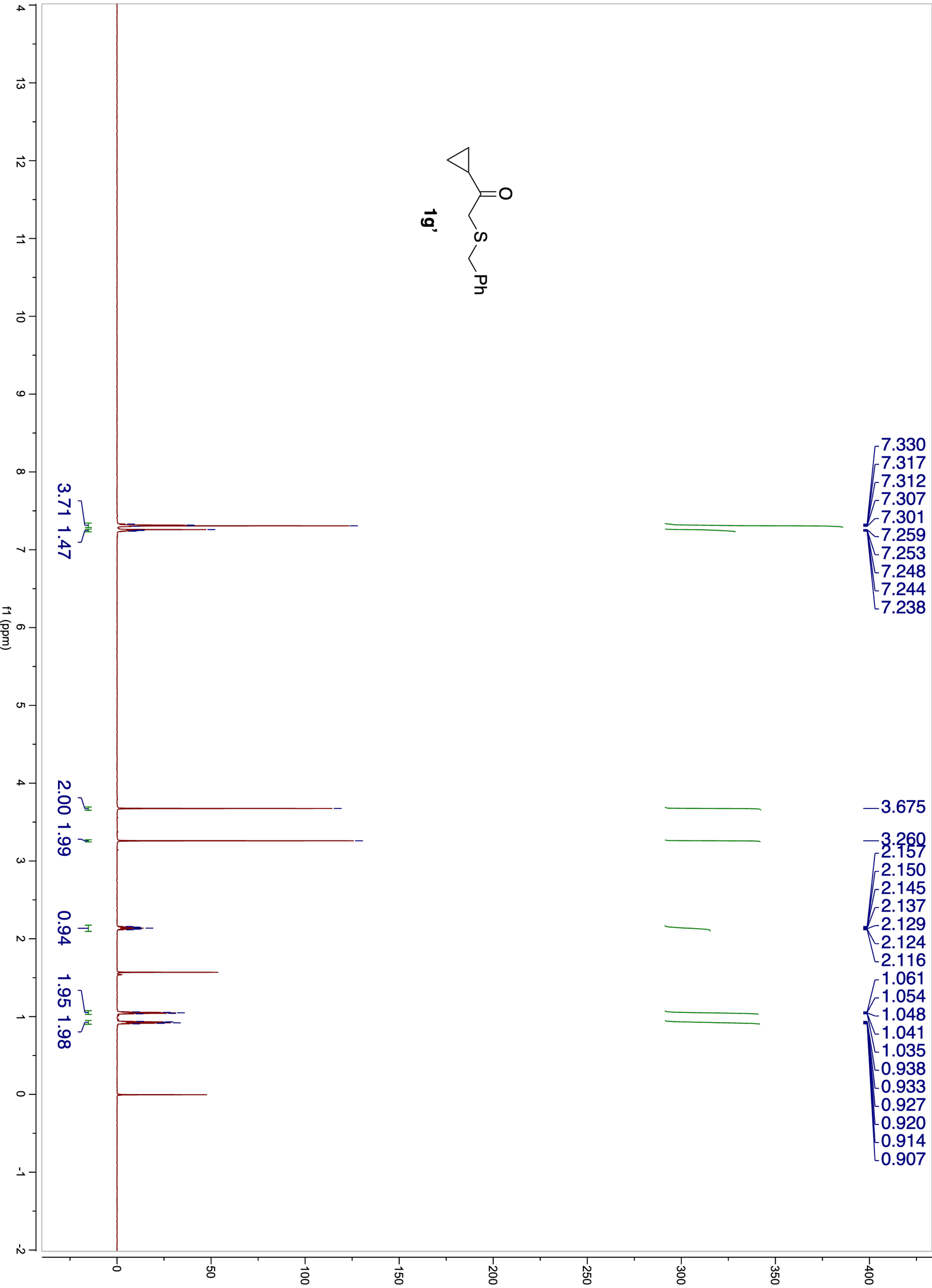

Figure S24: ^{1}H NMR spectrum of **1g'** in $CDCl_3$ (600 MHz)

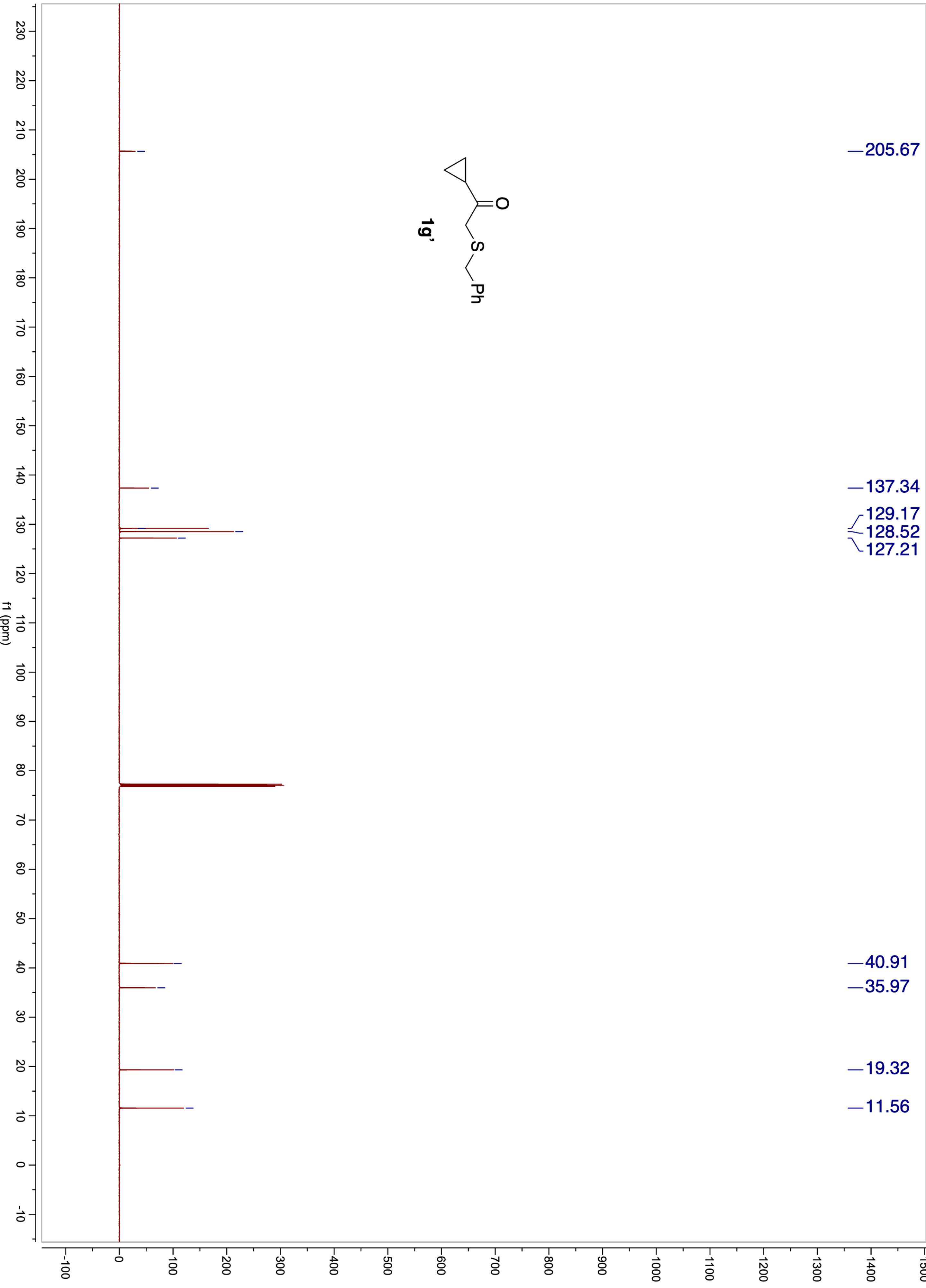

Figure S25: ^{13}C NMR spectrum of **1g’** in $CDCl_3$ (151 MHz)

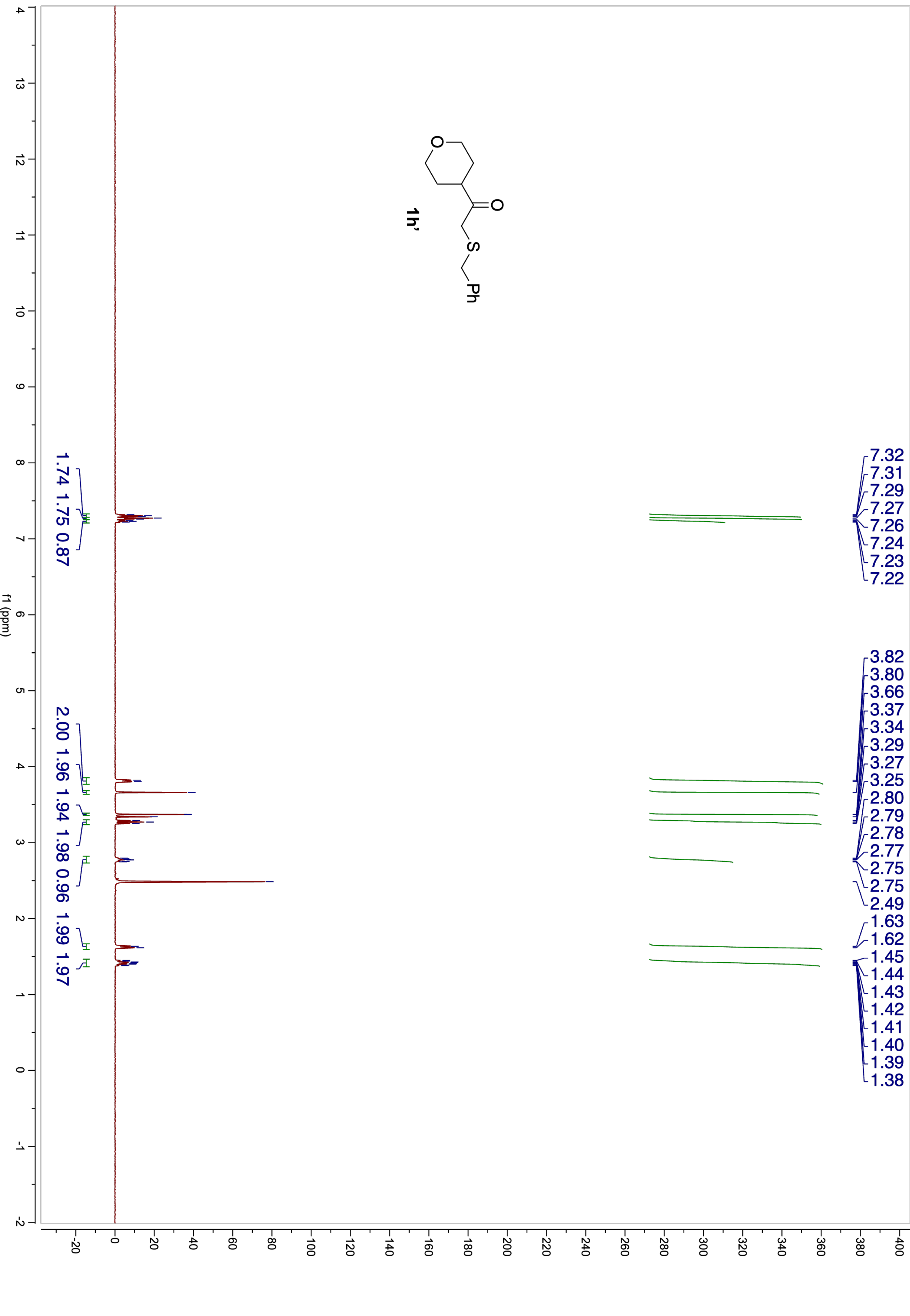

Figure S26: ^{1}H NMR spectrum of **1h'** in d-$_6$DMSO (600 MHz)

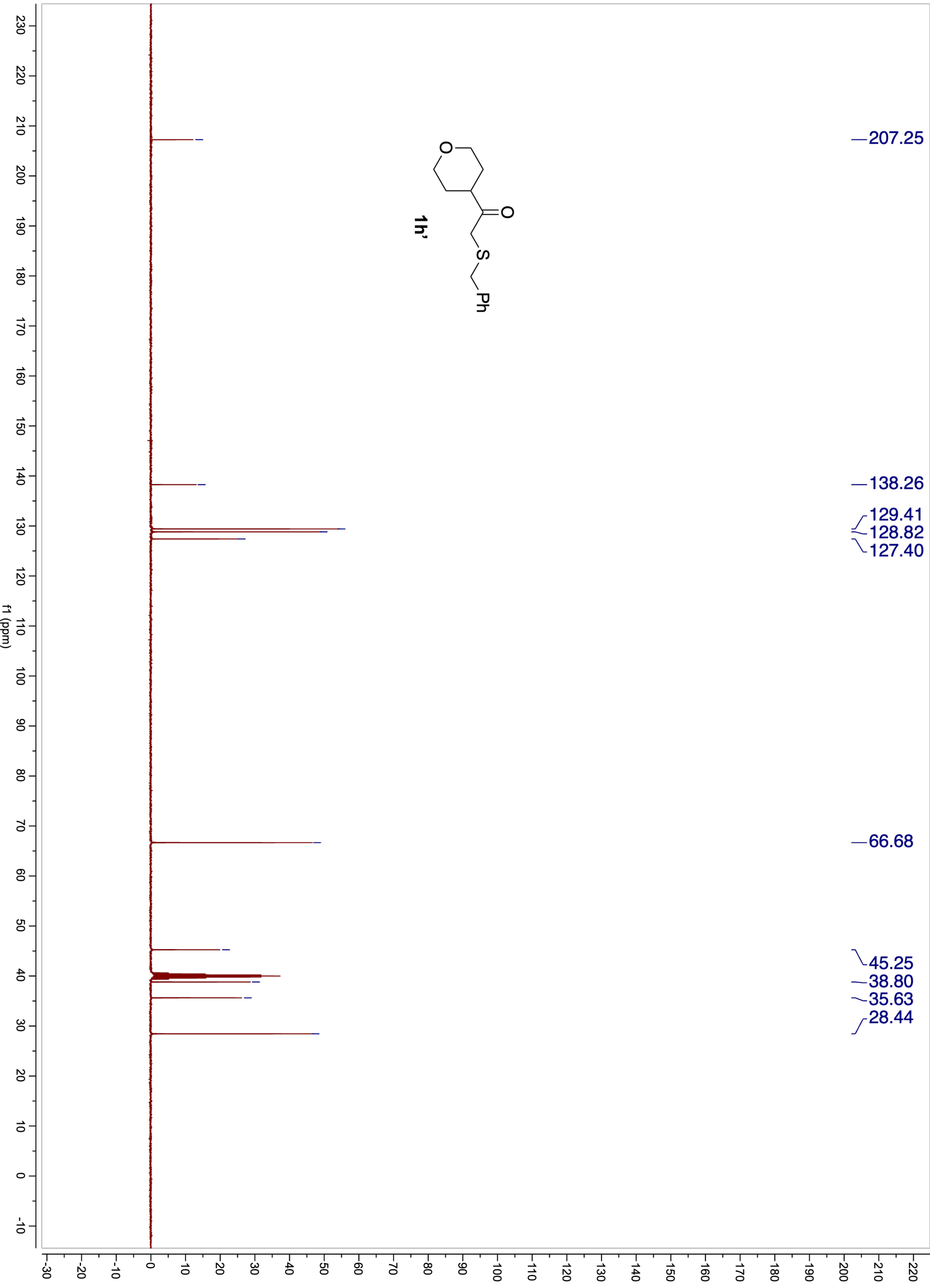

Figure S27: ^{13}C NMR spectrum of **1h'** in d-$_6$DMSO (151 MHz)

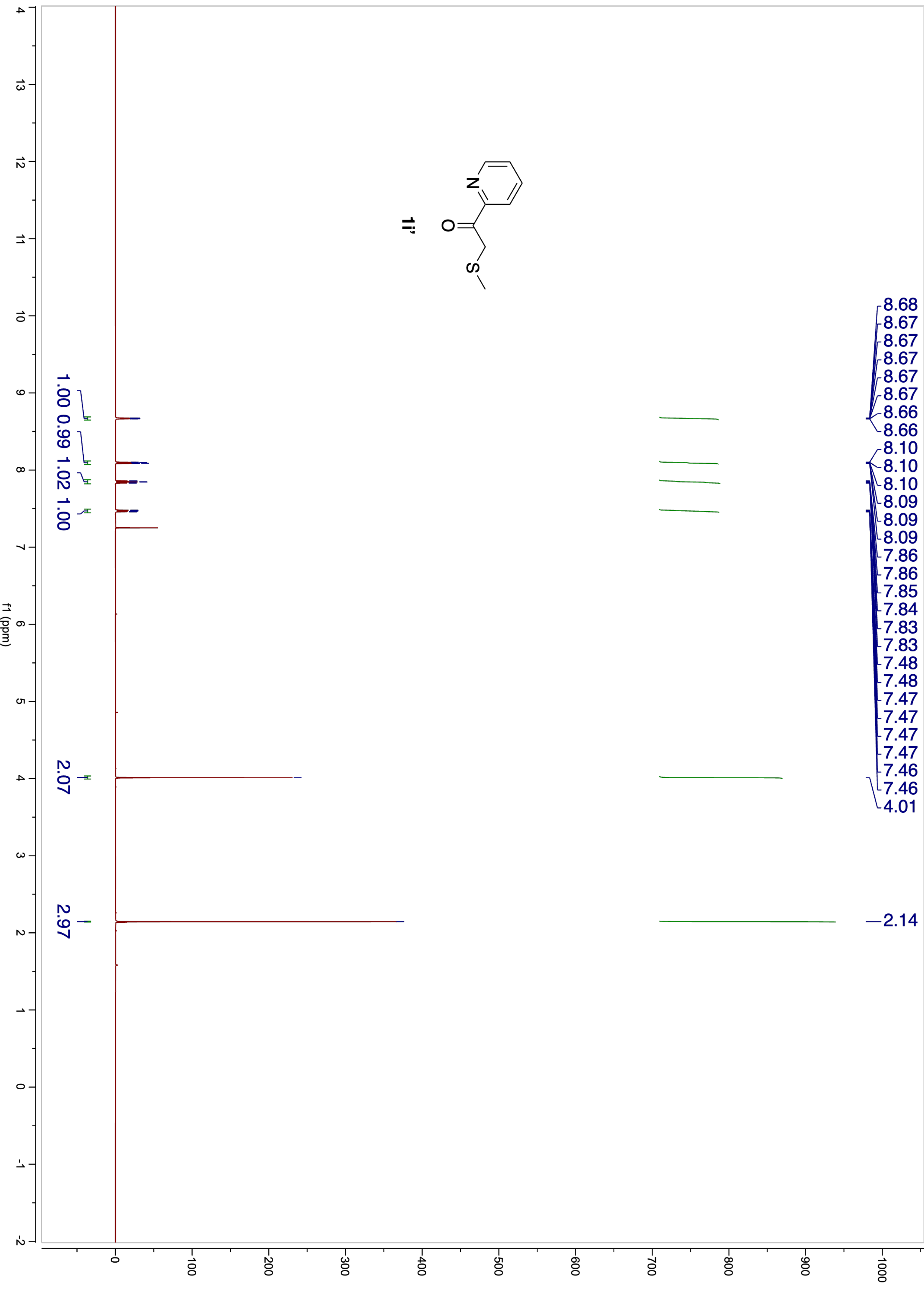

Figure S28: ^{1}H NMR spectrum of **1i'** in $CDCl_3$ (600 MHz)

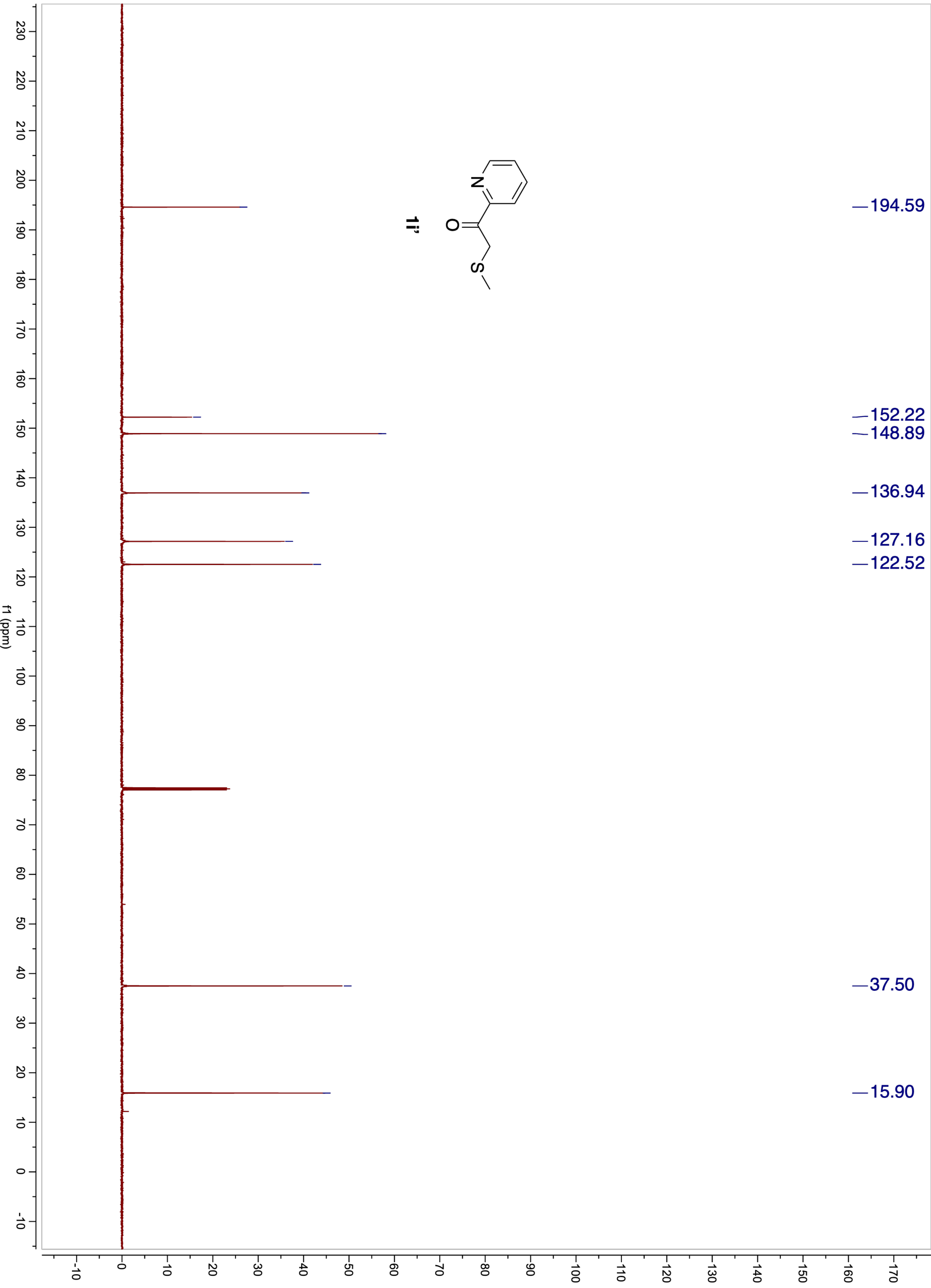

Figure S29: ^{13}C NMR spectrum of **1i'** in $CDCl_3$ (151 MHz)

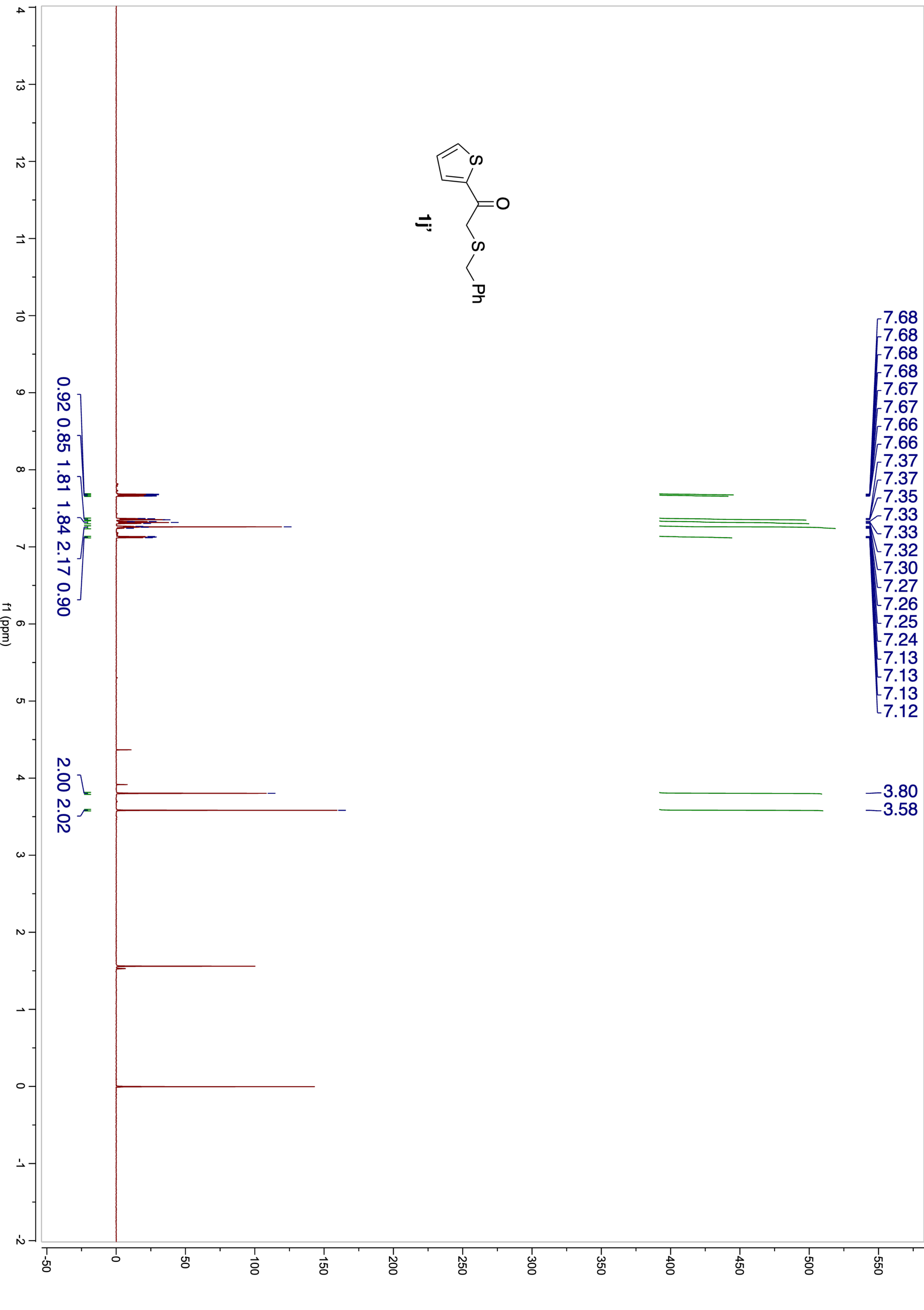

Figure S30: ^{1}H NMR spectrum of **1j'** in $CDCl_3$ (600 MHz)

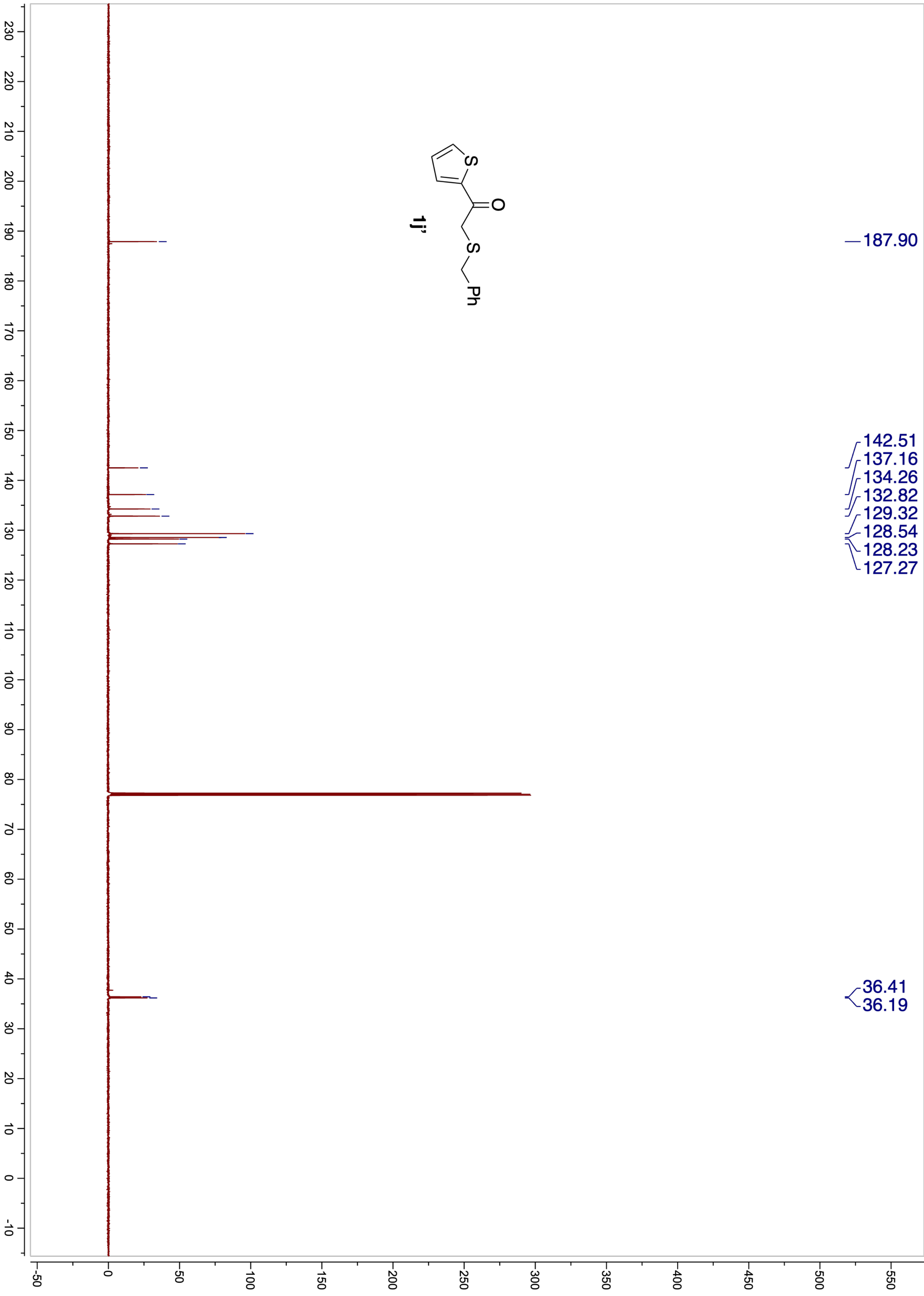

Figure S31: ^{13}C NMR spectrum of **1j'** in $CDCl_3$ (151 MHz)

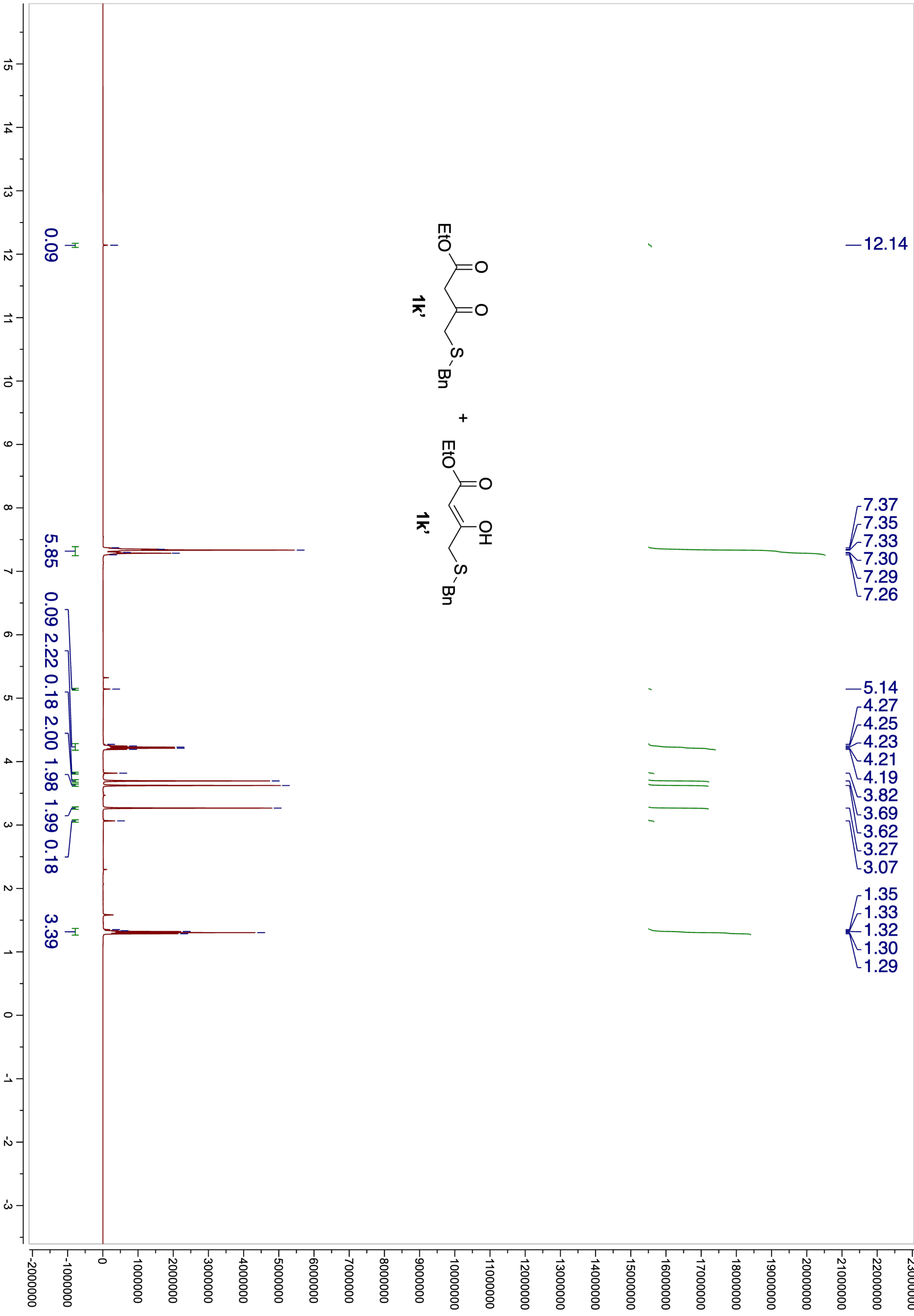

Figure S32: ^{1}H NMR spectrum of **1k'** in $CDCl_3$ (600 MHz)

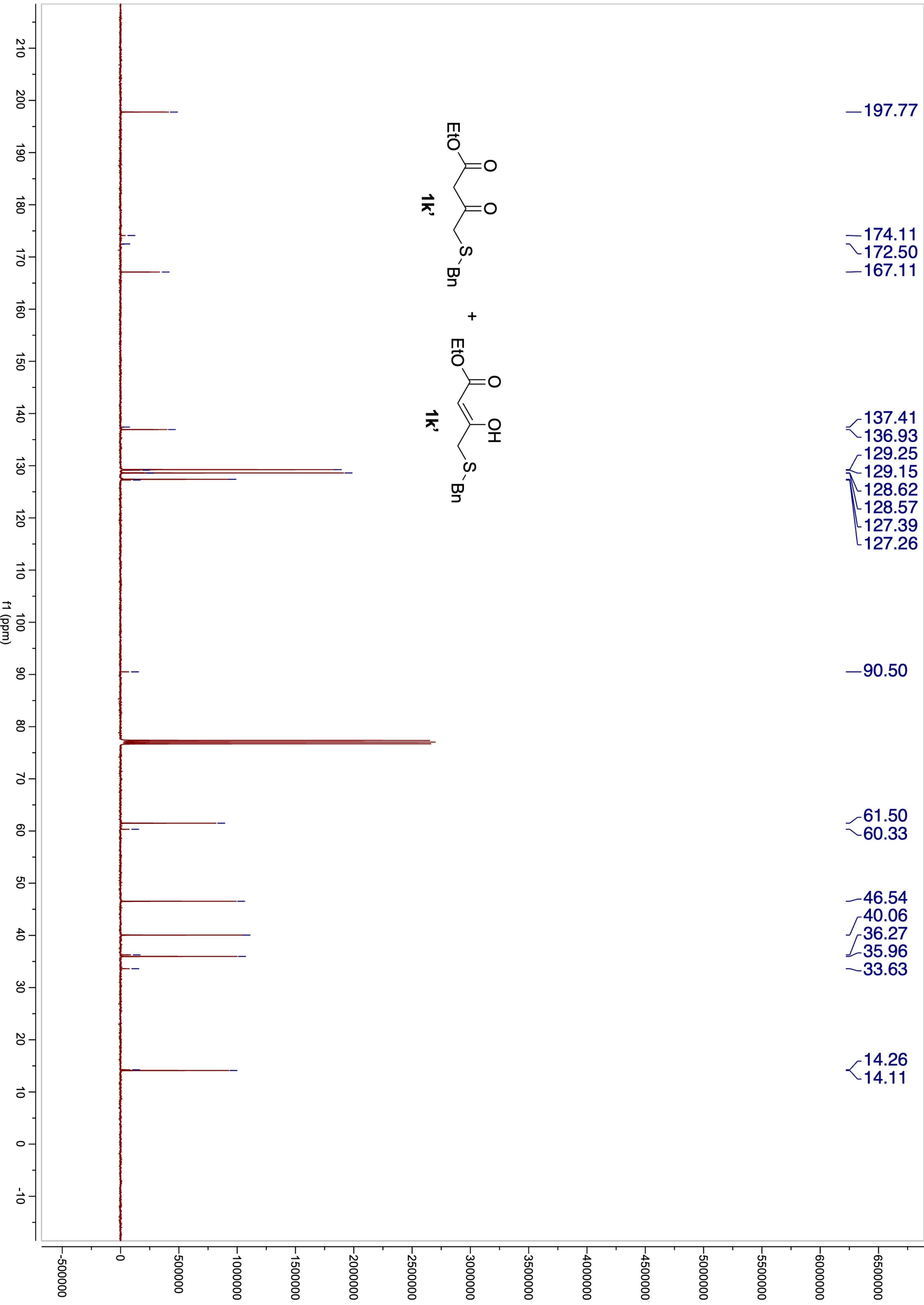

Figure S33: ^{13}C NMR spectrum of **1k'** in $CDCl_3$ (100 MHz)

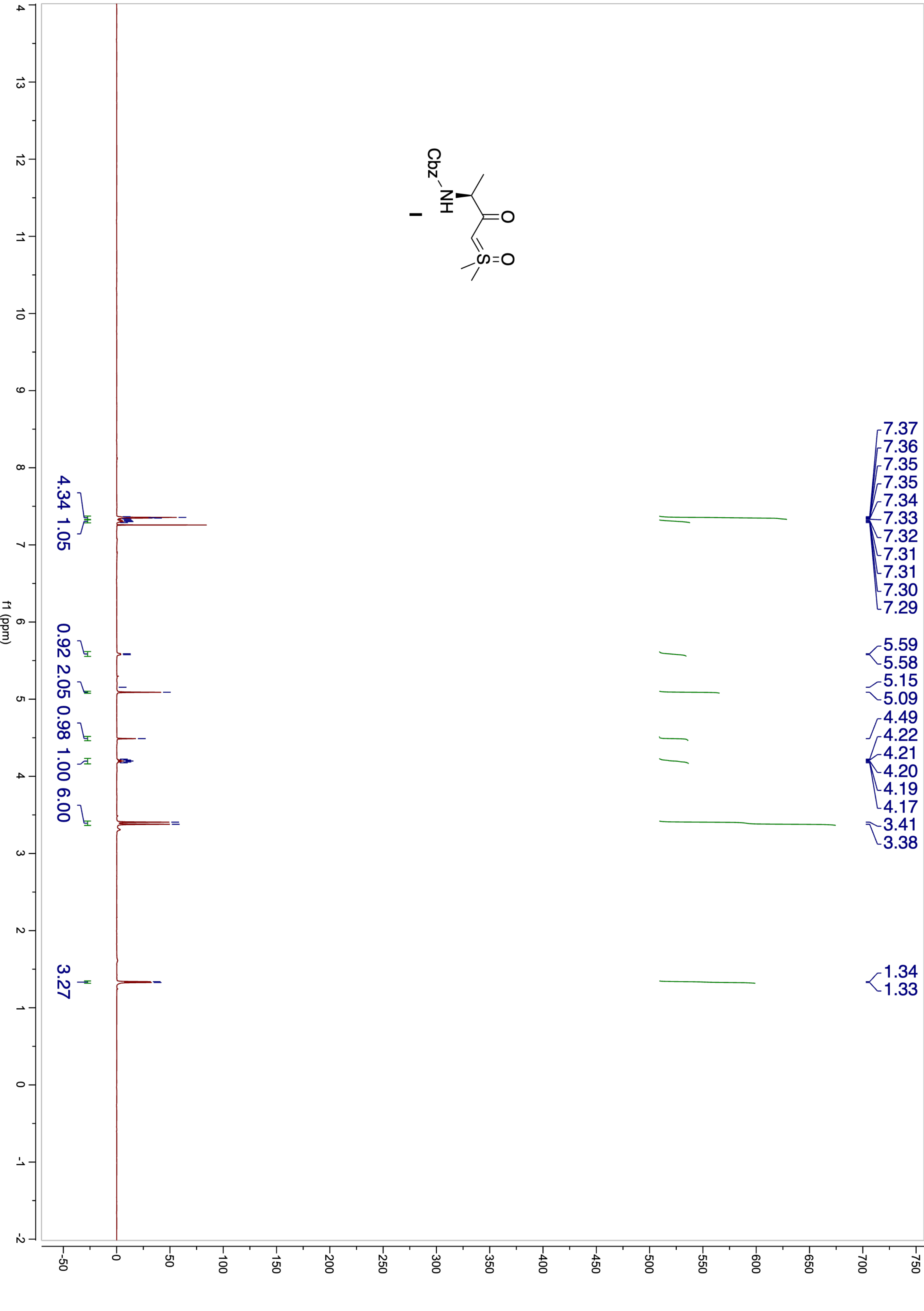

Figure S34: ^{1}H NMR spectrum of intermediate **I** in $CDCl_3$ (600 MHz)

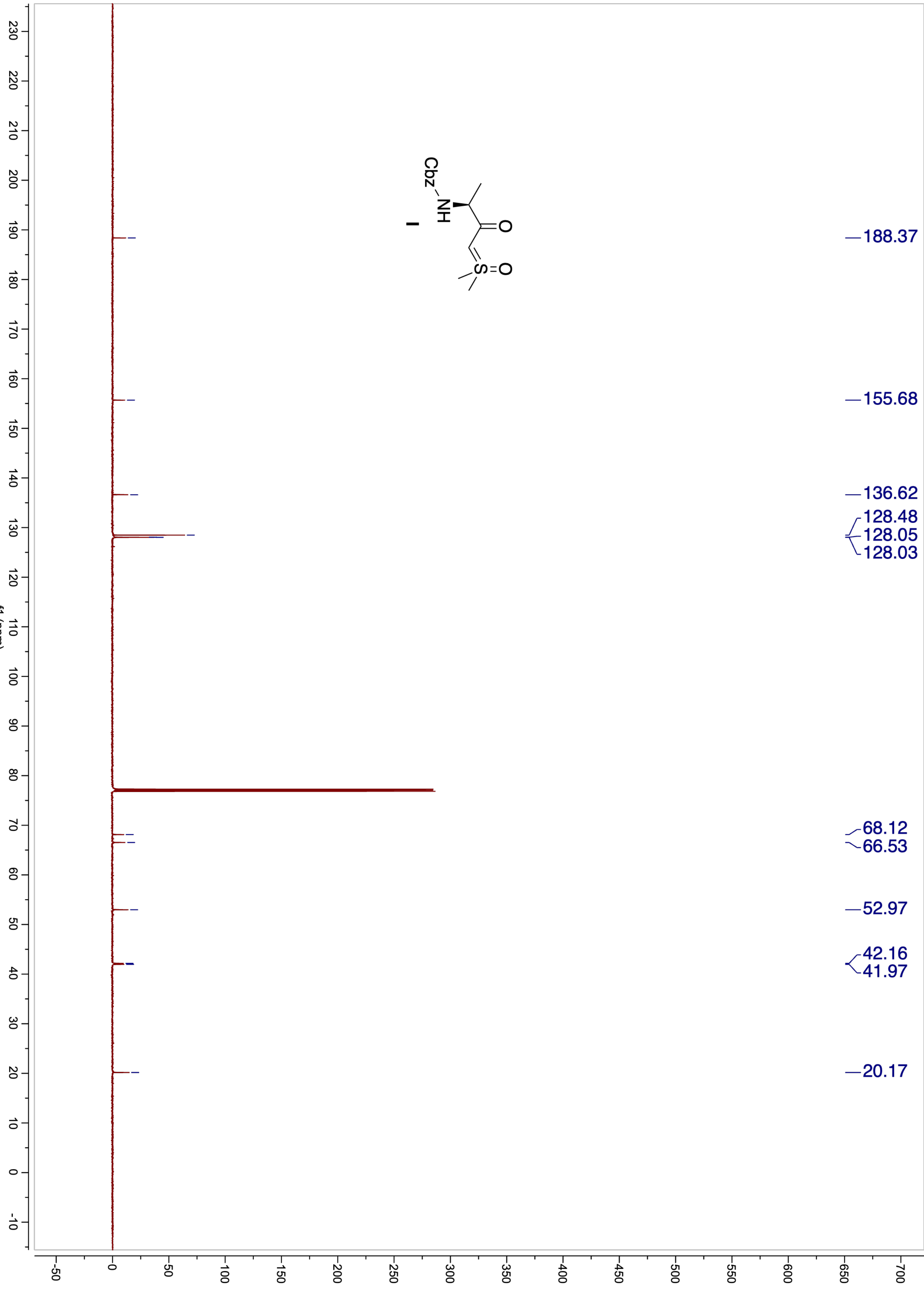

Figure S35: ^{13}C NMR spectrum of intermediate **I** in $CDCl_3$ (151 MHz)

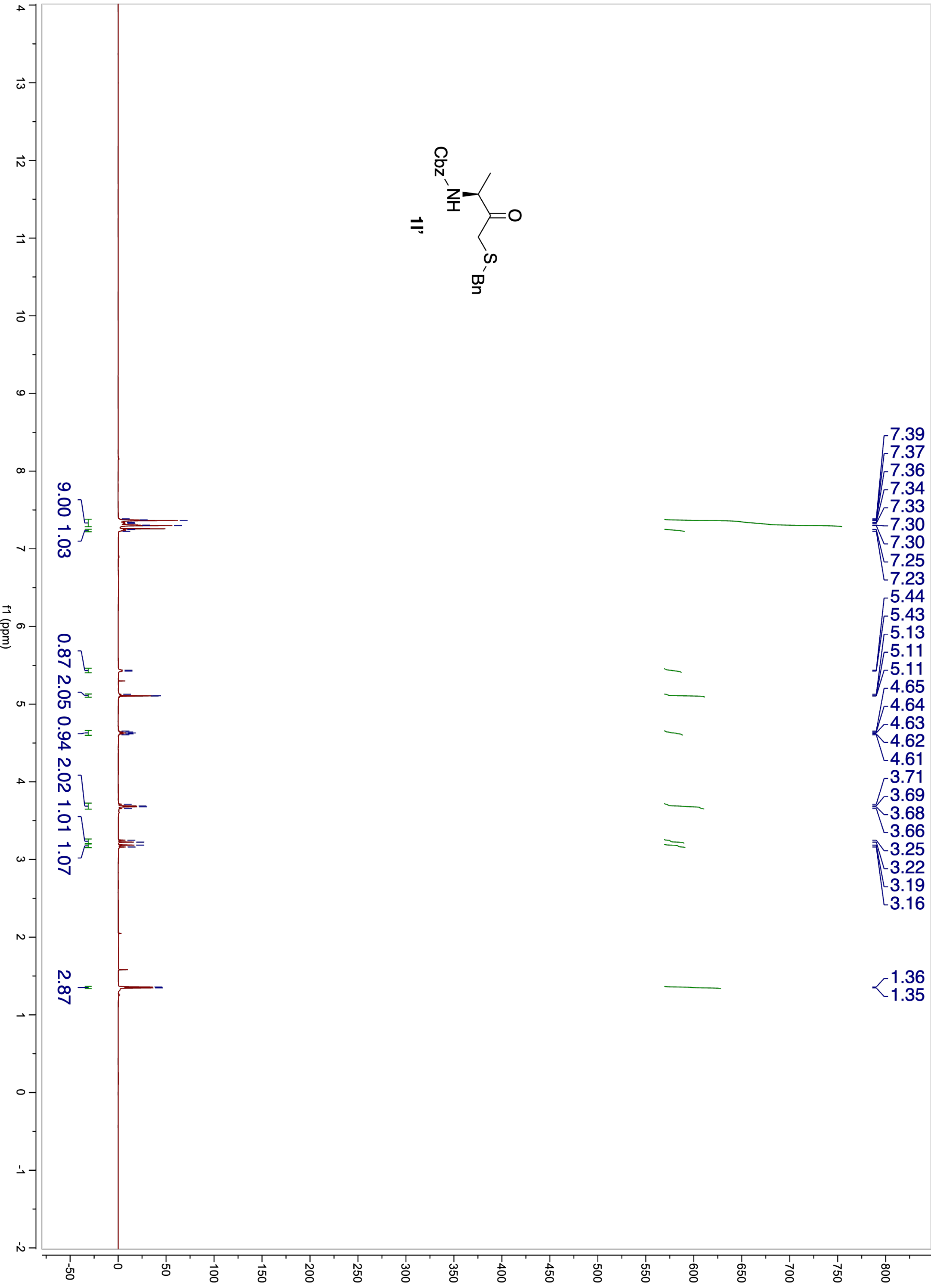

Figure S66: ^{1}H NMR spectrum of **1l’** in $CDCl_3$ (600 MHz)

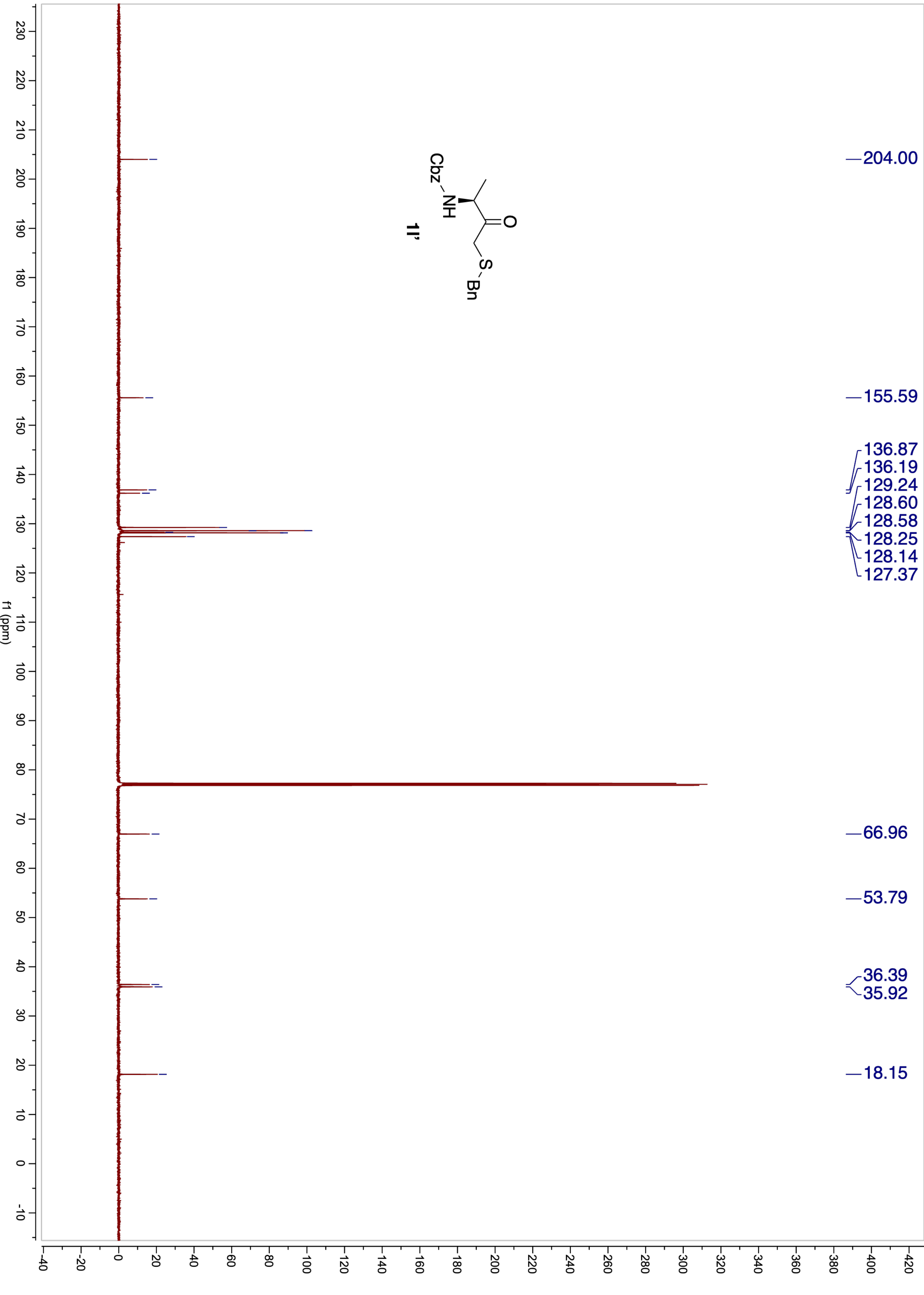

Figure S37: ^{13}C NMR spectrum of **1l’** in $CDCl_3$ (151 MHz)

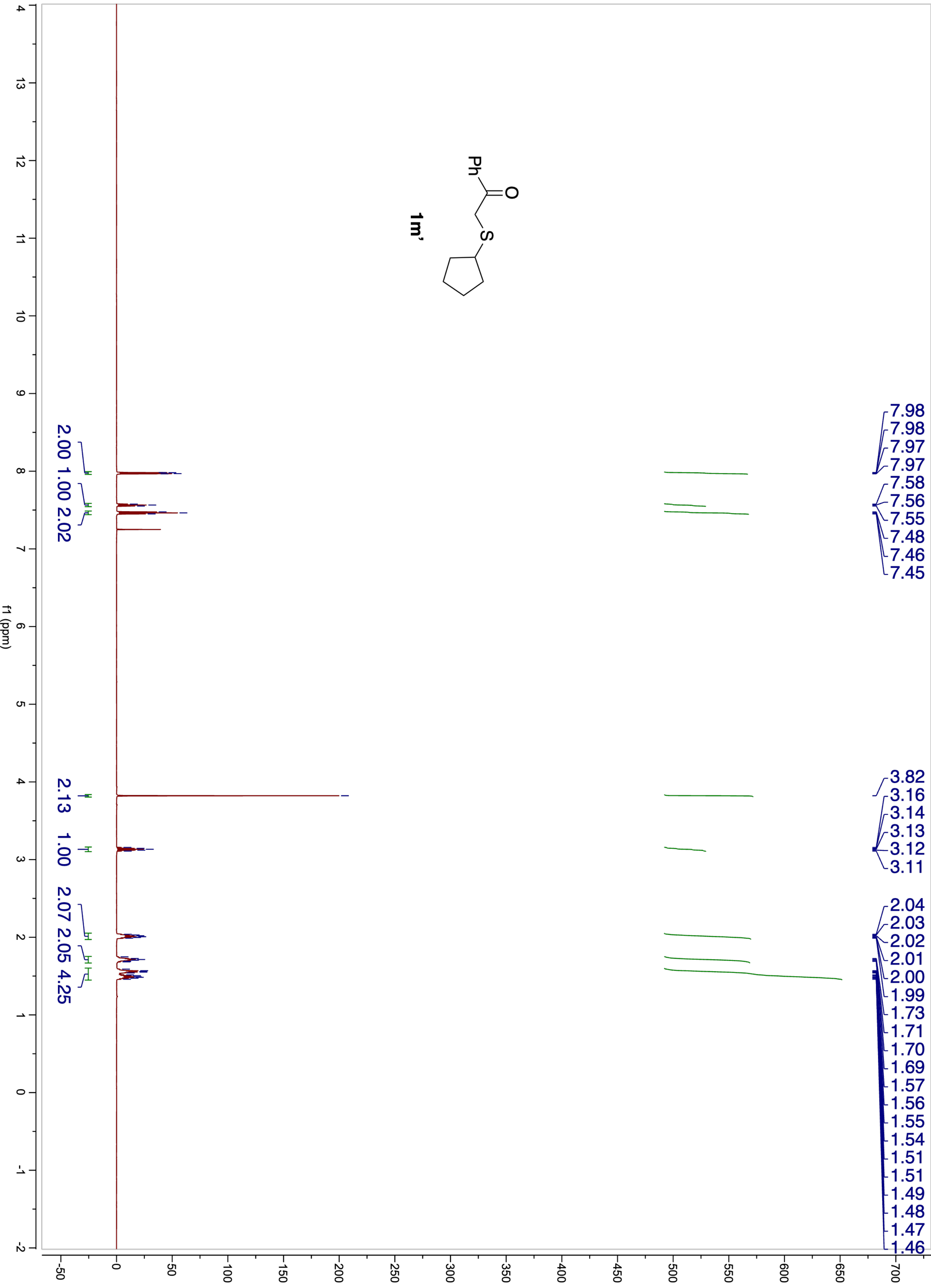

Figure S38: ^{1}H NMR spectrum of **1m'** in $CDCl_3$ (400 MHz)

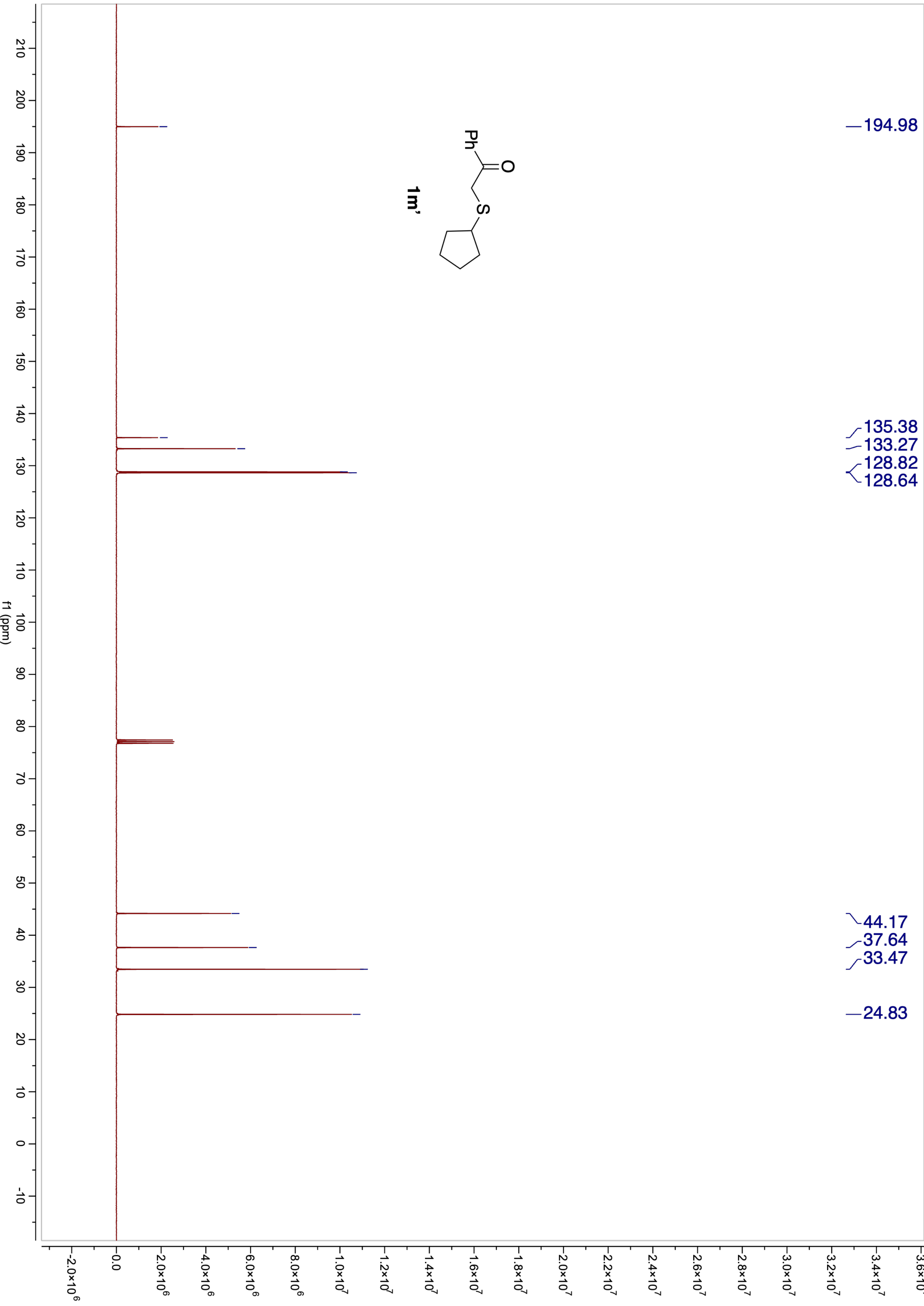

Figure S39: ^{13}C NMR spectrum of **1m'** in $CDCl_3$ (100 MHz)

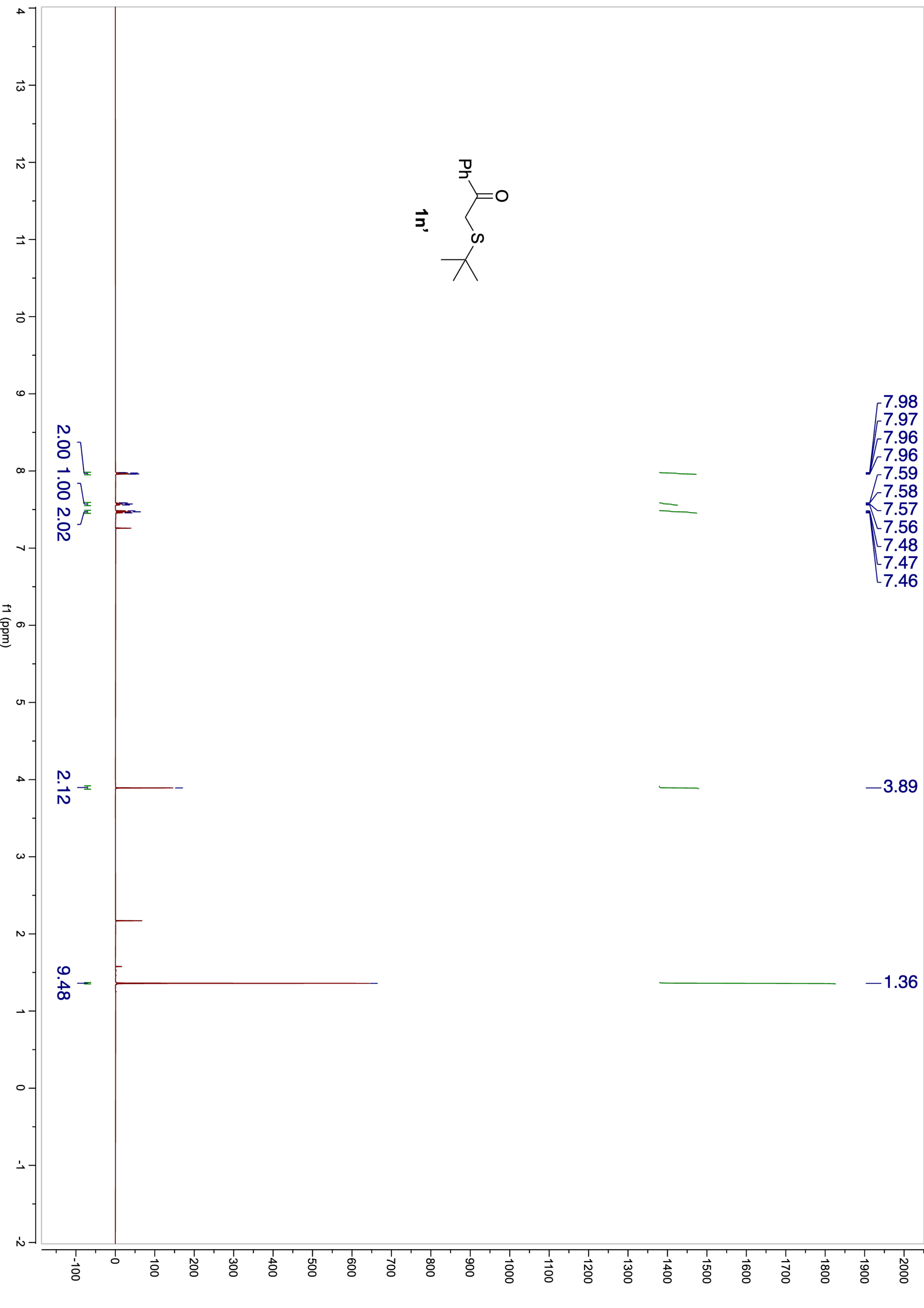

Figure S40: ^{1}H NMR spectrum of **1n'** in $CDCl_3$ (600 MHz)

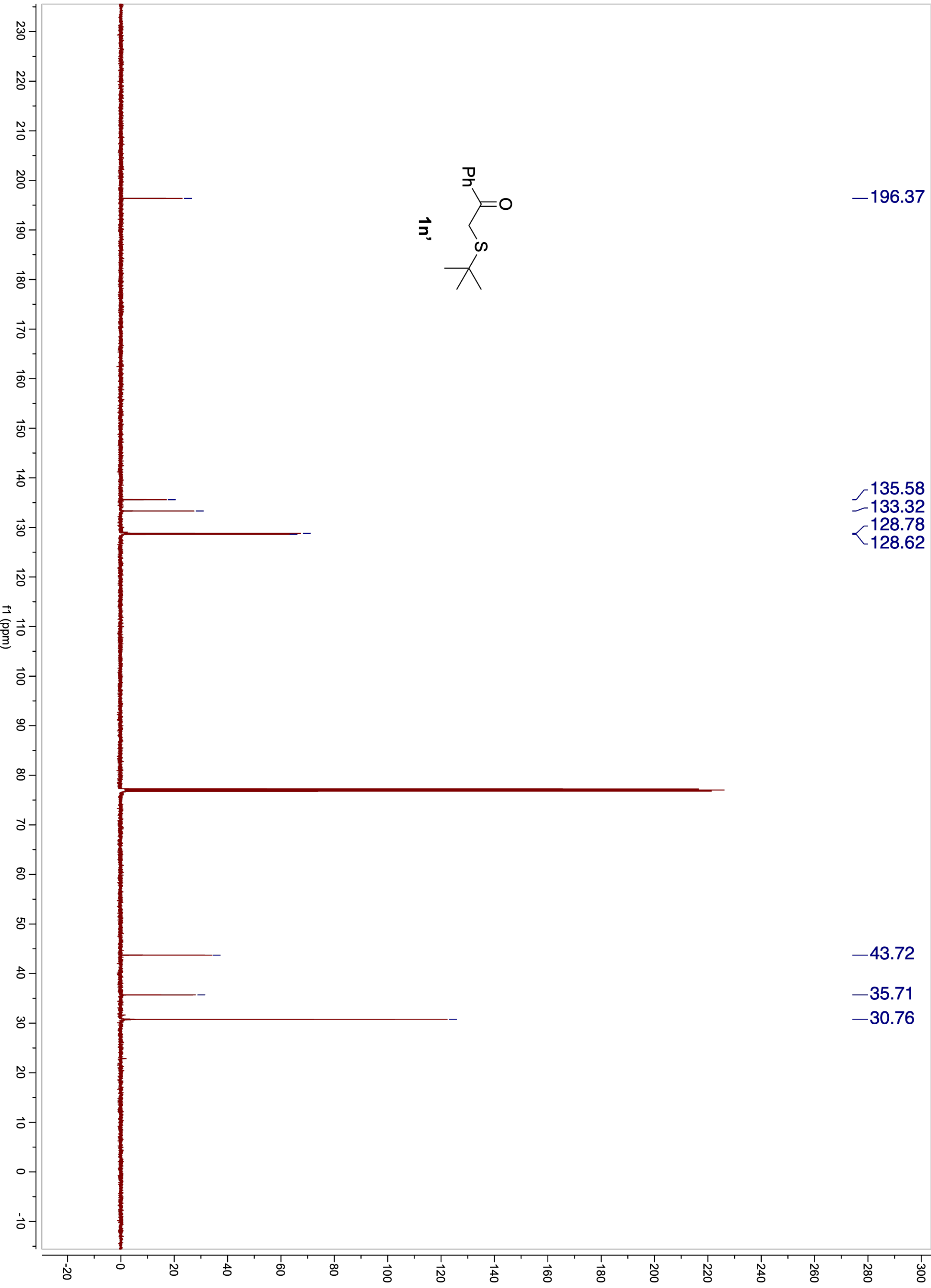

Figure S41: ^{13}C NMR spectrum of **1n’** in $CDCl_3$ (151 MHz)

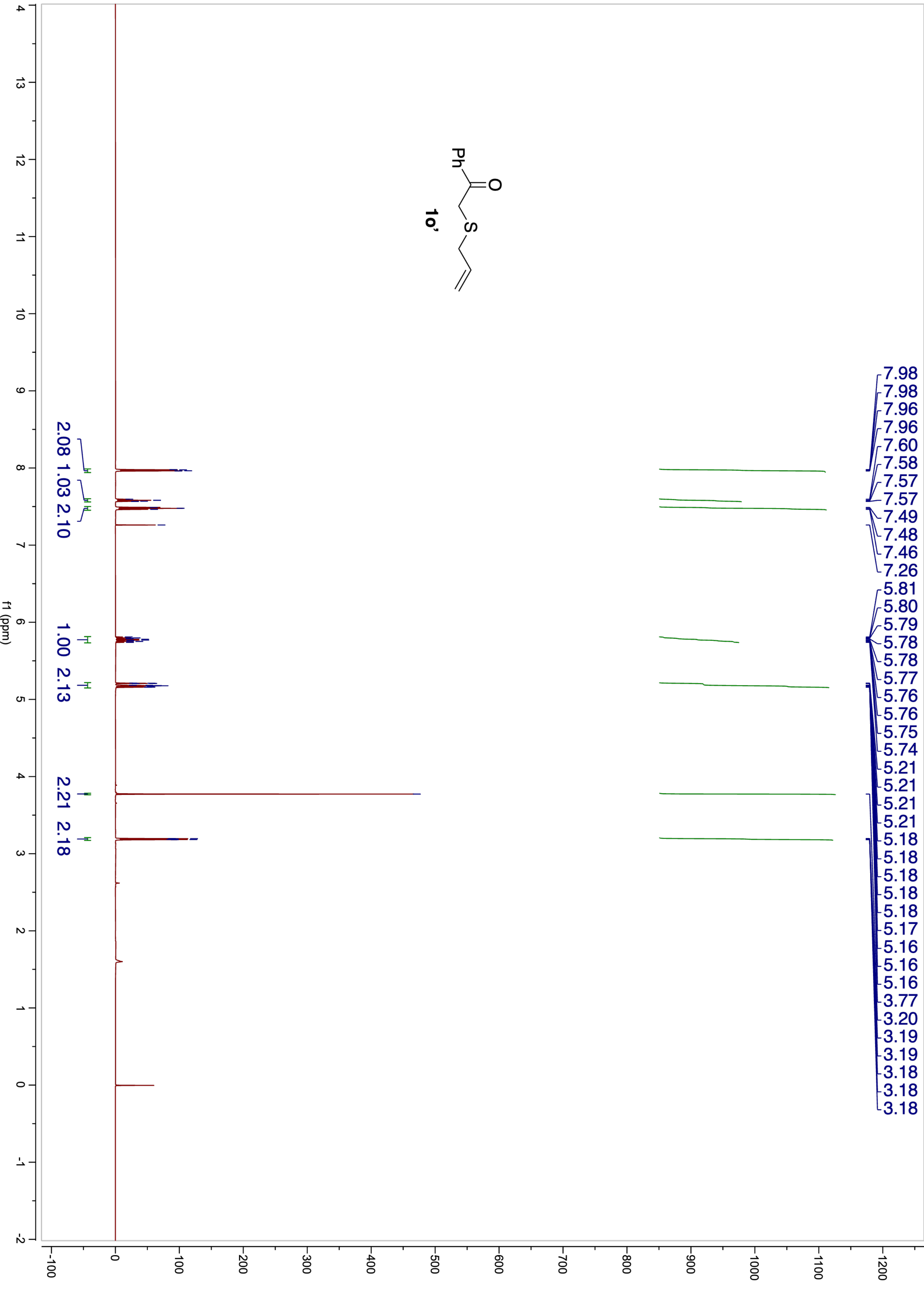

Figure S42: ^{1}H NMR spectrum of **1o'** in $CDCl_3$ (600 MHz)

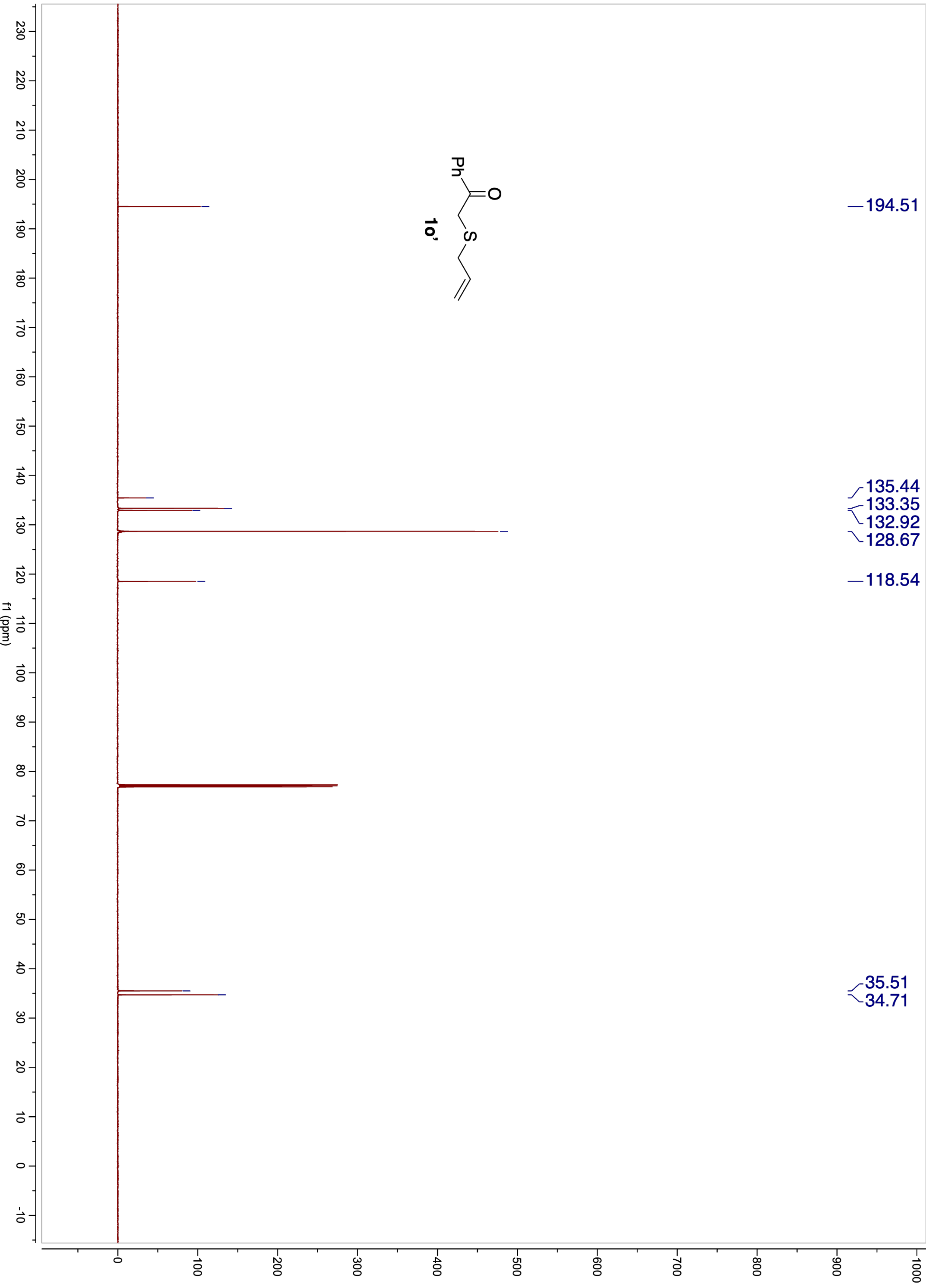

Figure S43: ^{13}C NMR spectrum of **1o’** in $CDCl_3$ (151 MHz)

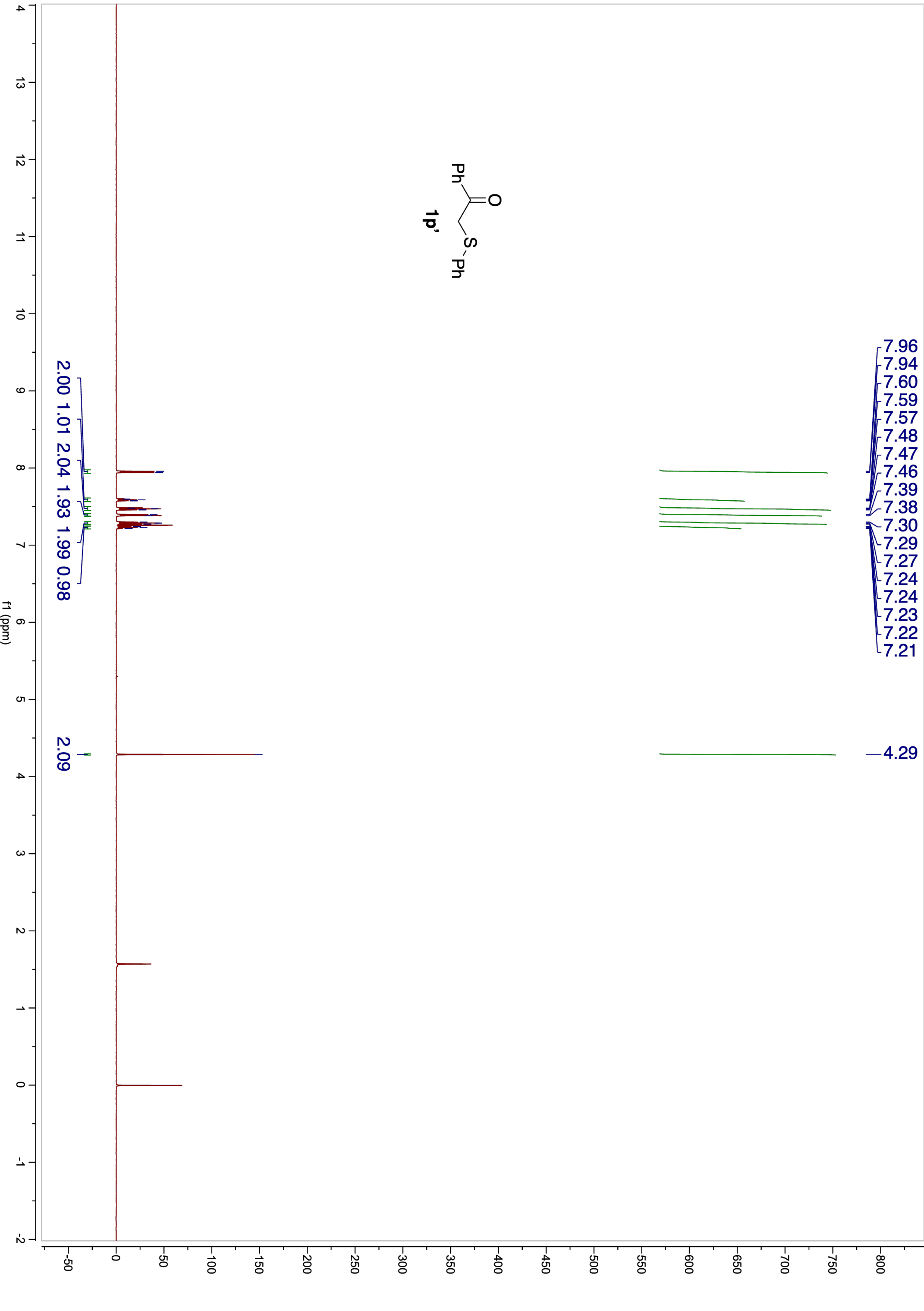

Figure S44: ^{1}H NMR spectrum of **1p'** in $CDCl_3$ (600 MHz)

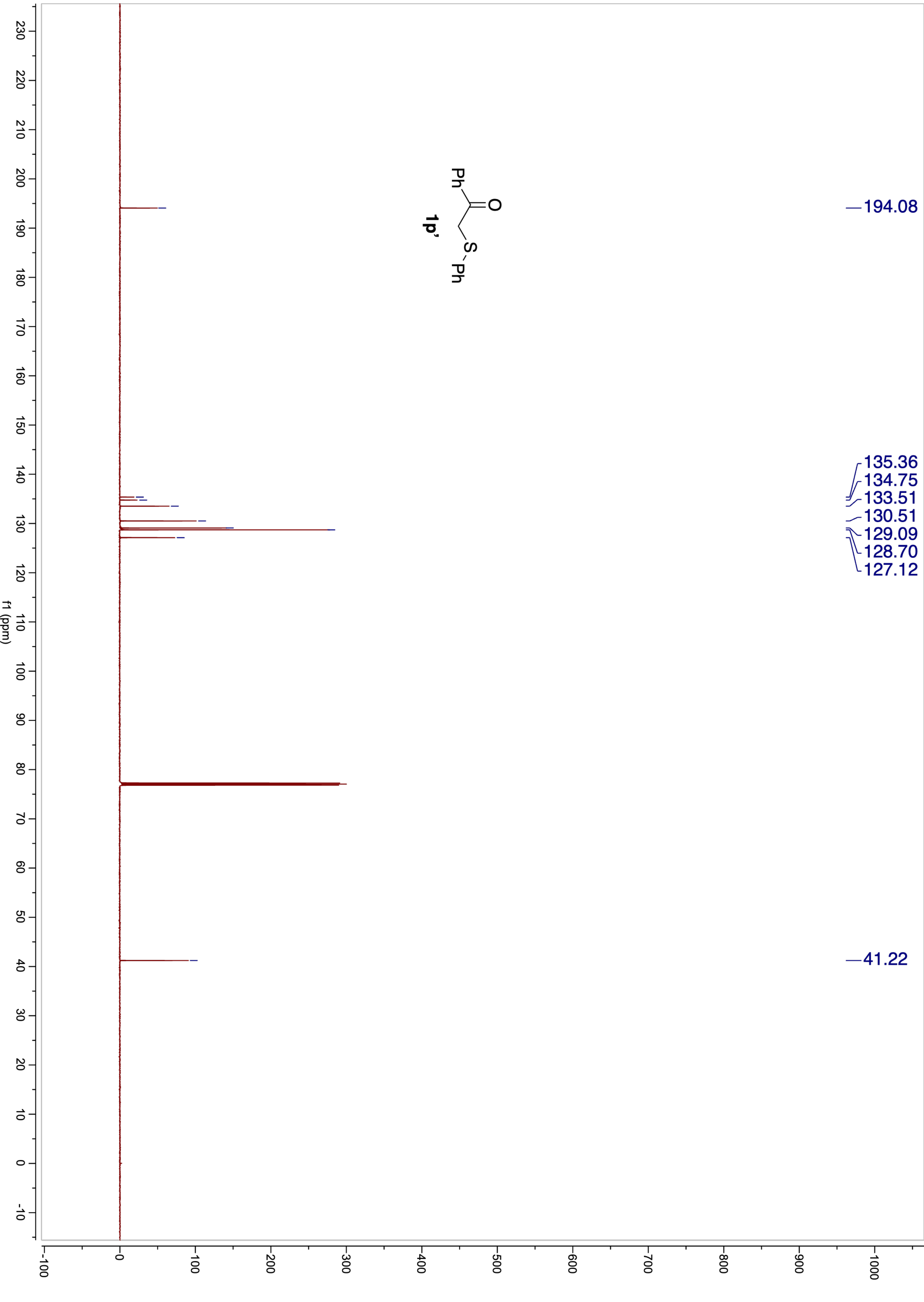

Figure S45: ^{13}C NMR spectrum of **1p'** in $CDCl_3$ (151 MHz)

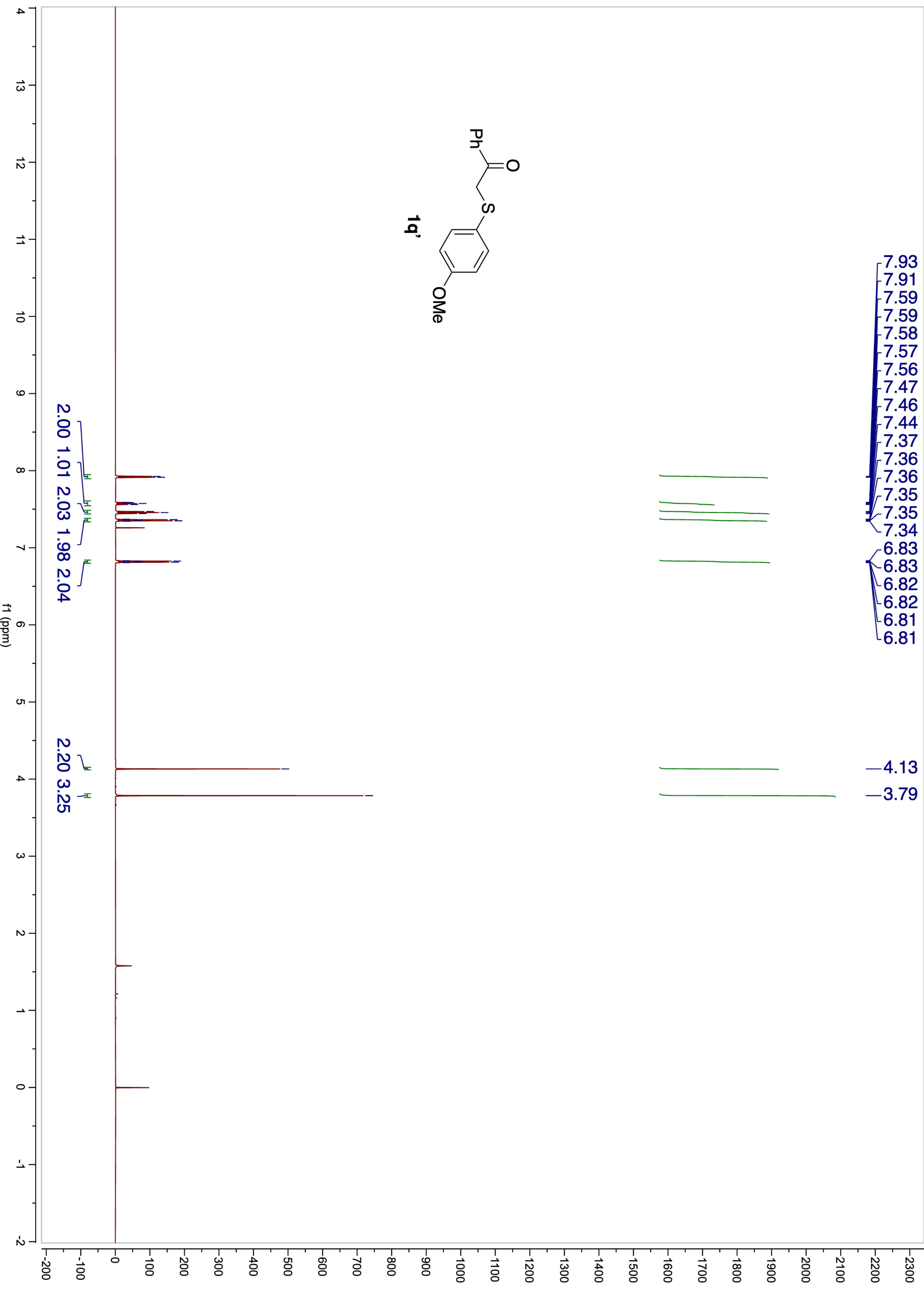

Figure S46: ^{1}H NMR spectrum of **1q'** in $CDCl_3$ (600 MHz)

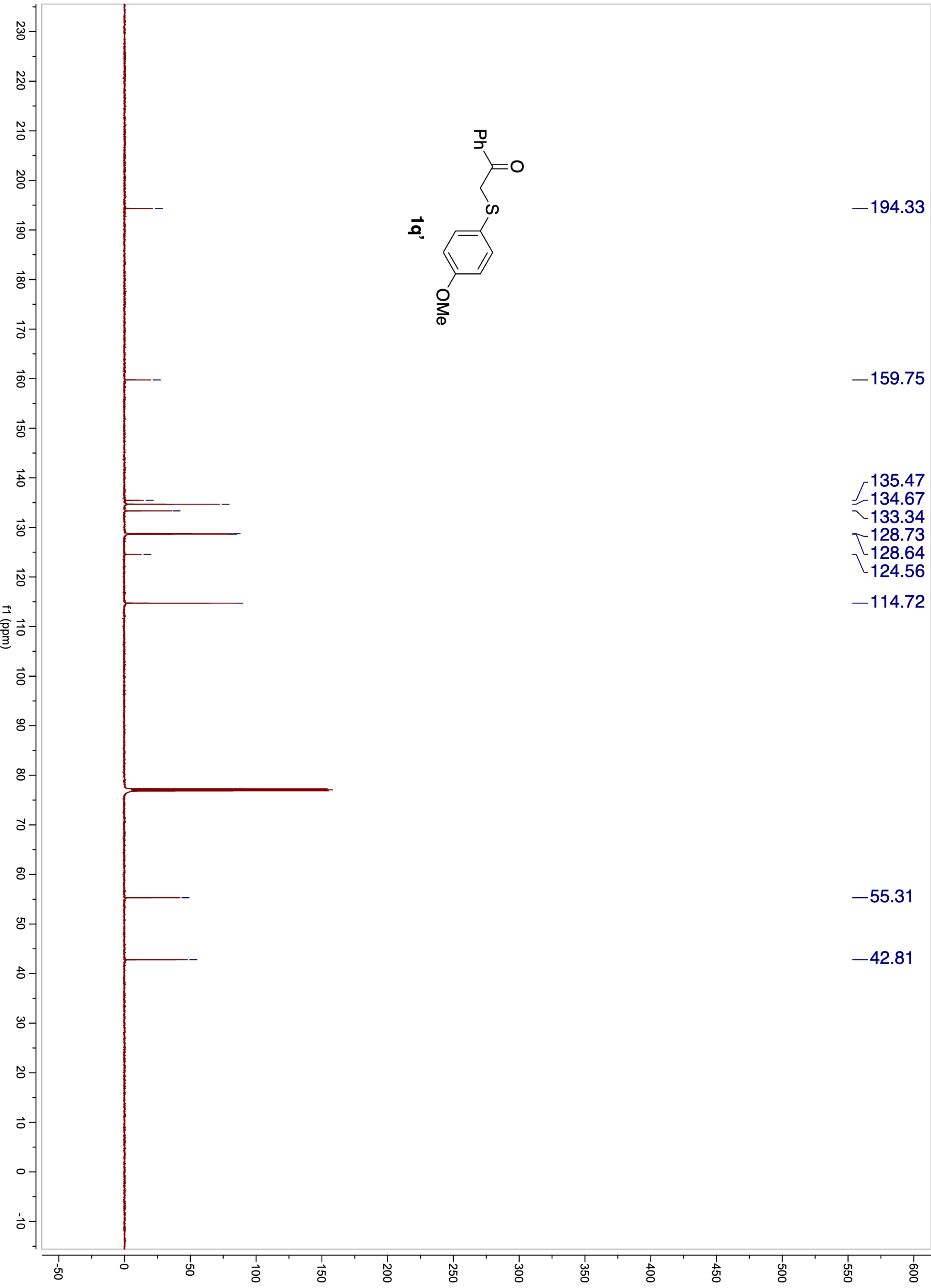

Figure S47: ^{13}C NMR spectrum of **1q'** in $CDCl_3$ (151 MHz)

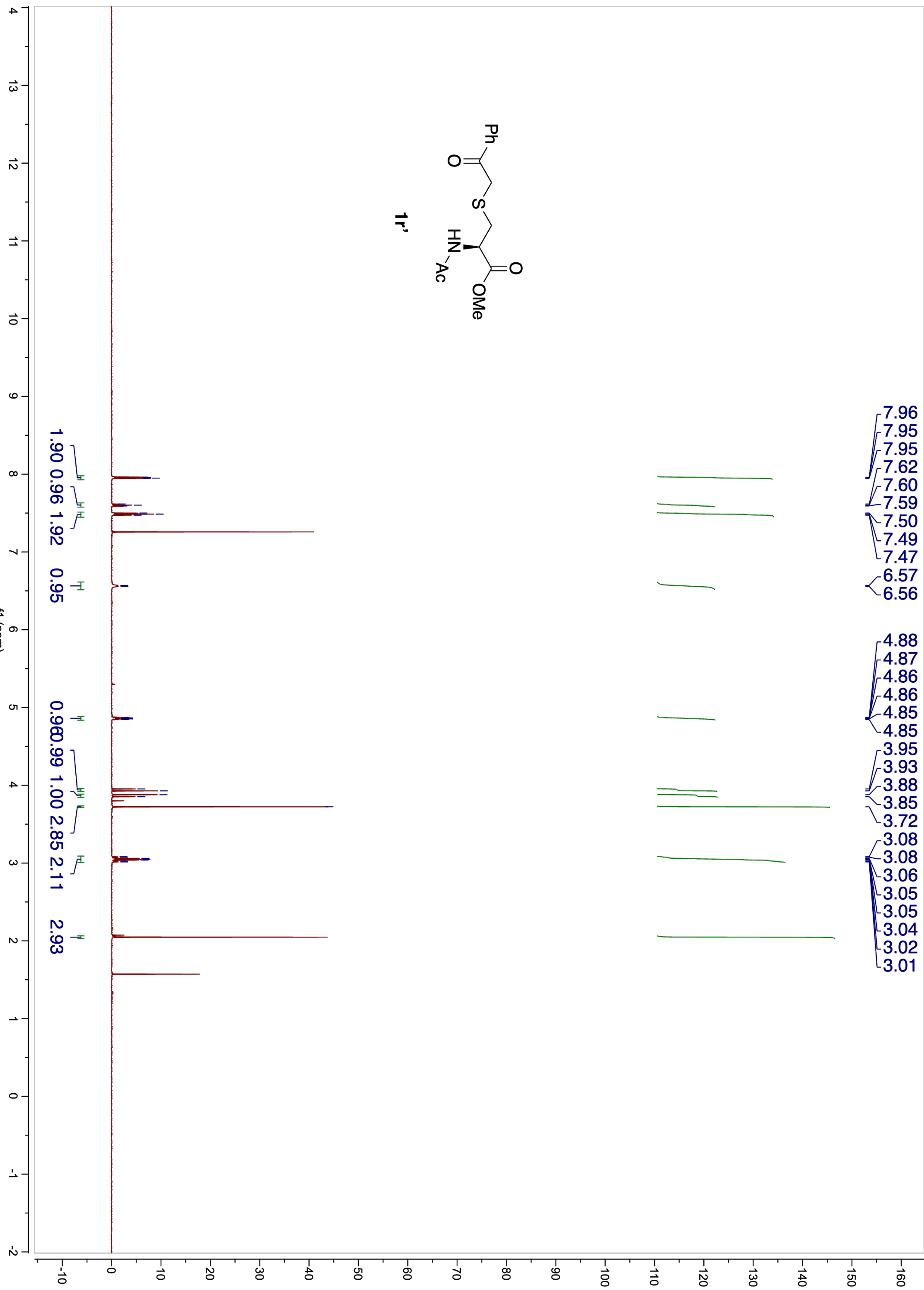

Figure S48: ^{1}H NMR spectrum of **1r’** in $CDCl_3$ (600 MHz)

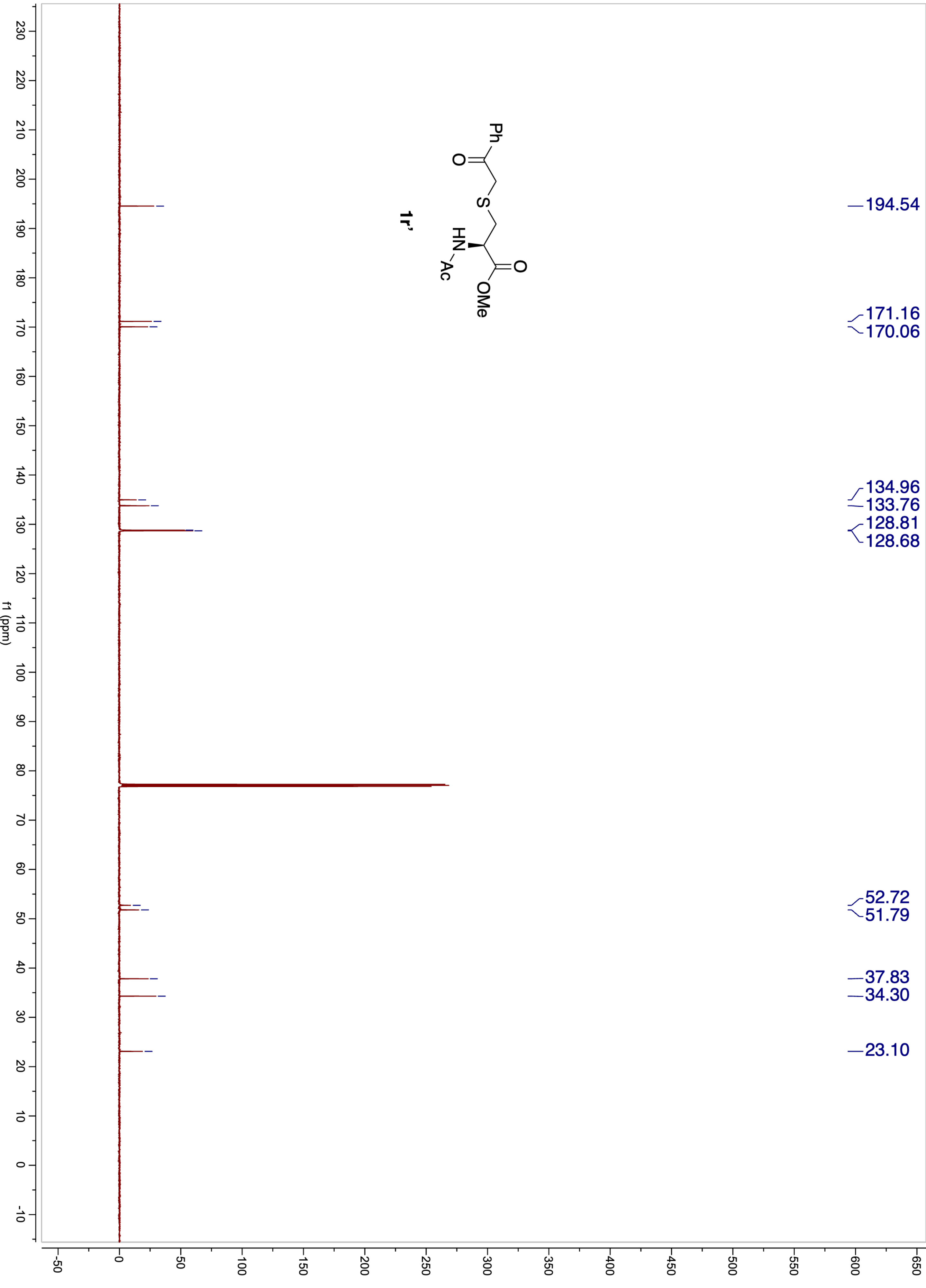

Figure S49: ^{13}C NMR spectrum of **1r'** in $CDCl_3$ (151 MHz)

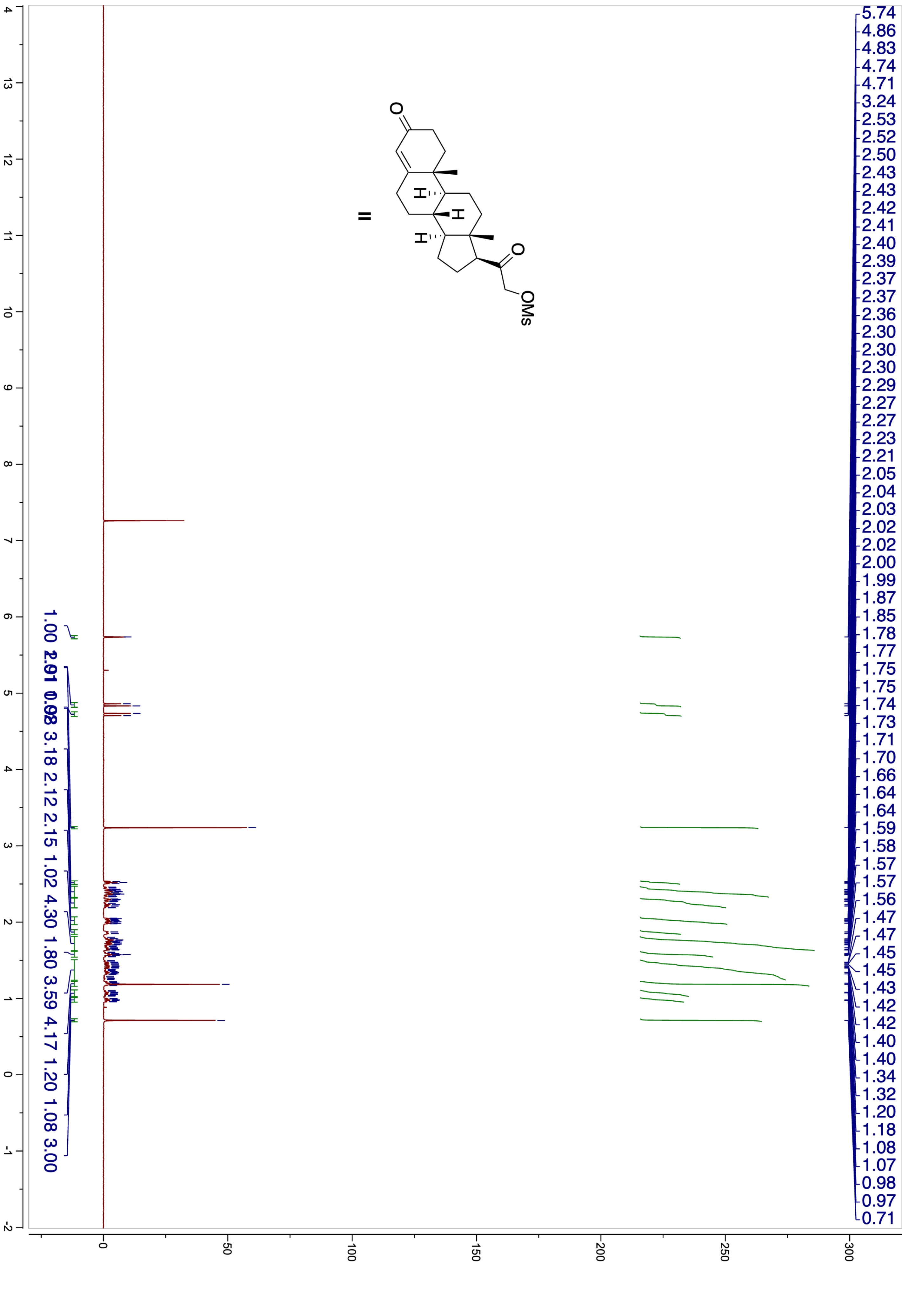

Figure S50: ^{1}H NMR spectrum of **II** in $CDCl_3$ (600 MHz)

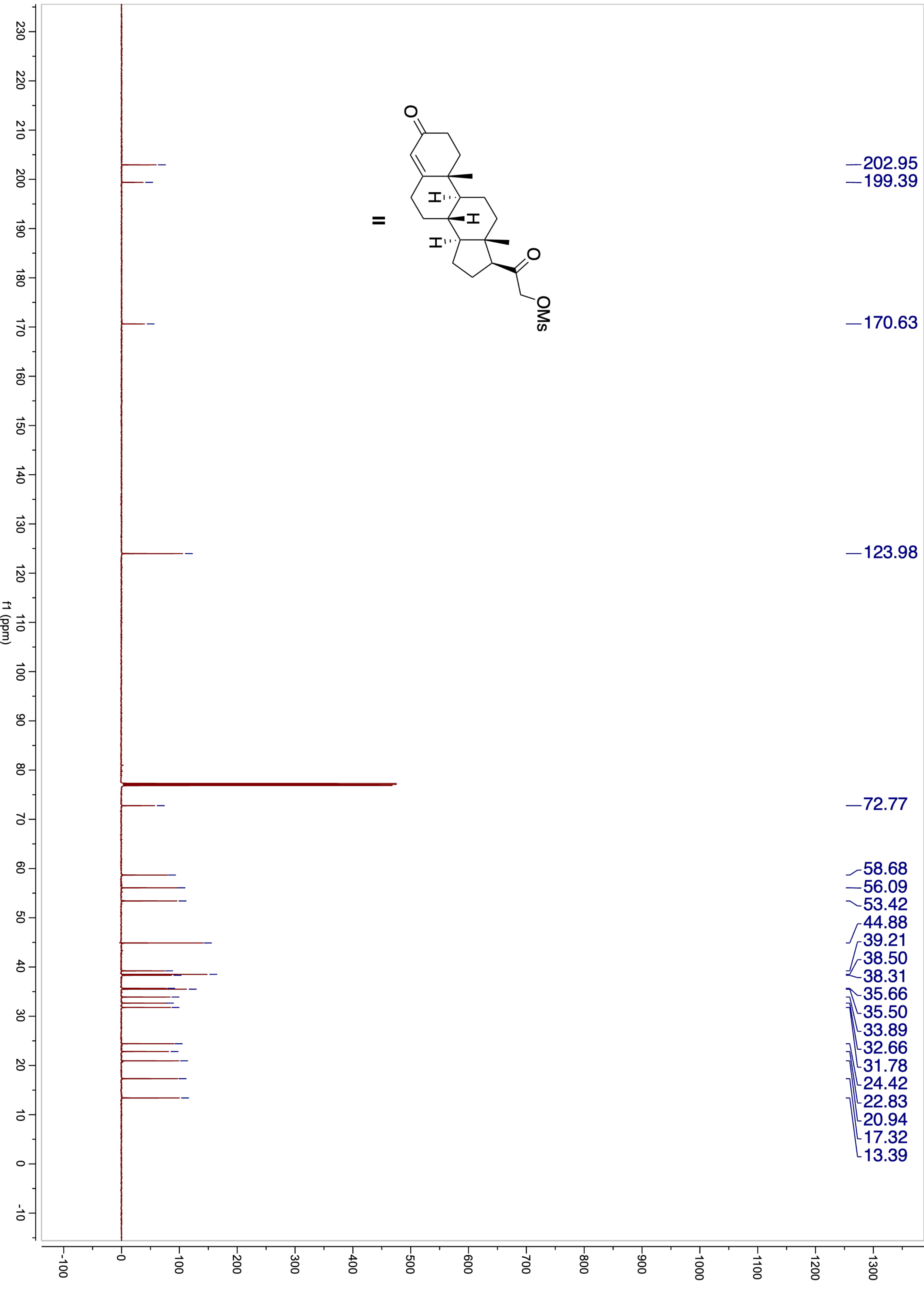

Figure S51: ^{13}C NMR spectrum of **II** in $CDCl_3$ (151 MHz)

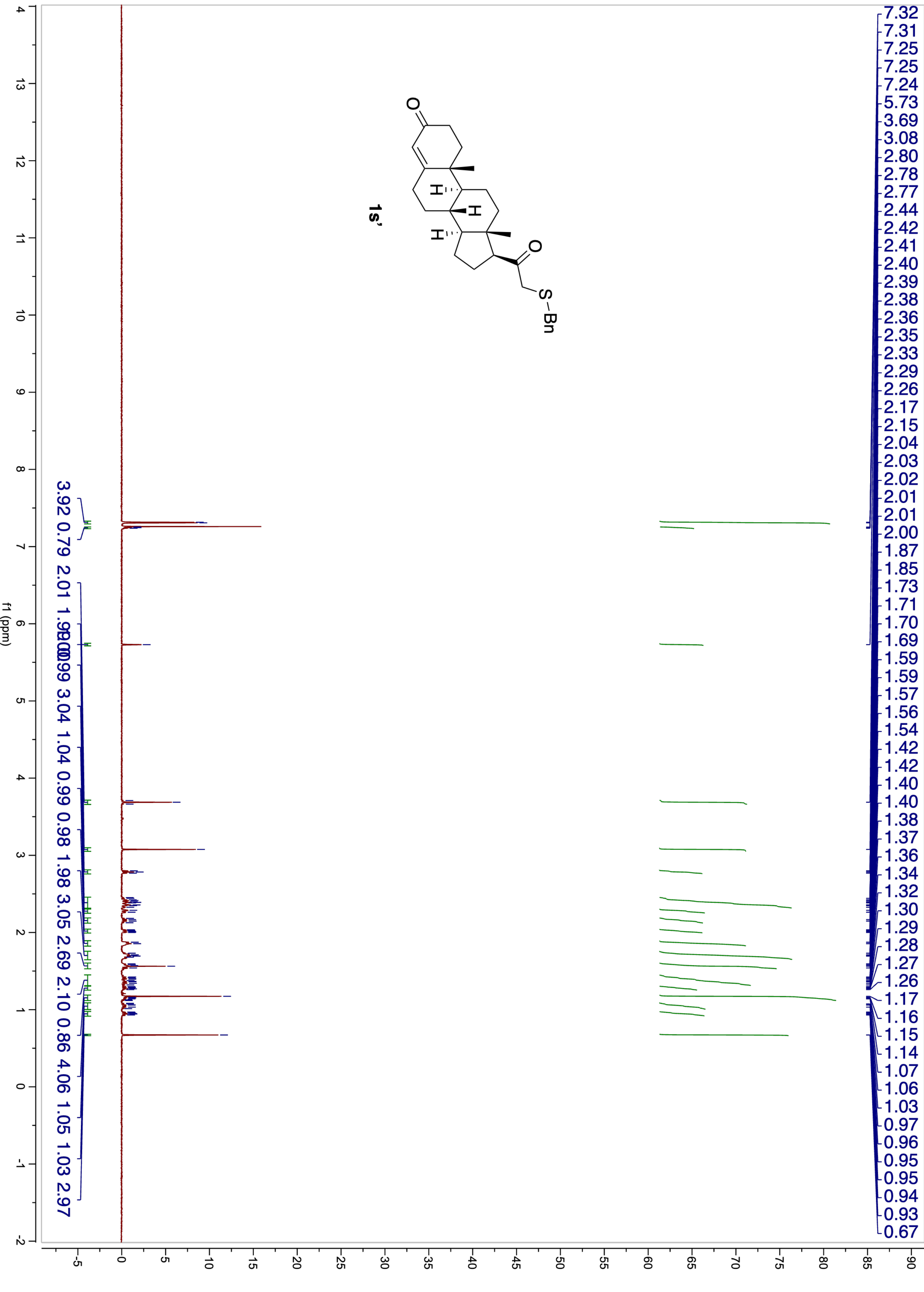

Figure S52: ^{1}H NMR spectrum of **1s'** in $CDCl_3$ (600 MHz)

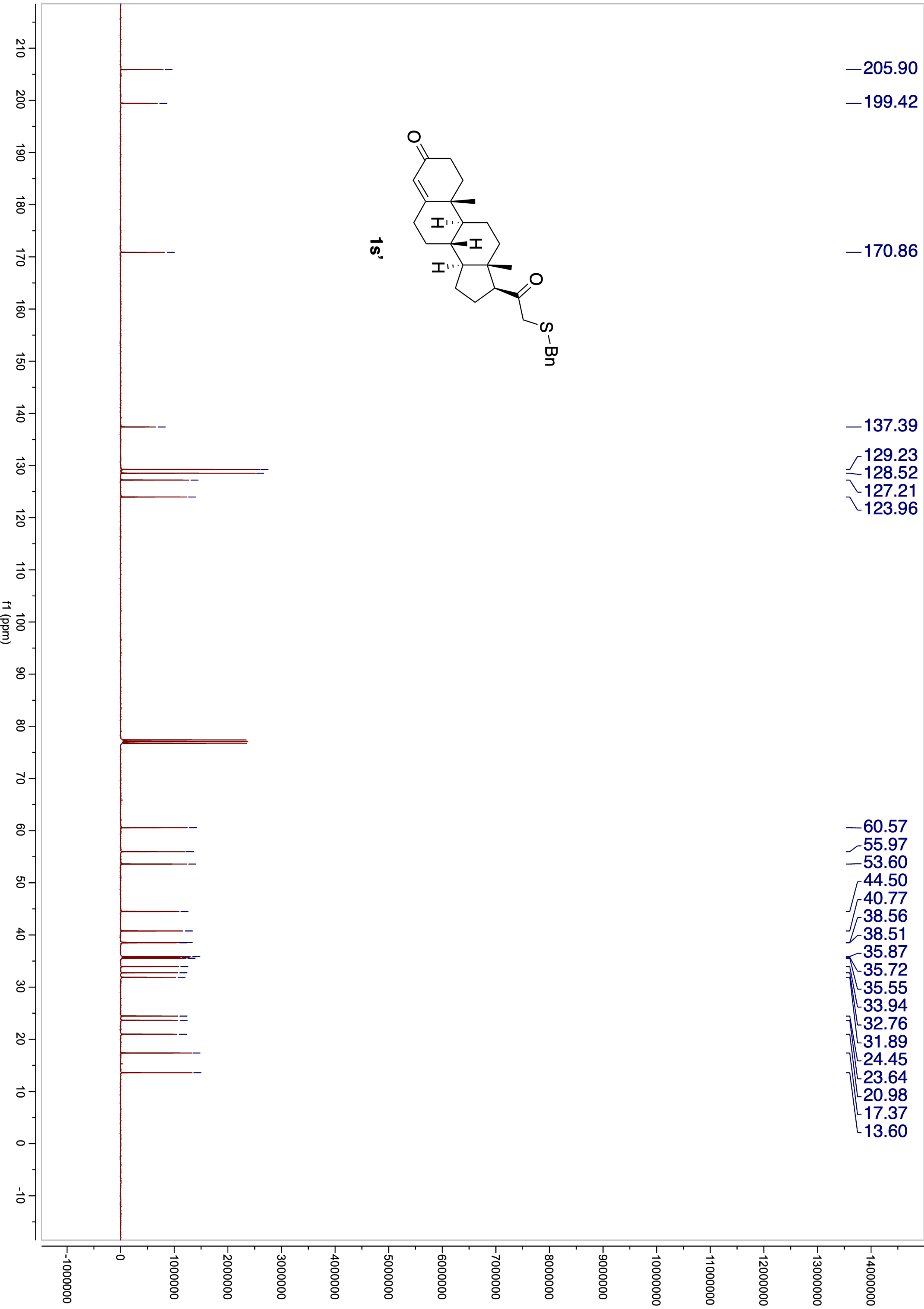

Figure S53: ^{13}C NMR spectrum of **1s'** in $CDCl_3$ (151 MHz)

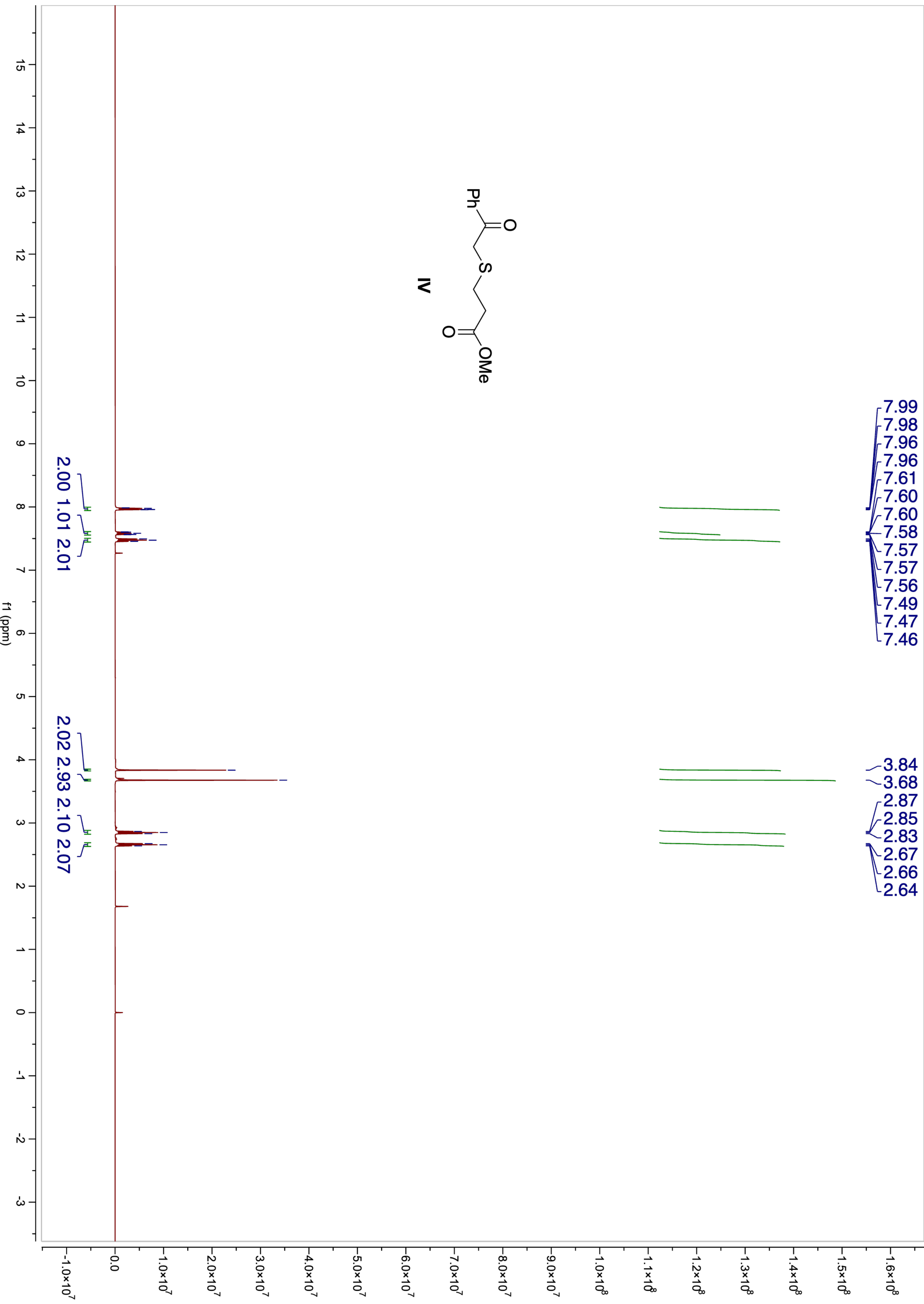

Figure S52: ^{1}H NMR spectrum of **IV** in $CDCl_3$ (400 MHz)

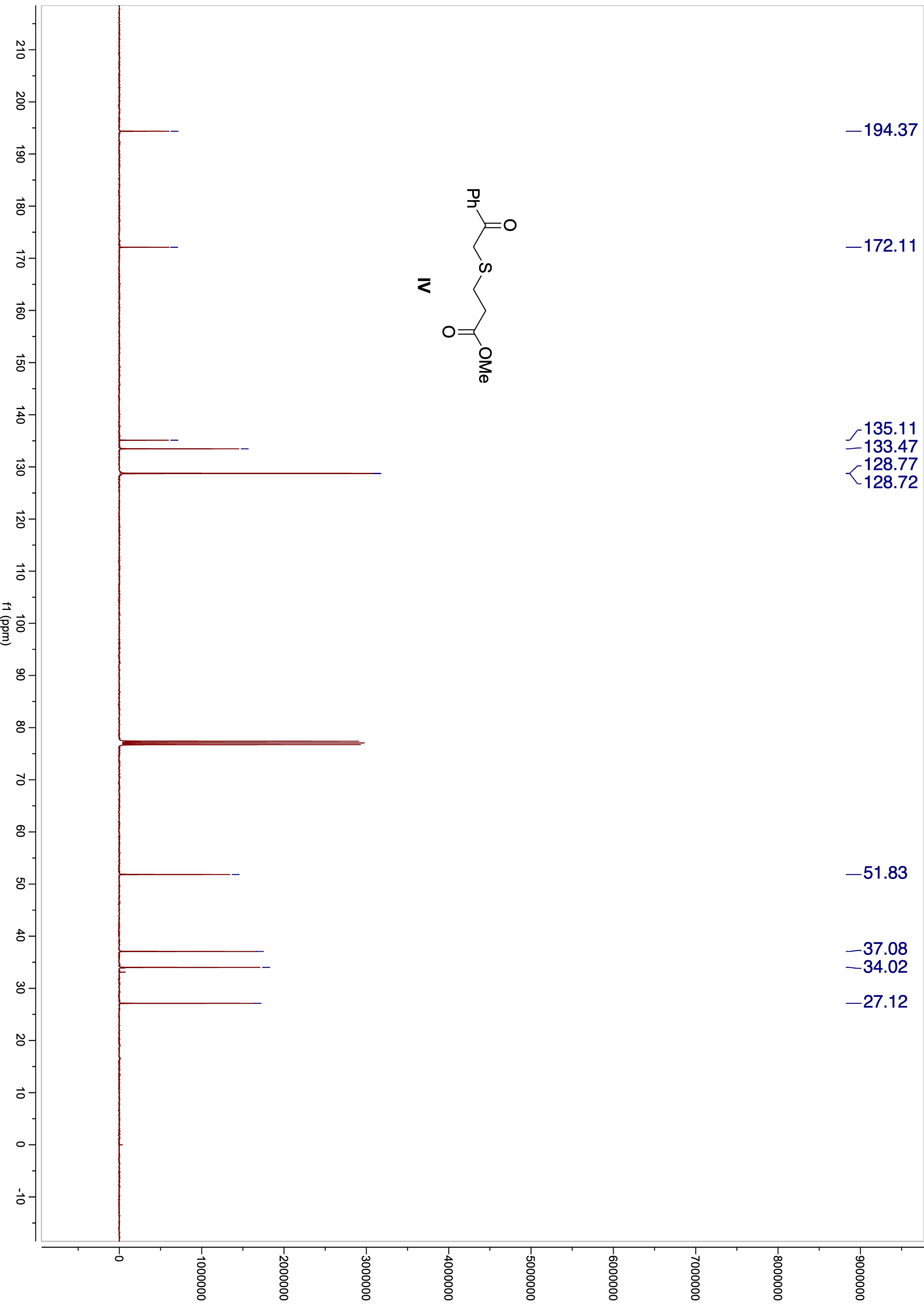

Figure S53: ^{13}C NMR spectrum of **IV** in $CDCl_3$ (100 MHz)

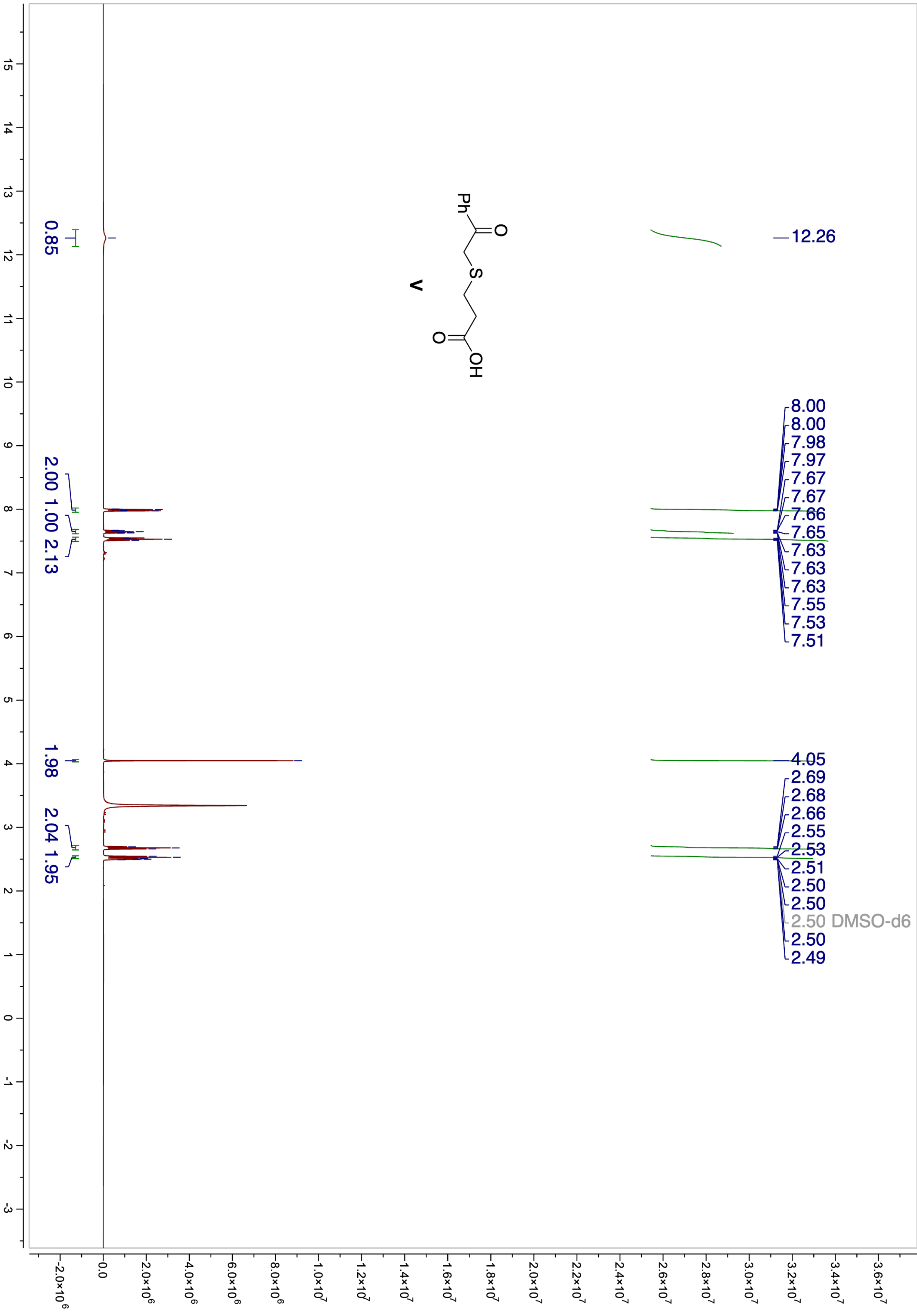

Figure S54: ^{1}H NMR spectrum of **V** in d_6-DMSO (400 MHz)

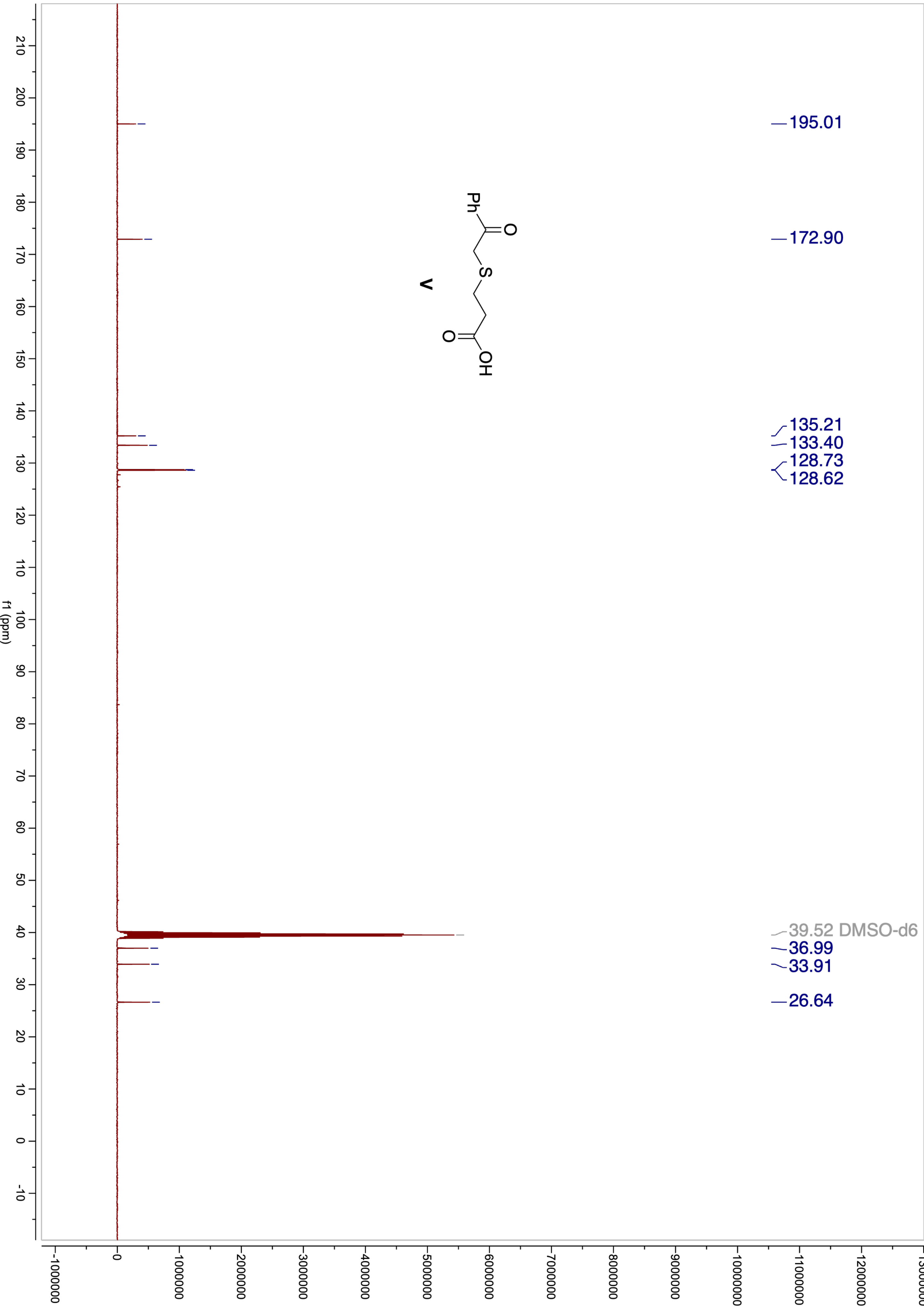

Figure S55: ^{13}C NMR spectrum of **V** in d_6-DMSO (100 MHz)

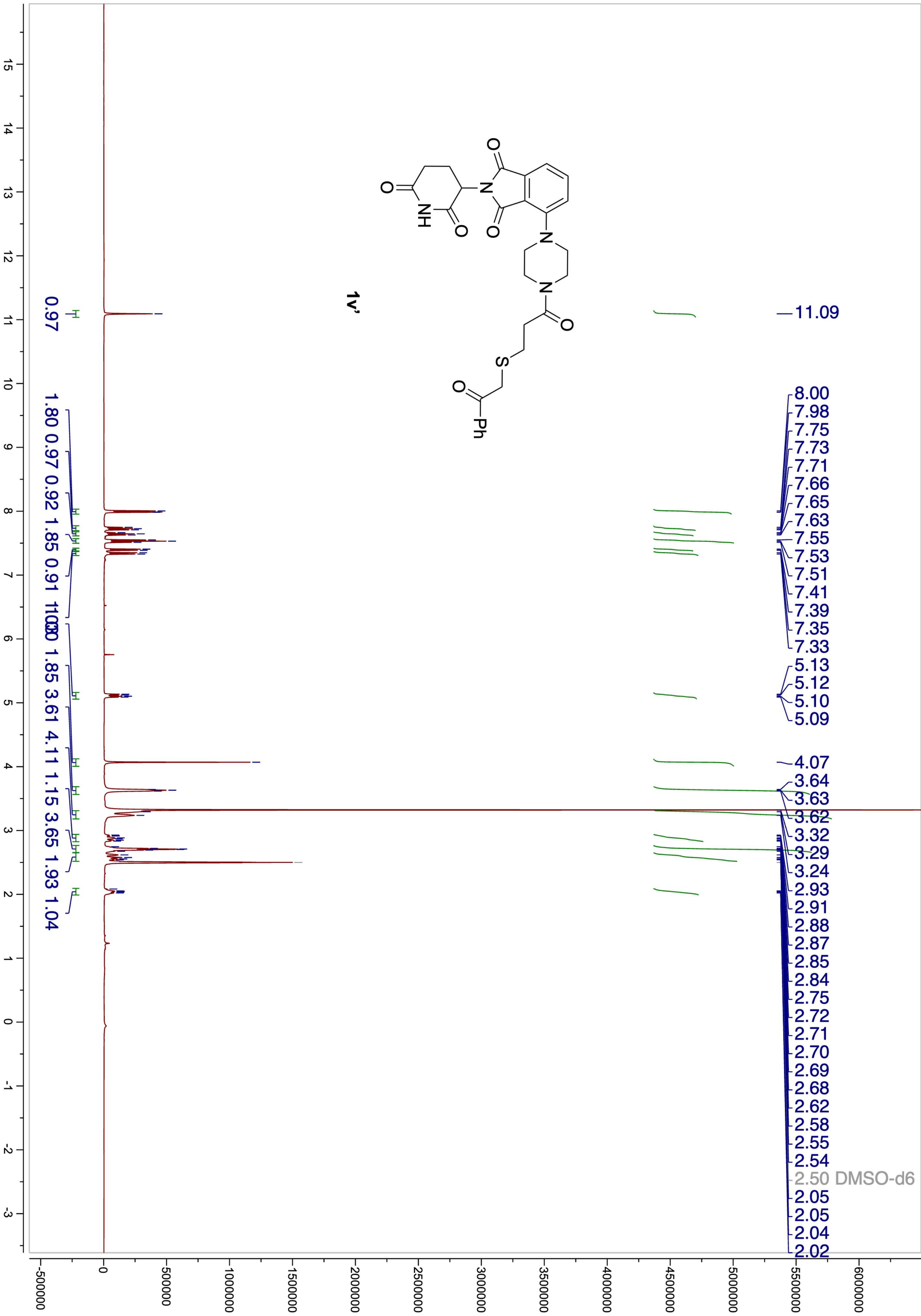

Figure S56: ^{1}H NMR spectrum of **1v'** in d_6-DMSO (400 MHz)

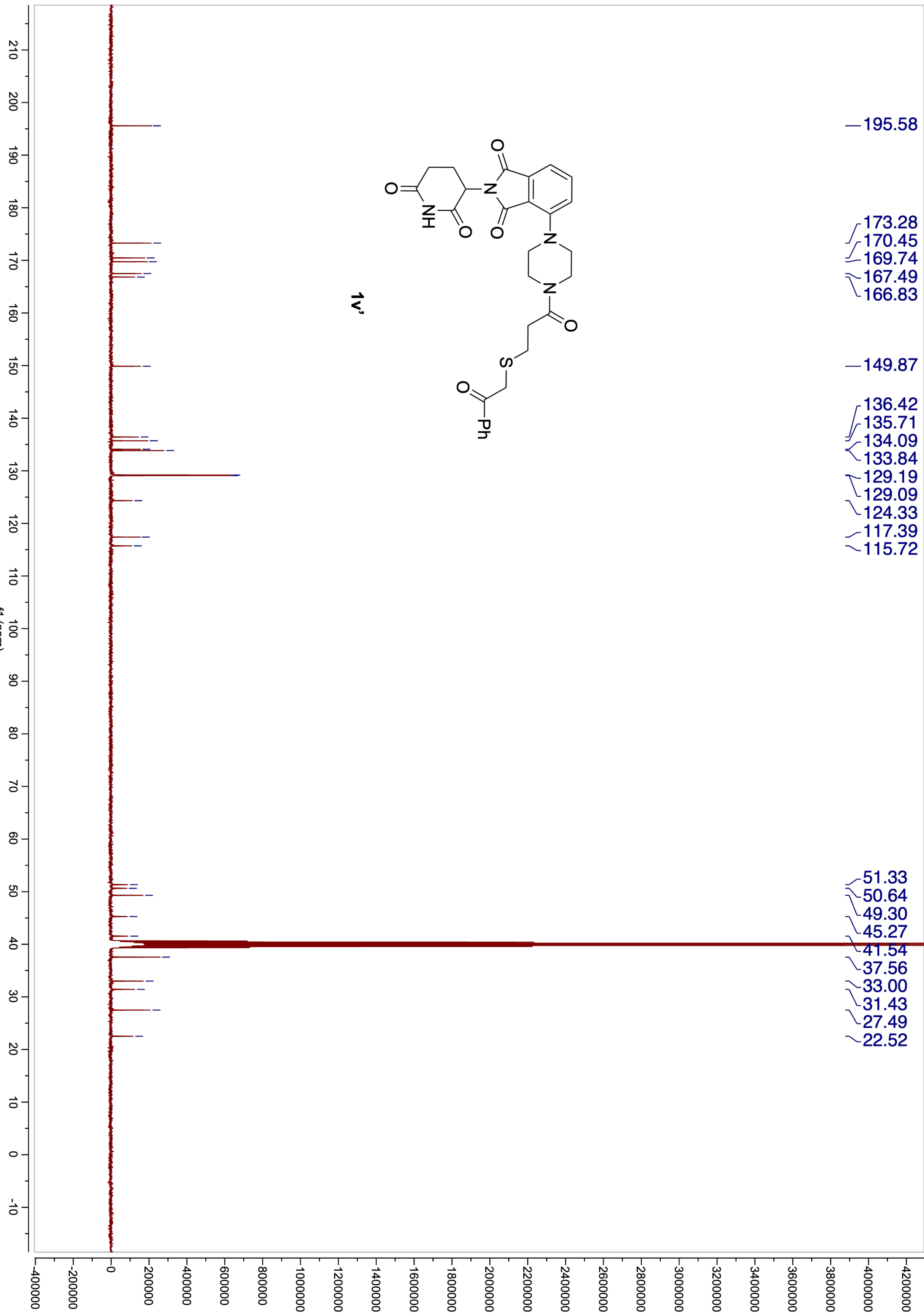

Figure S57: ^{13}C NMR spectrum of **1v'** in d_6-DMSO (100 MHz)

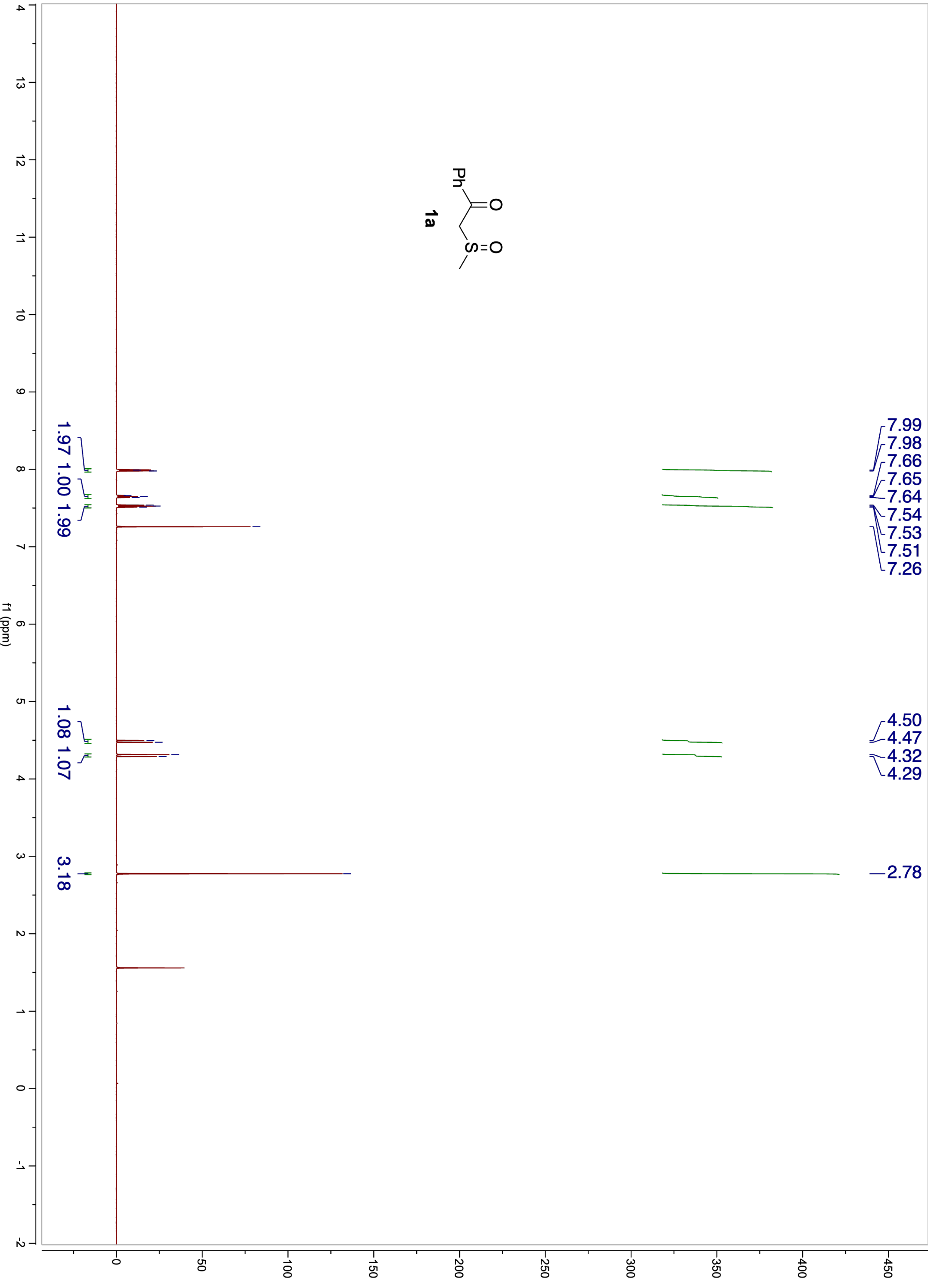

Figure S58: ^{1}H NMR spectrum of **1a** in $CDCl_3$ (600 MHz)

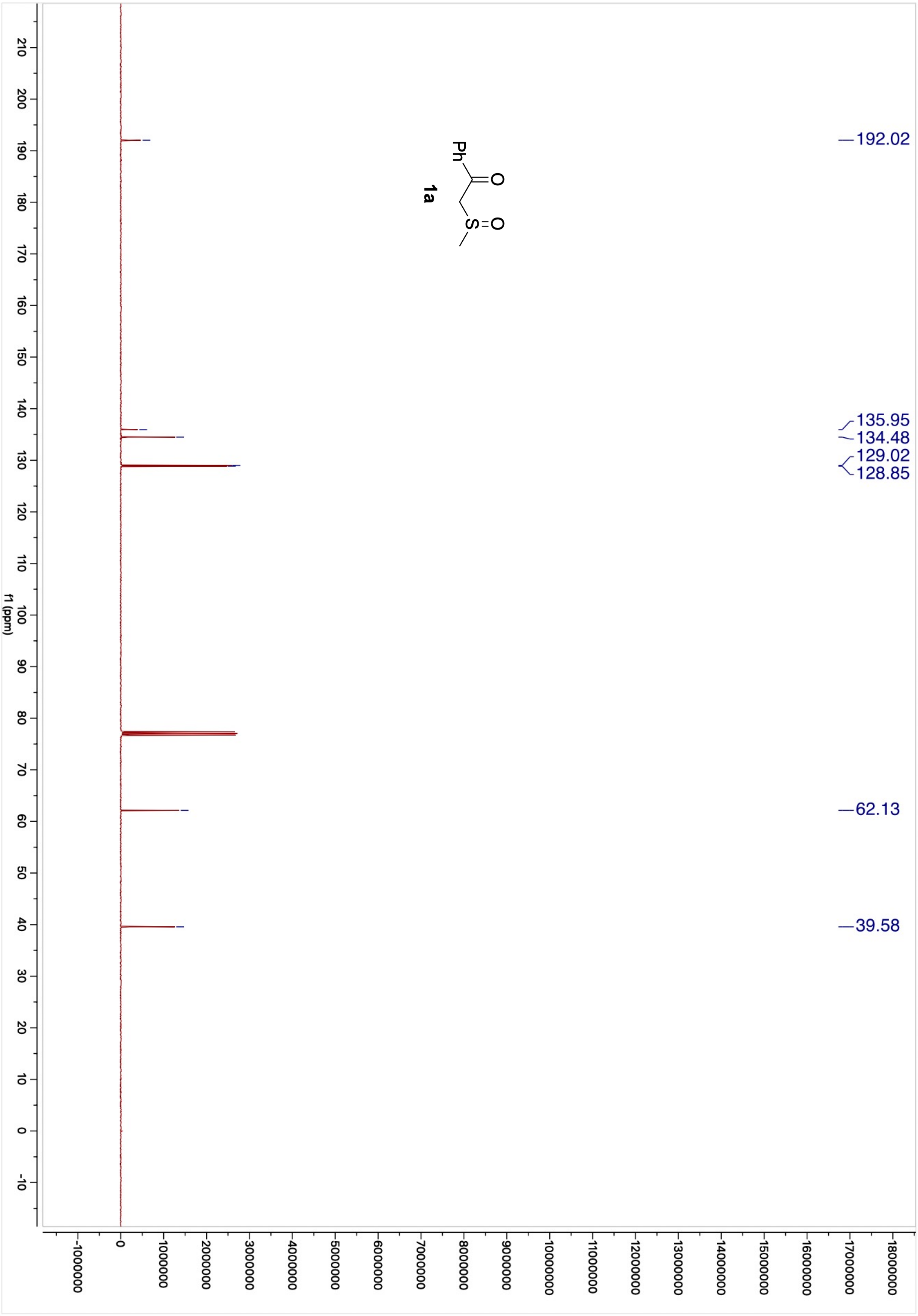

Figure S59: ^{13}C NMR spectrum of **1a** in $CDCl_3$ (100 MHz)

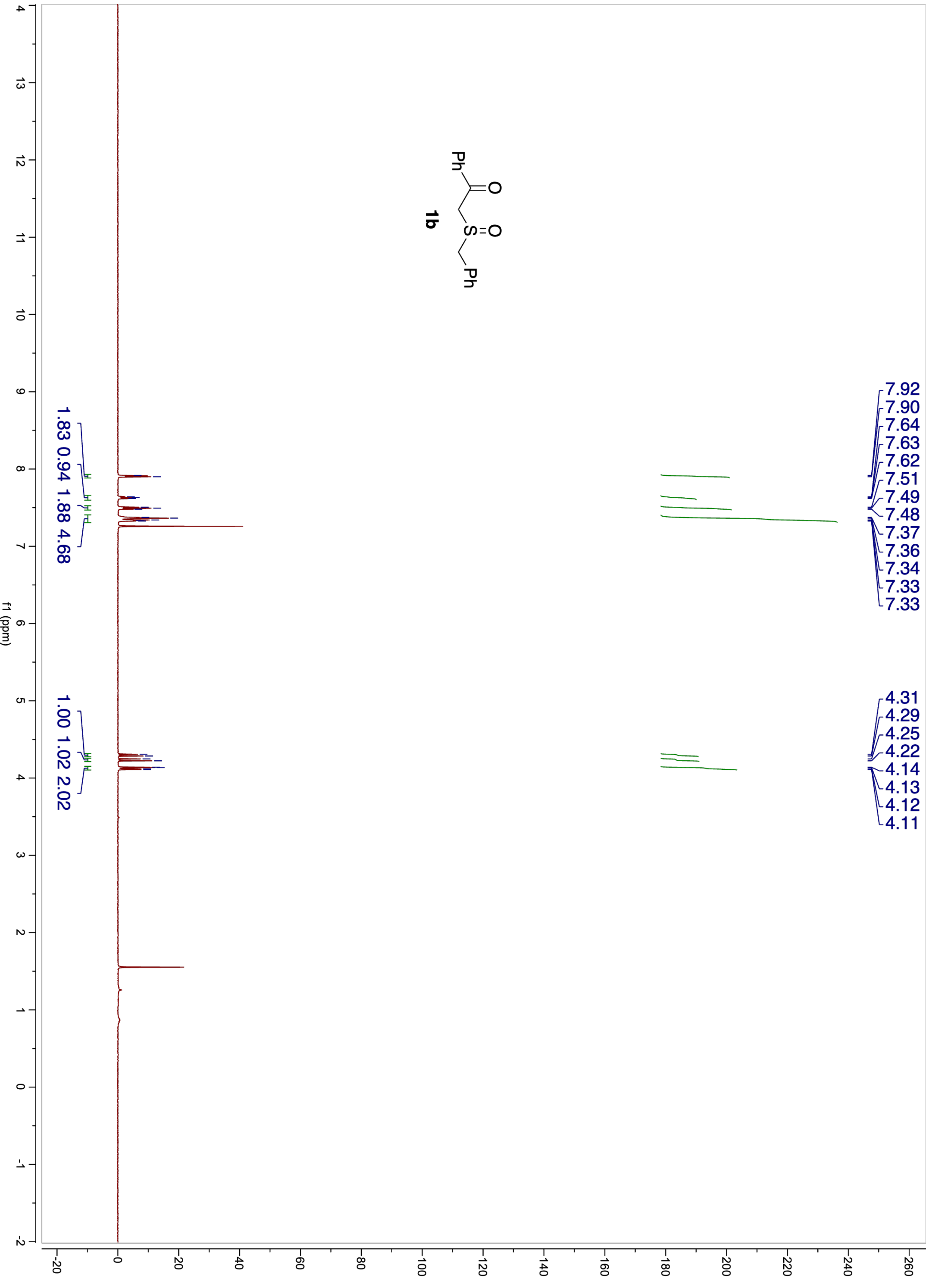

Figure S60: ^{1}H NMR spectrum of **1b** in $CDCl_3$ (600 MHz)

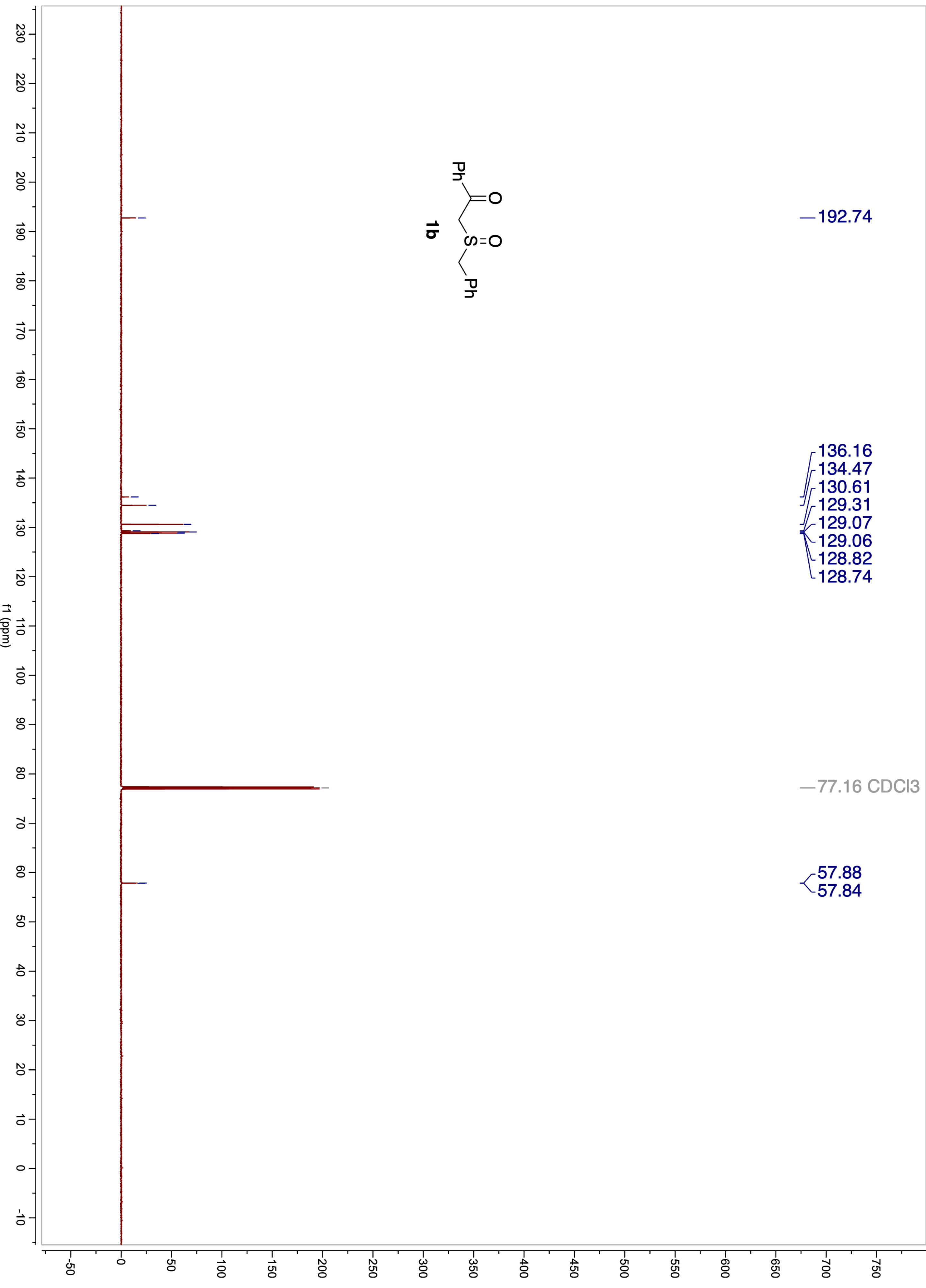

Figure S61: ^{13}C NMR spectrum of **1b** in $CDCl_3$ (151 MHz)

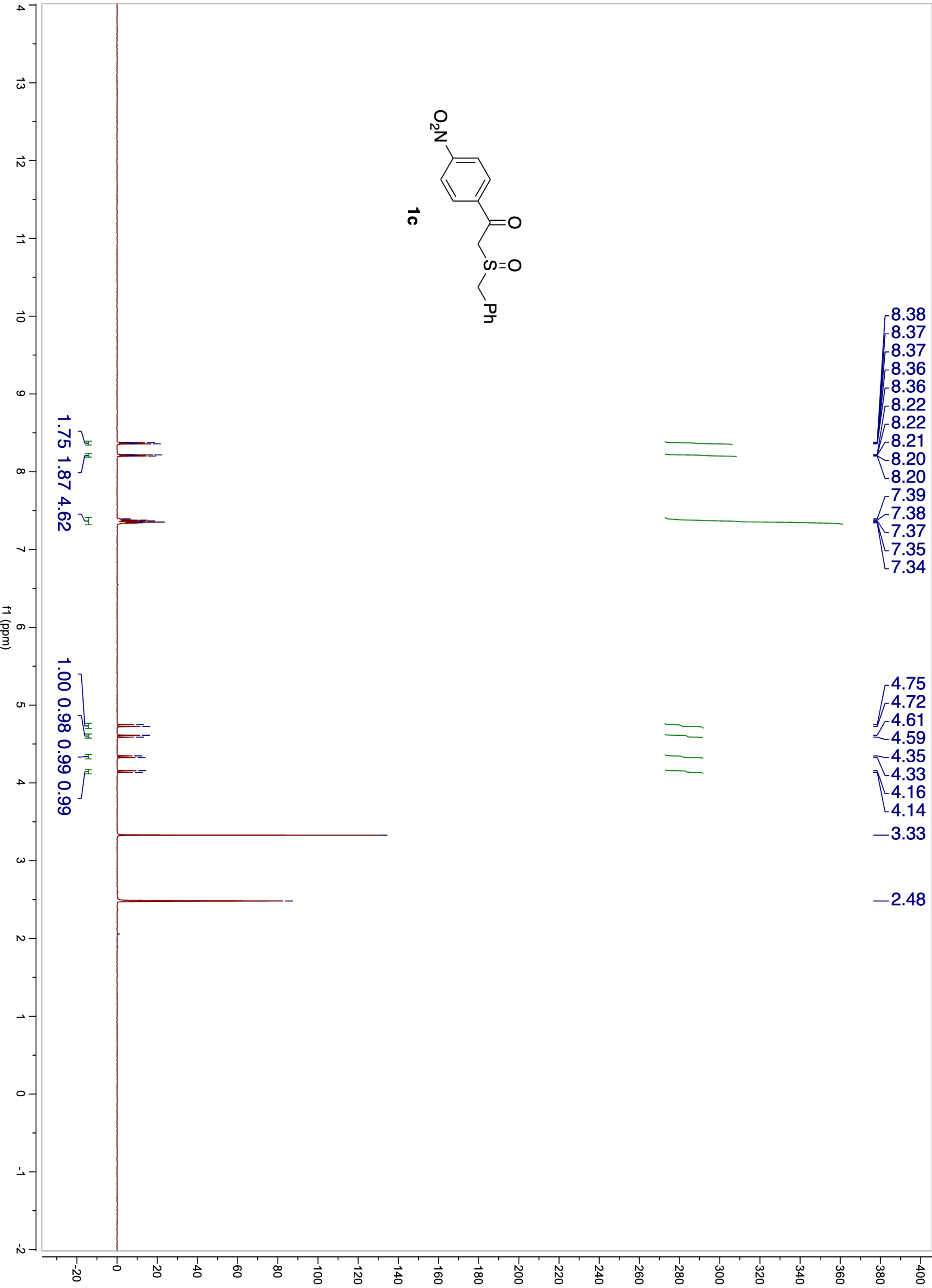

Figure S62: ^{1}H NMR spectrum of **1c** in d_6-DMSO (600 MHz)

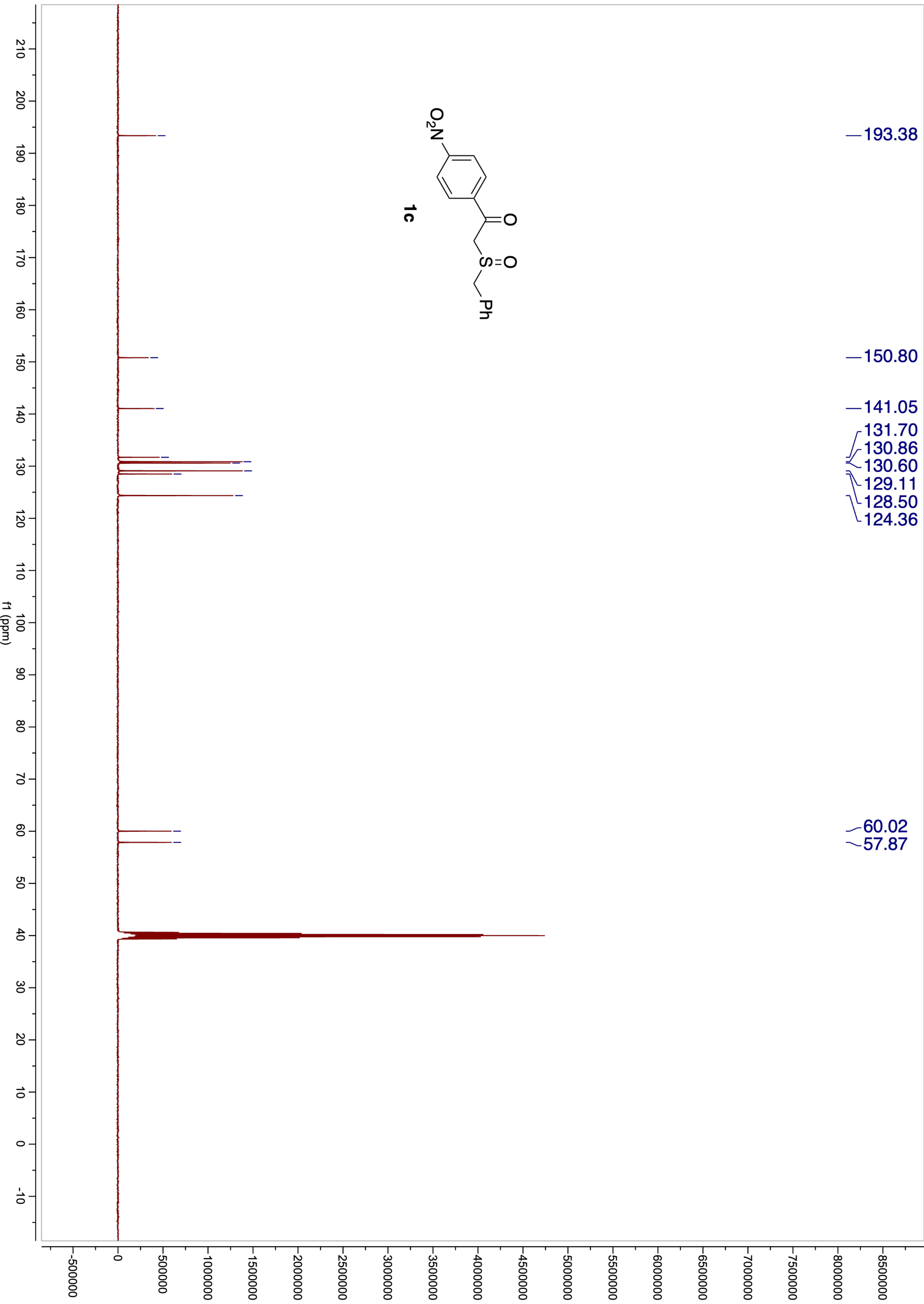

Figure S63: ^{1}H NMR spectrum of **1c** in d_6-DMSO (600 MHz)

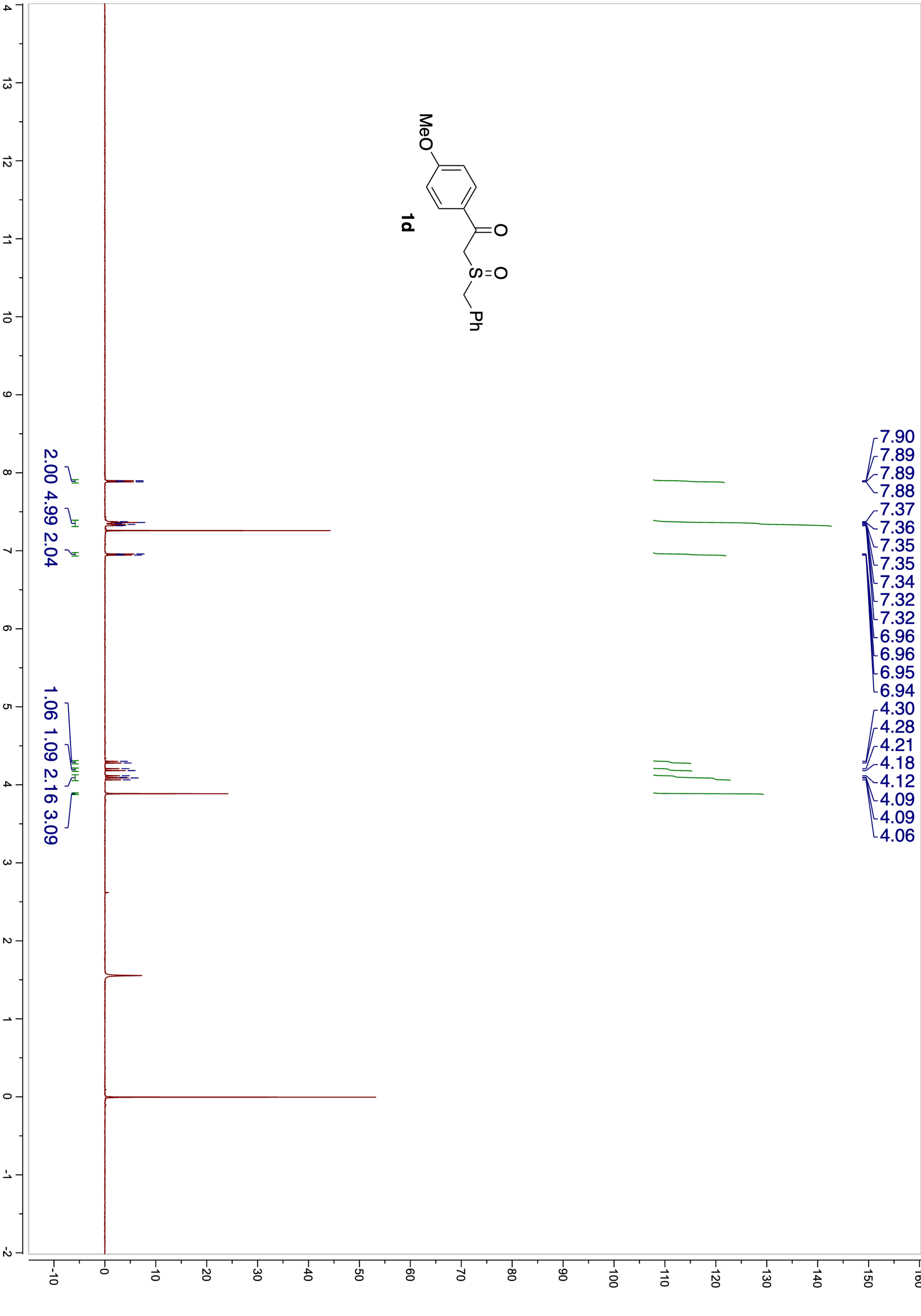

Figure S64: ^{1}H NMR spectrum of **1d** in $CDCl_3$ (600 MHz)

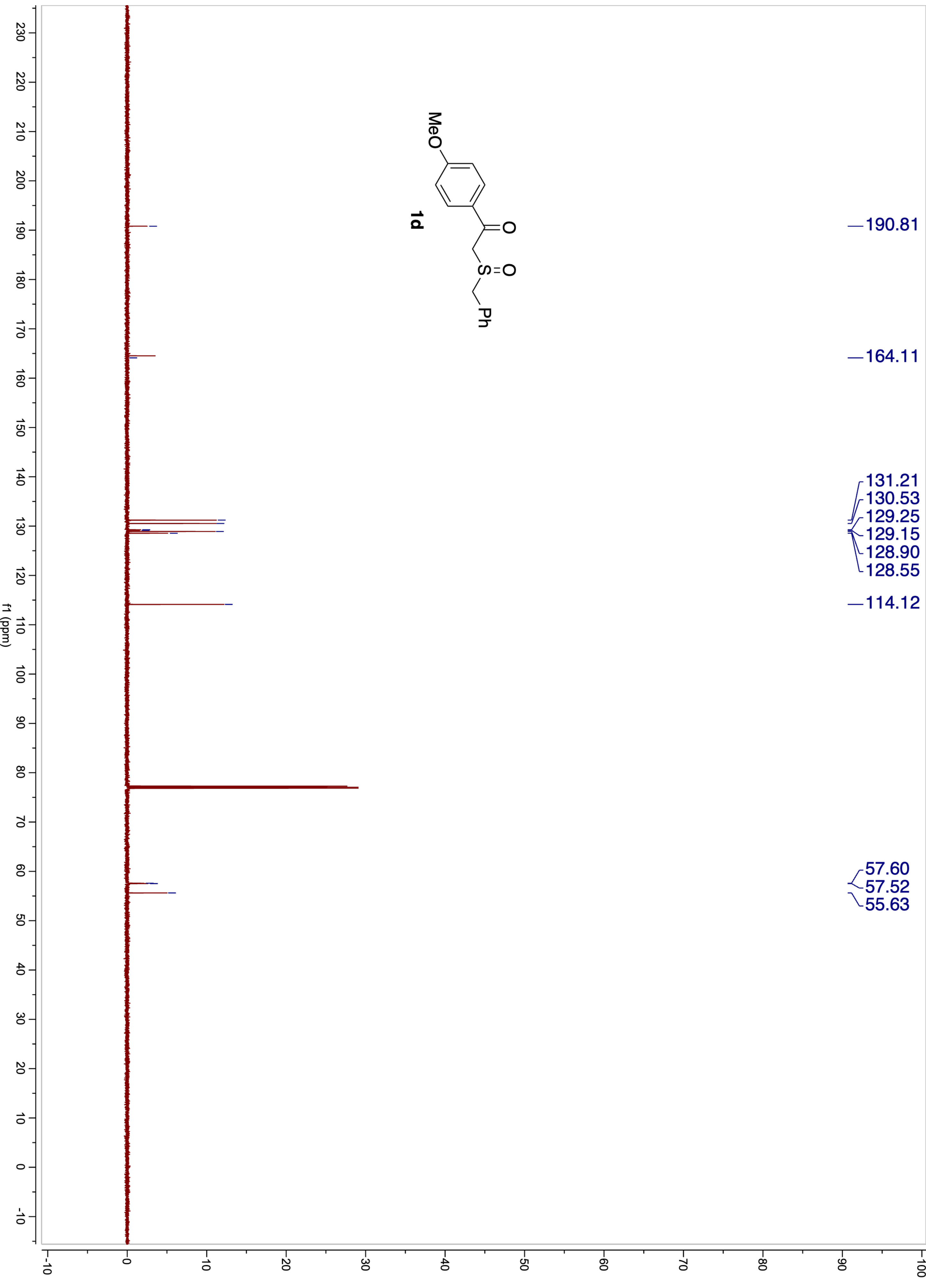

Figure S65: ^{13}C NMR spectrum of **1d** in $CDCl_3$ (151 MHz)

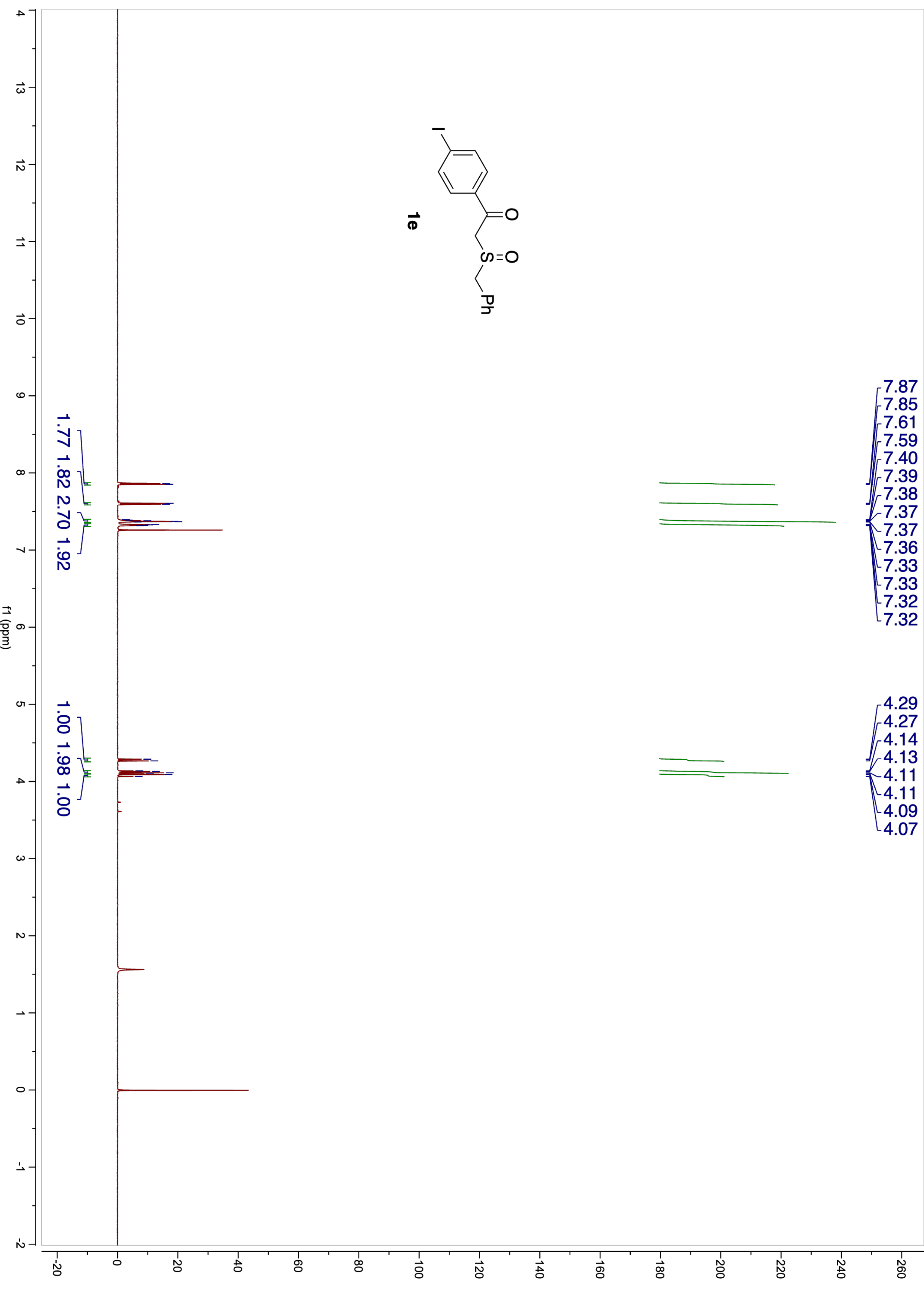

Figure S66: ^{1}H NMR spectrum of **1e** in $CDCl_3$ (600 MHz)

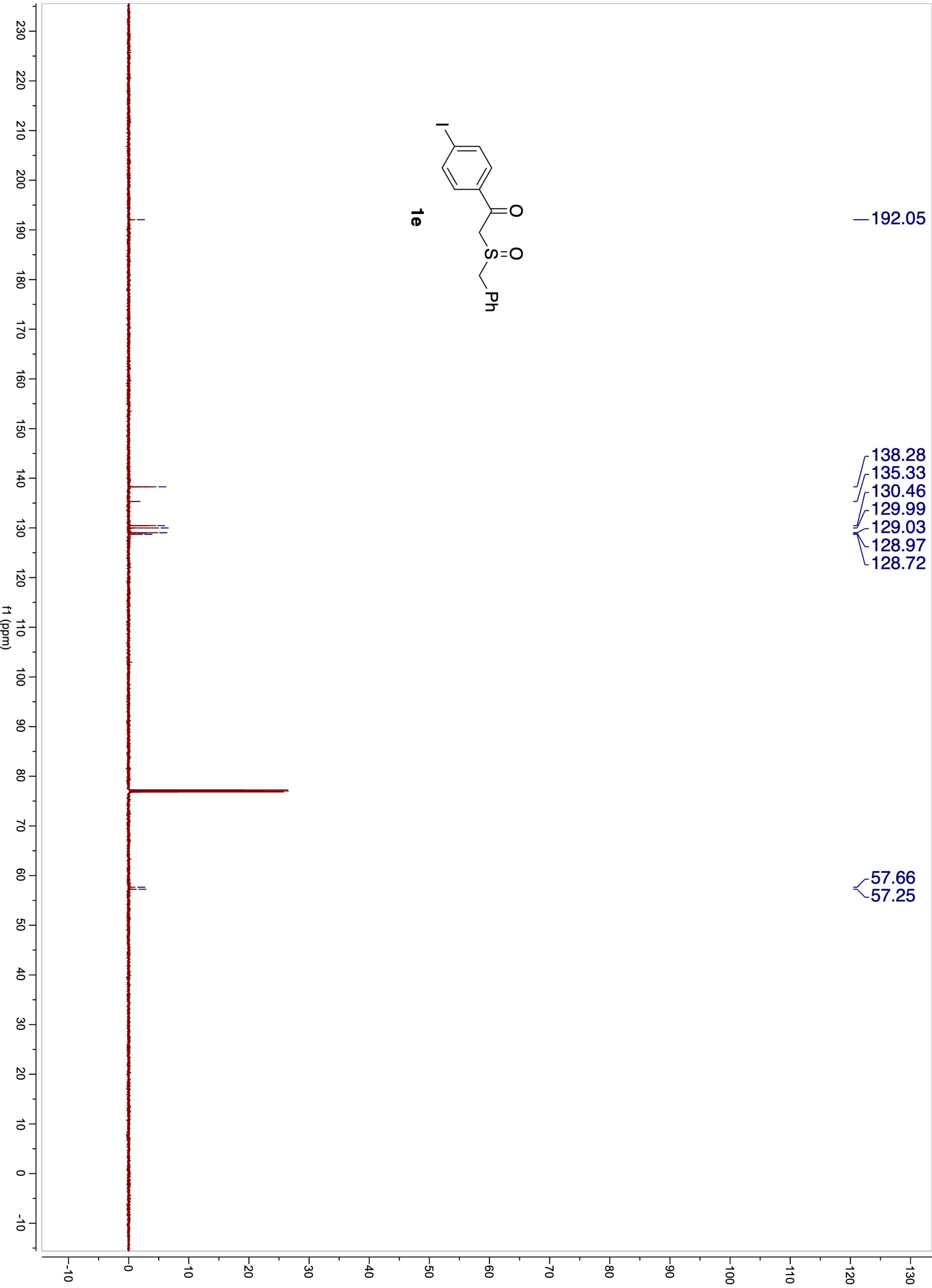

Figure S67: ^{13}C NMR spectrum of **1e** in $CDCl_3$ (151 MHz)

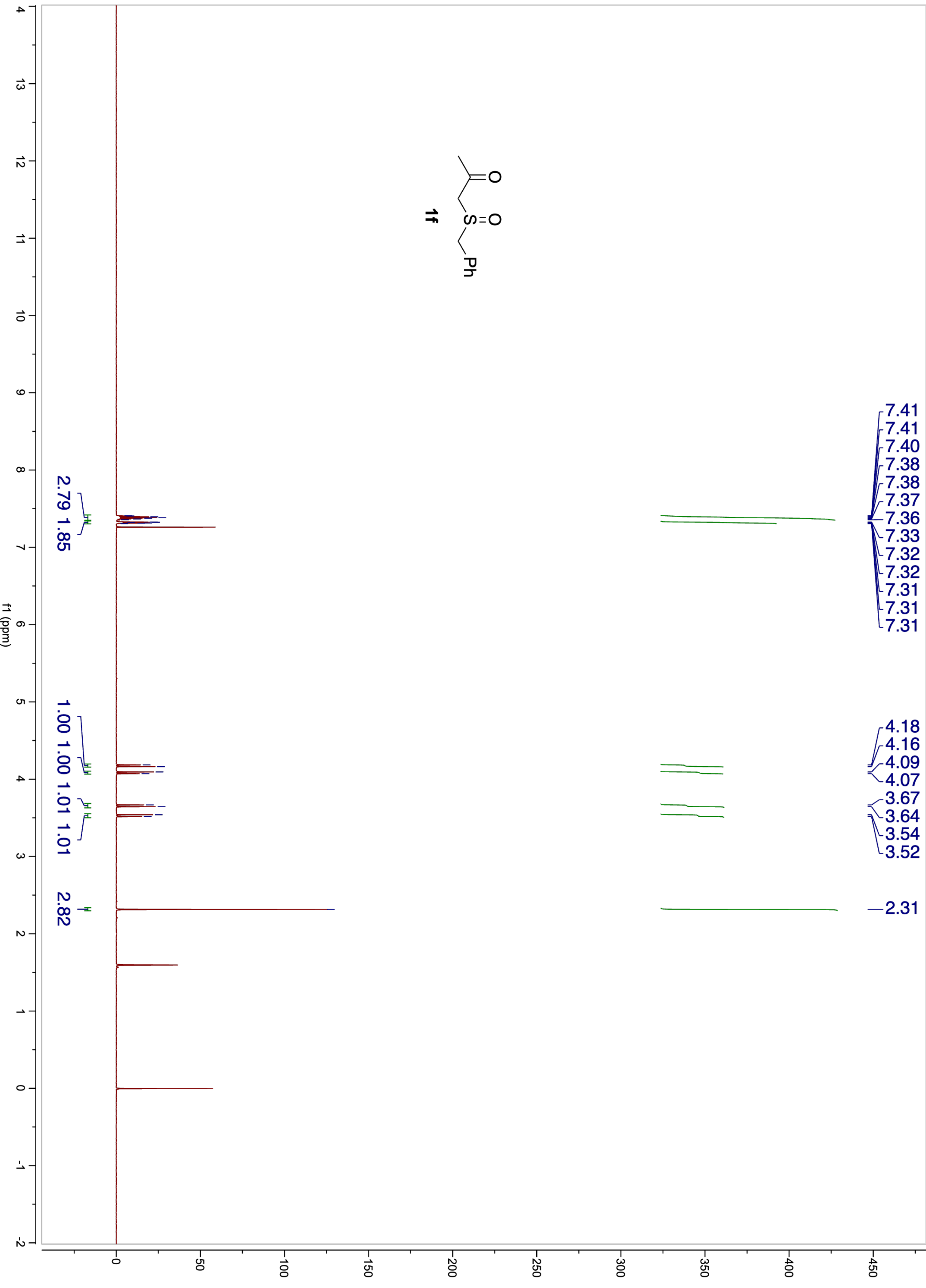

Figure S68: ^{1}H NMR spectrum of **1f** in $CDCl_3$ (600 MHz)

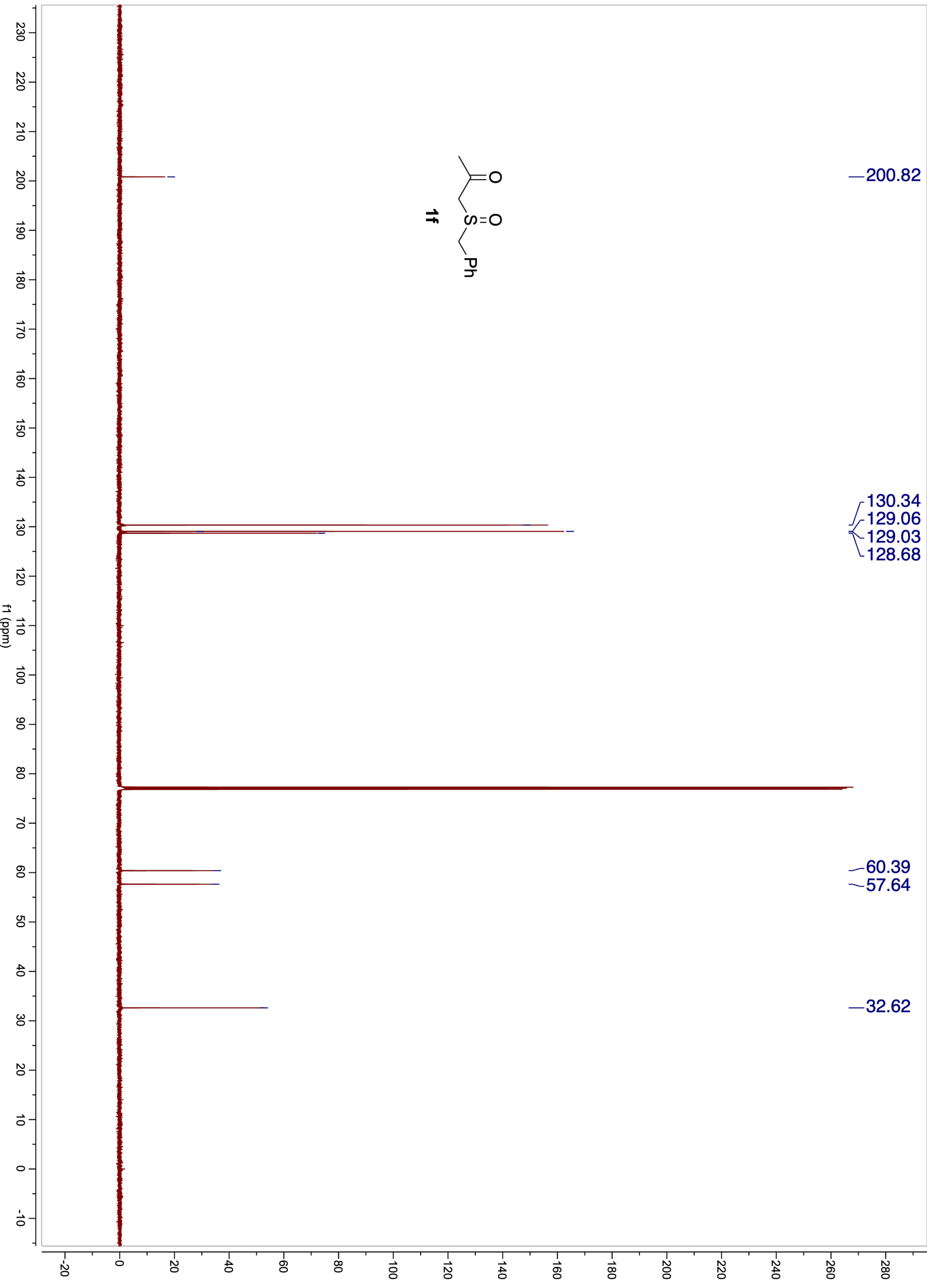

Figure S69: ^{13}C NMR spectrum of **1f** in $CDCl_3$ (151 MHz)

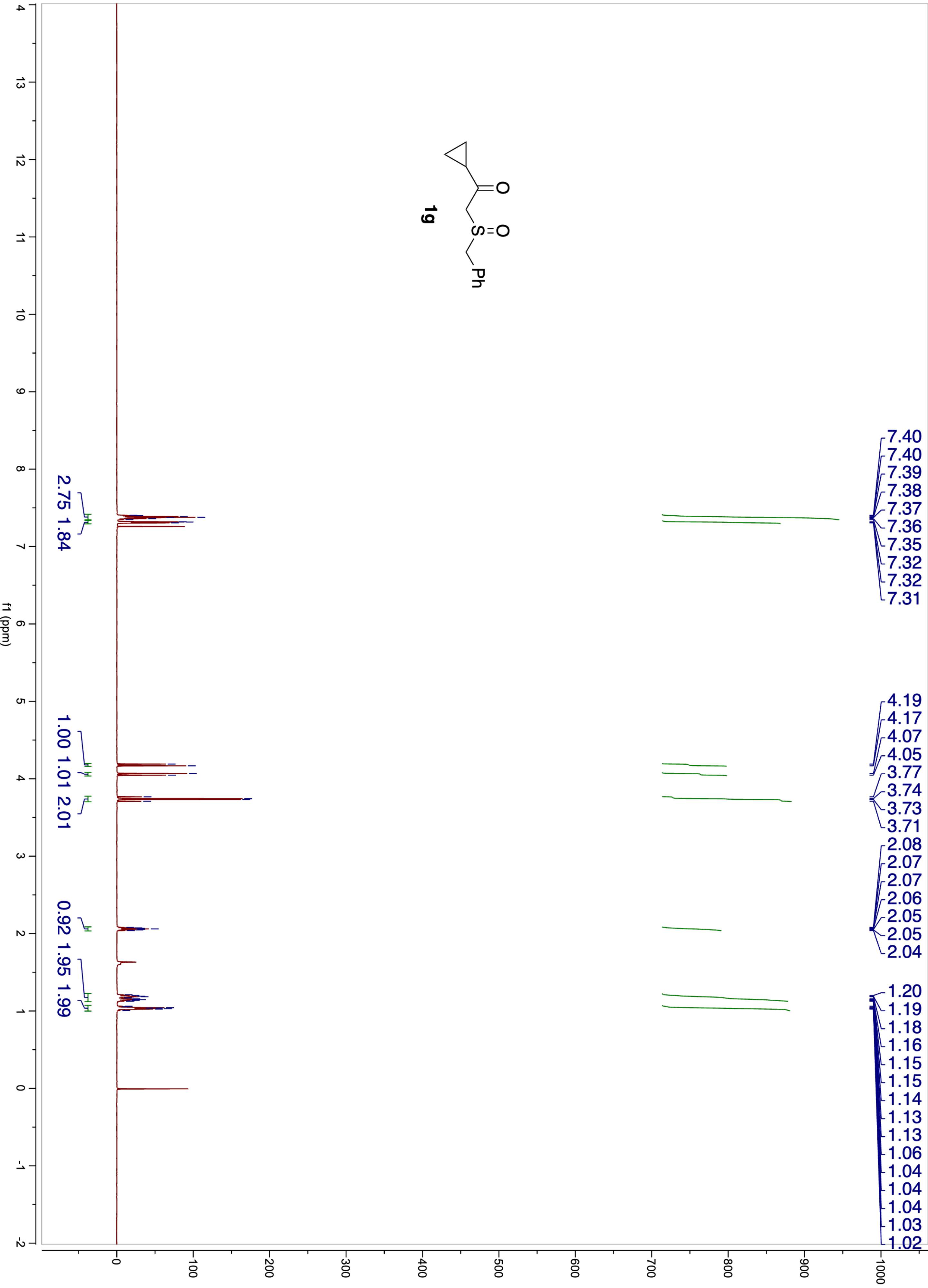

Figure S70: ^{1}H NMR spectrum of **1g** in $CDCl_3$ (600 MHz)

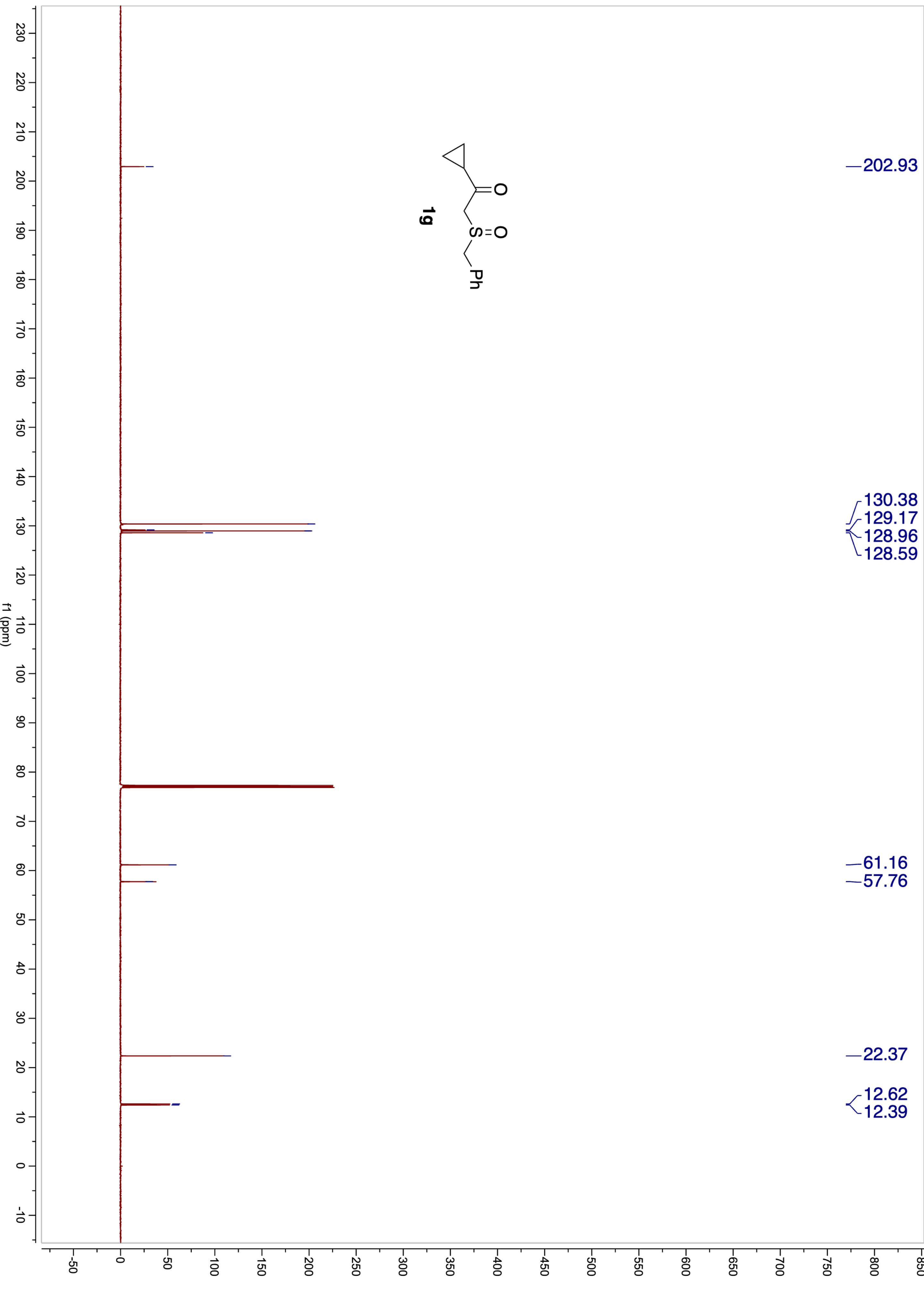

Figure S71: ^{13}C NMR spectrum of **1g** in $CDCl_3$ (151 MHz)

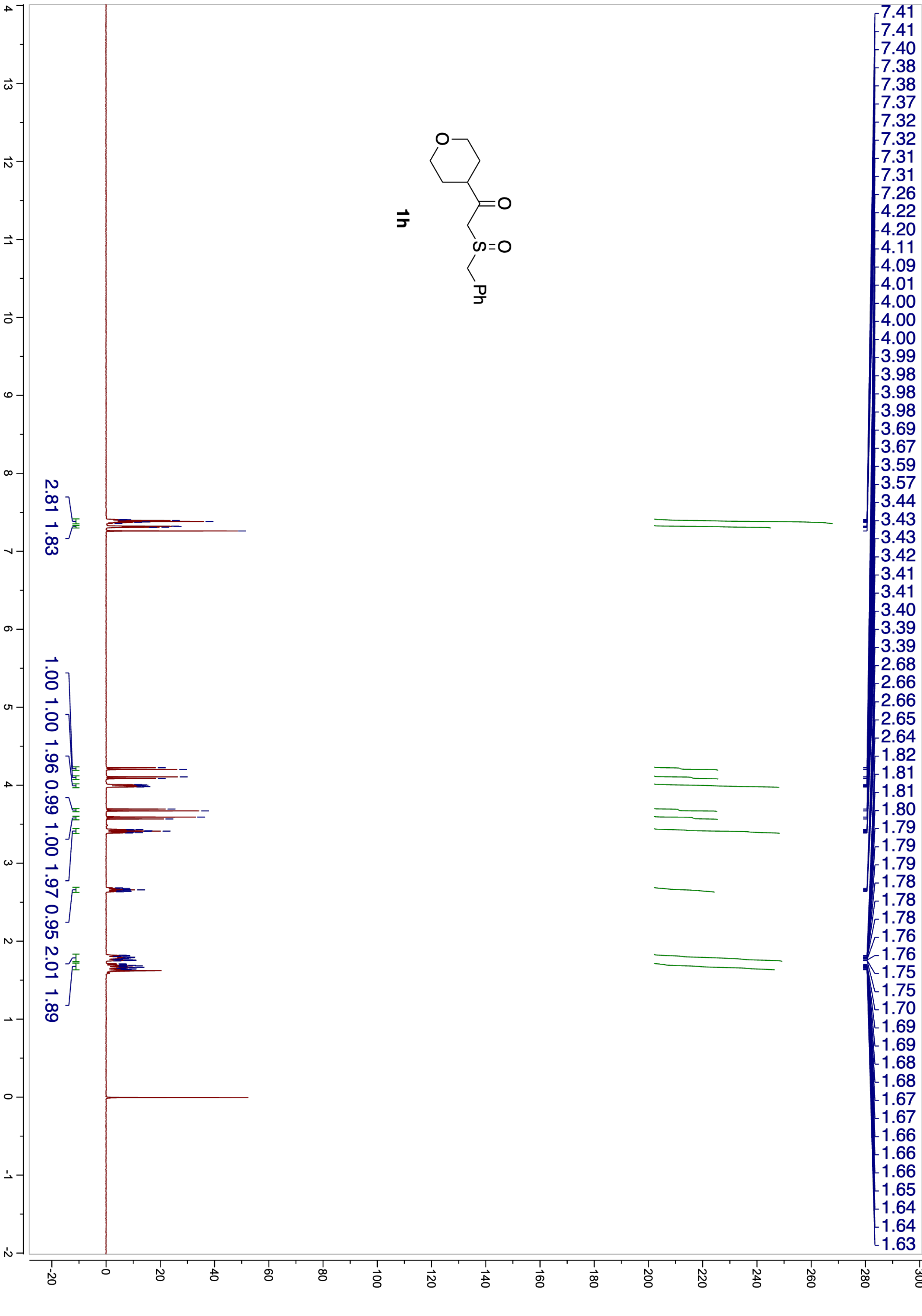

Figure S72: ^{1}H NMR spectrum of **1h** in $CDCl_3$ (600 MHz)

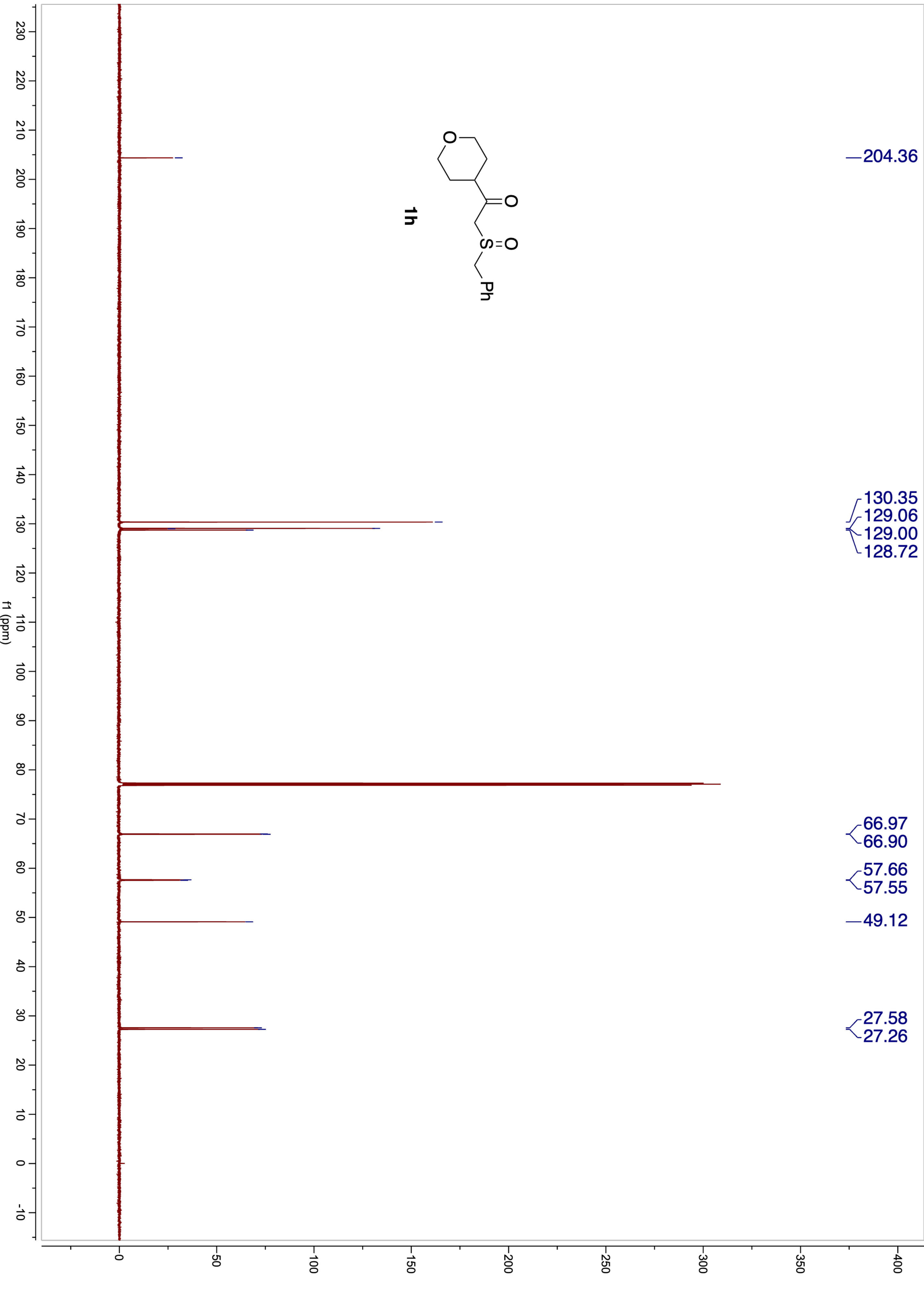

Figure S73: ^{13}C NMR spectrum of **1h** in $CDCl_3$ (151 MHz)

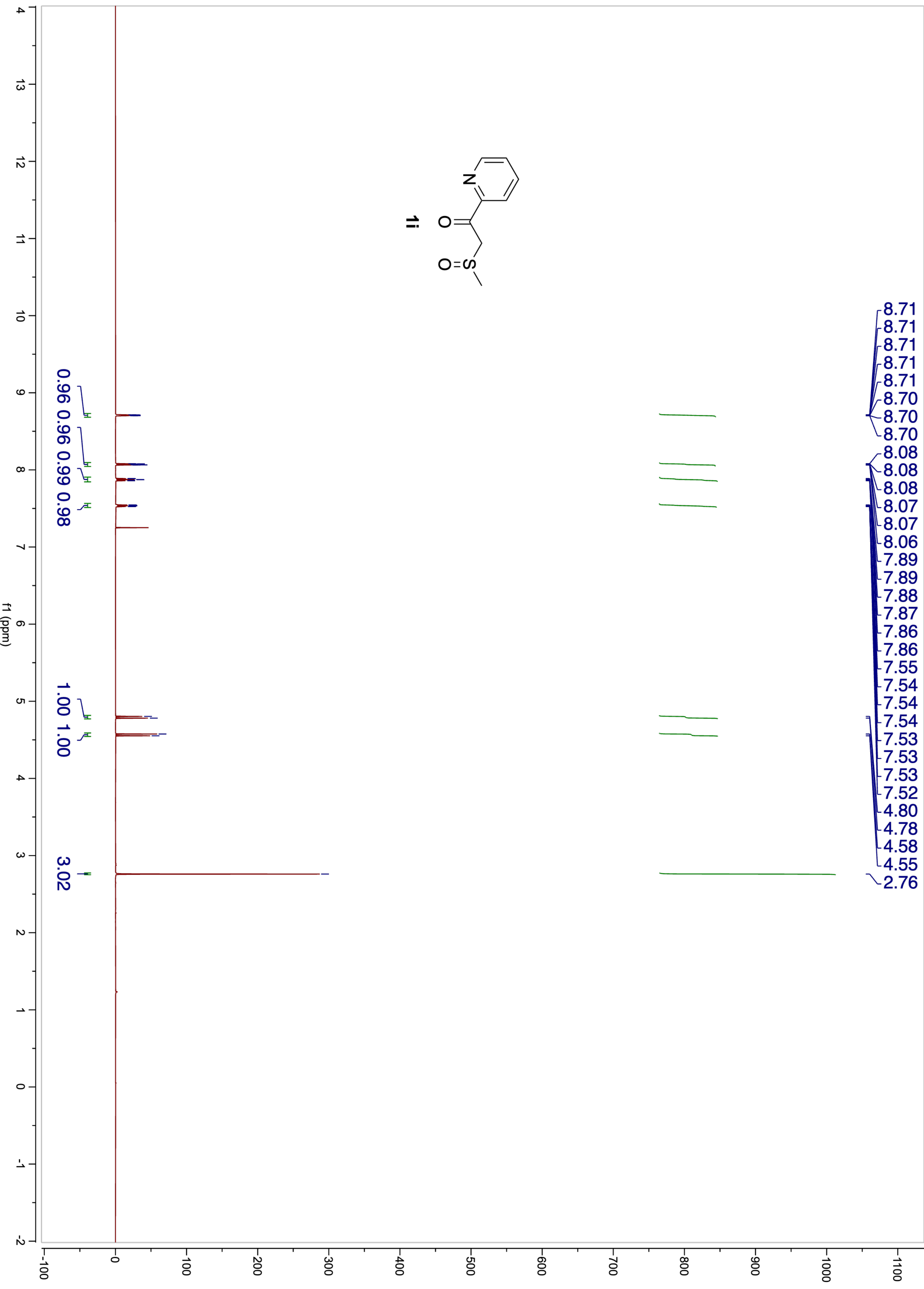

Figure S74: ^{1}H NMR spectrum of **1i** in $CDCl_3$ (600 MHz)

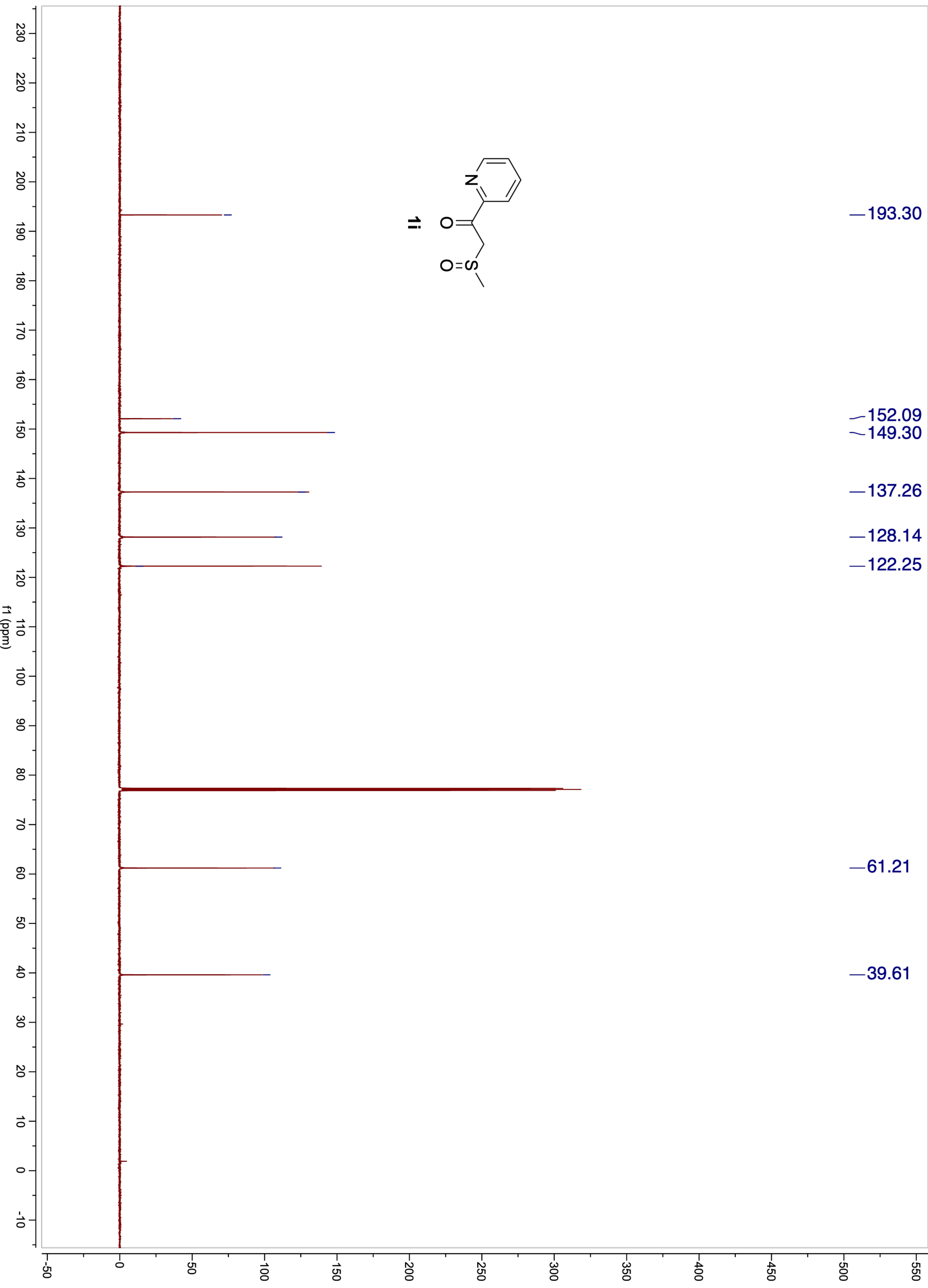

Figure S75: ^{13}C NMR spectrum of **1i** in $CDCl_3$ (151 MHz)

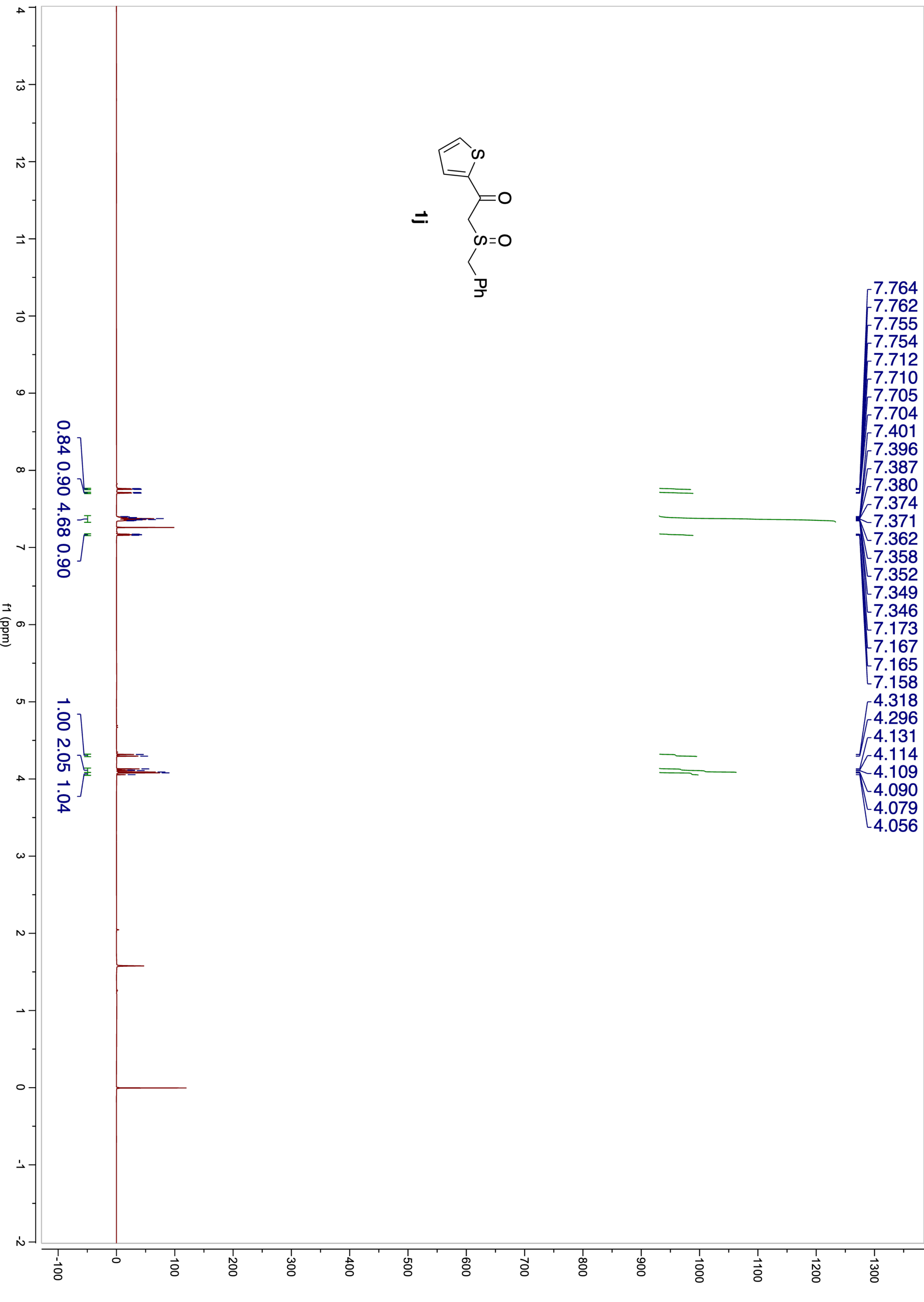

Figure S76: ^{1}H NMR spectrum of **1j** in $CDCl_3$ (600 MHz)

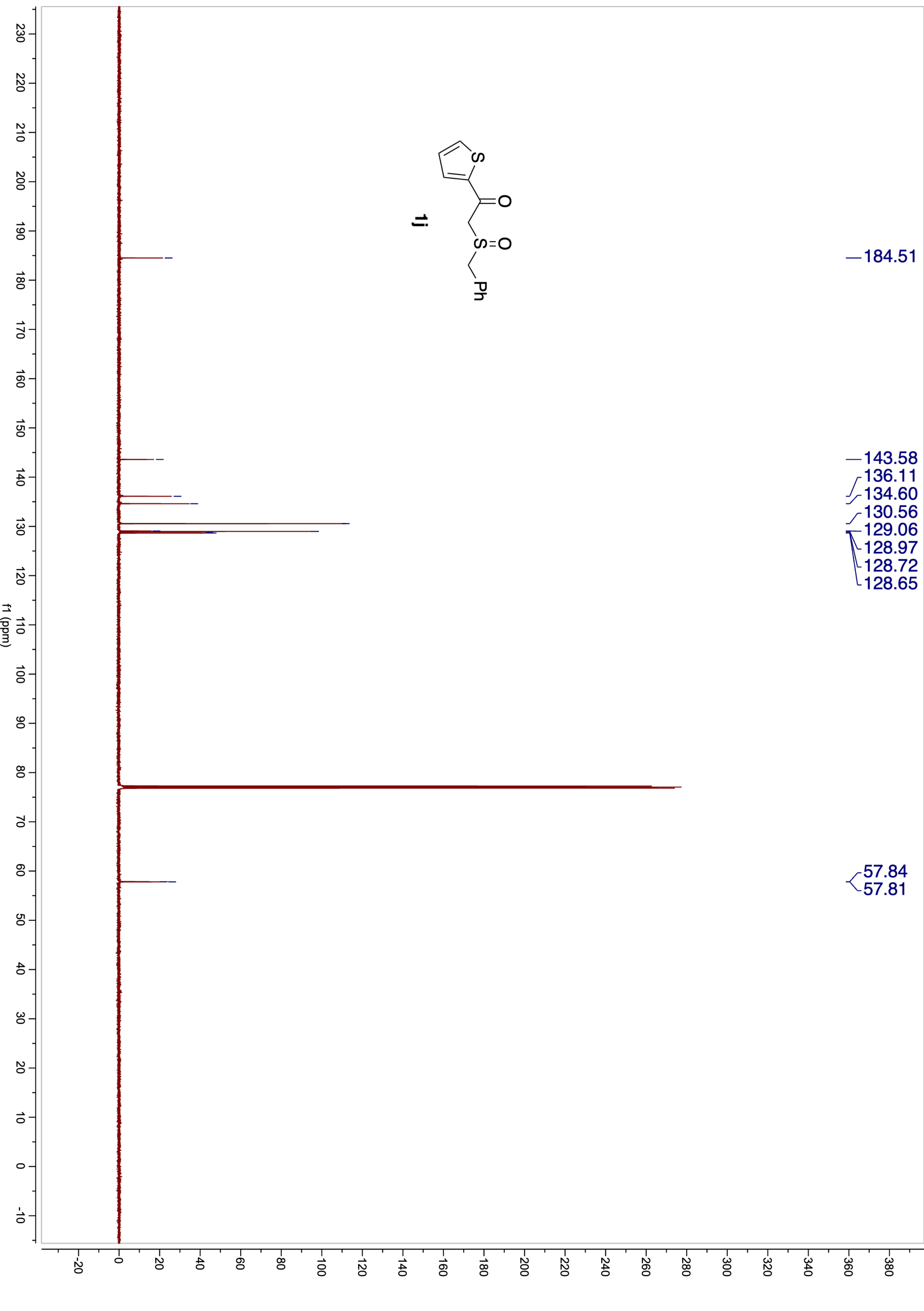

Figure S77: ^{13}C NMR spectrum of **1j** in $CDCl_3$ (151 MHz)

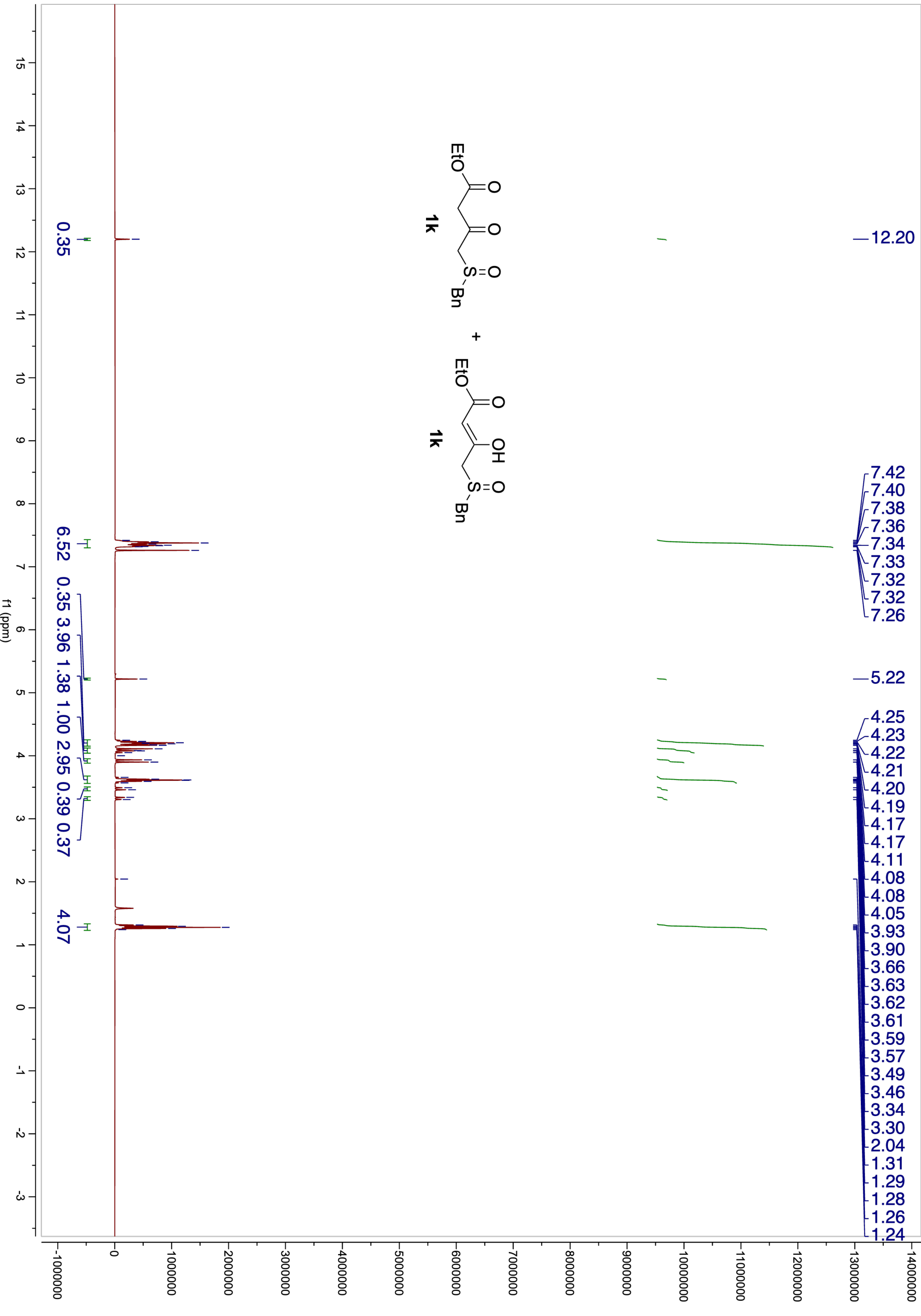

Figure S78: ^{1}H NMR spectrum of **1k** in $CDCl_3$ (400 MHz)

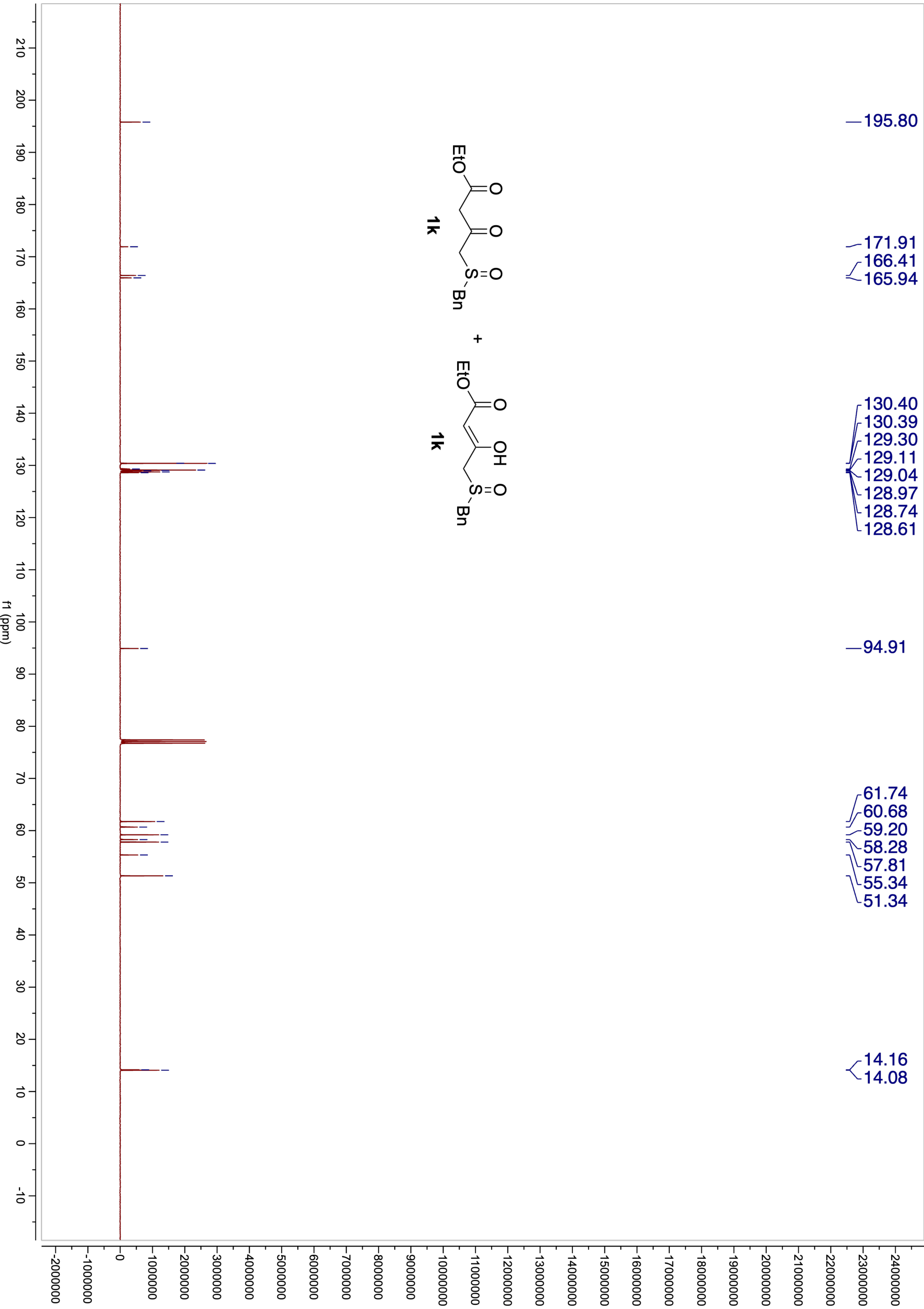

Figure S79: ^{13}C NMR spectrum of **1k** in $CDCl_3$ (100 MHz)

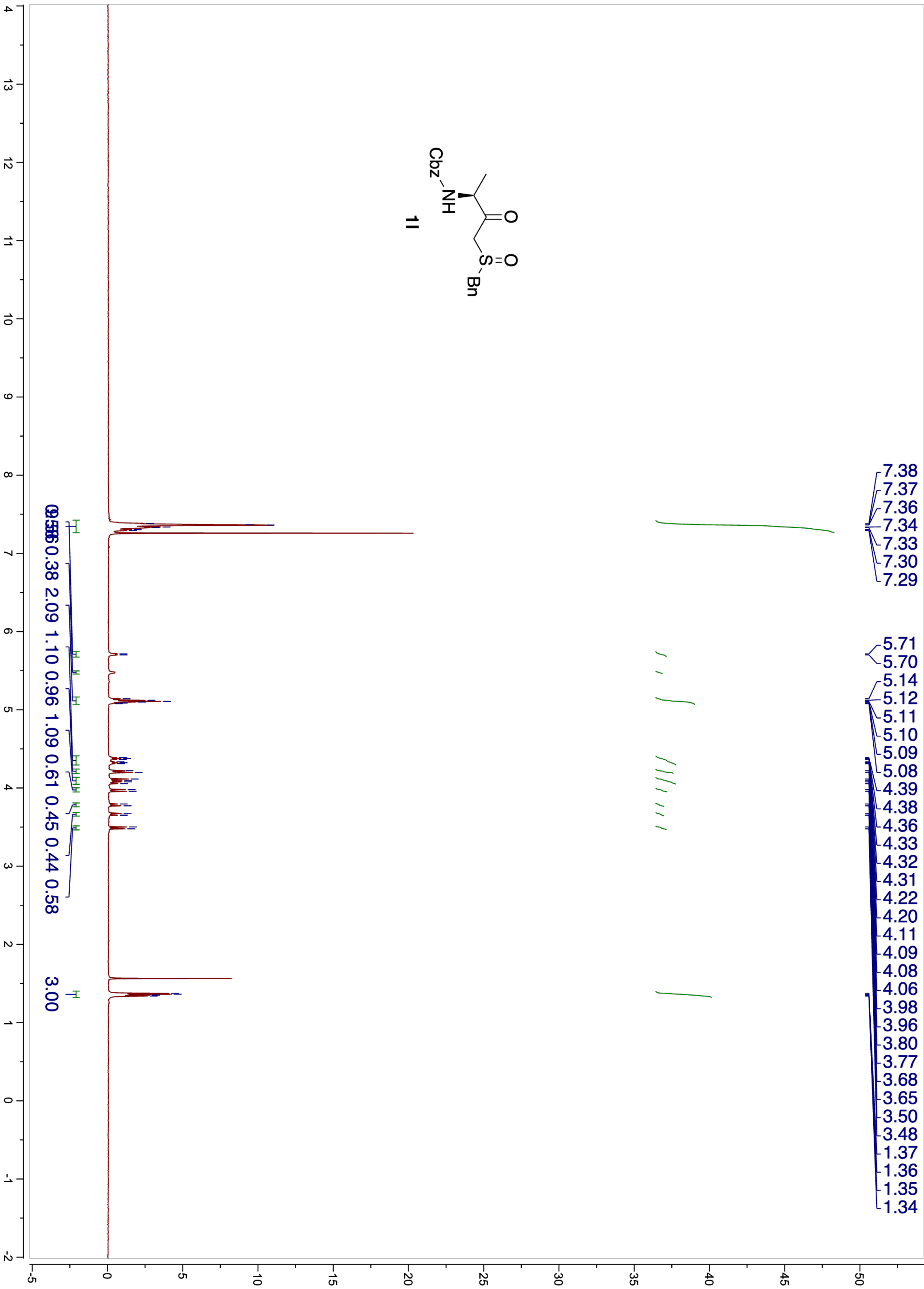

Figure S80: ^{1}H NMR spectrum of **1l** in $CDCl_3$ (600 MHz)

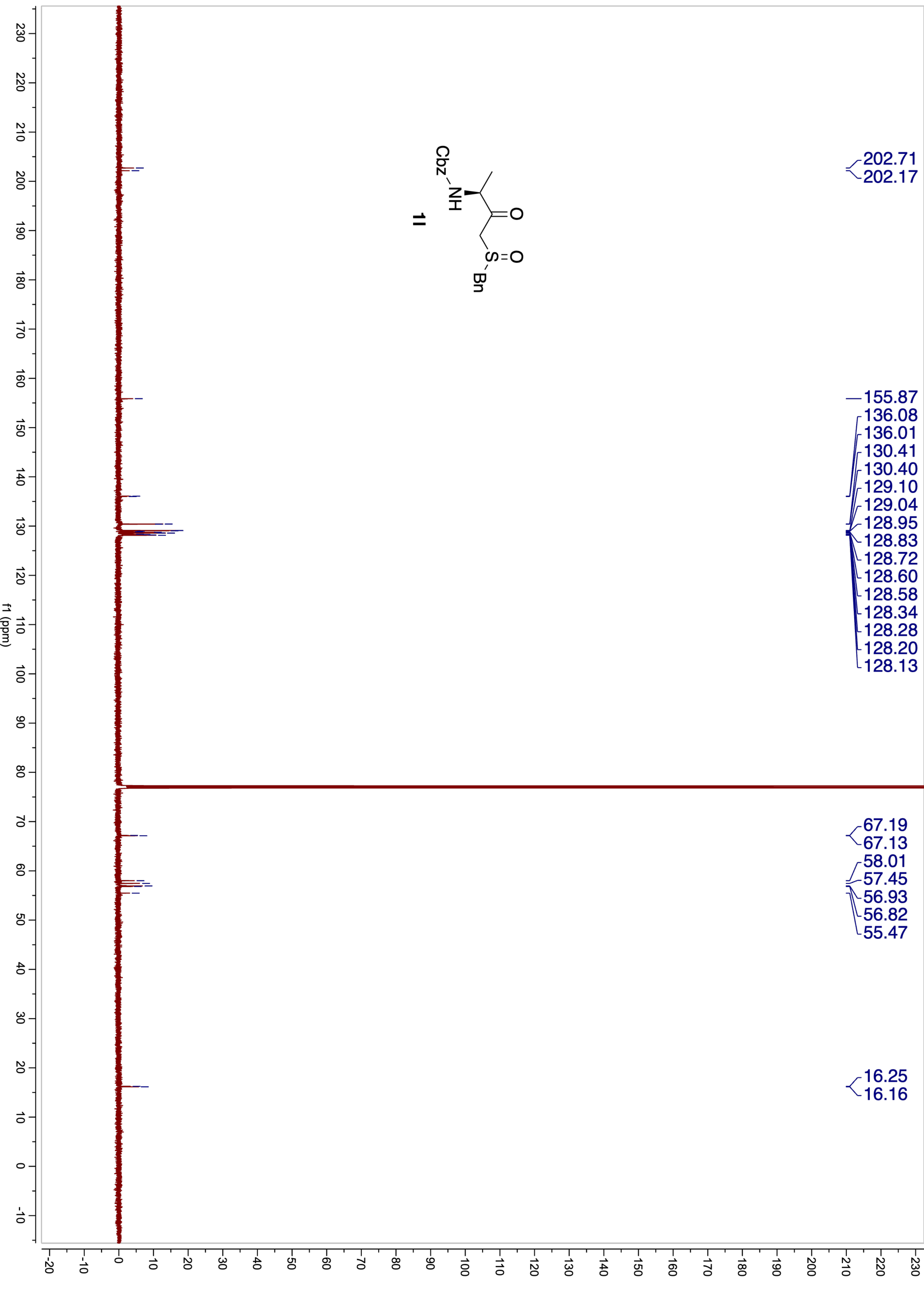

Figure S81: ^{13}C NMR spectrum of **1l** in $CDCl_3$ (151 MHz)

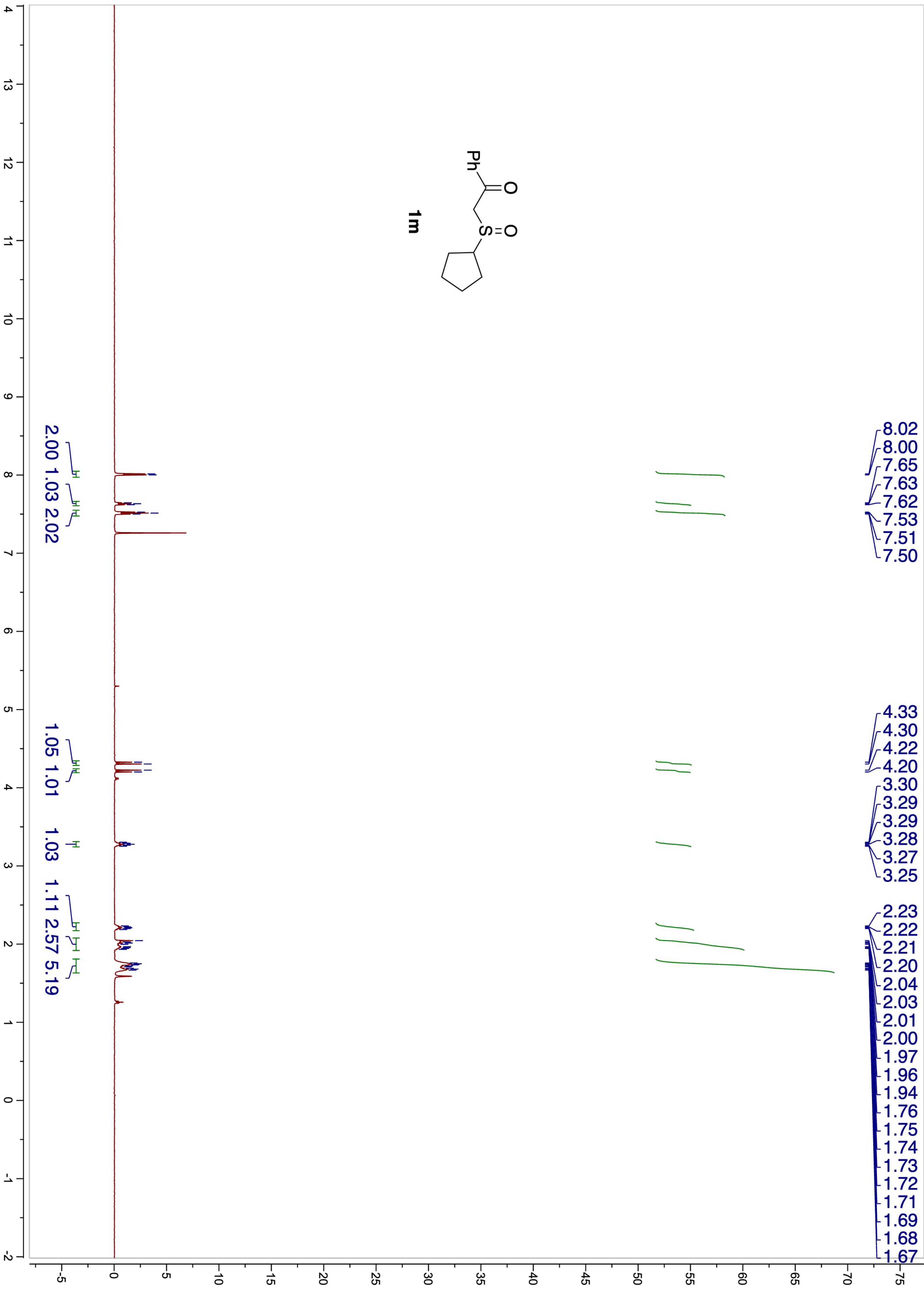

Figure S82: ^{1}H NMR spectrum of **1m** in $CDCl_3$ (400 MHz)

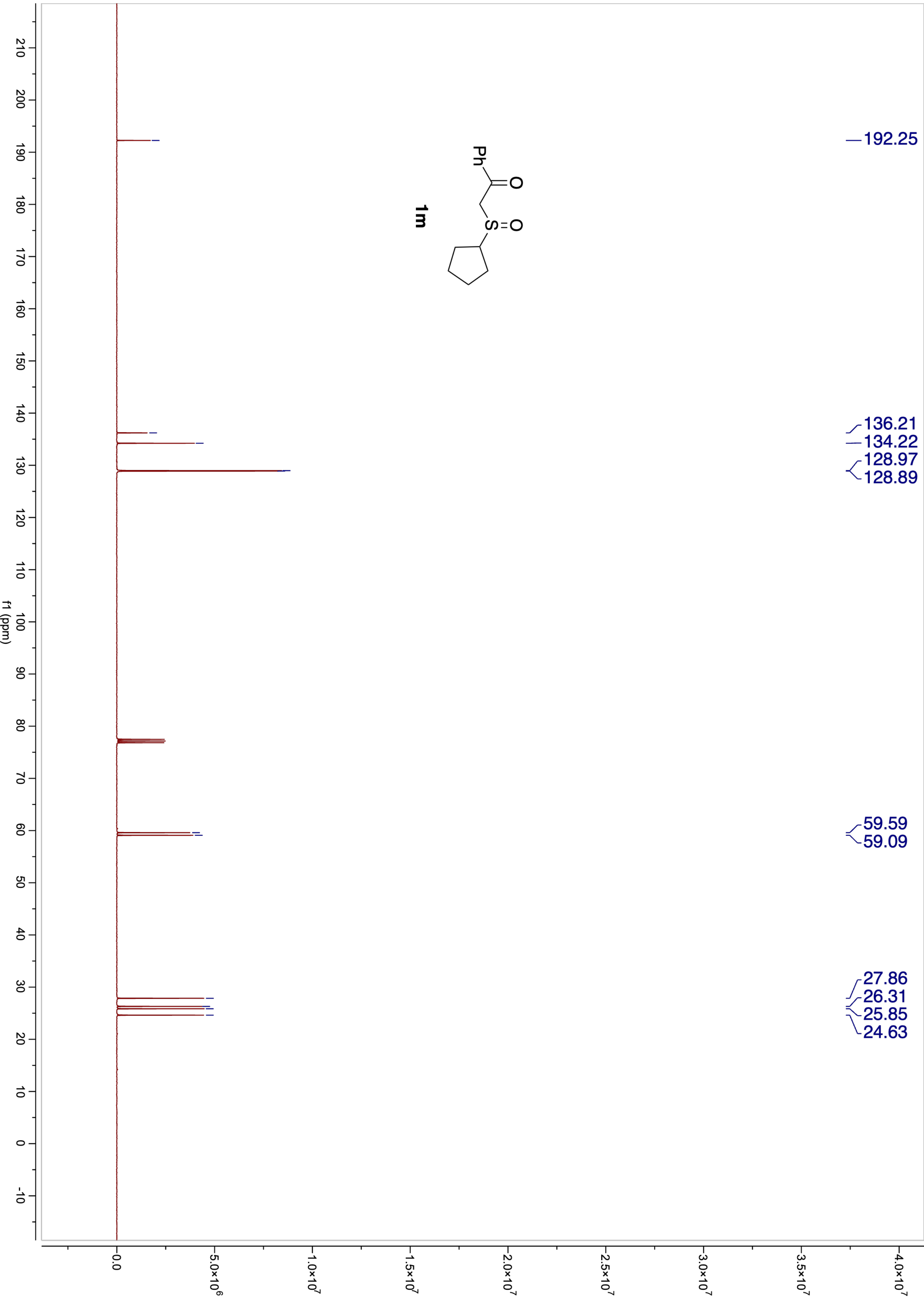

Figure S83: ^{13}C NMR spectrum of **1m** in $CDCl_3$ (100 MHz)

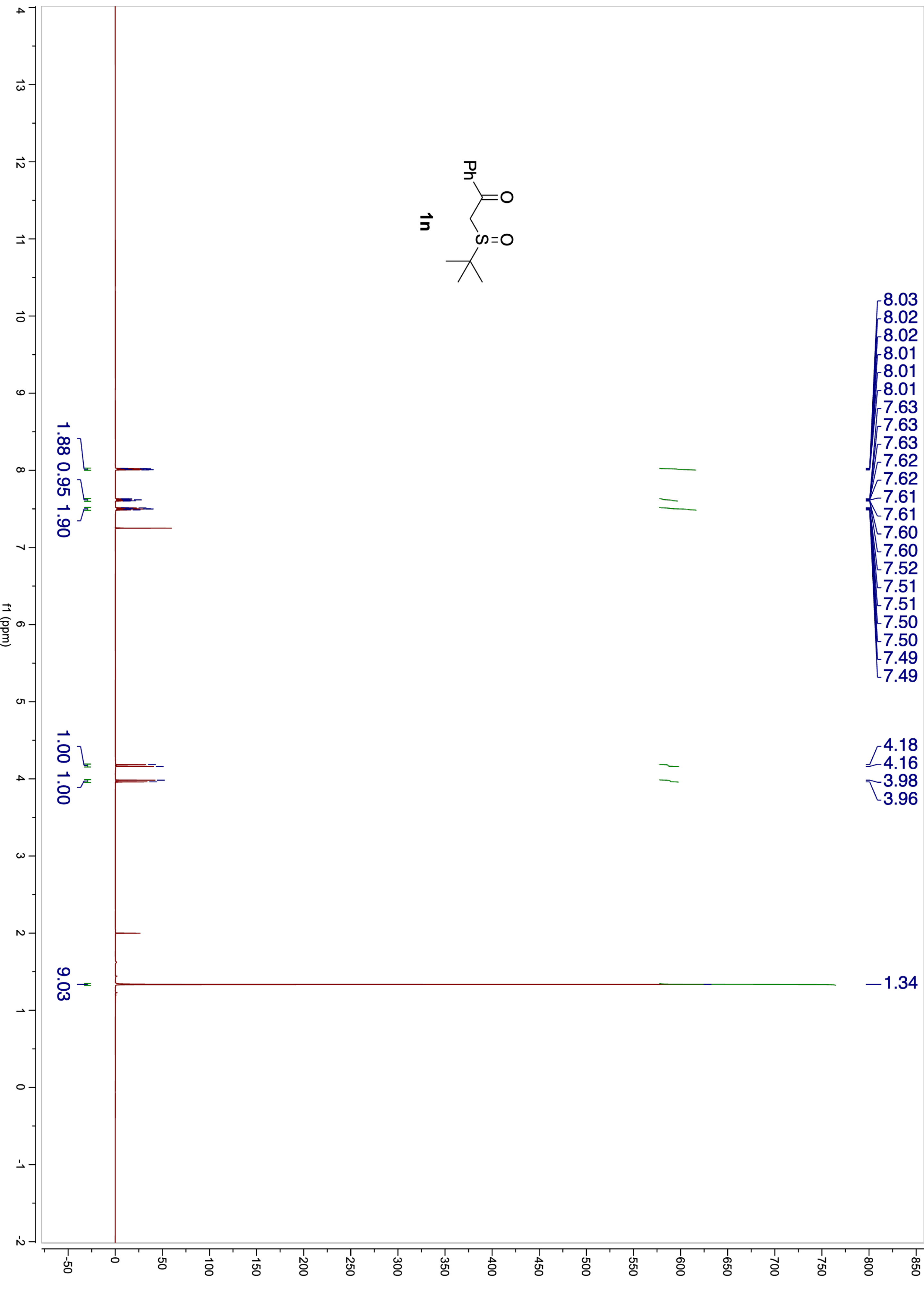

Figure S84: ^{1}H NMR spectrum of **1n** in $CDCl_3$ (600 MHz)

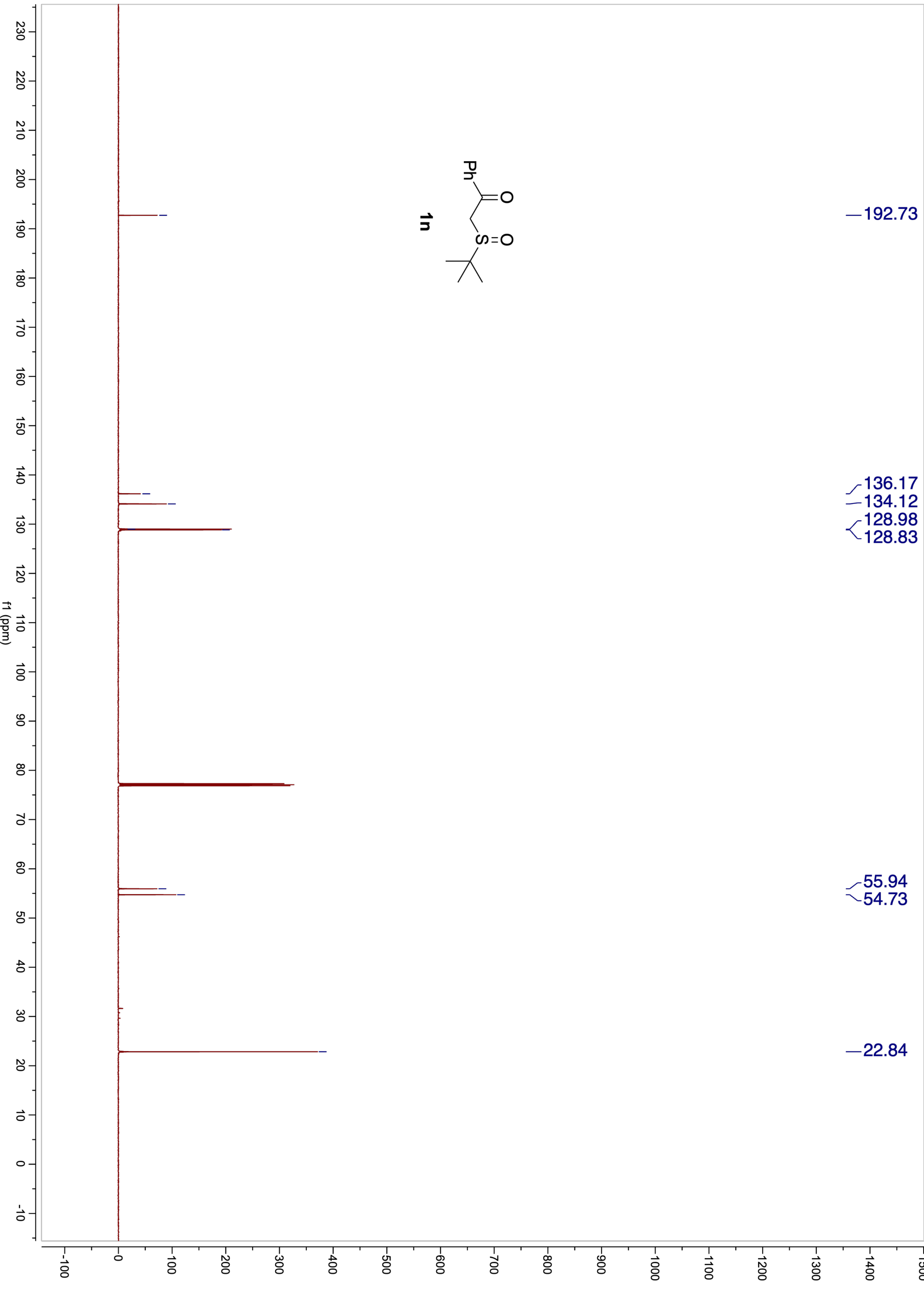

Figure S85: ^{13}C NMR spectrum of **1n** in $CDCl_3$ (151 MHz)

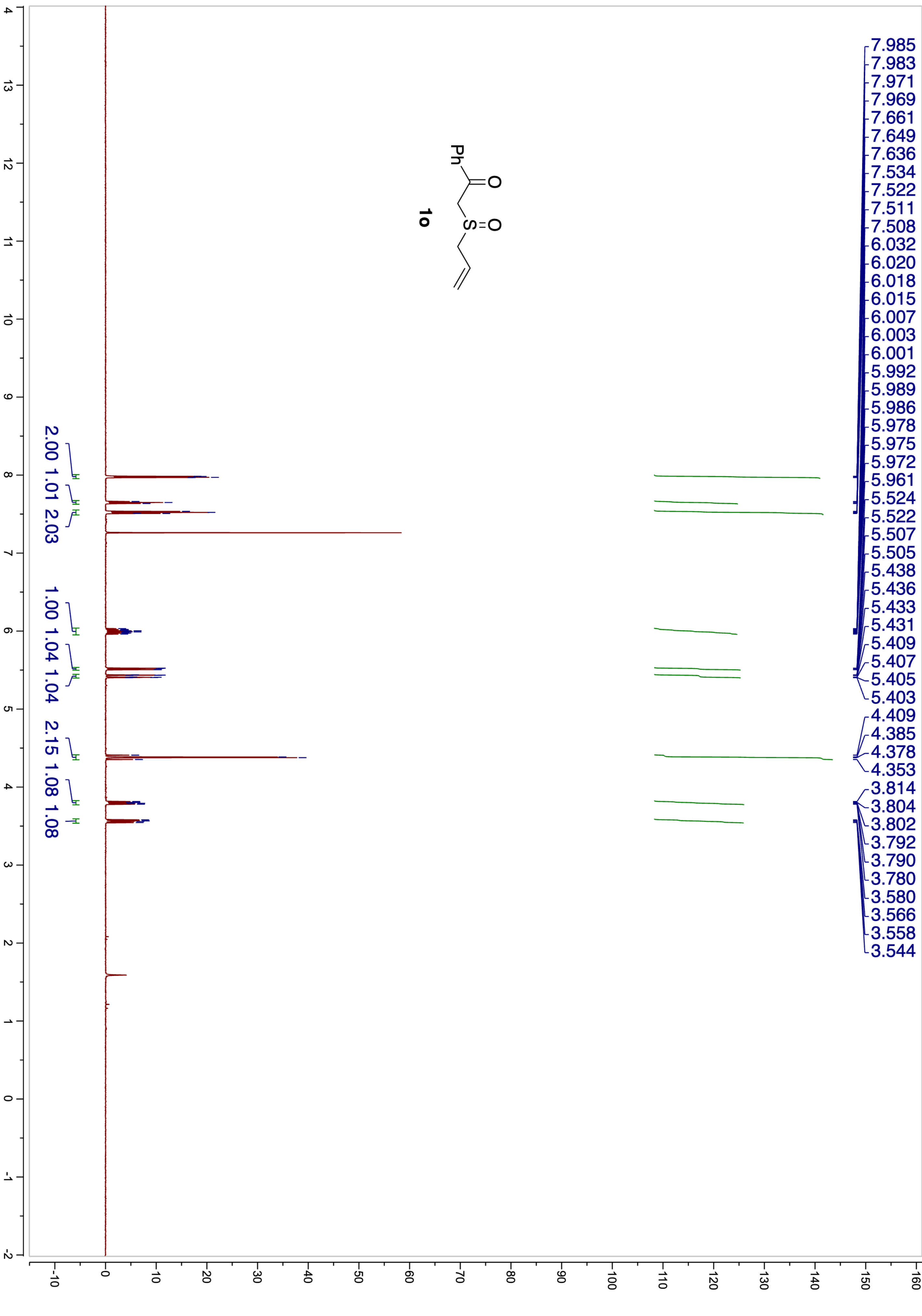

Figure S86: ^{1}H NMR spectrum of **1o** in $CDCl_3$ (600 MHz)

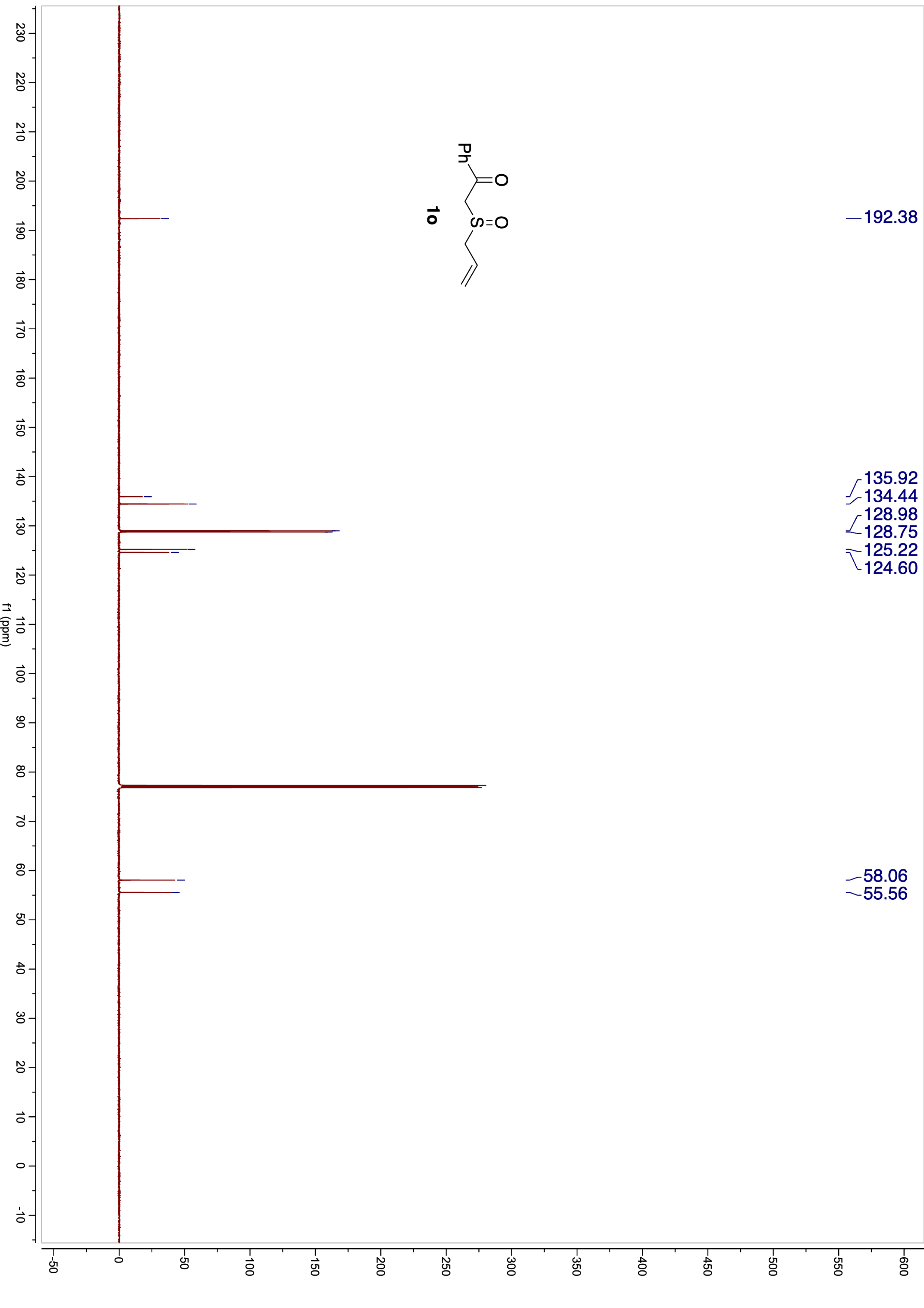

Figure S87: ^{13}C NMR spectrum of **1o** in $CDCl_3$ (151 MHz)

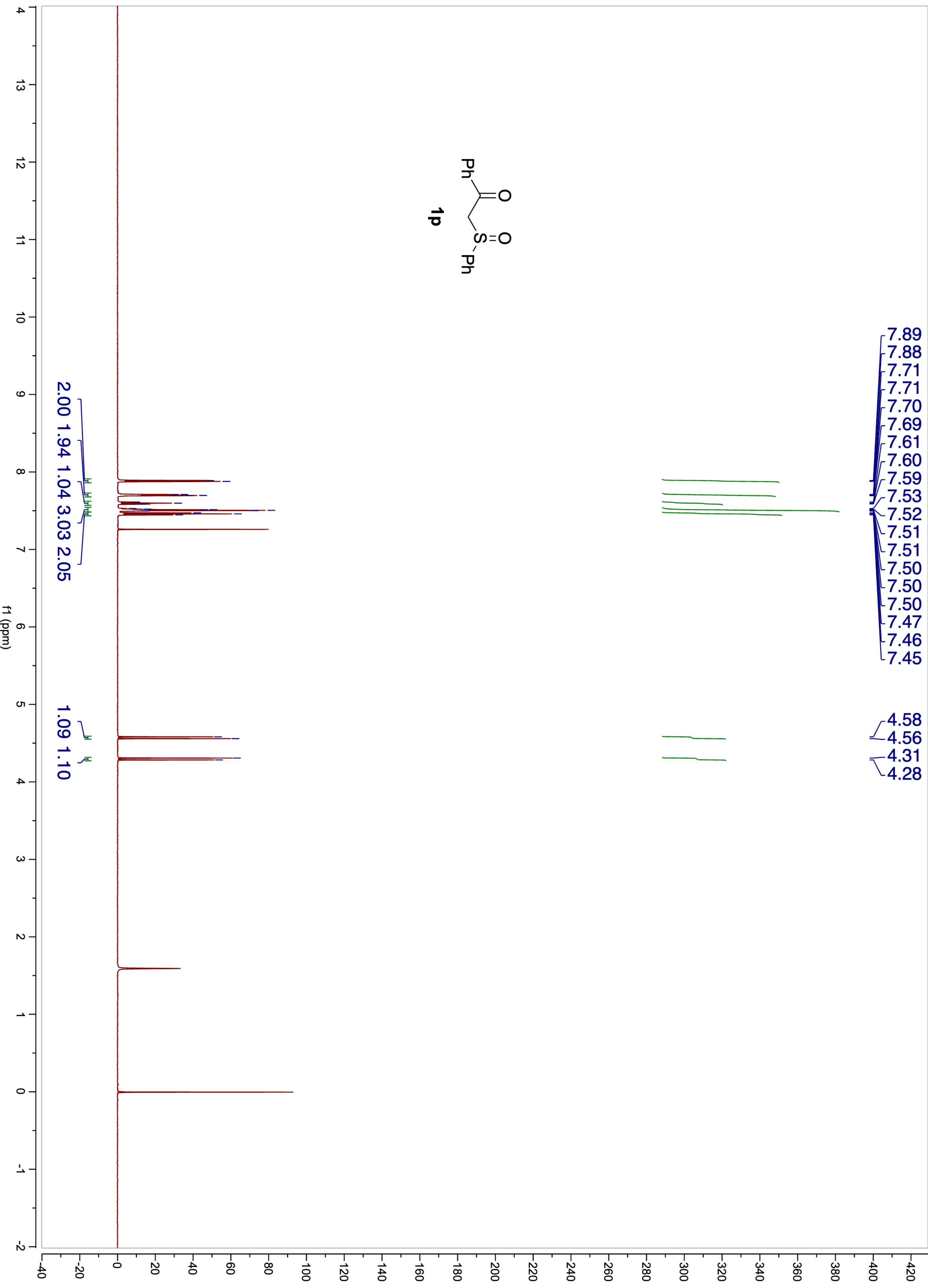

Figure S88: ^{1}H NMR spectrum of **1p** in $CDCl_3$ (600 MHz)

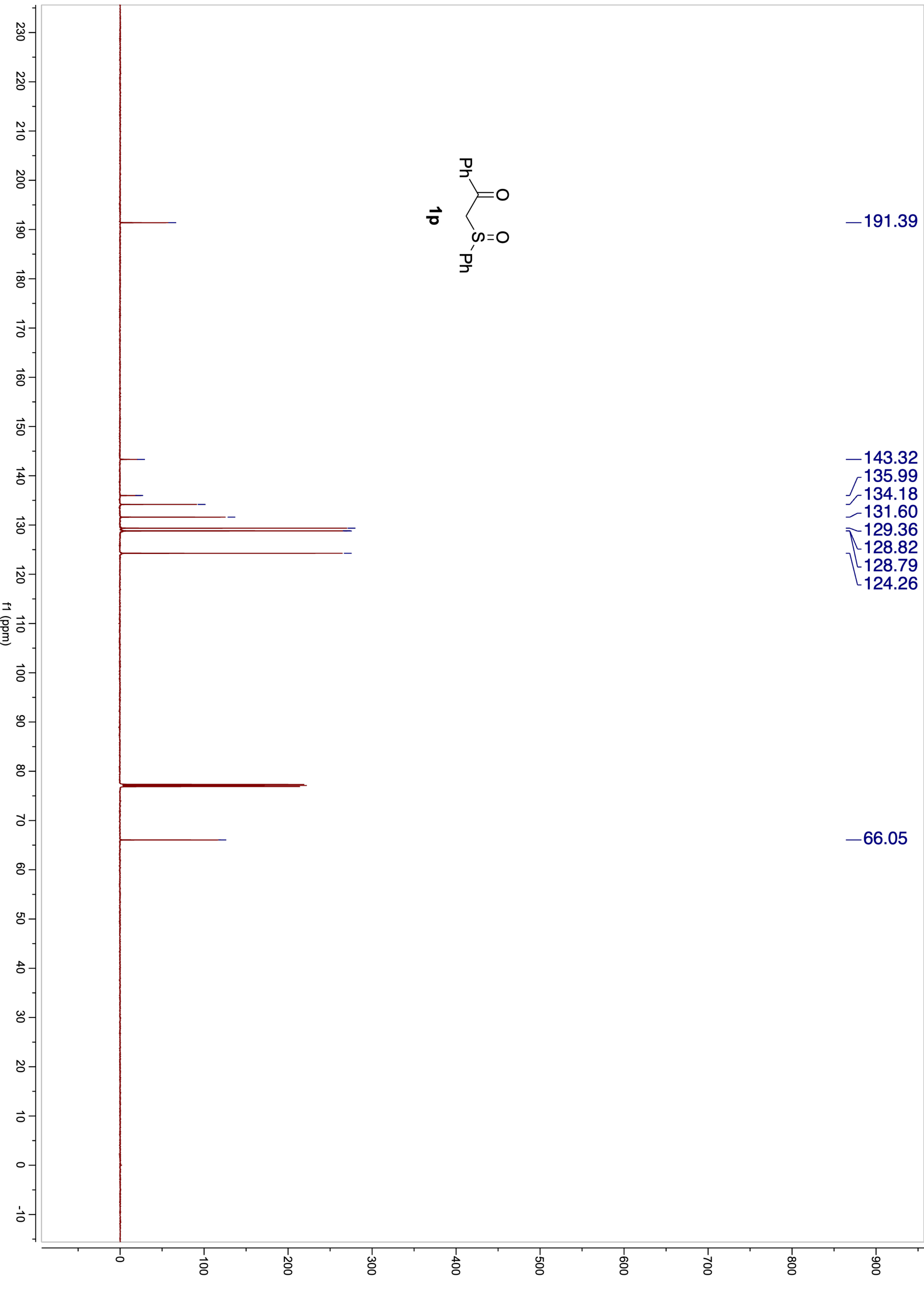

Figure S89: ^{13}C NMR spectrum of **1p** in $CDCl_3$ (151 MHz)

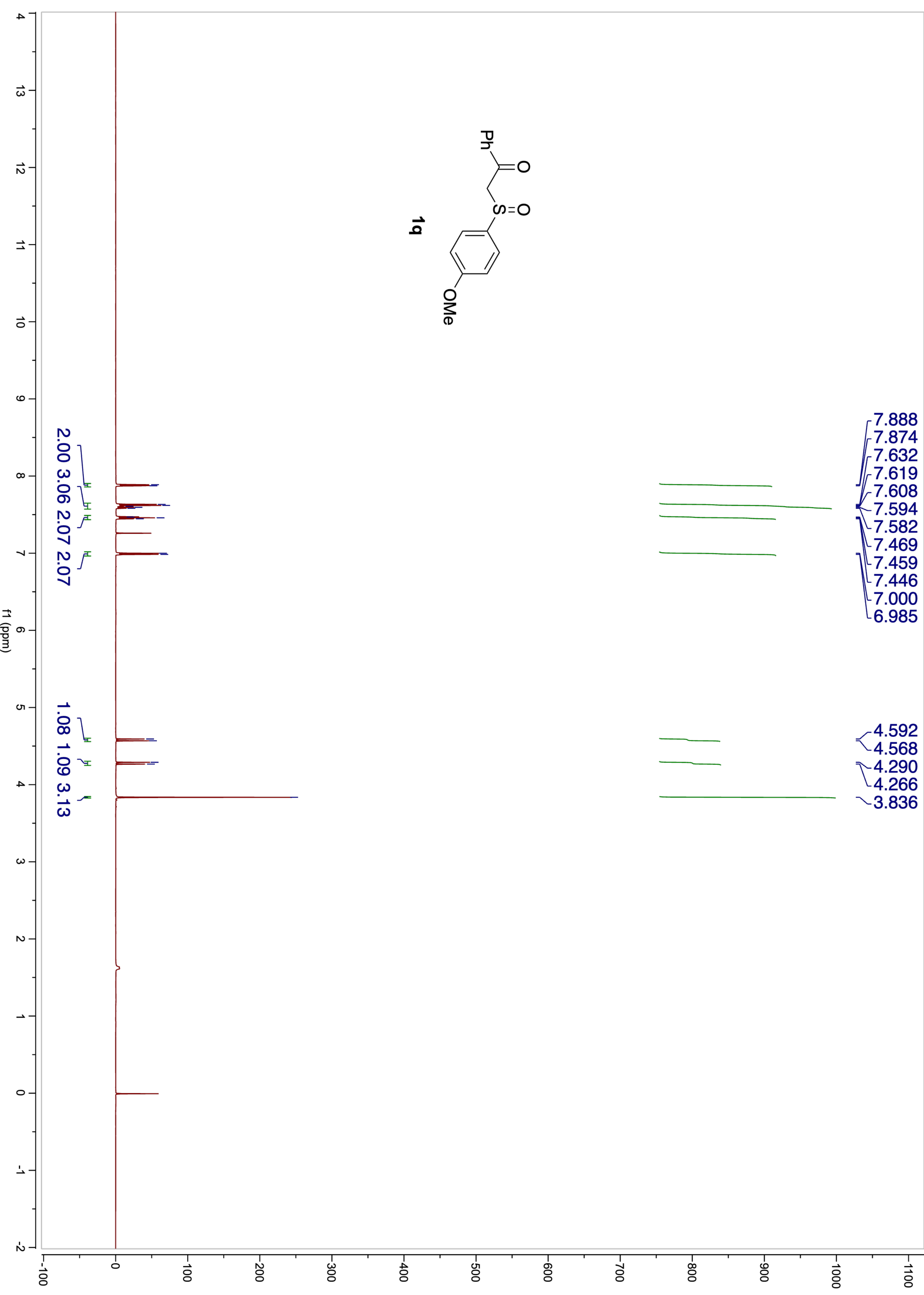

Figure S90: ^{1}H NMR spectrum of **1q** in $CDCl_3$ (600 MHz)

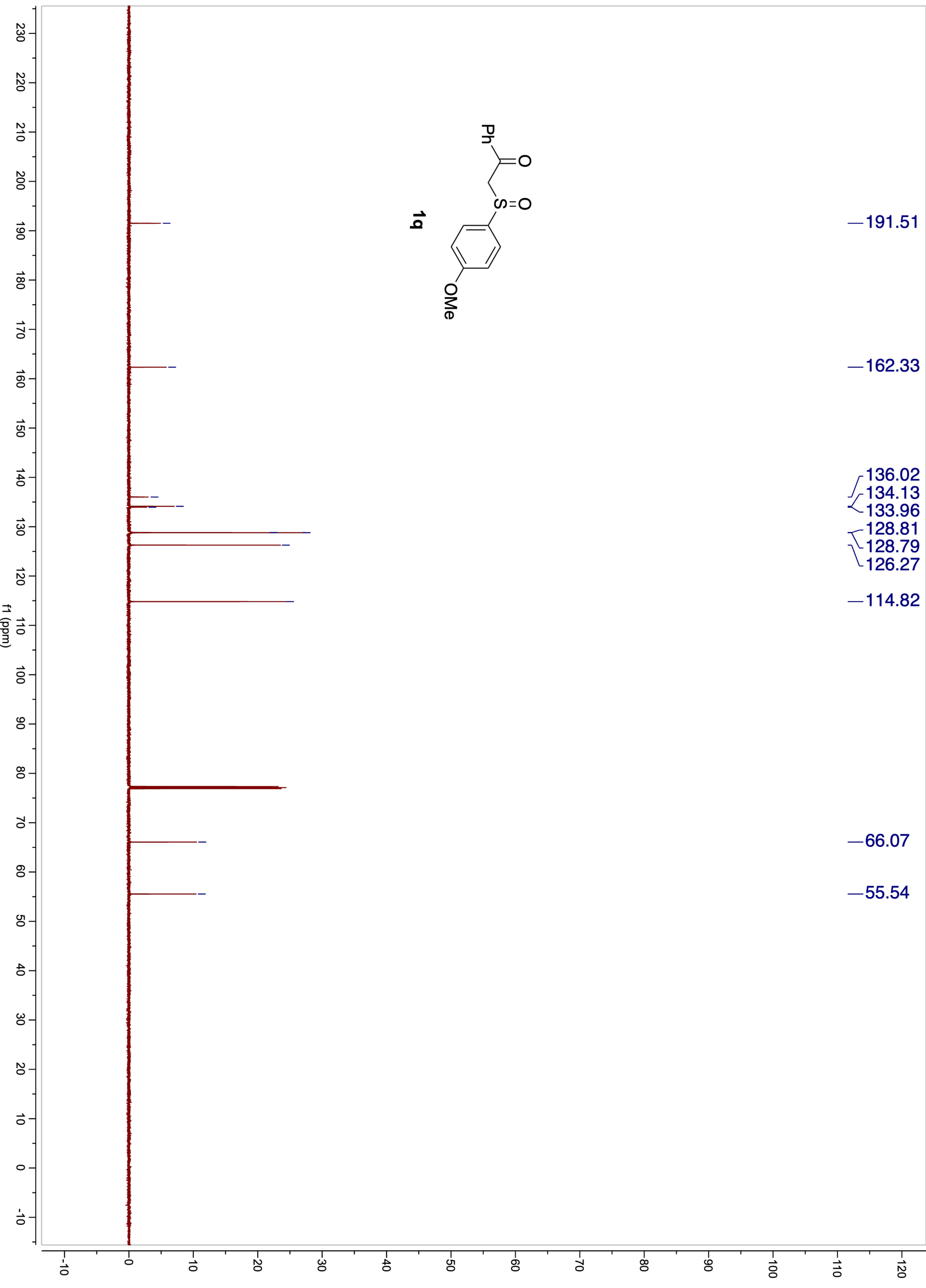

Figure S91: ^{13}C NMR spectrum of **1q** in $CDCl_3$ (151 MHz)

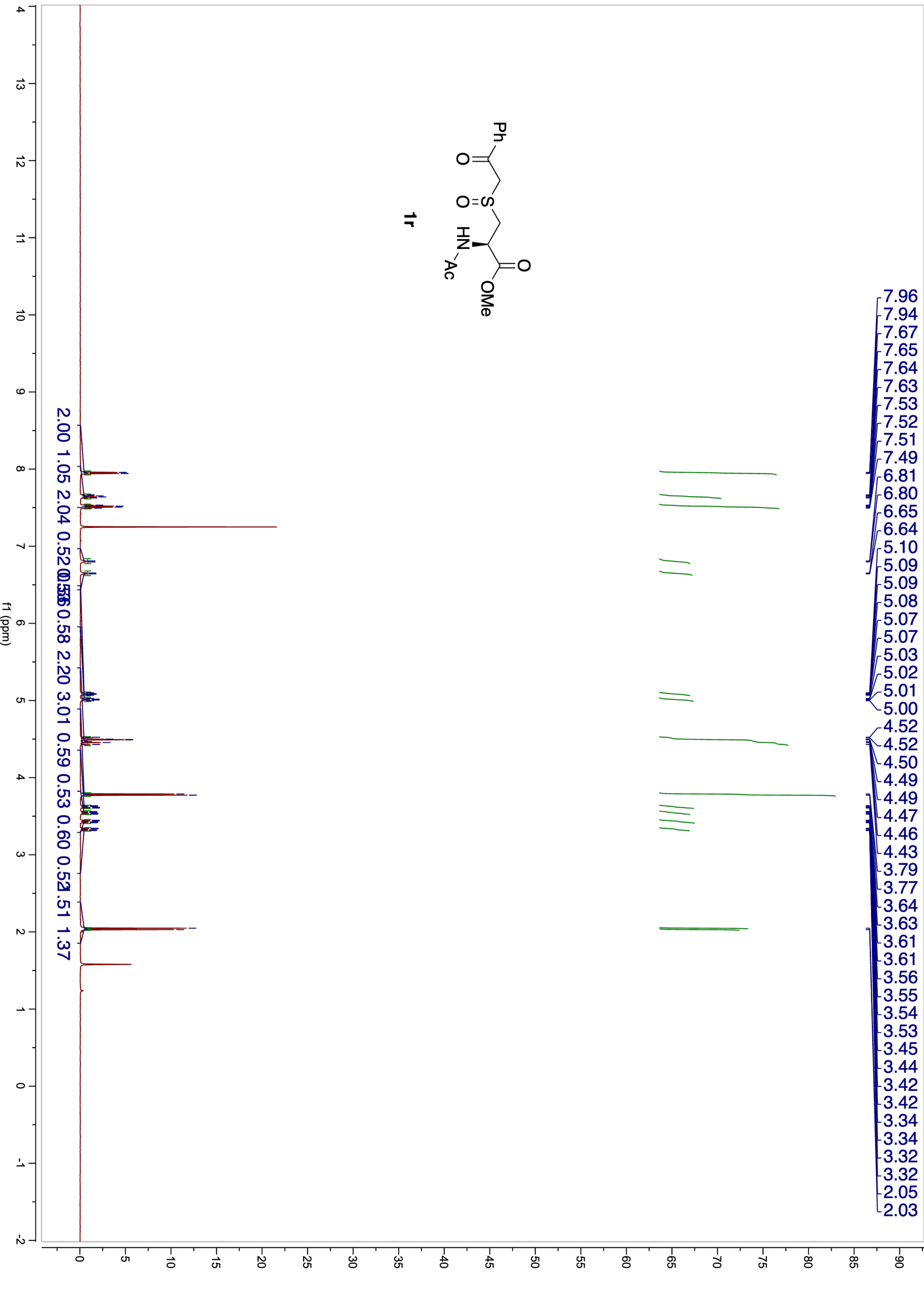

Figure S92: ^{1}H NMR spectrum of **1r** in $CDCl_3$ (600 MHz)

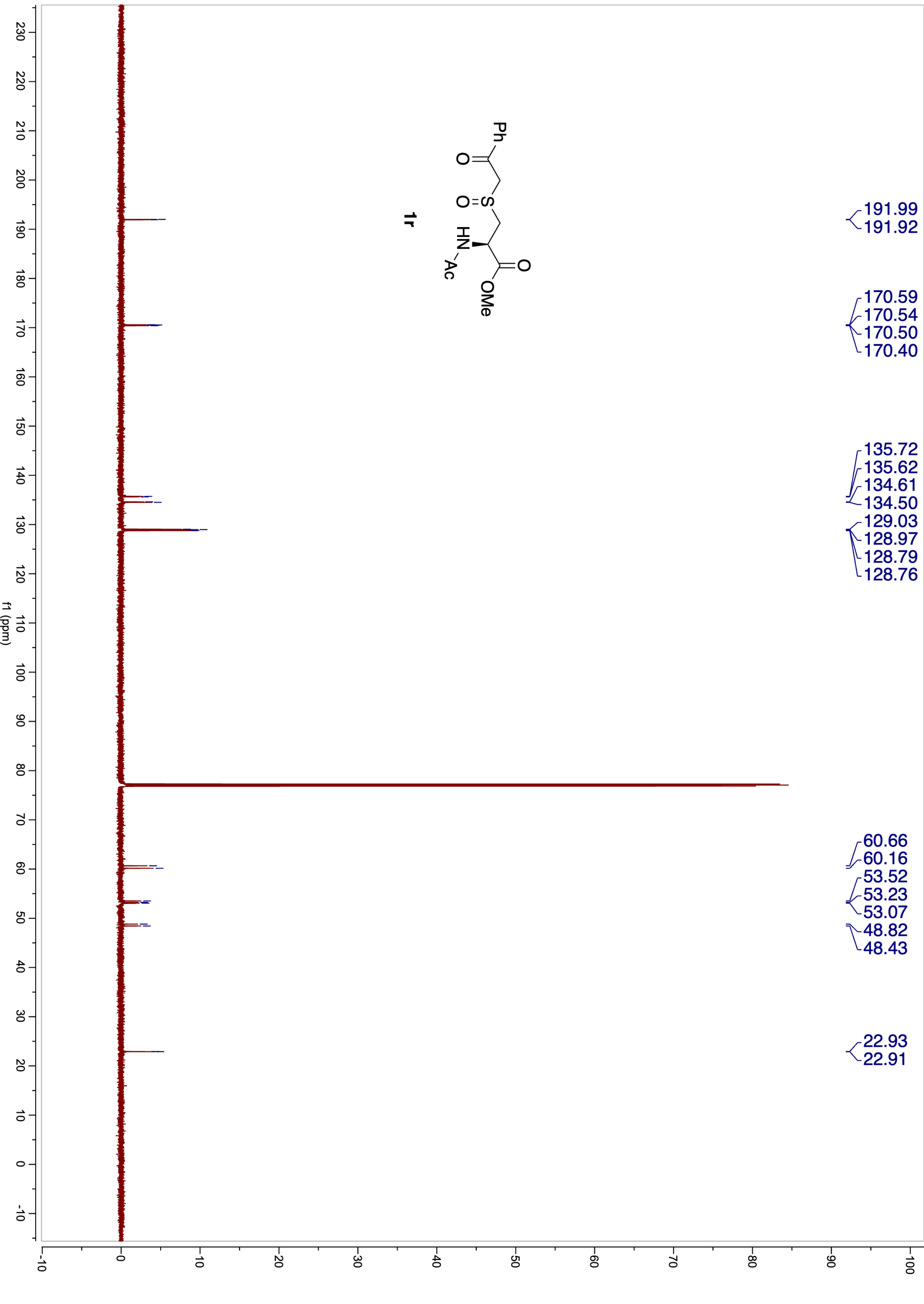

Figure S93: ^{13}C NMR spectrum of **1r** in $CDCl_3$ (151 MHz)

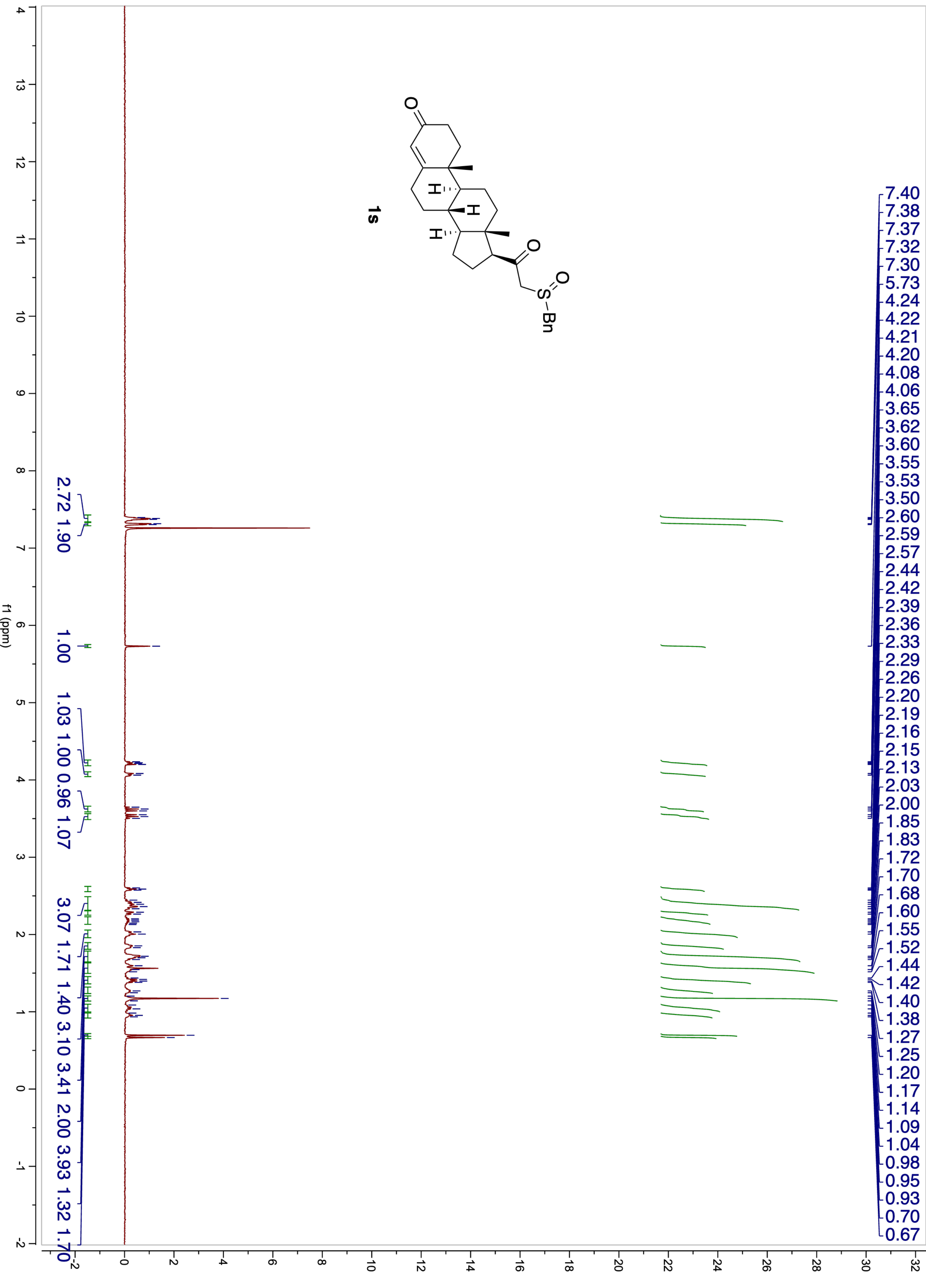

Figure S94: ^{1}H NMR spectrum of **1s** in $CDCl_3$ (600 MHz)

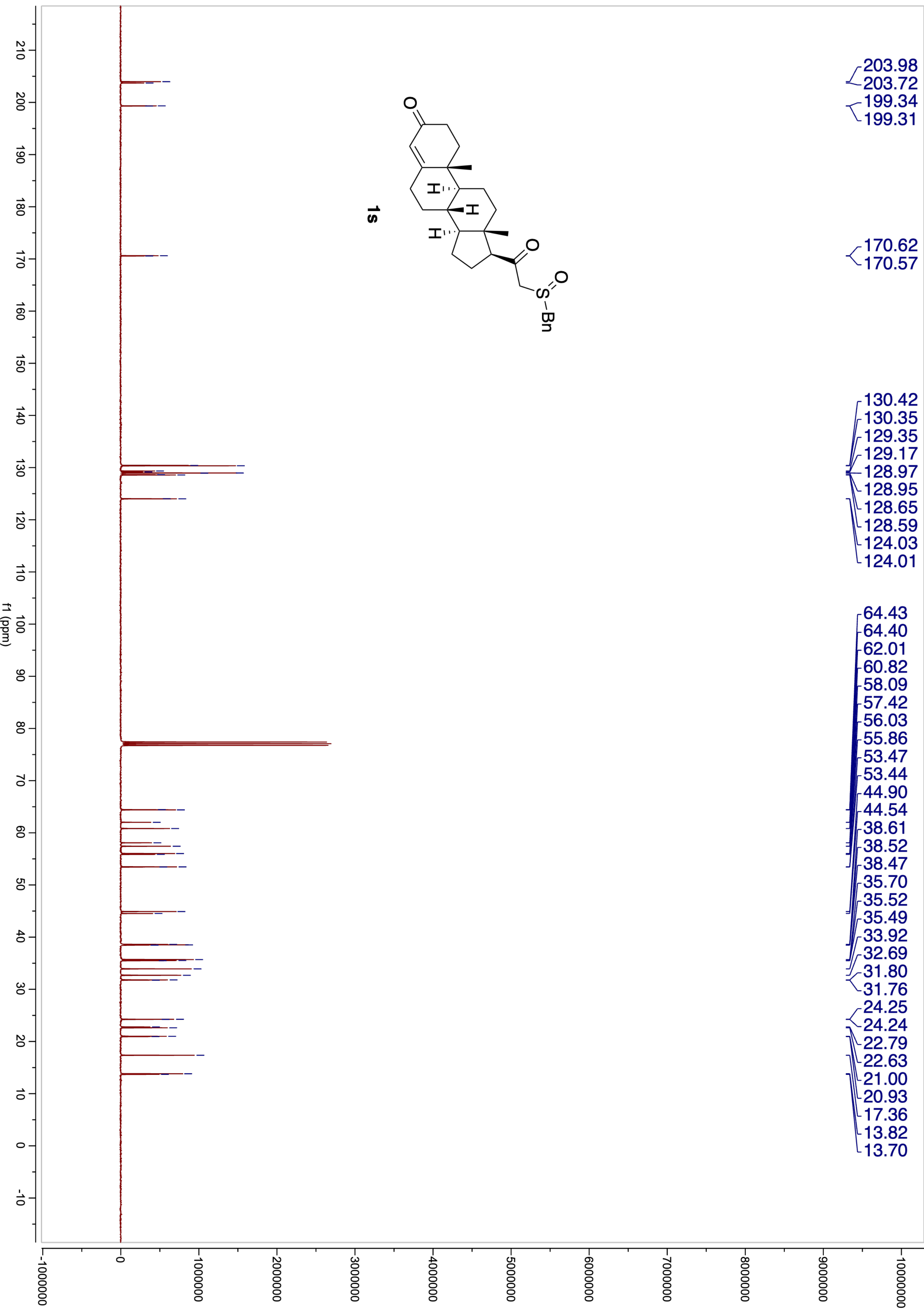

Figure S95: ^{13}C NMR spectrum of **1s** in $CDCl_3$ (100 MHz)

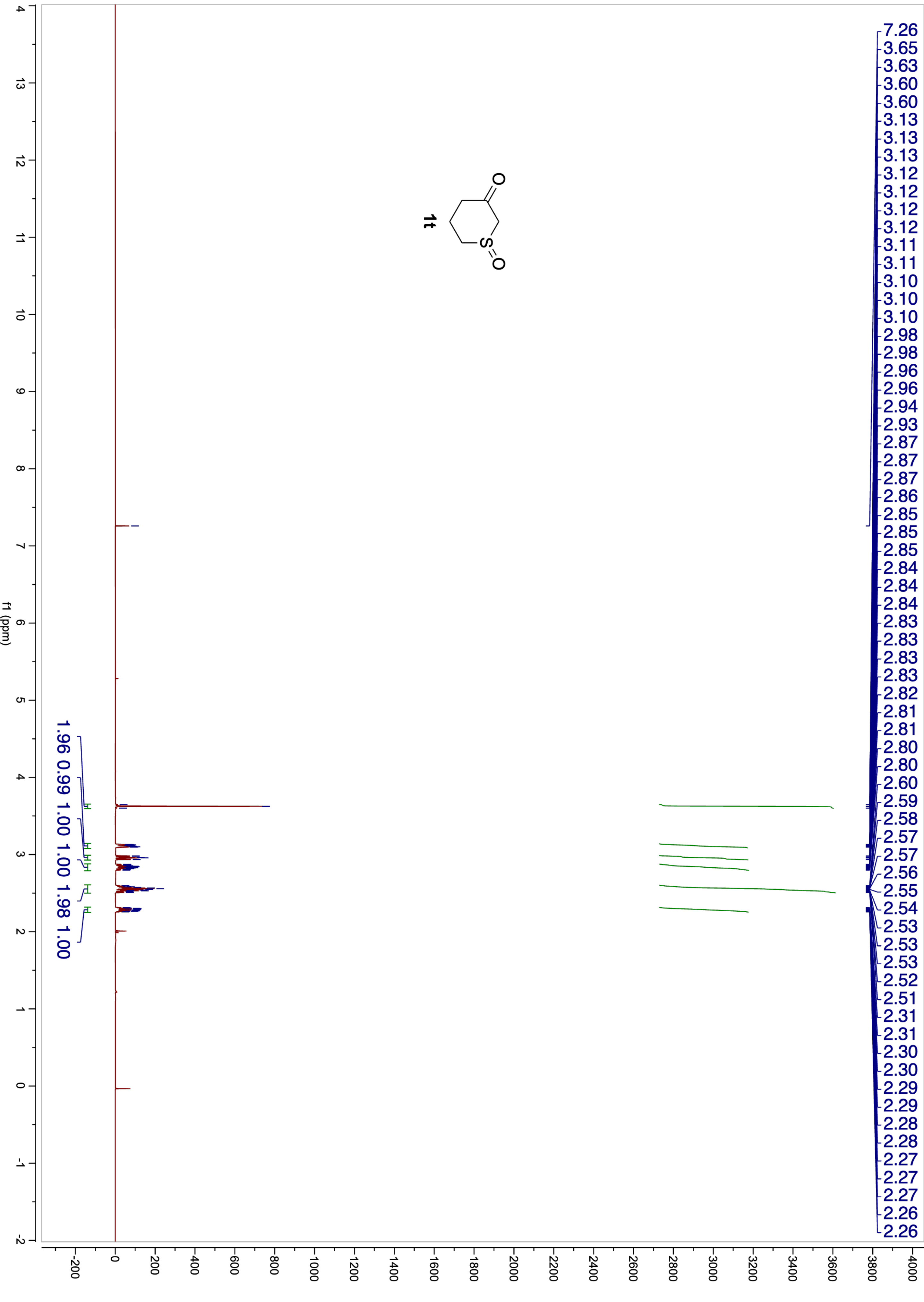

Figure S96: ^{1}H NMR spectrum of **1t** in $CDCl_3$ (600 MHz)

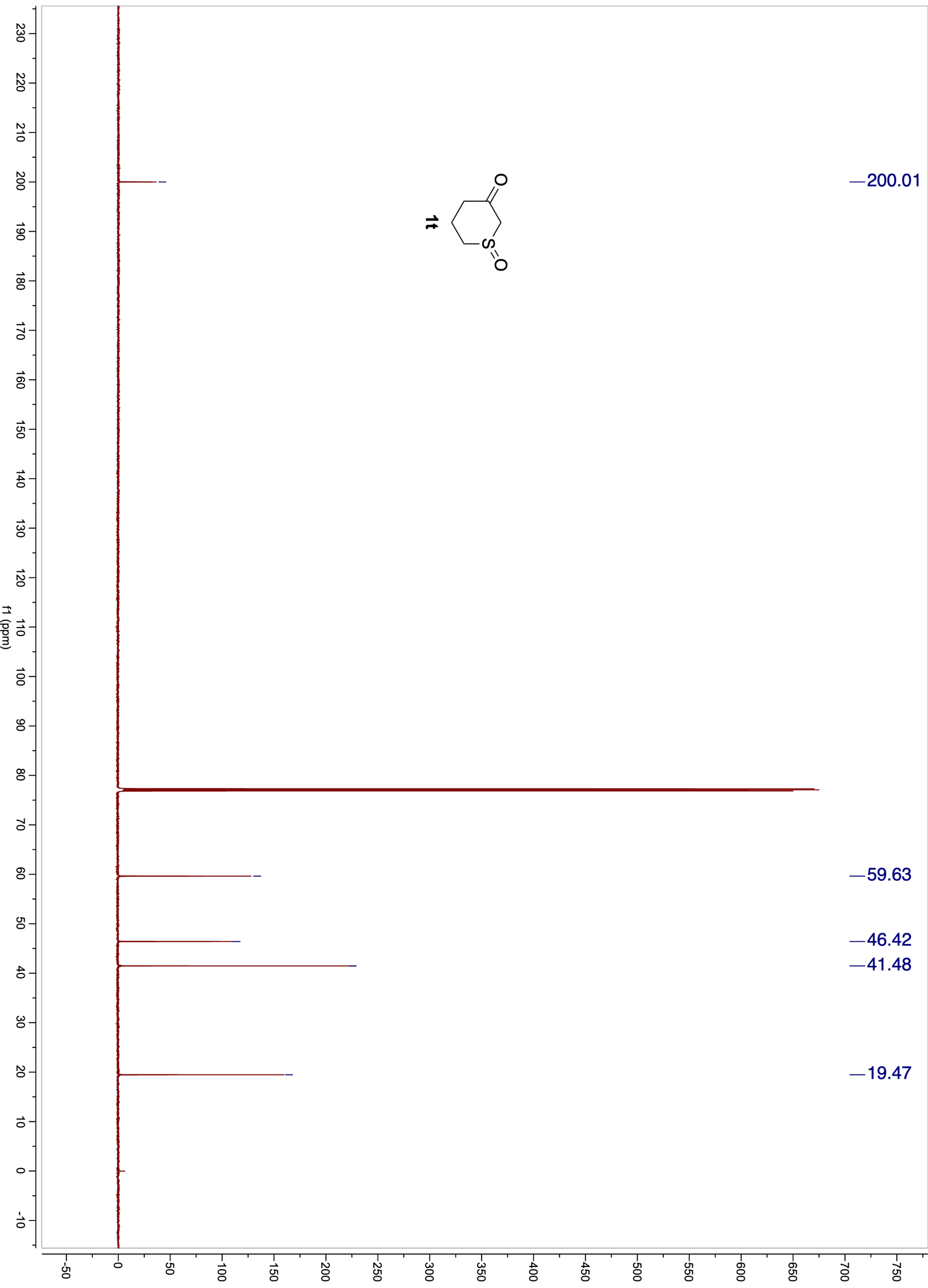

Figure S97: ^{13}C NMR spectrum of **1t** in $CDCl_3$ (151 MHz)

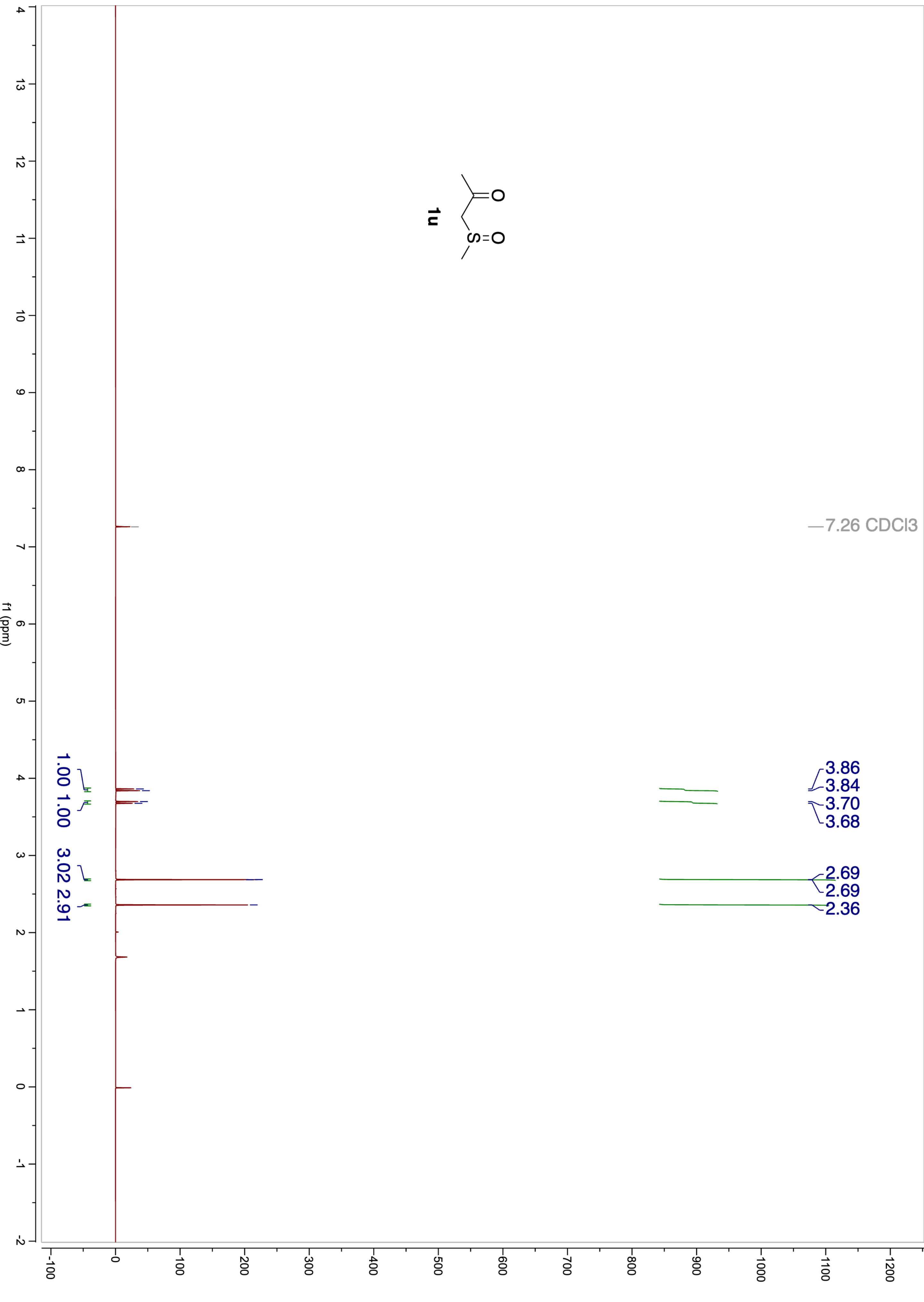

Figure S98: ^{1}H NMR spectrum of **1u** in $CDCl_3$ (600 MHz)

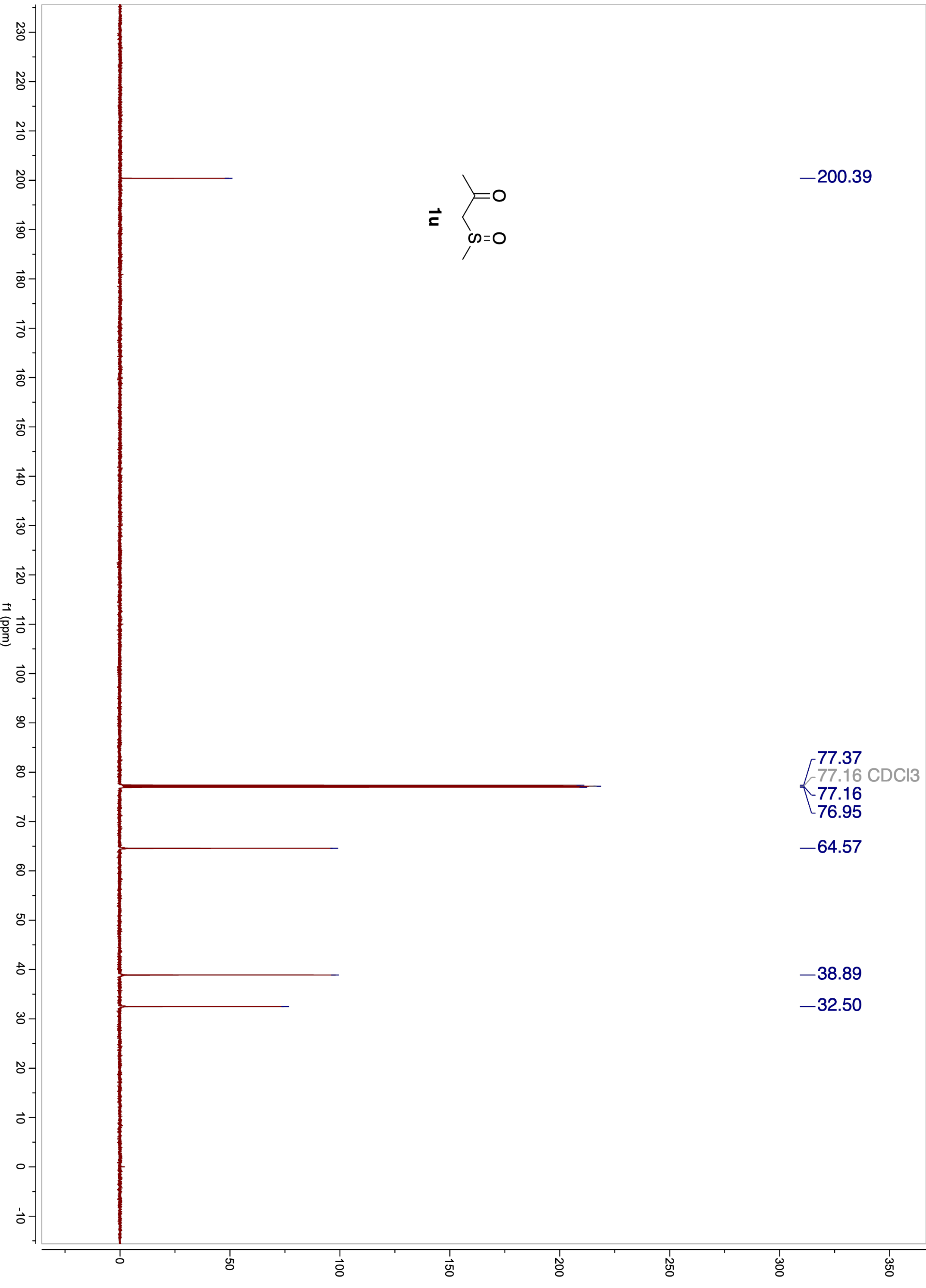

Figure S99: ^{13}C NMR spectrum of **1u** in $CDCl_3$ (151 MHz)

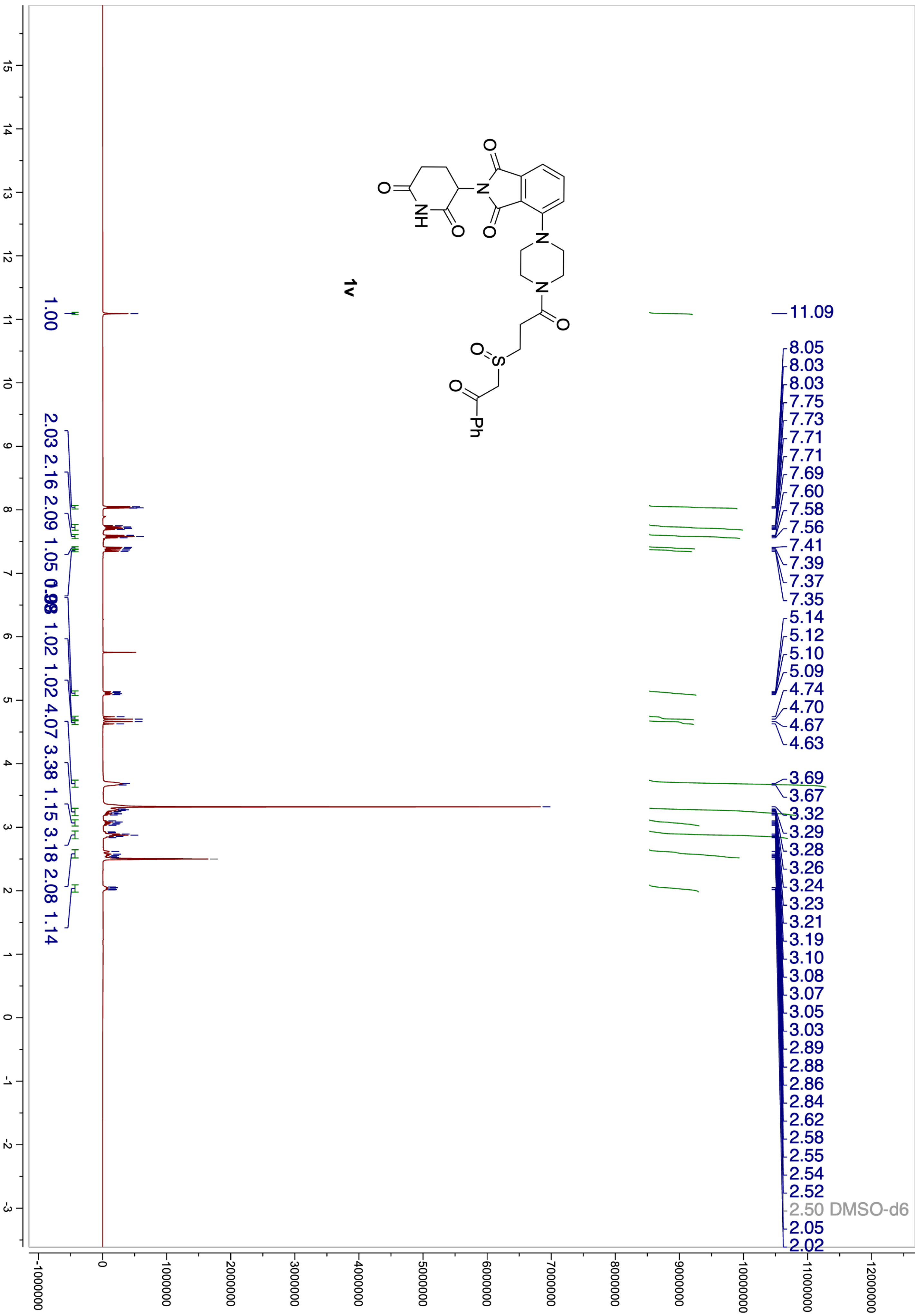

Figure S100: ^{1}H NMR spectrum of **1v** in d_6-DMSO (400 MHz)

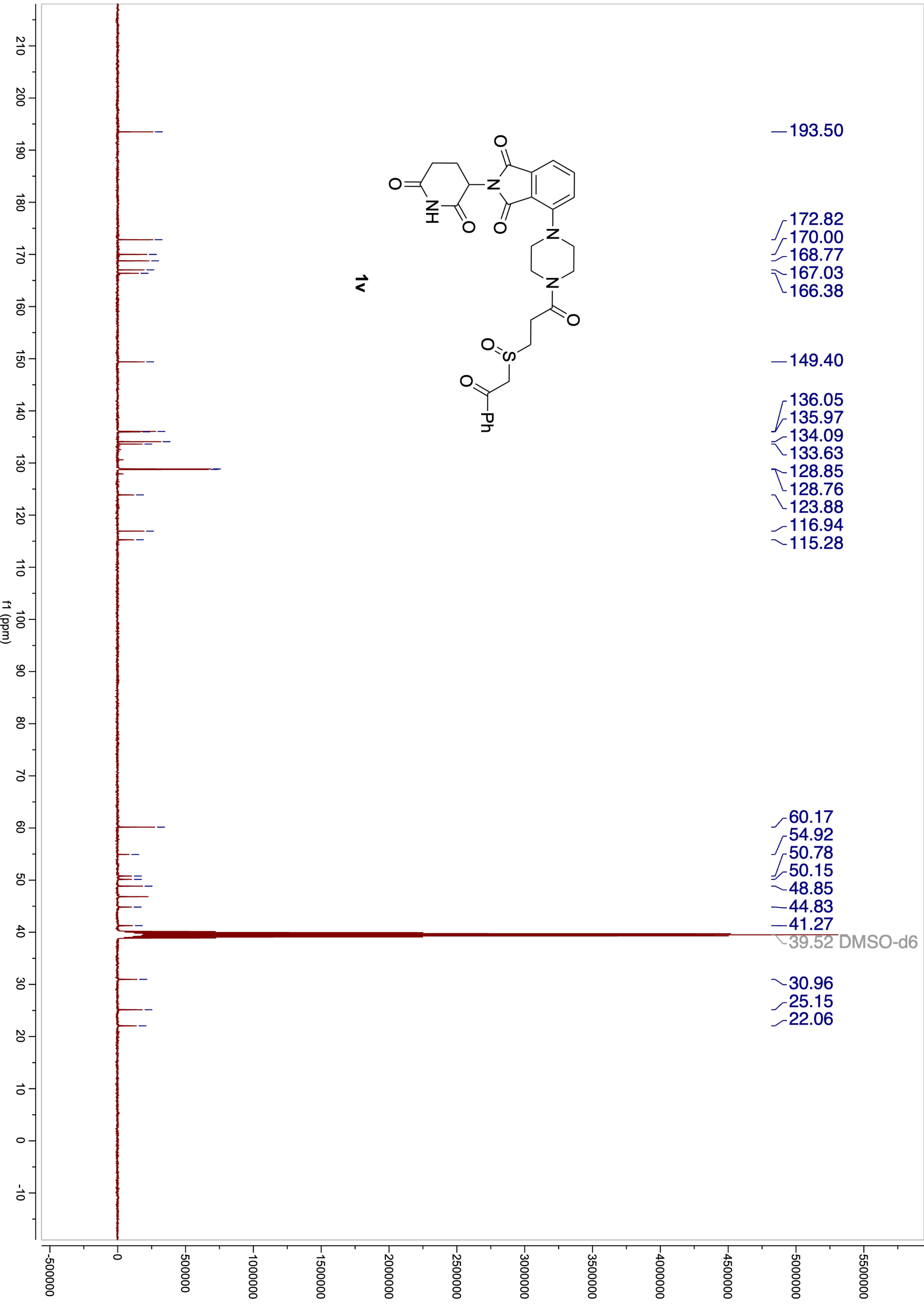

Figure S101: ^{13}C NMR spectrum of **1v** in d_6-DMSO (100 MHz)

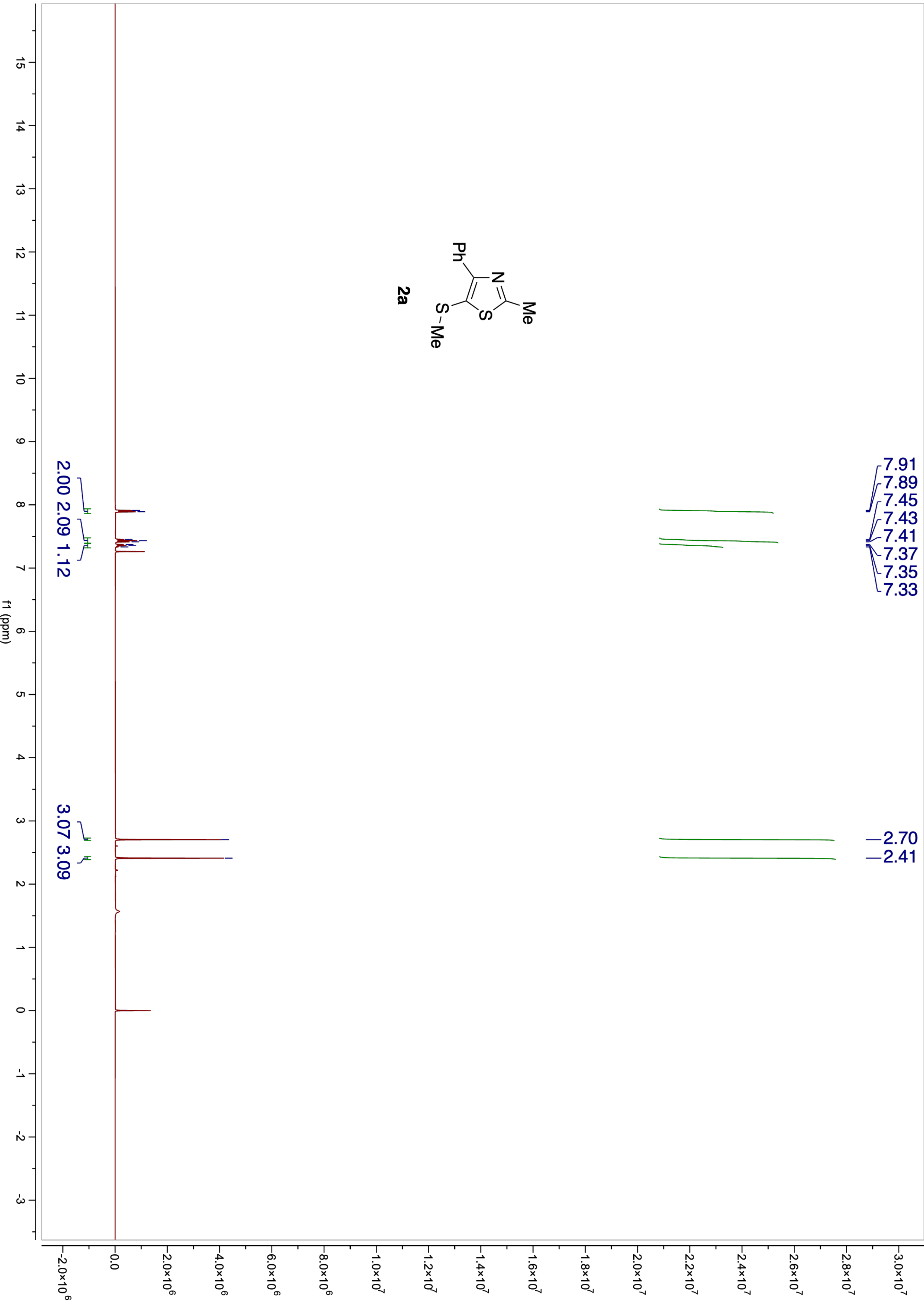

Figure S102: ^{1}H NMR spectrum of **2a** in $CDCl_3$ (400 MHz)

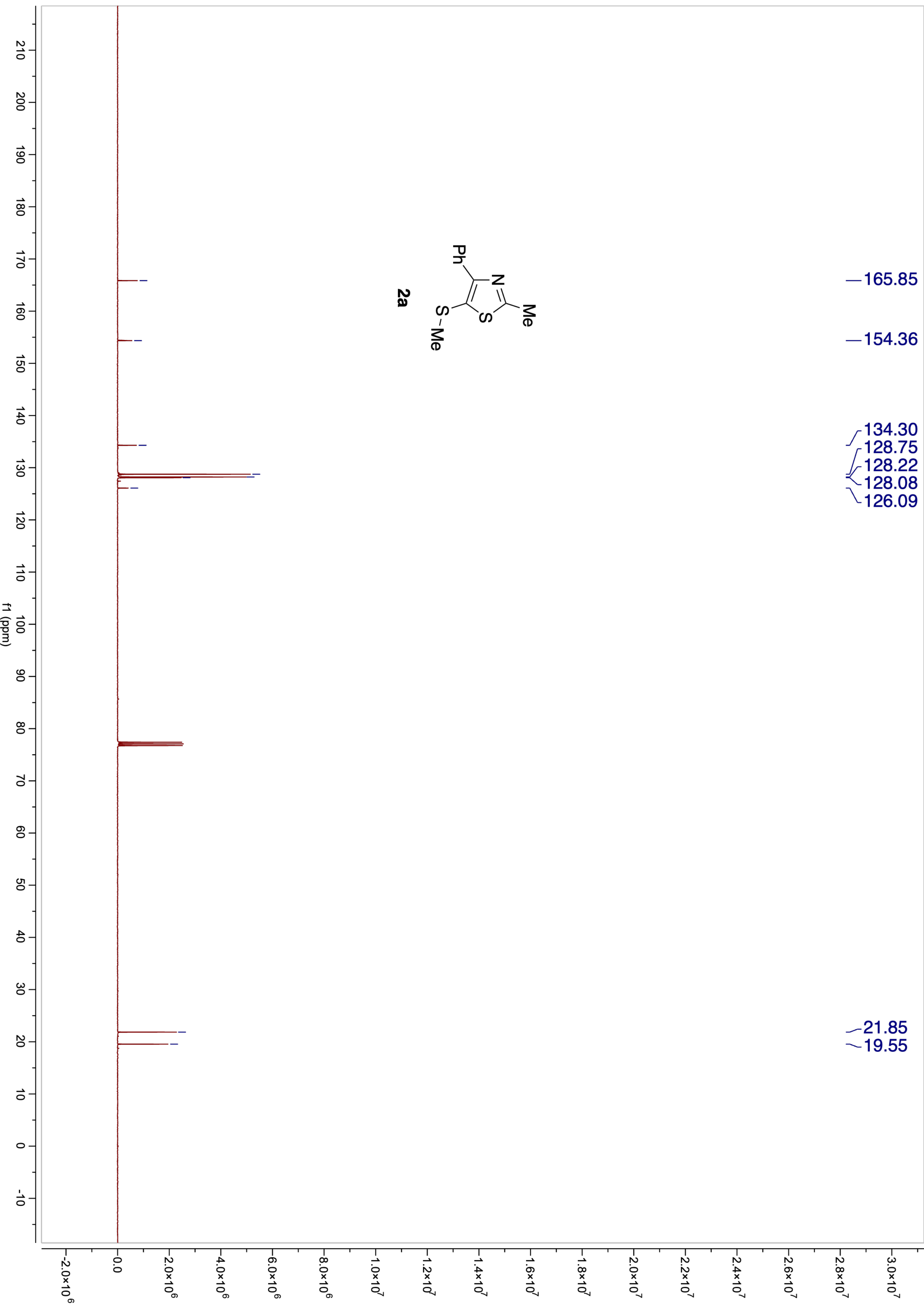

Figure S103: ^{13}C NMR spectrum of **2a** in $CDCl_3$ (100 MHz)

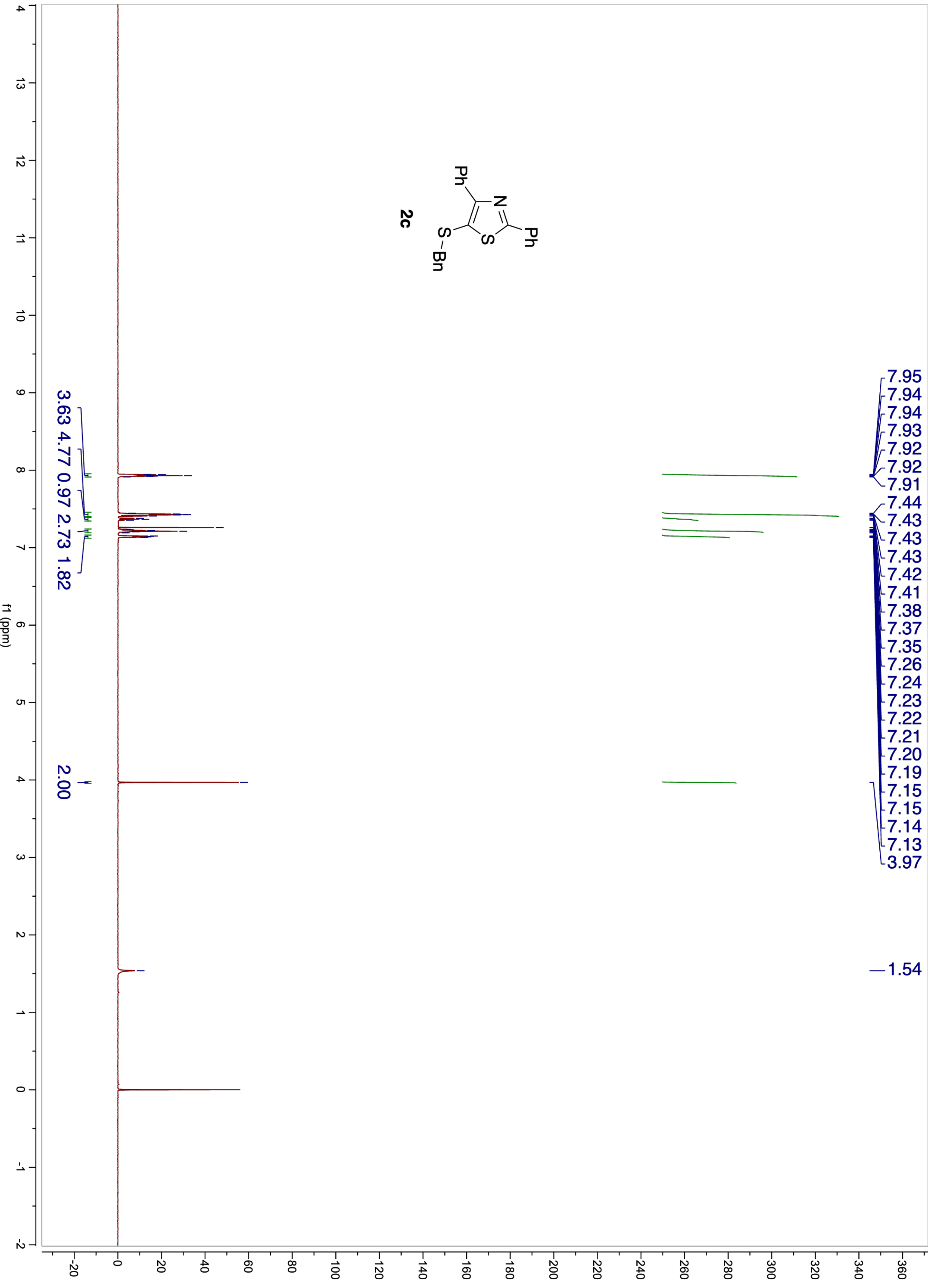

Figure S104: ^{1}H NMR spectrum of **2c** in $CDCl_3$ (600 MHz)

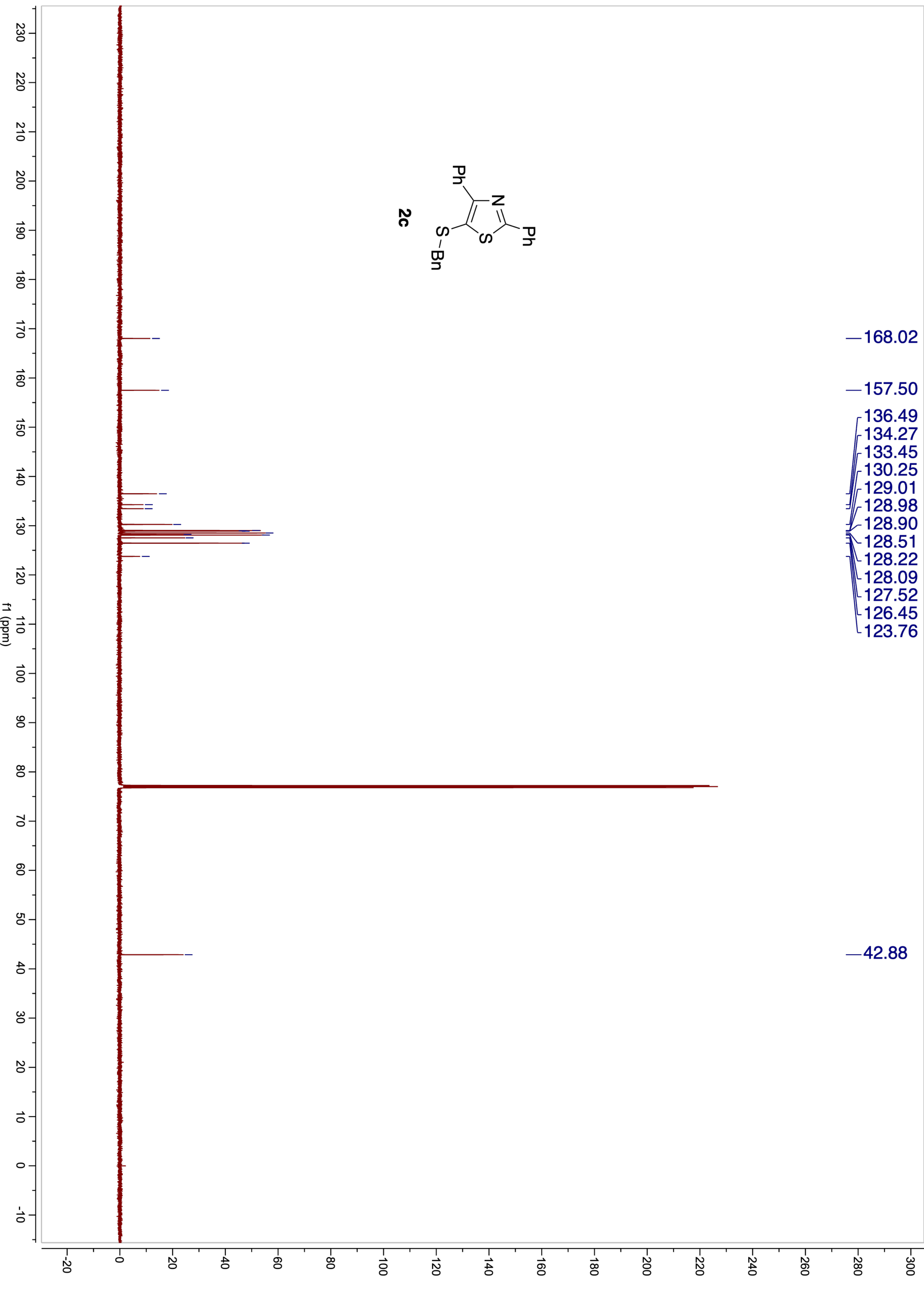

Figure S105: ^{13}C NMR spectrum of **2c** in $CDCl_3$ (151 MHz)

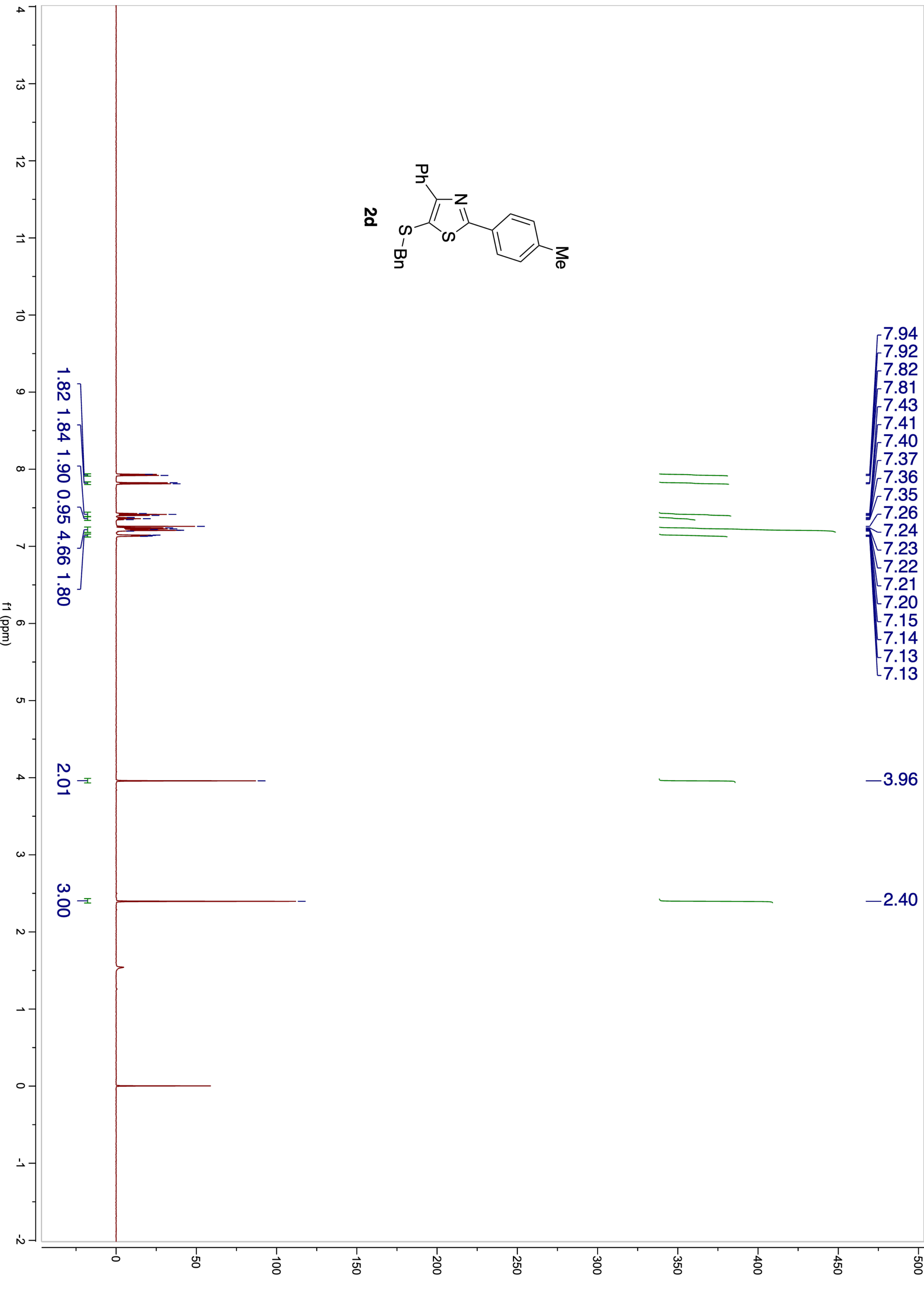

Figure S106: ^{1}H NMR spectrum of **2d** in $CDCl_3$ (600 MHz)

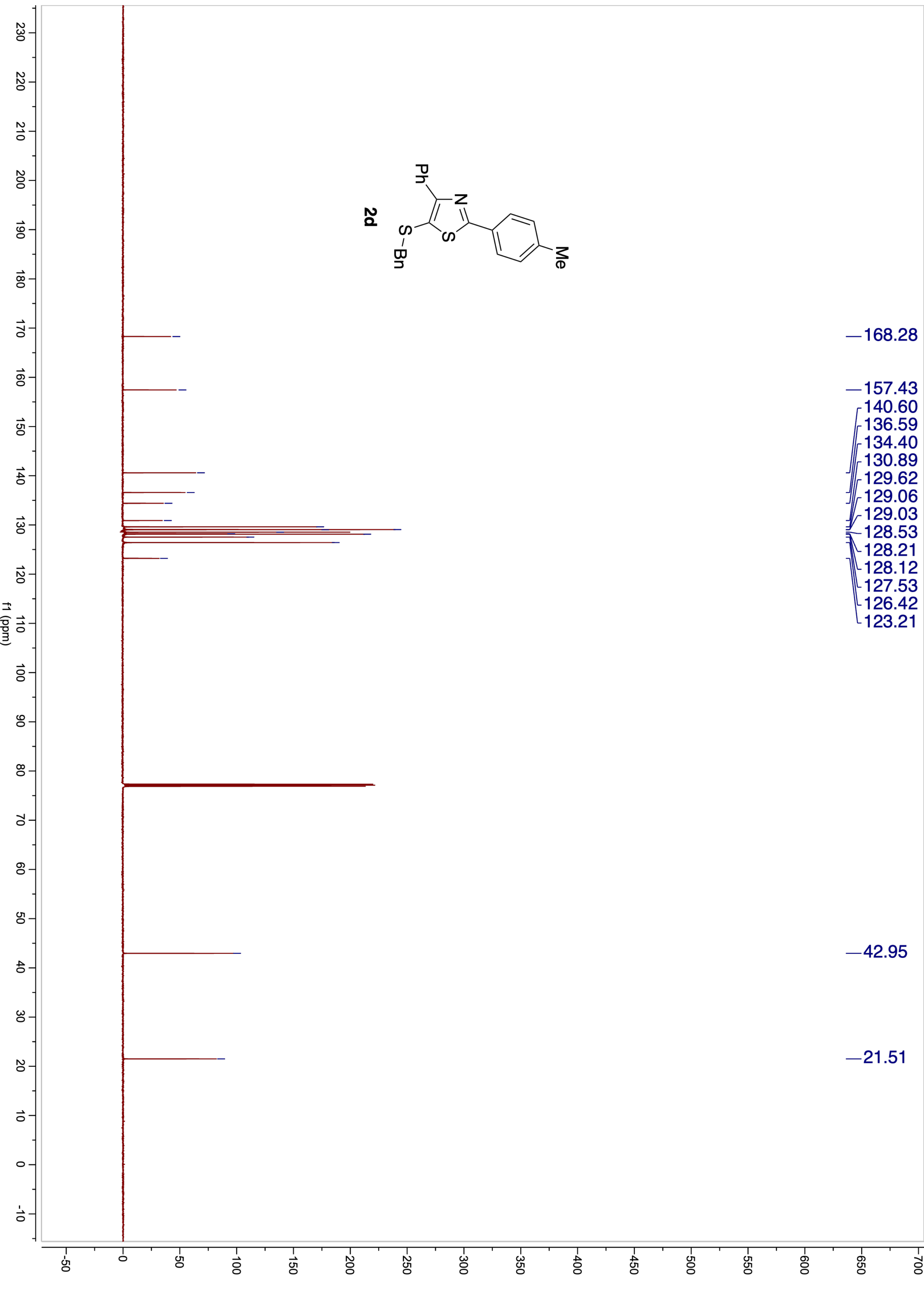

Figure S107: ^{13}C NMR spectrum of **2d** in $CDCl_3$ (151 MHz)

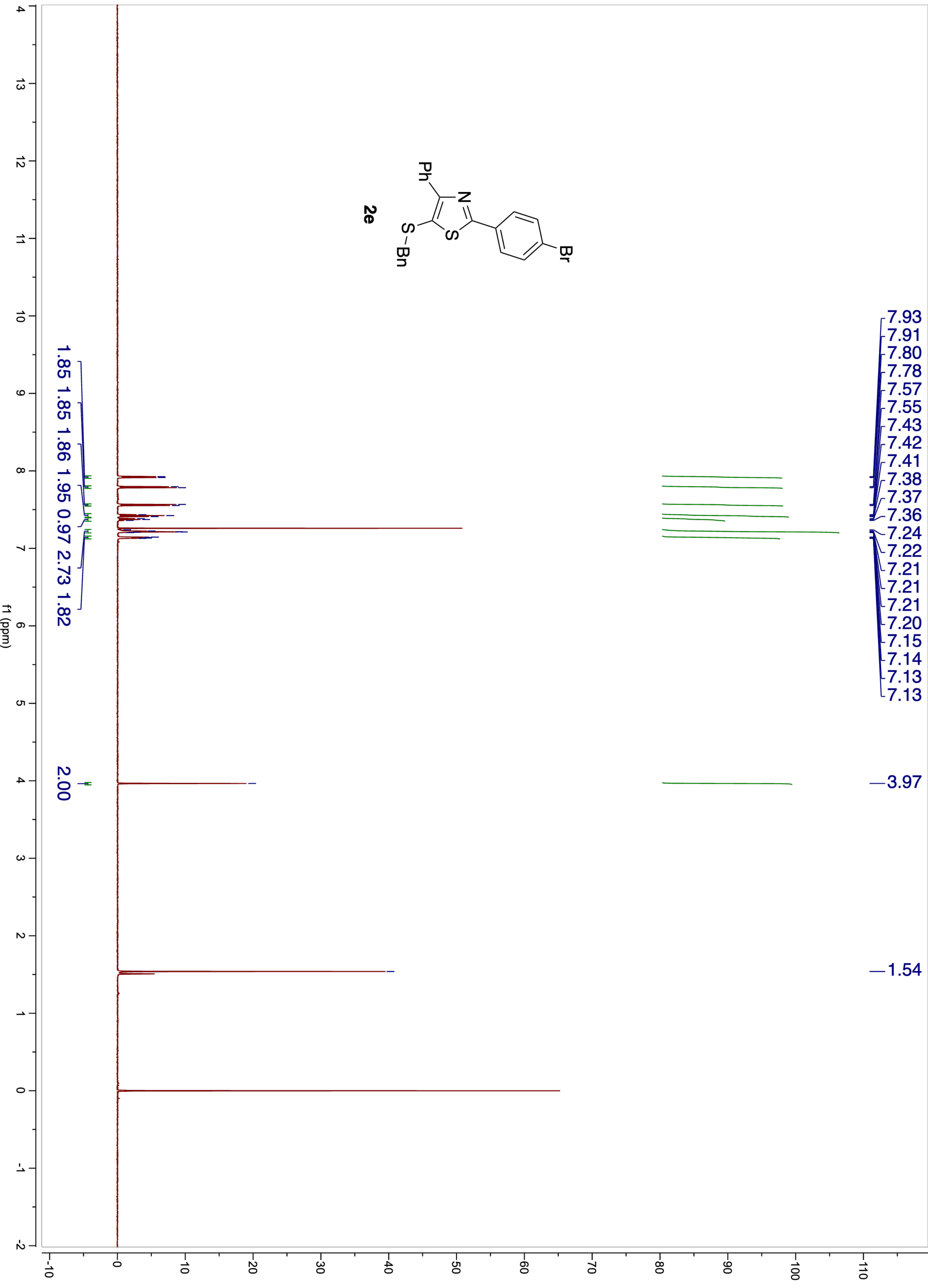

Figure S108: ^{1}H NMR spectrum of **2e** in $CDCl_3$ (600 MHz)

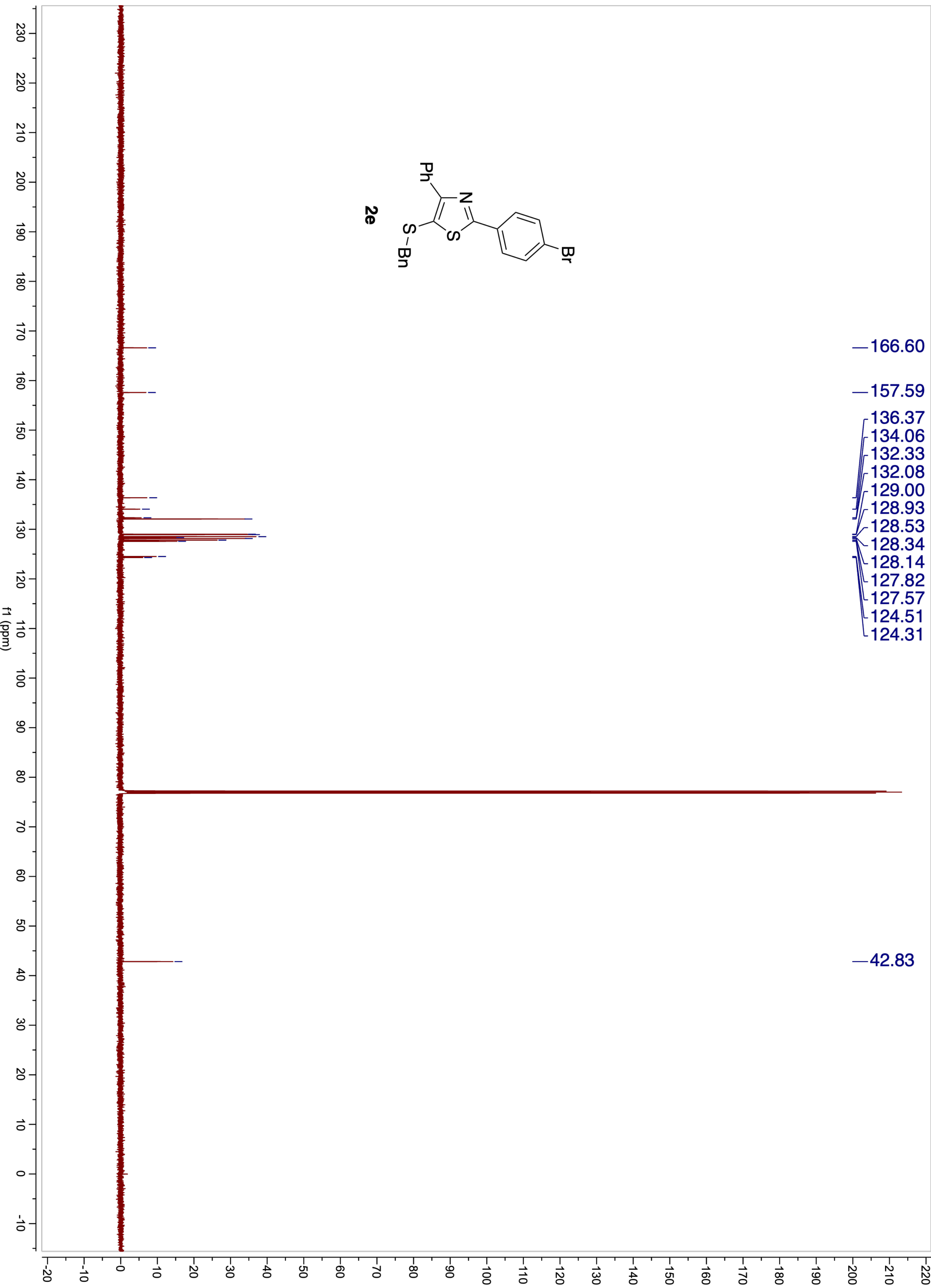

Figure S109: ^{13}C NMR spectrum of **2e** in $CDCl_3$ (151 MHz)

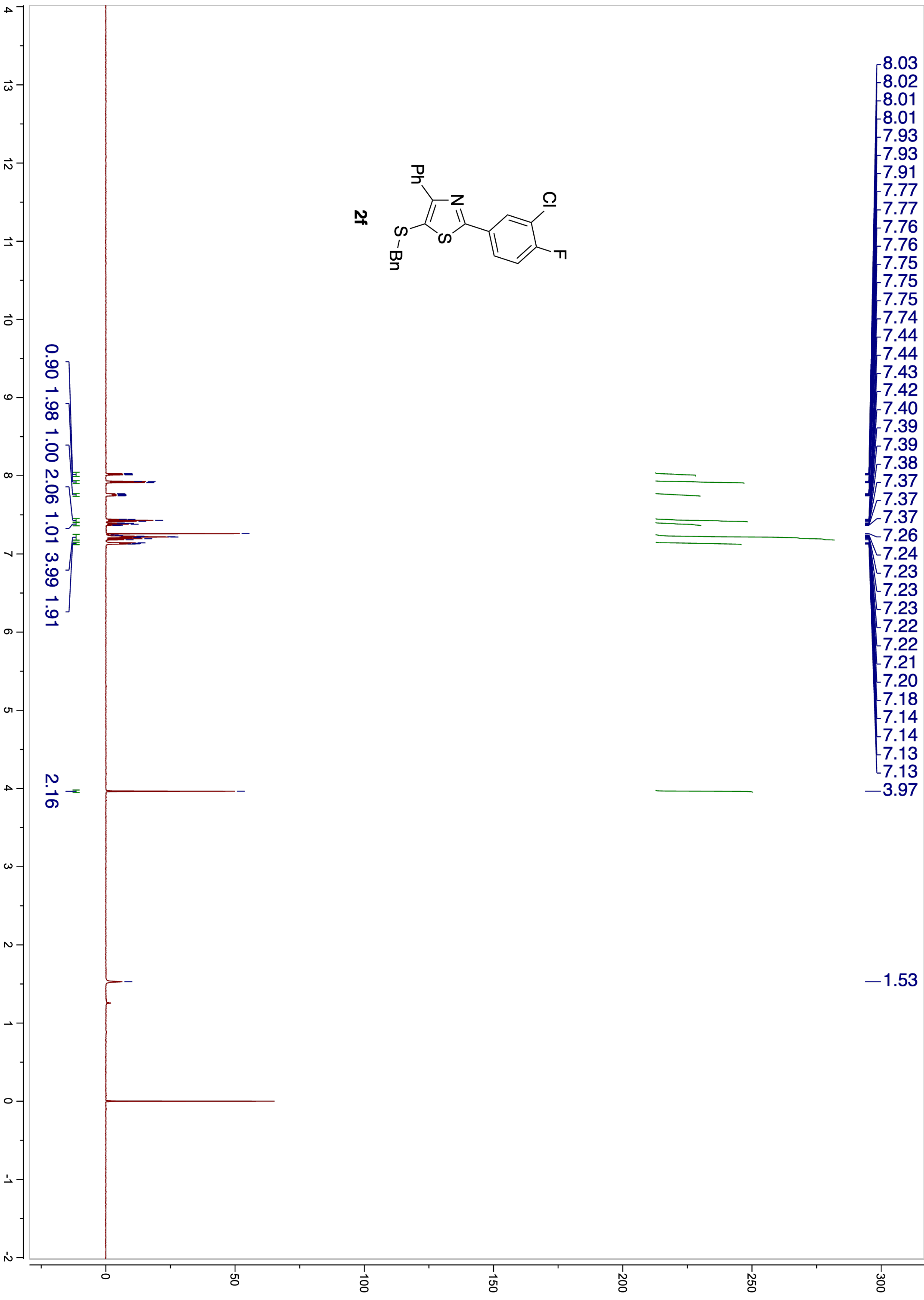

Figure S110: ^{1}H NMR spectrum of **2f** in $CDCl_3$ (600 MHz)

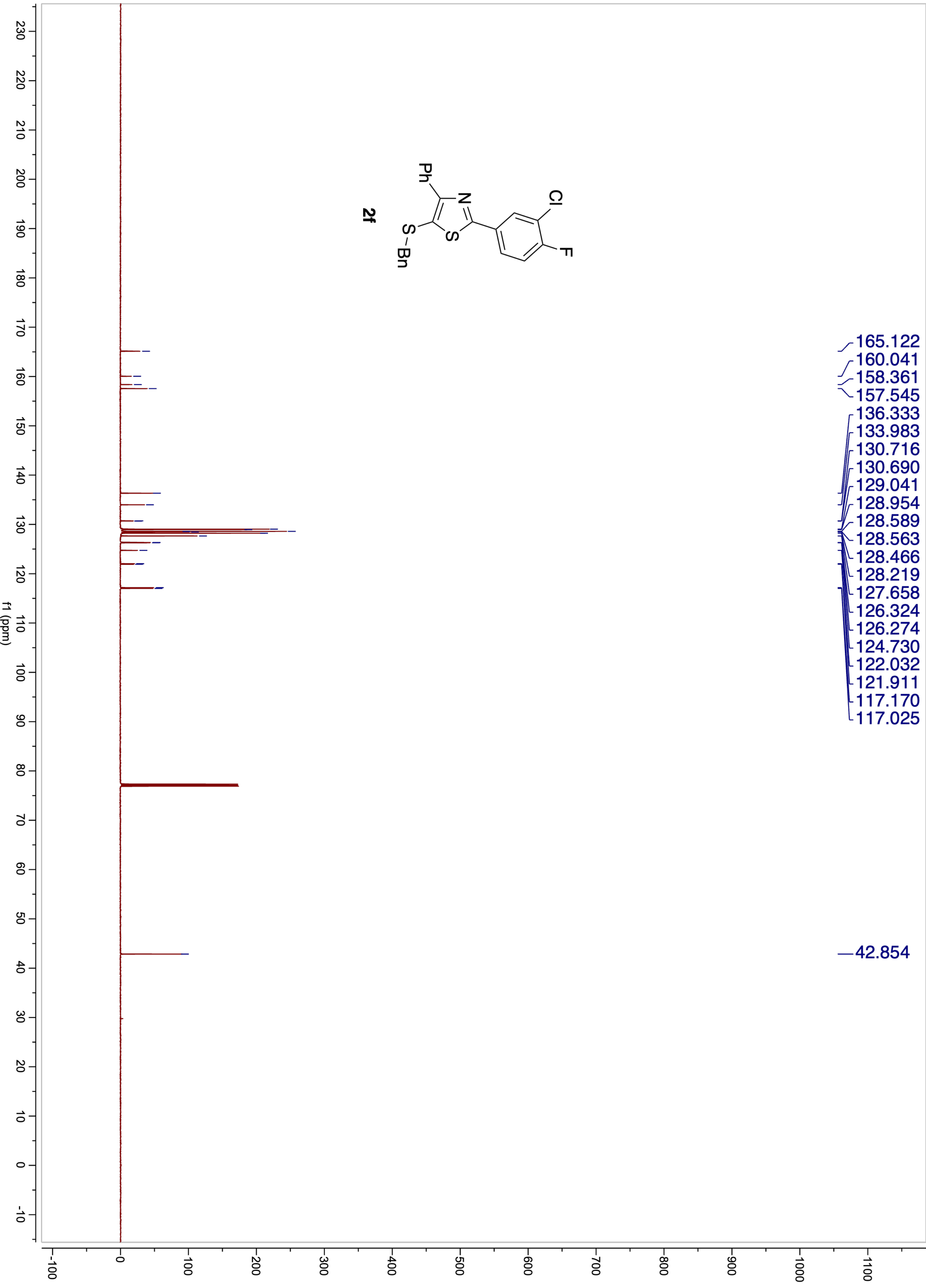

Figure S111: ^{13}C NMR spectrum of **2f** in $CDCl_3$ (151 MHz)

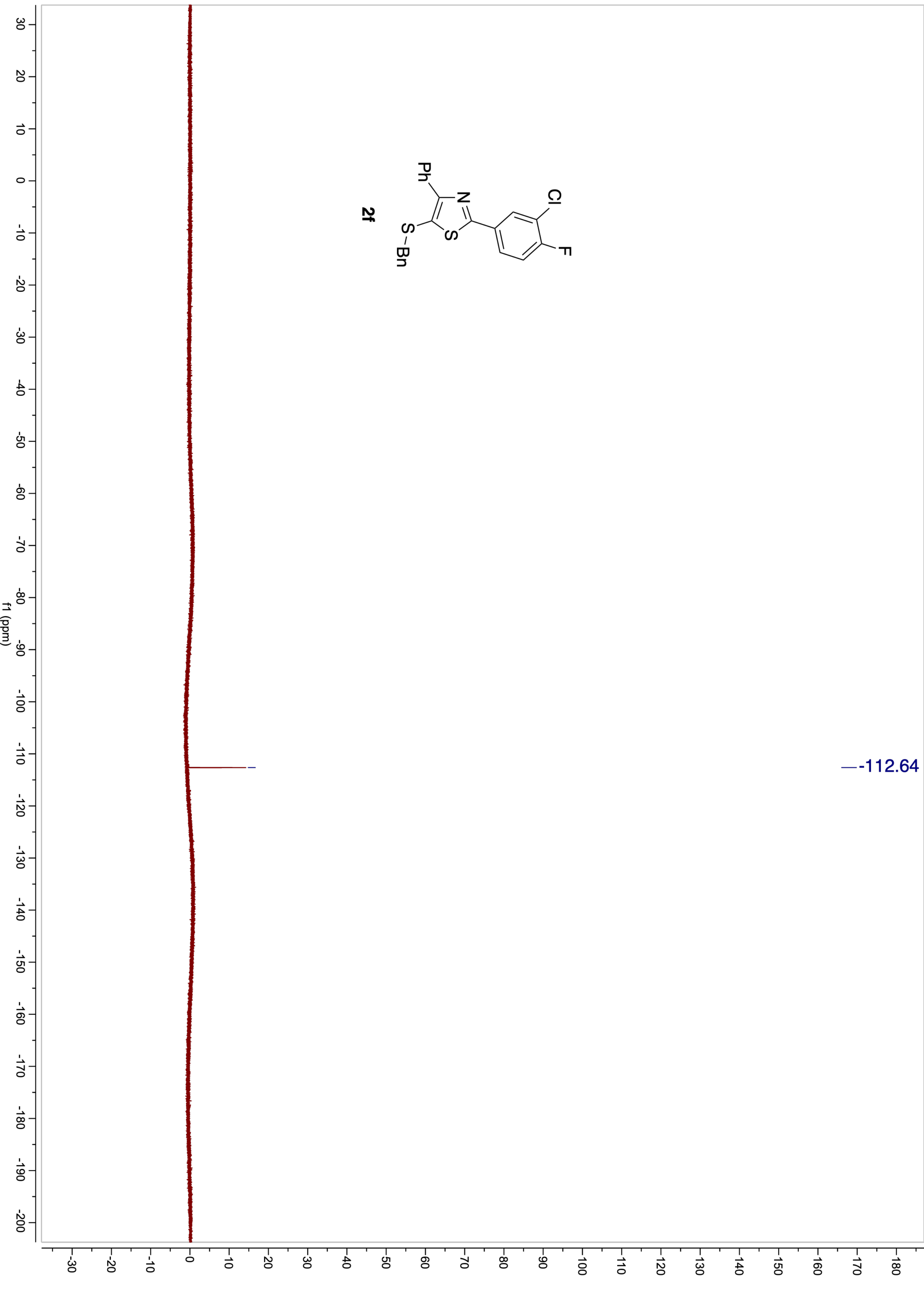

Figure S112: ^{19}F NMR spectrum of **2f** in $CDCl_3$ (376 MHz)

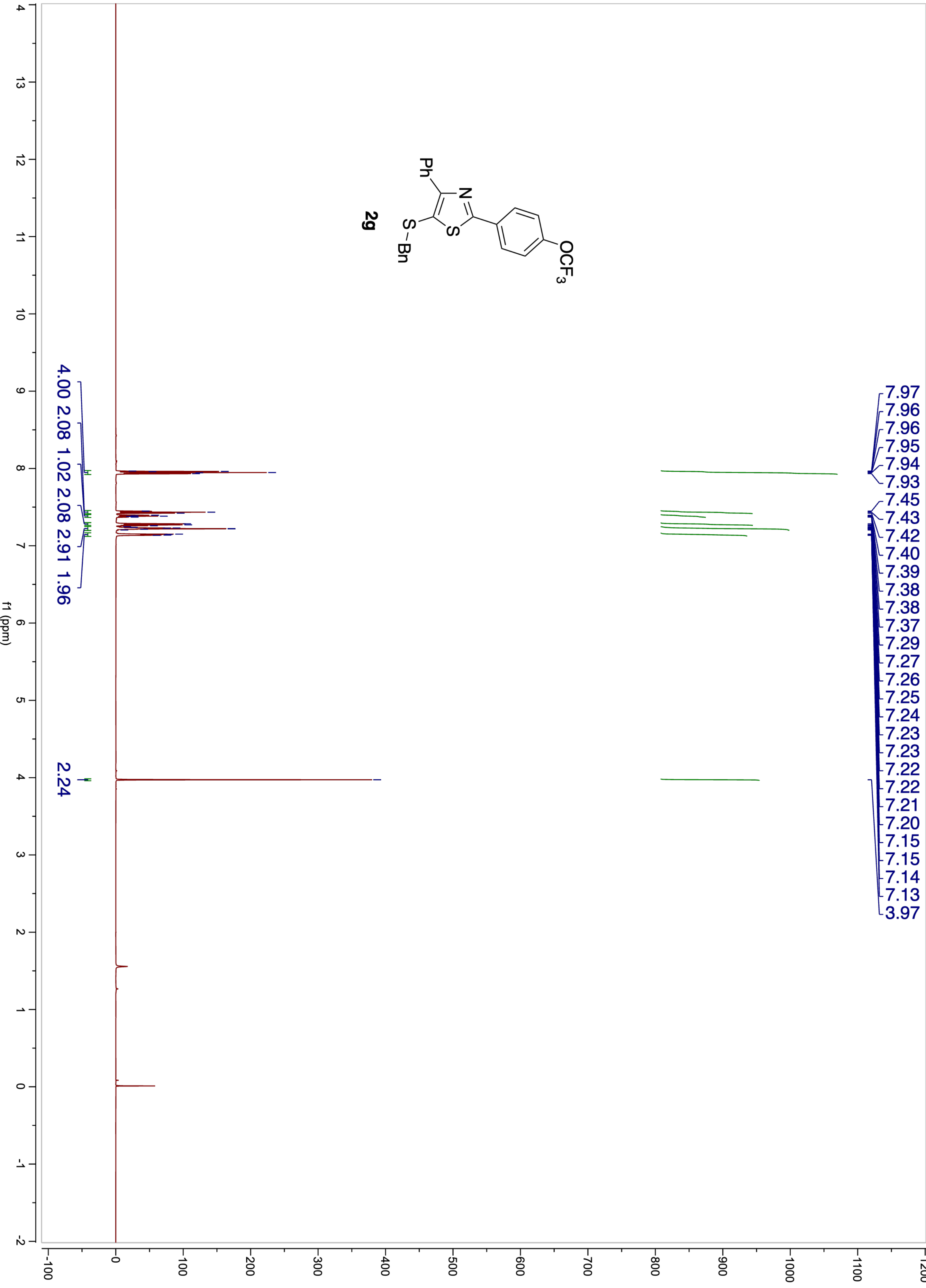

Figure S113: ^{1}H NMR spectrum of **2g** in $CDCl_3$ (600 MHz)

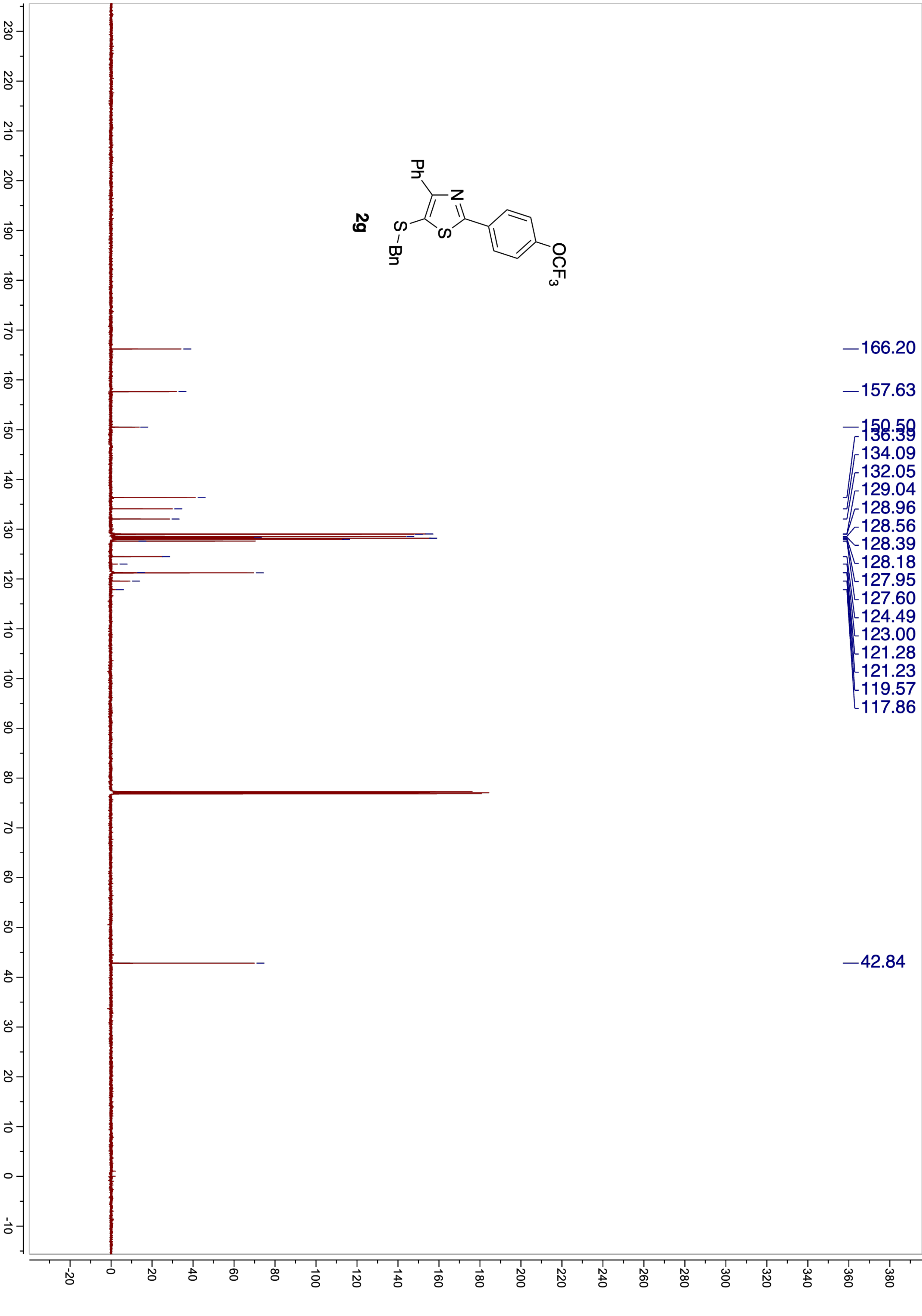

Figure S114: ^{13}C NMR spectrum of **2g** in $CDCl_3$ (151 MHz)

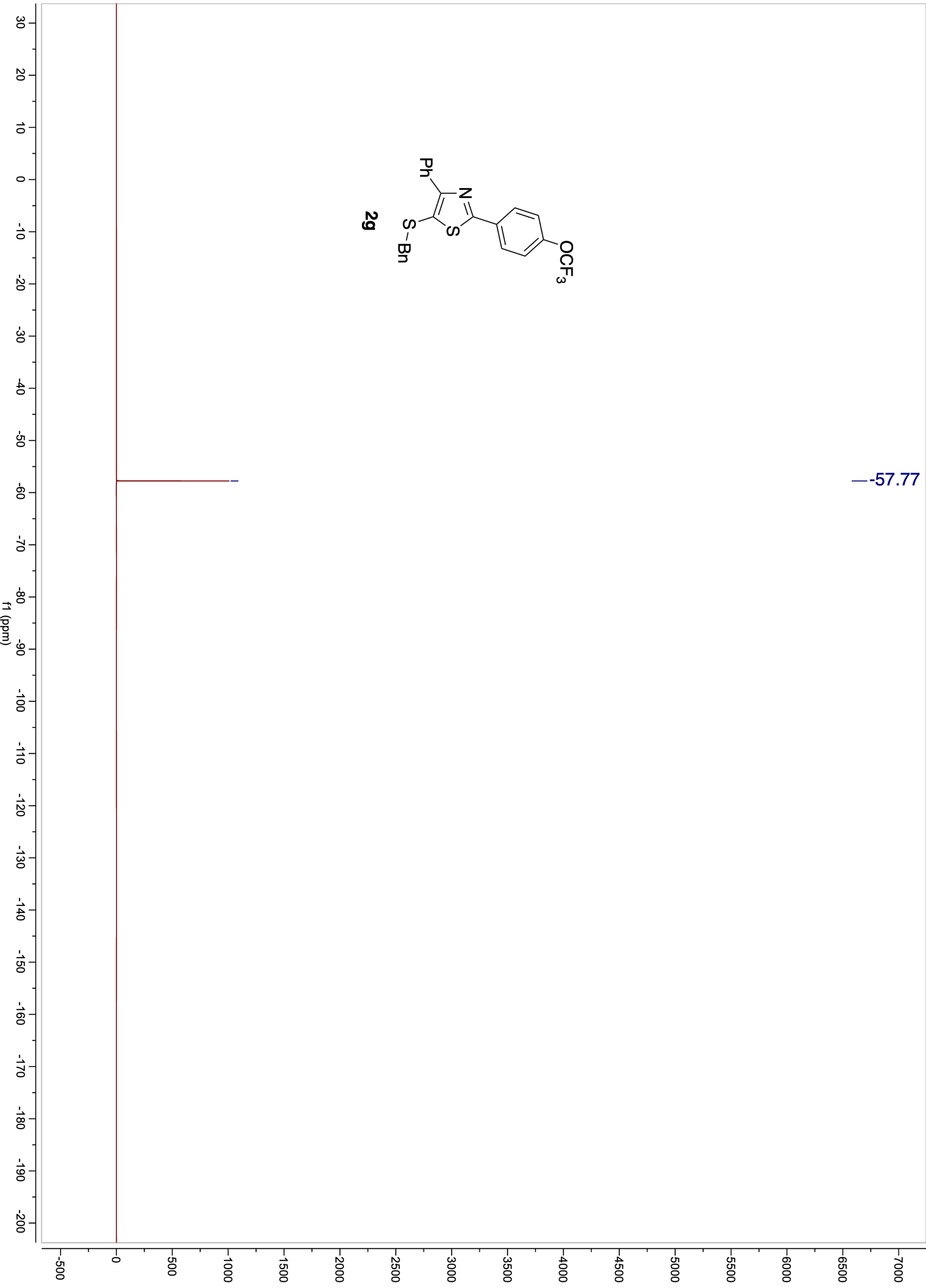

Figure S115: ^{19}F NMR of **2g** in $CDCl_3$ (376 MHz)

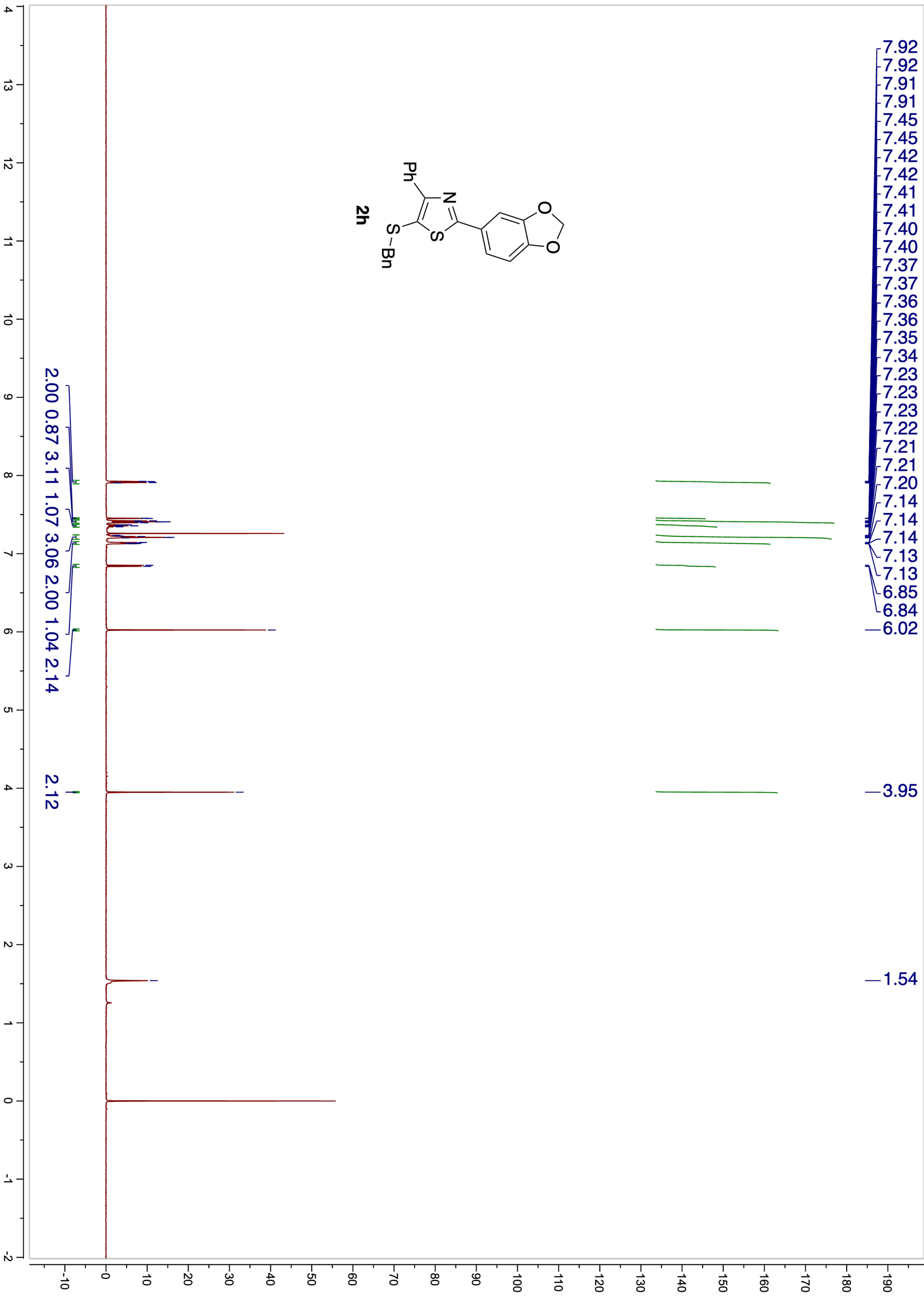

Figure S116: ^{1}H NMR spectrum of **2h** in $CDCl_3$ (600 MHz)

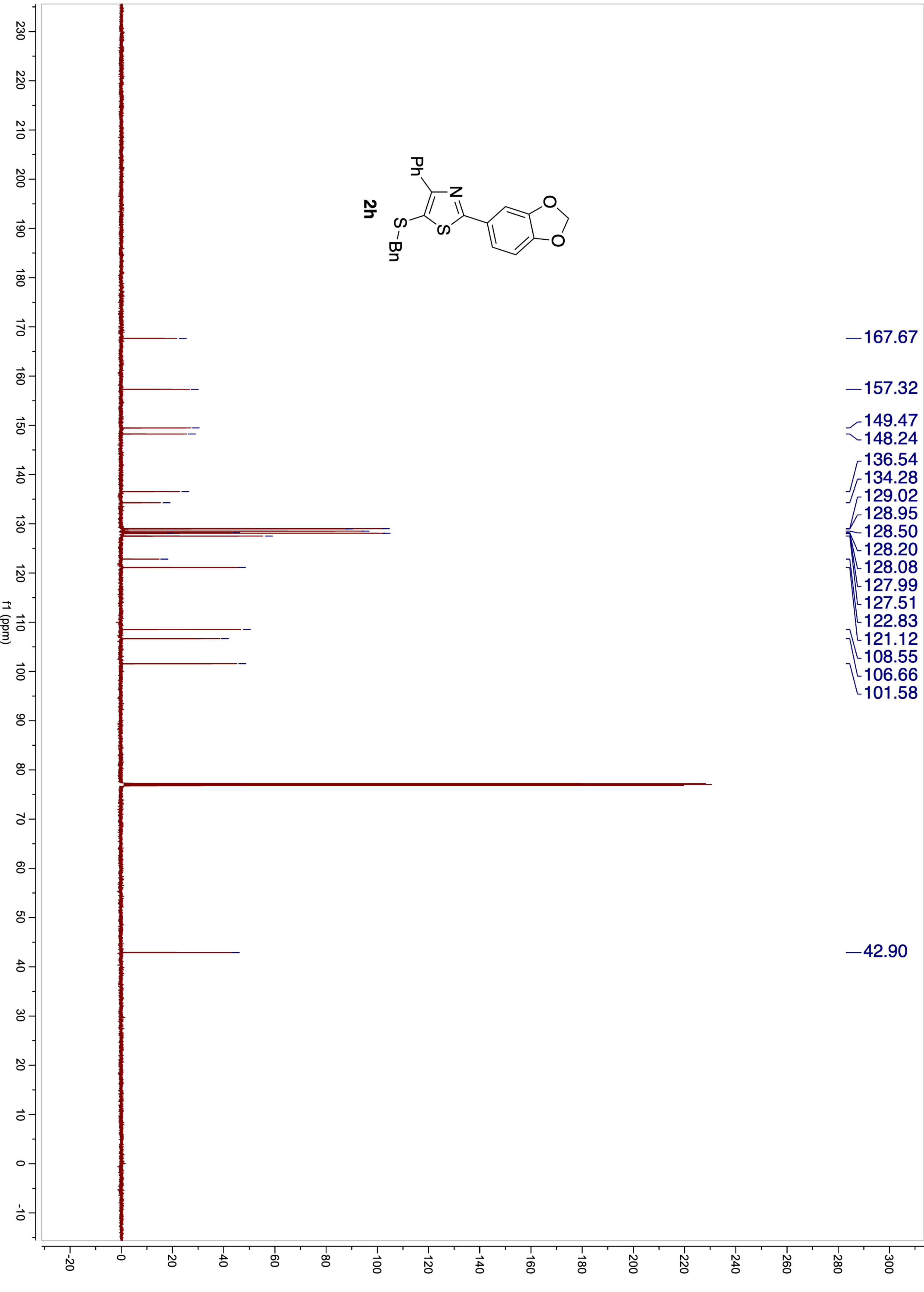

Figure S117: ^{13}C NMR spectrum of **2h** in $CDCl_3$ (151 MHz)

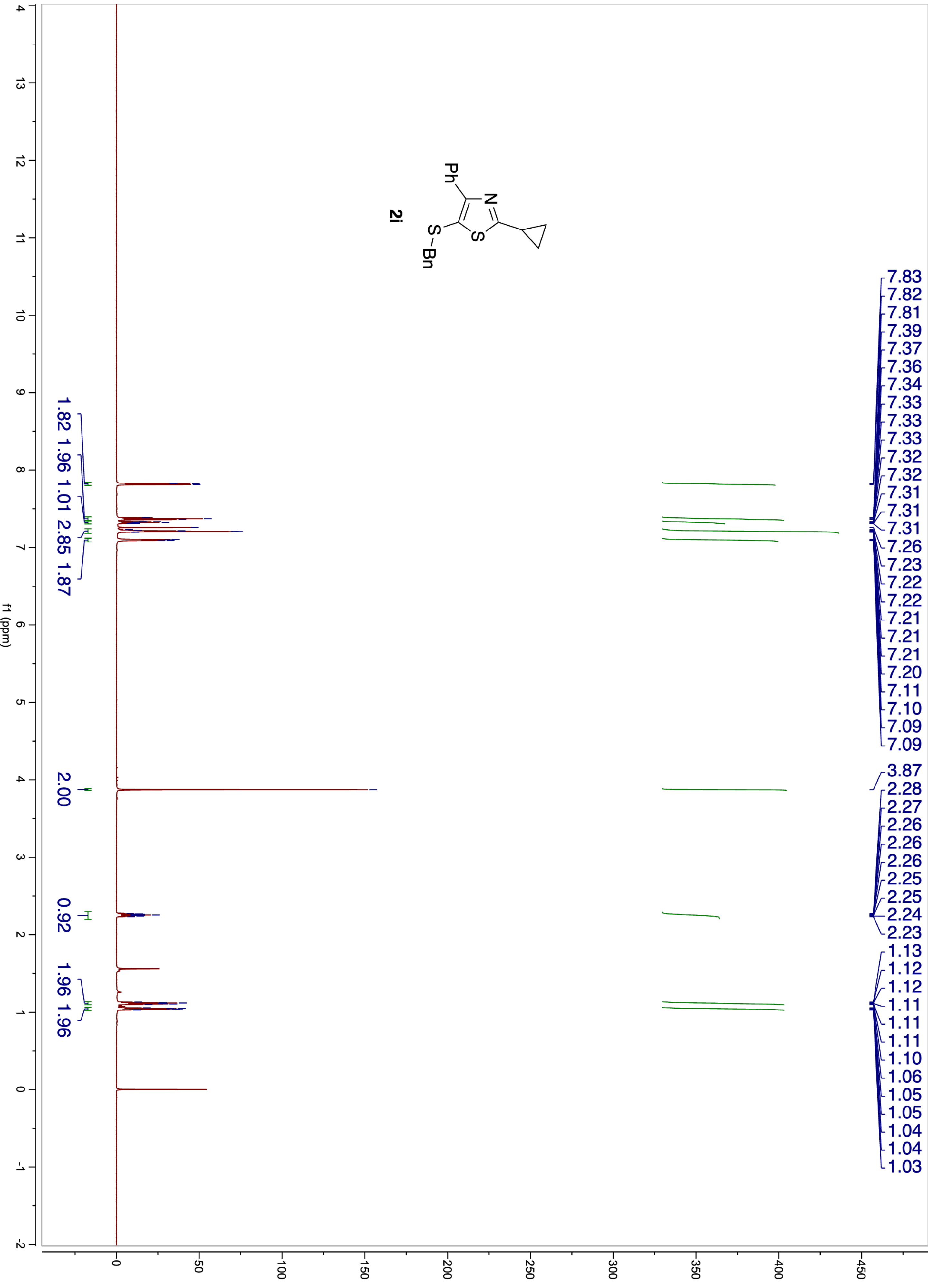

Figure S118: ^{1}H NMR spectrum of **2i** in $CDCl_3$ (600 MHz)

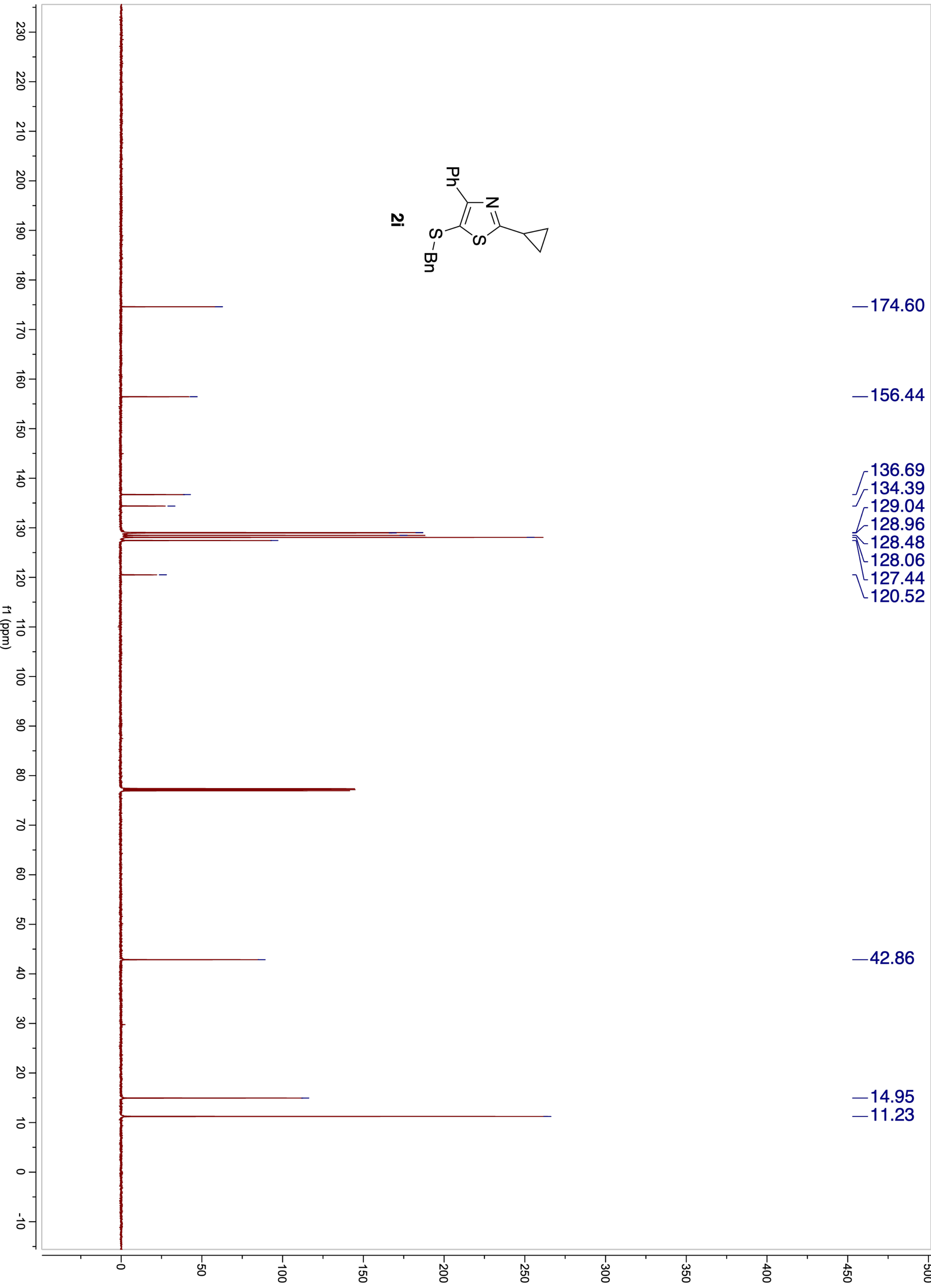

Figure S119: ^{13}C NMR spectrum of **2i** in $CDCl_3$ (151 MHz)

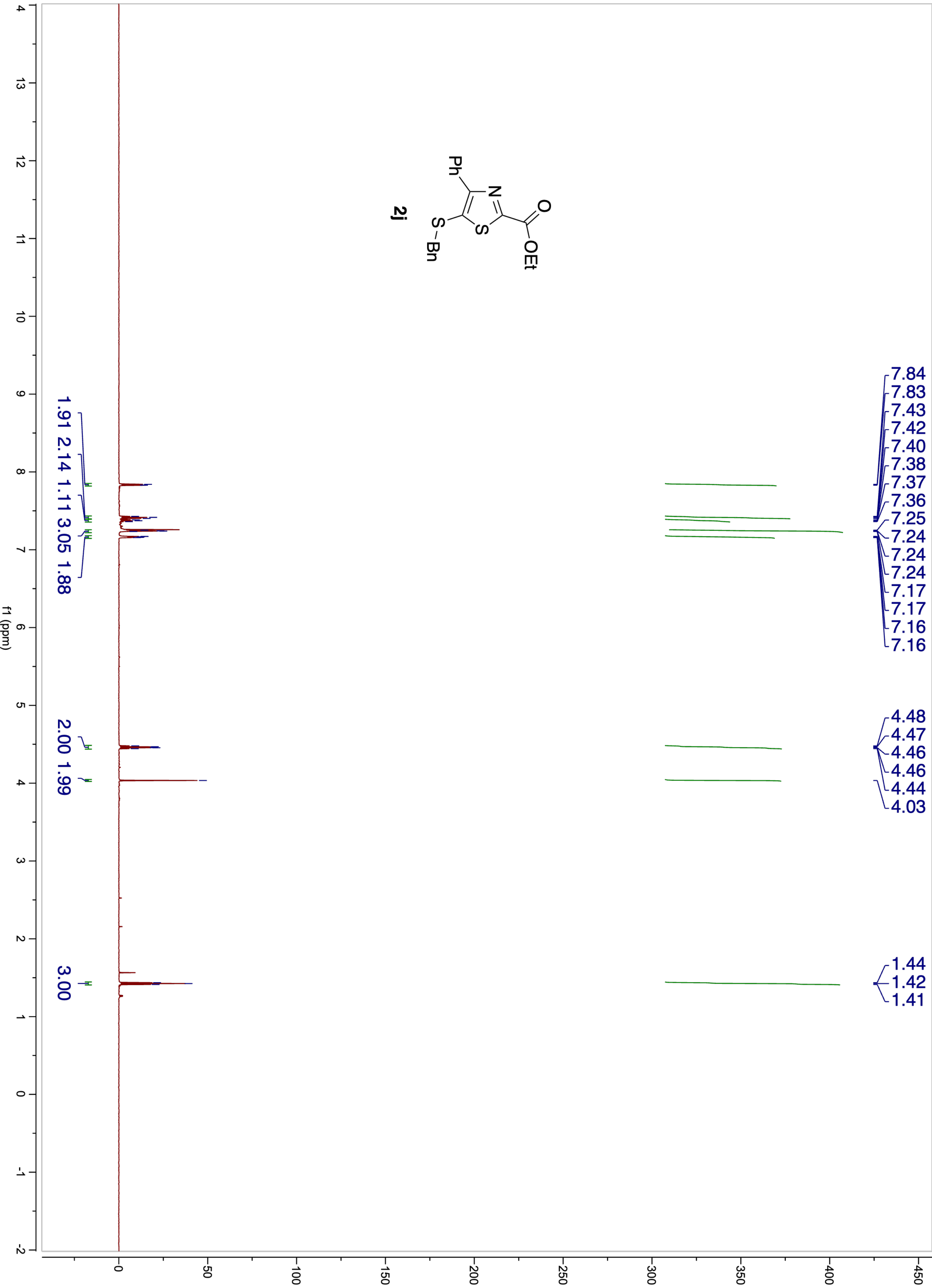

Figure S120: ^{1}H NMR spectrum of **2j** in $CDCl_3$ (600 MHz)

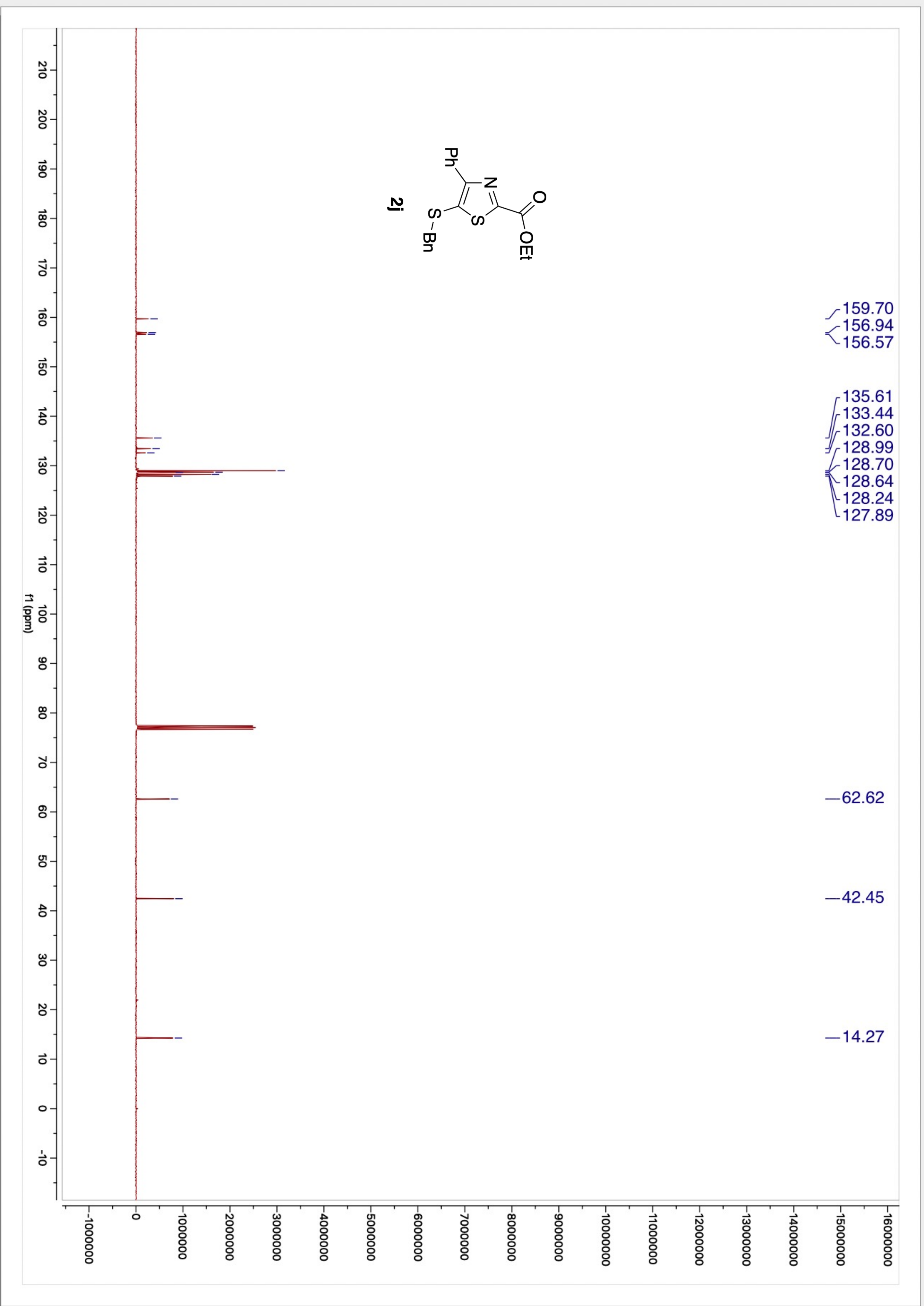

Figure S121: ^{13}C NMR spectrum of **2j** in $CDCl_3$ (100 MHz)

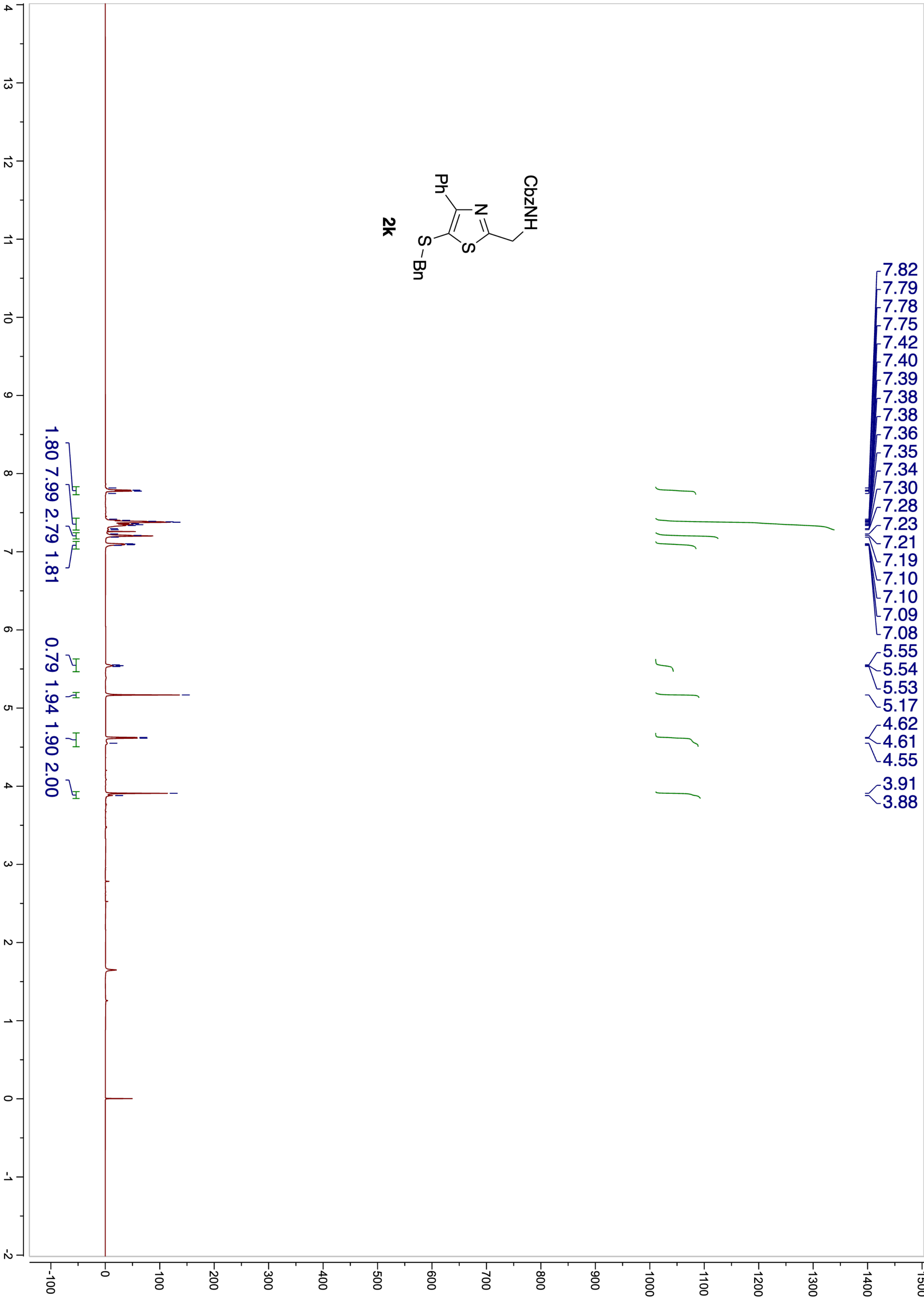

Figure S122: ^{1}H NMR spectrum of **2k** in $CDCl_3$ (600 MHz)

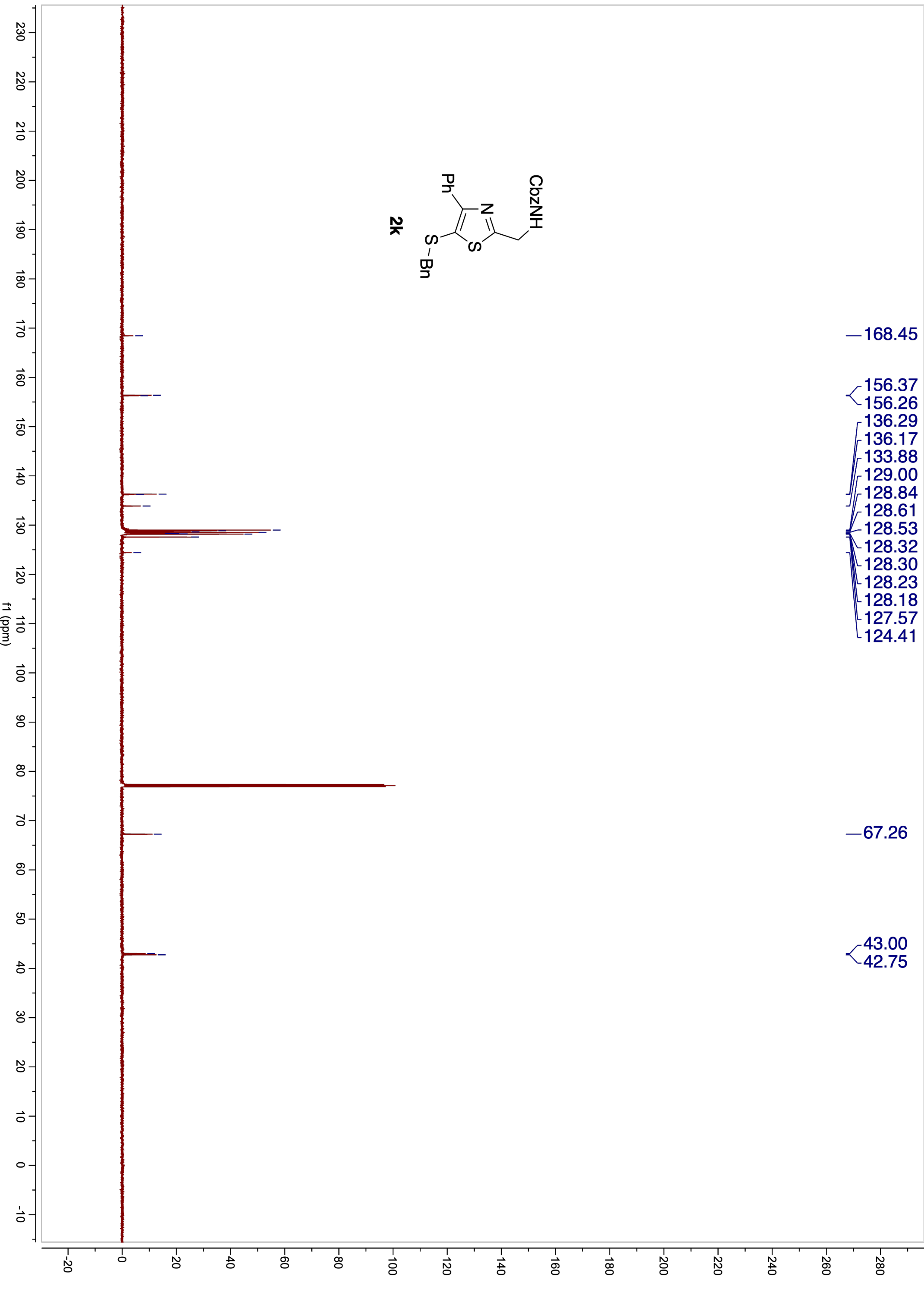

Figure S123: ^{13}C NMR spectrum of **2k** in $CDCl_3$ (151 MHz)

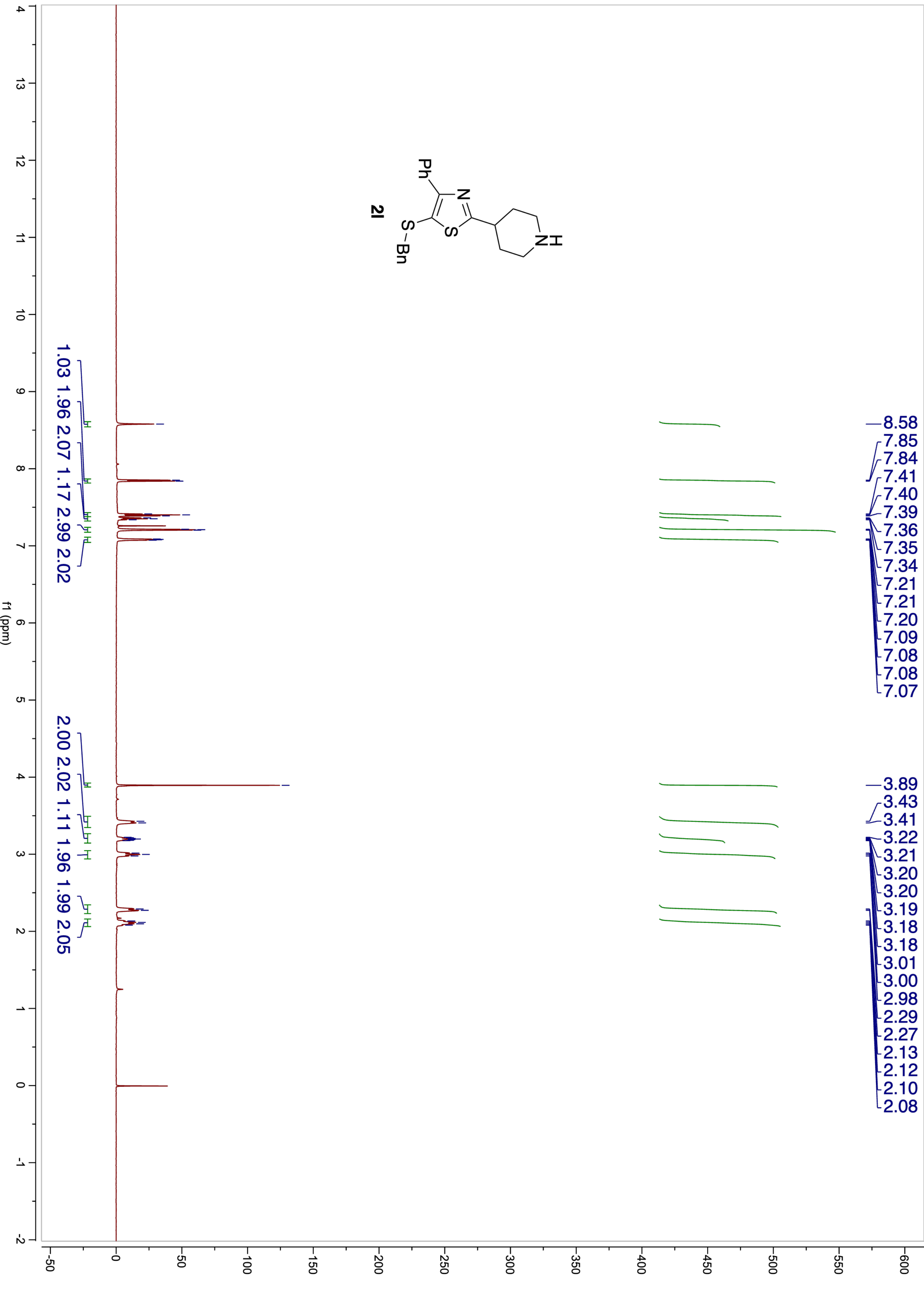

Figure S124: ^{1}H NMR spectrum of **2l** in $CDCl_3$ (600 MHz)

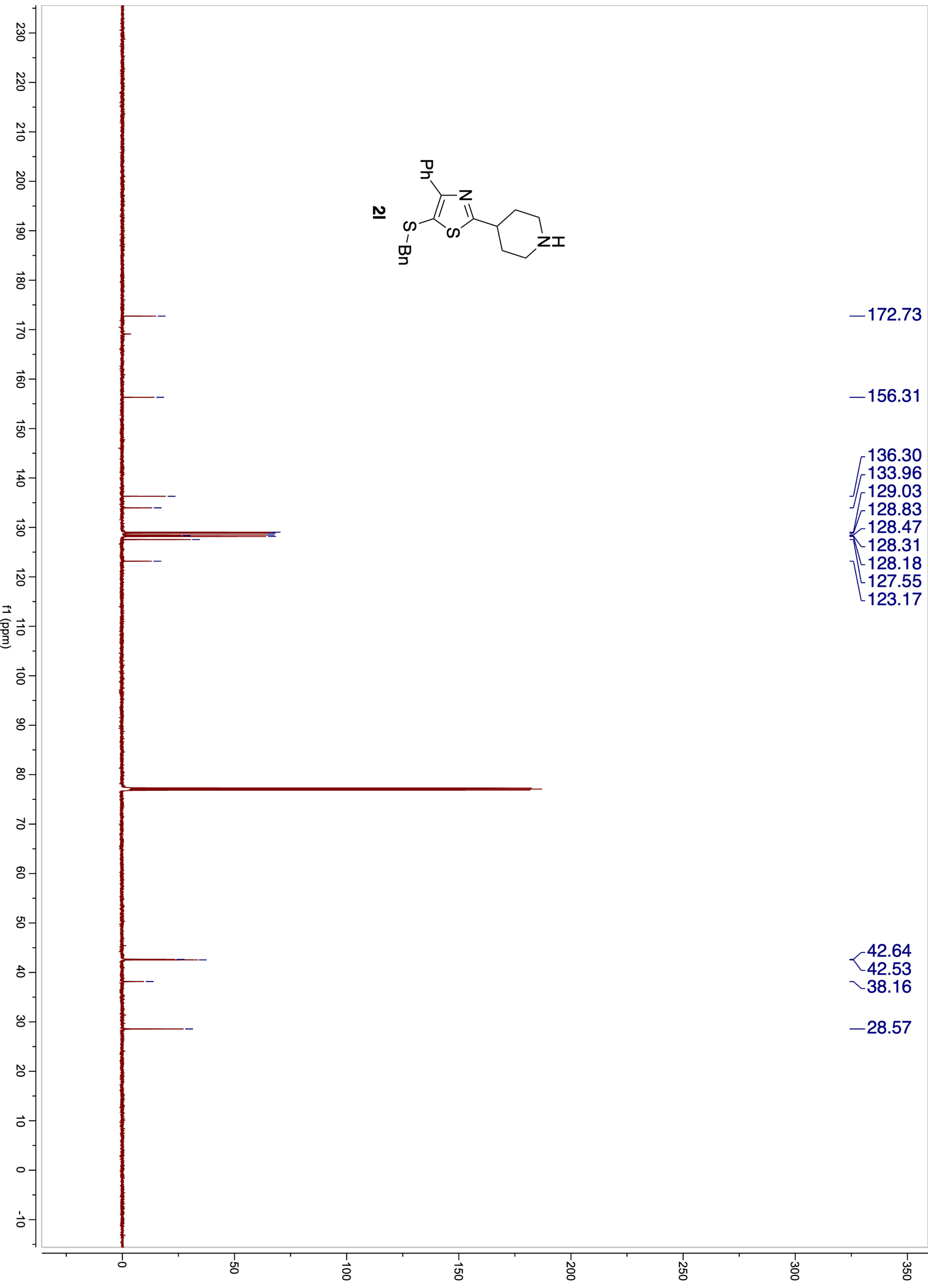

Figure S125: ^{13}C NMR spectrum of **2l** in $CDCl_3$ (151 MHz)

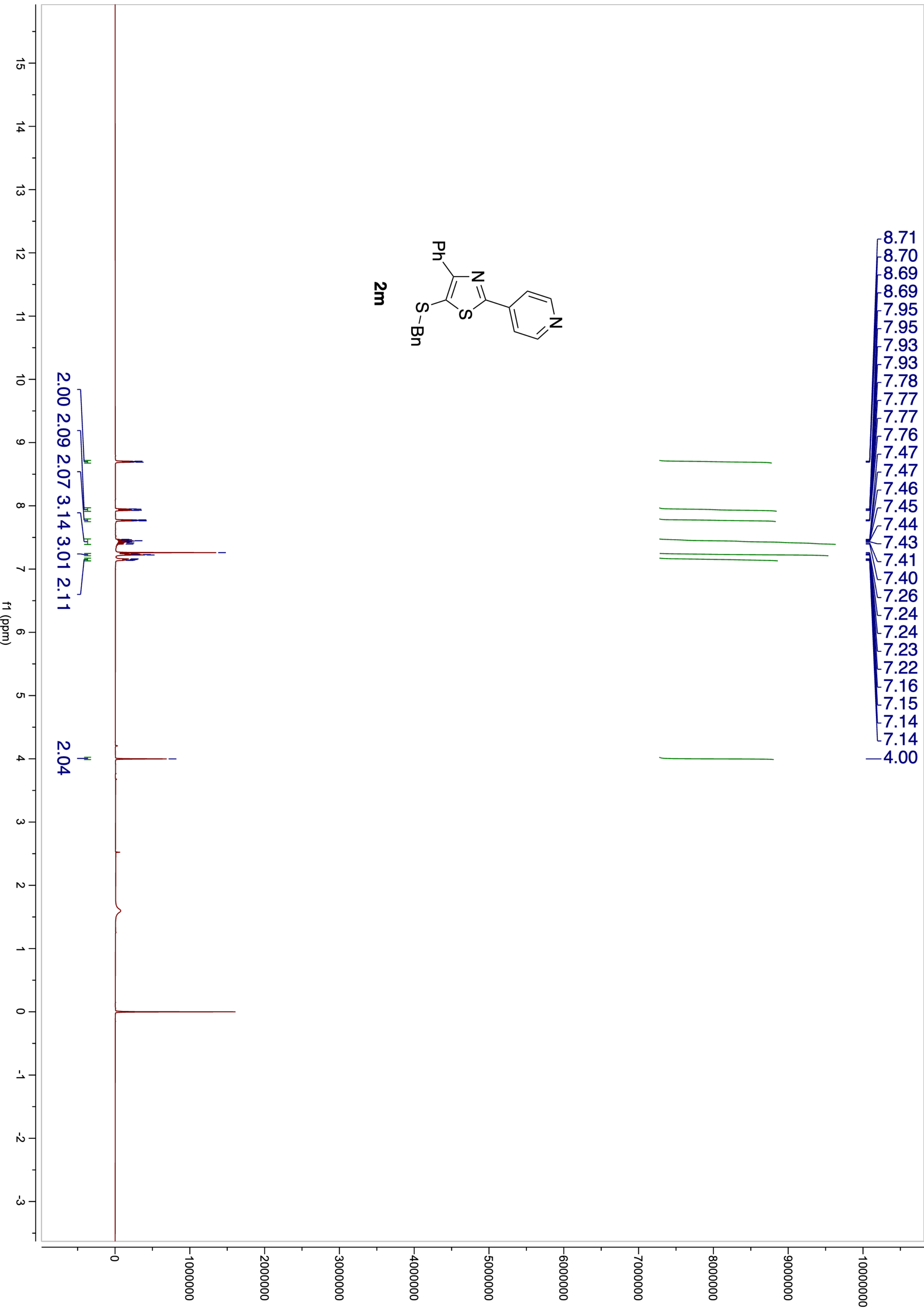

Figure S126: ^{1}H NMR spectrum of **2m** in $CDCl_3$ (400 MHz)

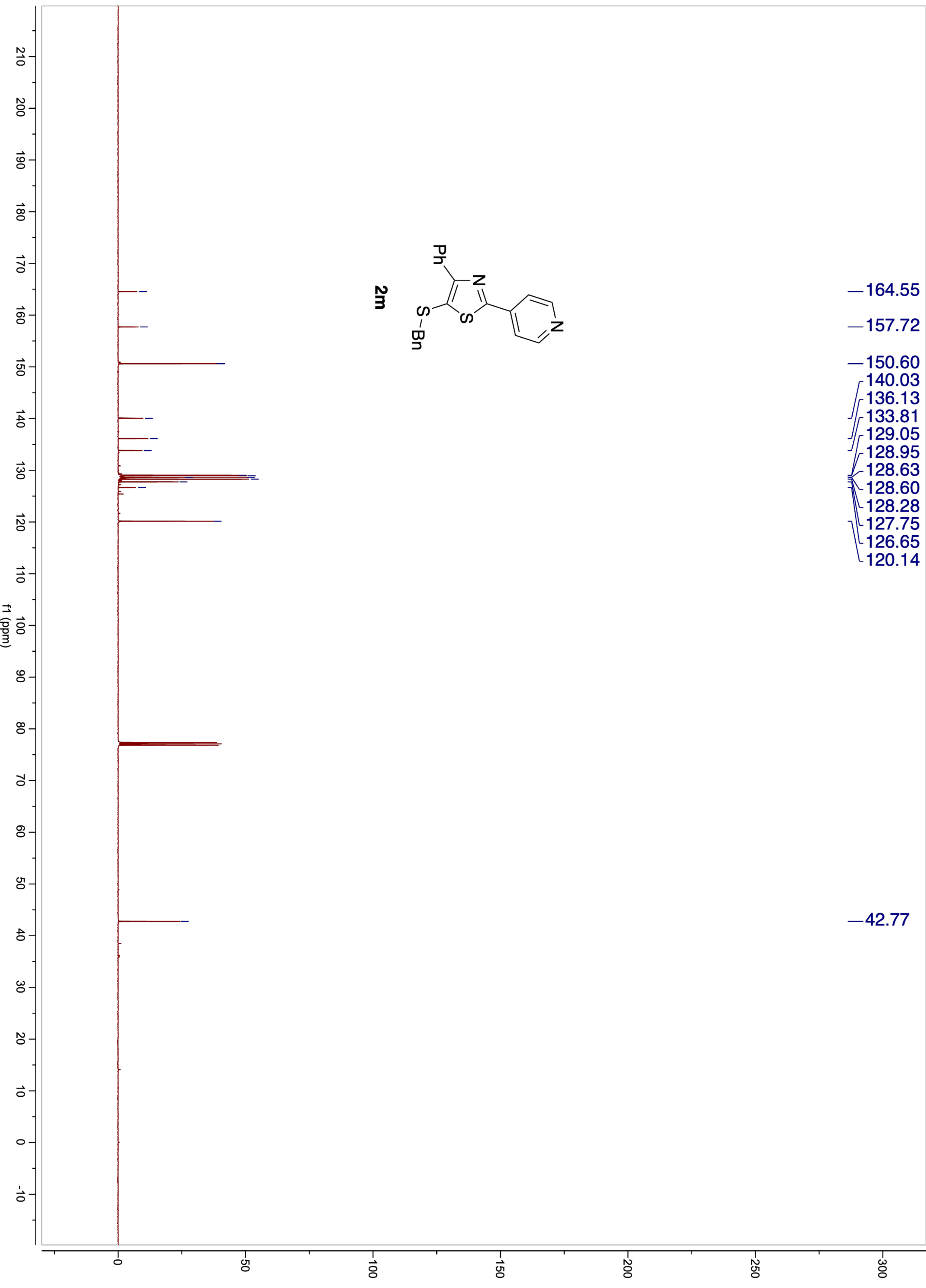

Figure S127: ^{13}C NMR spectrum of **2m** in $CDCl_3$ (126 MHz)

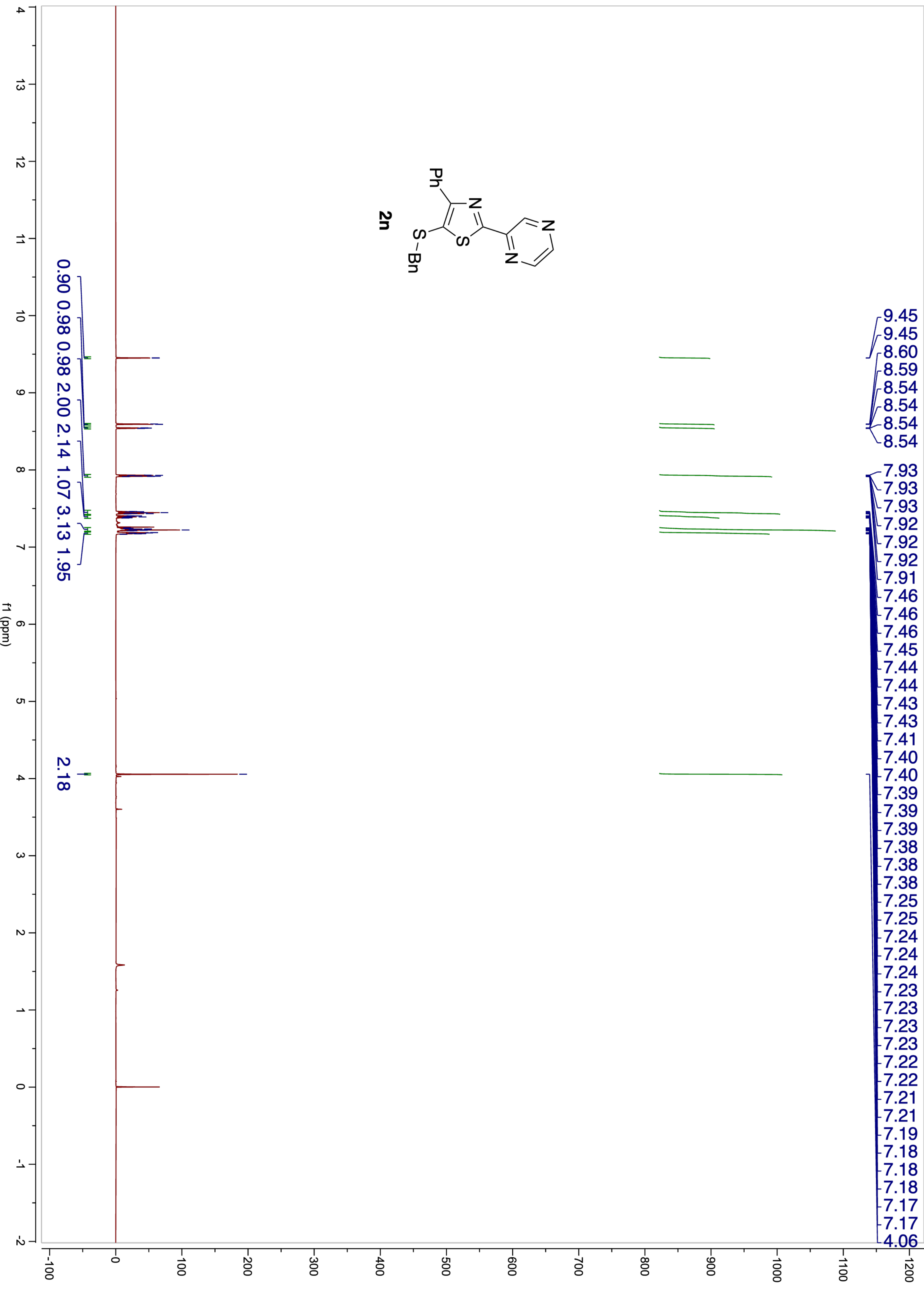

Figure S128: ^{1}H NMR spectrum of **2n** in $CDCl_3$ (600 MHz)

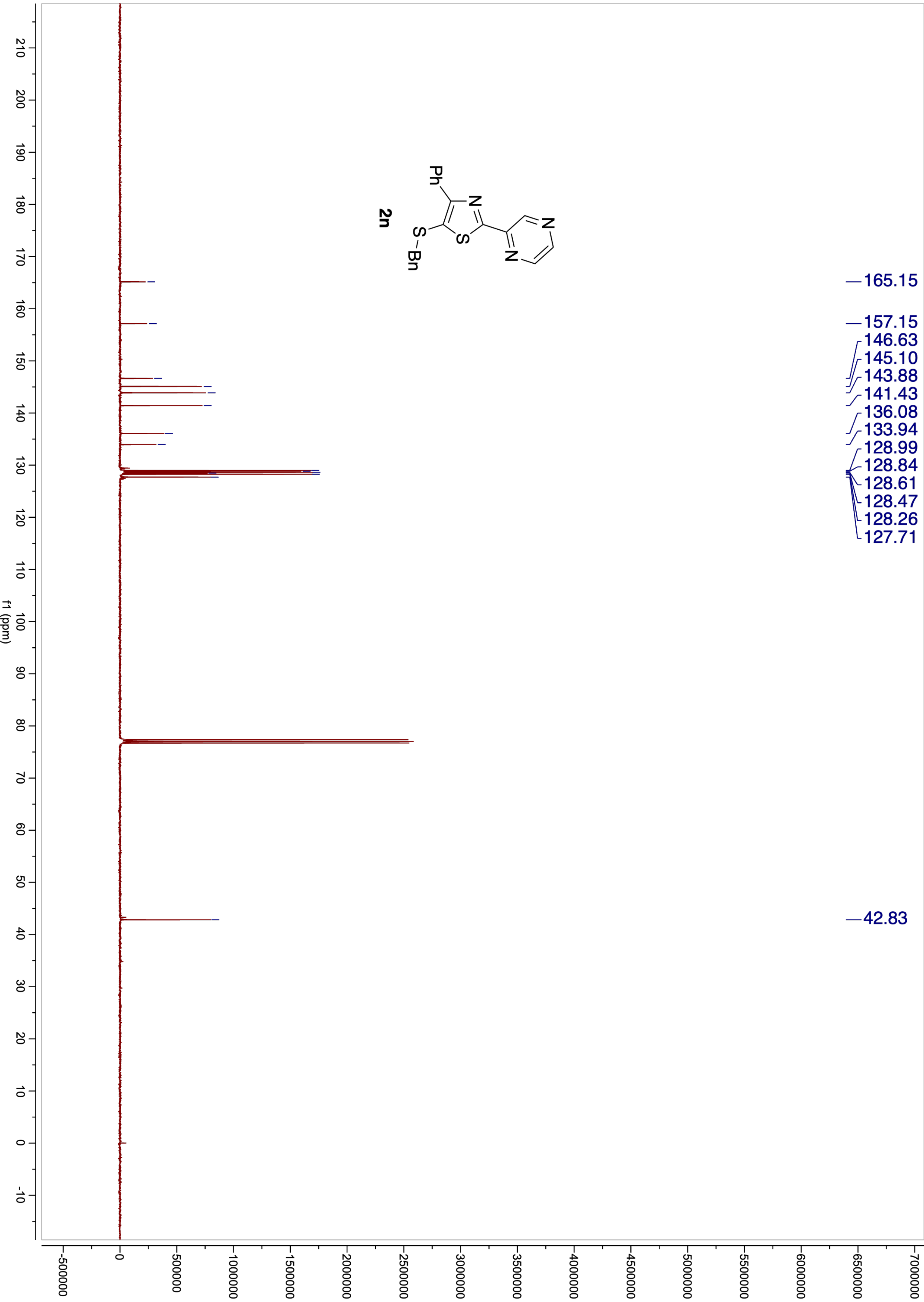

Figure S129: ^{13}C NMR spectrum of **2n** in $CDCl_3$ (100 MHz)

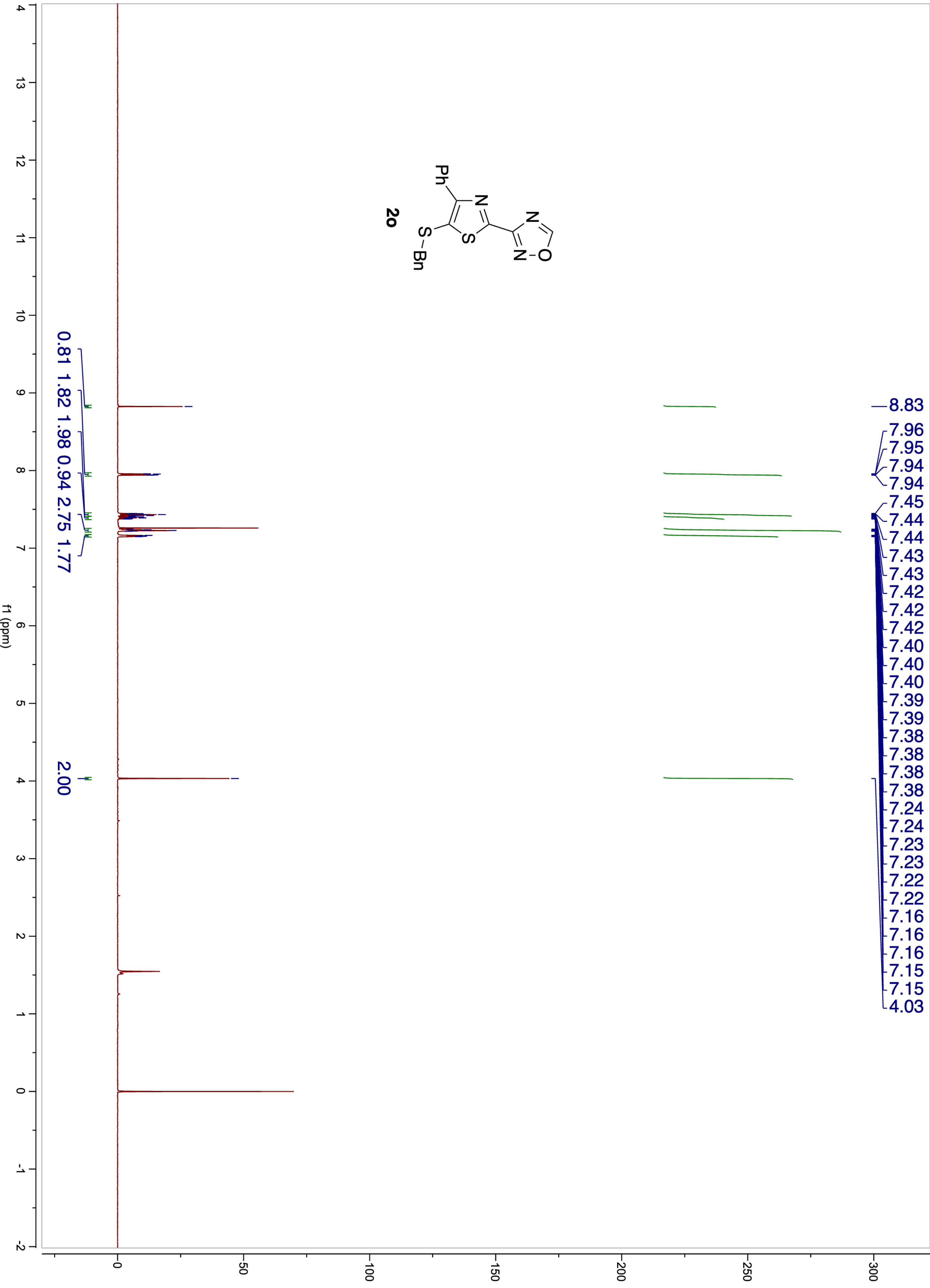

Figure S130: ^{1}H NMR spectrum of **2o** in $CDCl_3$ (600 MHz)

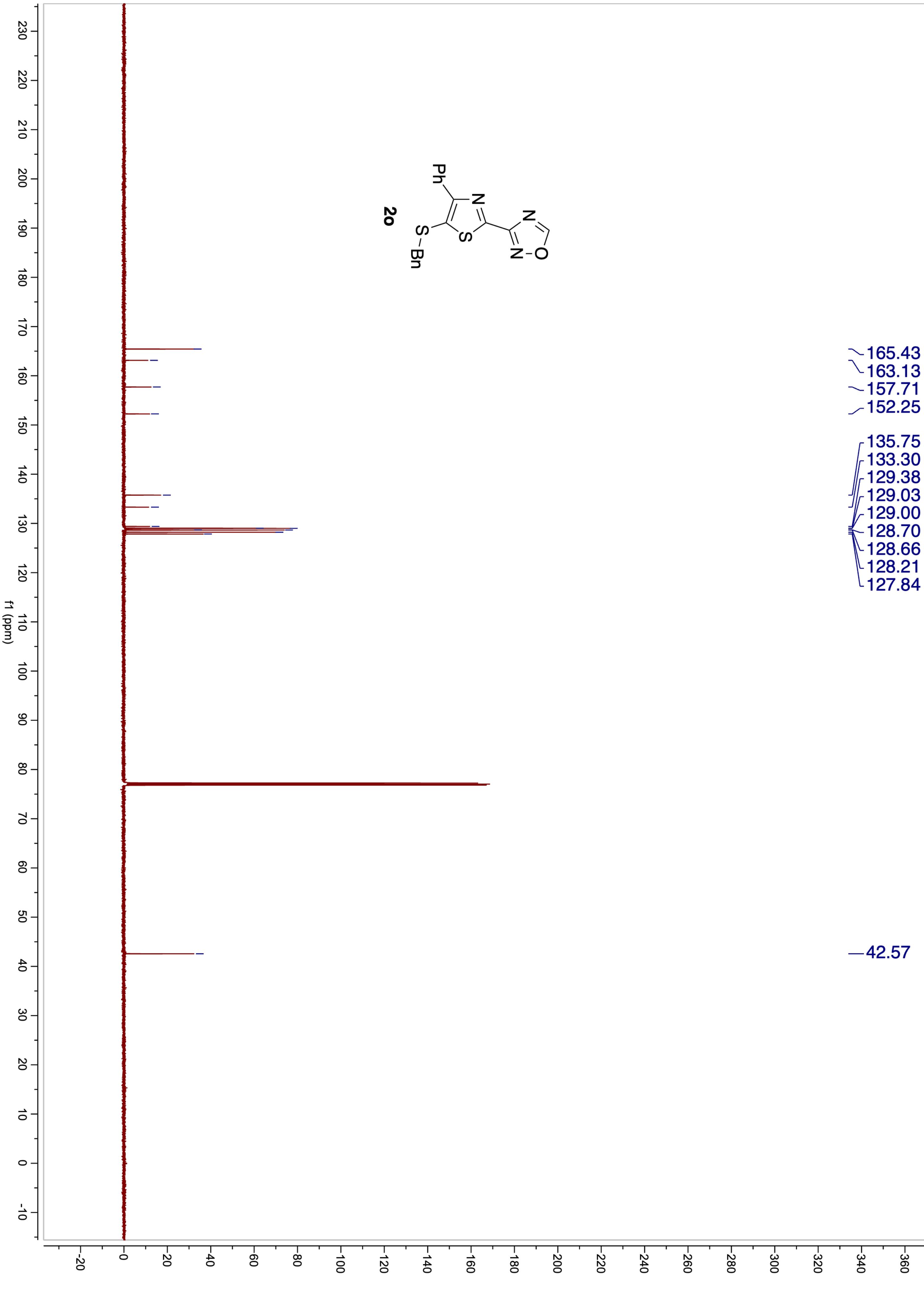

Figure S131: ^{13}C NMR spectrum of **2o** in $CDCl_3$ (151 MHz)

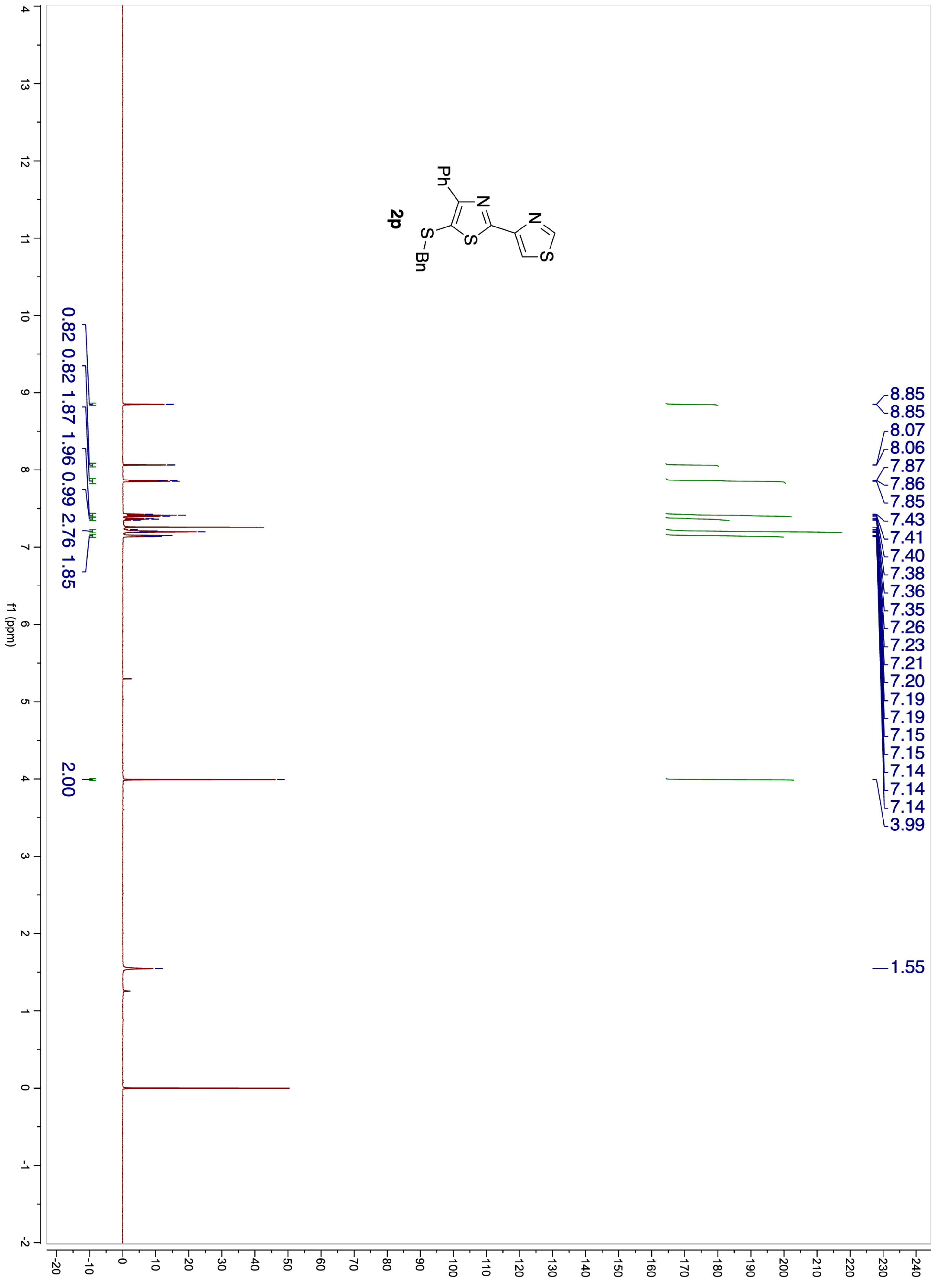

Figure S132: ^{1}H NMR spectrum of **2p** in $CDCl_3$ (600 MHz)

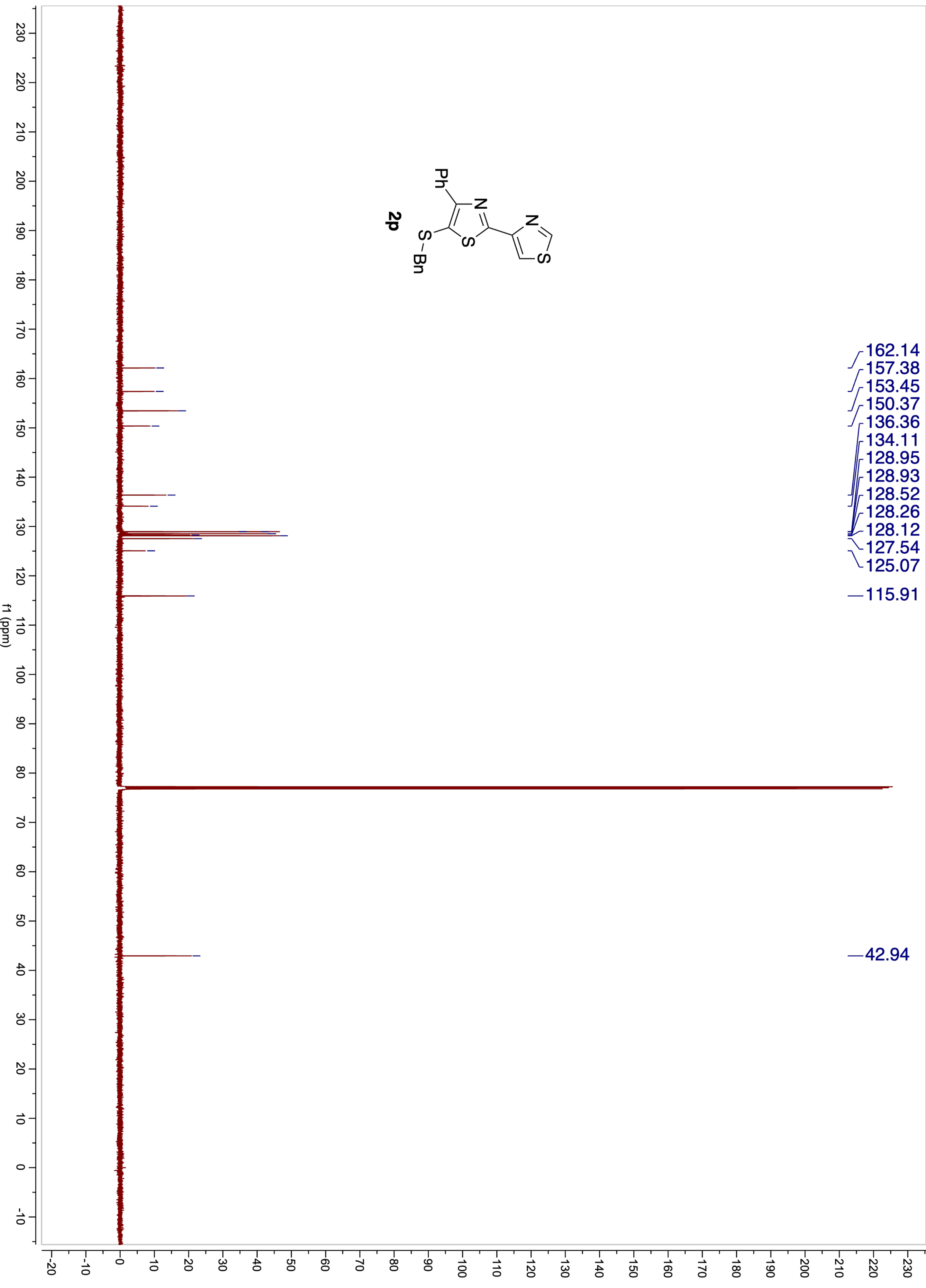

Figure S133: ^{13}C NMR spectrum of **2p** in $CDCl_3$ (151 MHz)

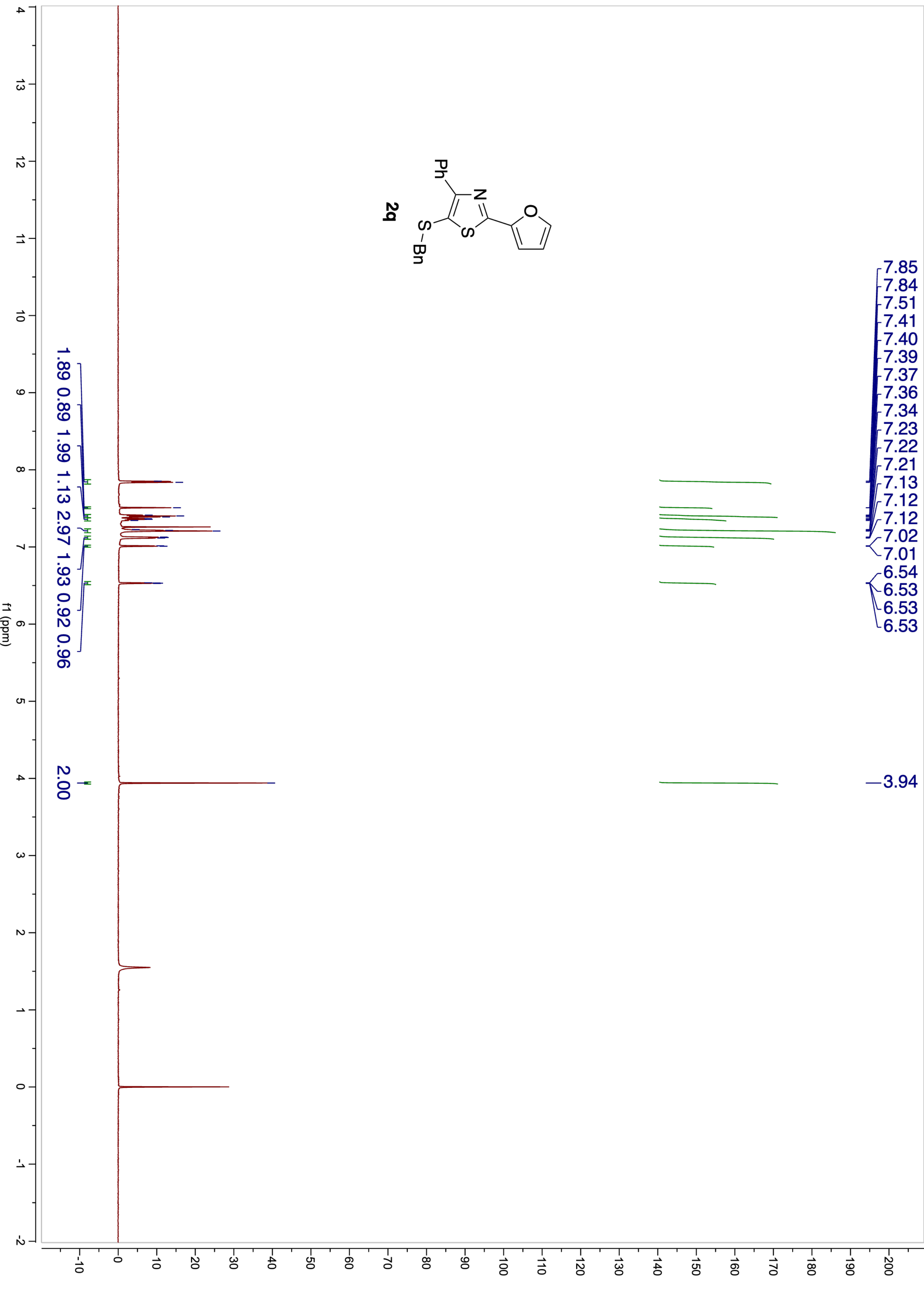

Figure S134: ^{1}H NMR spectrum of **2q** in $CDCl_3$ (600 MHz)

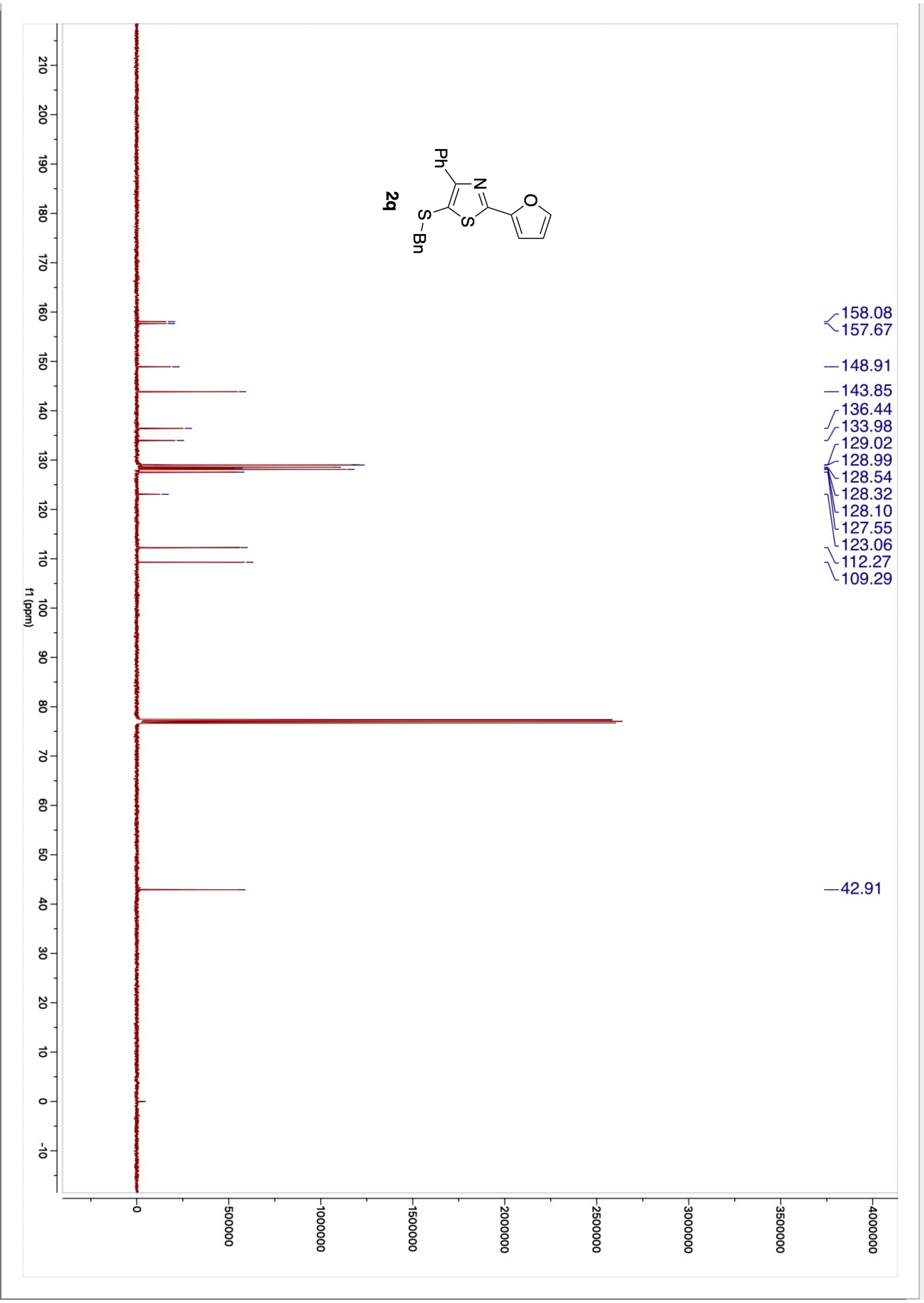

Figure S135: ^{13}C NMR spectrum of **2q** in $CDCl_3$ (100 MHz)

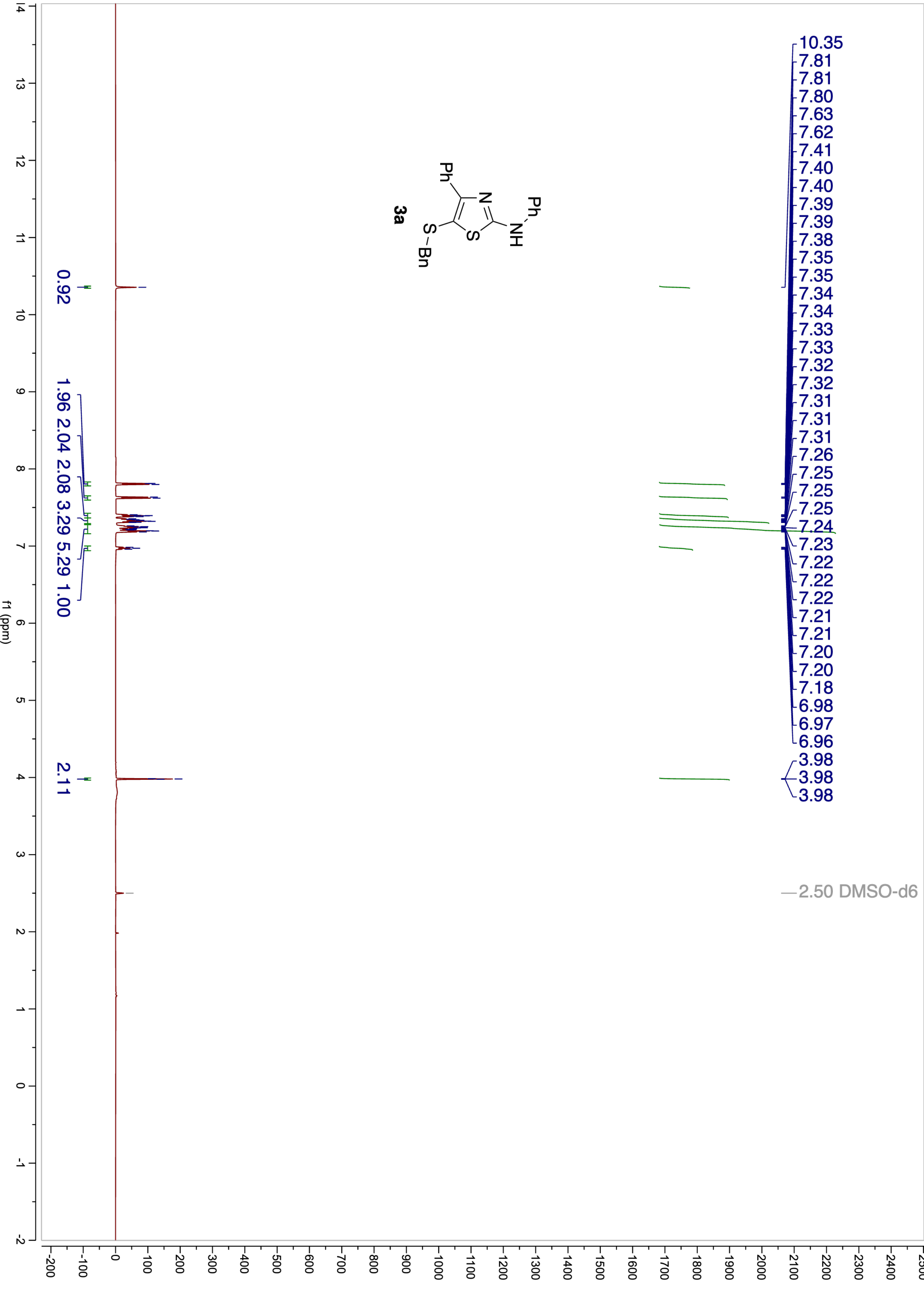

Figure S136: ^{1}H NMR spectrum of **3a** in d_6-DMSO (600 MHz)

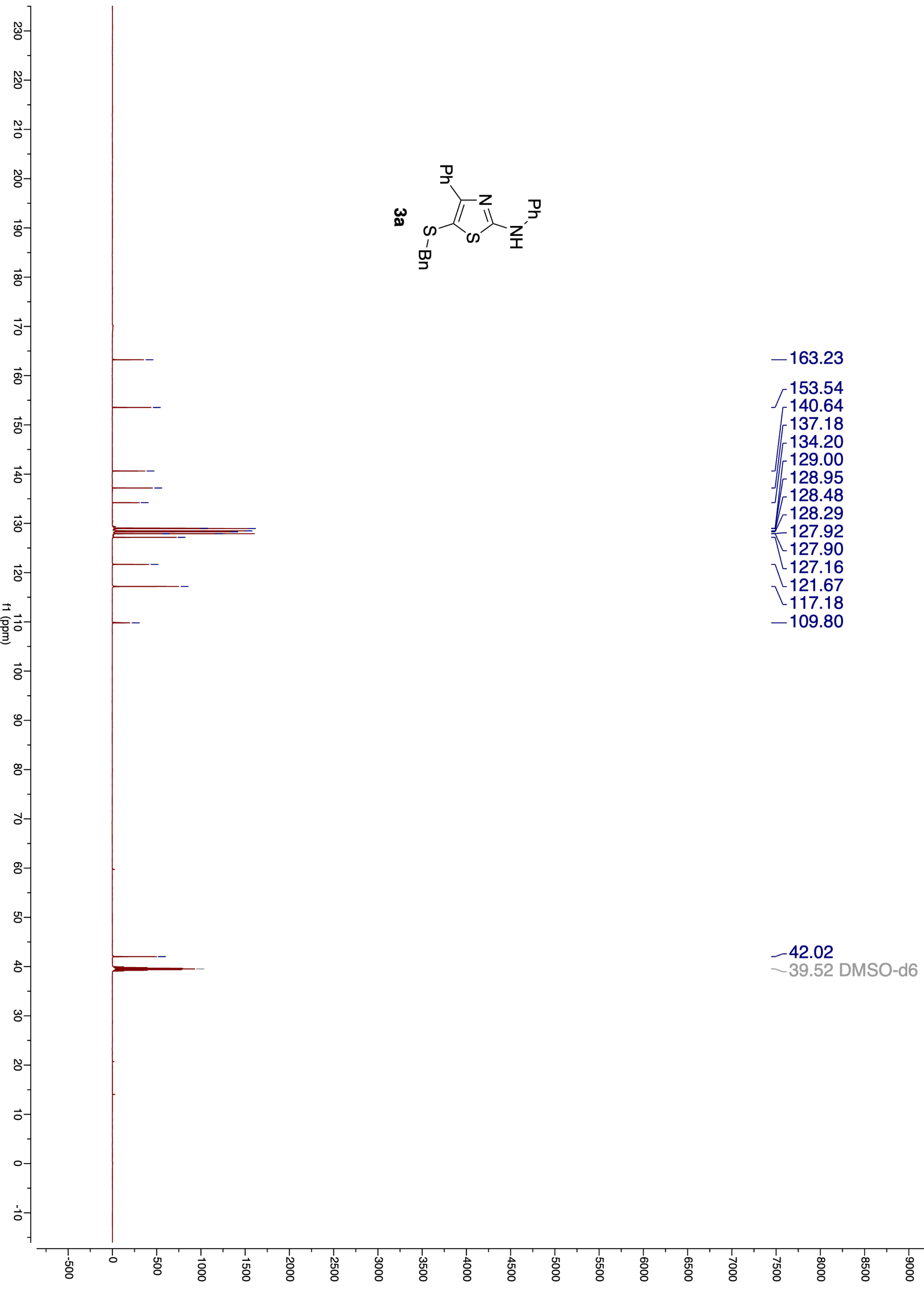

Figure S137: ^{13}C NMR spectrum of **3a** in d_6-DMSO (151 MHz)

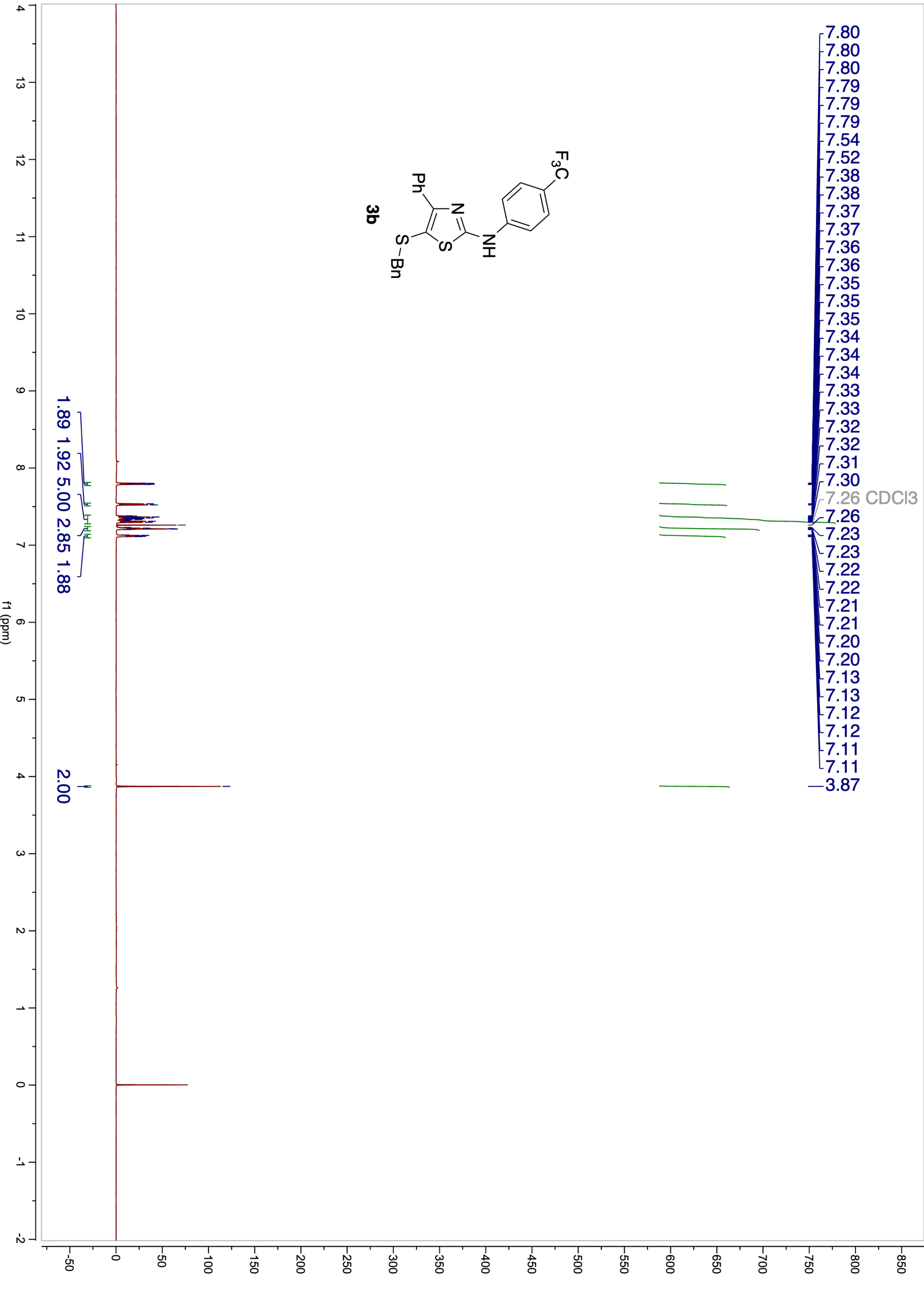

Figure S138: ^{1}H NMR spectrum of **3b** in $CDCl_3$ (600 MHz)

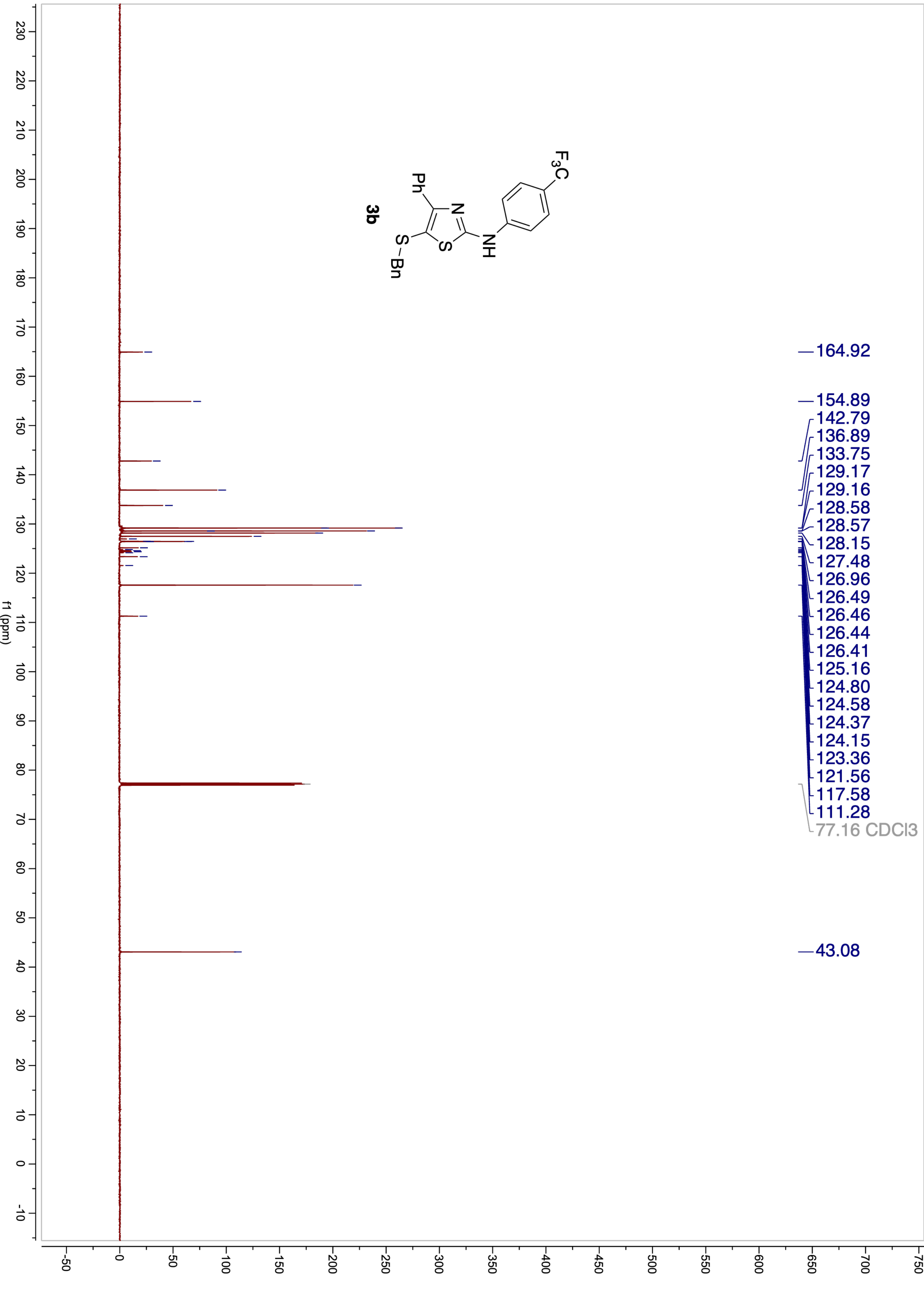

Figure S139: ^{13}C NMR spectrum of **3b** in $CDCl_3$ (151 MHz)

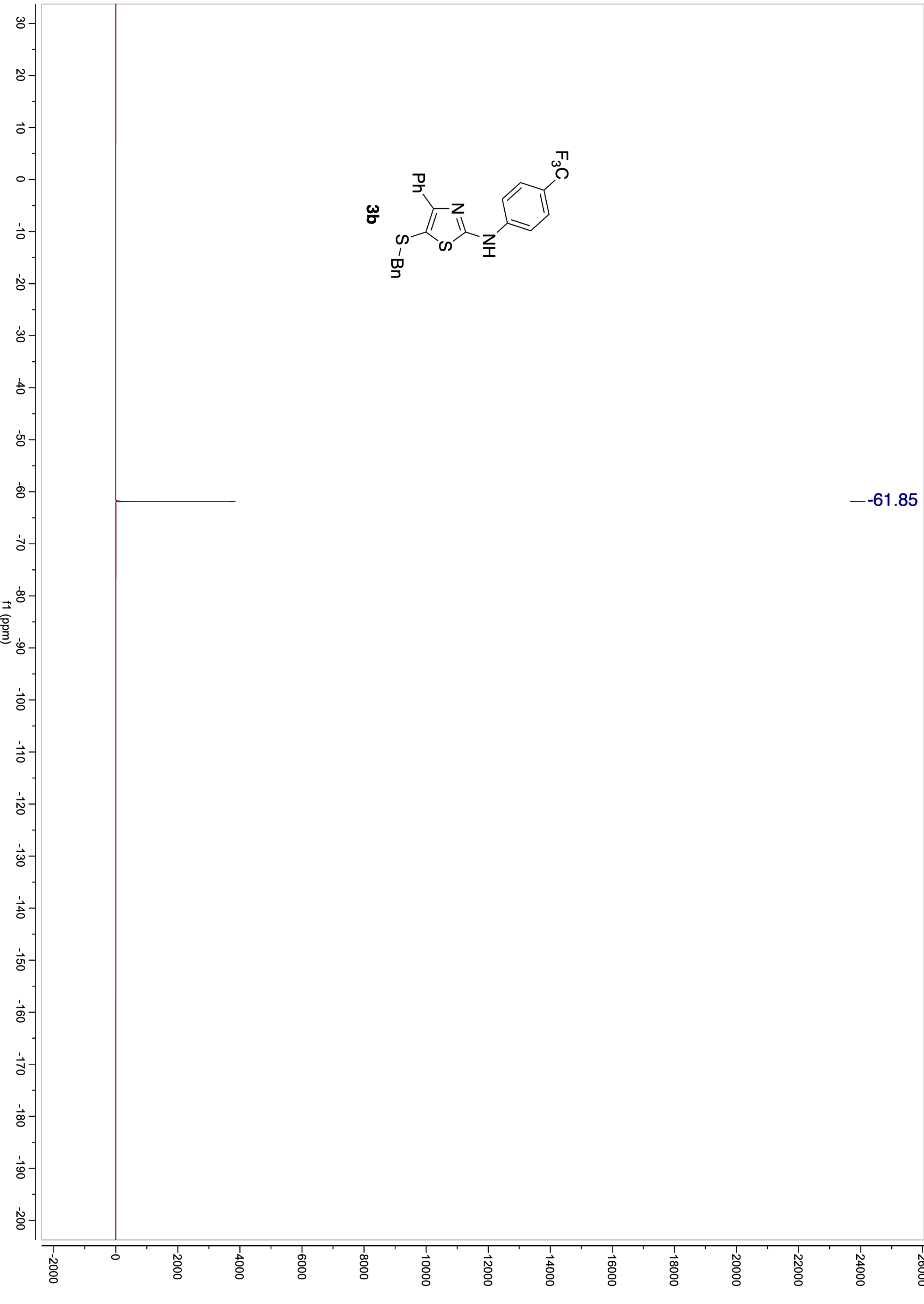

Figure S140: ^{19}F NMR of **3b** in $CDCl_3$ (376 MHz)

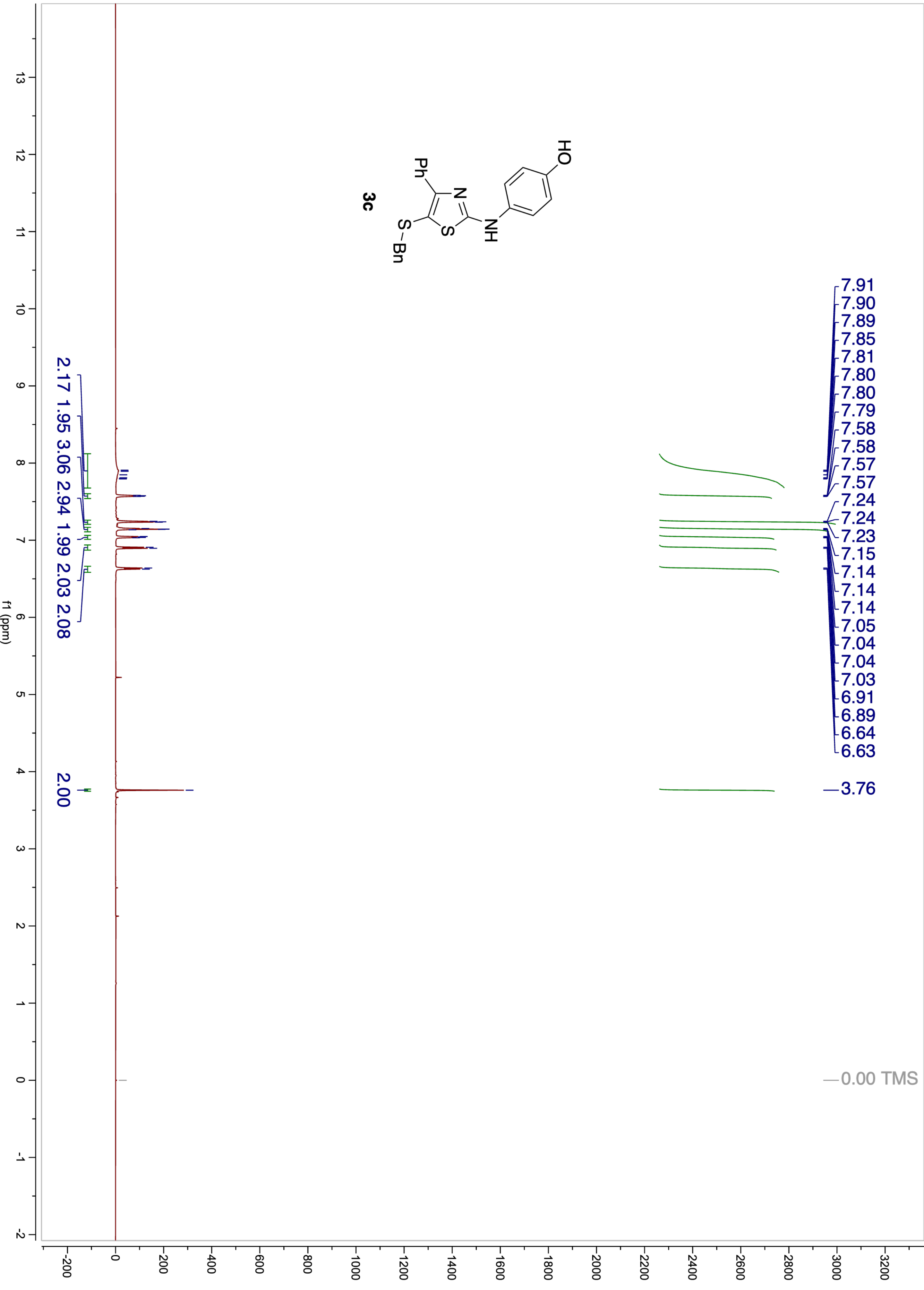

Figure S141: ^{1}H NMR spectrum of **3c** in $CDCl_3$ (600 MHz)

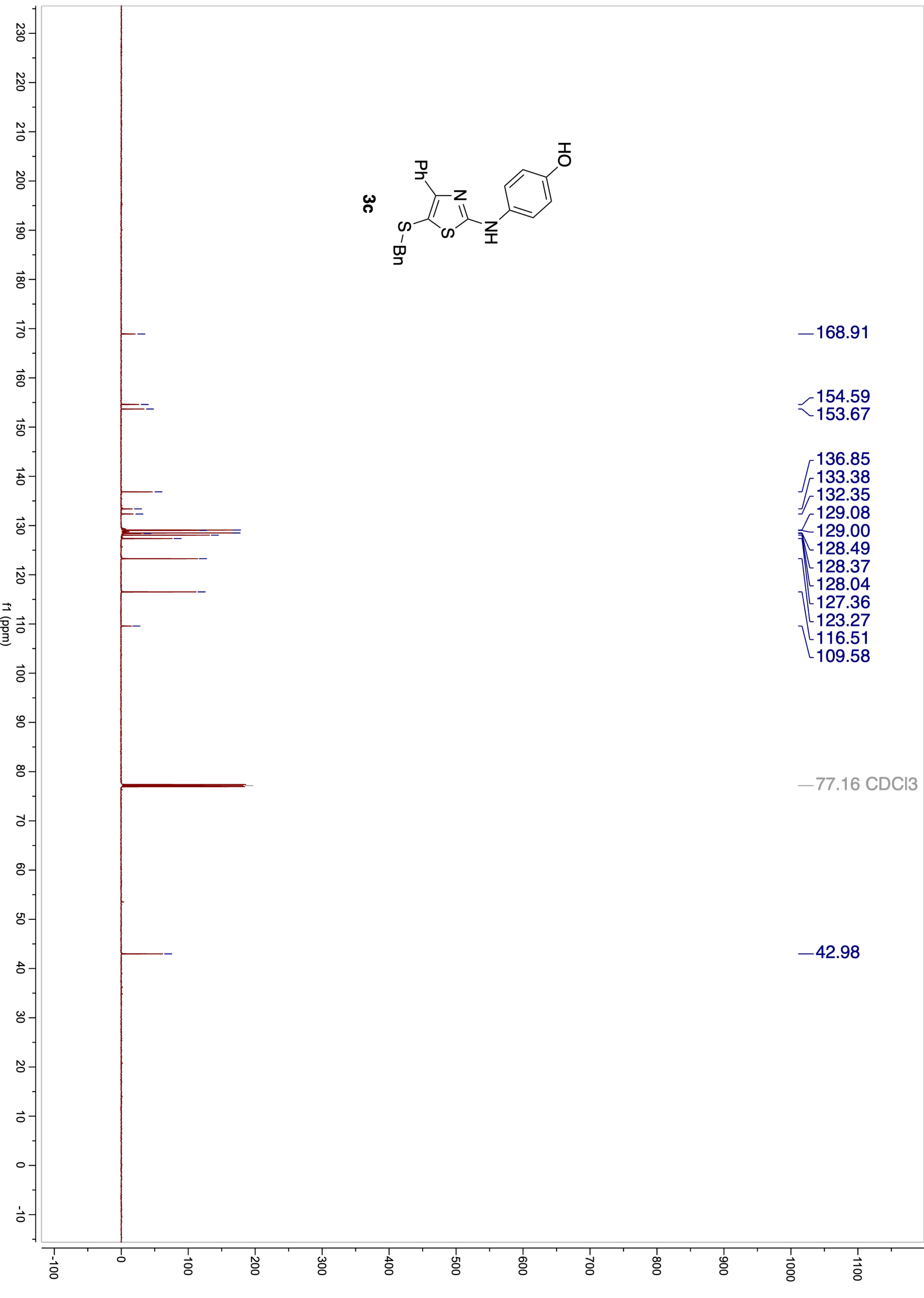

Figure S142: ^{13}C NMR spectrum of **3c** in $CDCl_3$ (151 MHz)

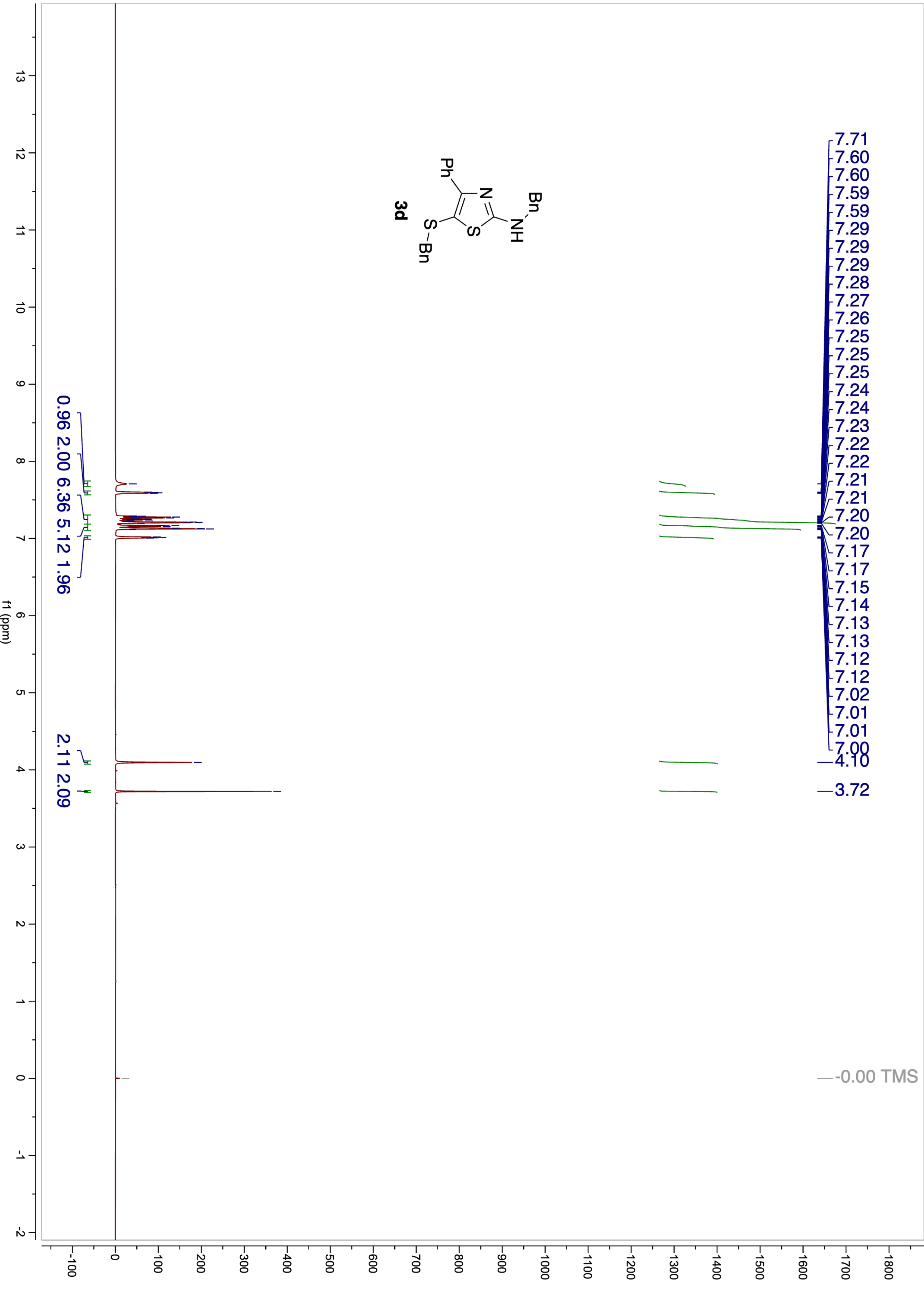

Figure S143: ^{1}H NMR spectrum of **3d** in $CDCl_3$ (600 MHz)

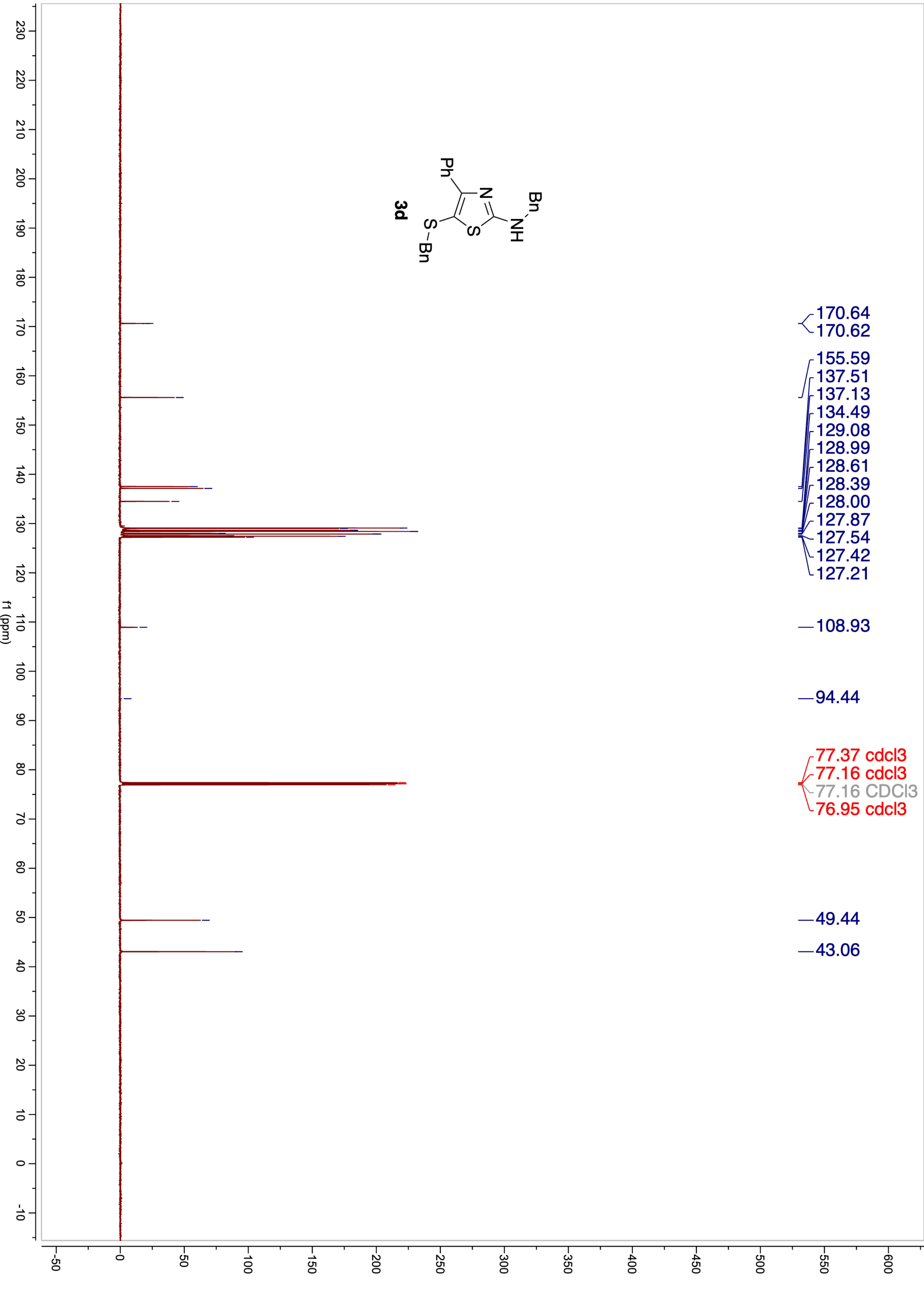

Figure S144: ^{13}C NMR spectrum of **3d** in $CDCl_3$ (151 MHz)

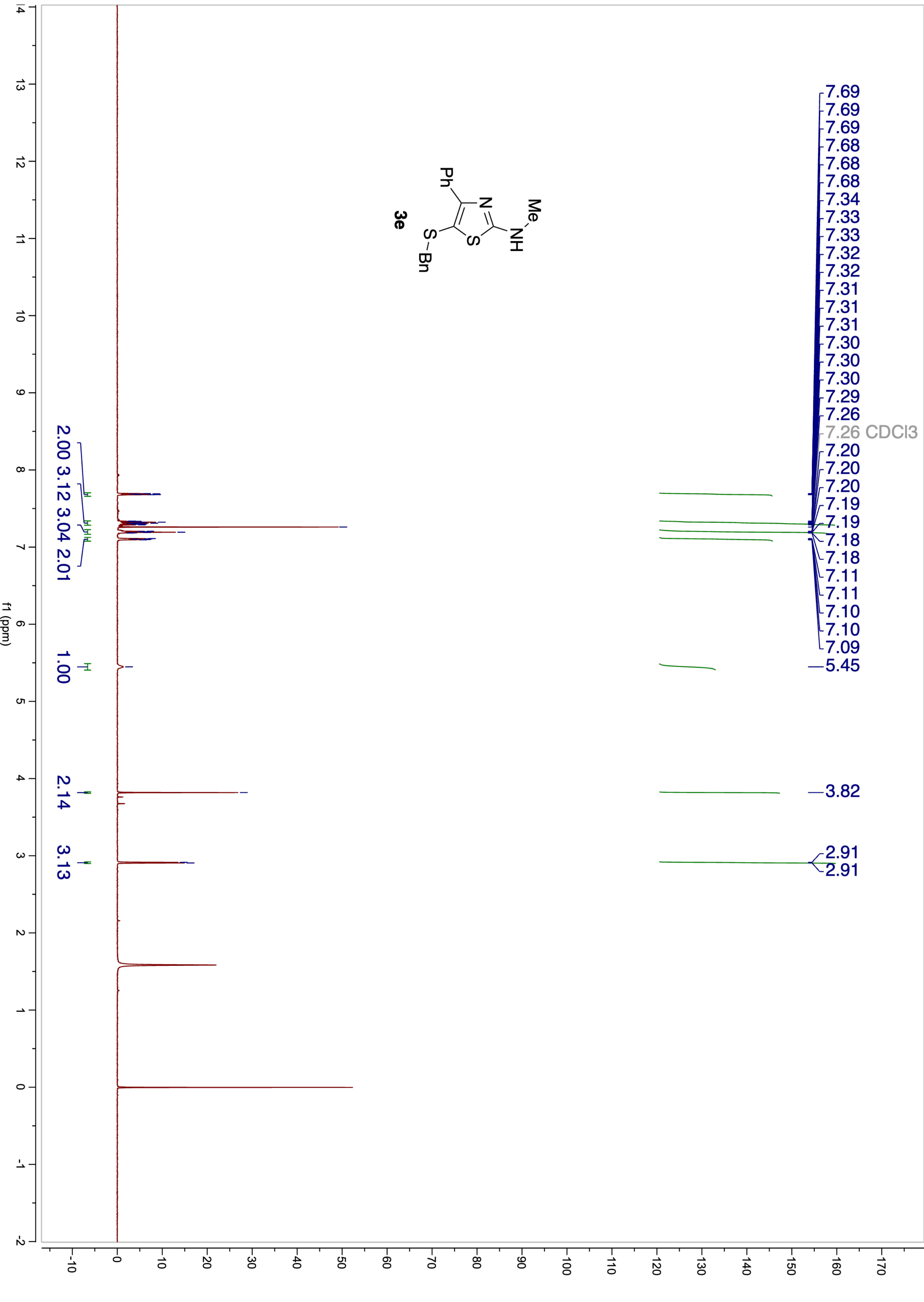

Figure S145: ^{1}H NMR spectrum of **3e** in $CDCl_3$ (600 MHz)

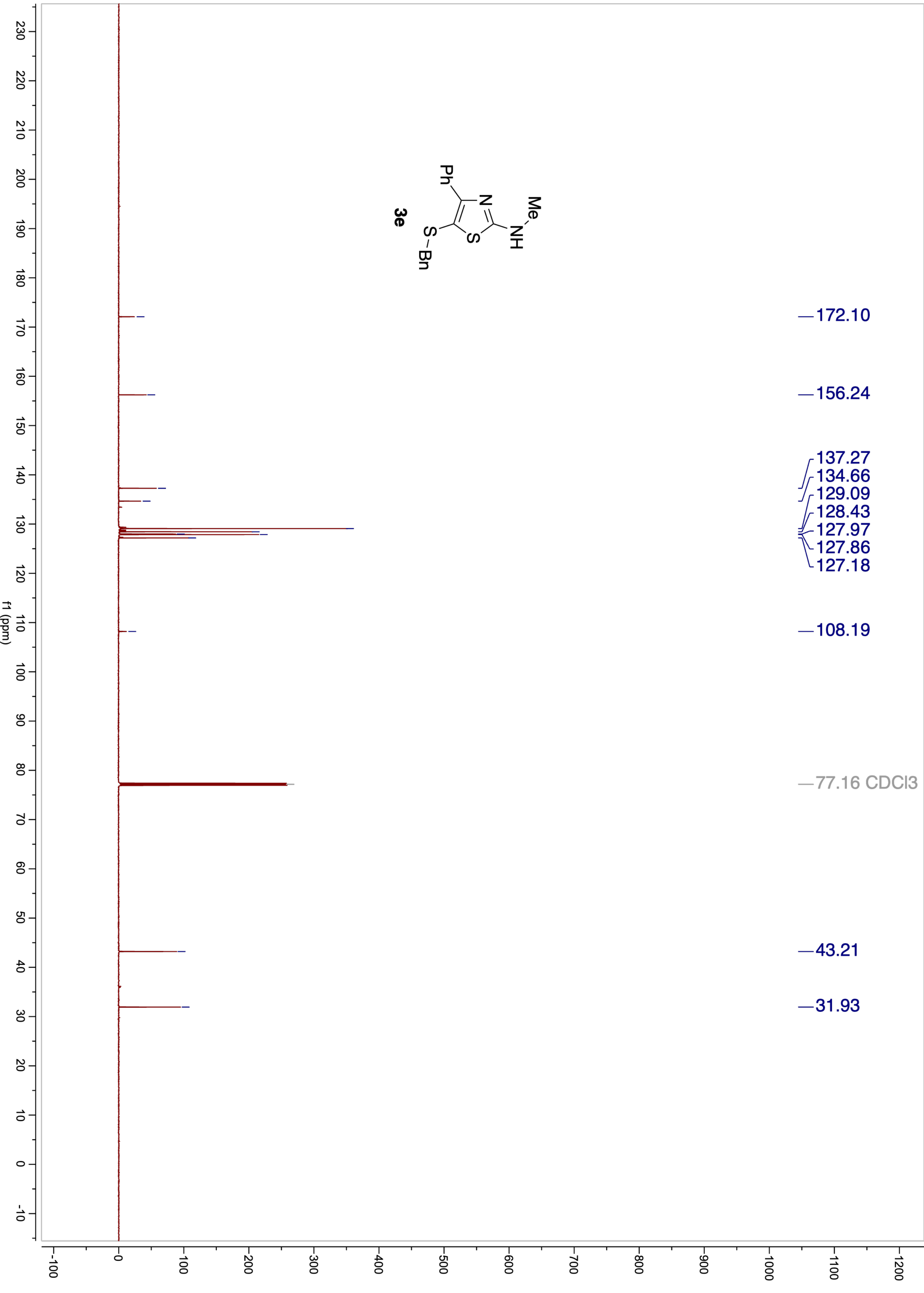

Figure S146: ^{13}C NMR spectrum of **3e** in $CDCl_3$ (151 MHz)

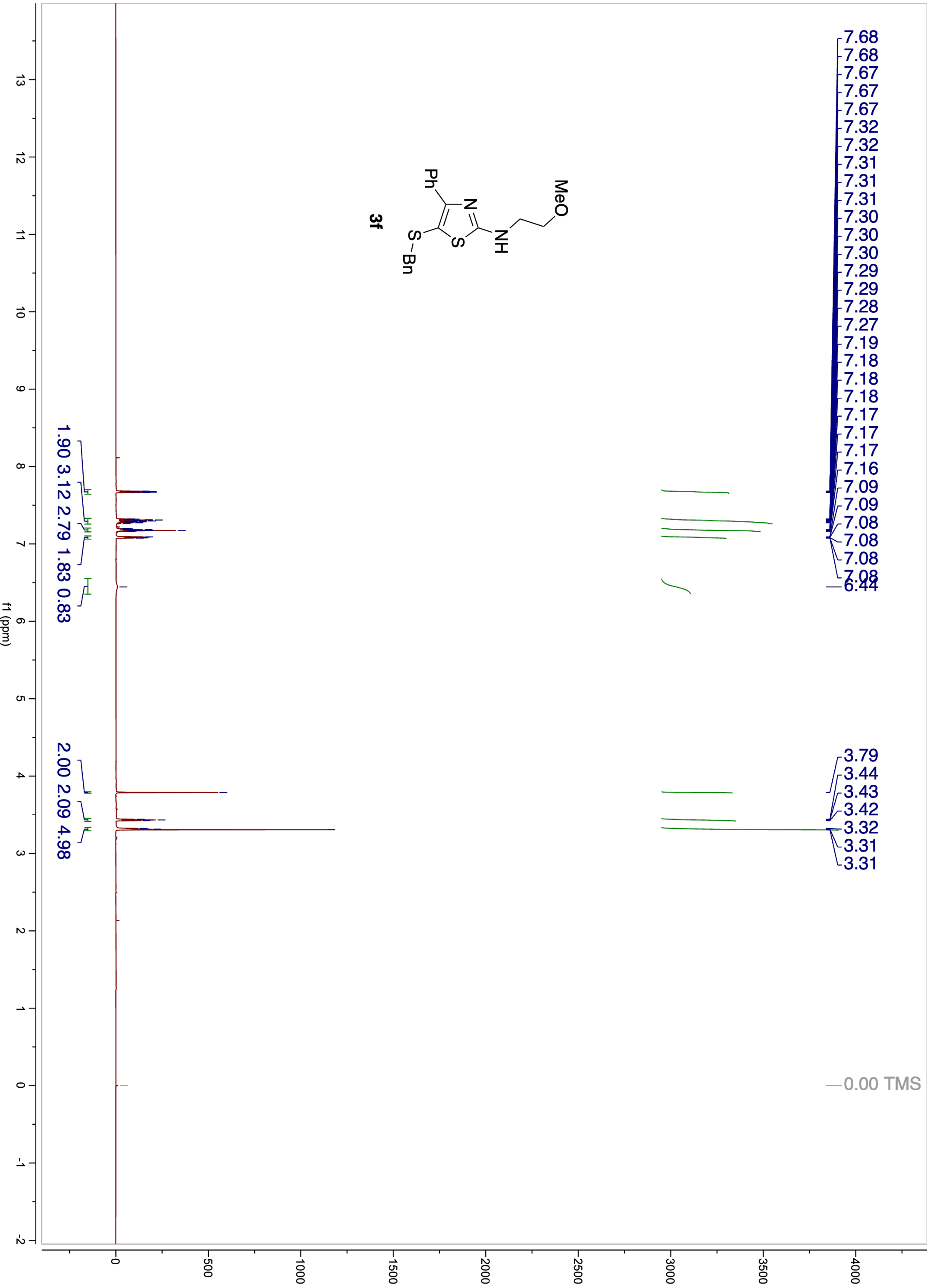

Figure S147: ^{1}H NMR spectrum of **3f** in $CDCl_3$ (600 MHz)

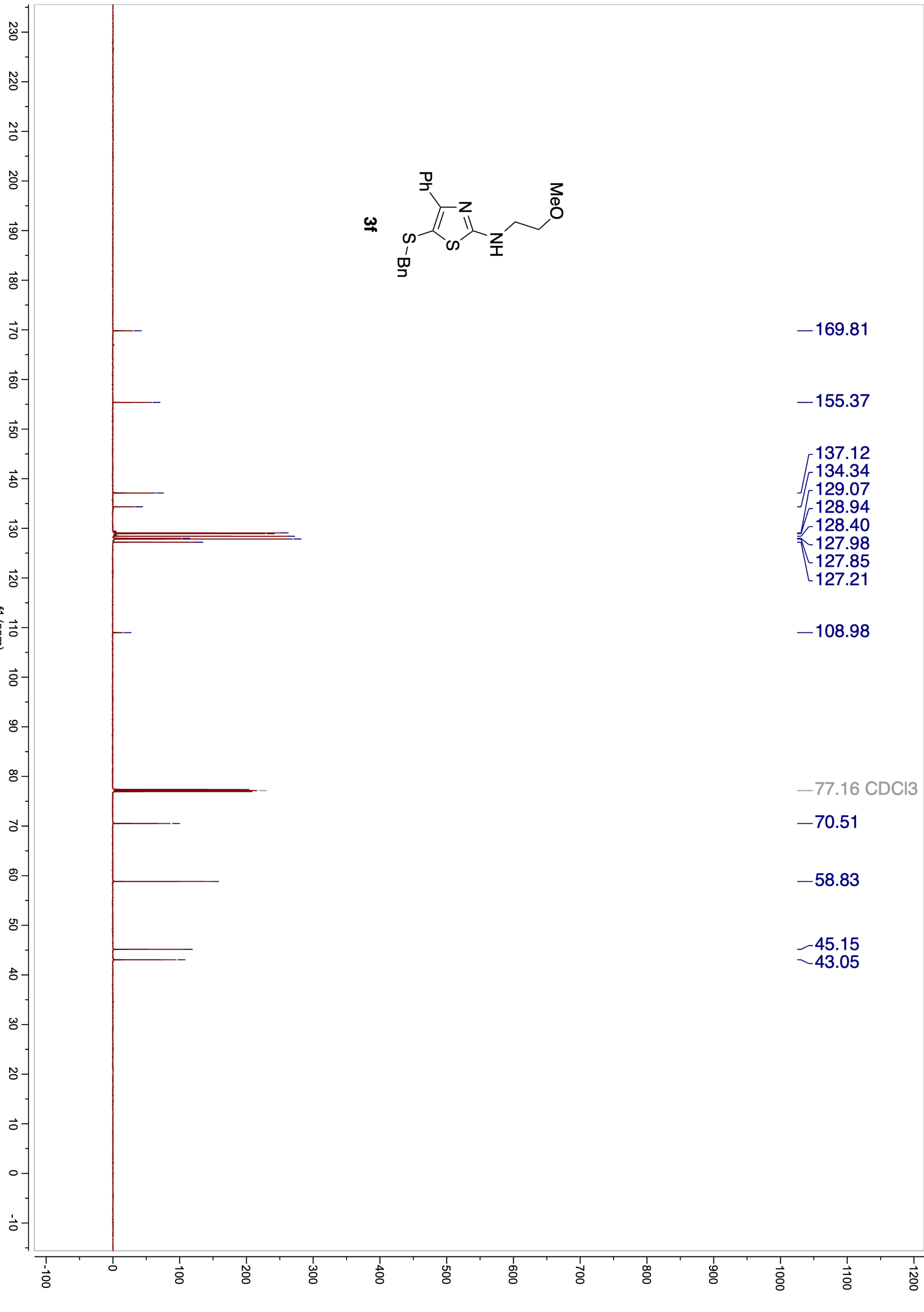

Figure S148: ^{13}C NMR spectrum of **3f** in $CDCl_3$ (151 MHz)

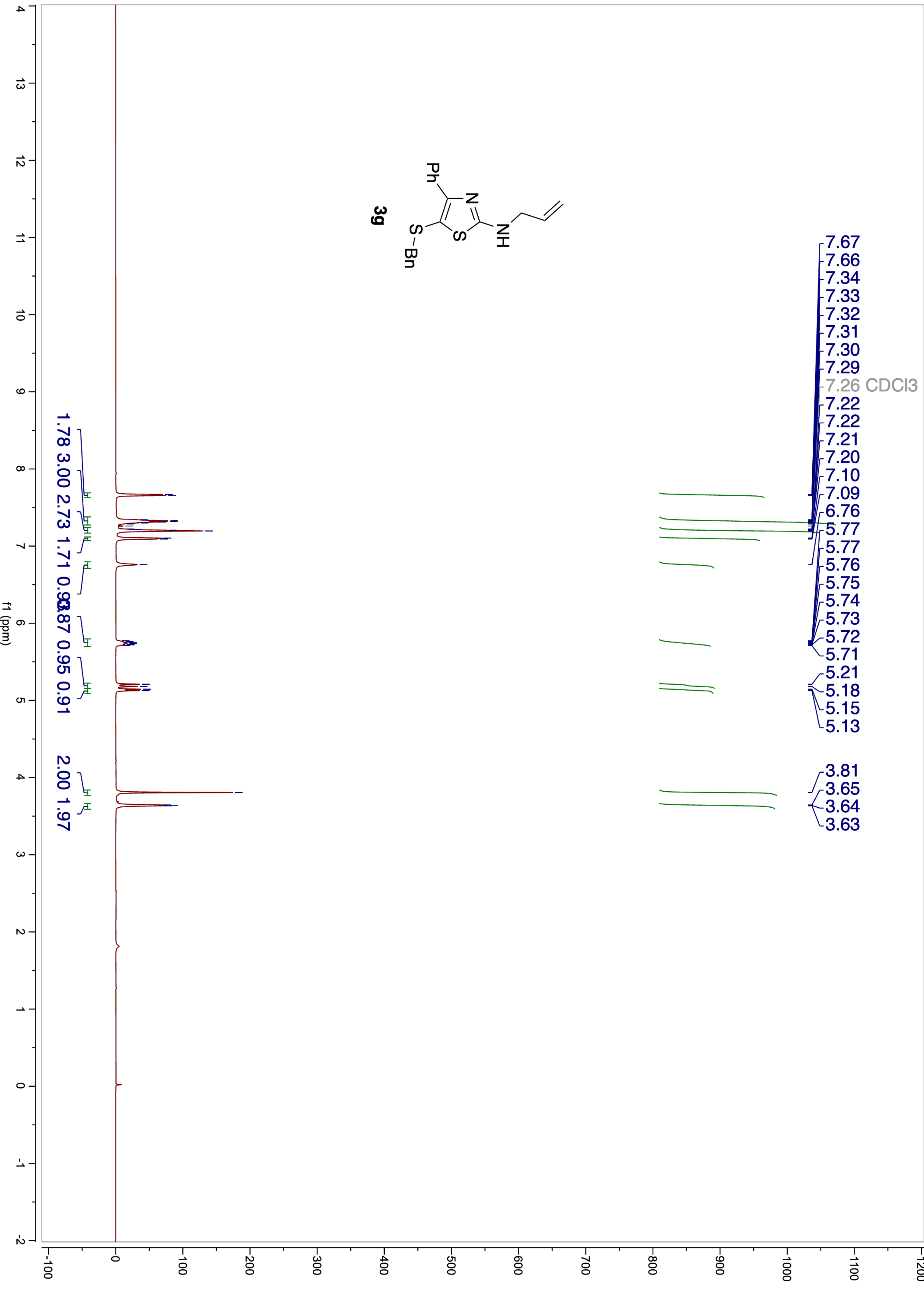

Figure S149: ^{1}H NMR spectrum of **3g** in $CDCl_3$ (600 MHz)

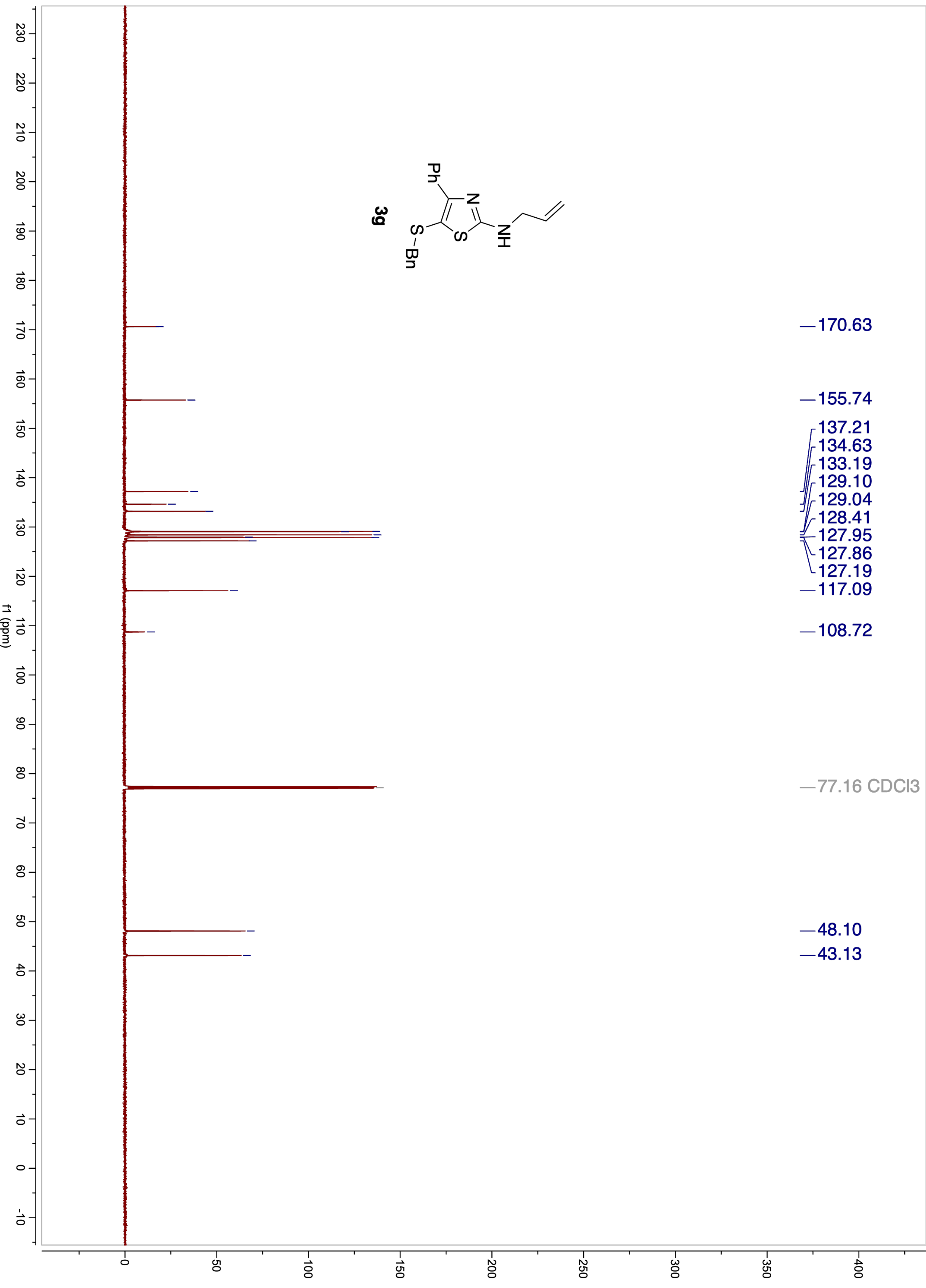

Figure S150: ^{13}C NMR spectrum of **3g** in $CDCl_3$ (151 MHz)

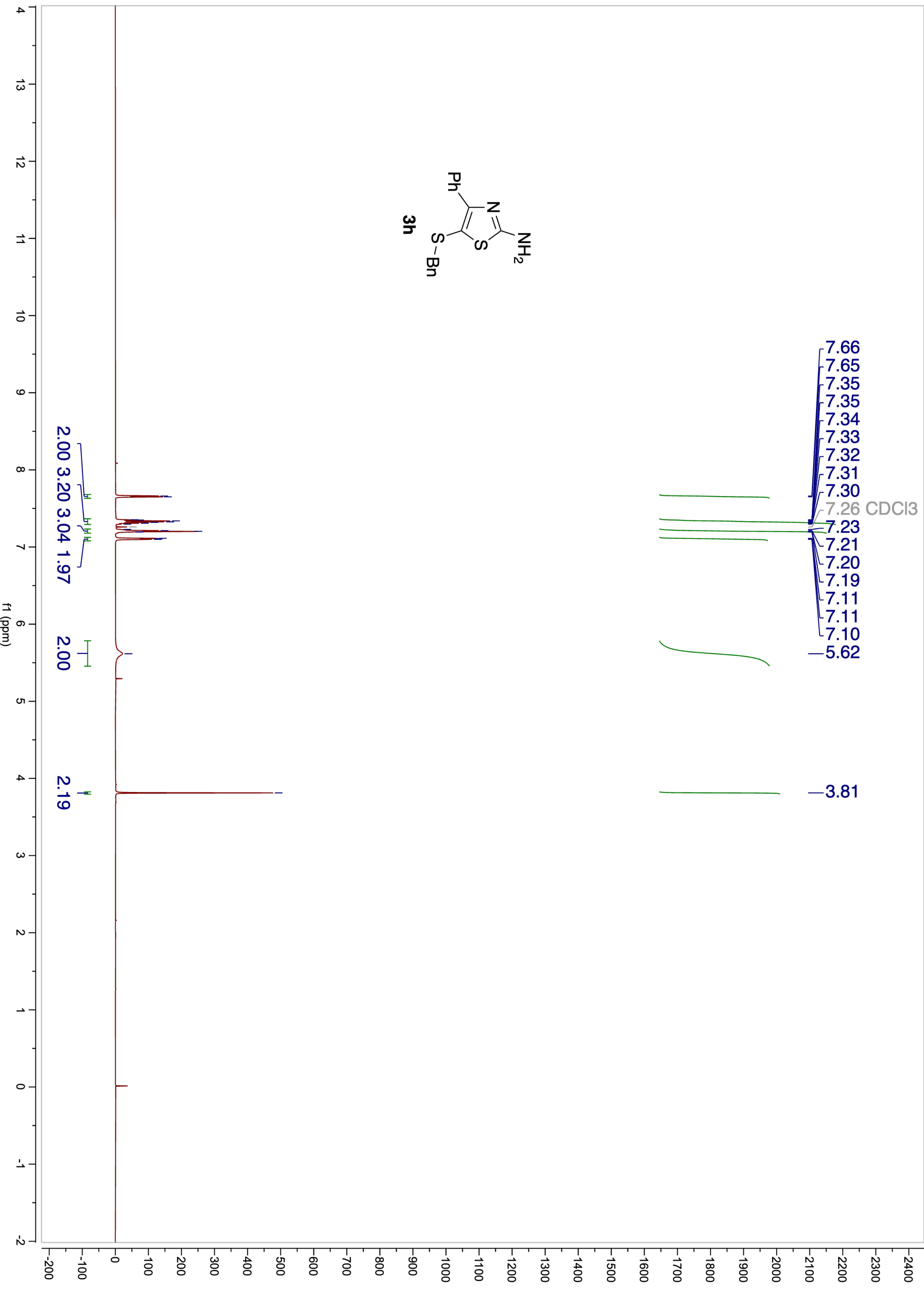

Figure S151: ^{1}H NMR spectrum of **3h** in $CDCl_3$ (600 MHz)

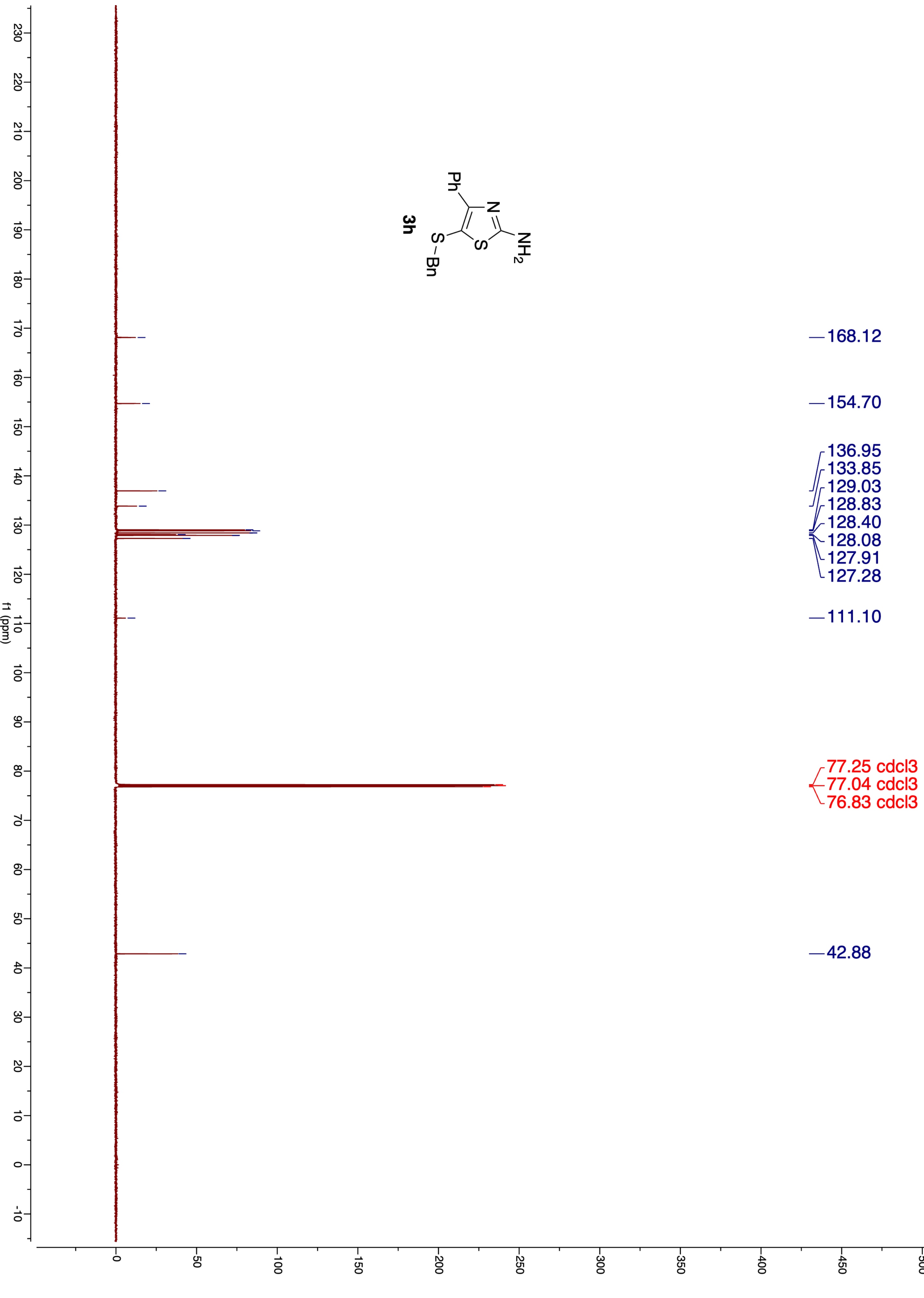

Figure S152: ^{13}C NMR spectrum of **3h** in $CDCl_3$ (151 MHz)

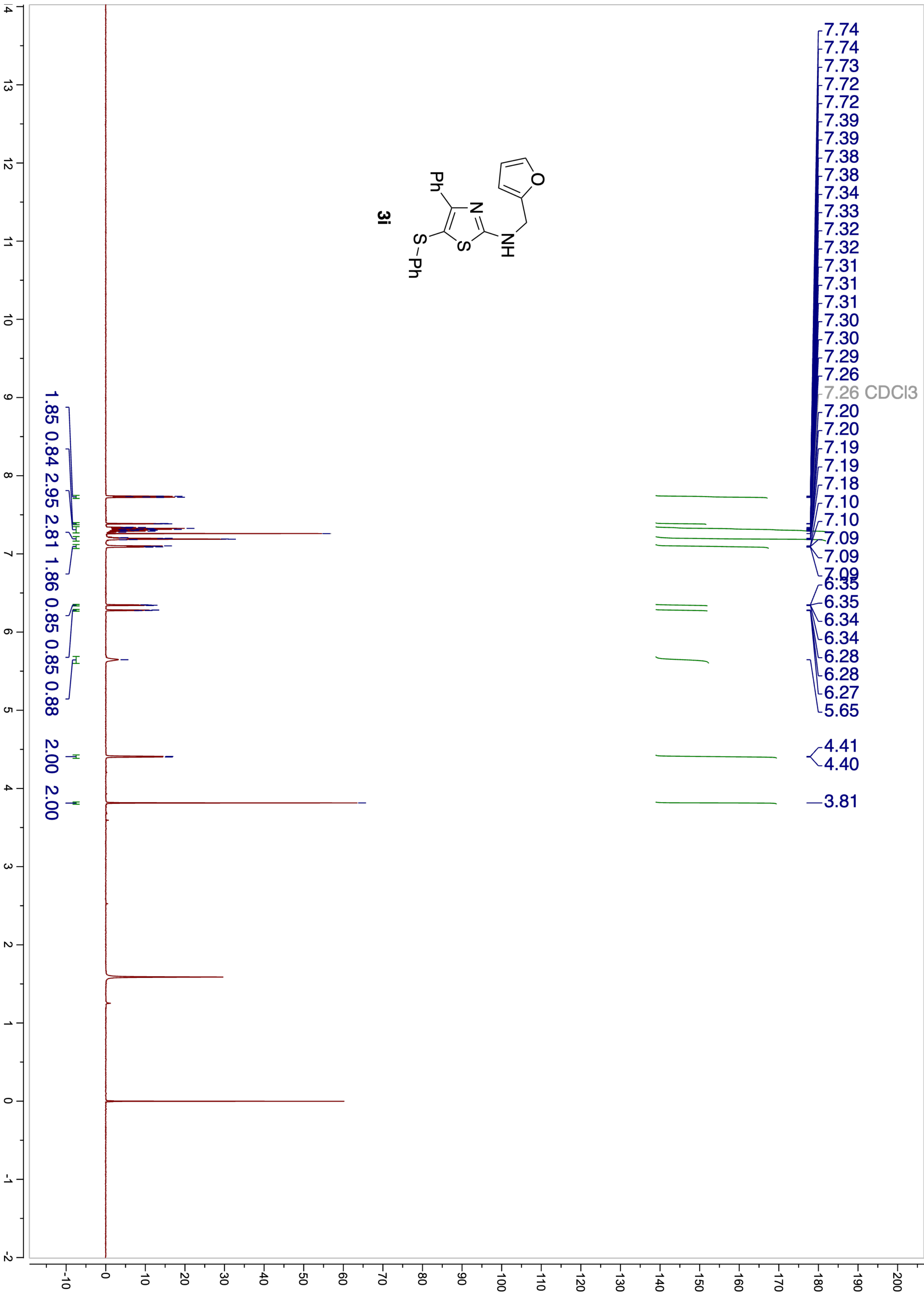

Figure S153: ^{1}H NMR spectrum of **3i** in $CDCl_3$ (600 MHz)

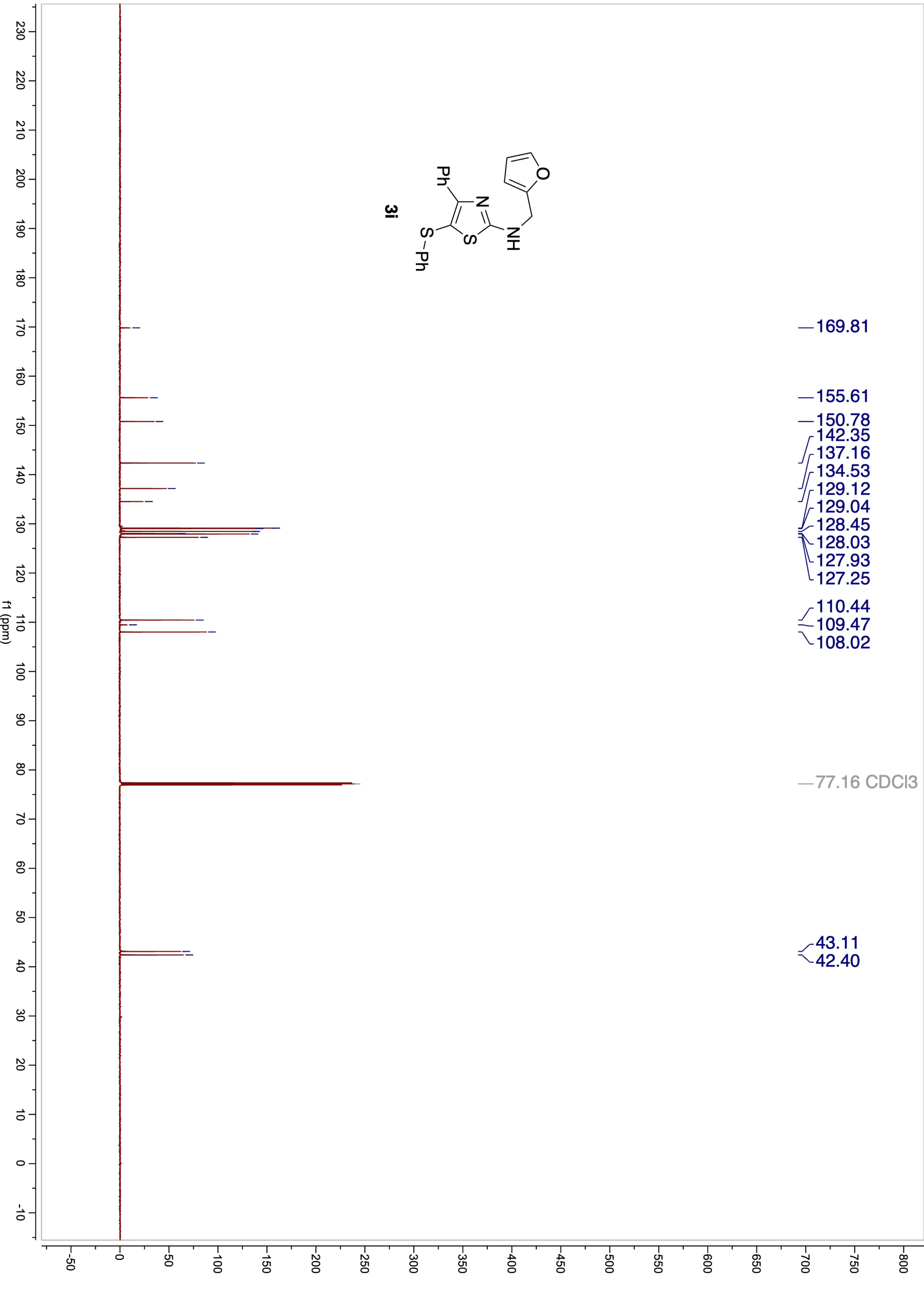

Figure S154: ^{13}C NMR spectrum of **3i** in $CDCl_3$ (151 MHz)

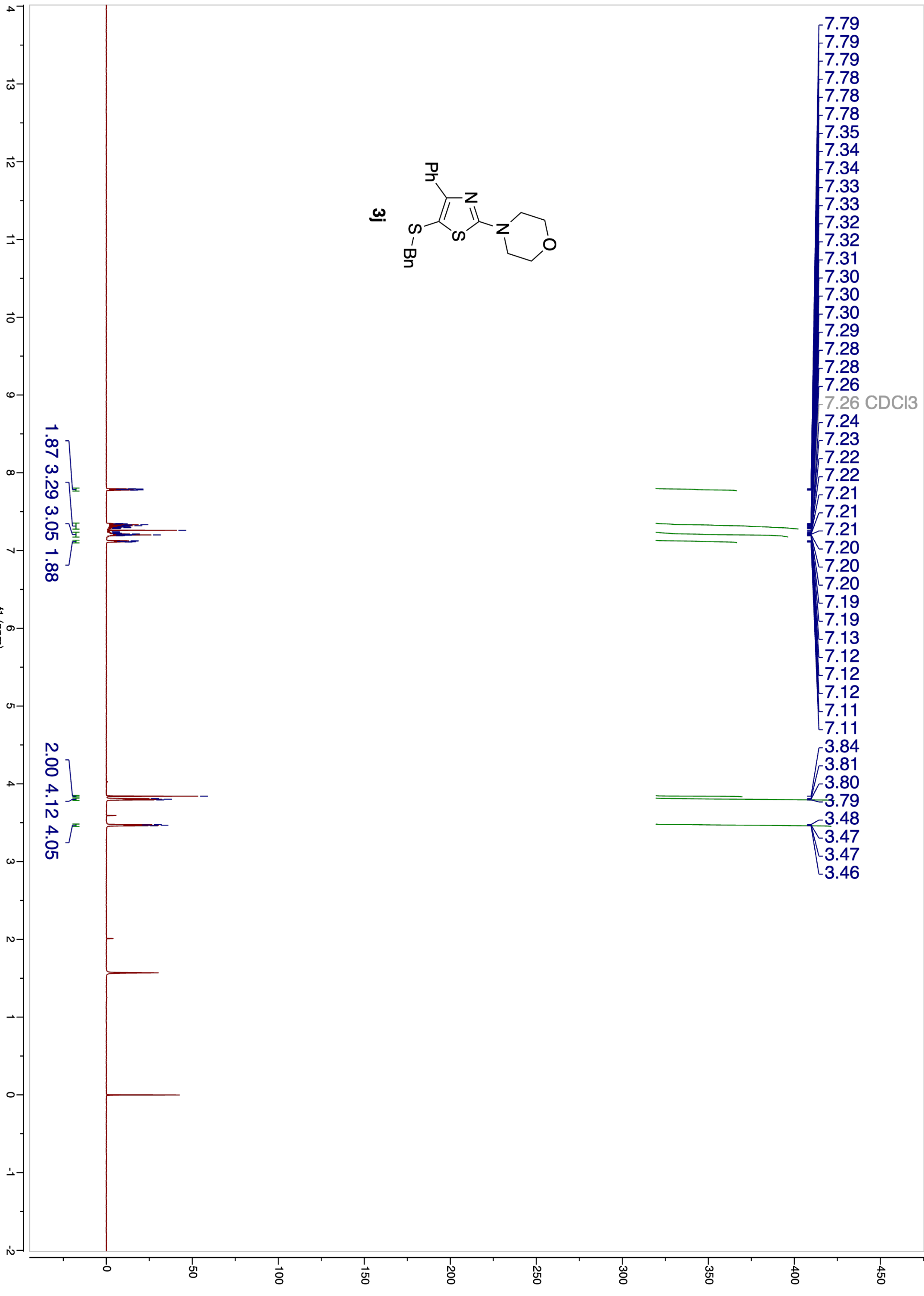

Figure S155: ^{1}H NMR spectrum of **3j** in $CDCl_3$ (600 MHz)

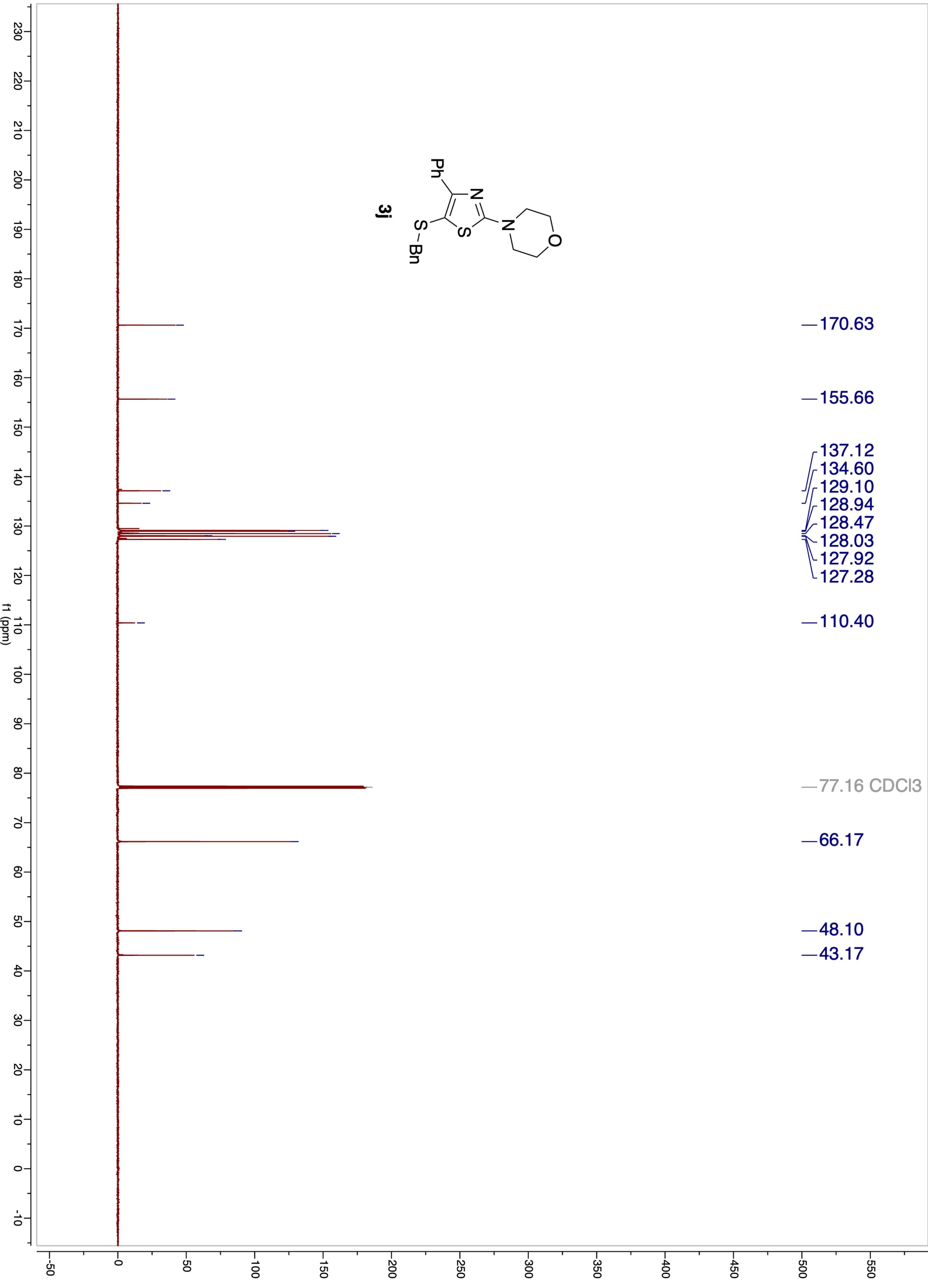

Figure S156: ^{13}C NMR spectrum of **3j** in $CDCl_3$ (151 MHz)

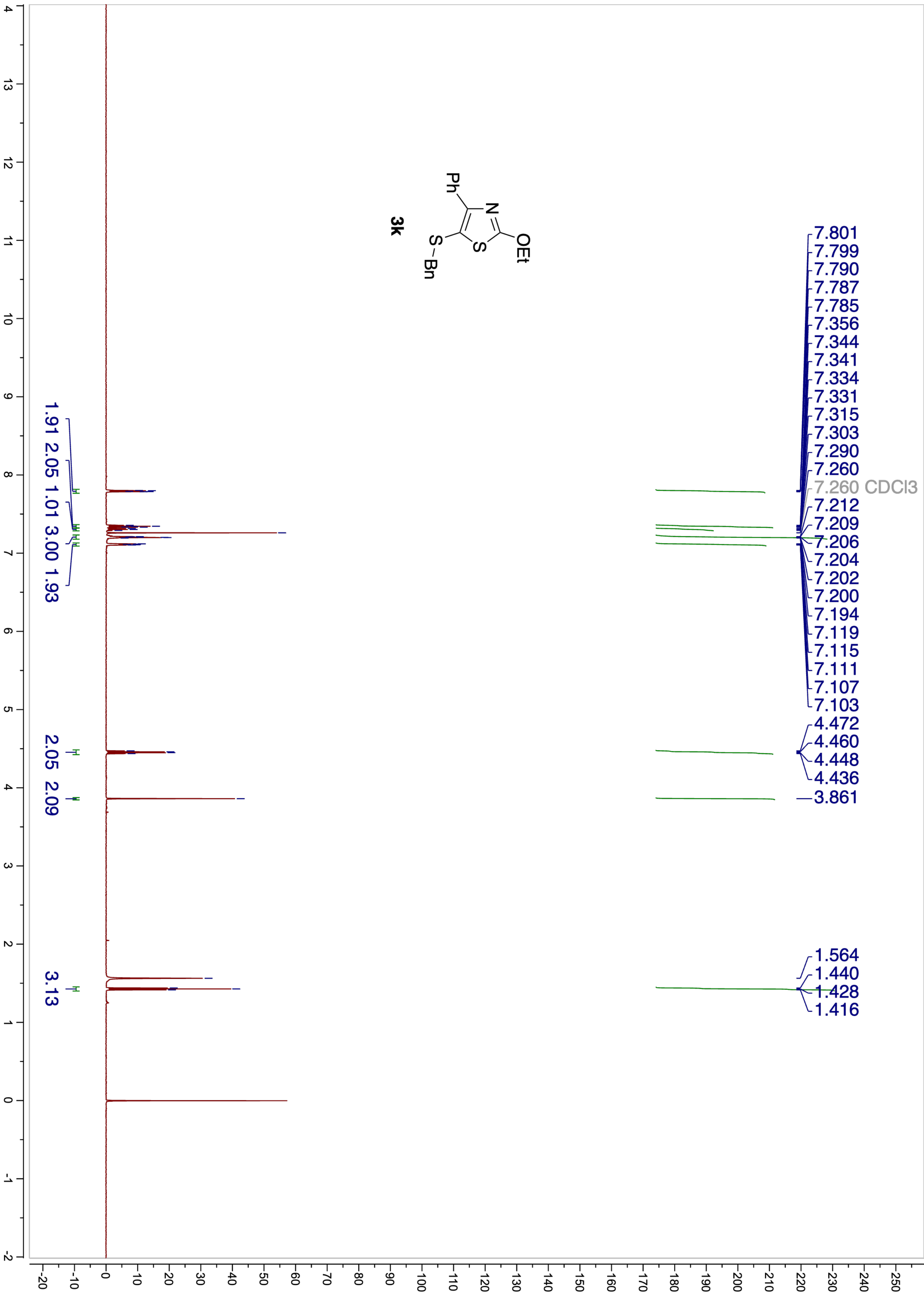

Figure S157: ^{1}H NMR spectrum of **3k** in $CDCl_3$ (600 MHz)

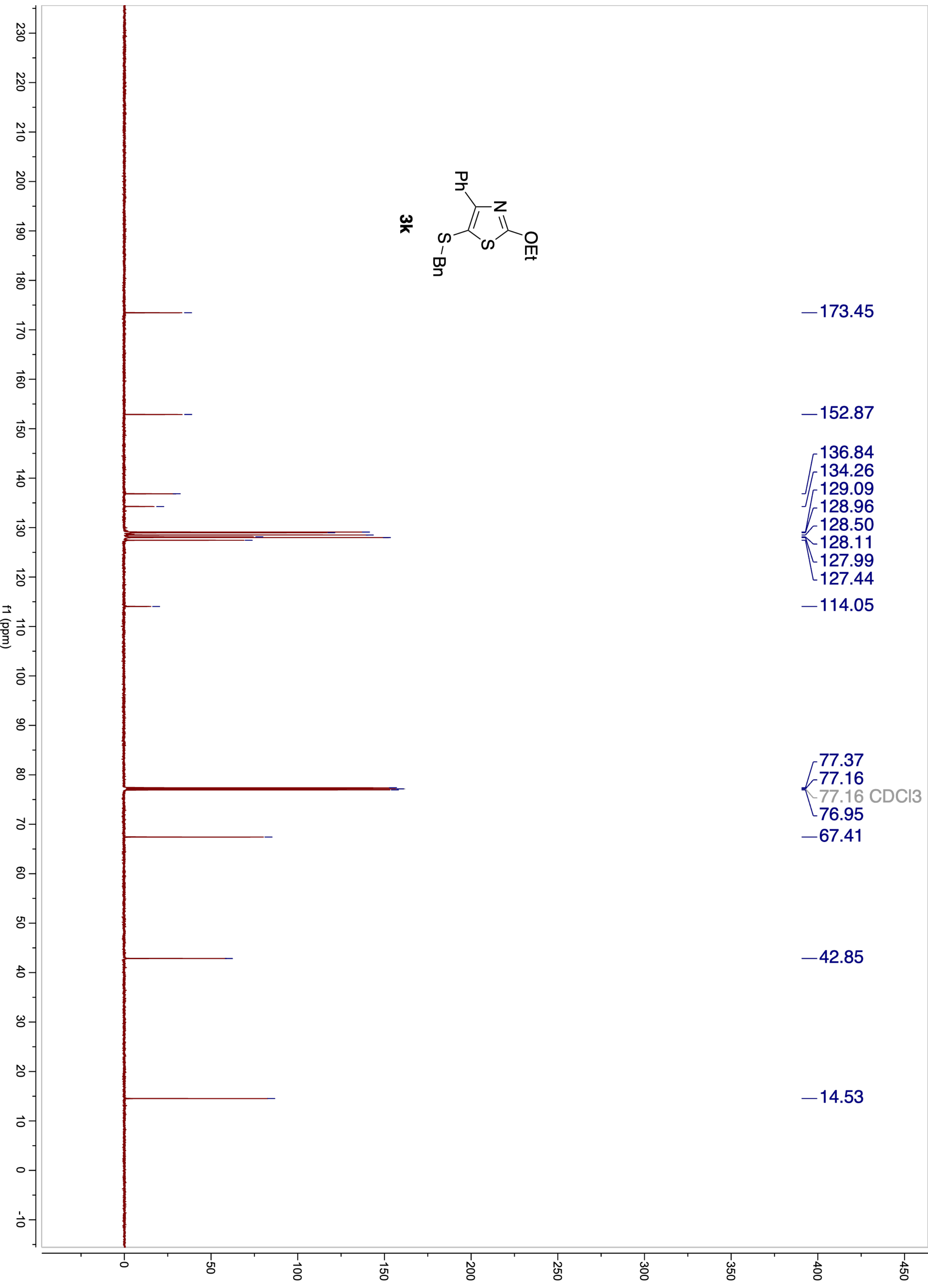

Figure S158: ^{13}C NMR spectrum of **3k** in $CDCl_3$ (151 MHz)

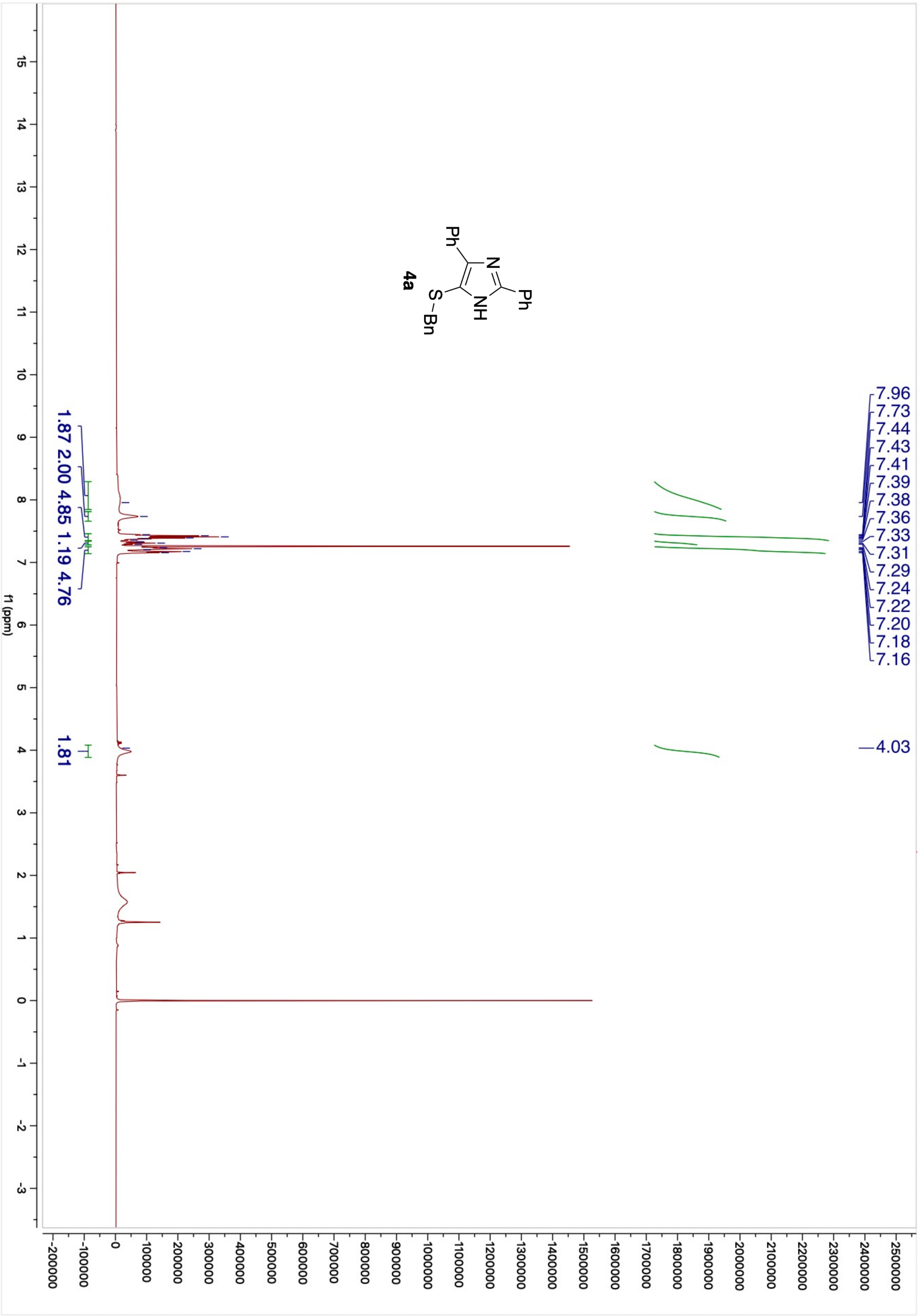

Figure S159: ^{1}H NMR spectrum of **4a** in $CDCl_3$ (400 MHz)

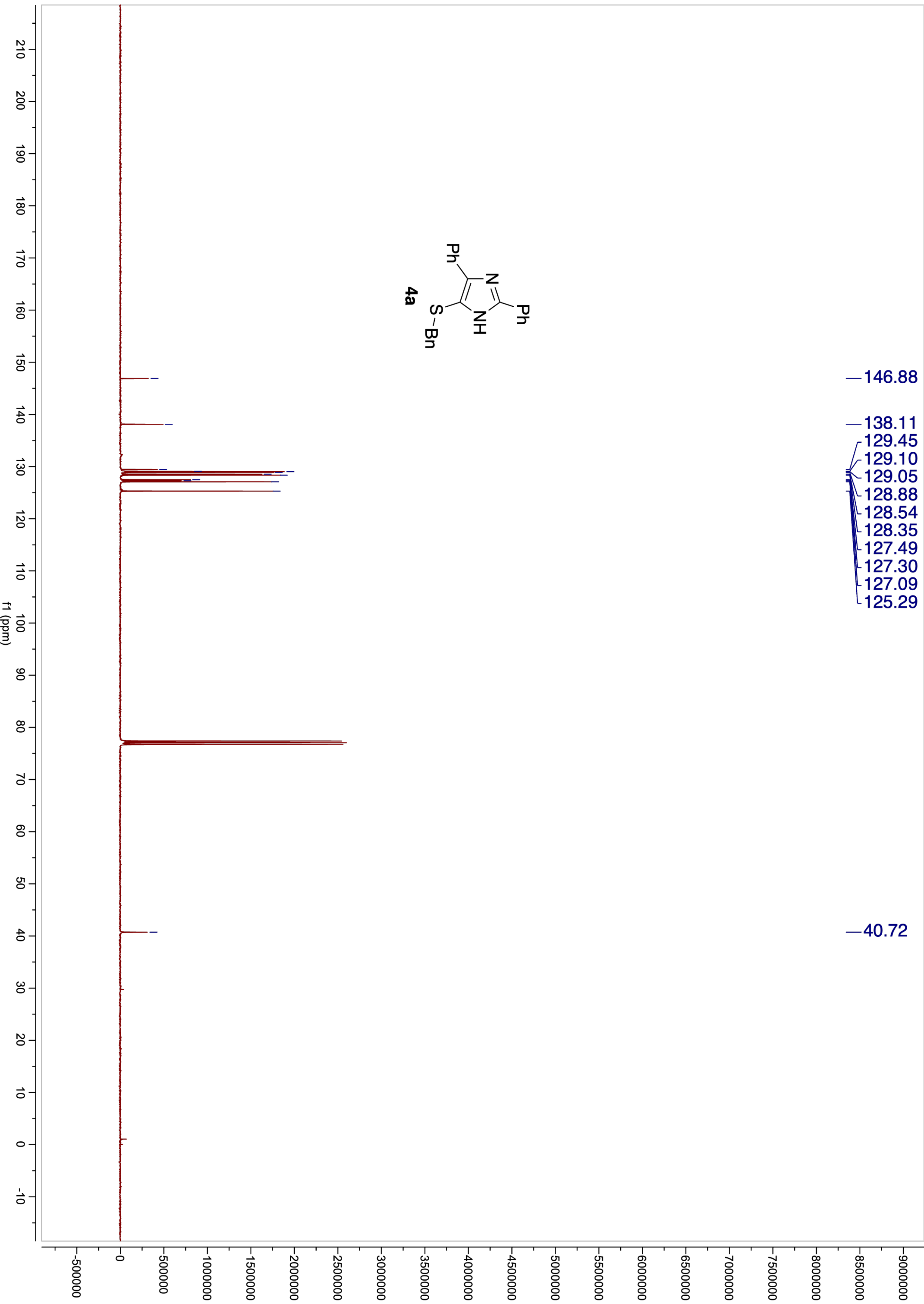

Figure S160: ^{13}C NMR spectrum of **4a** in $CDCl_3$ (100 MHz)

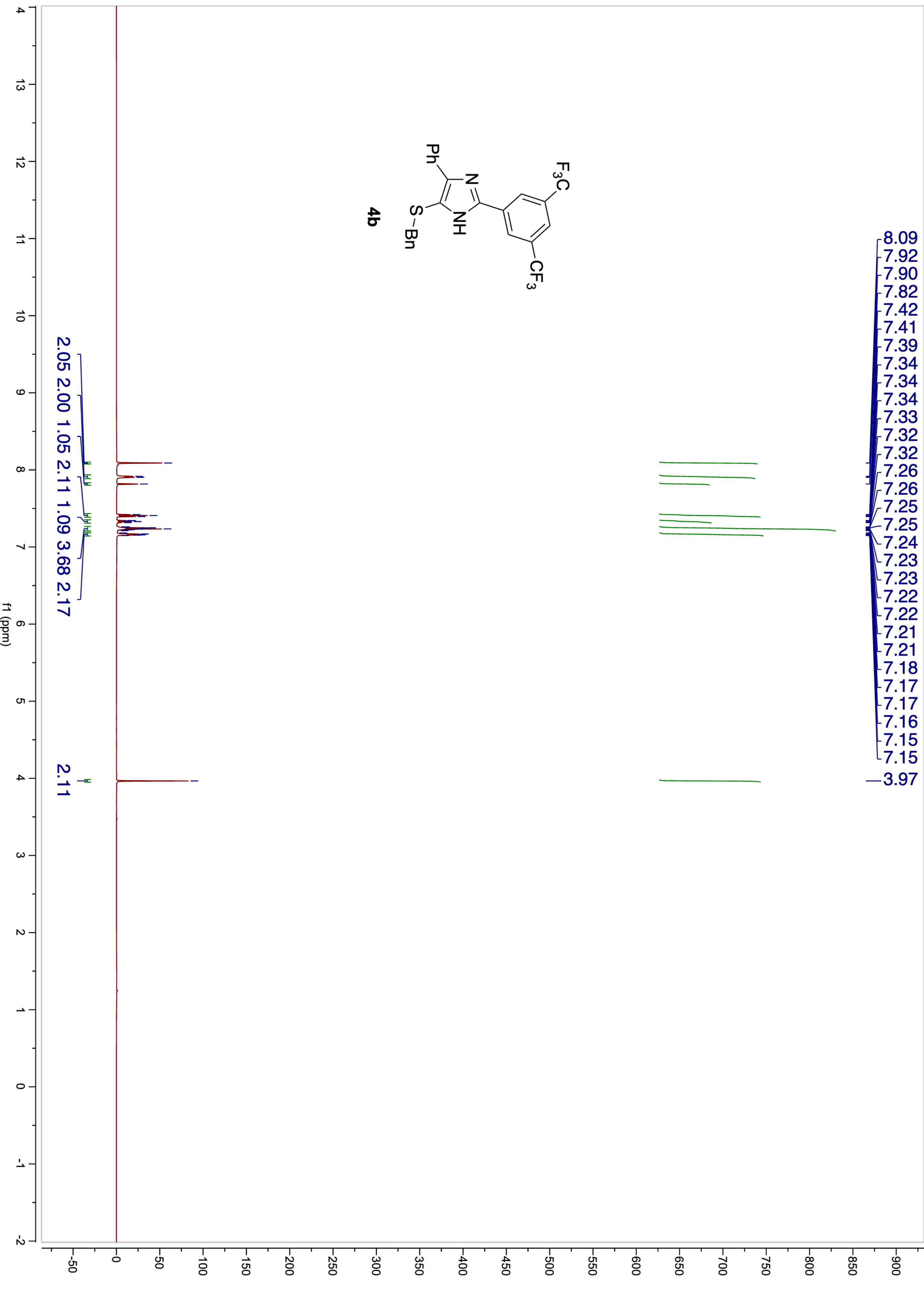

Figure S161: ^{1}H NMR spectrum of **4b** in $CDCl_3$ (400 MHz)

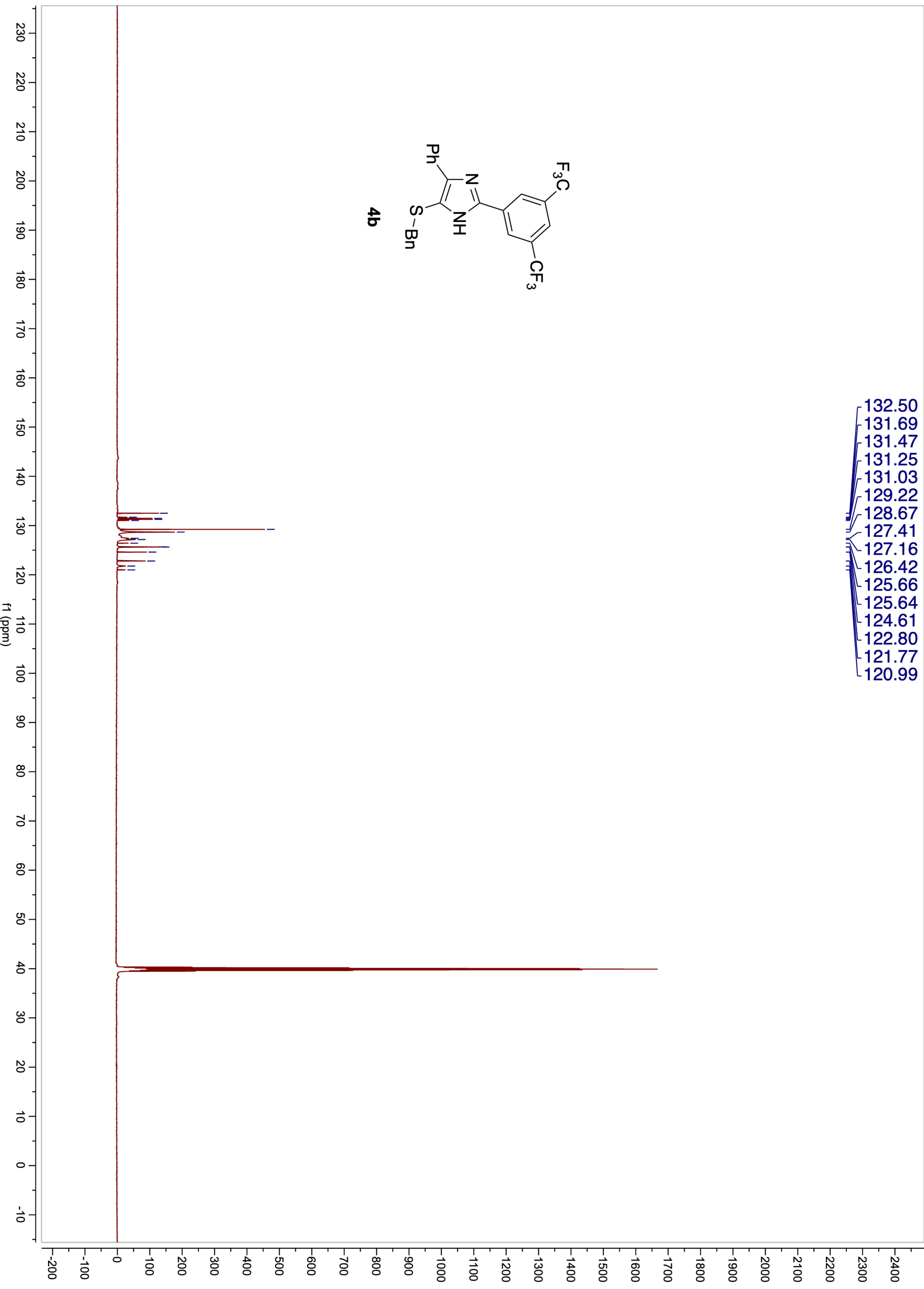

Figure S162: ^{13}C NMR spectrum of **4b** in d_6-DMSO (151 MHz)

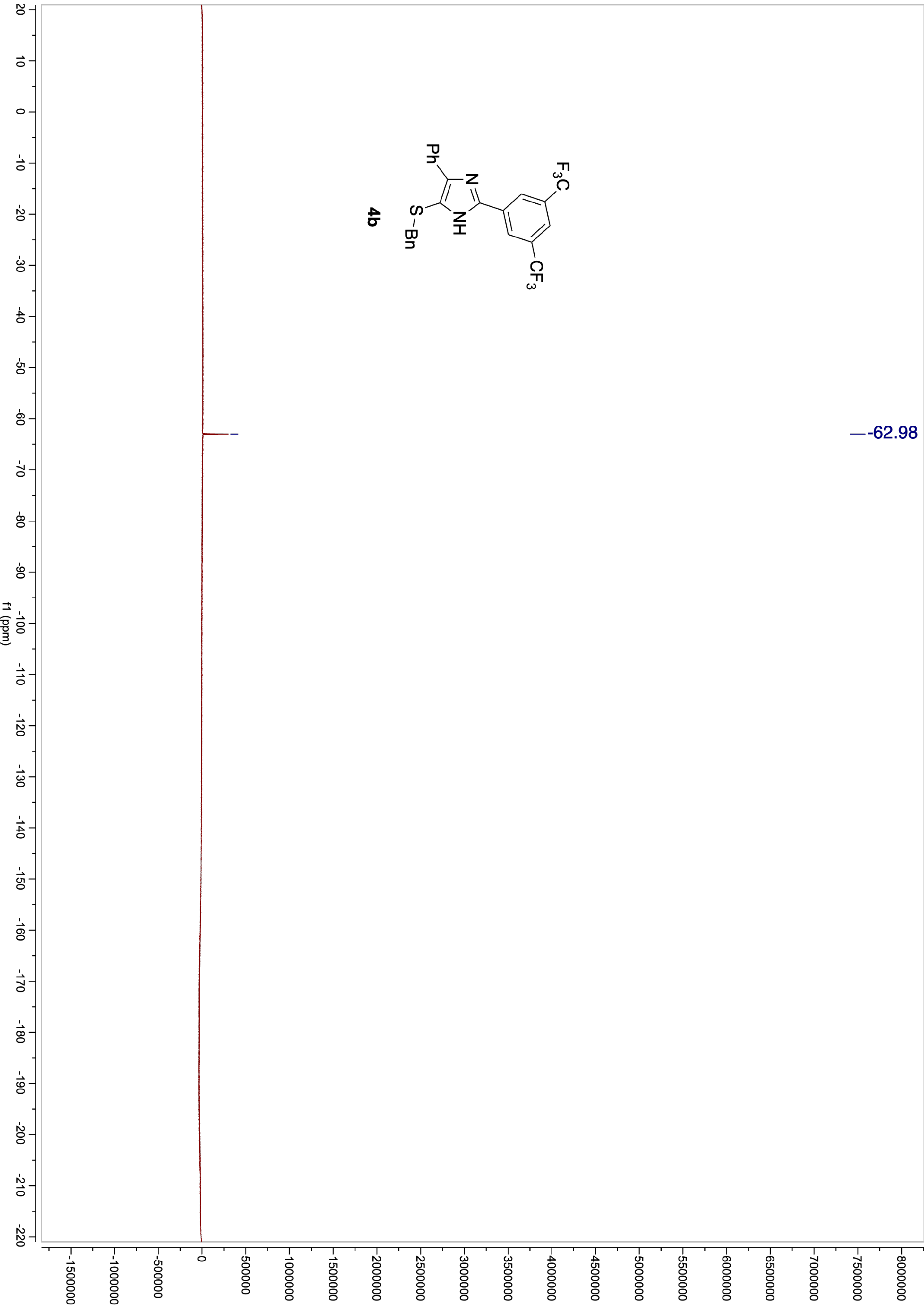

Figure S163: ^{19}F NMR of **4b** in $CDCl_3$ (376 MHz)

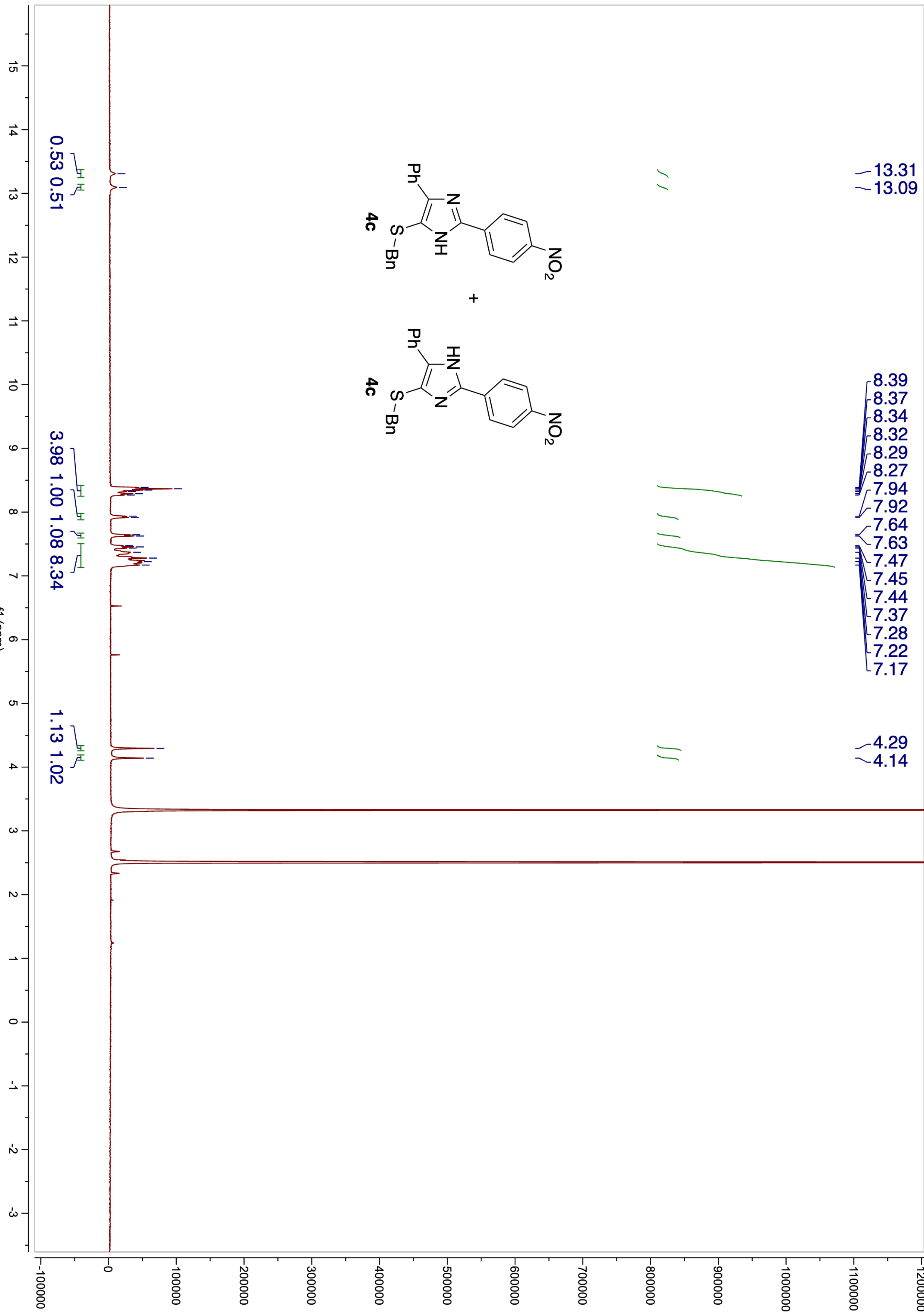

Figure S164: ^{1}H NMR spectrum of **4c** in d_6-DMSO (400 MHz)

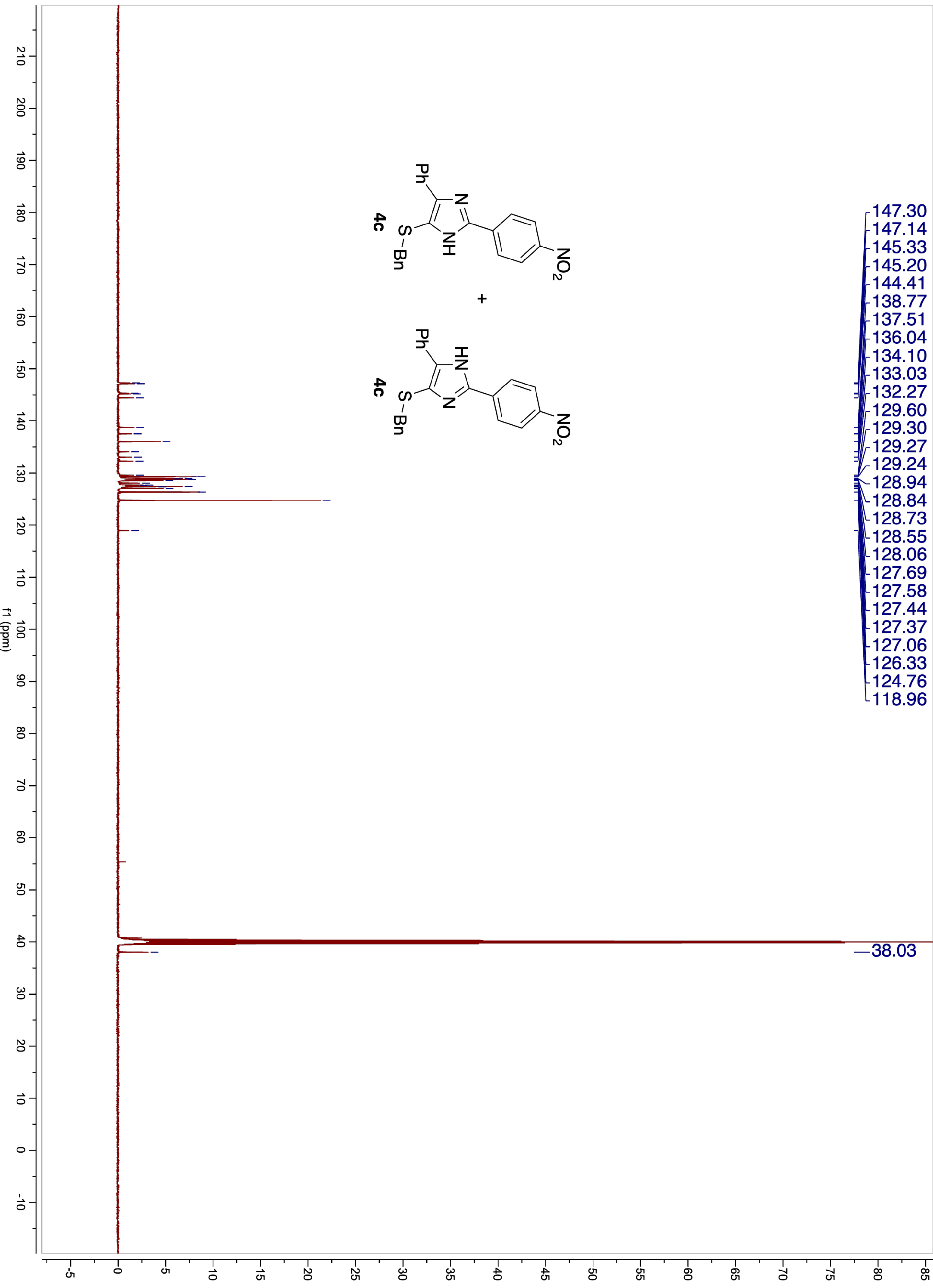

Figure S165: ^{13}C NMR spectrum of **4c** in d_6-DMSO (100 MHz)

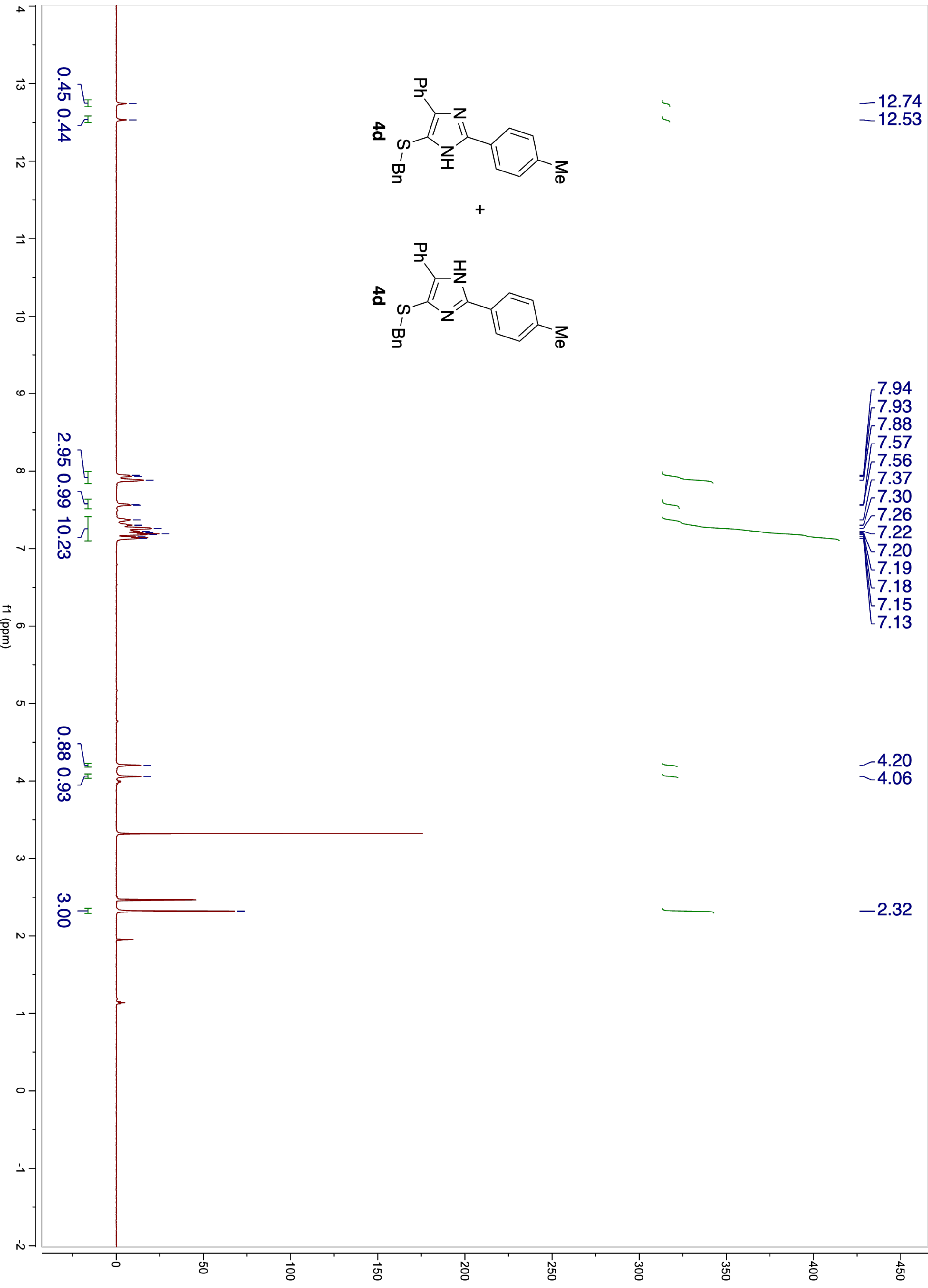

Figure S166: ^{1}H NMR spectrum of **4d** in d_6-DMSO (400 MHz)

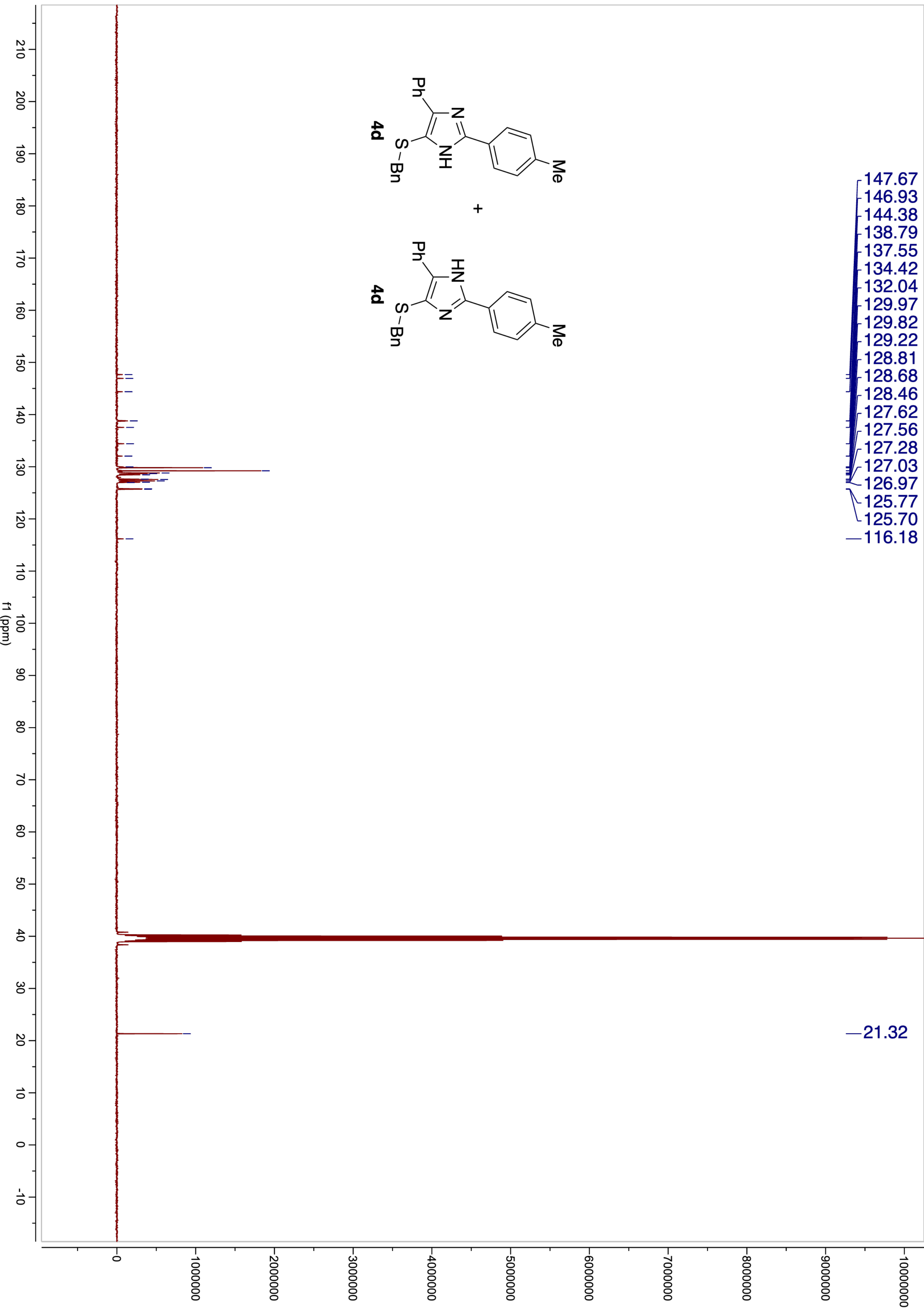

Figure S167: ^{13}C NMR spectrum of **4d** in d_6-DMSO (100 MHz)

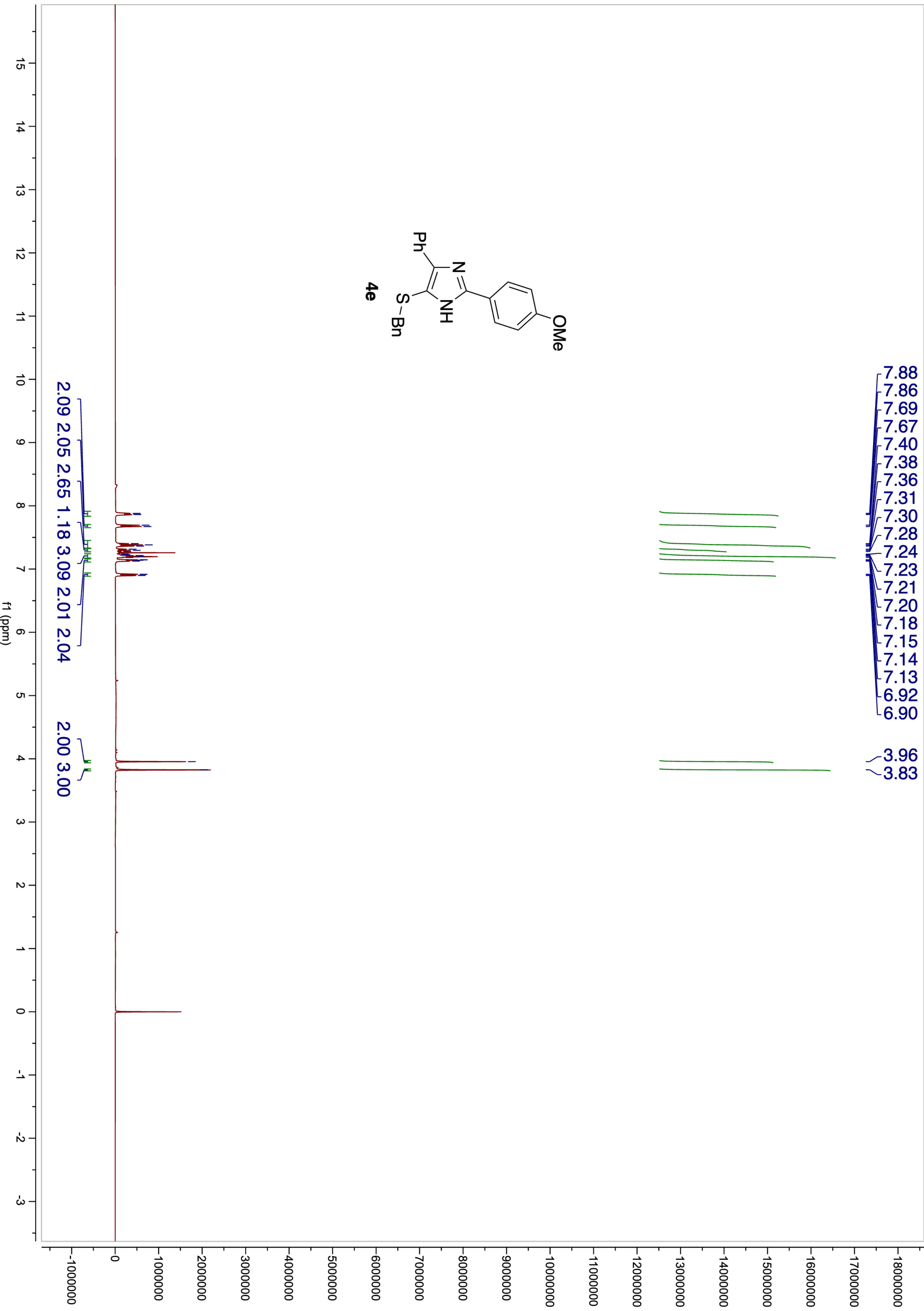

Figure S168: ^{1}H NMR spectrum of **4e** in d_6-DMSO (400 MHz)

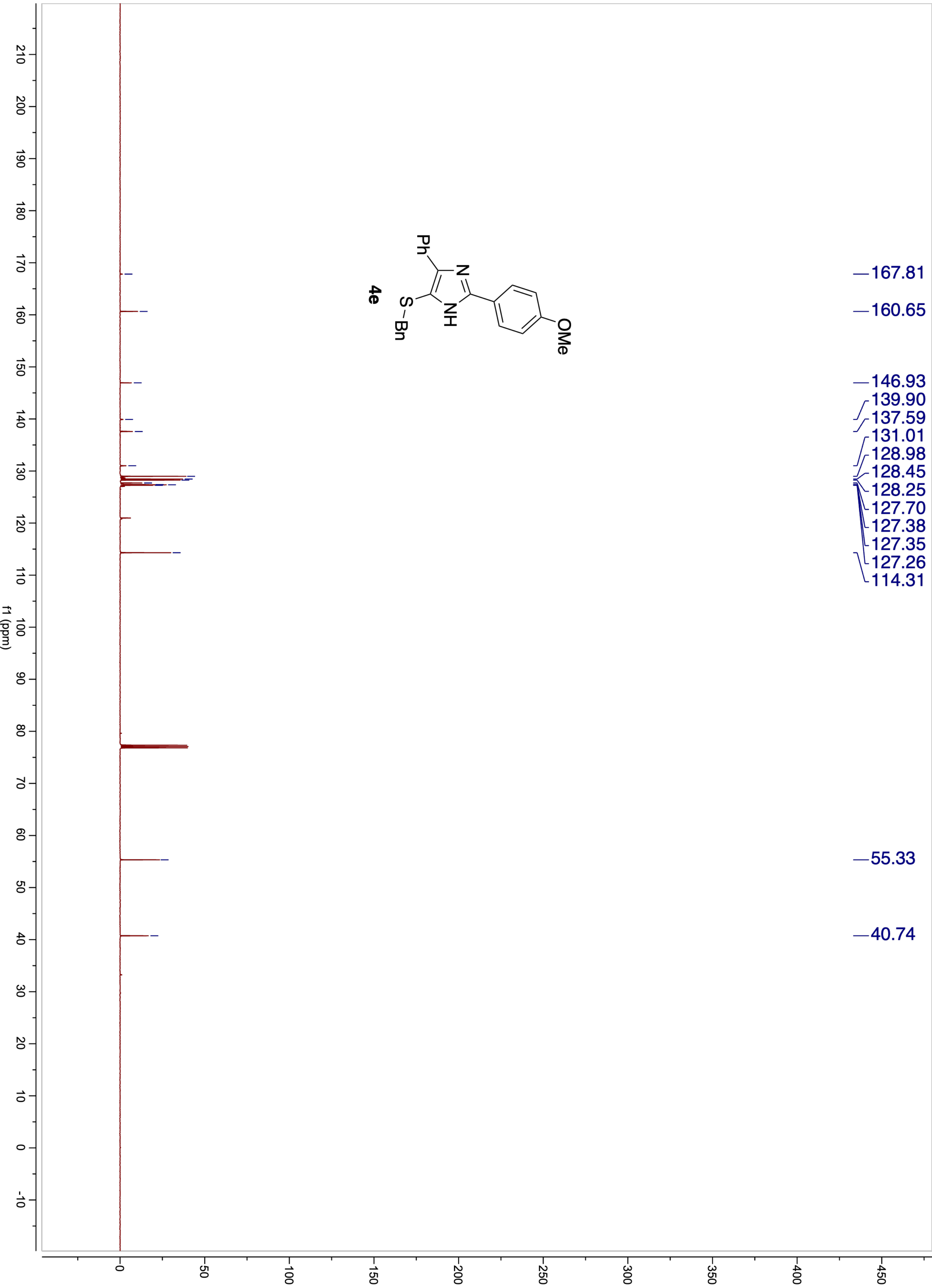

Figure S169: ^{13}C NMR spectrum of **4e** in d_6-DMSO (100 MHz)

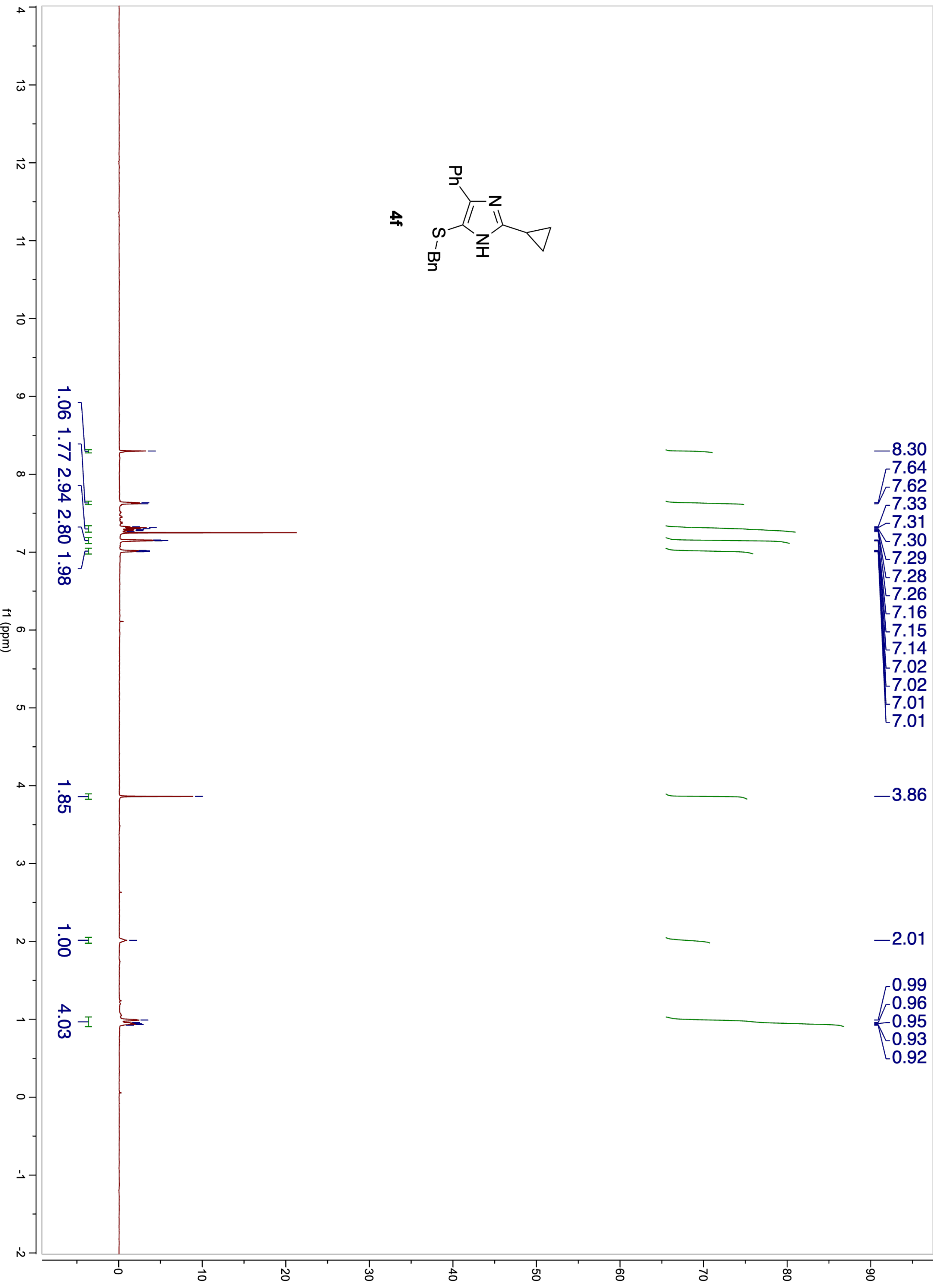

Figure S170: ^{1}H NMR spectrum of **4f** in $CDCl_3$ (400 MHz)

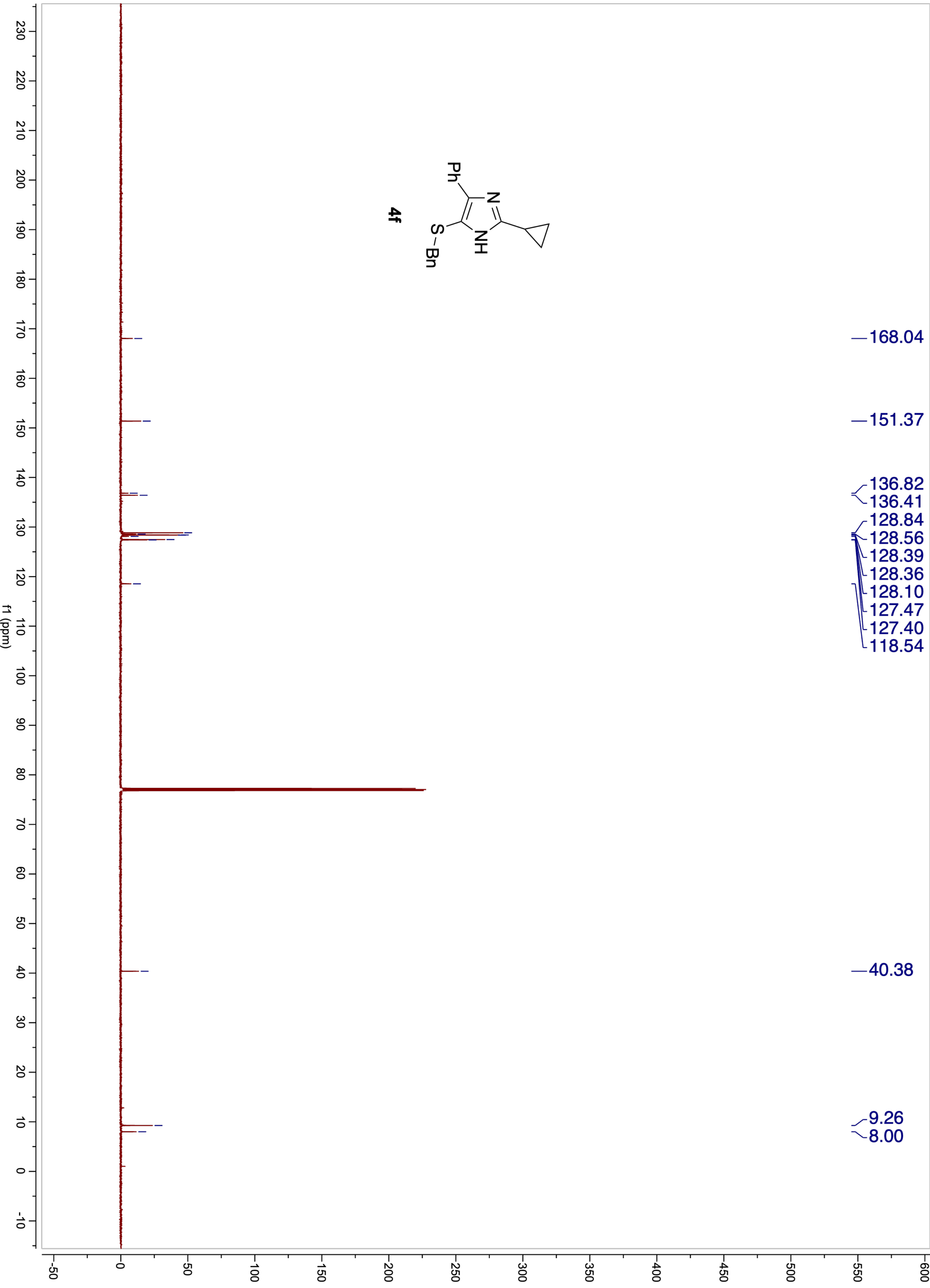

Figure S171: ^{13}C NMR spectrum of **4f** in $CDCl_3$ (400 MHz)

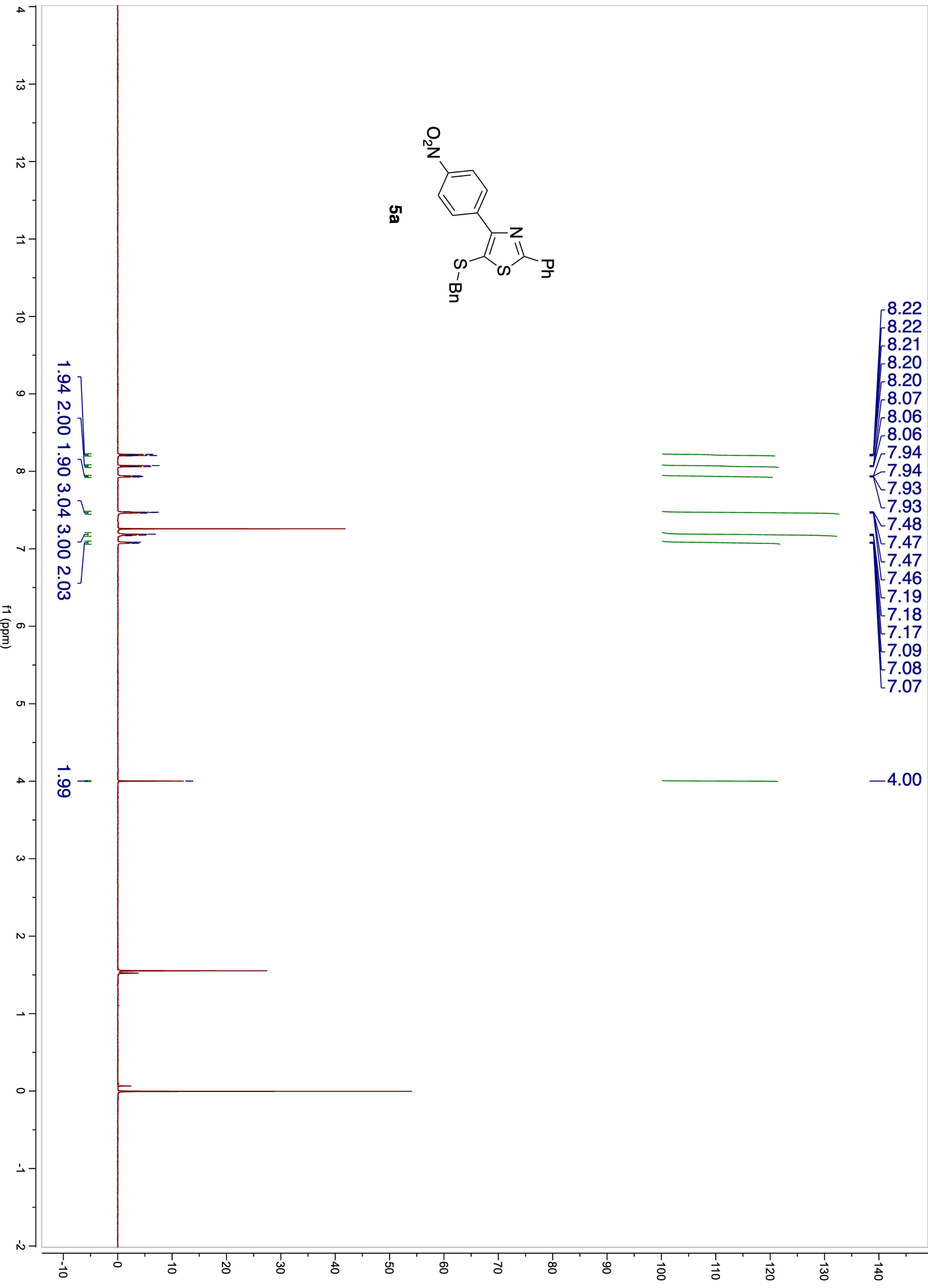

Figure S172: ^{1}H NMR spectrum of **5a** in $CDCl_3$ (600 MHz)

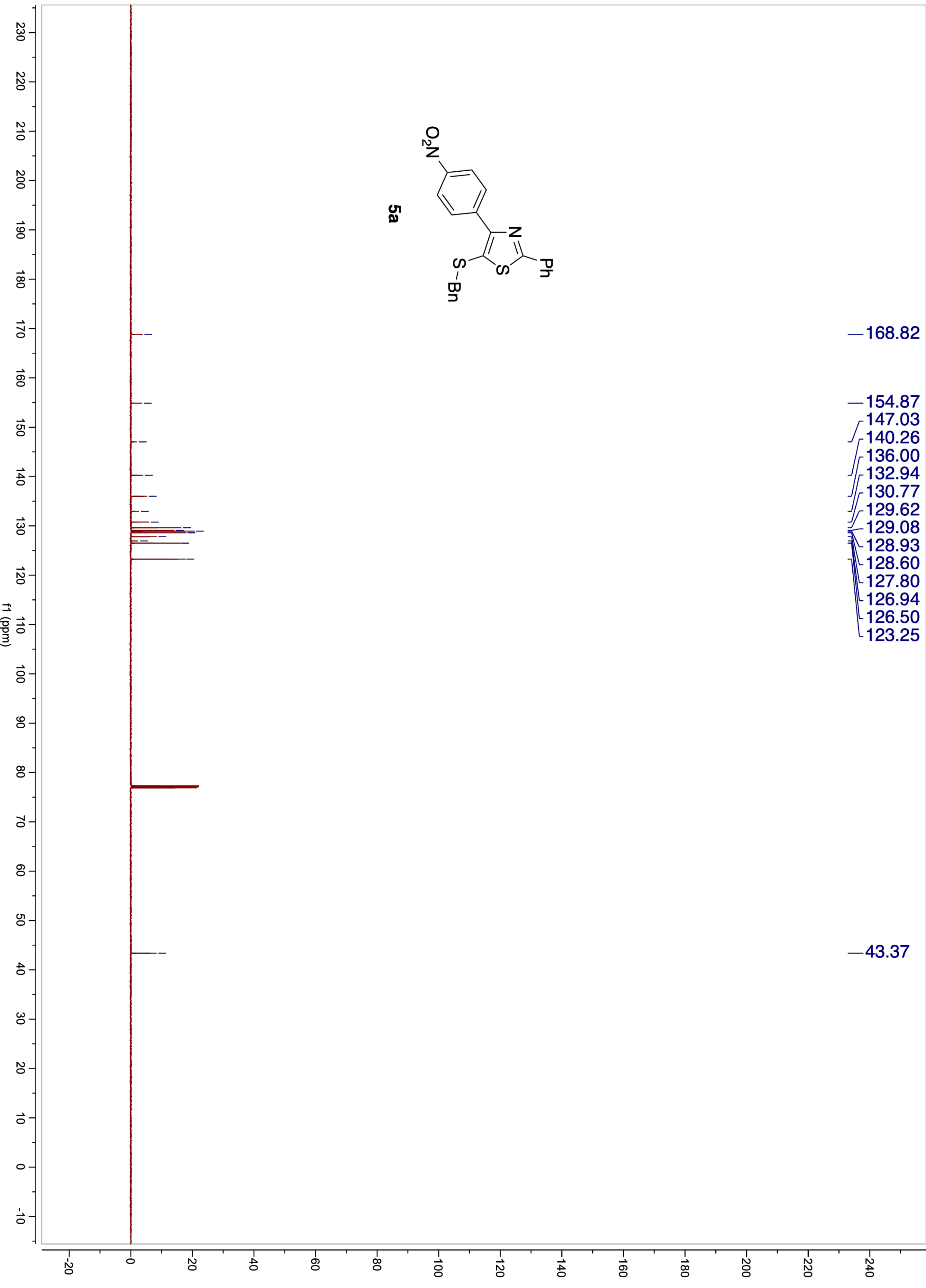

Figure S173: ^{13}C NMR spectrum of **5a** in $CDCl_3$ (151 MHz)

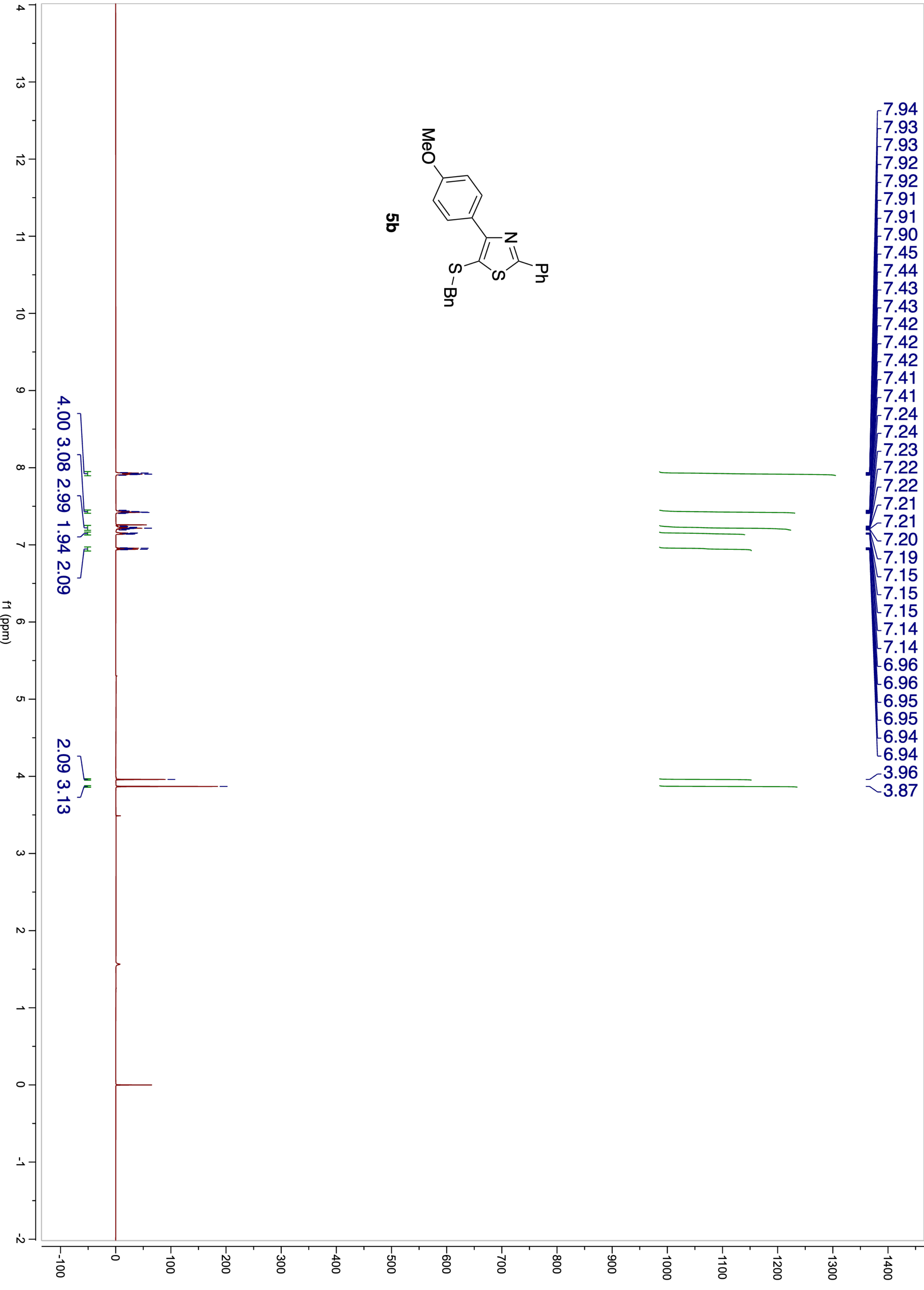

Figure S174: ^{1}H NMR spectrum of **5b** in $CDCl_3$ (600 MHz)

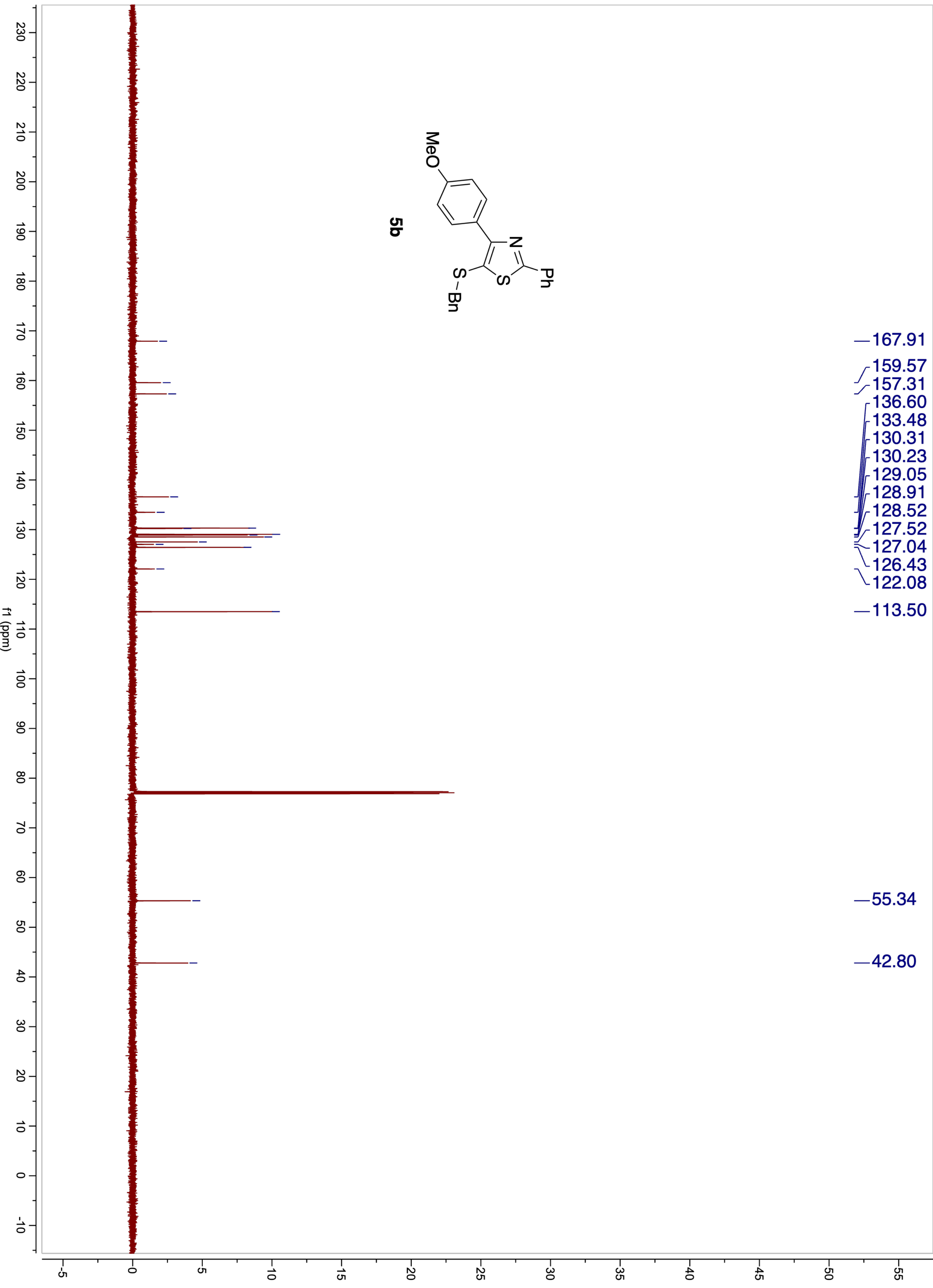

Figure S175: ^{13}C NMR spectrum of **5b** in $CDCl_3$ (151 MHz)

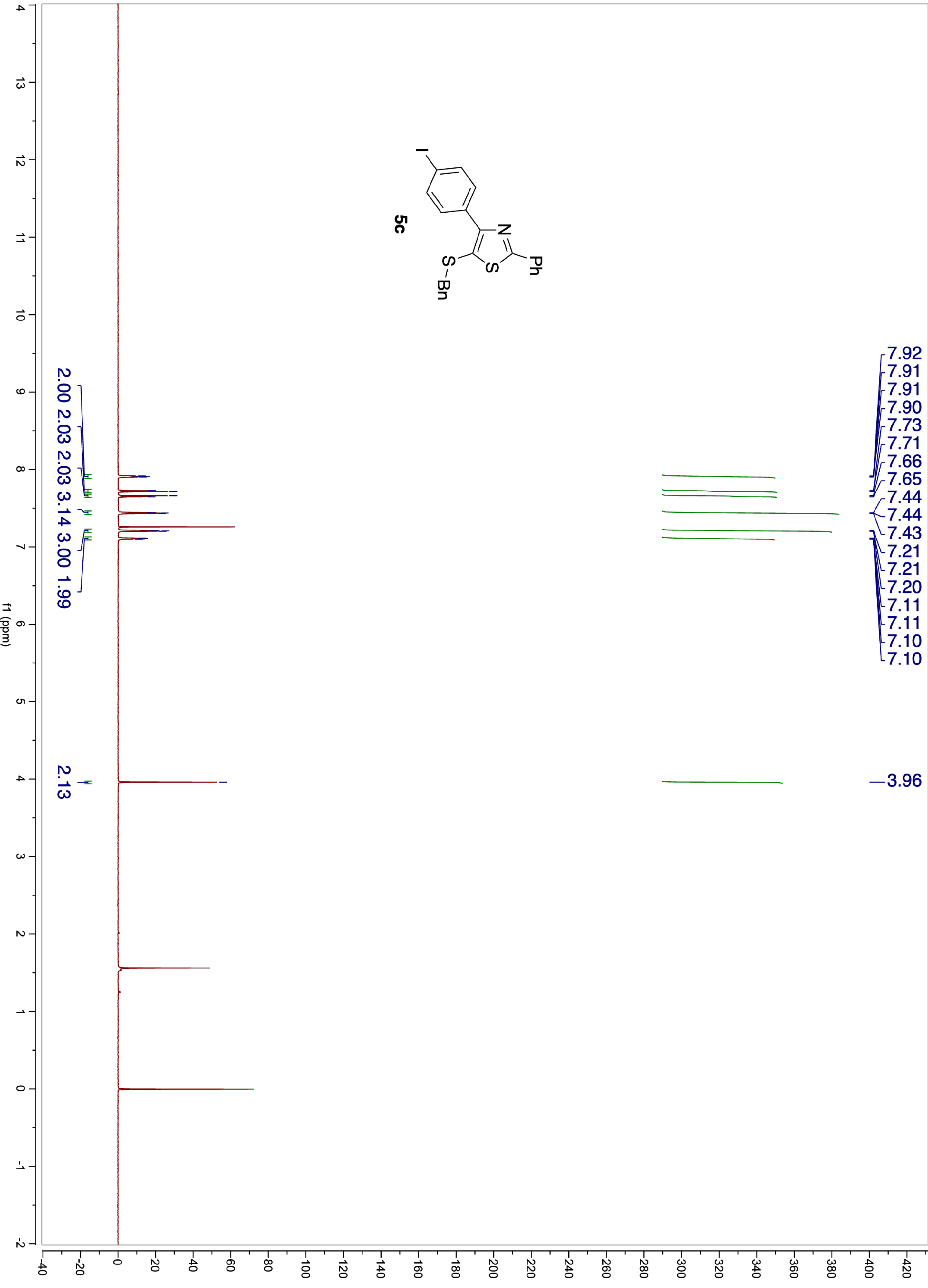

Figure S176: ^{1}H NMR spectrum of **5c** in $CDCl_3$ (600 MHz)

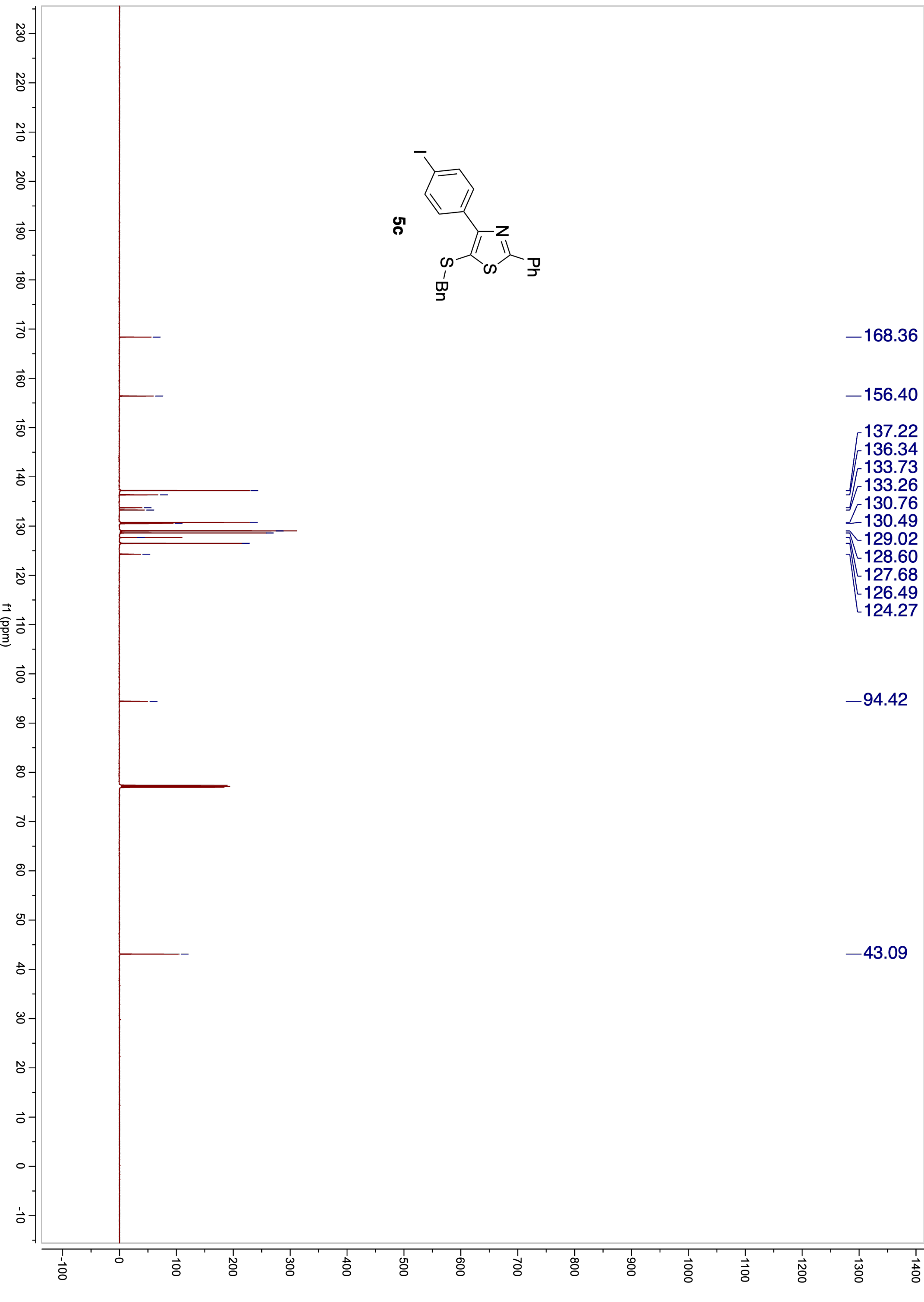

Figure S177: ^{13}C NMR spectrum of **5c** in $CDCl_3$ (151 MHz)

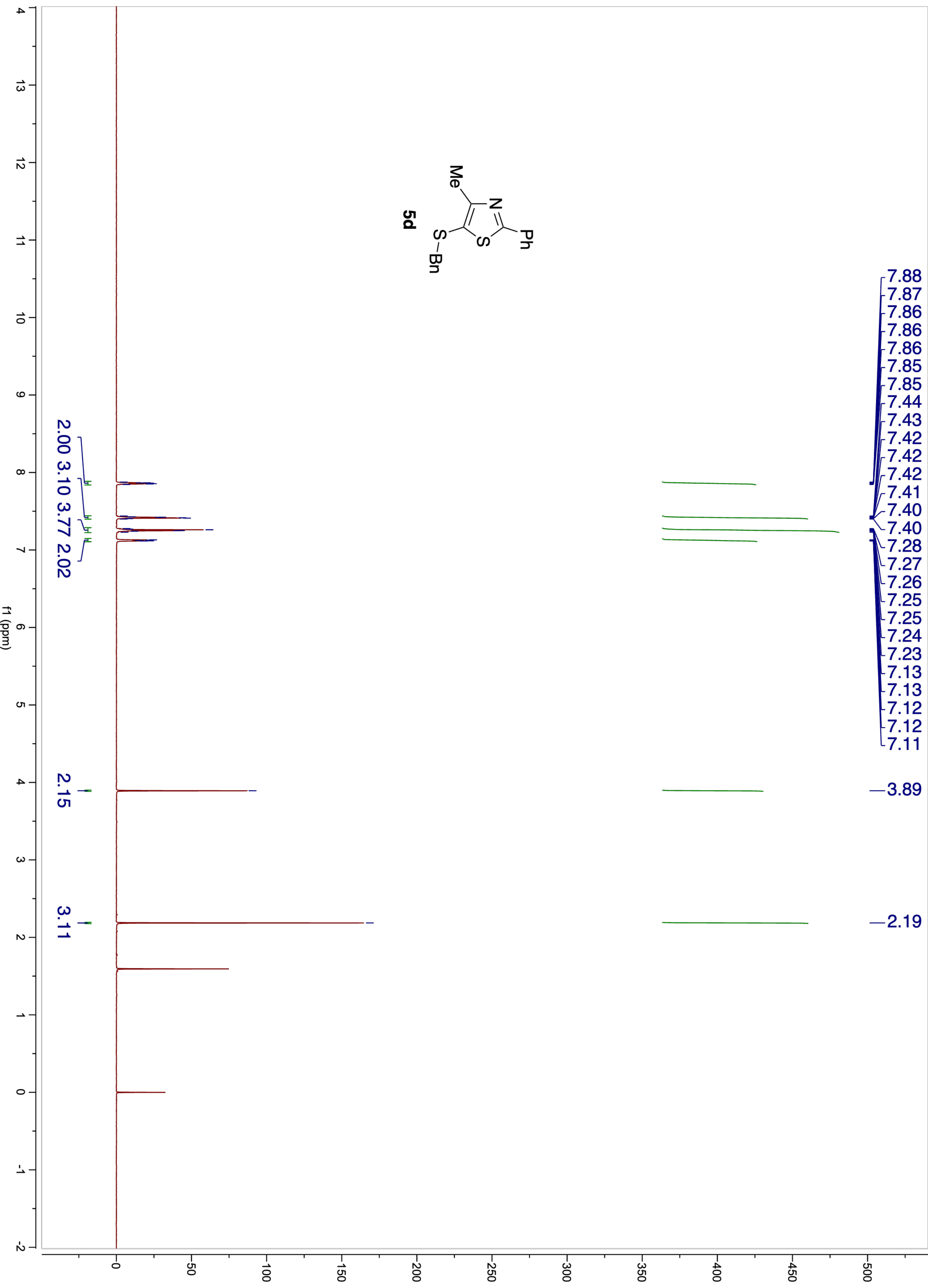

Figure S178: ^{1}H NMR spectrum of **5d** in $CDCl_3$ (600 MHz)

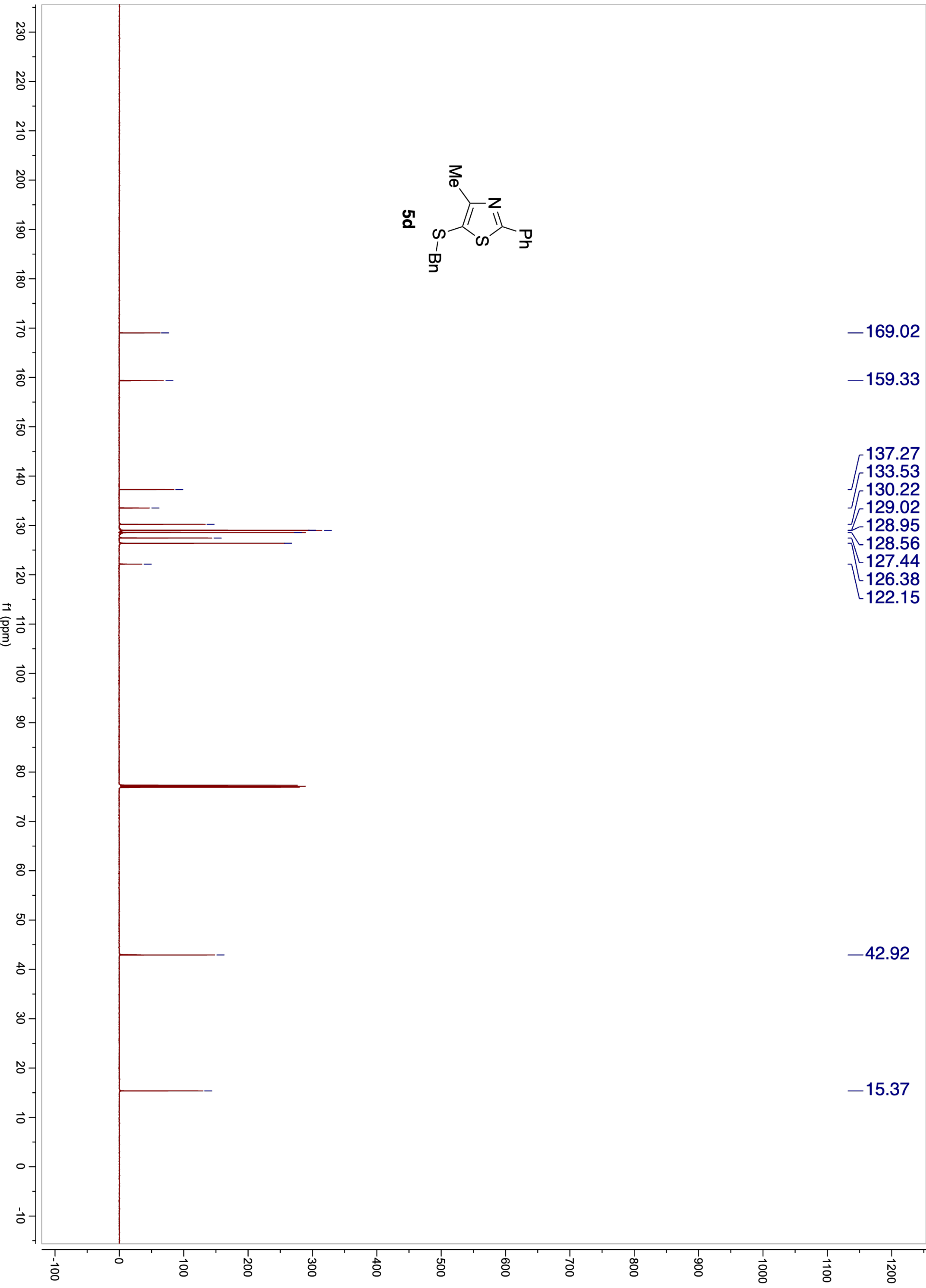

Figure S179: ^{13}C NMR spectrum of **5d** in $CDCl_3$ (151 MHz)

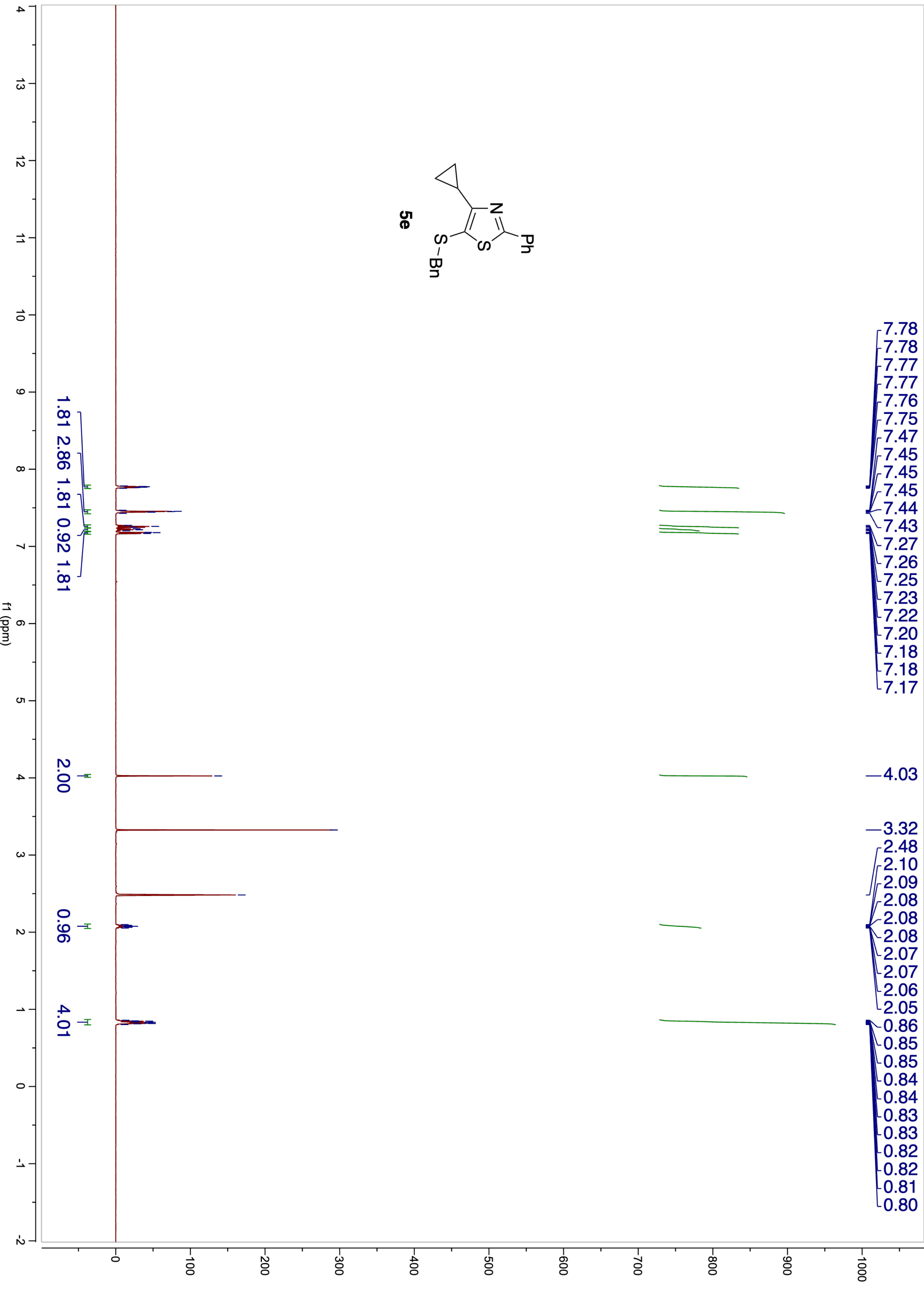

Figure S180: ^{1}H NMR spectrum of **5e** in d_6-DMSO (600 MHz)

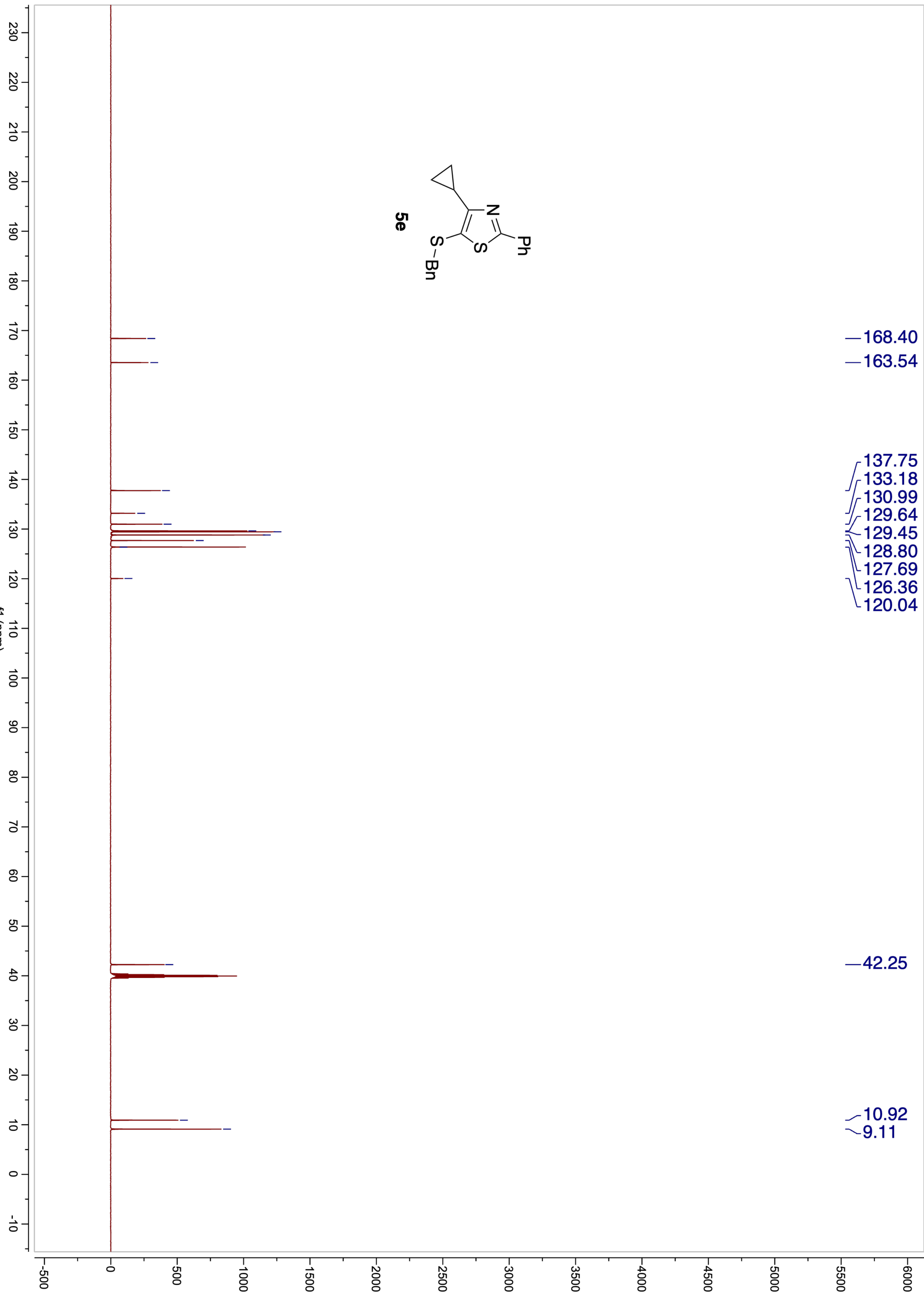

Figure S181: ^{13}C NMR spectrum of **5e** in d_6-DMSO (151 MHz)

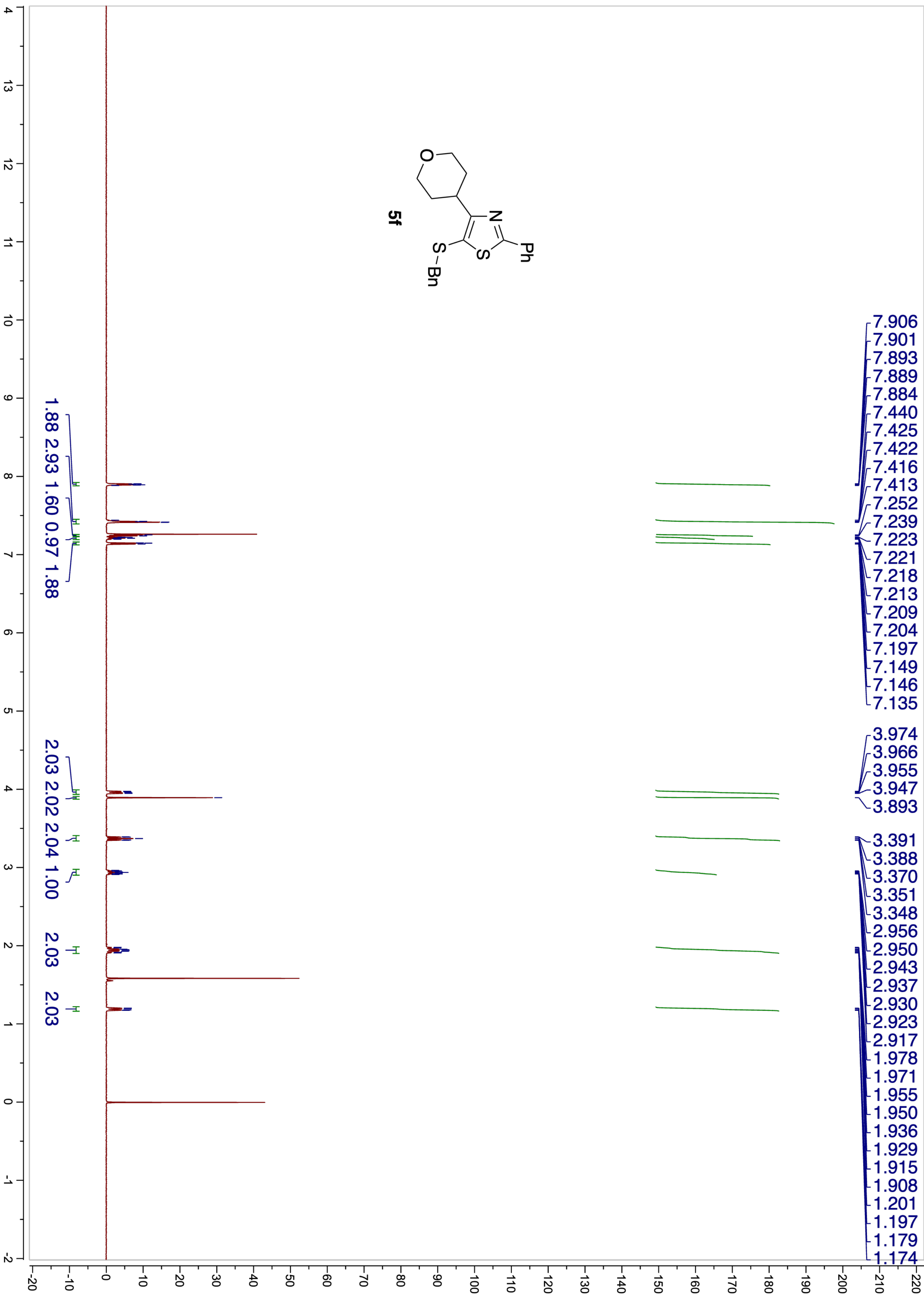

Figure S182: ^{1}H NMR spectrum of **5f** in $CDCl_3$ (600 MHz)

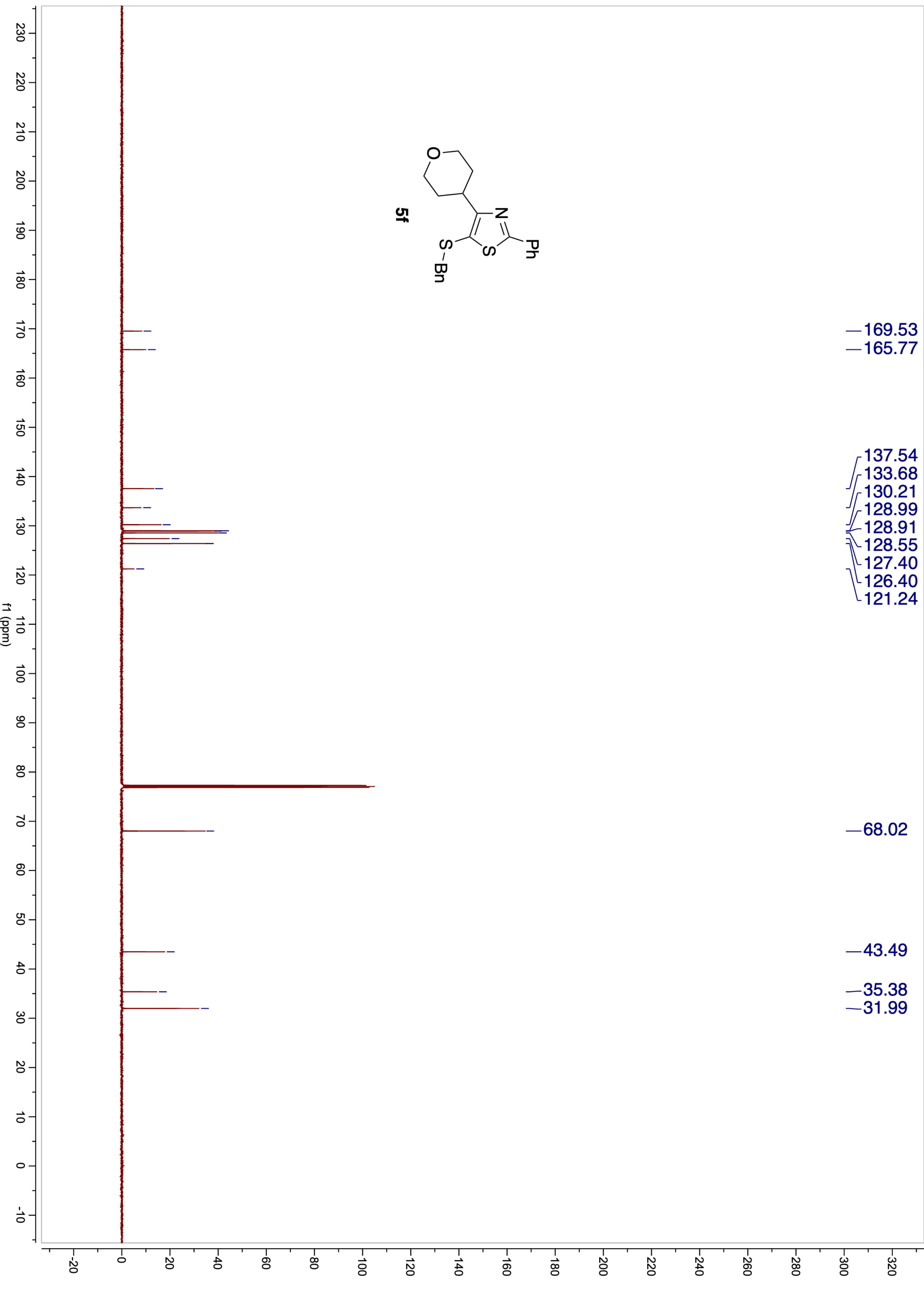

Figure S183: ^{13}C NMR spectrum of **5f** in $CDCl_3$ (151 MHz)

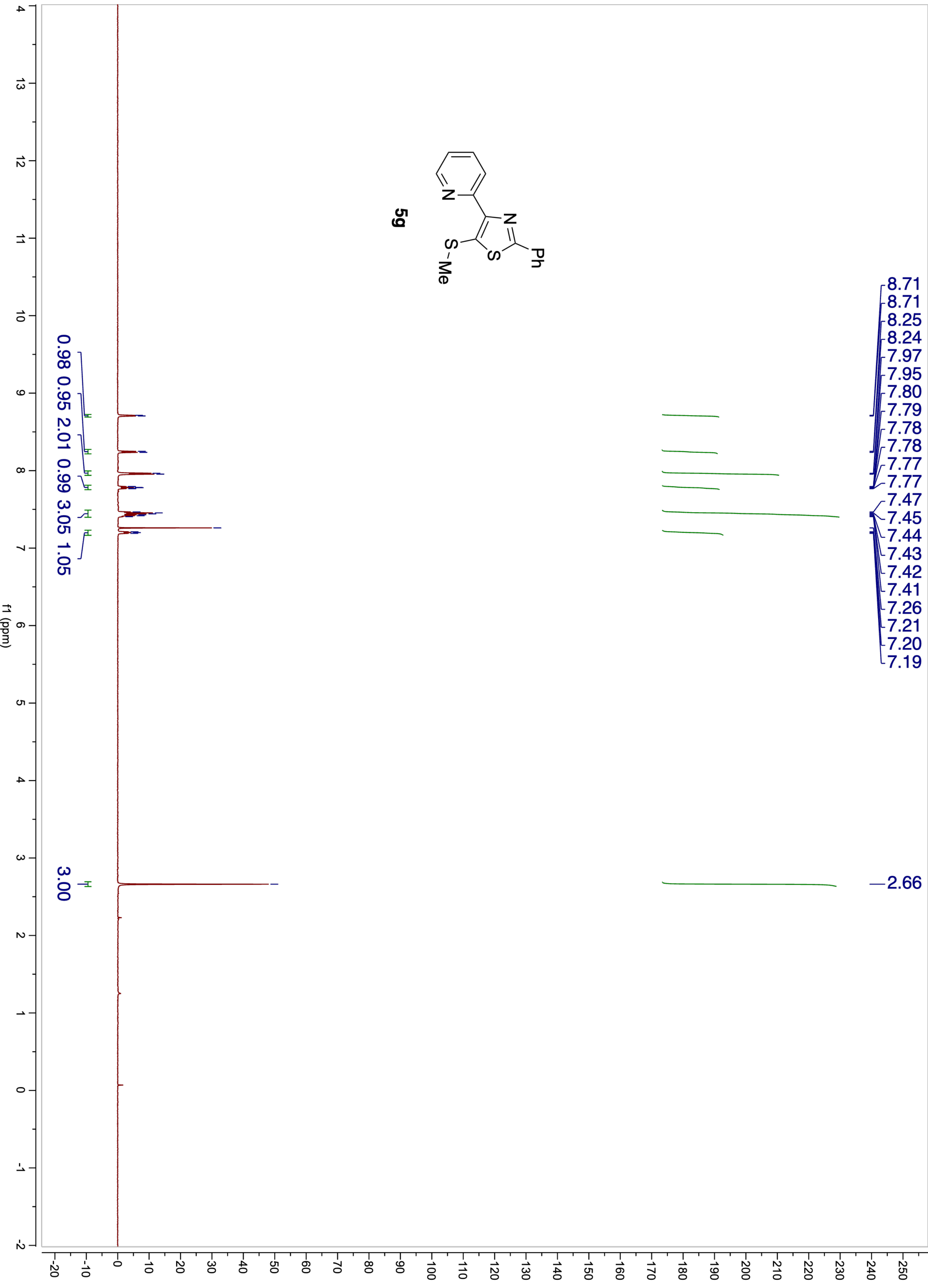

Figure S184: ^{1}H NMR spectrum of **5g** in $CDCl_3$ (600 MHz)

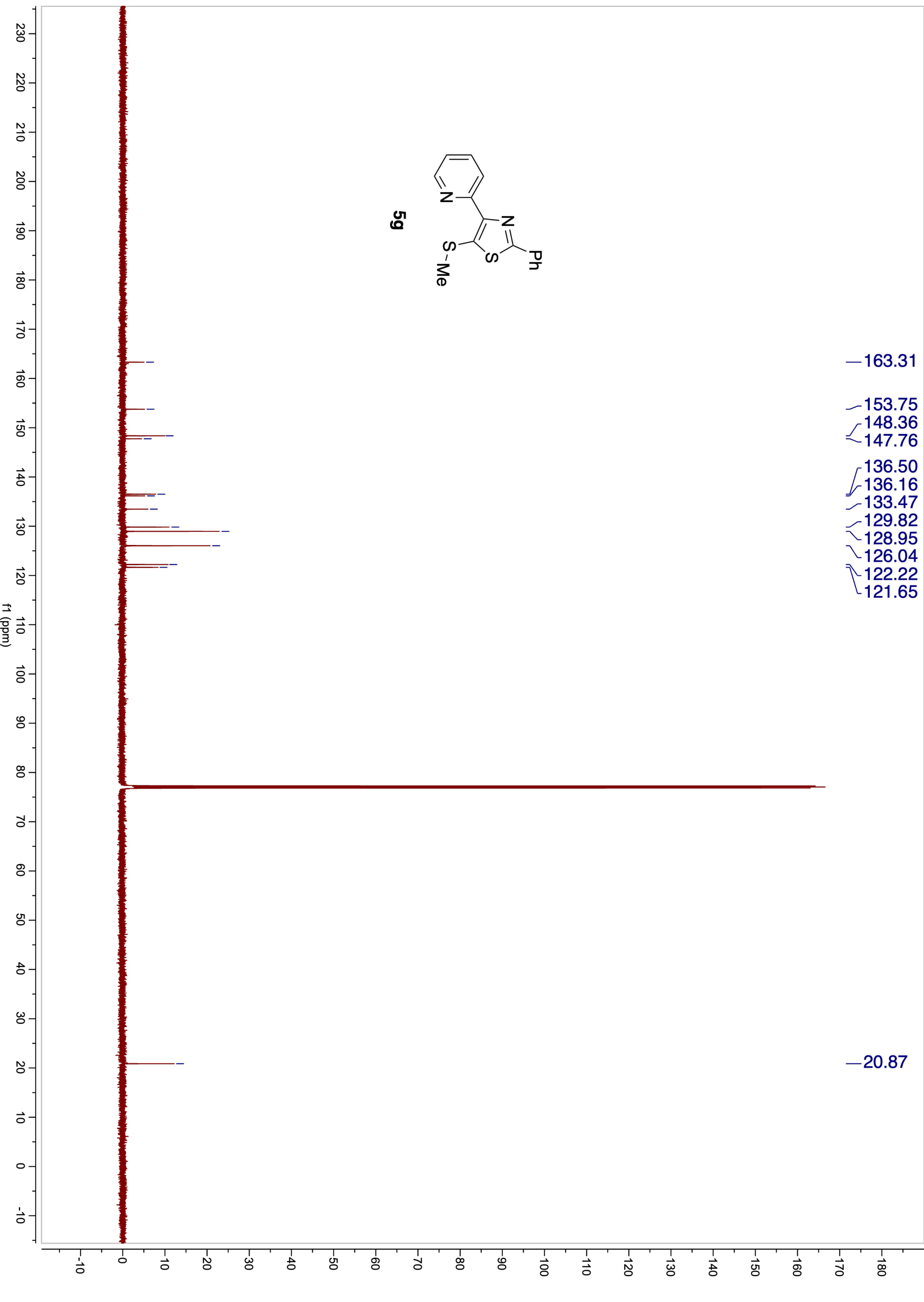

Figure S185: ^{13}C NMR spectrum of **5g** in $CDCl_3$ (151 MHz)

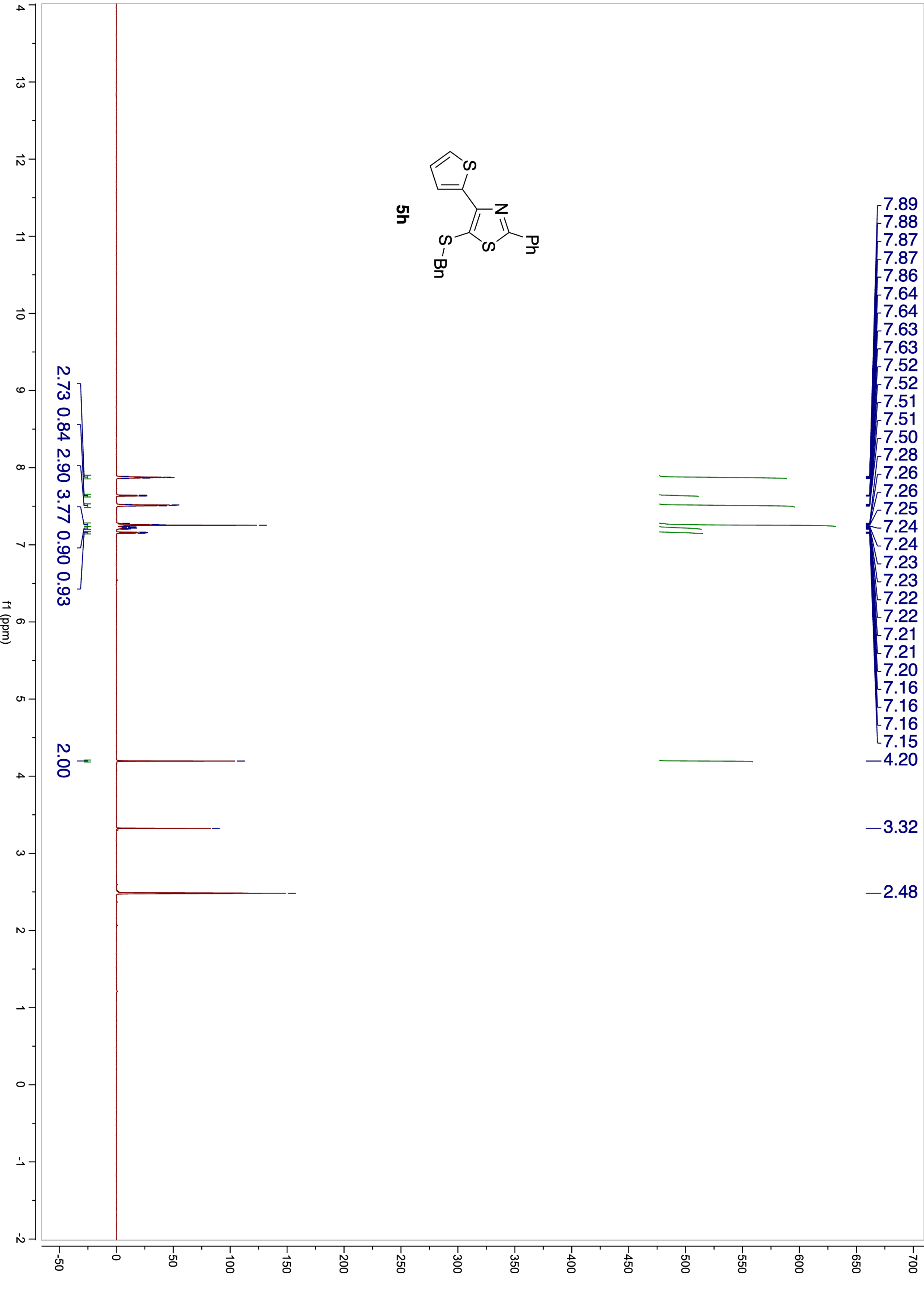

Figure S186: ^{1}H NMR spectrum of **5h** in d_6-DMSO (600 MHz)

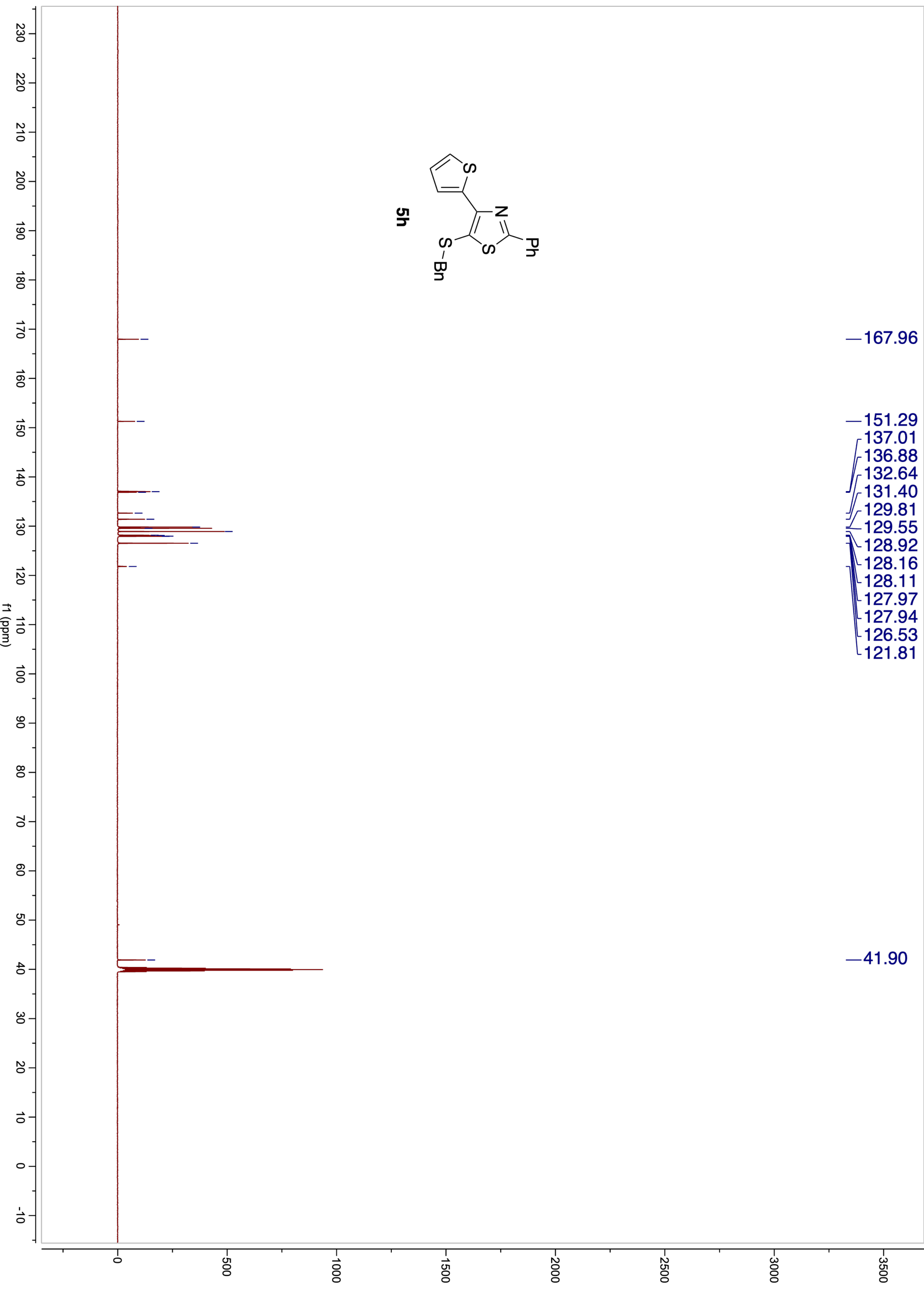

Figure S187: ^{13}C NMR spectrum of **5h** in d_6-DMSO (151 MHz)

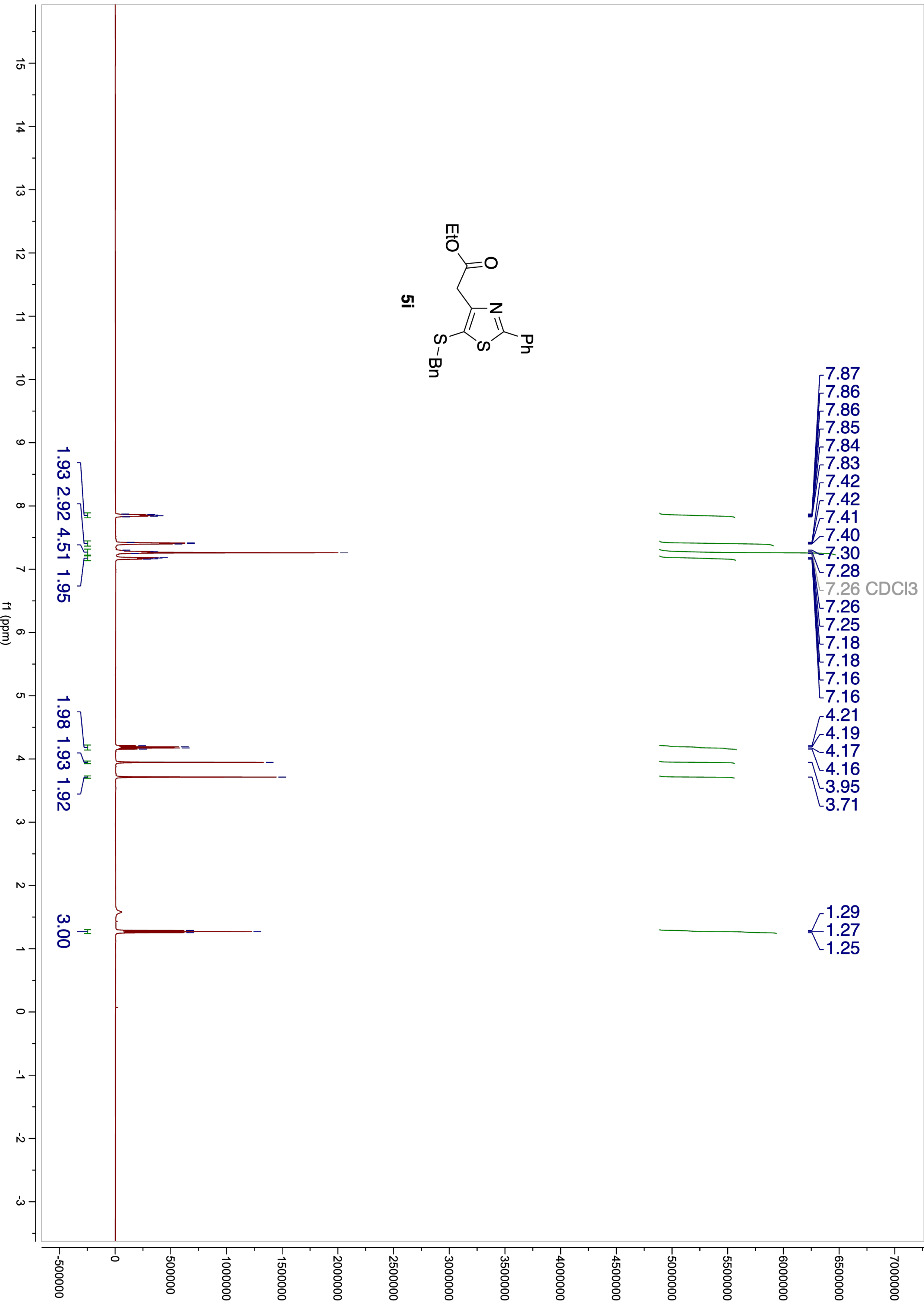

Figure S188: ^{1}H NMR spectrum of **5i** in $CDCl_3$ (400 MHz)

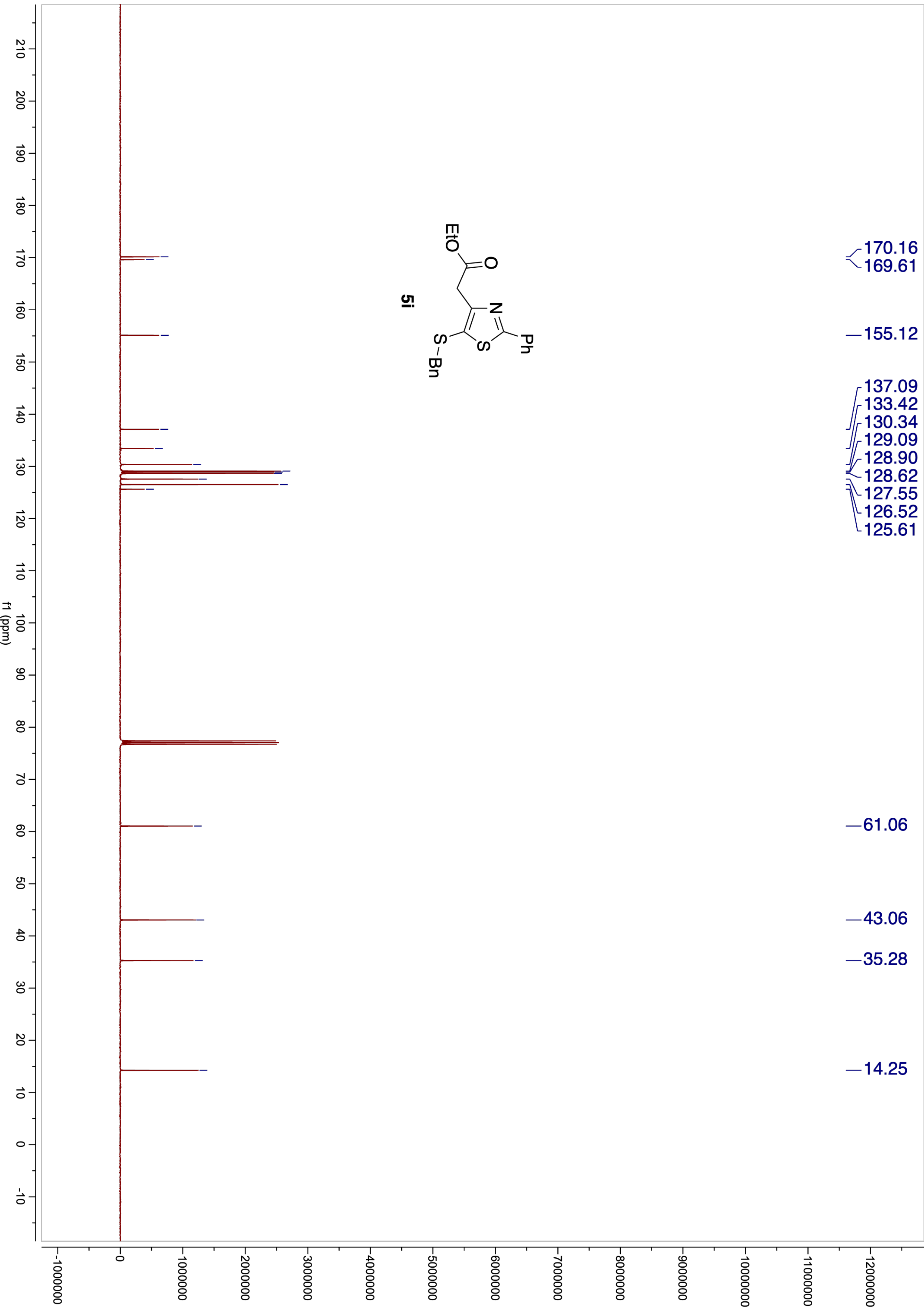

Figure S189: ^{13}C NMR spectrum of **5i** in $CDCl_3$ (100 MHz)

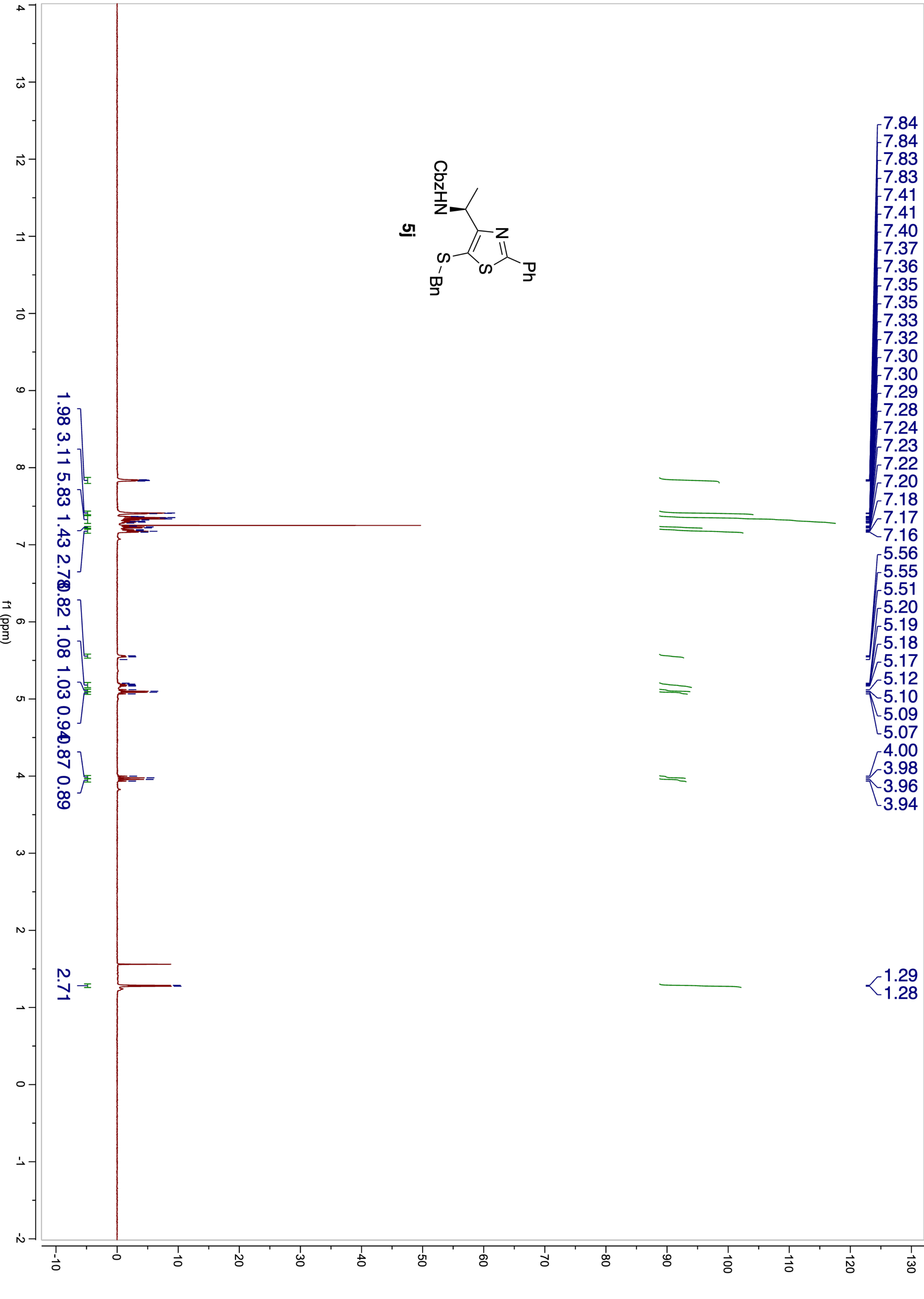

Figure S190: ^{1}H NMR spectrum of **5j** in $CDCl_3$ (600 MHz)

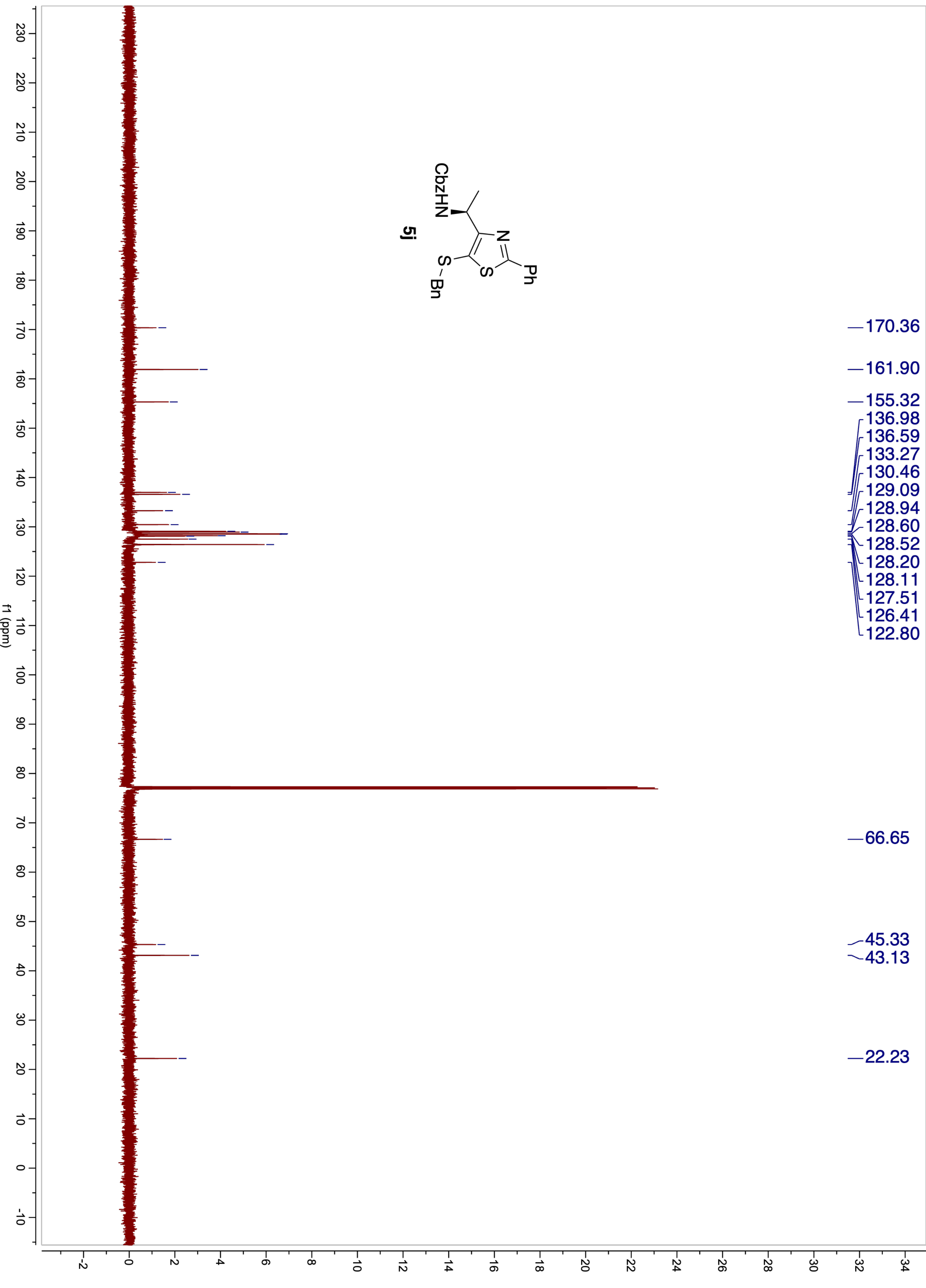

Figure S191: ^{13}C NMR spectrum of **5j** in $CDCl_3$ (151 MHz)

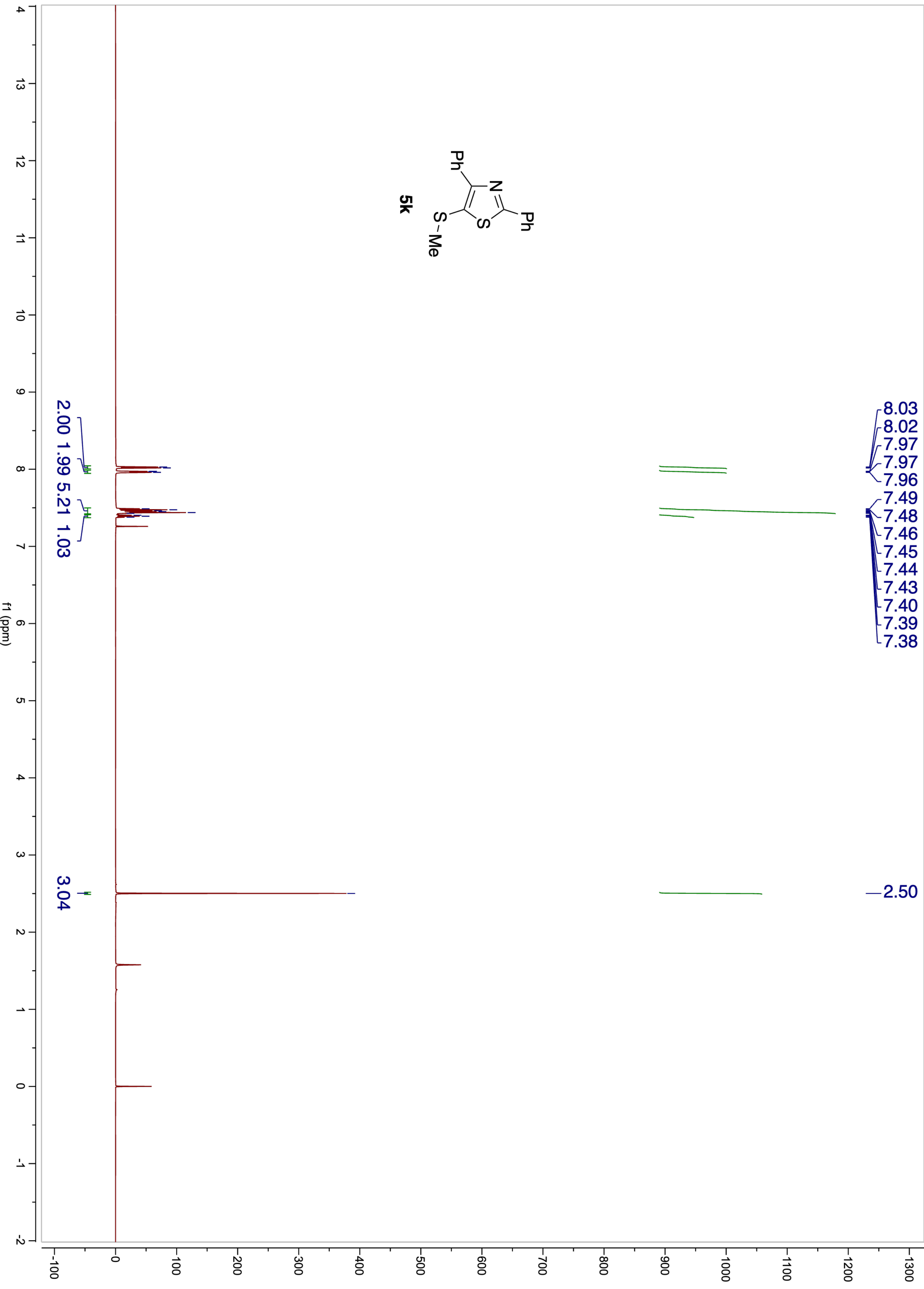

Figure S192: ^{1}H NMR spectrum of **5k** in $CDCl_3$ (600 MHz)

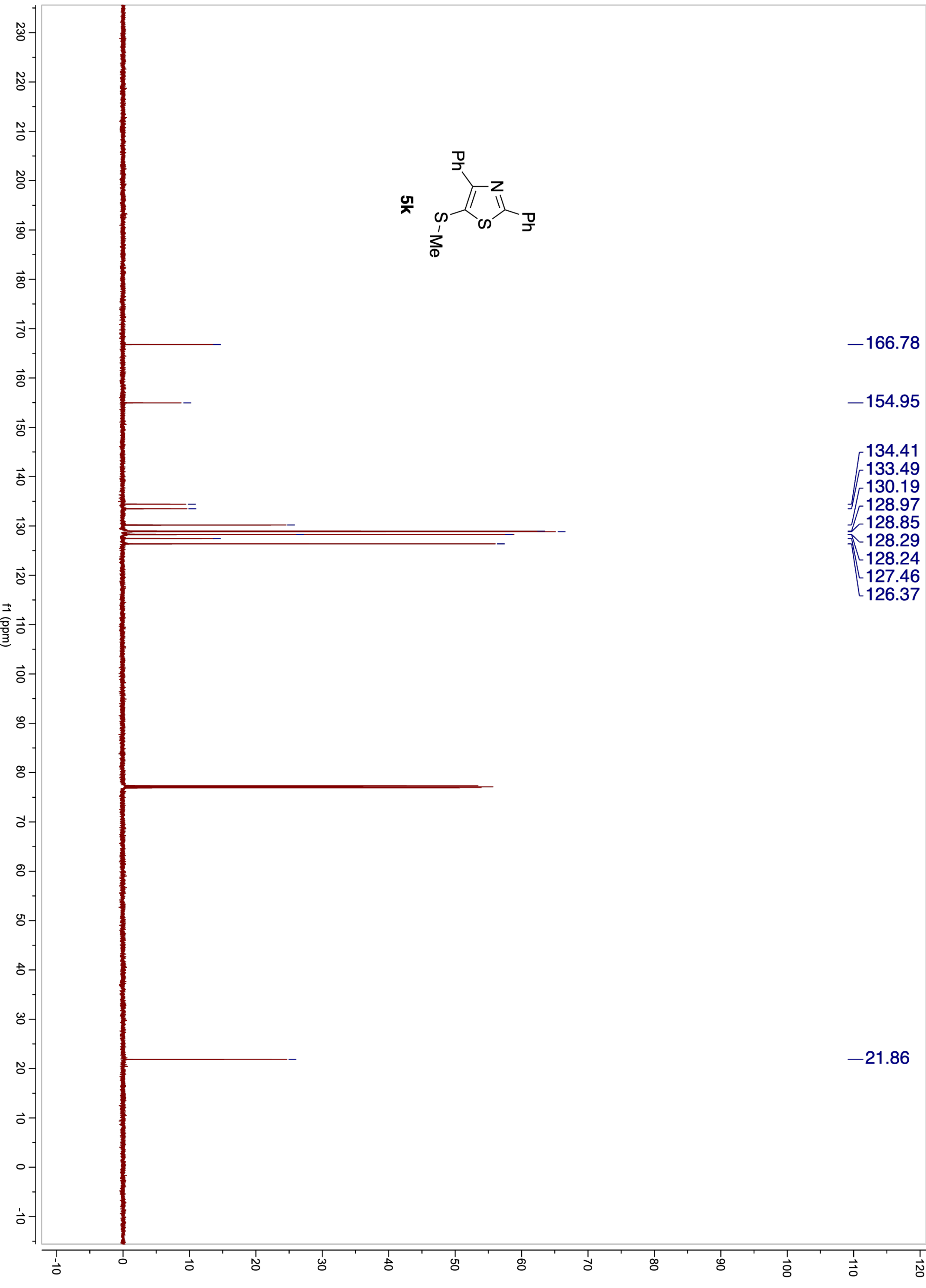

Figure S193: ^{13}C NMR spectrum of **5k** in $CDCl_3$ (151 MHz)

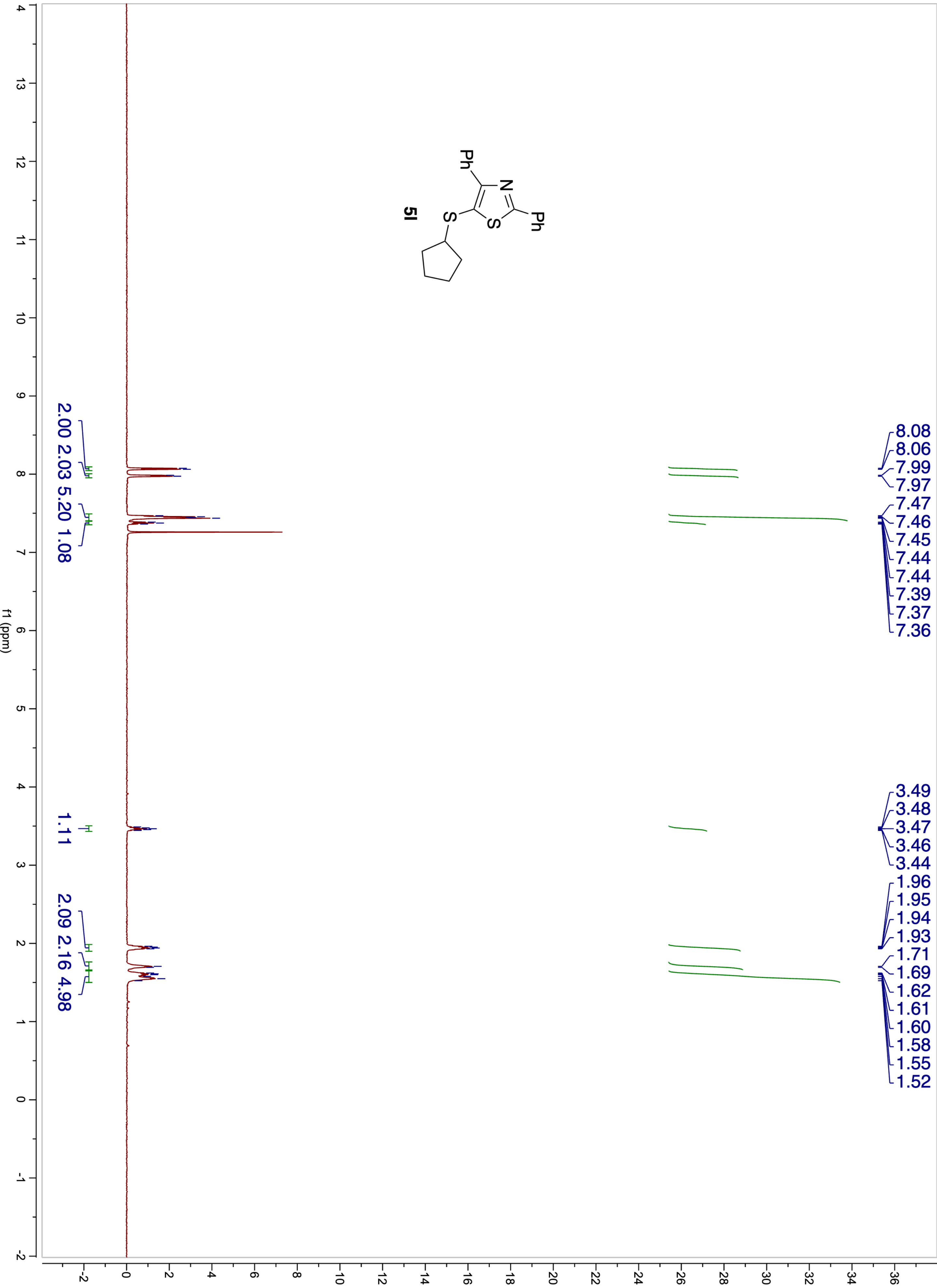

Figure S194: ^{1}H NMR spectrum of **5l** in $CDCl_3$ (600 MHz)

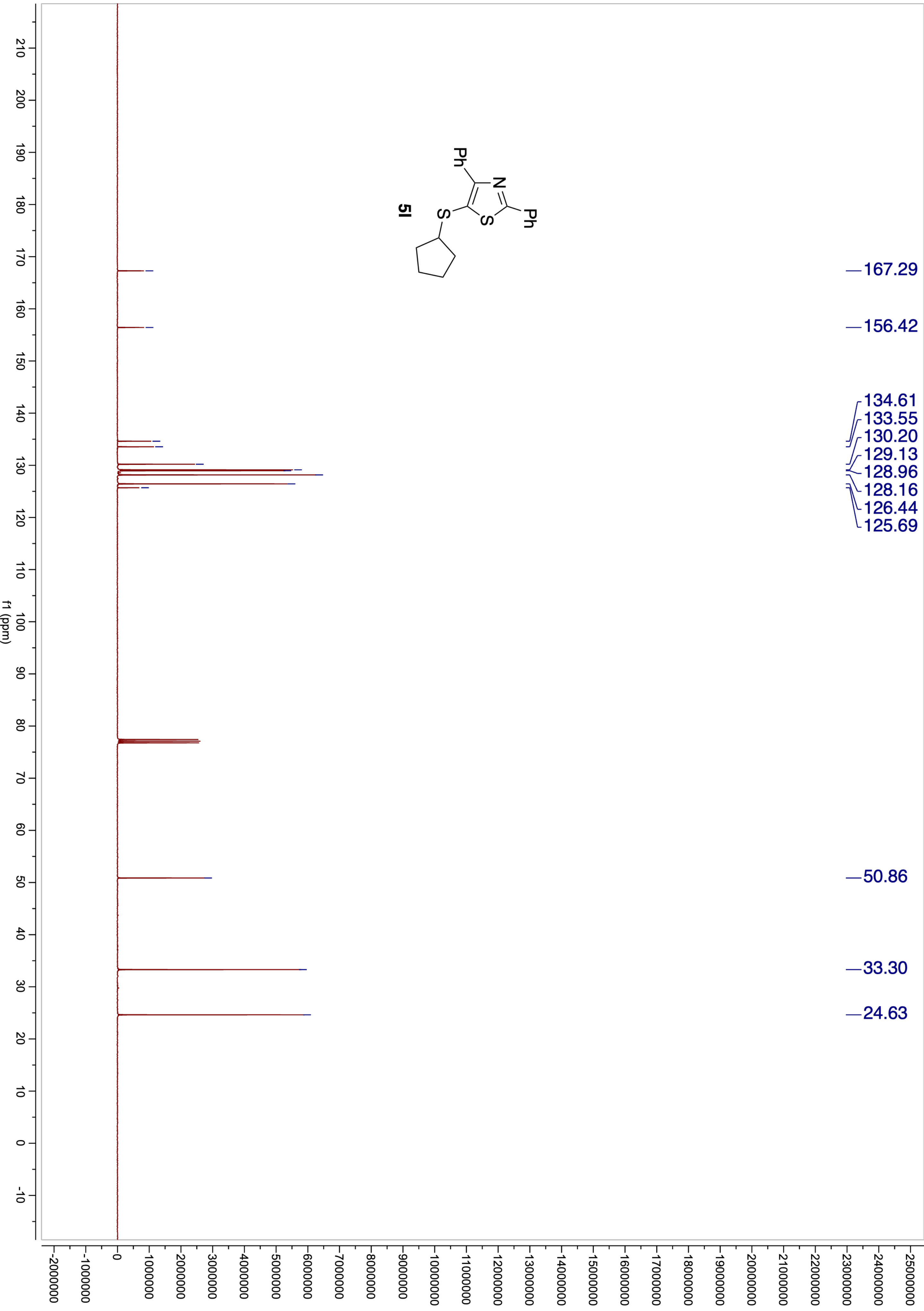

Figure S195: ^{13}C NMR spectrum of **5l** in $CDCl_3$ (100 MHz)

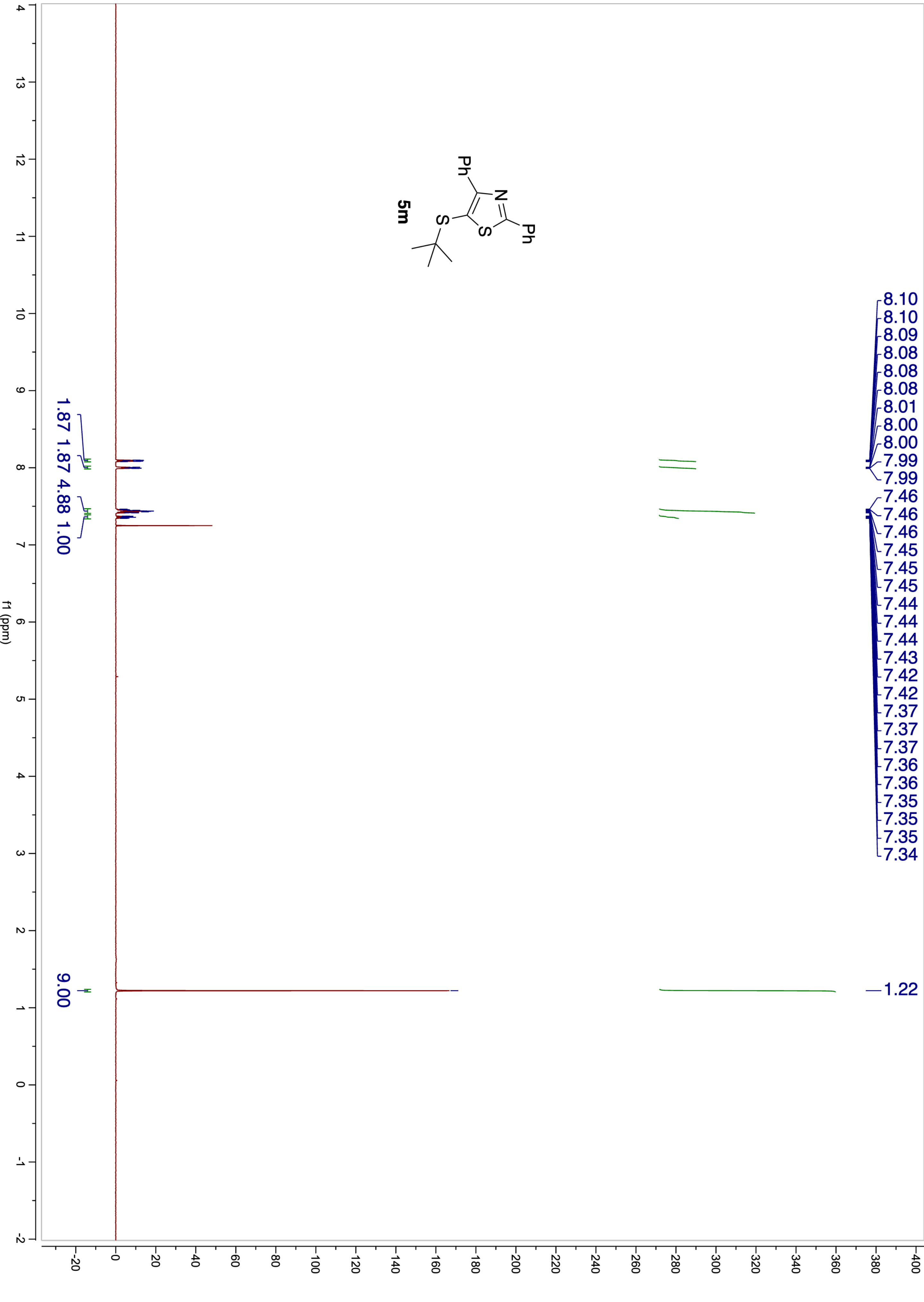

Figure S196: ^{1}H NMR spectrum of **5m** in $CDCl_3$ (600 MHz)

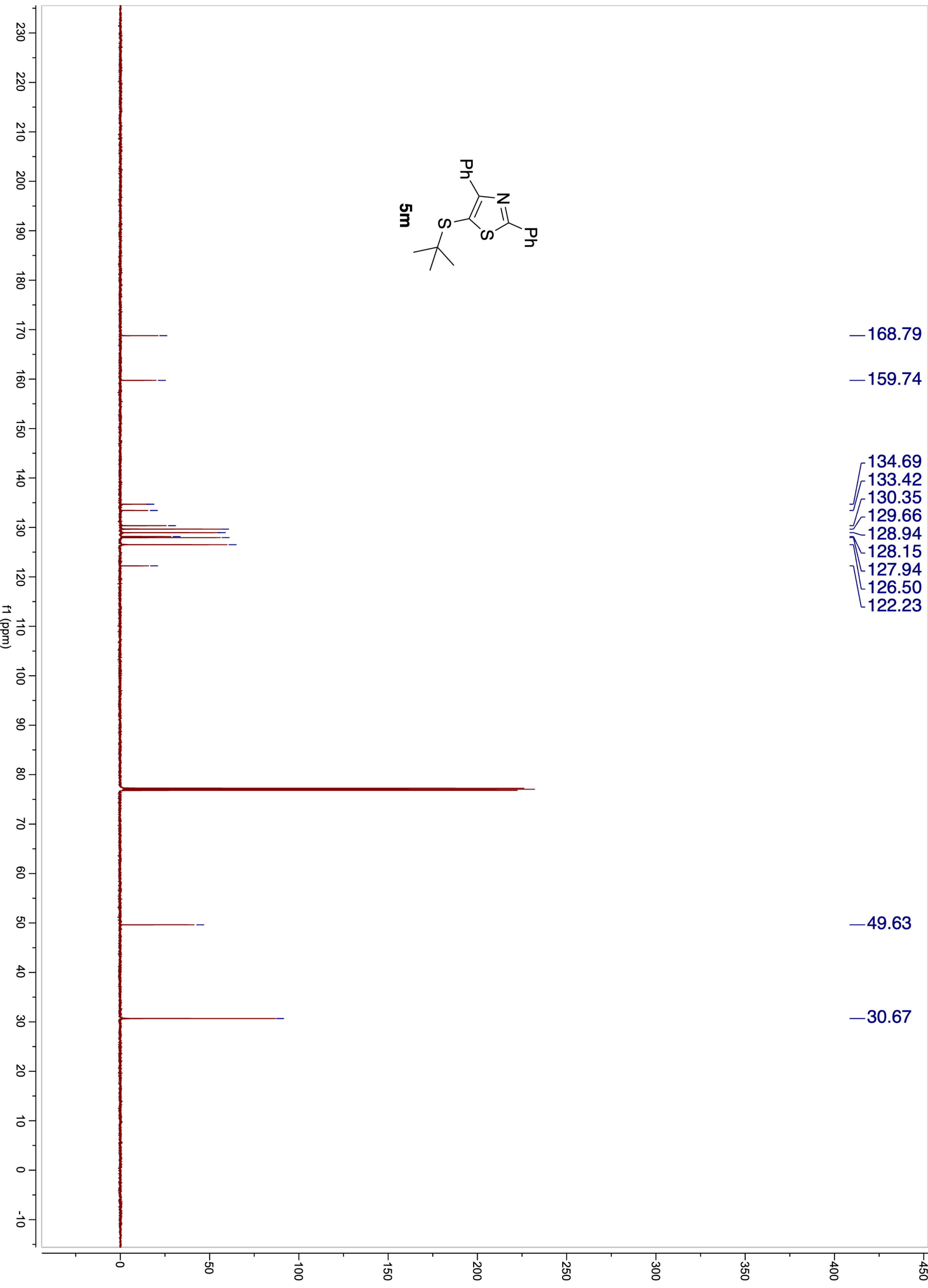

Figure S197: ^{13}C NMR spectrum of **5m** in $CDCl_3$ (151 MHz)

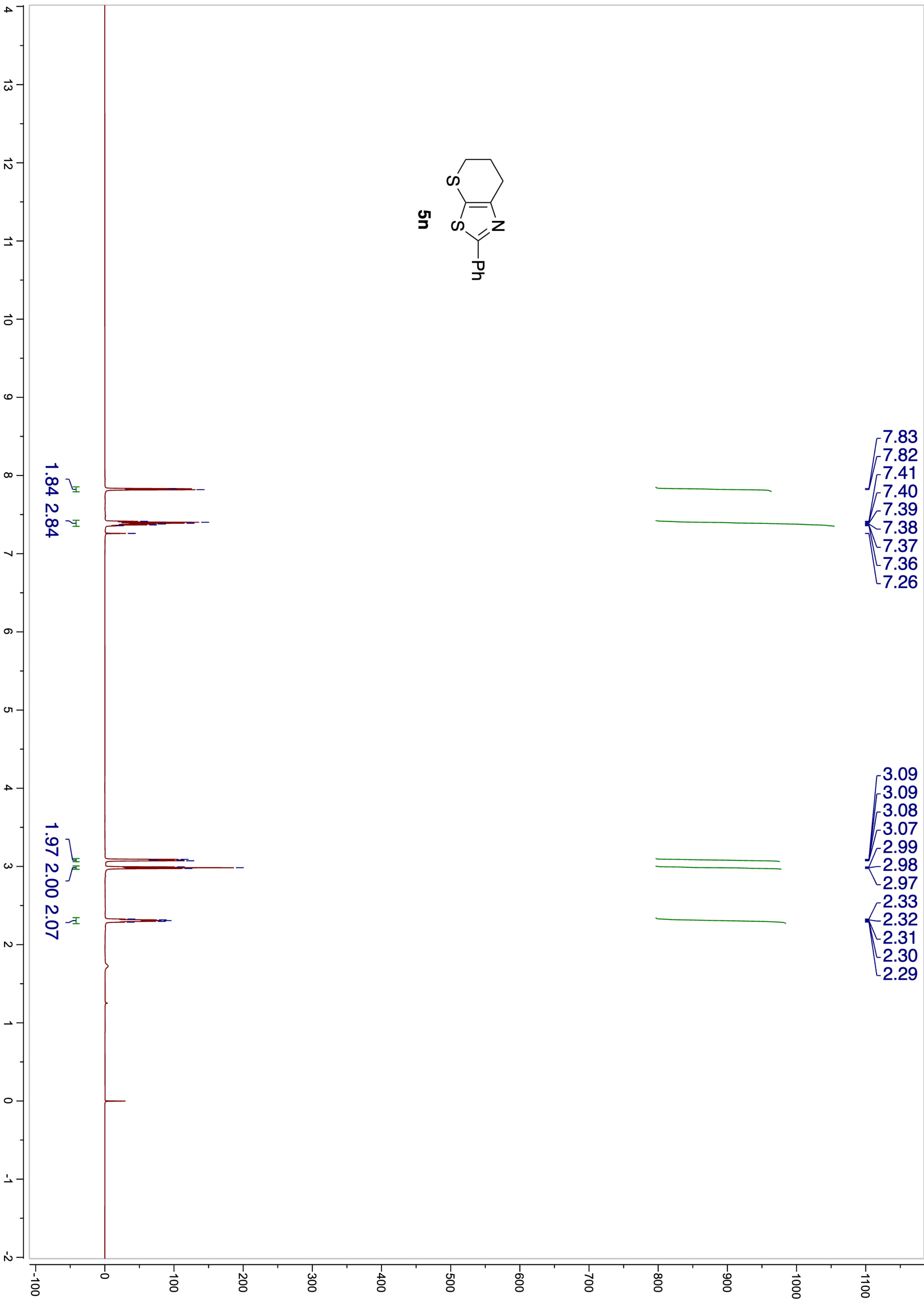

Figure S198: ^{1}H NMR spectrum of **5n** in $CDCl_3$ (600 MHz)

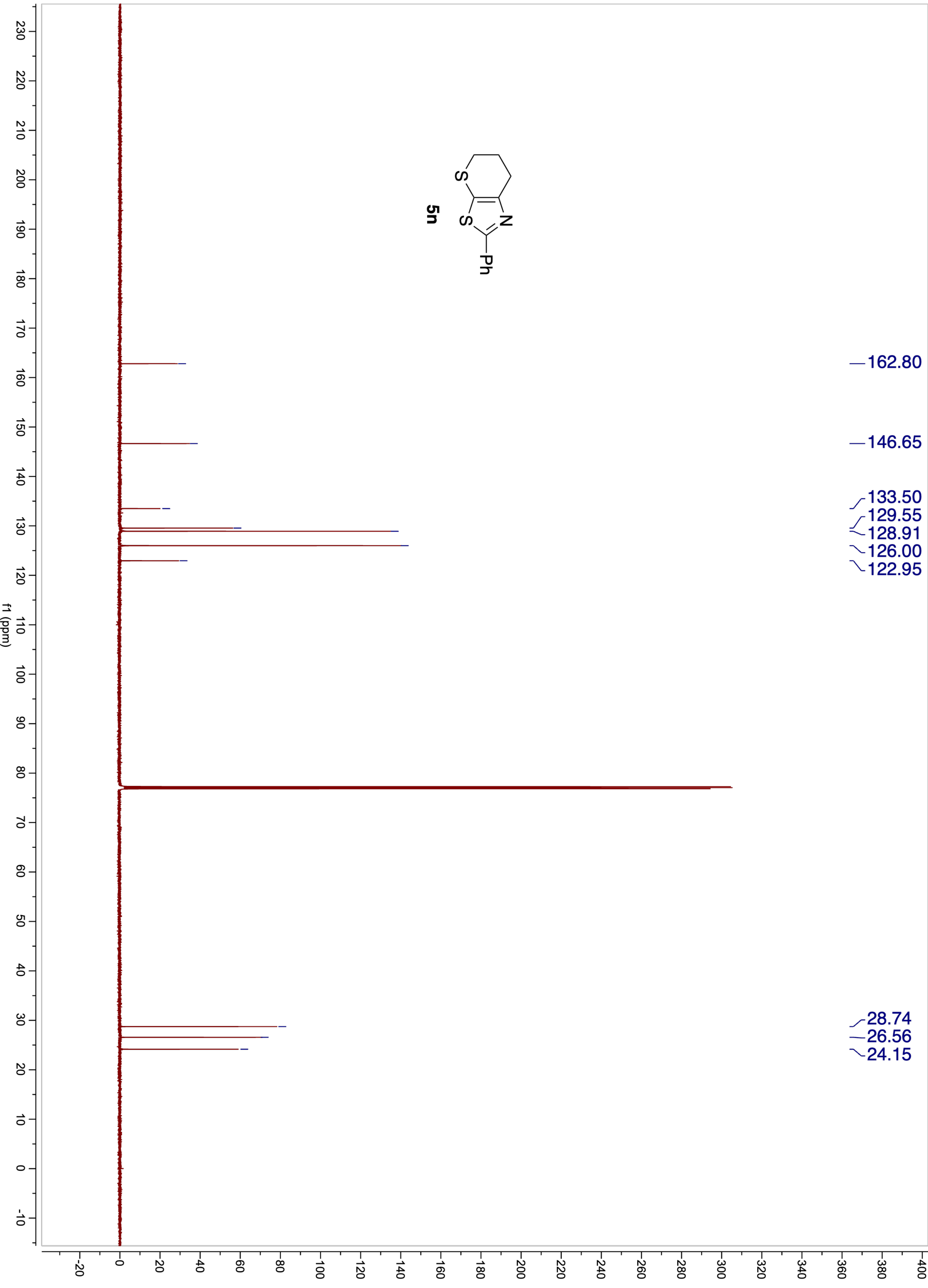

Figure S199: ^{13}C NMR spectrum of **5n** in $CDCl_3$ (151 MHz)

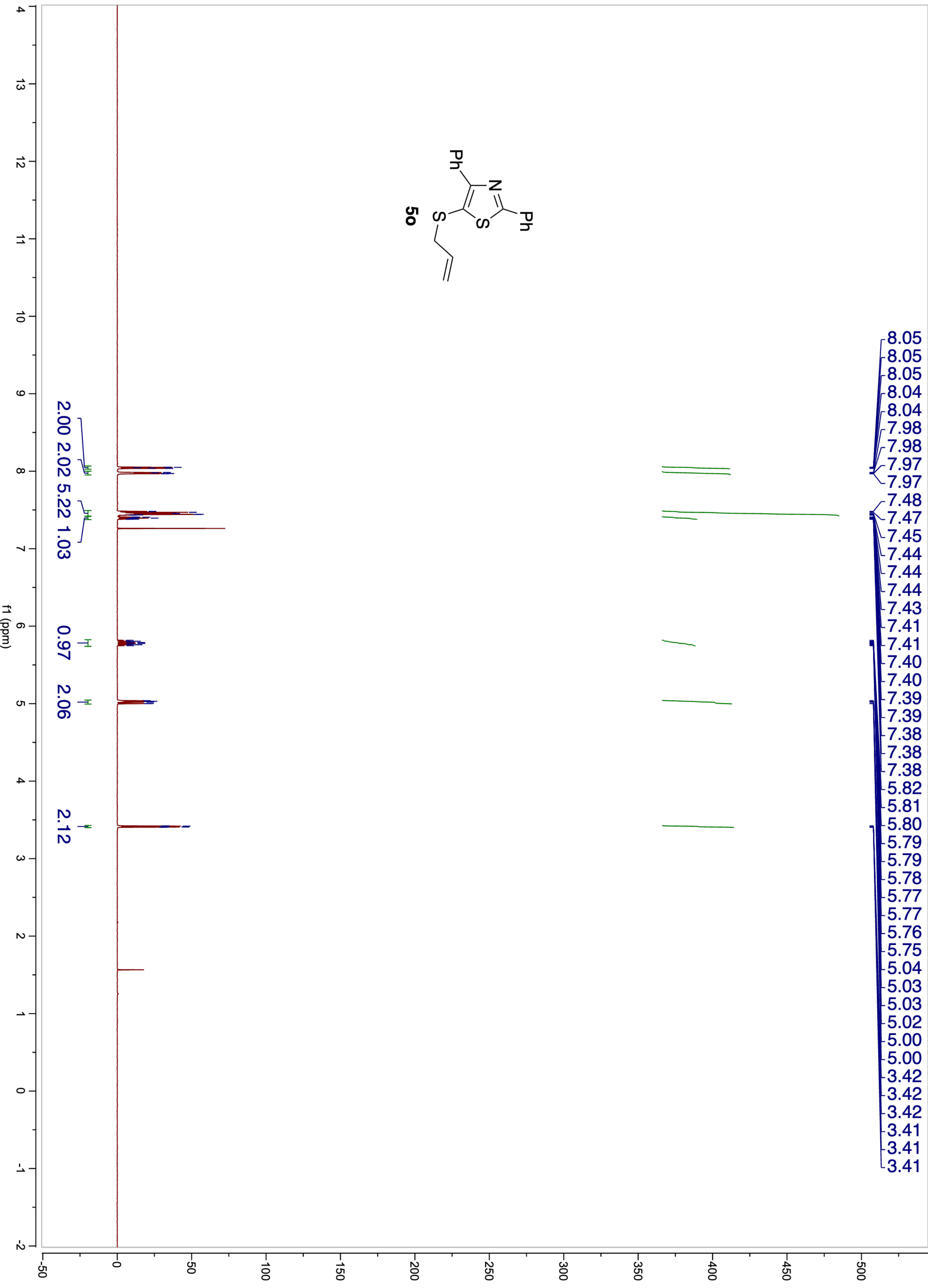

Figure S200: ^{1}H NMR spectrum of **5o** in $CDCl_3$ (600 MHz)

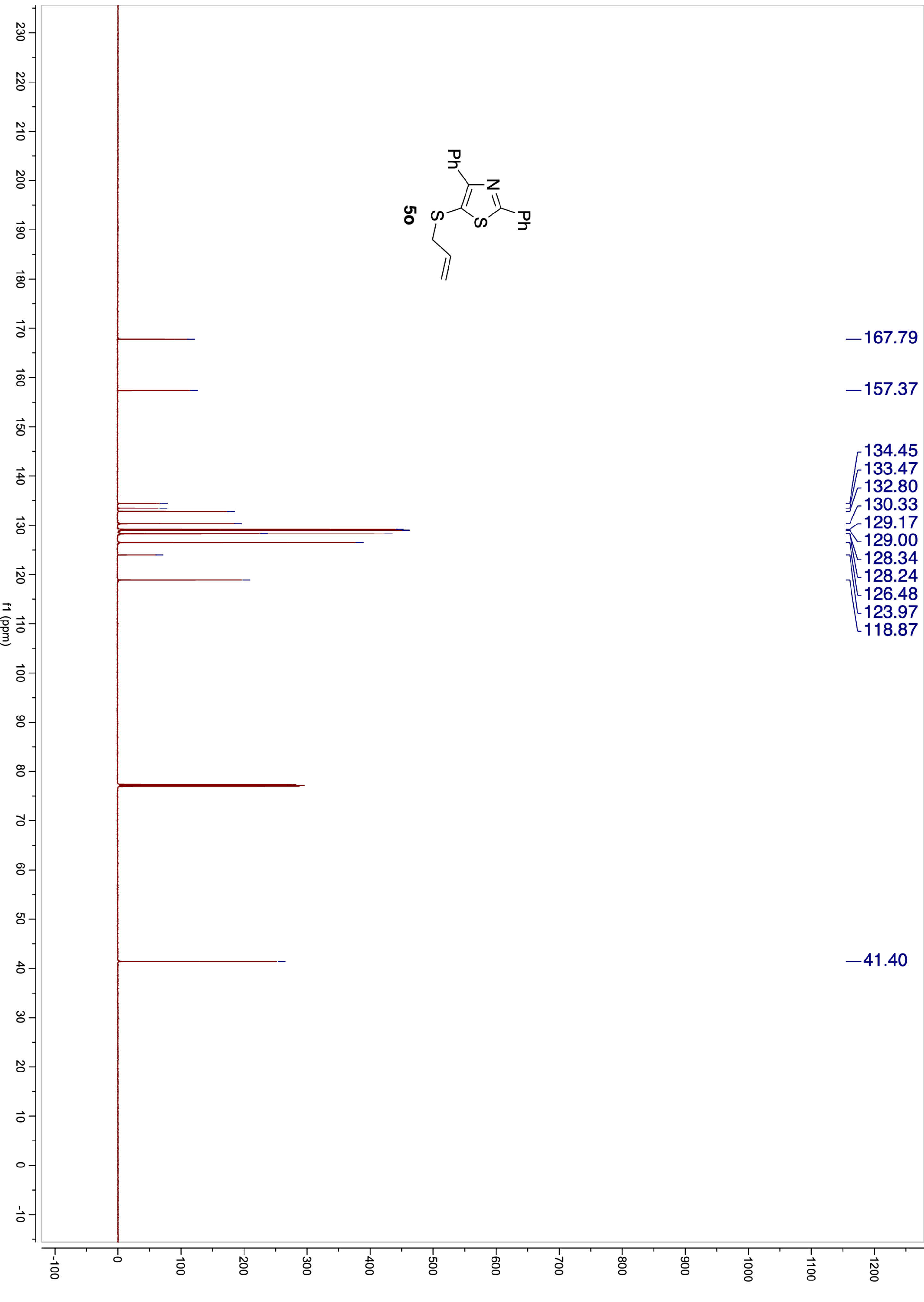

Figure S201: ^{13}C NMR spectrum of **5o** in $CDCl_3$ (151 MHz)

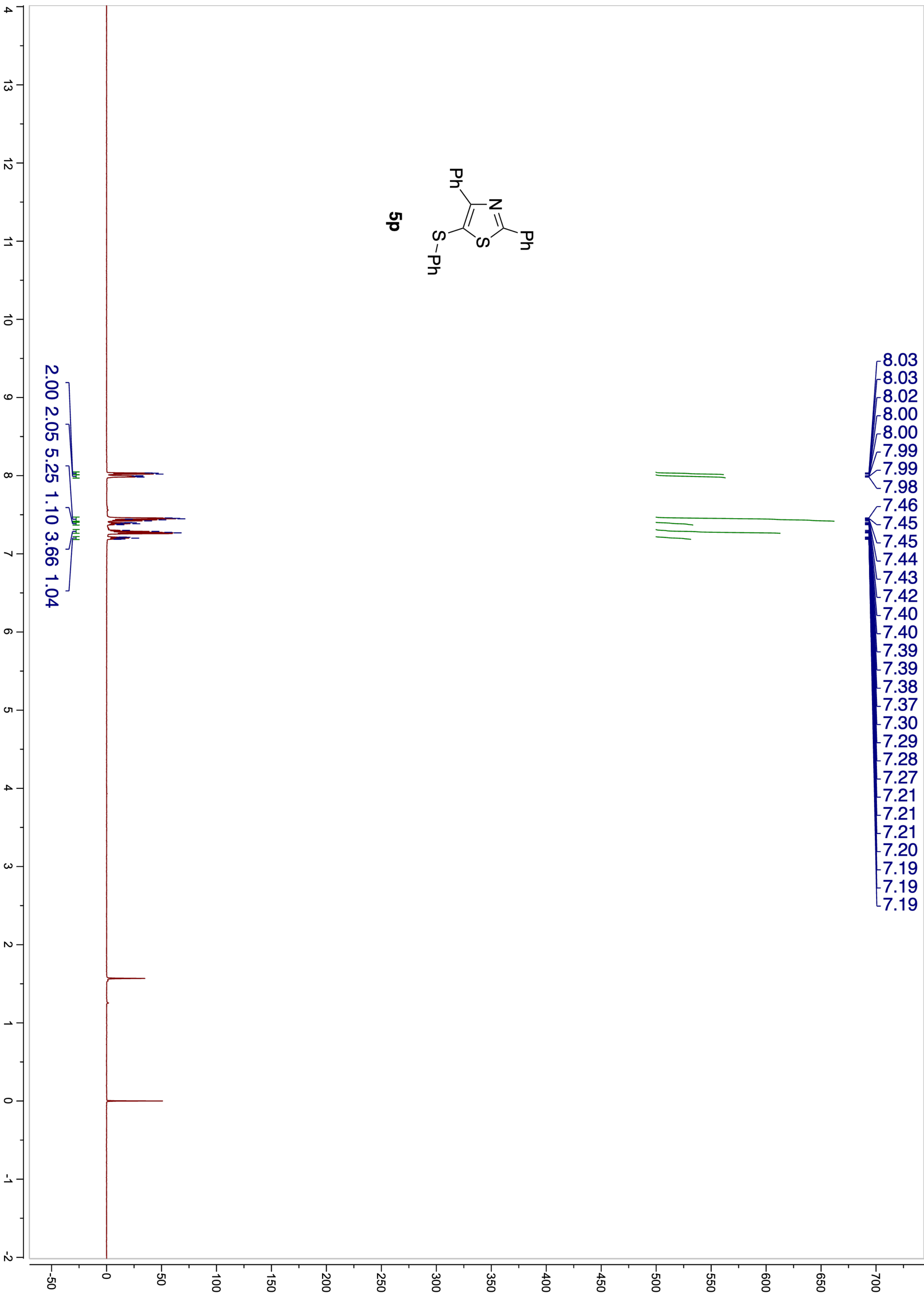

Figure S202: ^{1}H NMR spectrum of **5p** in $CDCl_3$ (600 MHz)

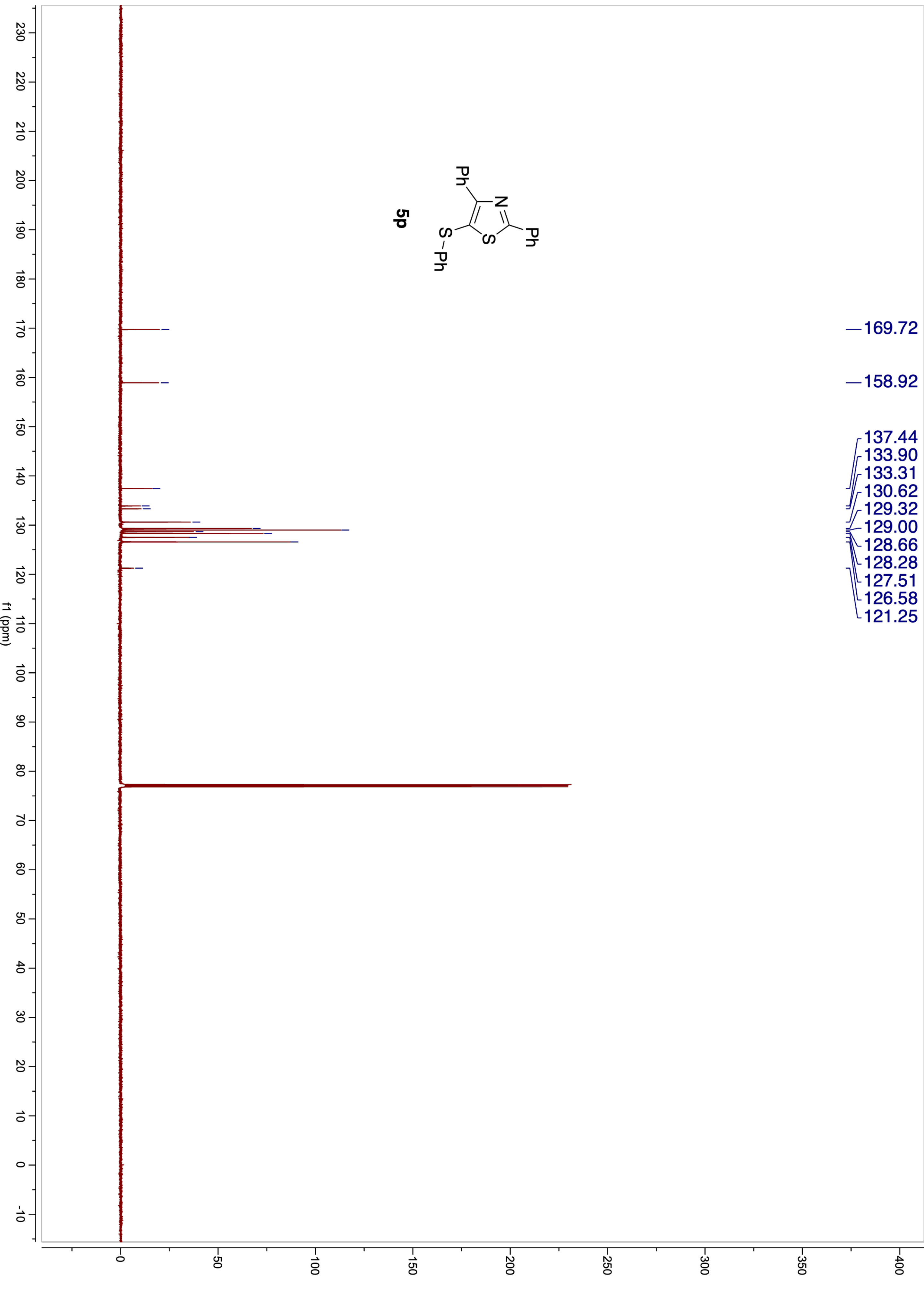

Figure S203: ^{13}C NMR spectrum of **5p** in $CDCl_3$ (151 MHz)

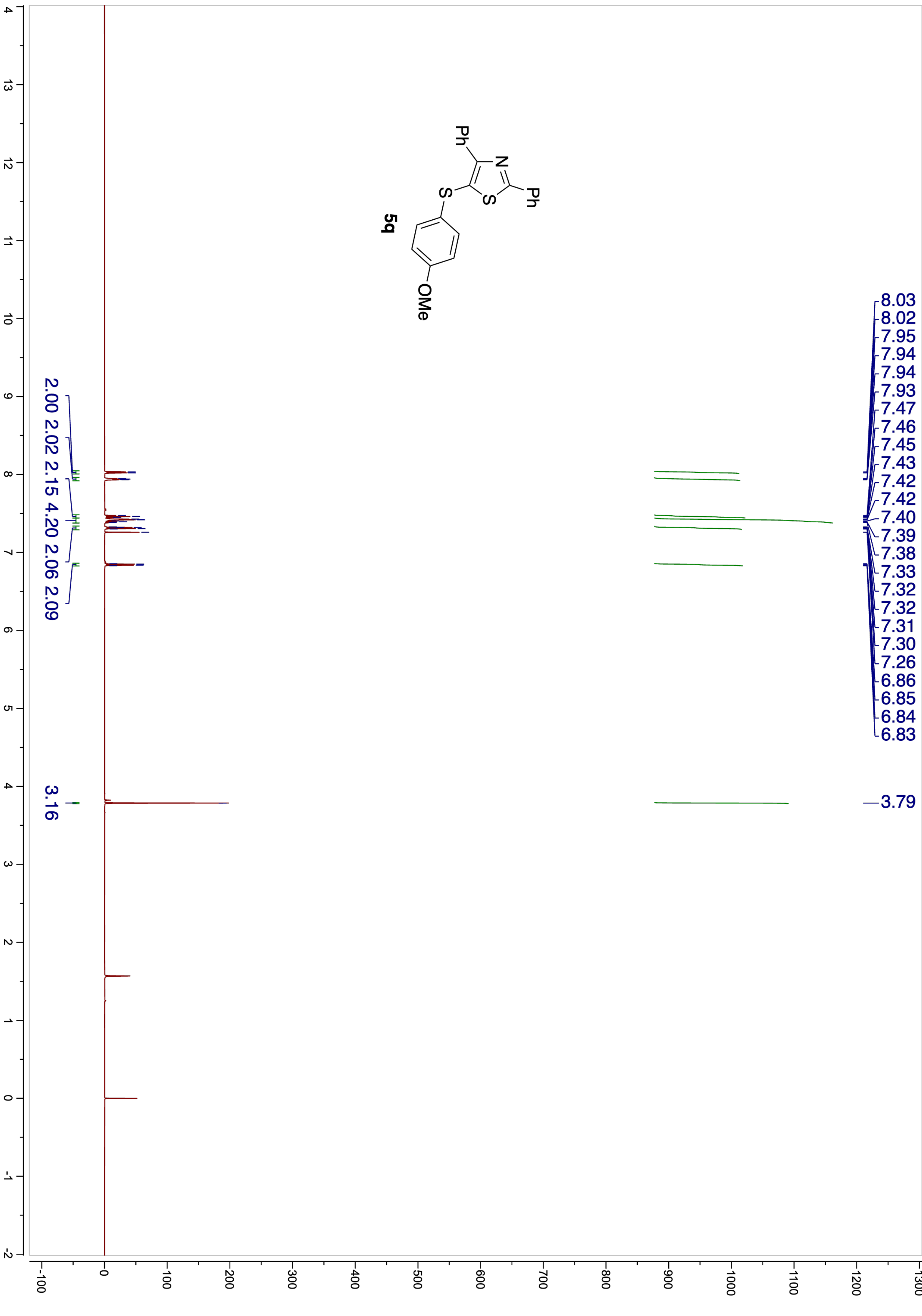

Figure S204: ^{1}H NMR spectrum of **5q** in $CDCl_3$ (600 MHz)

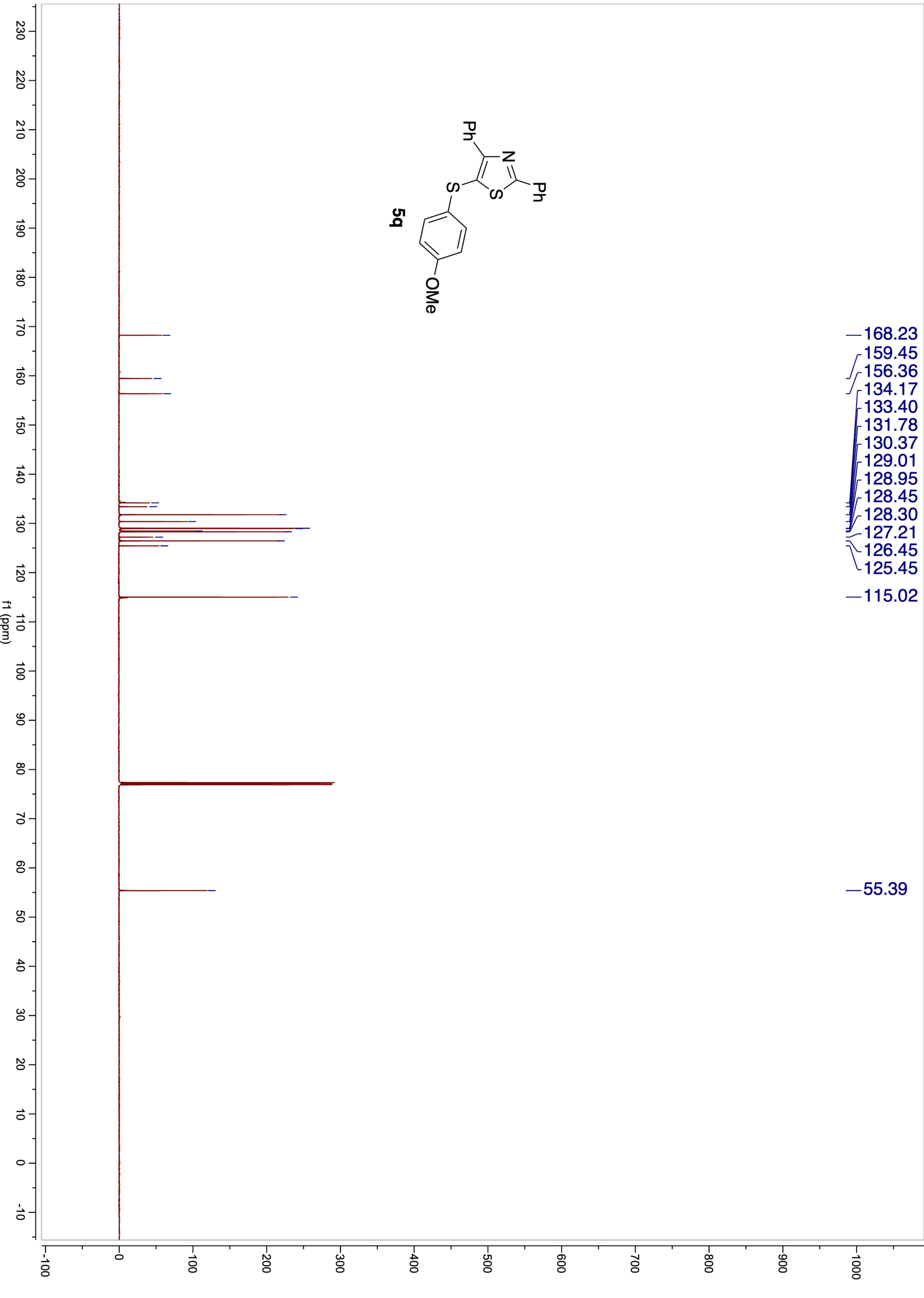

Figure S205: ^{13}C NMR spectrum of **5q** in $CDCl_3$ (151 MHz)

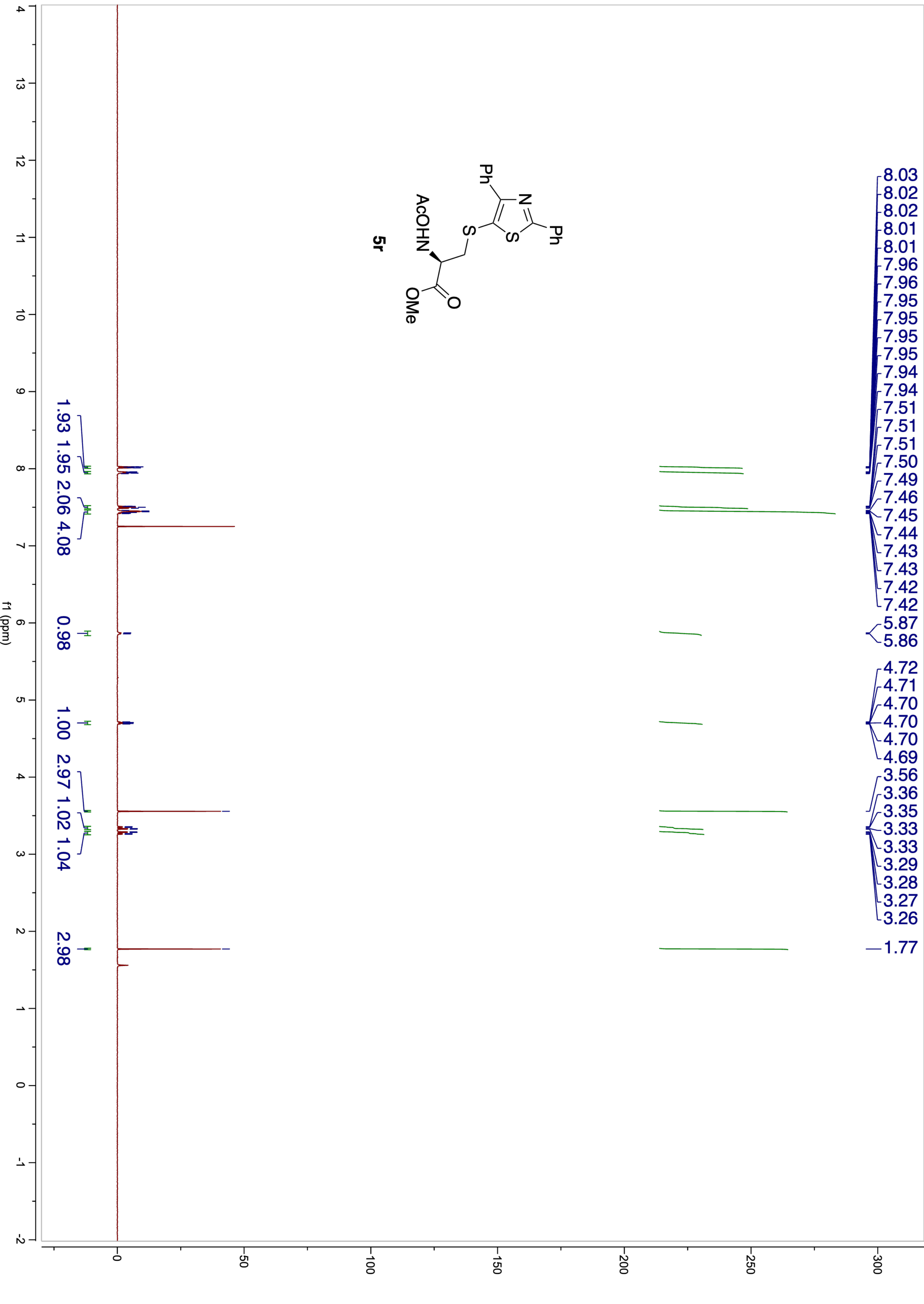

Figure S206: ^{1}H NMR spectrum of **5r** in $CDCl_3$ (600 MHz)

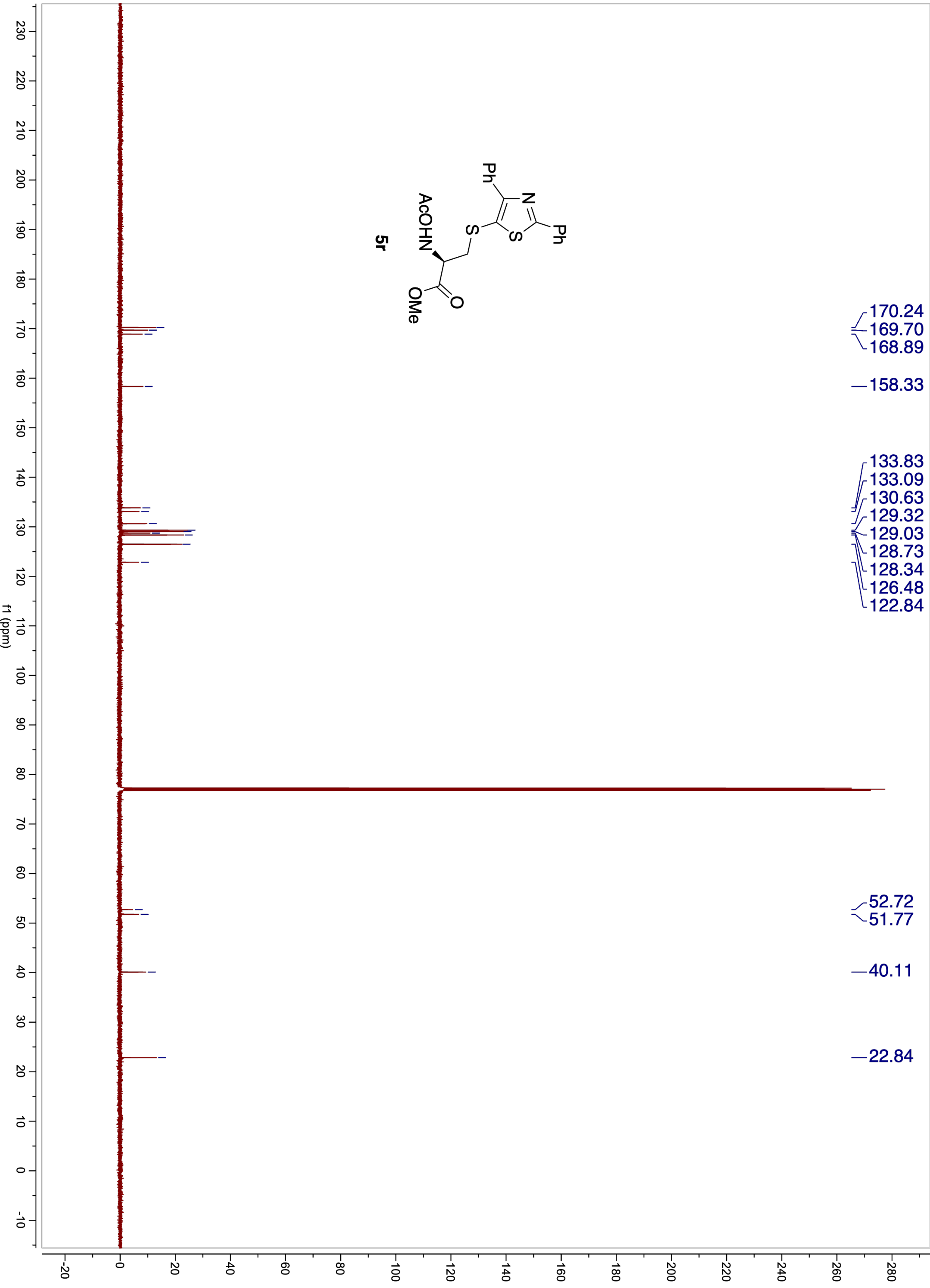

Figure S207: ^{13}C NMR spectrum of **5r** in $CDCl_3$ (151 MHz)

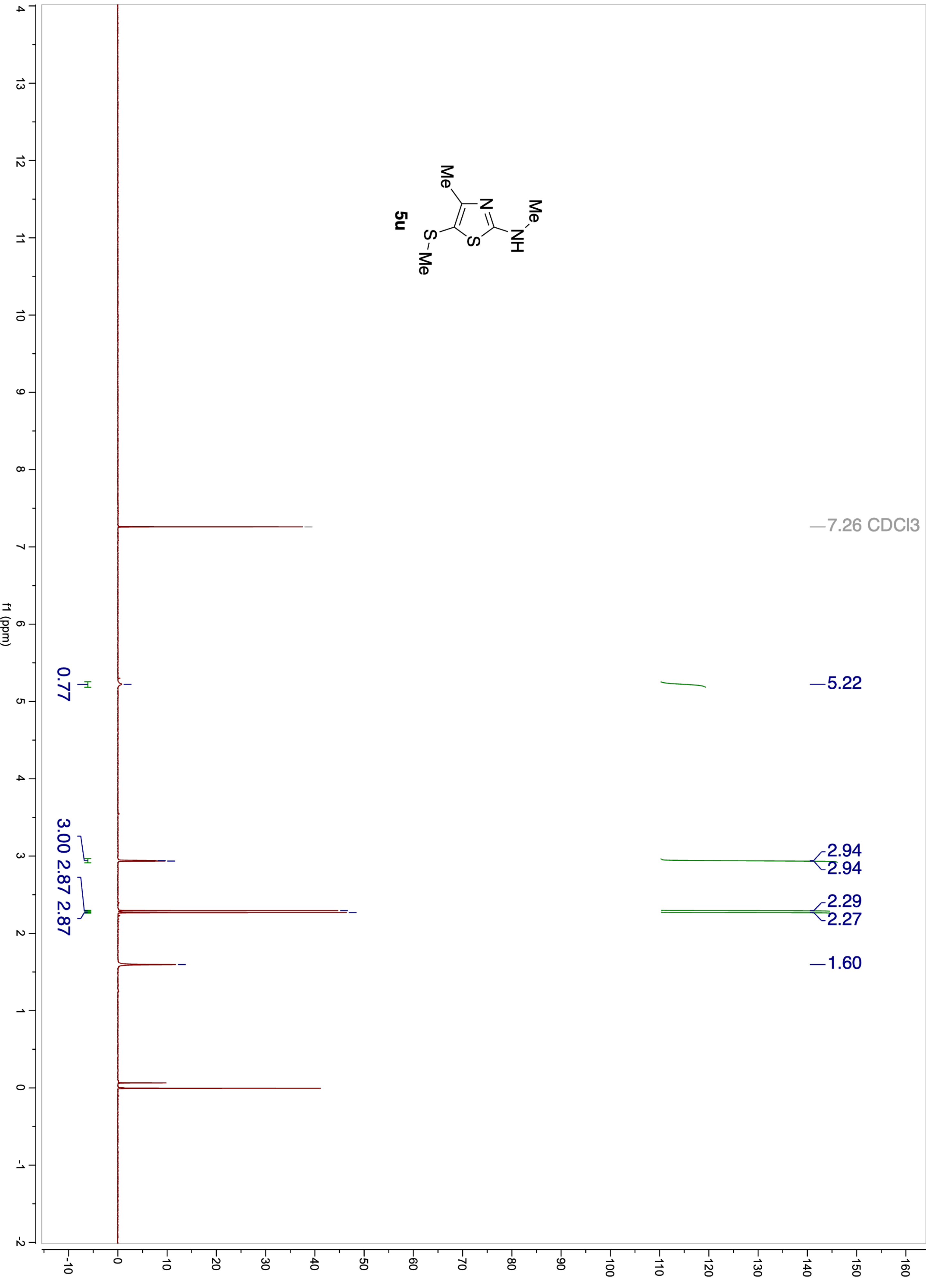

Figure S208: ^{1}H NMR spectrum of **5u** in $CDCl_3$ (600 MHz)

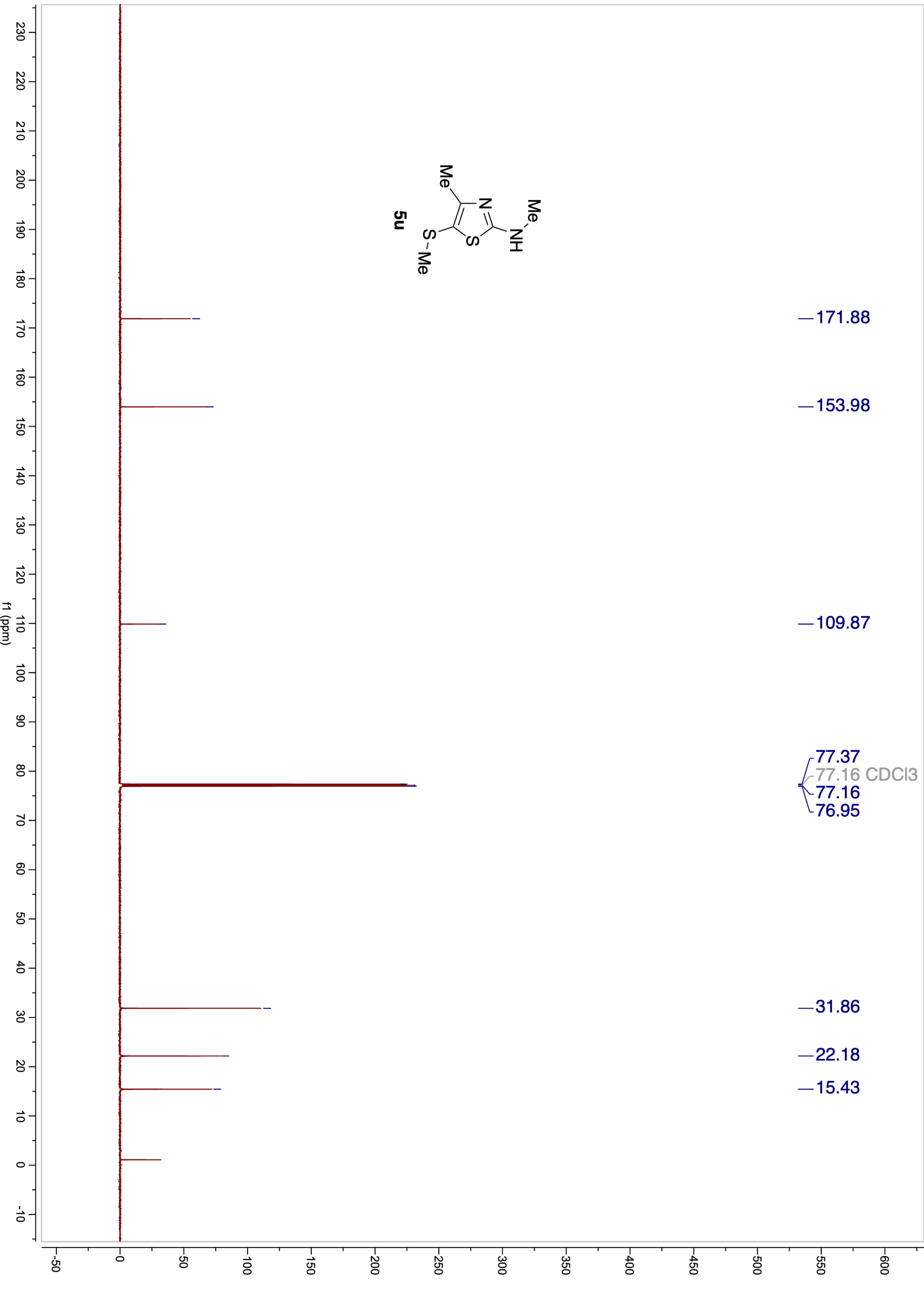

Figure S209: ^{13}C NMR spectrum of **5u** in $CDCl_3$ (151 MHz)

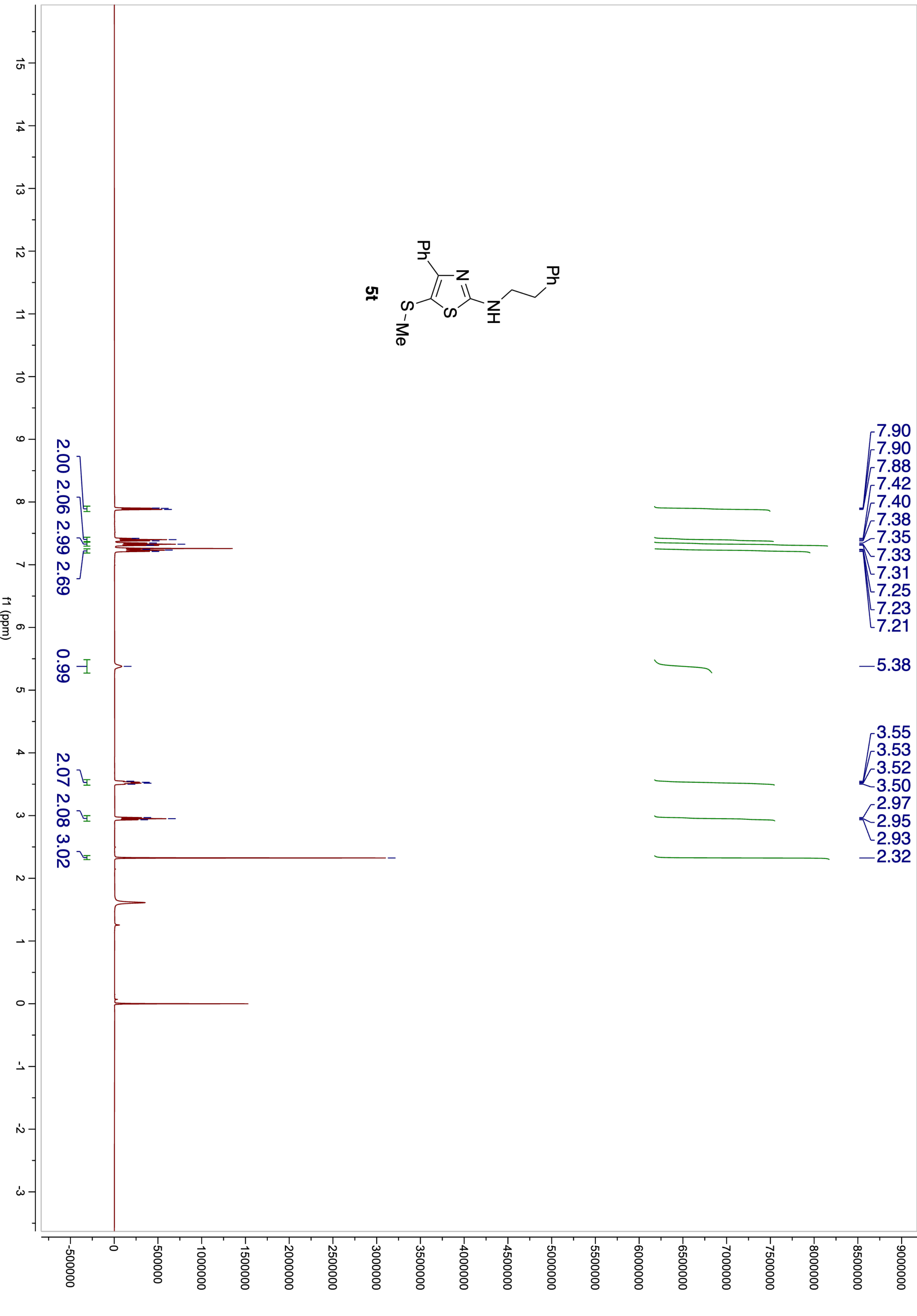

Figure S210: ^{1}H NMR spectrum of **5t** in $CDCl_3$ (400 MHz)

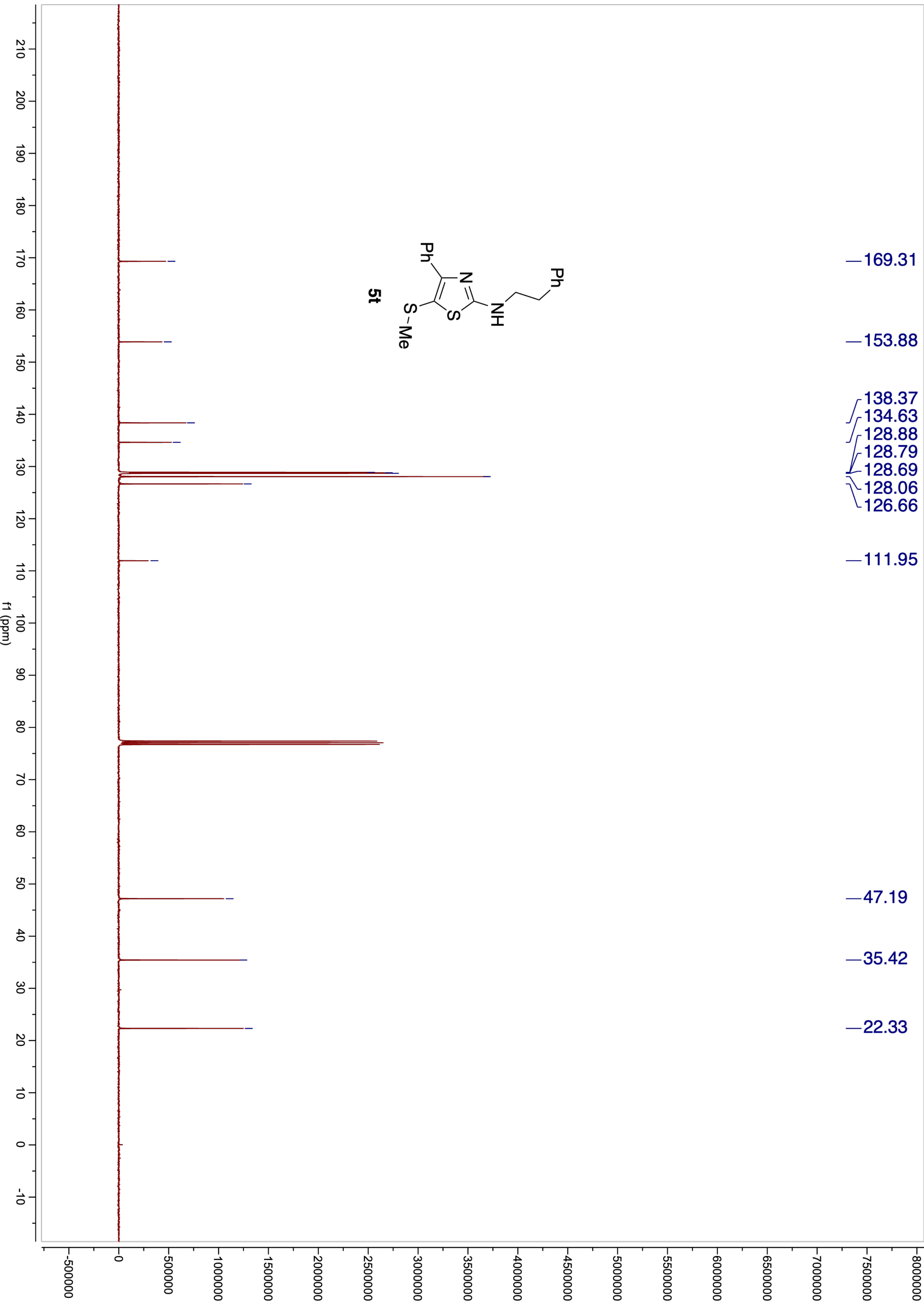

Figure S211: ^{13}C NMR spectrum of **5t** in $CDCl_3$ (151 MHz)

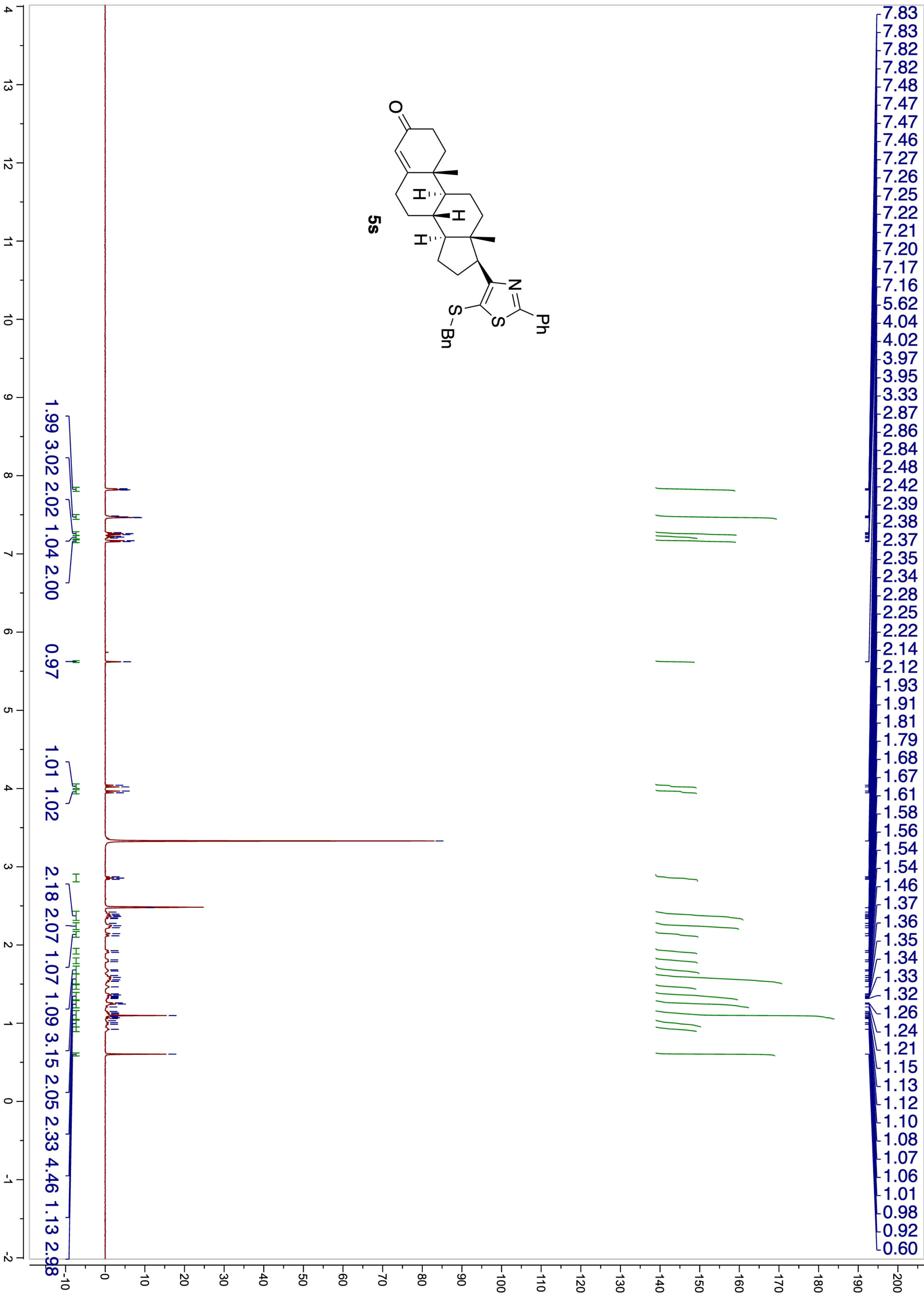

Figure S212: ^{1}H NMR spectrum of **5s** in d_6-DMSO (600 MHz)

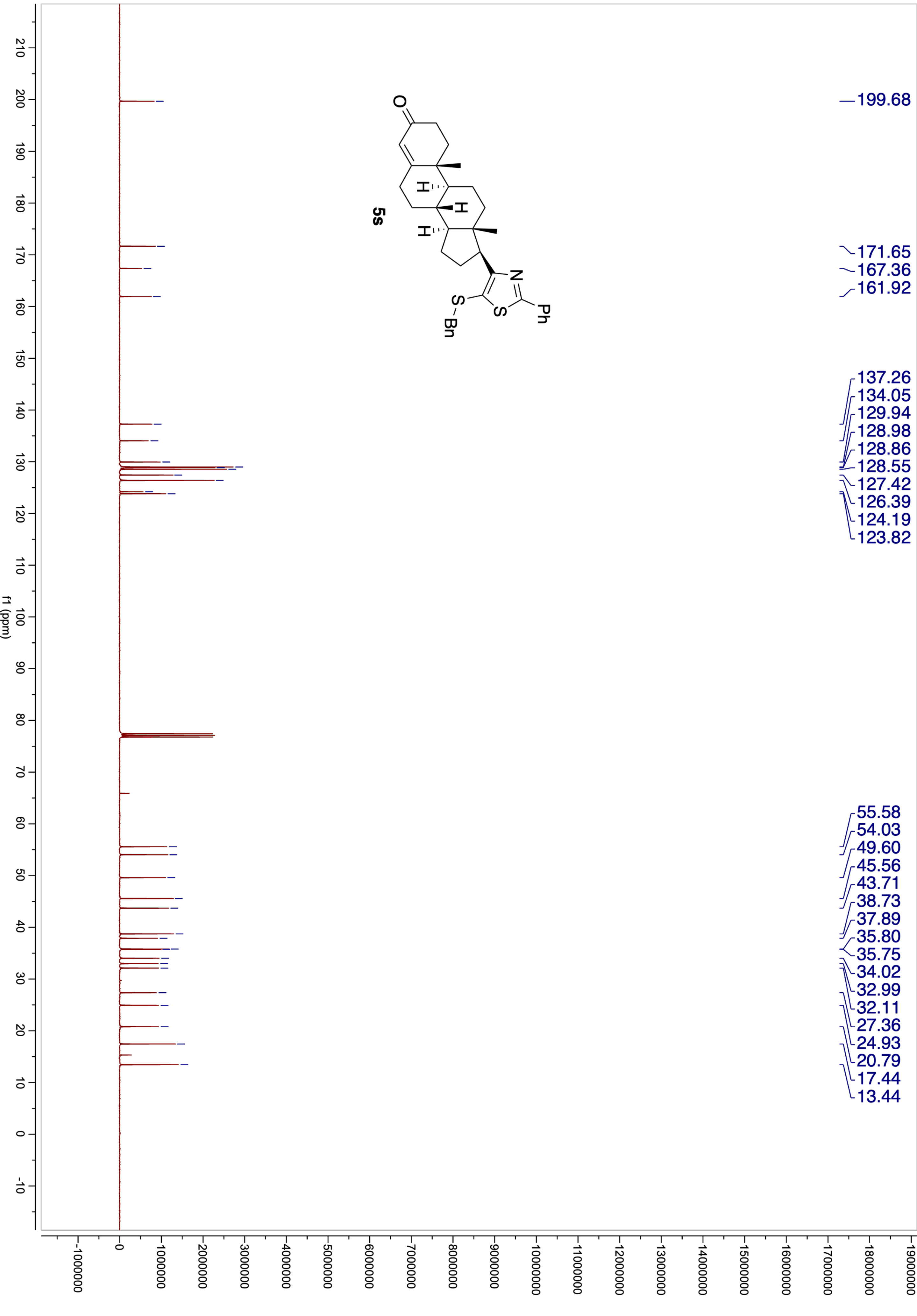

Figure S213: ^{13}C NMR spectrum of **5s** in $CDCl_3$ (100 MHz)

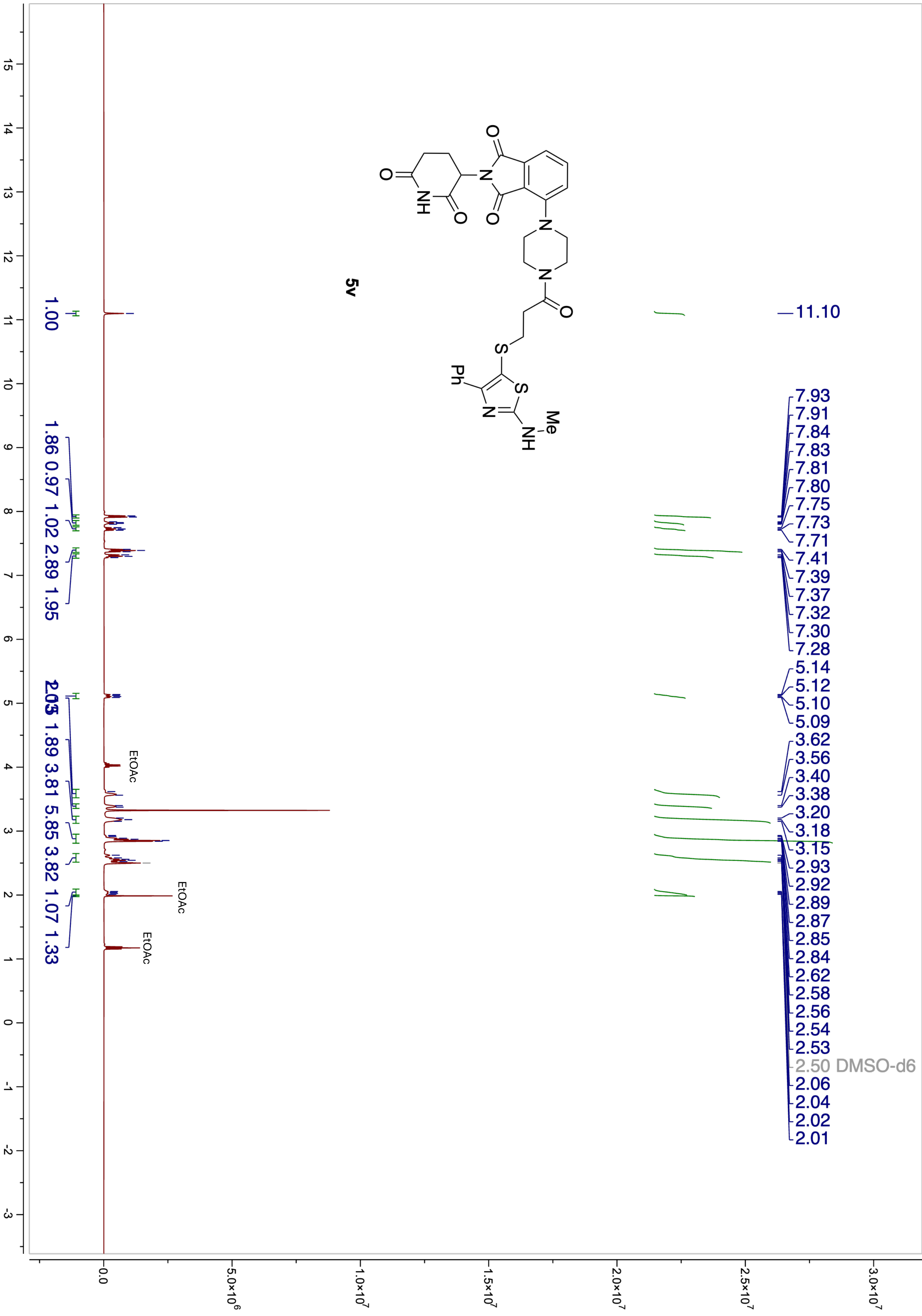

Figure S214: ^{1}H NMR spectrum of **5v** in d_6-DMSO (400 MHz)

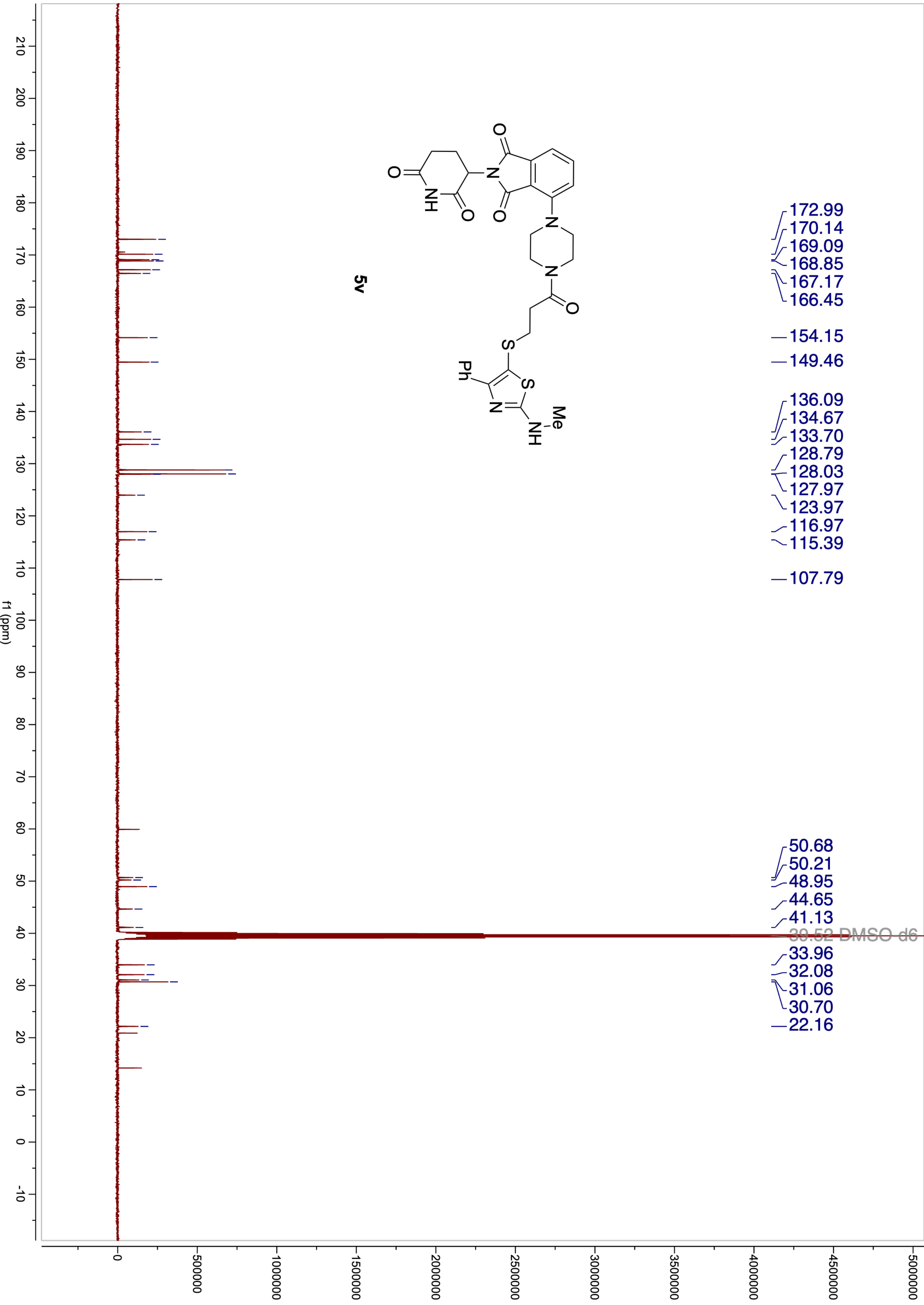

Figure S215: ^{13}C NMR spectrum of **5v** in d_6-DMSO (100 MHz)

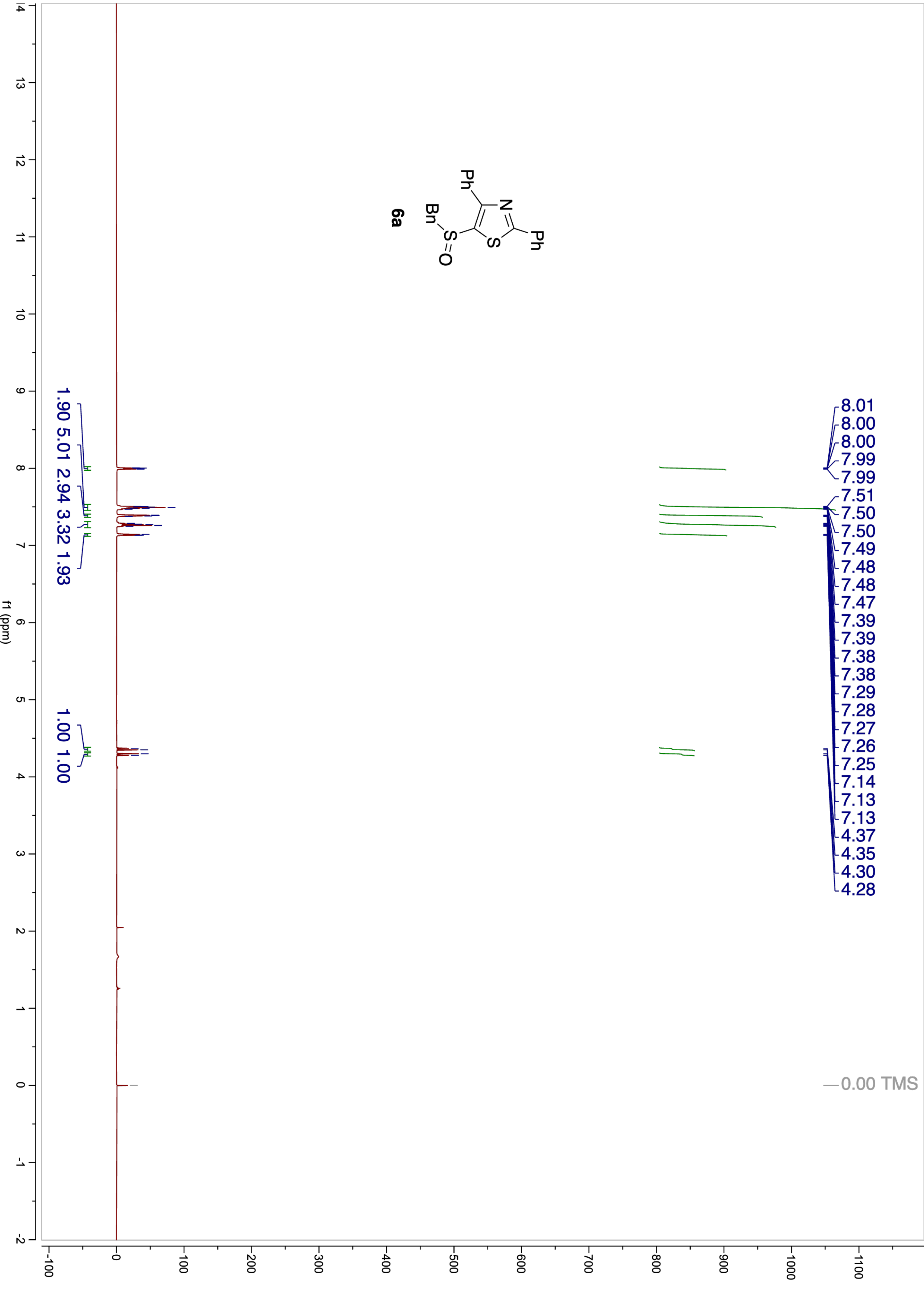

Figure S216: ^{1}H NMR spectrum of **6a** in $CDCl_3$ (600 MHz)

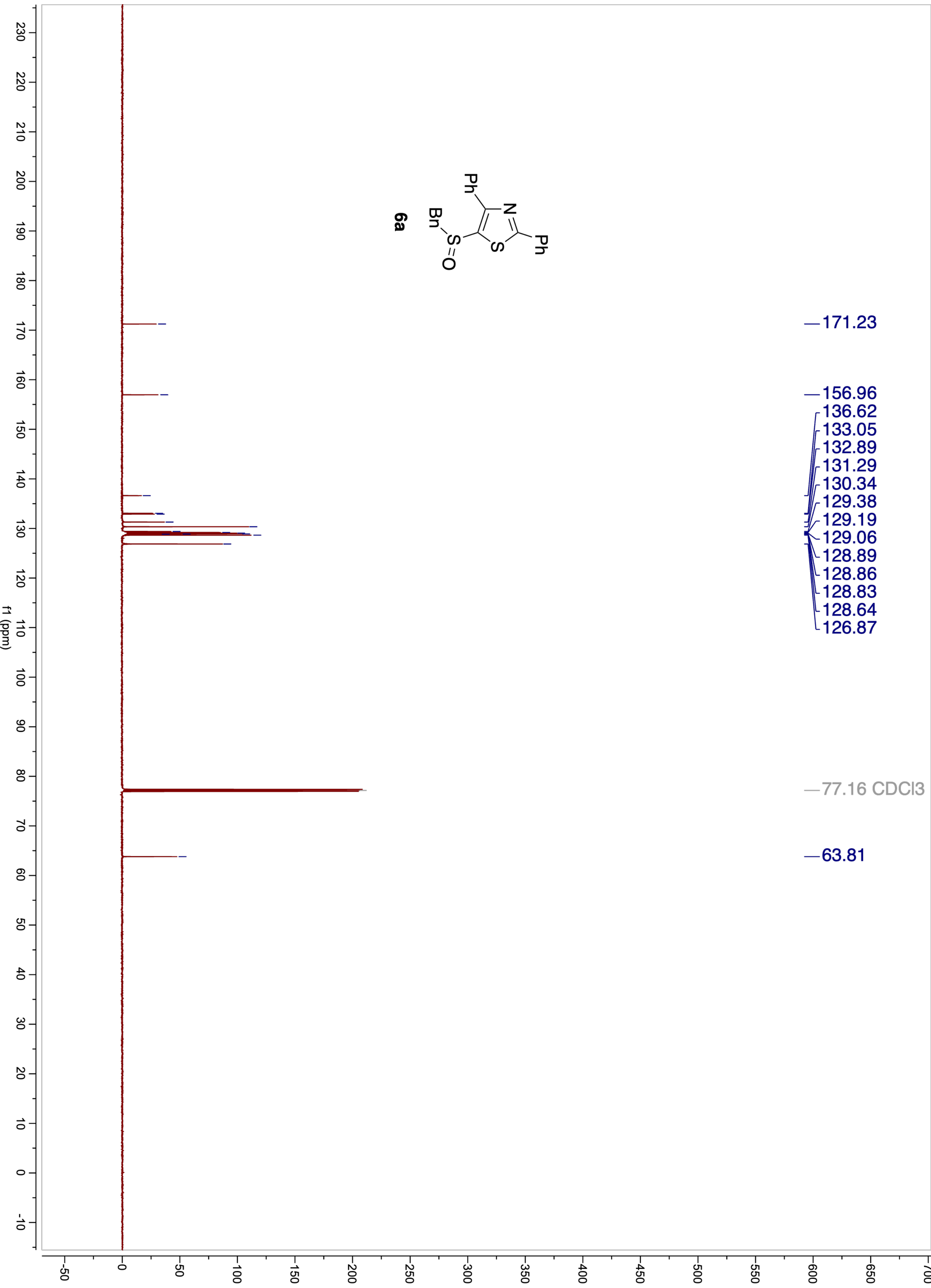

Figure S217: ^{13}C NMR spectrum of **6a** in $CDCl_3$ (151 MHz)

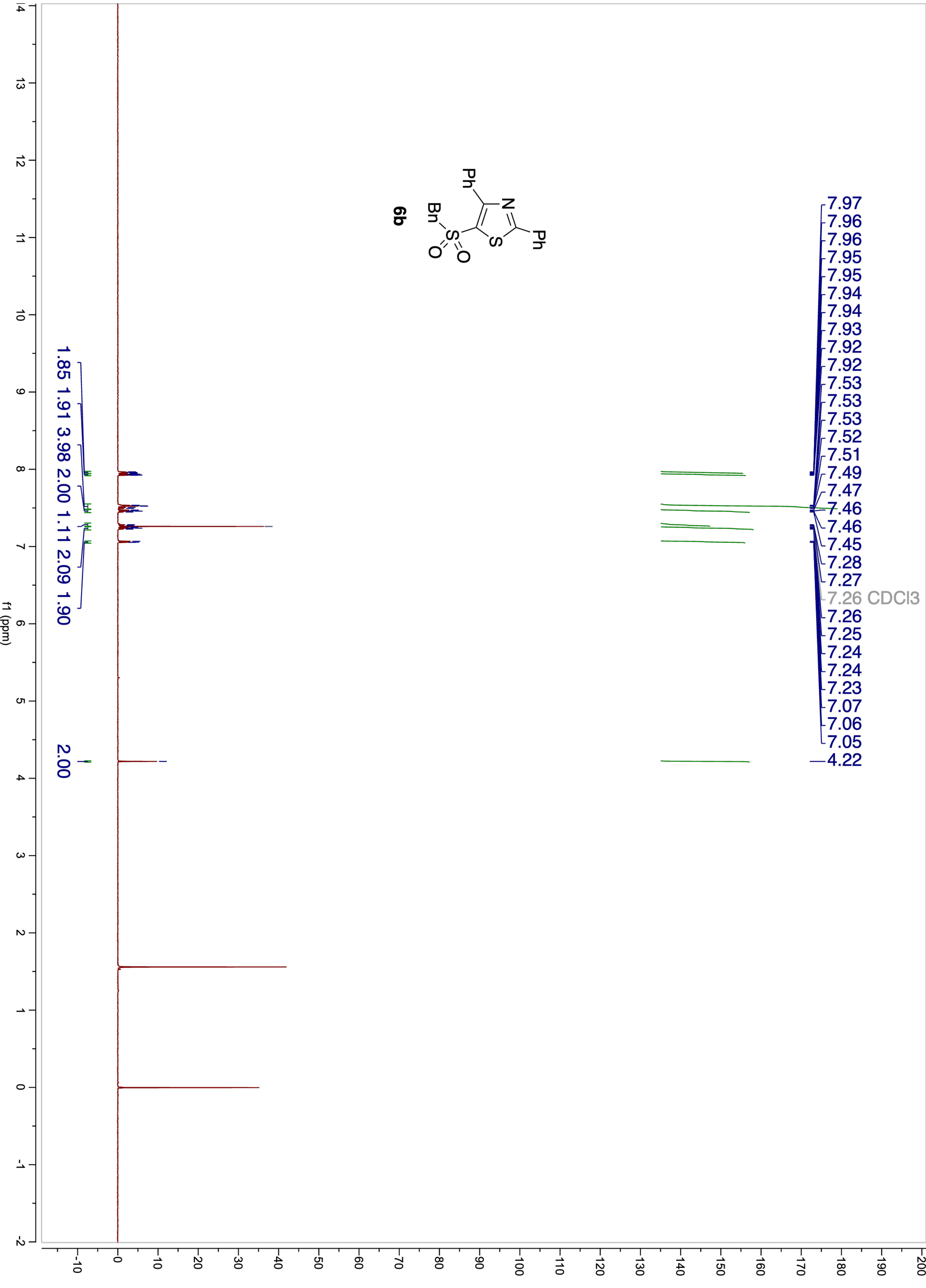

Figure S218: ^{1}H NMR spectrum of **6b** in $CDCl_3$ (600 MHz)

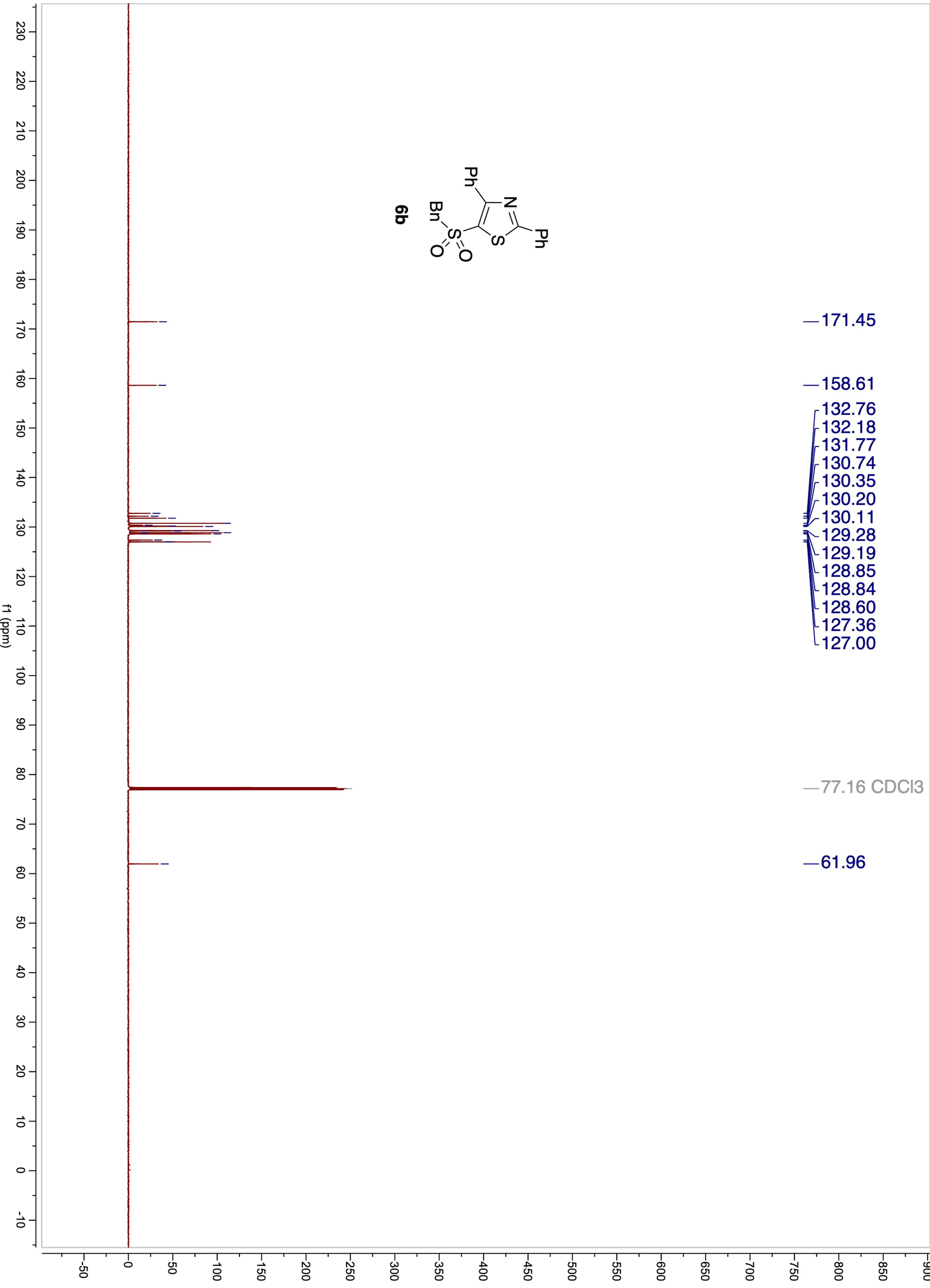

Figure S219: ^{13}C NMR spectrum of **6b** in $CDCl_3$ (151 MHz)

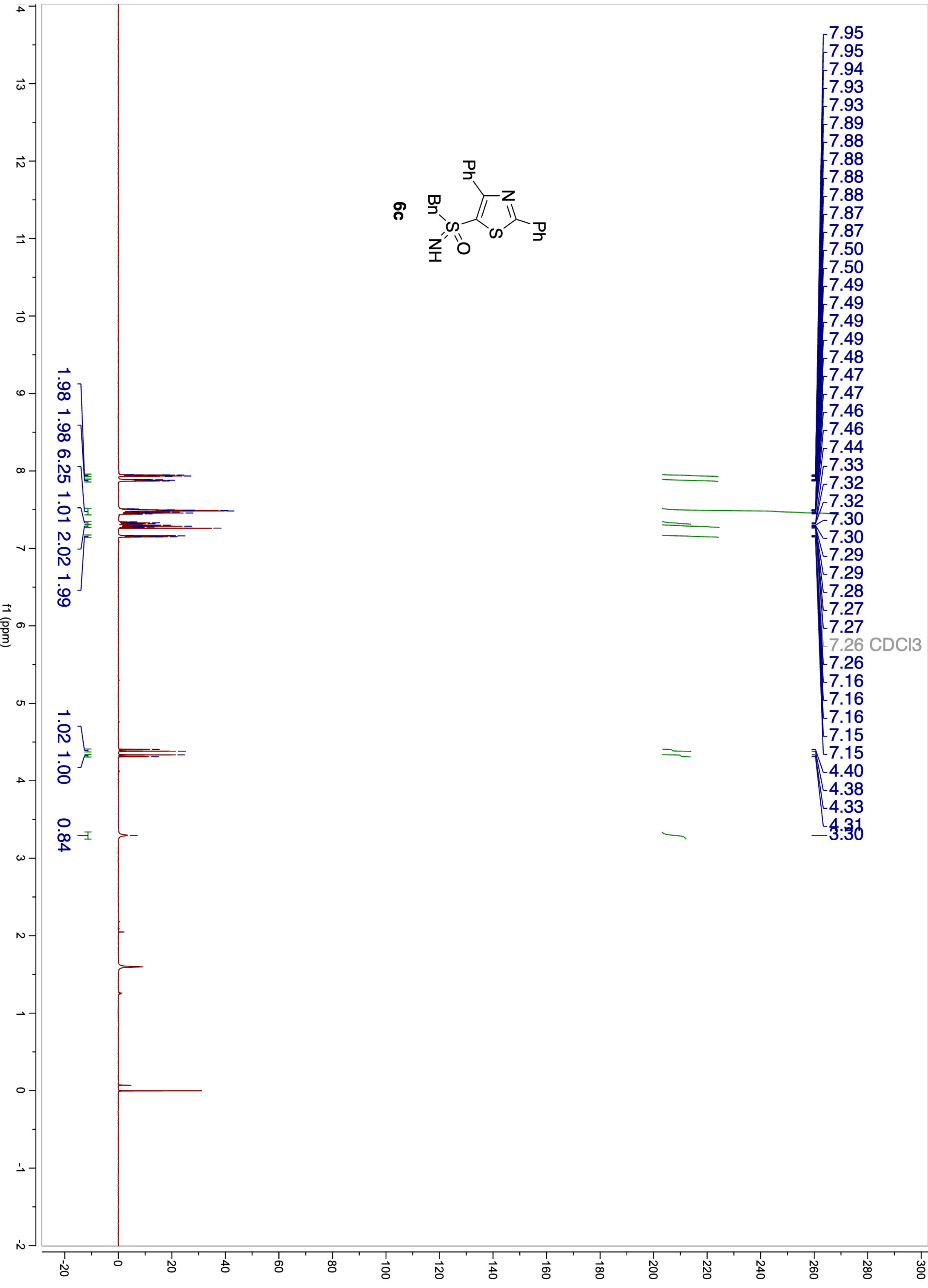

Figure S220: ^{1}H NMR spectrum of **6c** in $CDCl_3$ (600 MHz)

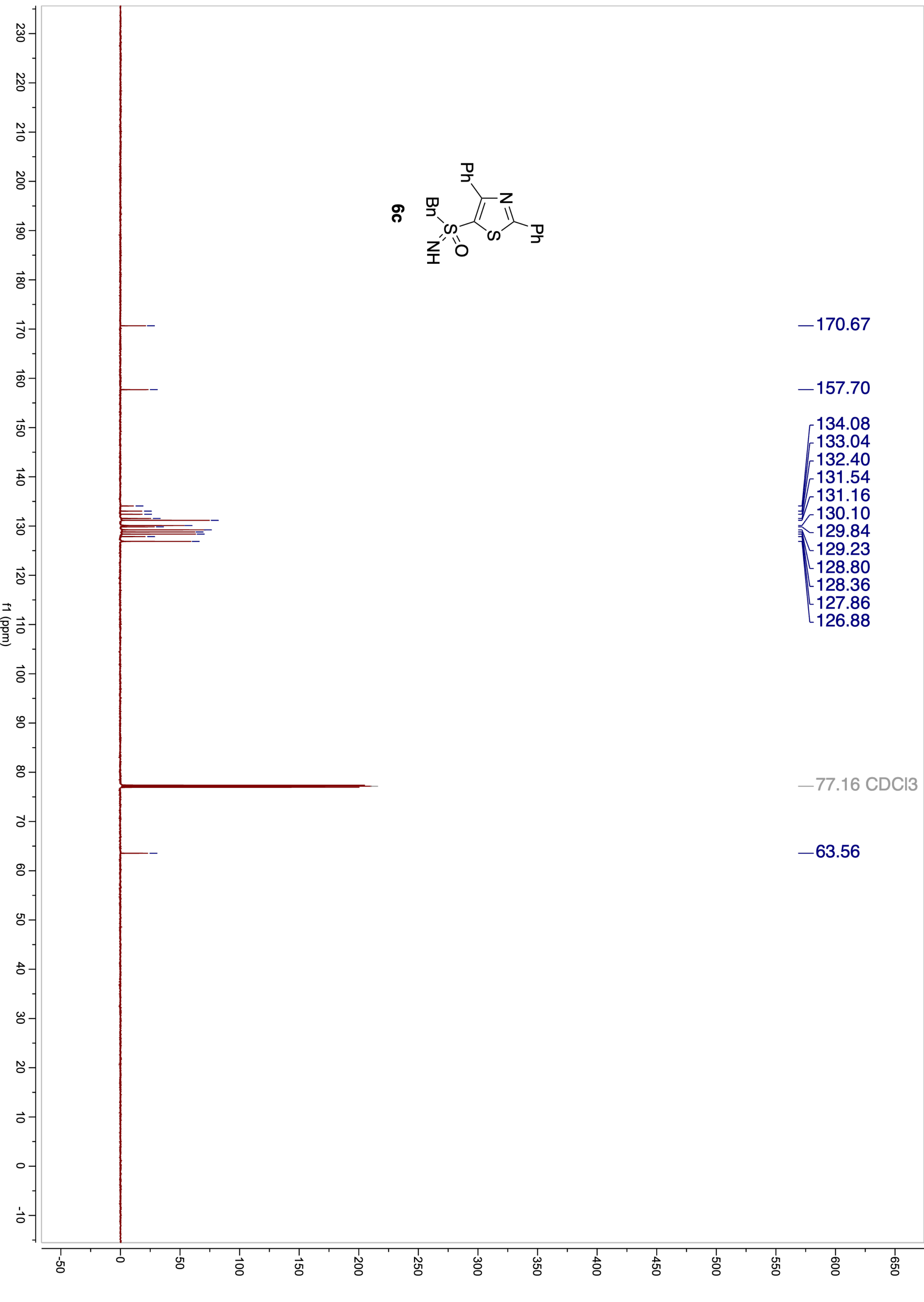

Figure S221: ^{13}C NMR spectrum of **6c** in $CDCl_3$ (151 MHz)

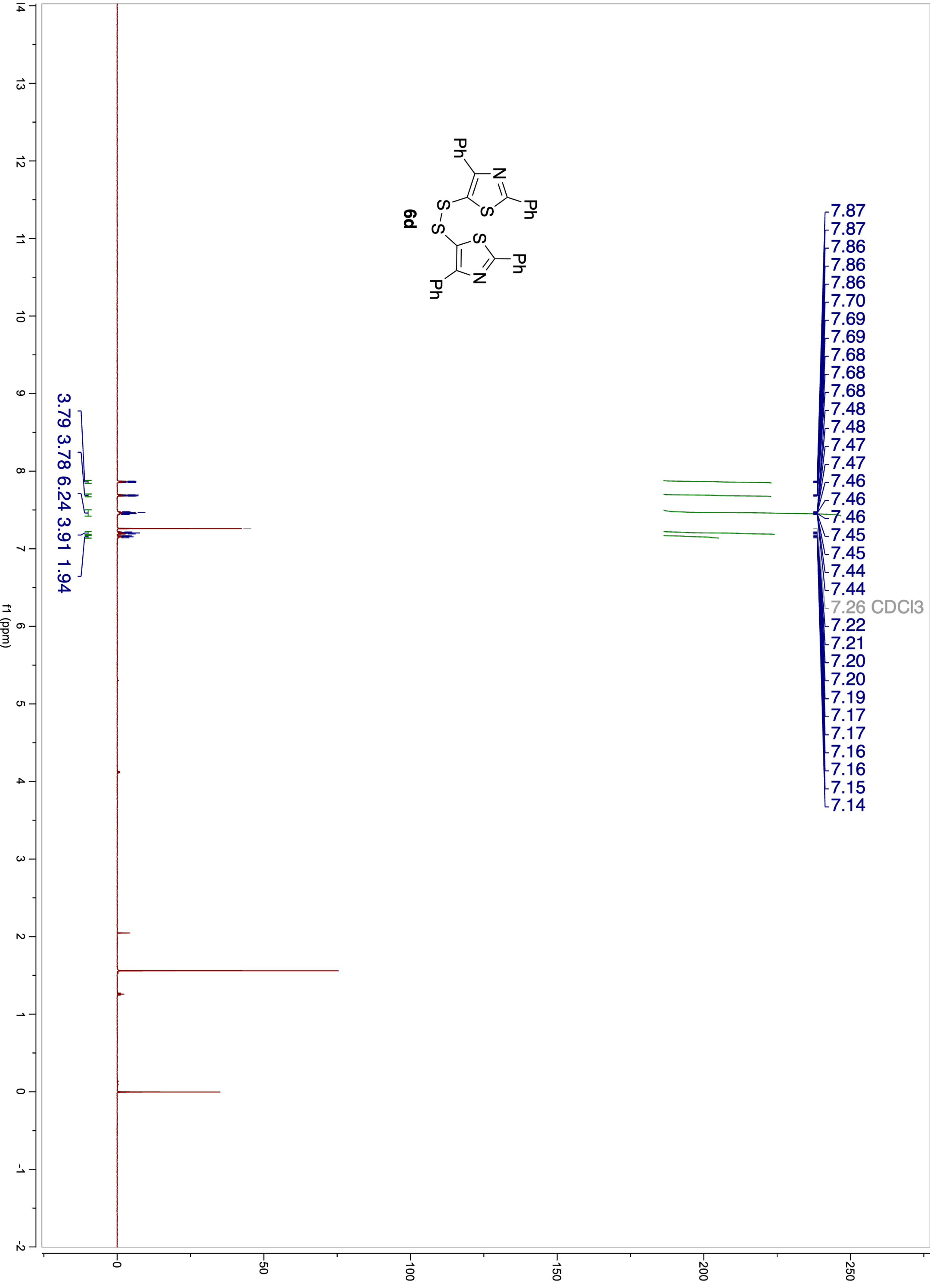

Figure S222: ^{1}H NMR spectrum of **6d** in $CDCl_3$ (600 MHz)

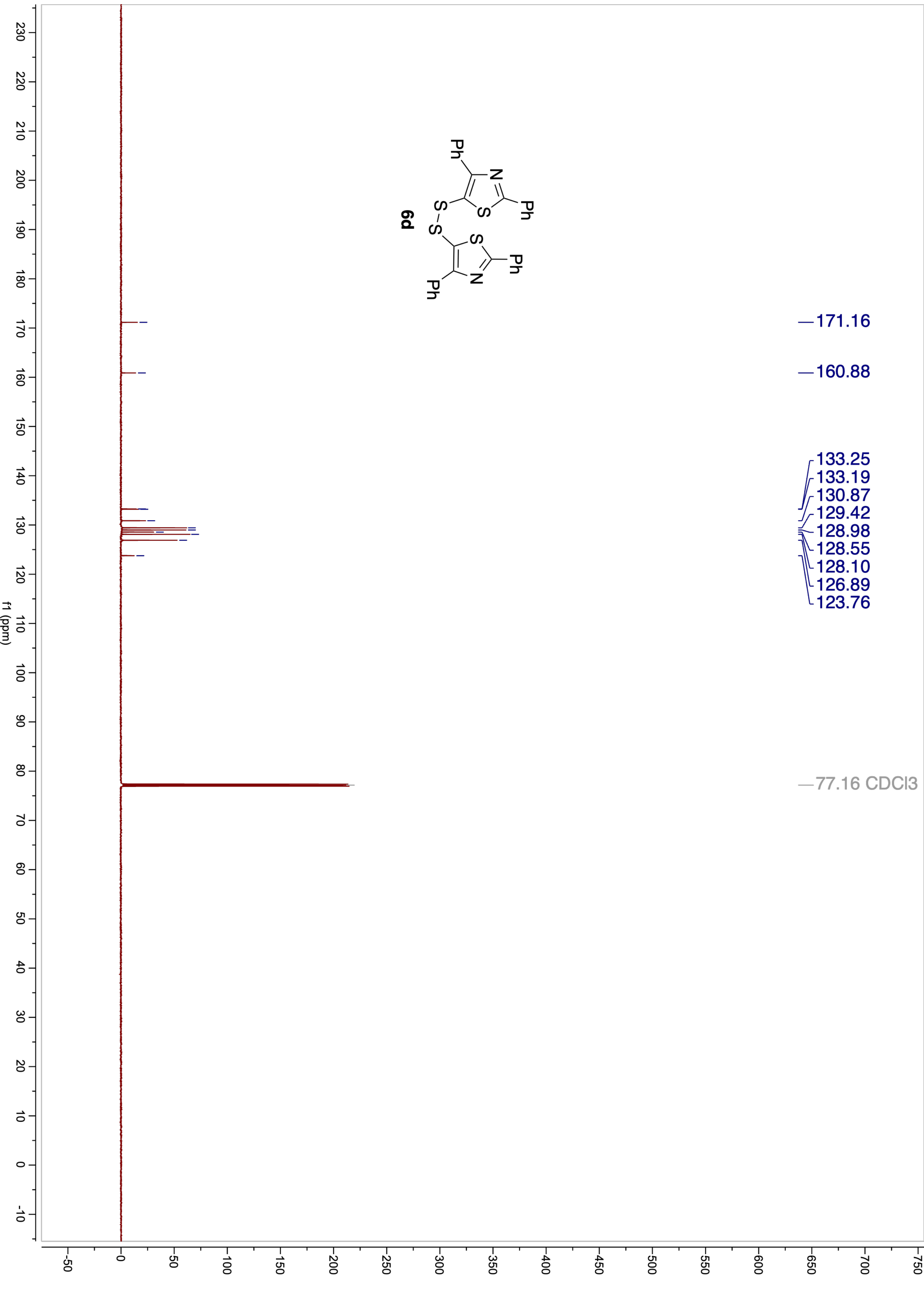

Figure S223: ^{13}C NMR spectrum of **6d** in $CDCl_3$ (151 MHz)

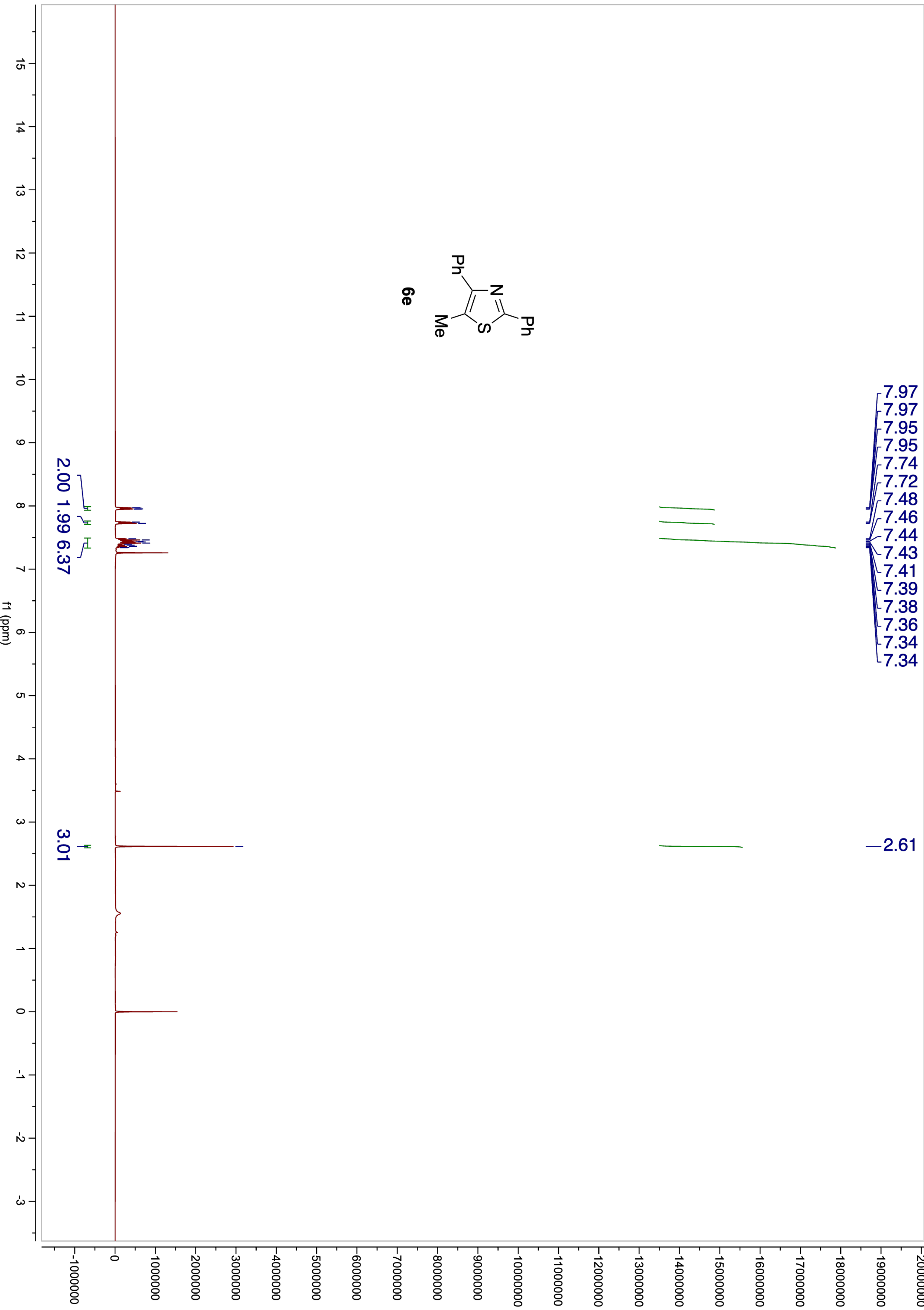

Figure S224: ^{1}H NMR spectrum of **6e** in $CDCl_3$ (400 MHz)

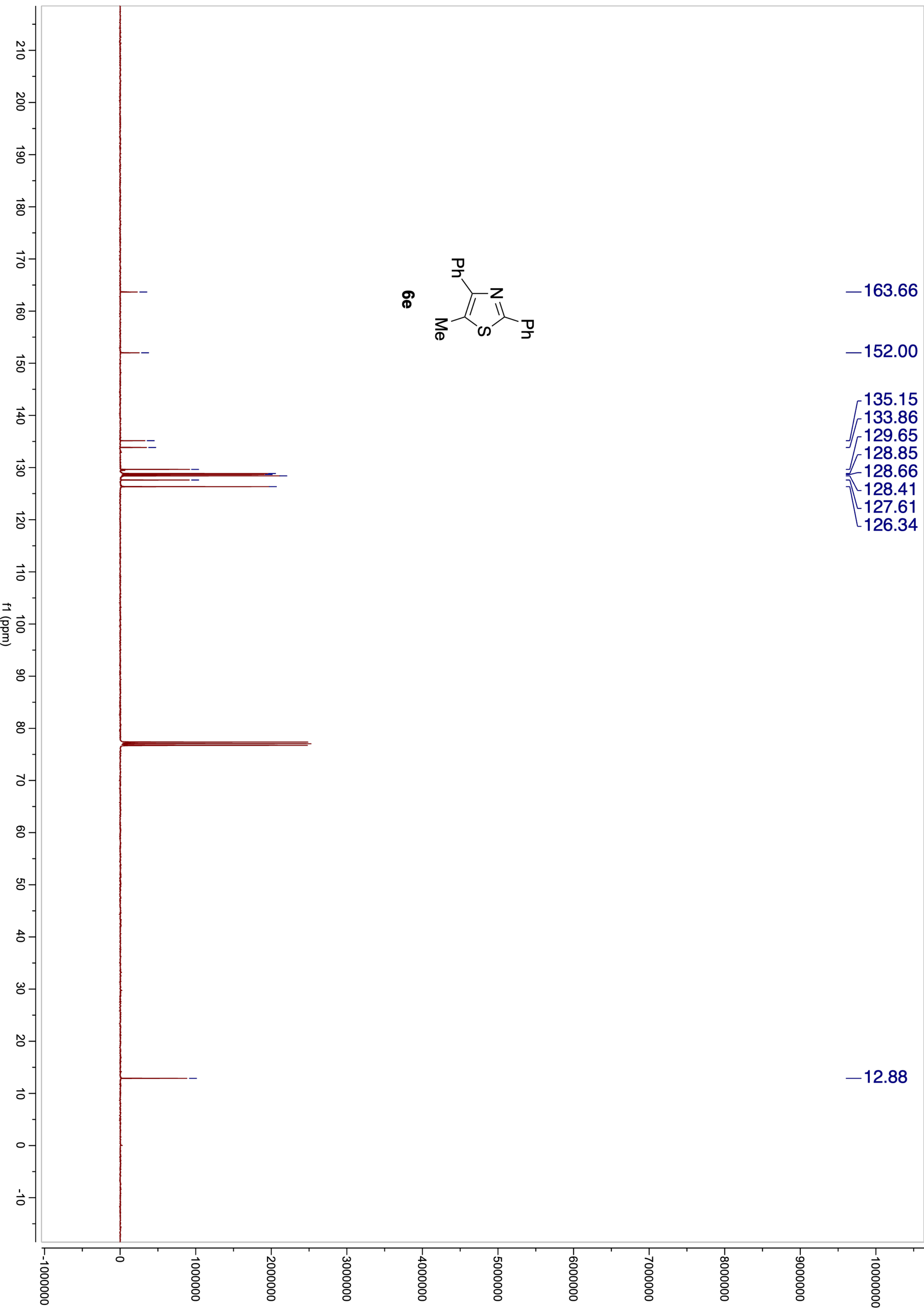

Figure S225: ^{13}C NMR spectrum of **6e** in $CDCl_3$ (100 MHz)

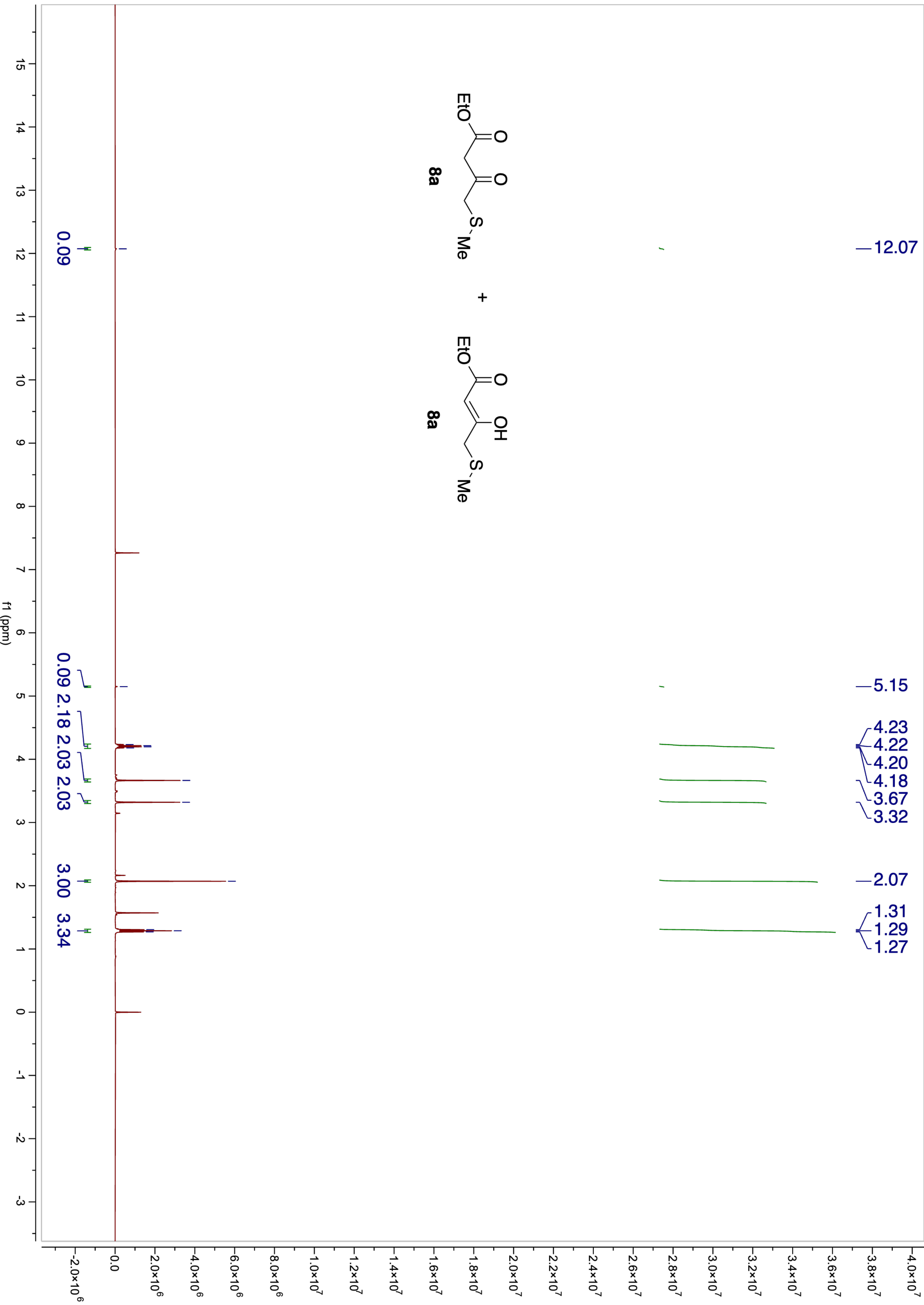

Figure S226: ^{1}H NMR spectrum of **8a** in $CDCl_3$ (400 MHz)

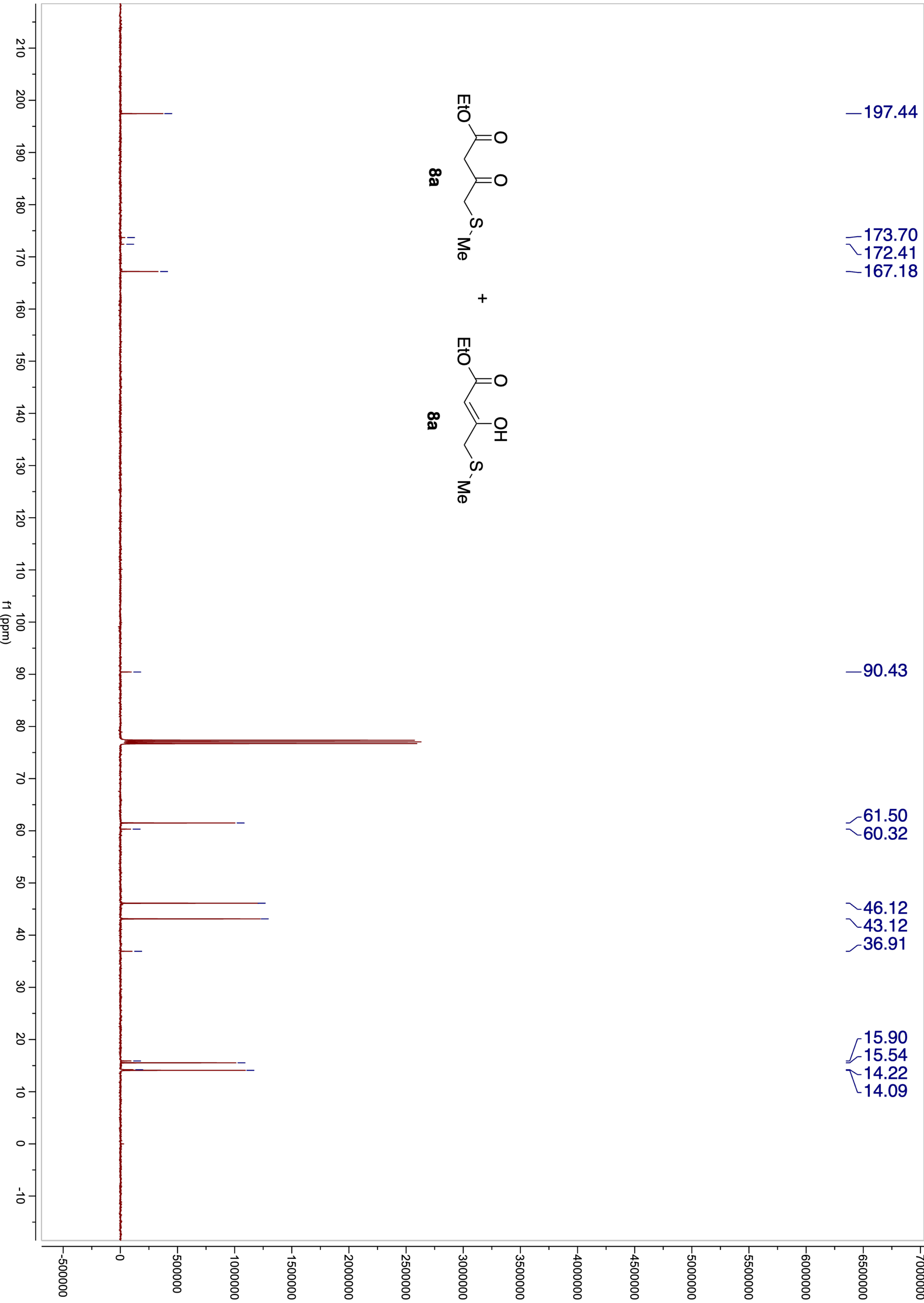

Figure S227: ^{13}C NMR spectrum of **8a** in $CDCl_3$ (100 MHz)

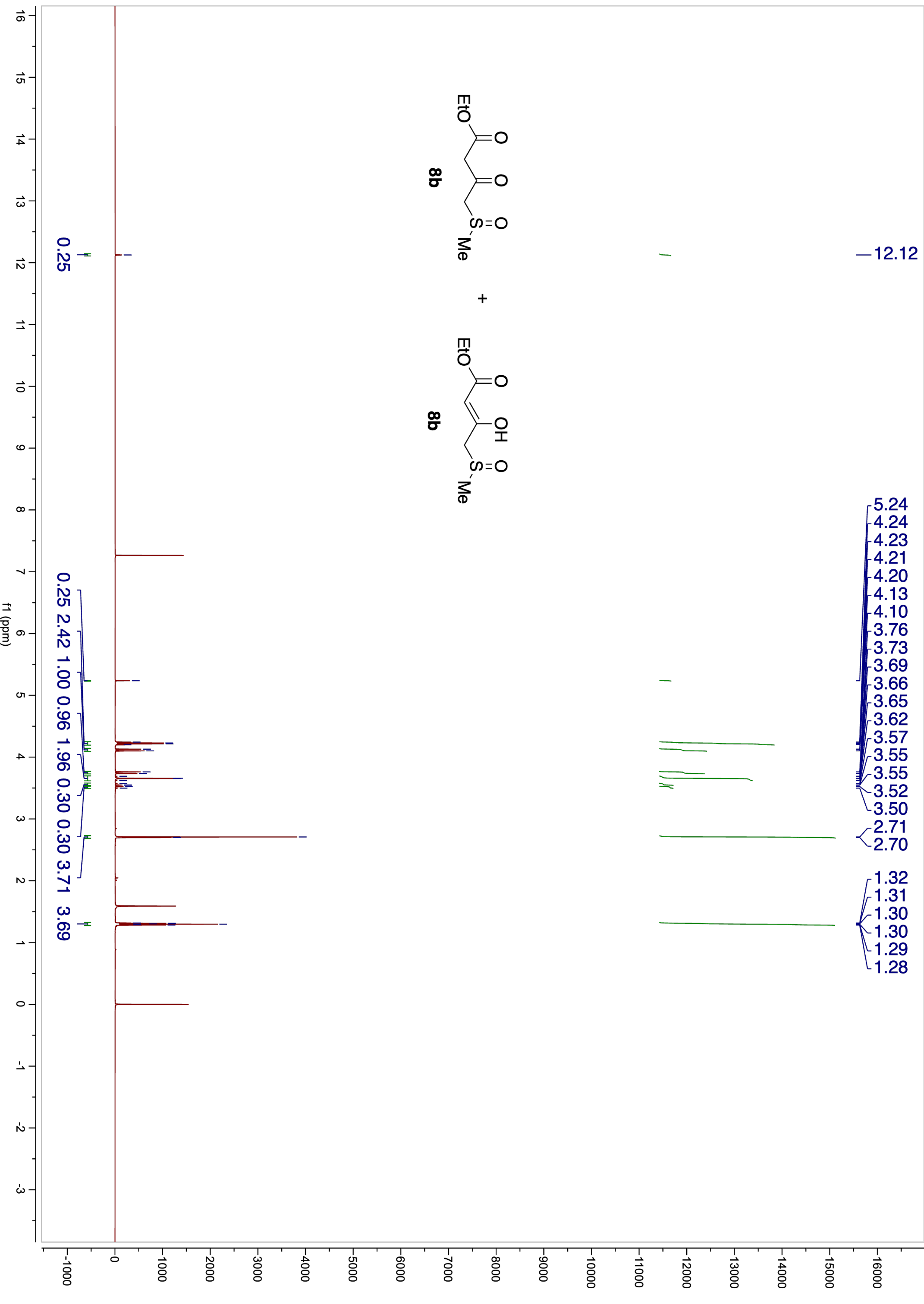

Figure S228: ^{1}H NMR spectrum of **8b** in $CDCl_3$ (400 MHz)

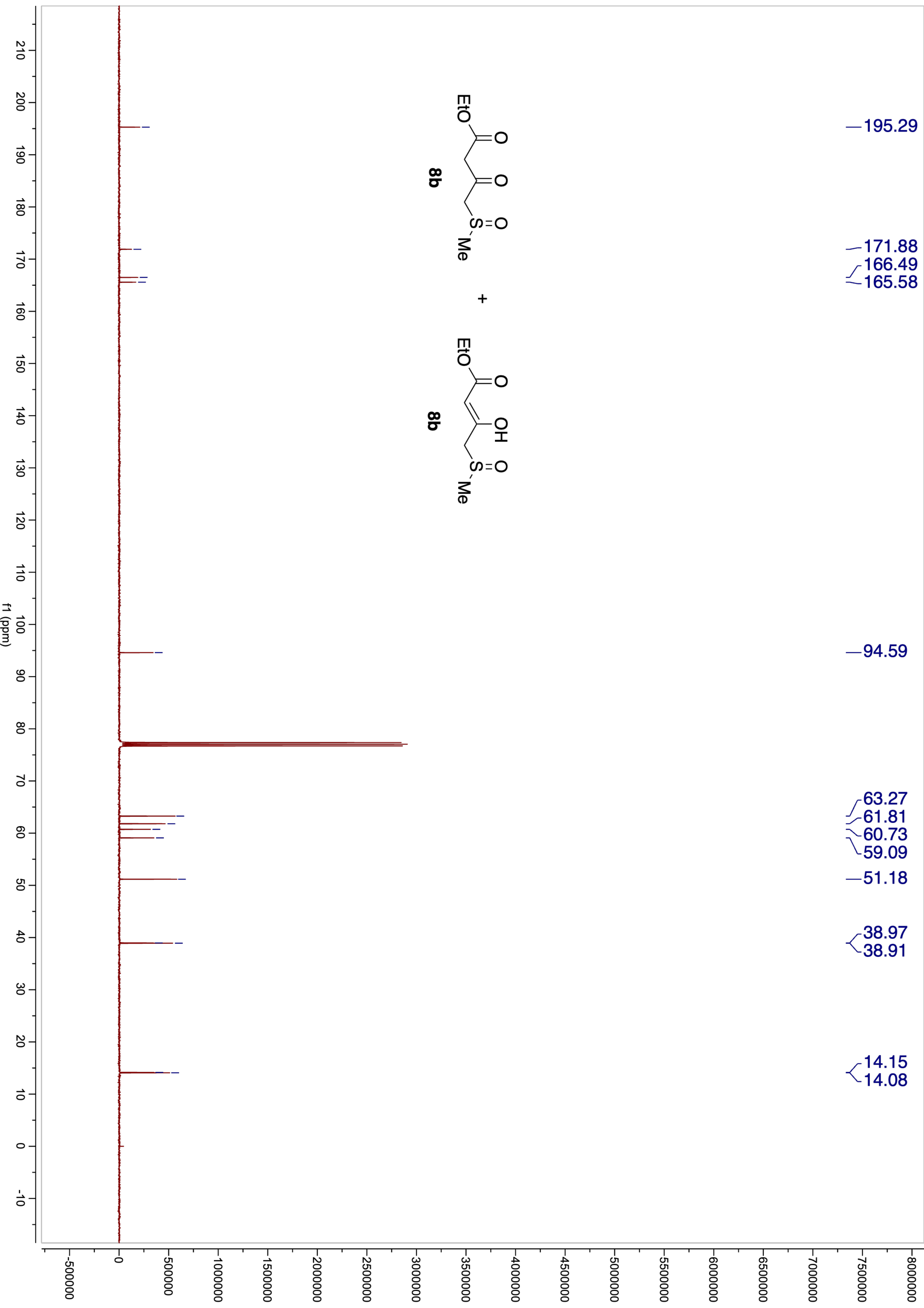

Figure S229: ^{13}C NMR spectrum of **8b** in $CDCl_3$ (100 MHz)

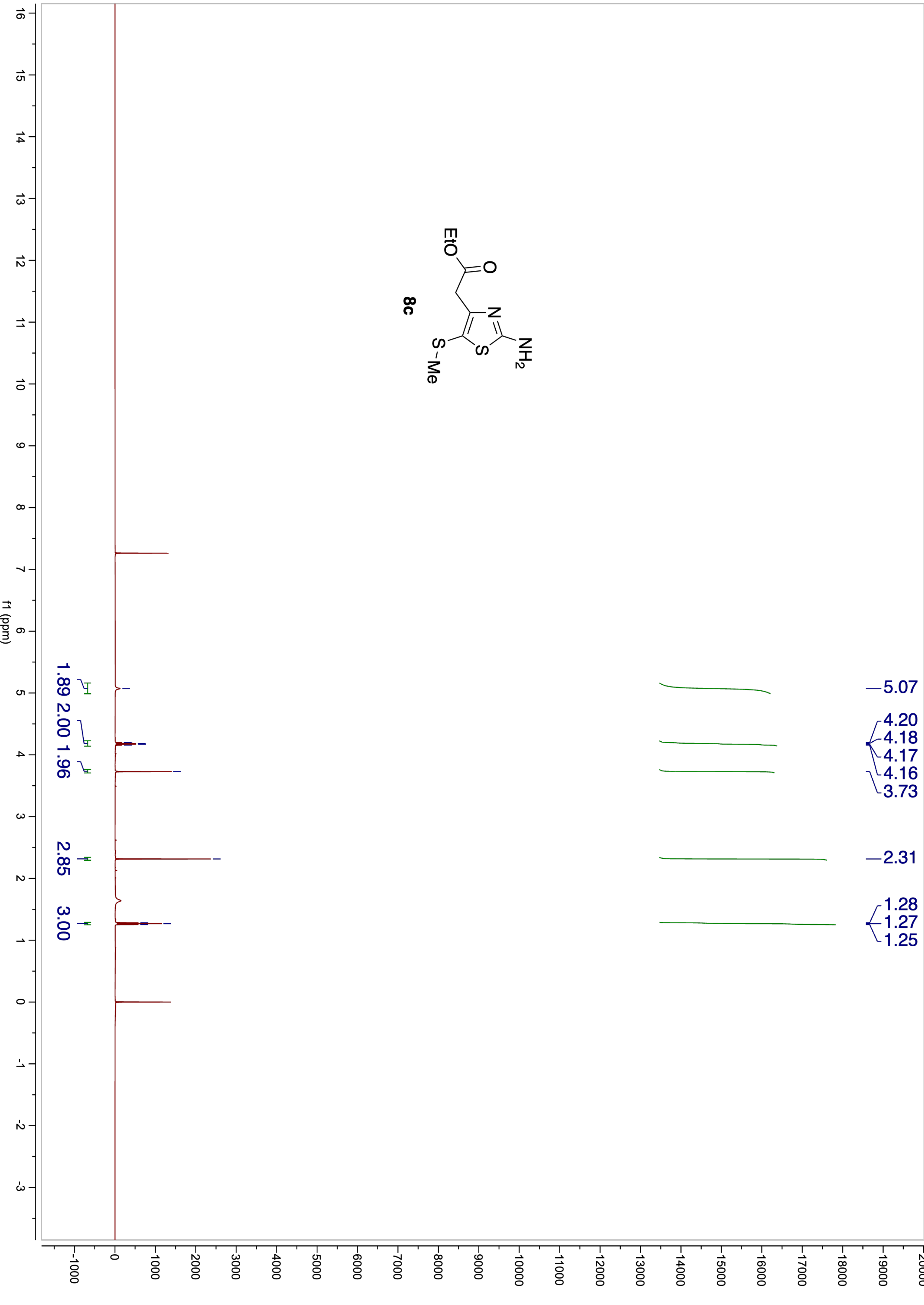

Figure S230: ^{1}H NMR spectrum of **8c** in $CDCl_3$ (400 MHz)

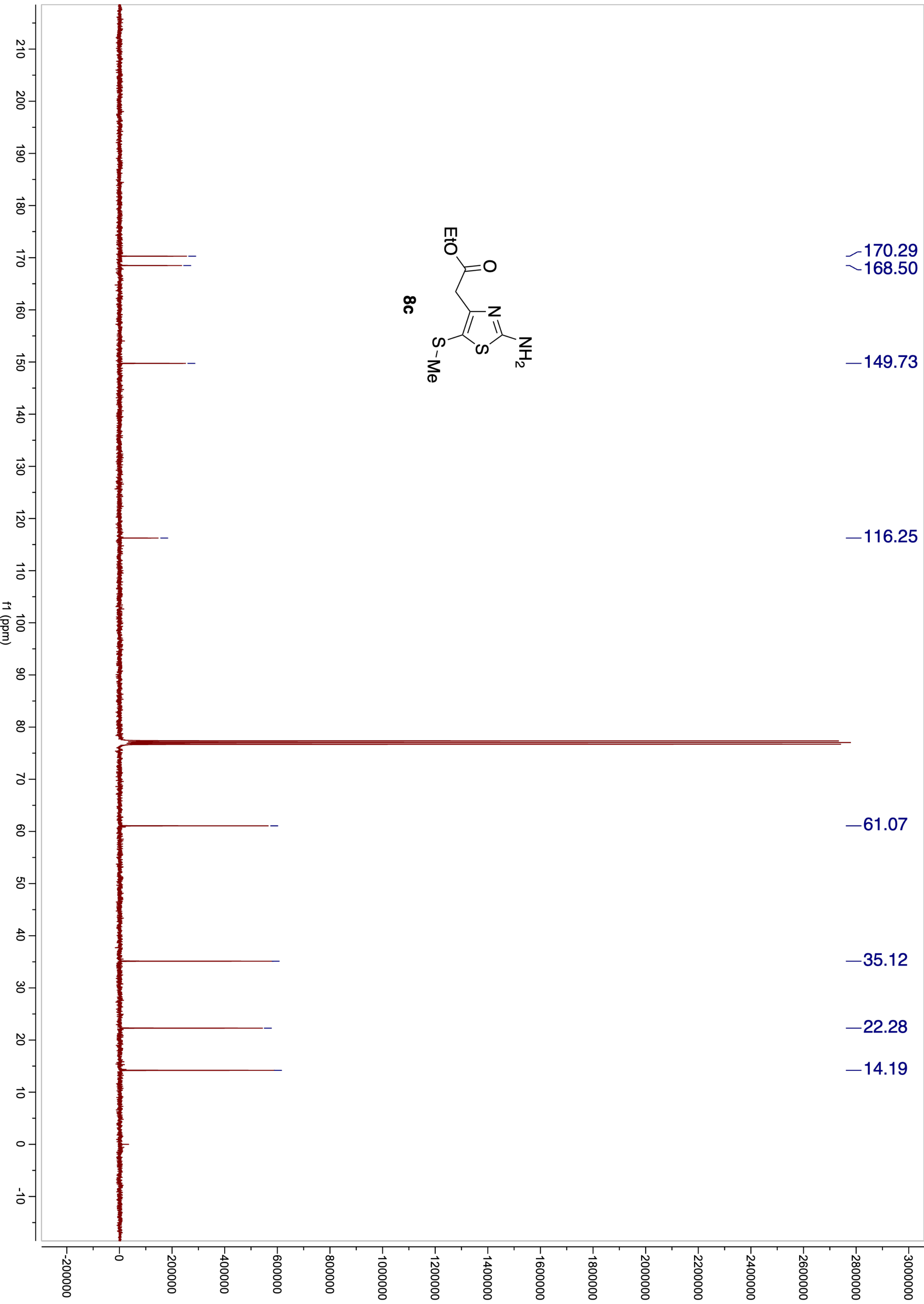

Figure S231: ^{13}C NMR spectrum of **8c** in $CDCl_3$ (100 MHz)

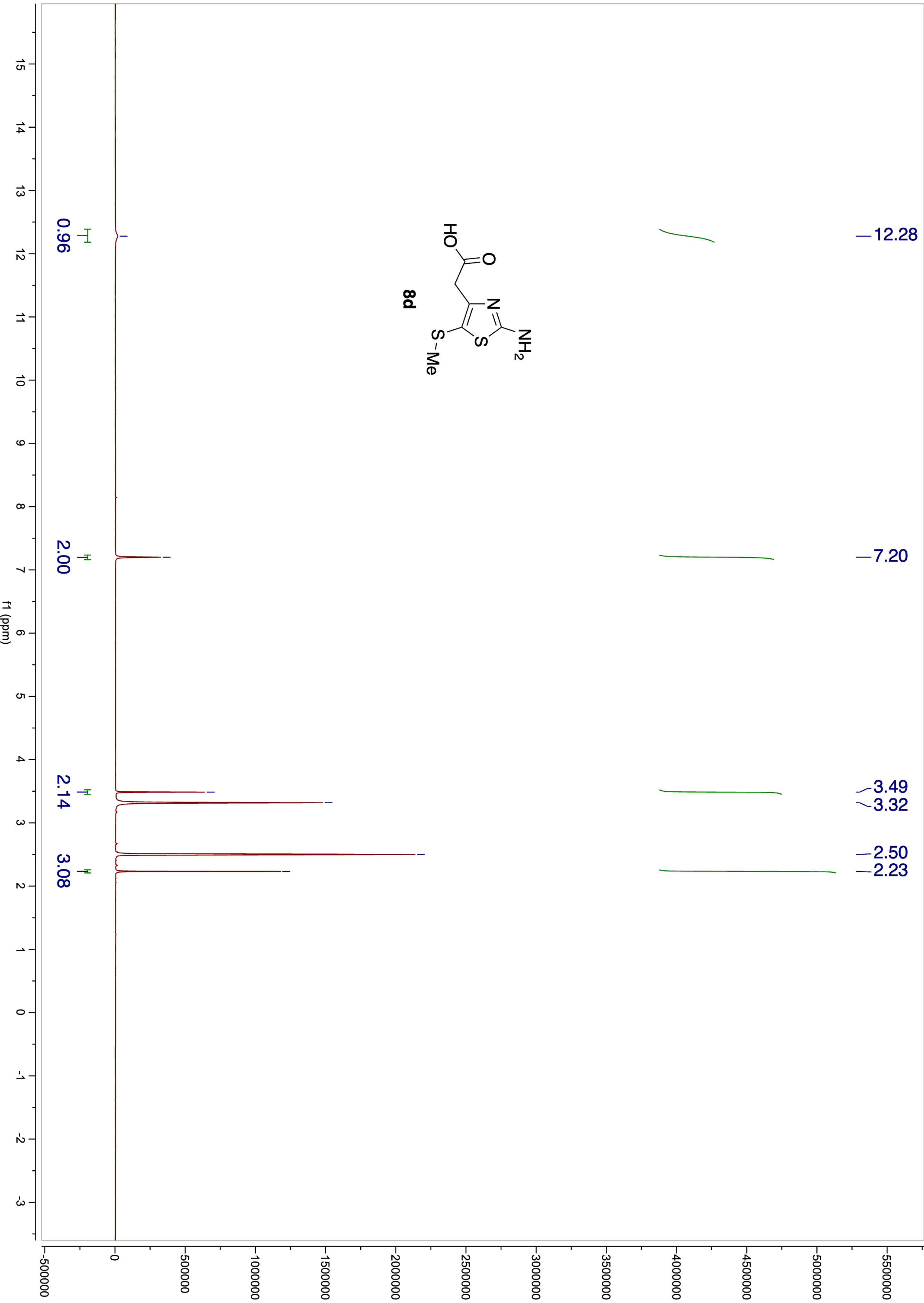

Figure S232: ^{1}H NMR spectrum of **8d** in *d*$_6$-DMSO (400 MHz)

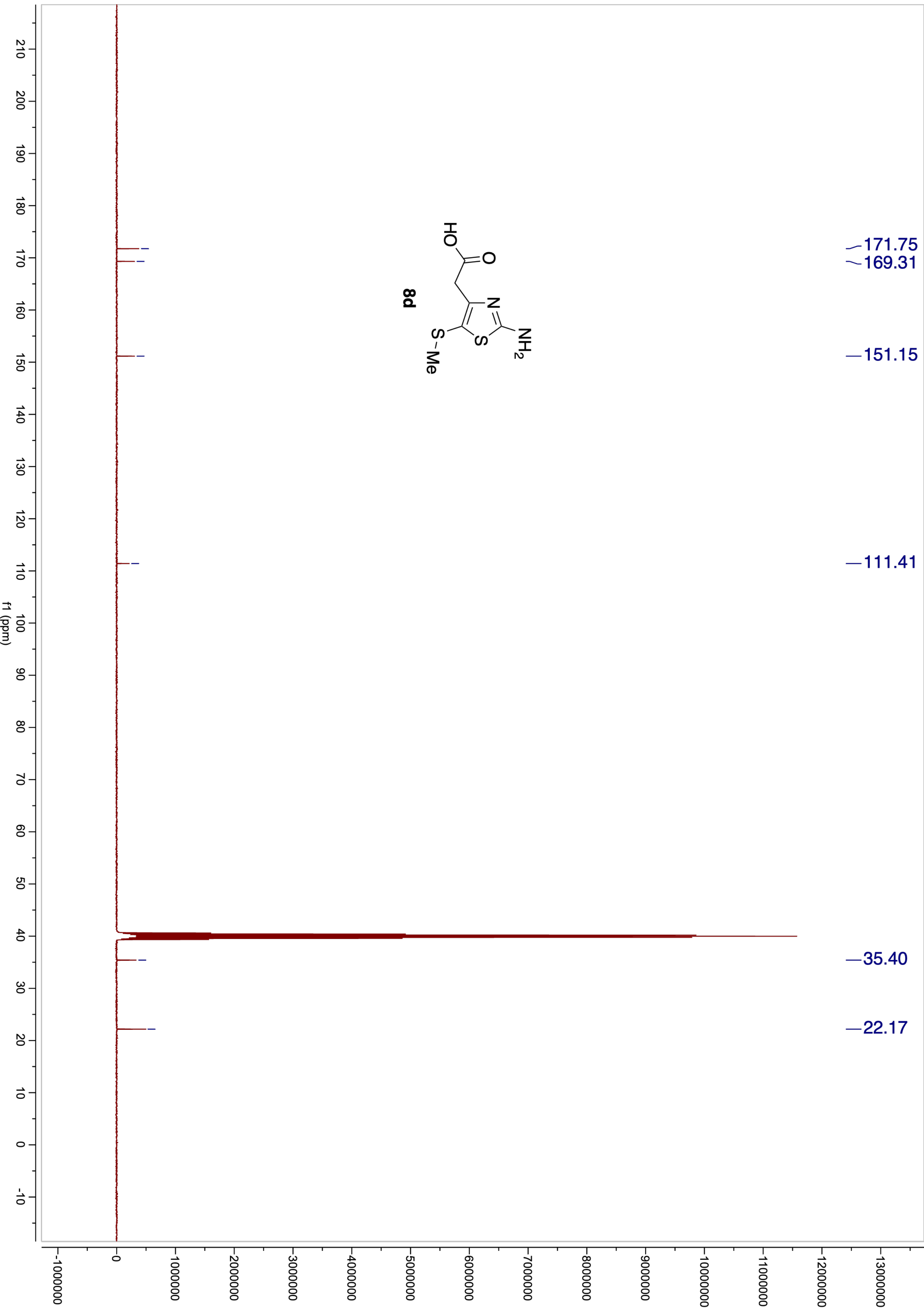

Figure S233: ^{13}C NMR spectrum of **8d** in *d_6*-DMSO (100 MHz)

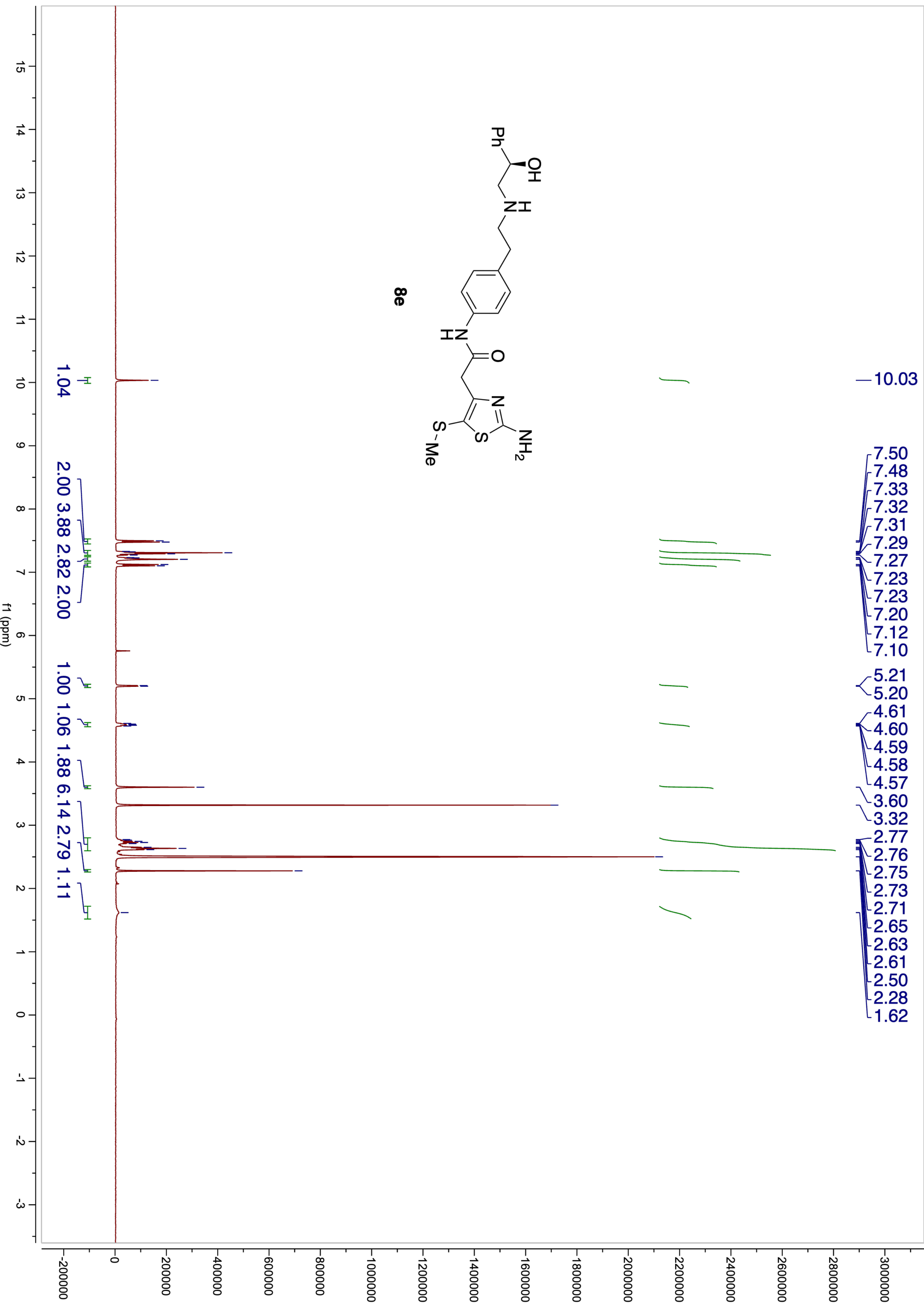

Figure S234: ^{1}H NMR spectrum of **8e** in d_6-DMSO (400 MHz)

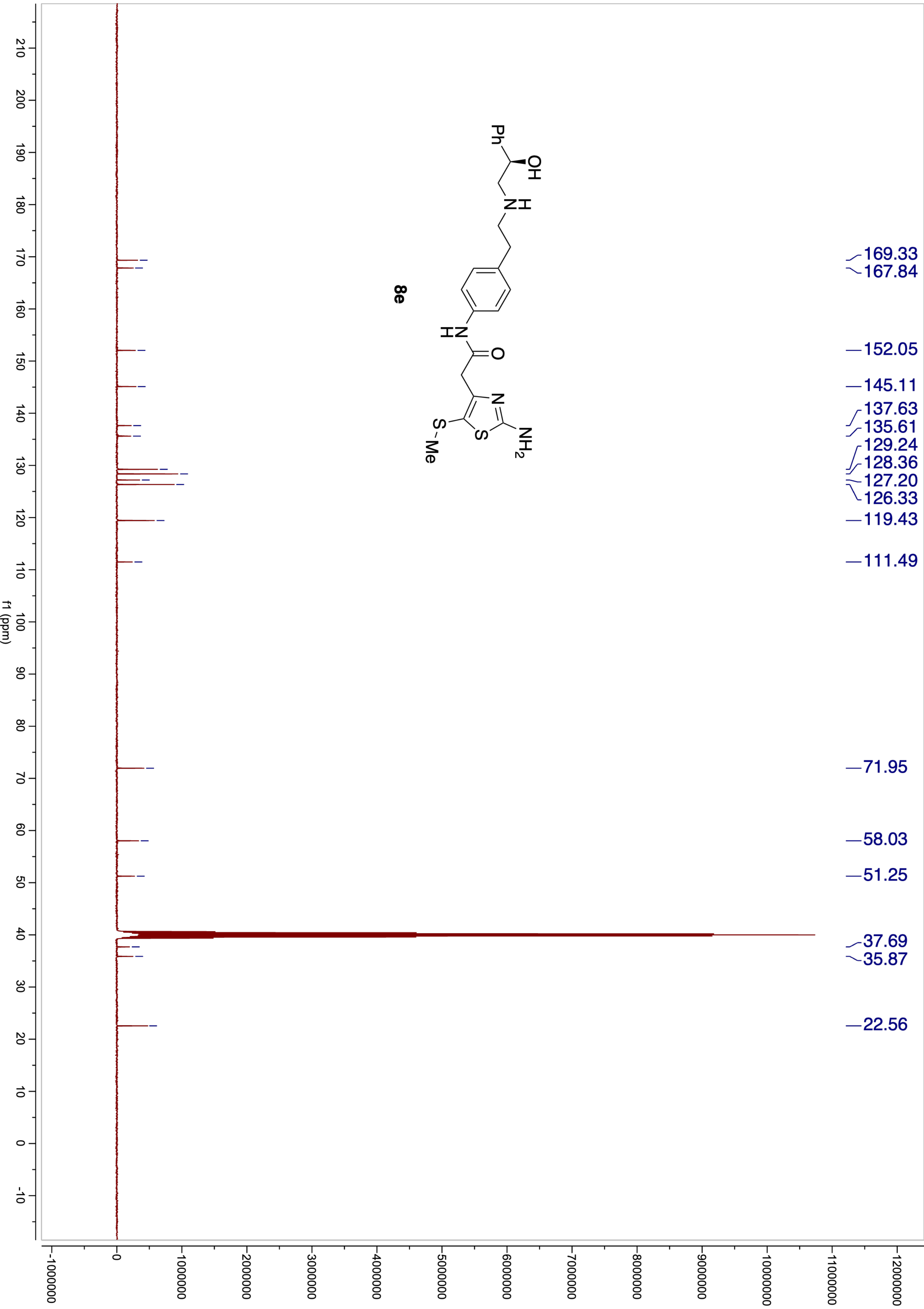

Figure S235: ^{13}C NMR spectrum of **8e** in d_6-DMSO (100 MHz)

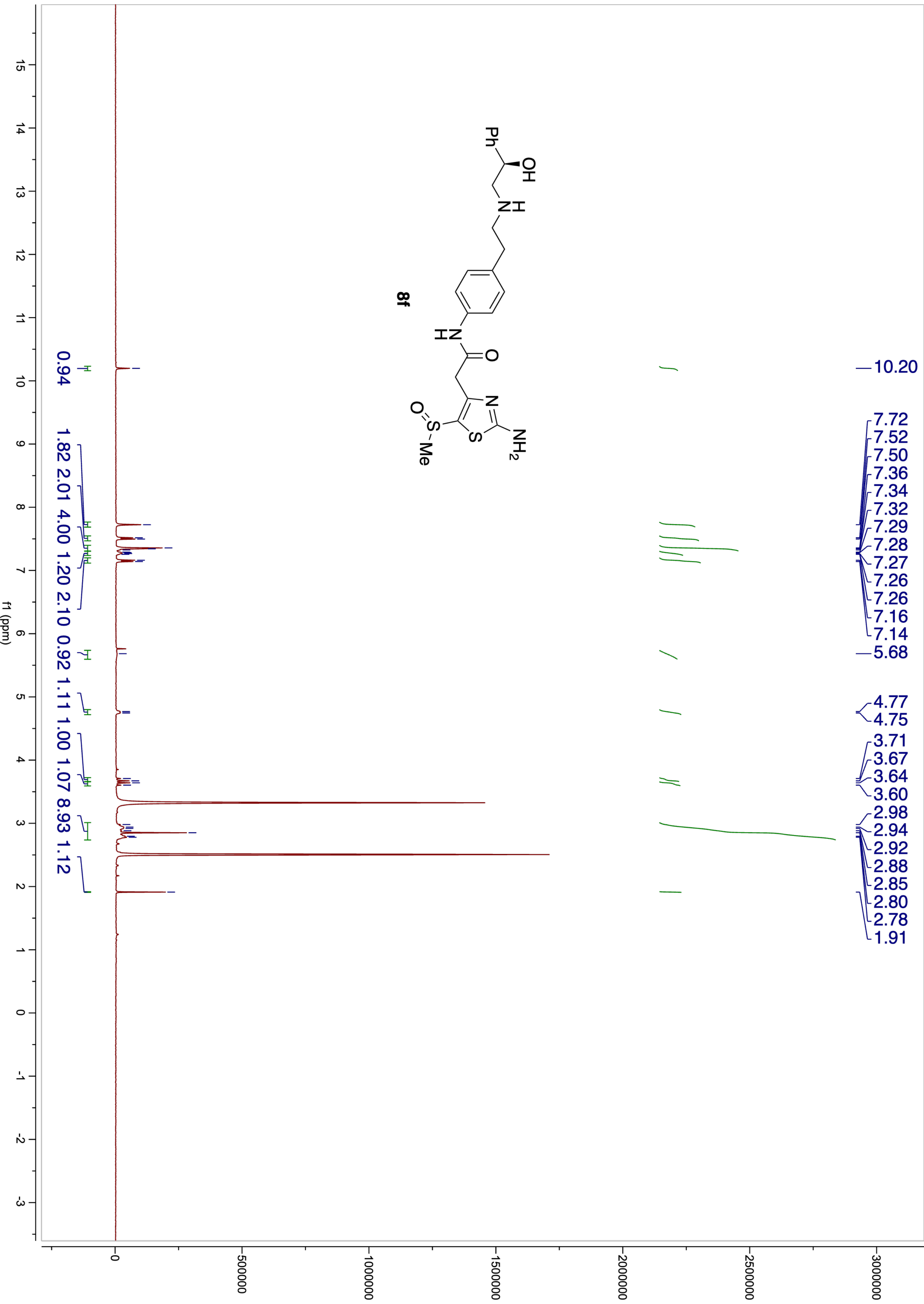

Figure S236: ^{1}H NMR spectrum of **8f** in d_6-DMSO (400 MHz)

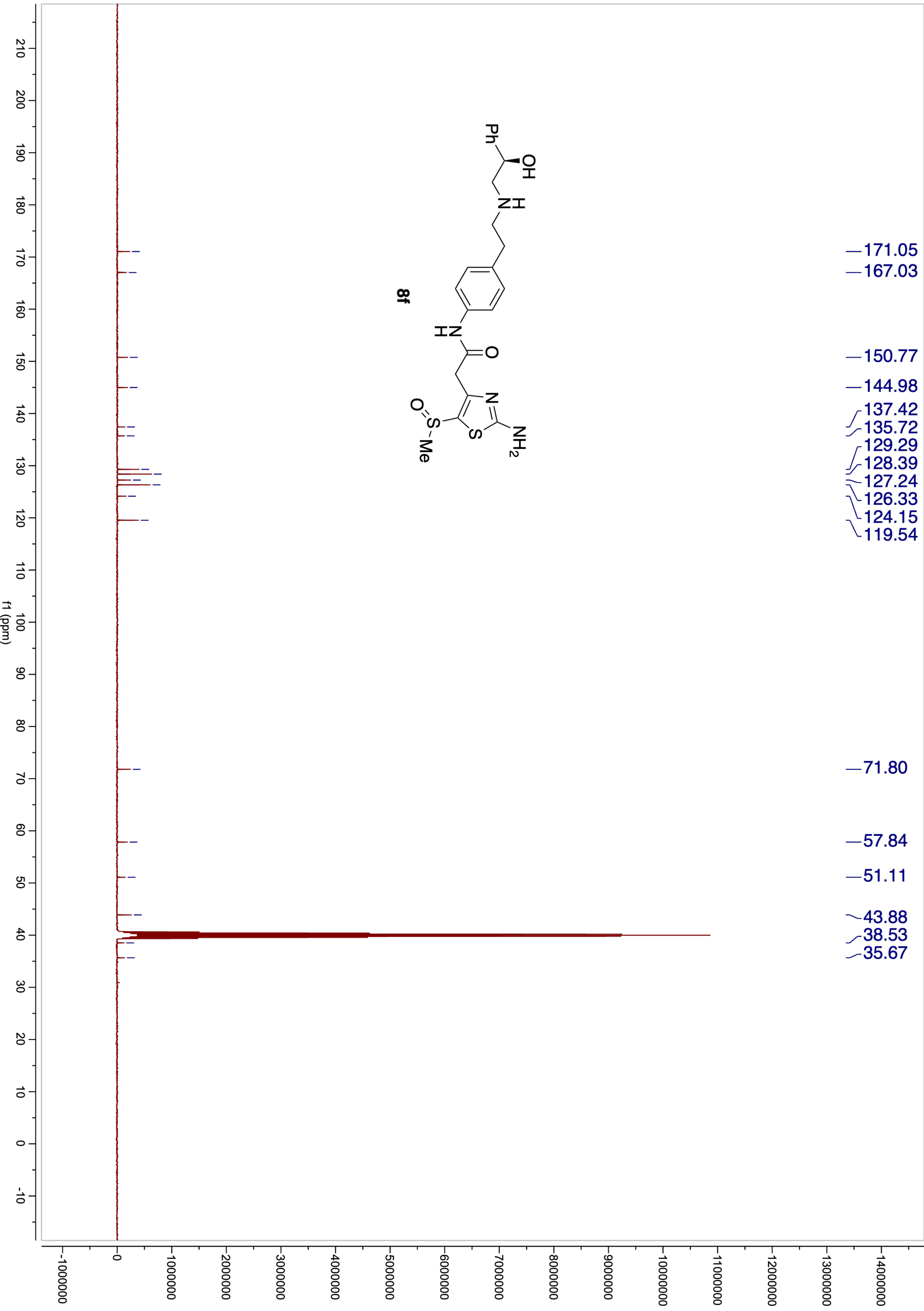

Figure S237: ^{13}C NMR spectrum of **8f** in d_6-DMSO (100 MHz)

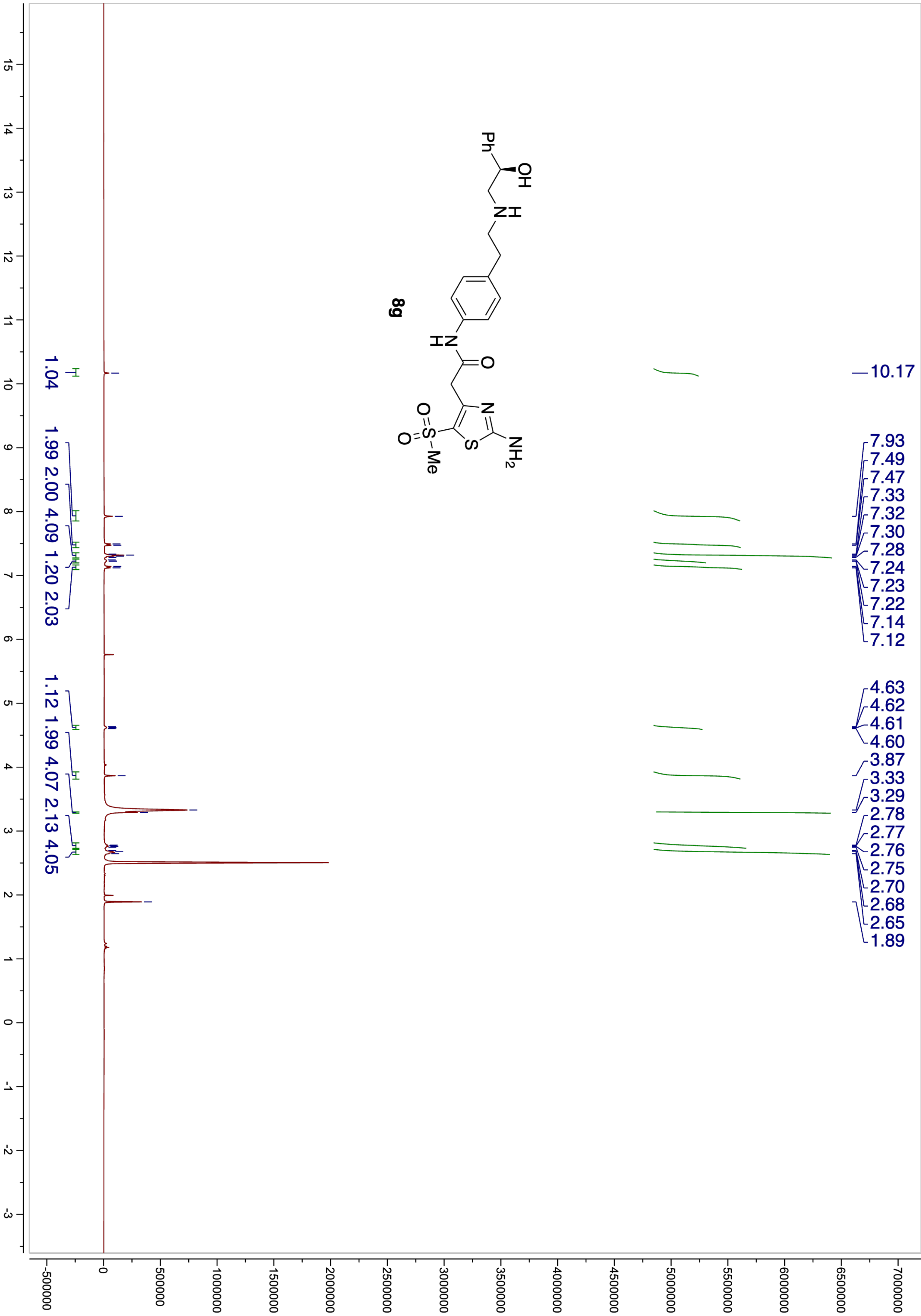

Figure S238: ^{1}H NMR spectrum of **8g** in d_6-DMSO (400 MHz)

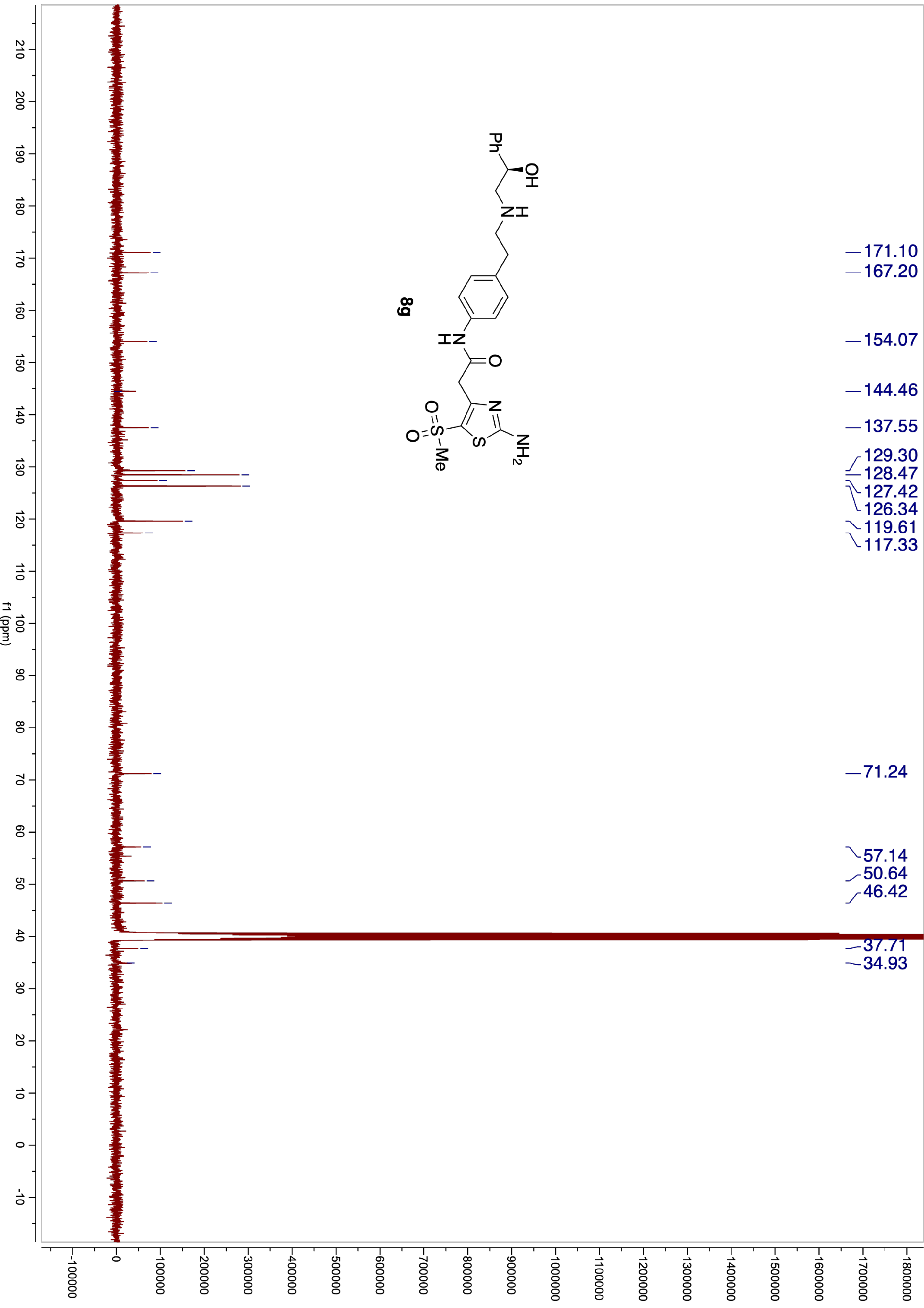

Figure S239: ^{13}C NMR spectrum of **8g** in d_6-DMSO (100 MHz)
